# Supplementary material for: The Medaka Inbred Kiyosu-Karlsruhe (MIKK) panel
Source: Genome Biol. 2022 Feb 21;23:59. doi: 10.1186/s13059-022-02623-z (PMC8862526; doi:10.1186/s13059-022-02623-z)
Supplement: Supplementary file 4 — Additional file 4: Figures S1-S8. Supplementary figures. Various supplementary figures. [file 13059_2022_2623_MOESM4_ESM.docx]

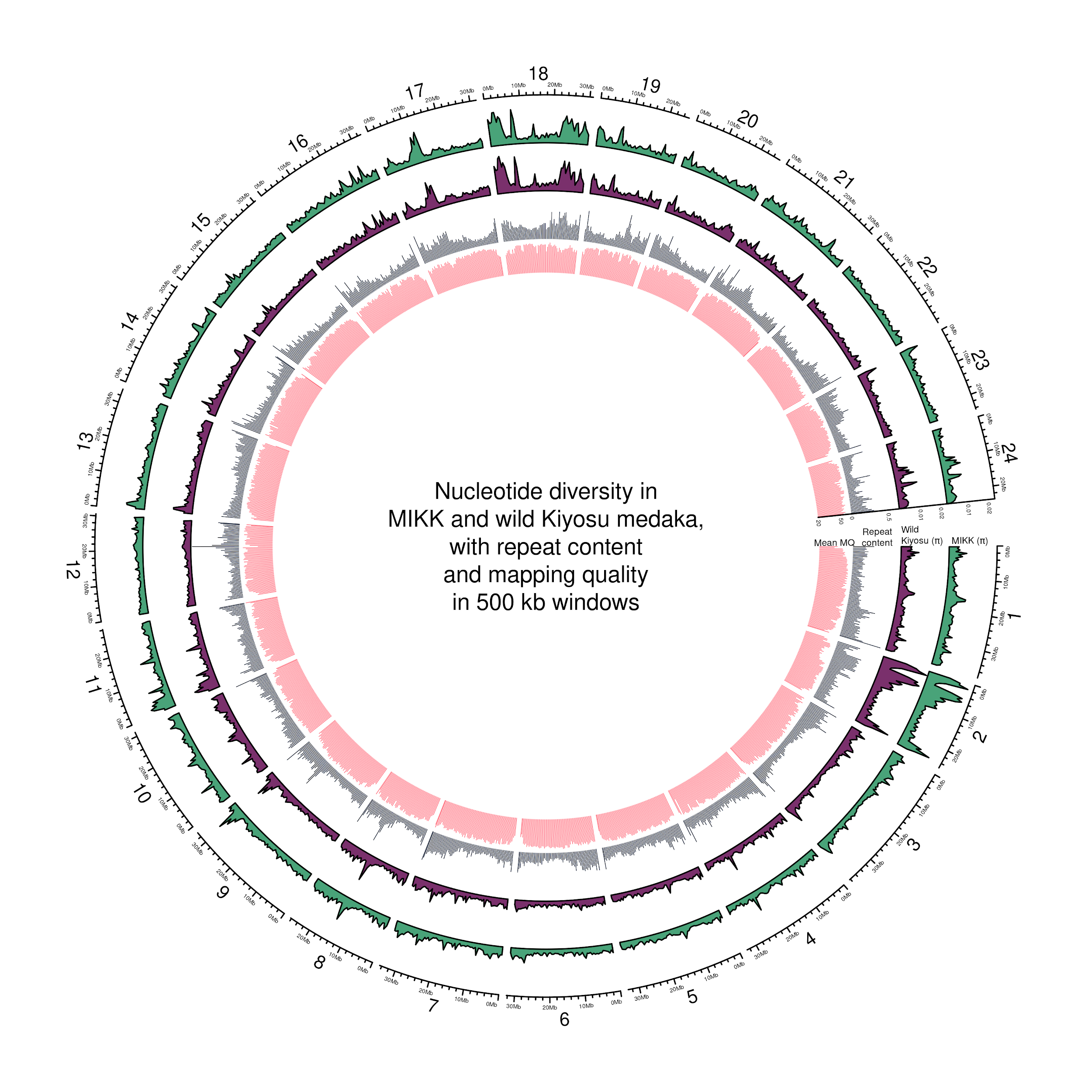


**Figure S1:** *Circos plot with nucleotide diversity (*$\hat{\pi}$*) calculated within 500-kb non-overlapping windows for 63 non-“sibling” lines from the MIKK panel (green) and 7 wild Kiyosu medaka samples from the same originating population (purple); proportion of sequence classified as repeats by RepeatMasker (blue); and mean mapping quality (pink).*


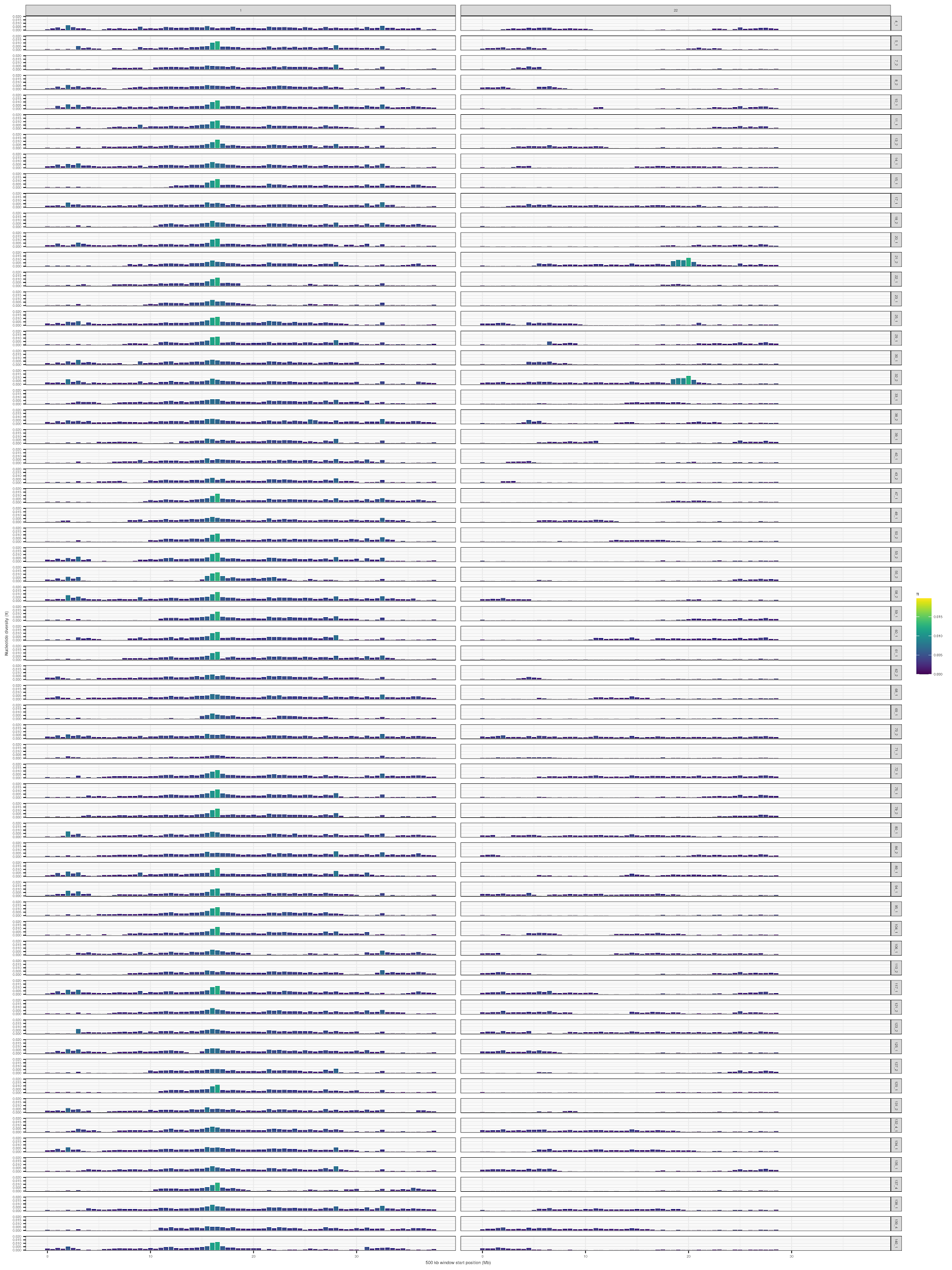


**Figure S2:** *Nucleotide diversity (*$\hat{\pi}$*) calculated within 500-kb non-overlapping windows for each of the 63 non-“sibling” lines from the MIKK panel individually, for chromosome 1 (left) and 22 (right). The windows that include the medaka sex determination region (1:~16-17 Mb) show consistent elevated nucleotide diversity across most lines.*

*
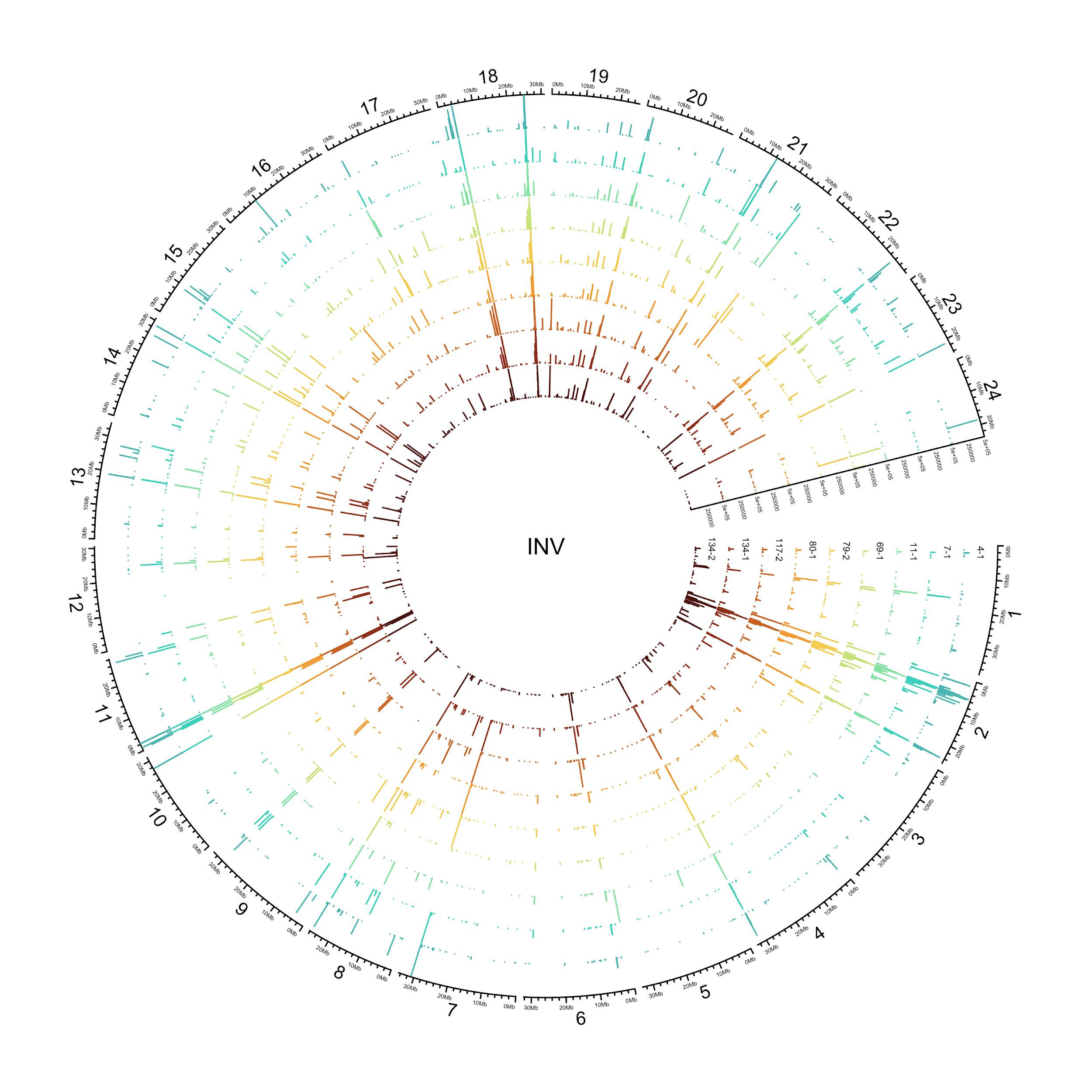
*

**Figure S3:** *Inversions identified in 9 MIKK panel lines using a combination of Oxford Nanopore Technologies long-read and Illumina short-read sequences (see companion paper).*

*
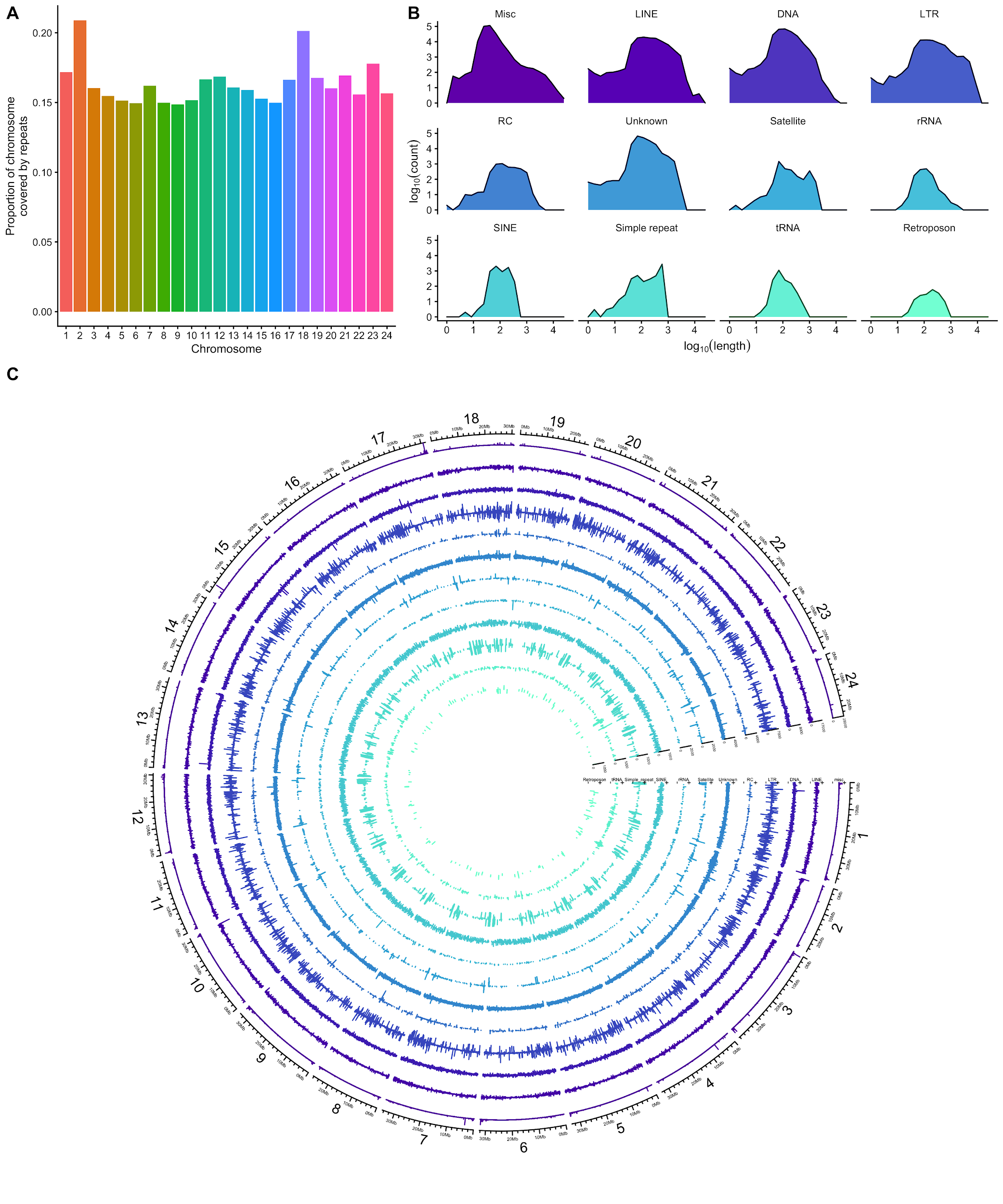
*

**Figure S4:** *Repeat content in the* HdrR *genome based on RepeatMasker results (****Methods****).* ***A****. Proportion of repeat content per-chromosome.* ***B****. log_10_ of repeat lengths and counts per repeat class. “Misc” includes all repeats assigned to their own specific class, for example “(GAG)n” or “(GATCCA)n”.* ***C****. Circos plot showing repeat length (radial axes) by locus (angular axis) and repeat class (track). The code and methods used to generate the figure are set out here:* [https://birneylab.github.io/MIKK_genome_main_paper/05_Repeats.html](https://birneylab.github.io/MIKK_genome_main_paper/20210409_repeats.html).


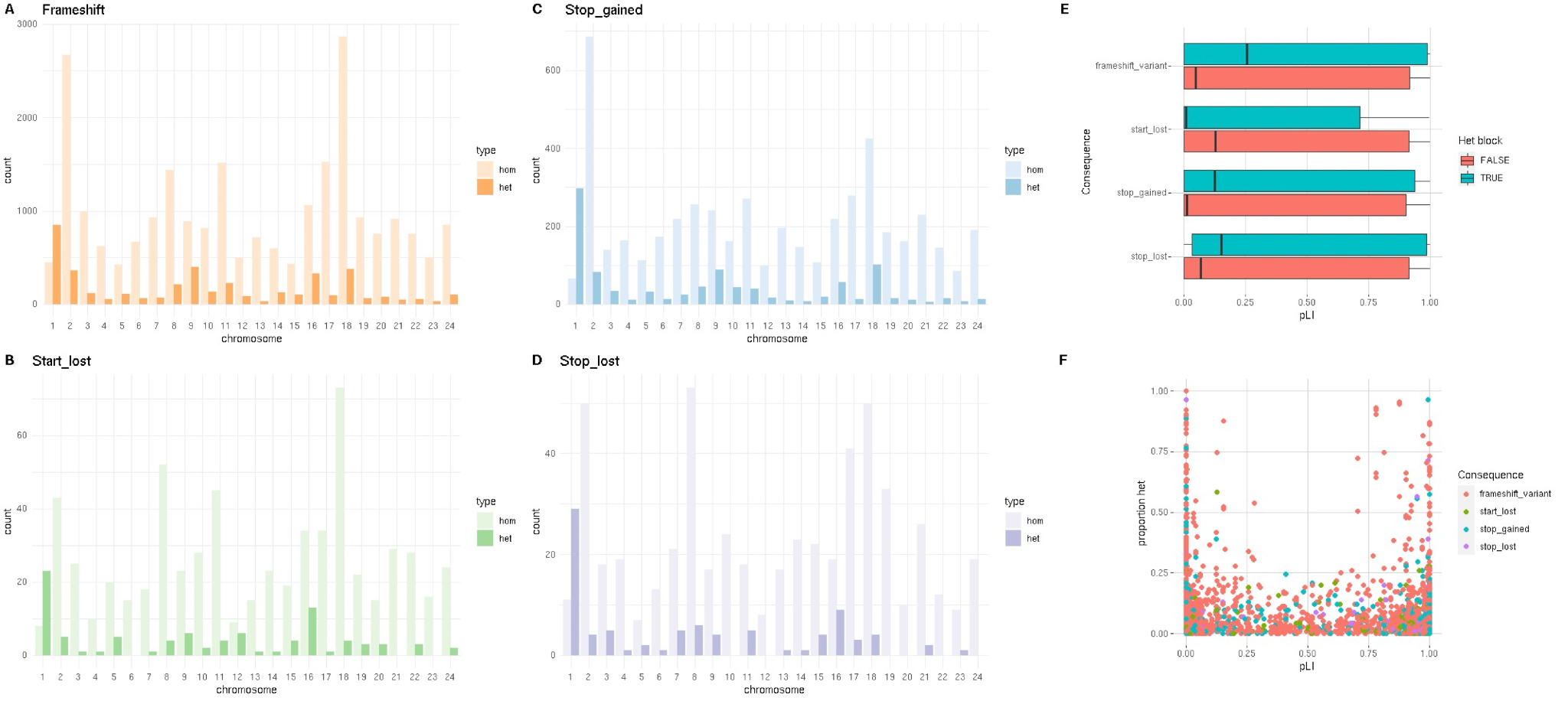


**Figure S5**: *High-confidence loss-of-function variants in the MIKK panel.* ***A****: Frameshift mutations per chromosome within and outside heterozygous blocks.* ***B****: Start-lost mutations per chromosome within and outside heterozygous blocks.* ***C****: Stop-gained mutations per chromosome in and outside heterozygous blocks.* ***D****: Stop-lost mutations per chromosome within and outside heterozygous blocks.* ***E****: Variant class against purifying selection (pLI) within and outside heterozygous blocks.* ***F****: Purifying selection (pLI) against the proportion of genotypes in MIKK panel lines that are heterozygous.*

*
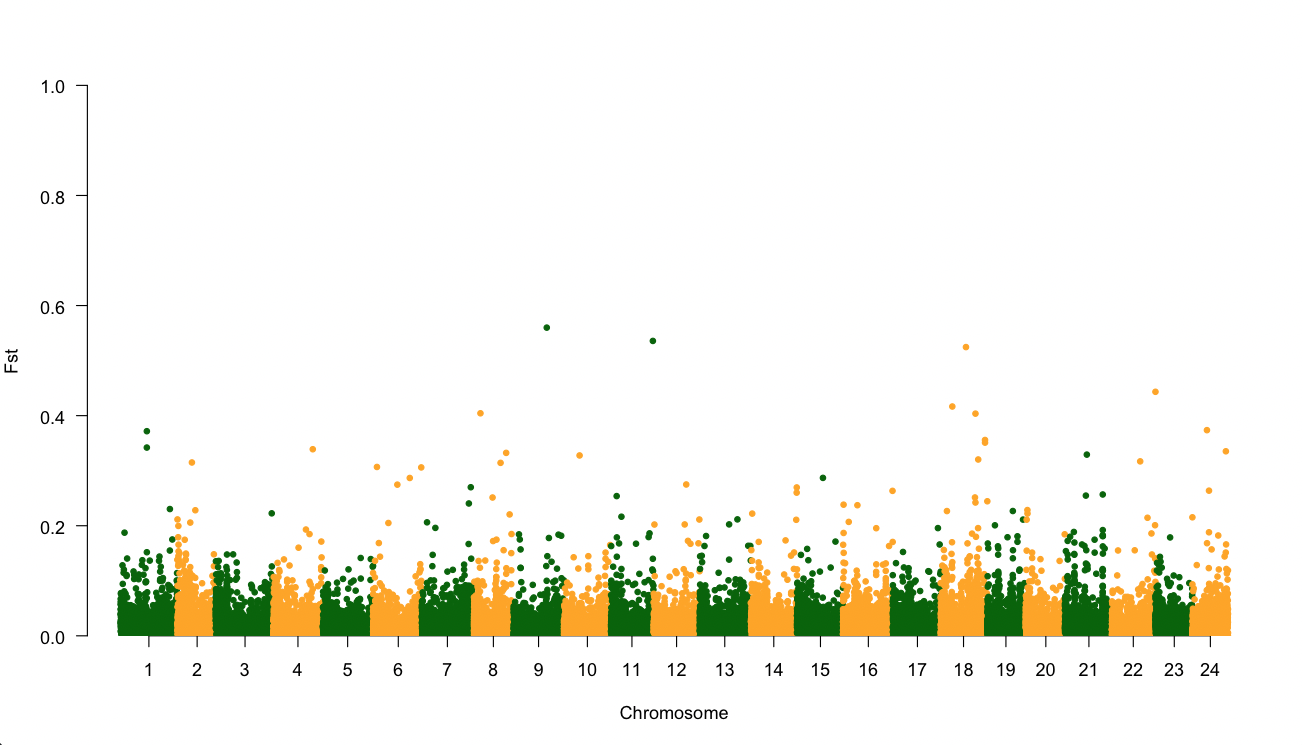
*

**Figure S6**: *Fixation Index (F_ST_* *) comparing allele frequencies of the MIKK panel against wild Kiyosu medaka. Mean F_ST_ in 10-kb windows across the medaka genome for all windows with 50 or more SNPs.*

*
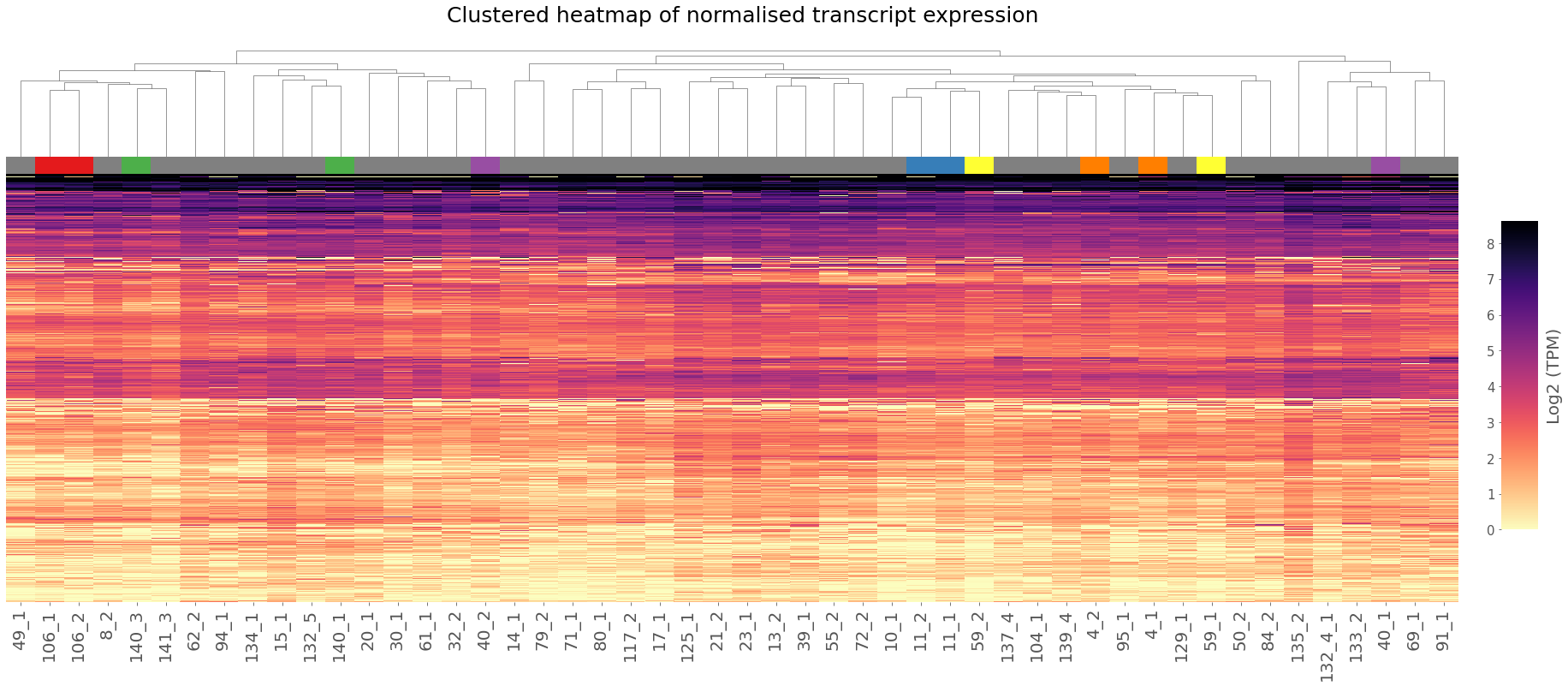
*

**Figure S7:** *Hierarchical clustering of normalised transcript expression across 50 female MIKK panel liver samples, sibling lines are highlighted in different colours.*

*
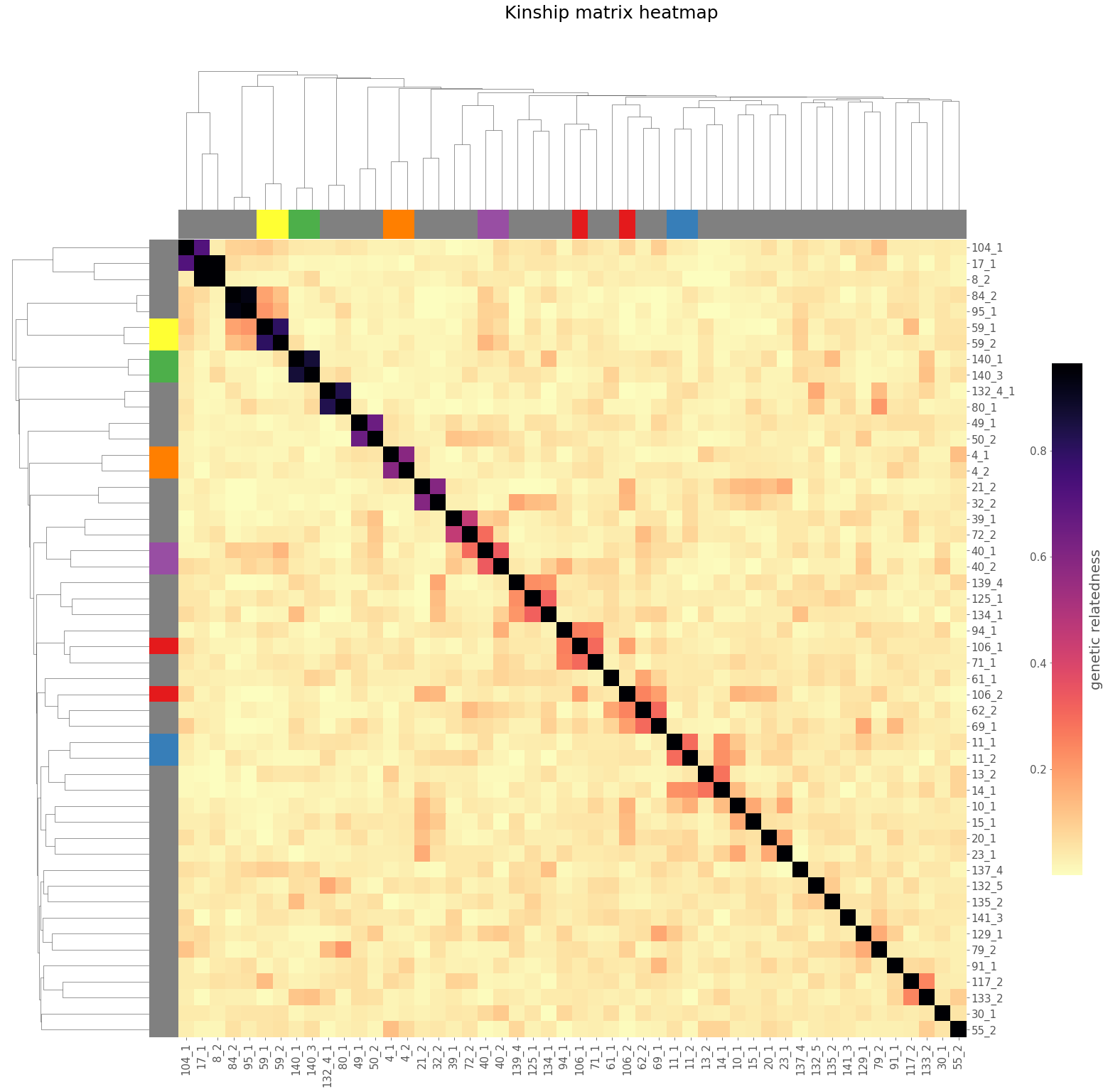
*

**Figure S8:** *Genetic relatedness matrix for the 50 female MIKK panel lines used in the eQTL analysis, derived from SNP genotypes called against the* HdrR *reference. Sibling lines are highlighted in different colours.*
