# Supplementary figures and images for: The Medaka Inbred Kiyosu-Karlsruhe (MIKK) panel
Source: Genome Biol. 2022 Feb 21;23:59. doi: 10.1186/s13059-022-02623-z (PMC8862526; doi:10.1186/s13059-022-02623-z)

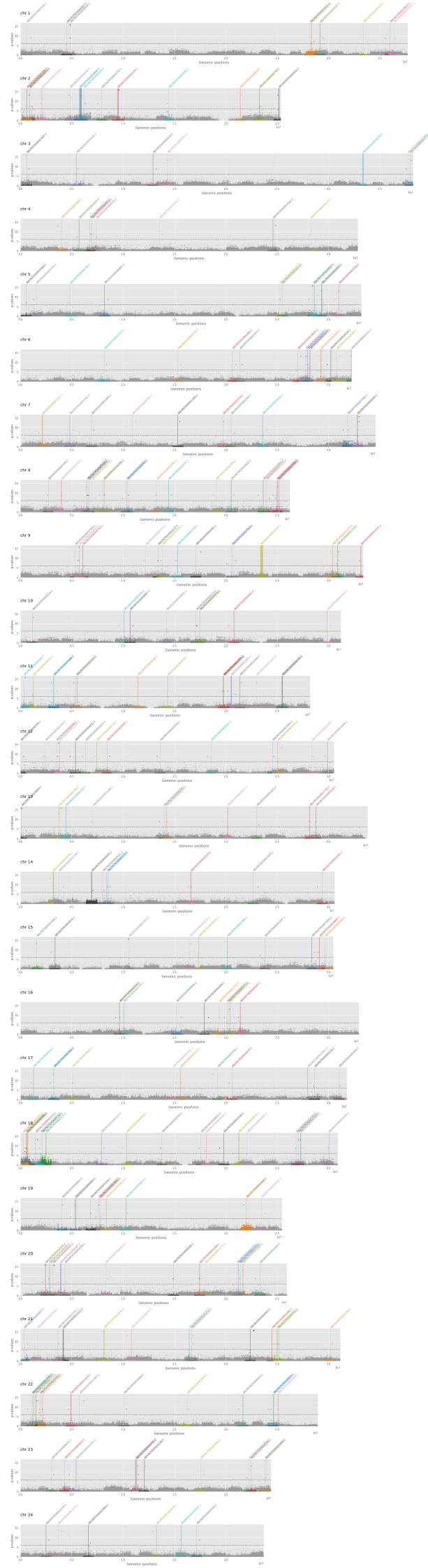

Supplement: Supplementary file 8 — Additional file 8: Figure S9. Copenhagen plot. Plot showing genomic positions of significant eQTL SNPs. [file 13059_2022_2623_MOESM8_ESM.pdf]
