# Supplementary material for: The Medaka Inbred Kiyosu-Karlsruhe (MIKK) panel
Source: Genome Biol. 2022 Feb 21;23:59. doi: 10.1186/s13059-022-02623-z (PMC8862526; doi:10.1186/s13059-022-02623-z)

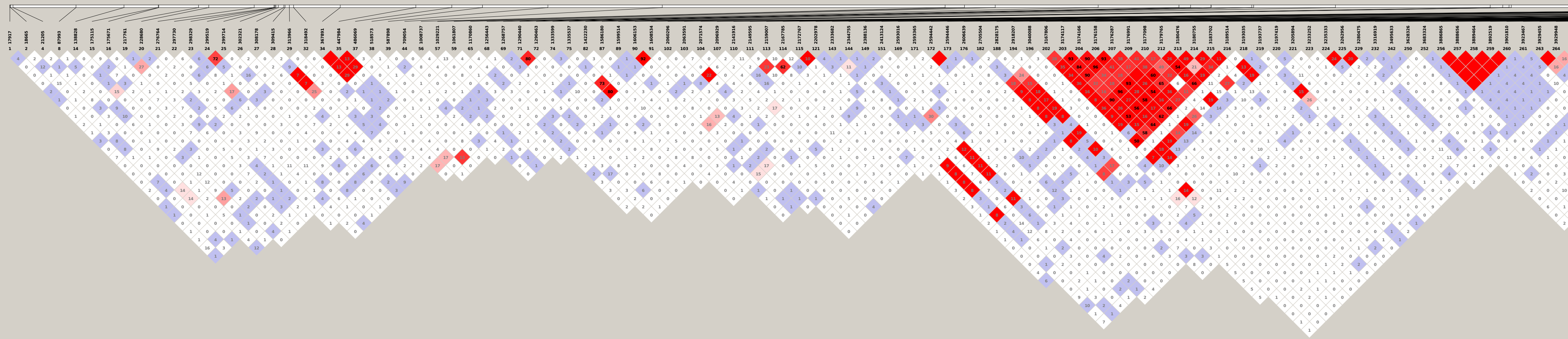

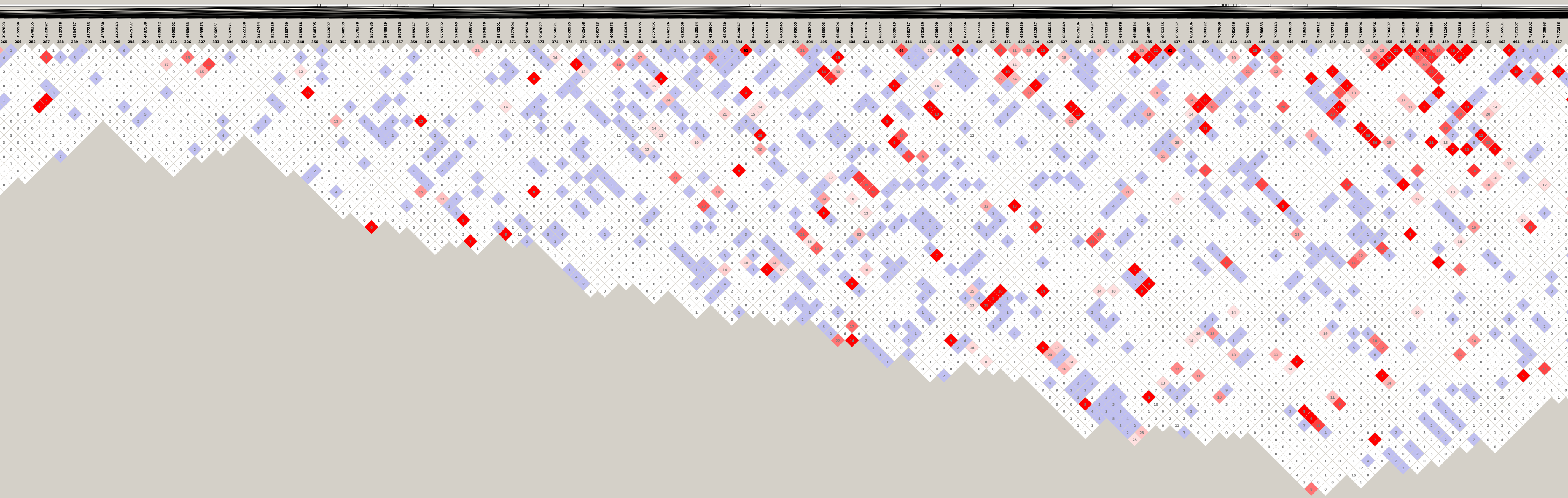

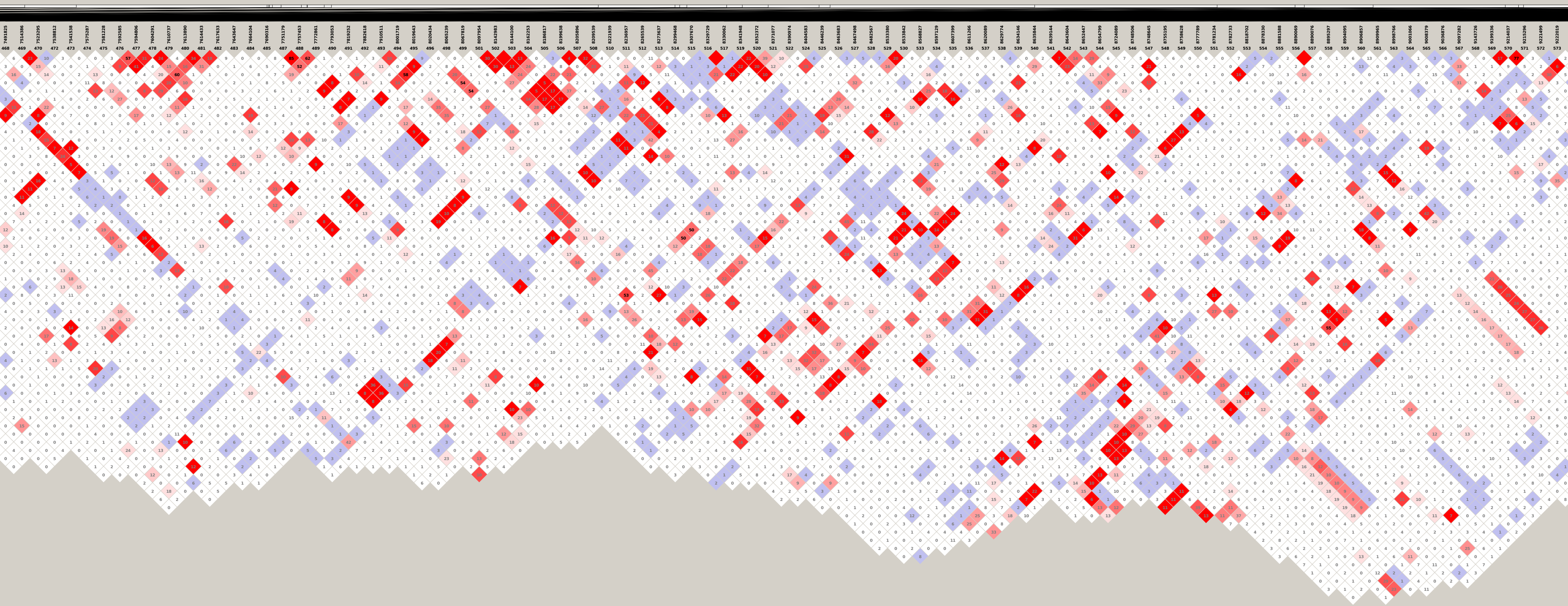

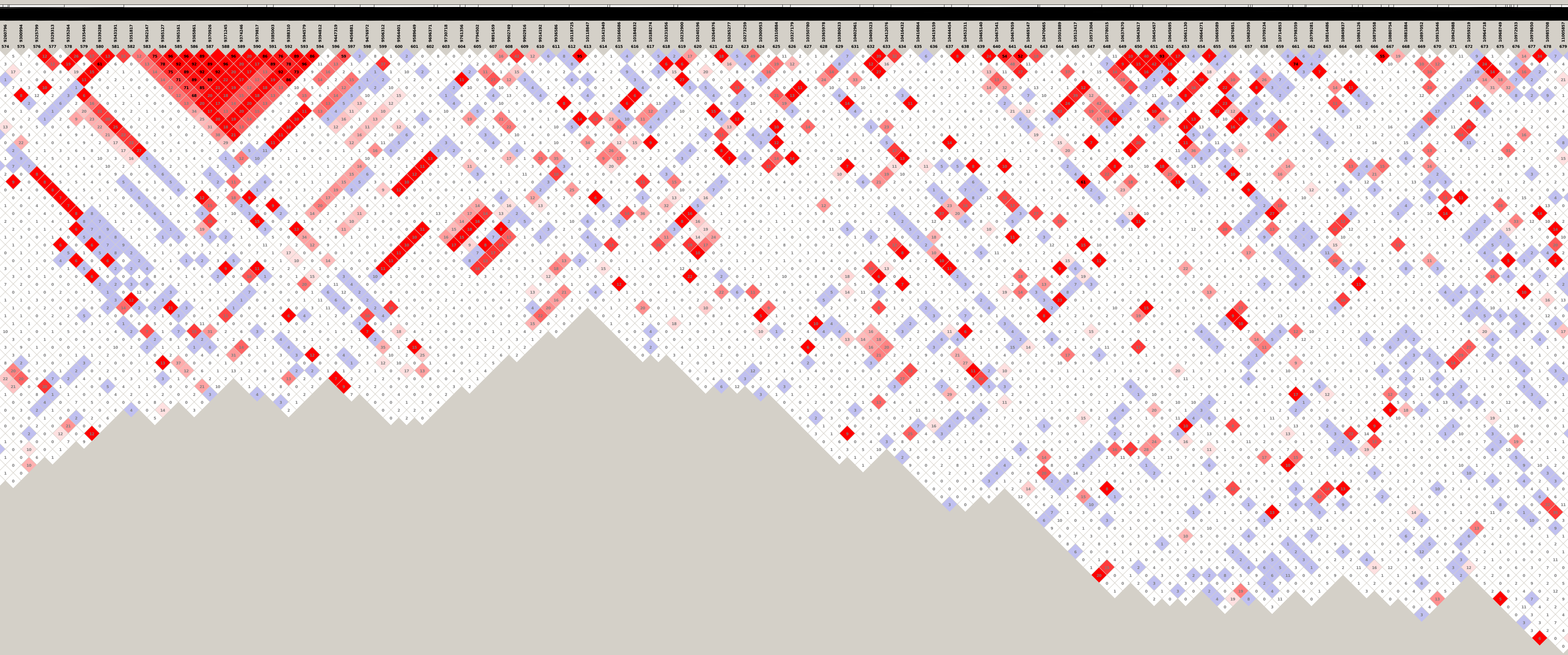

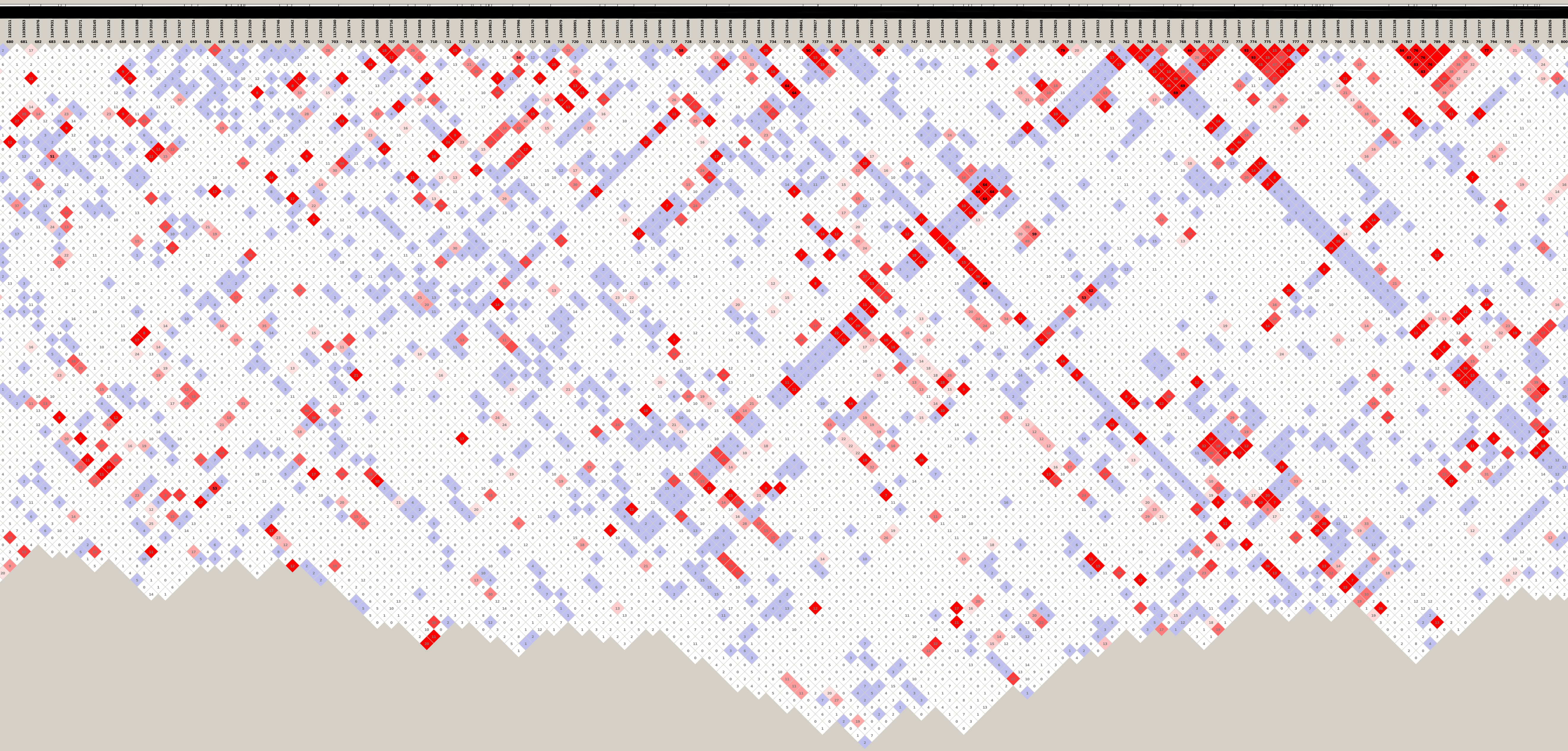

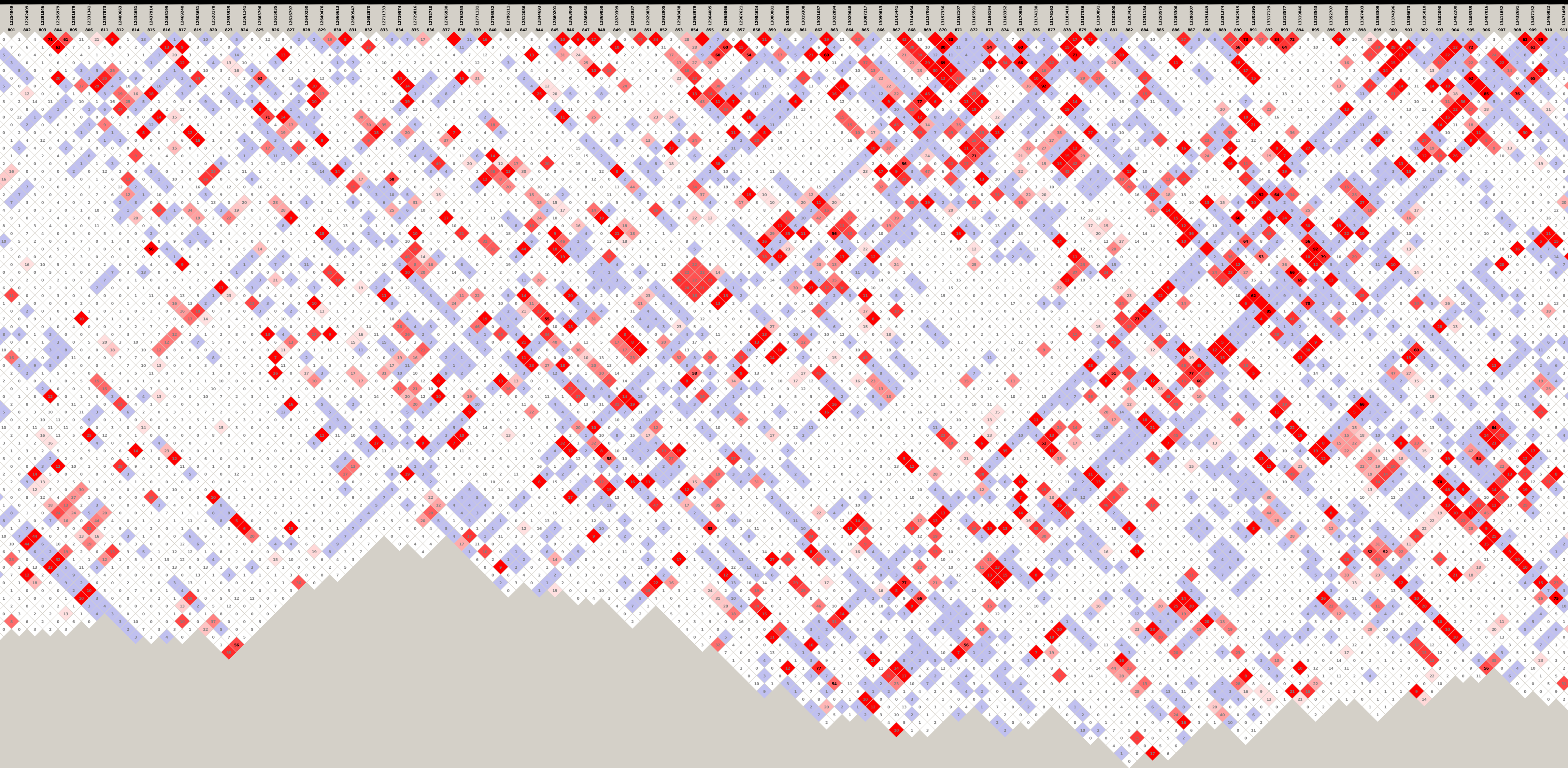

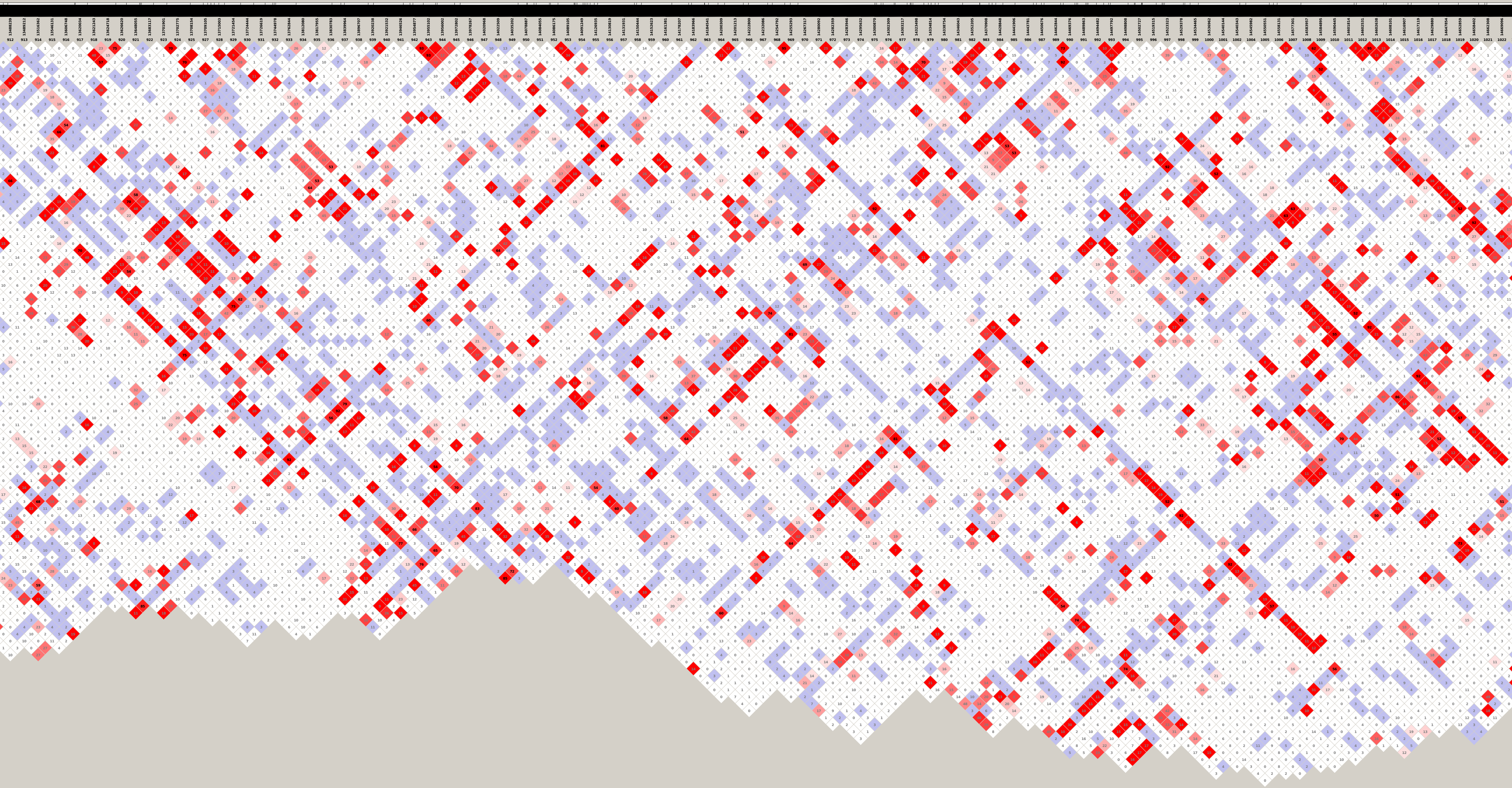

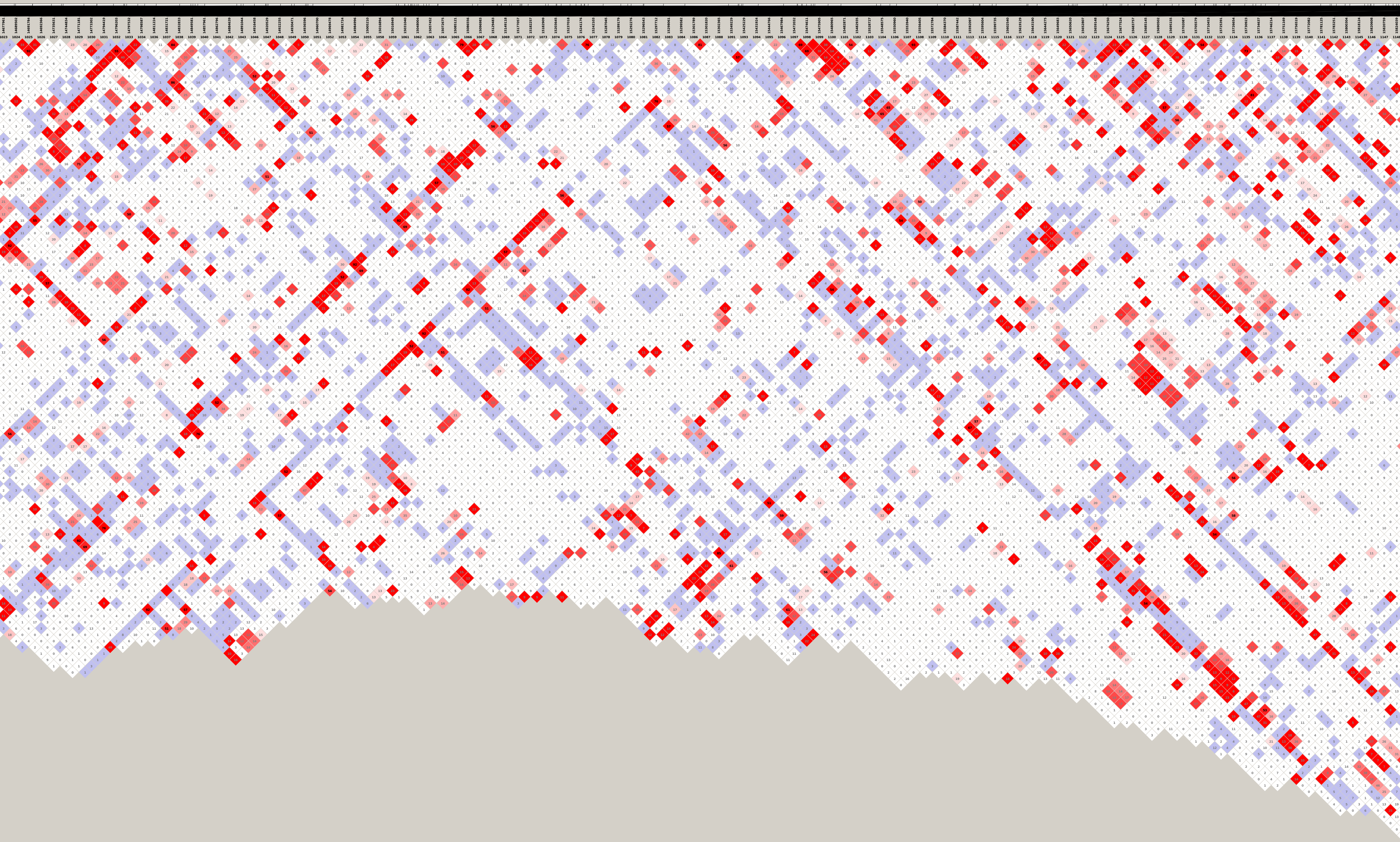

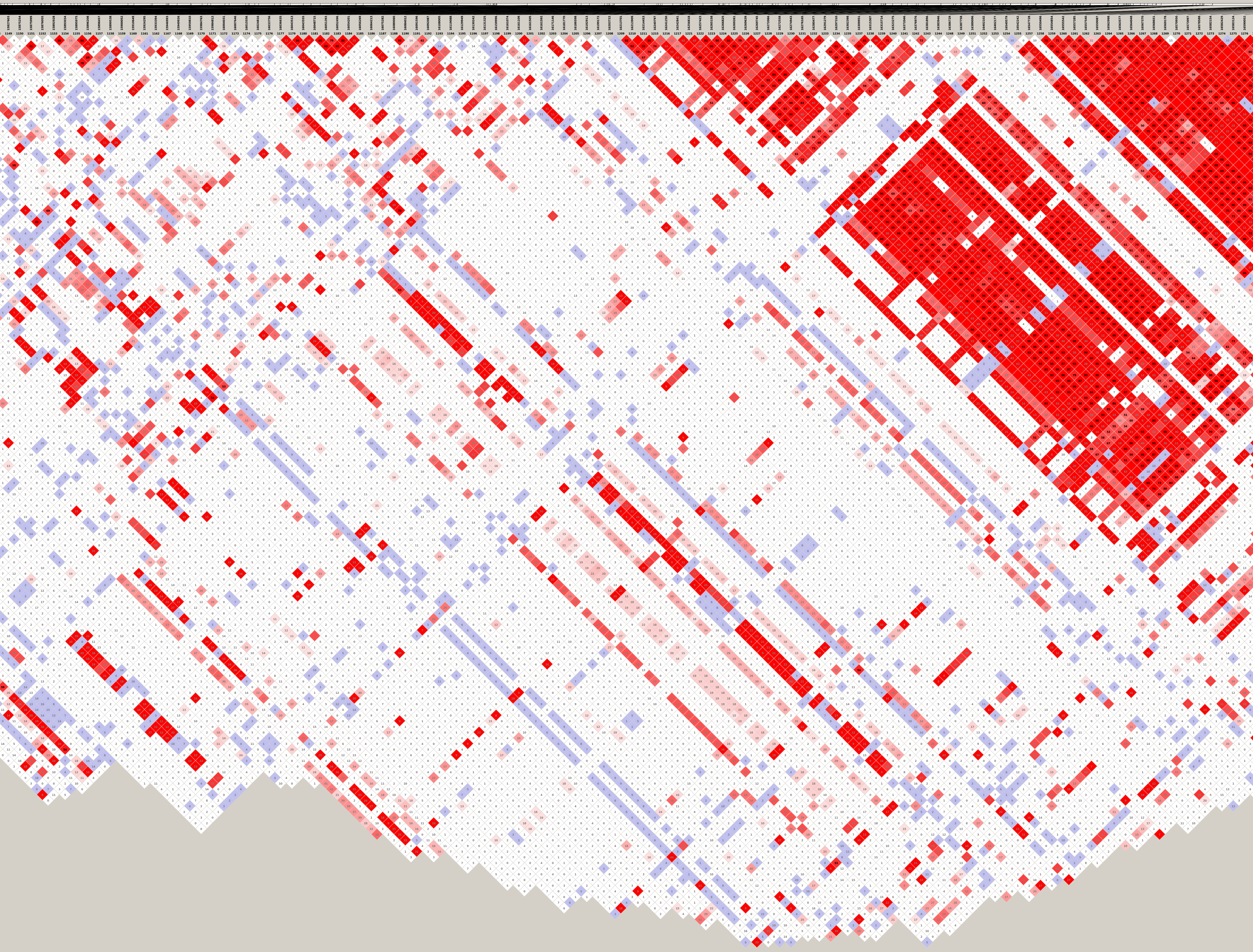

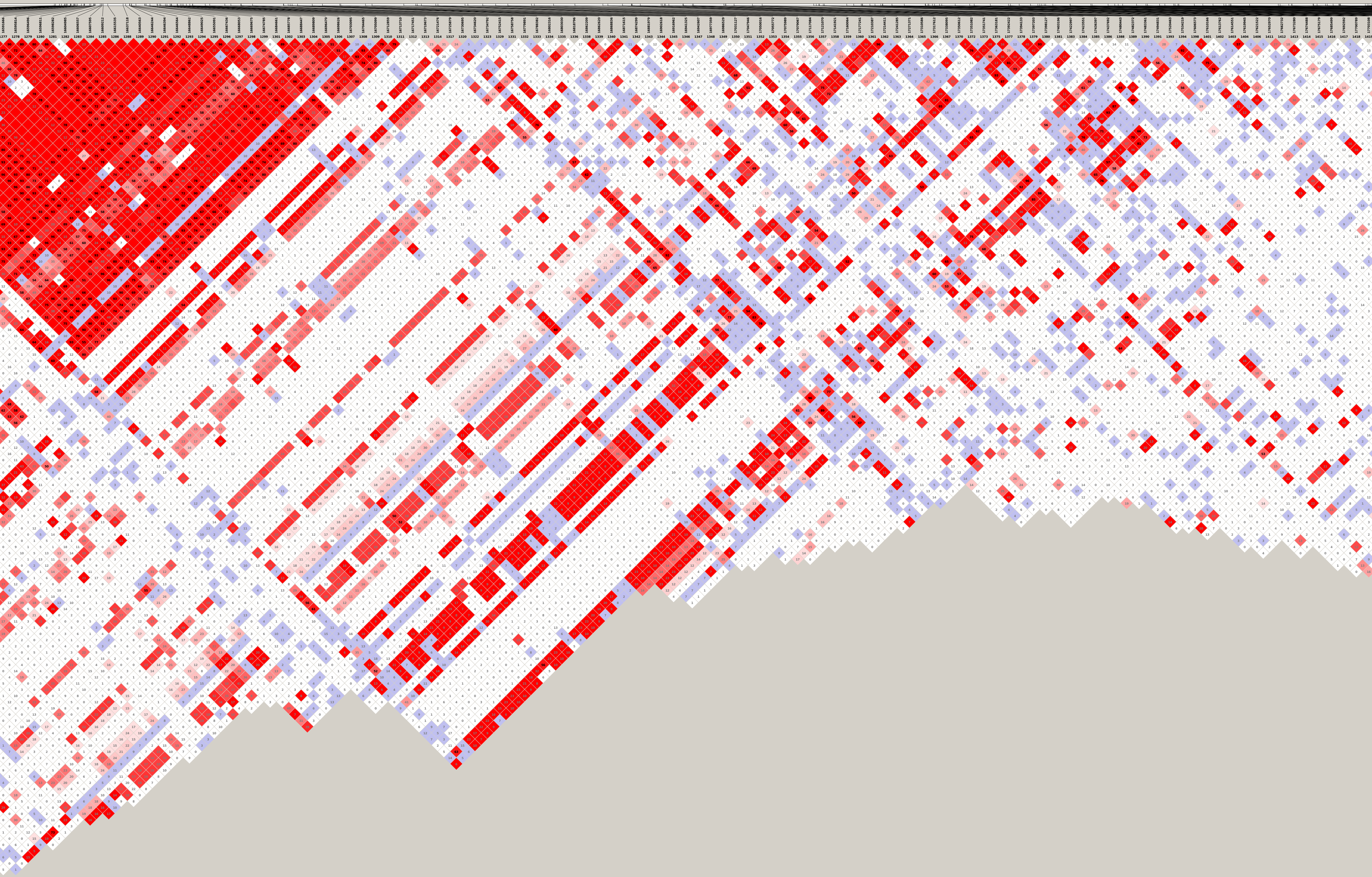

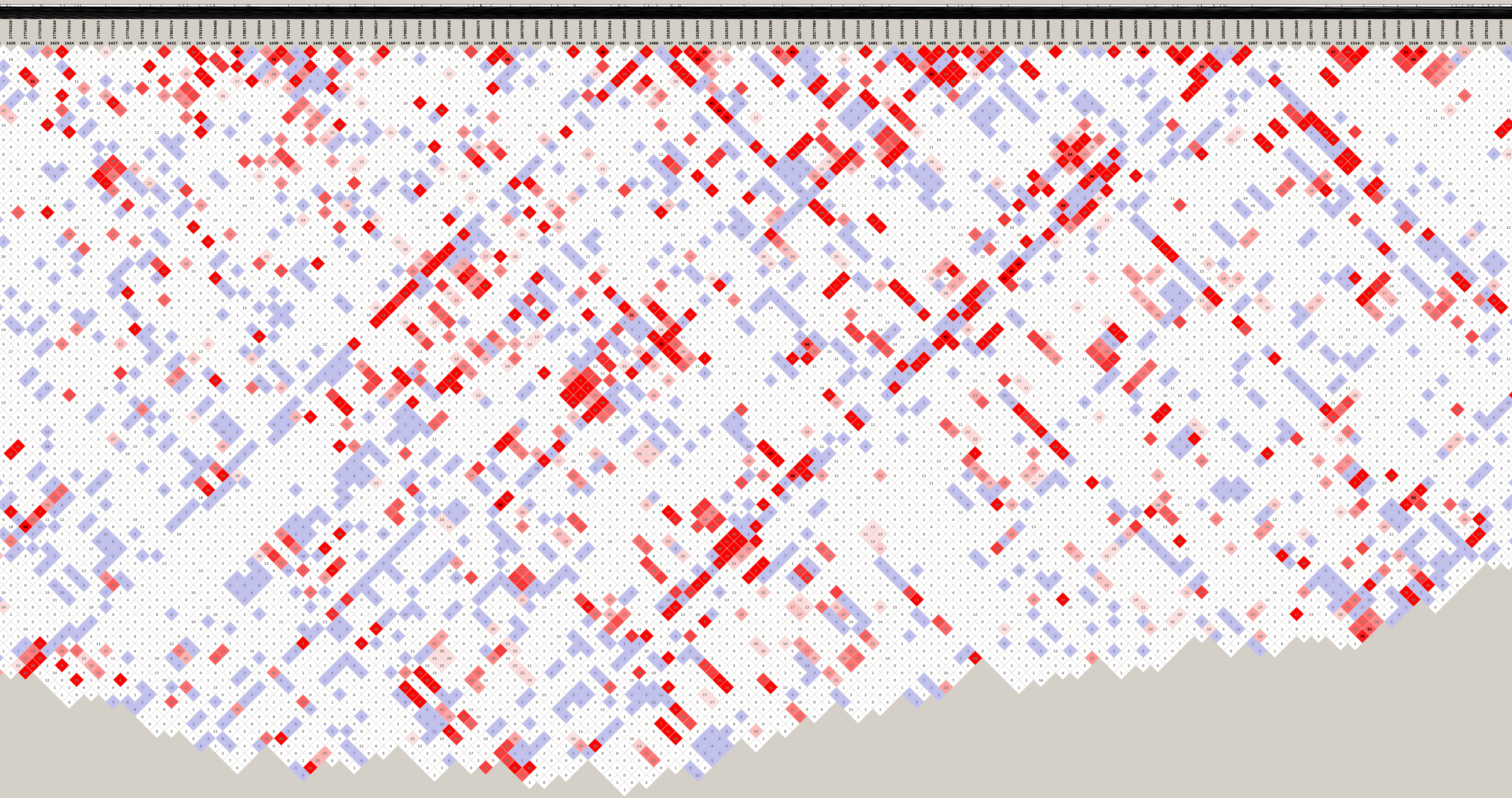

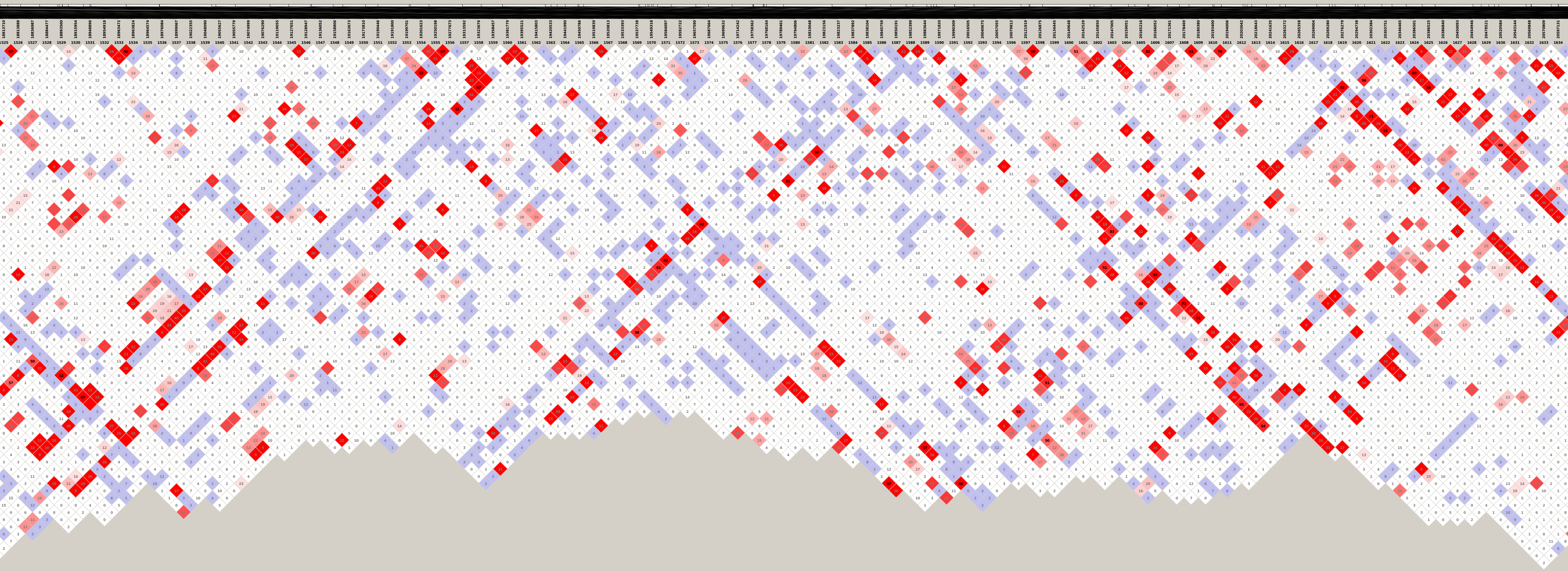

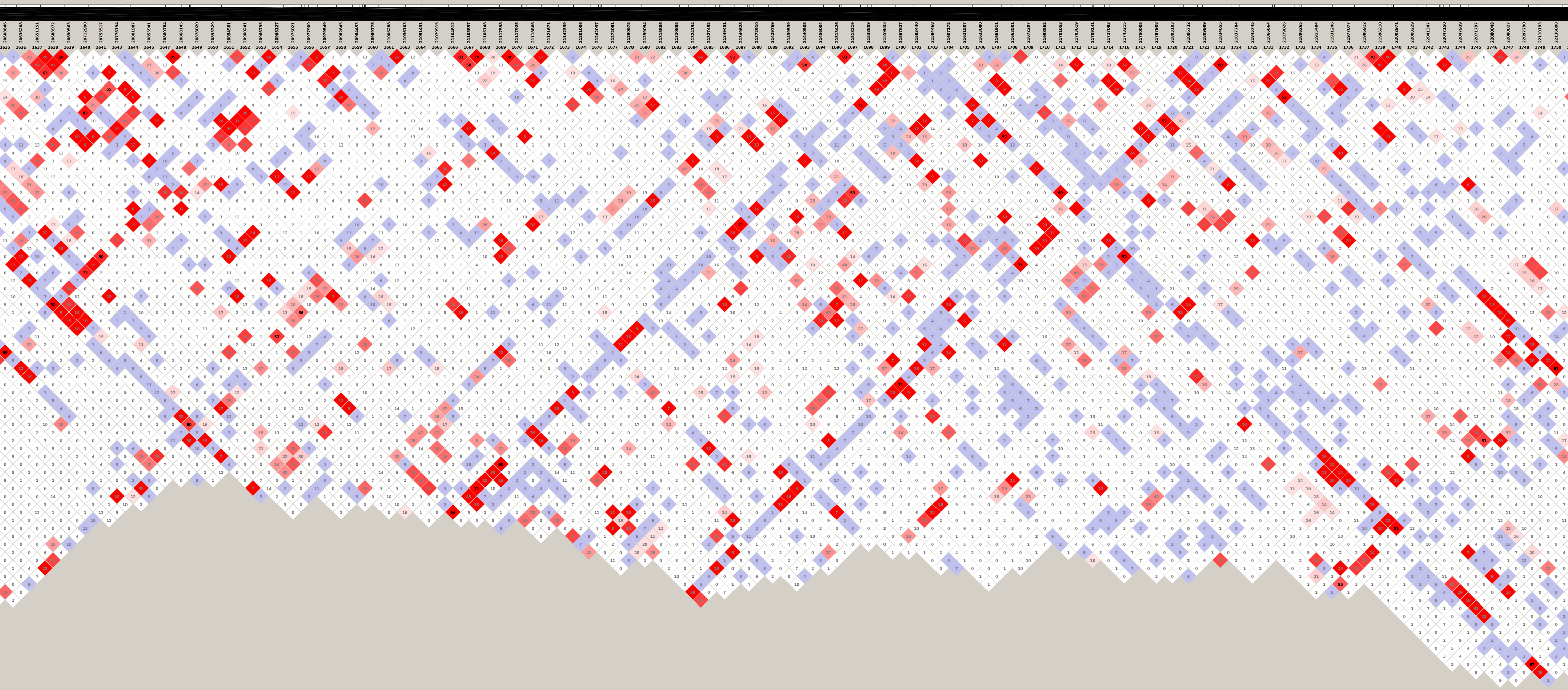

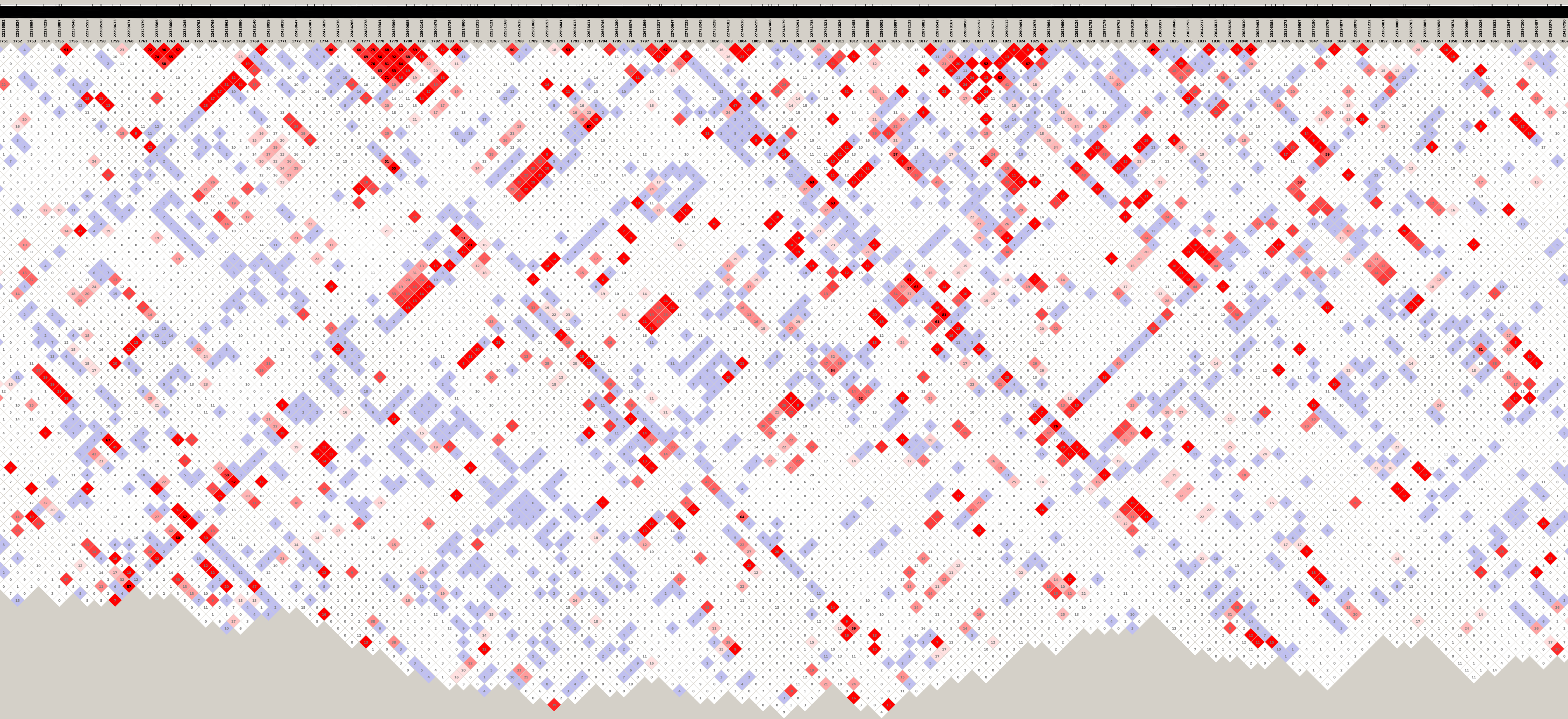

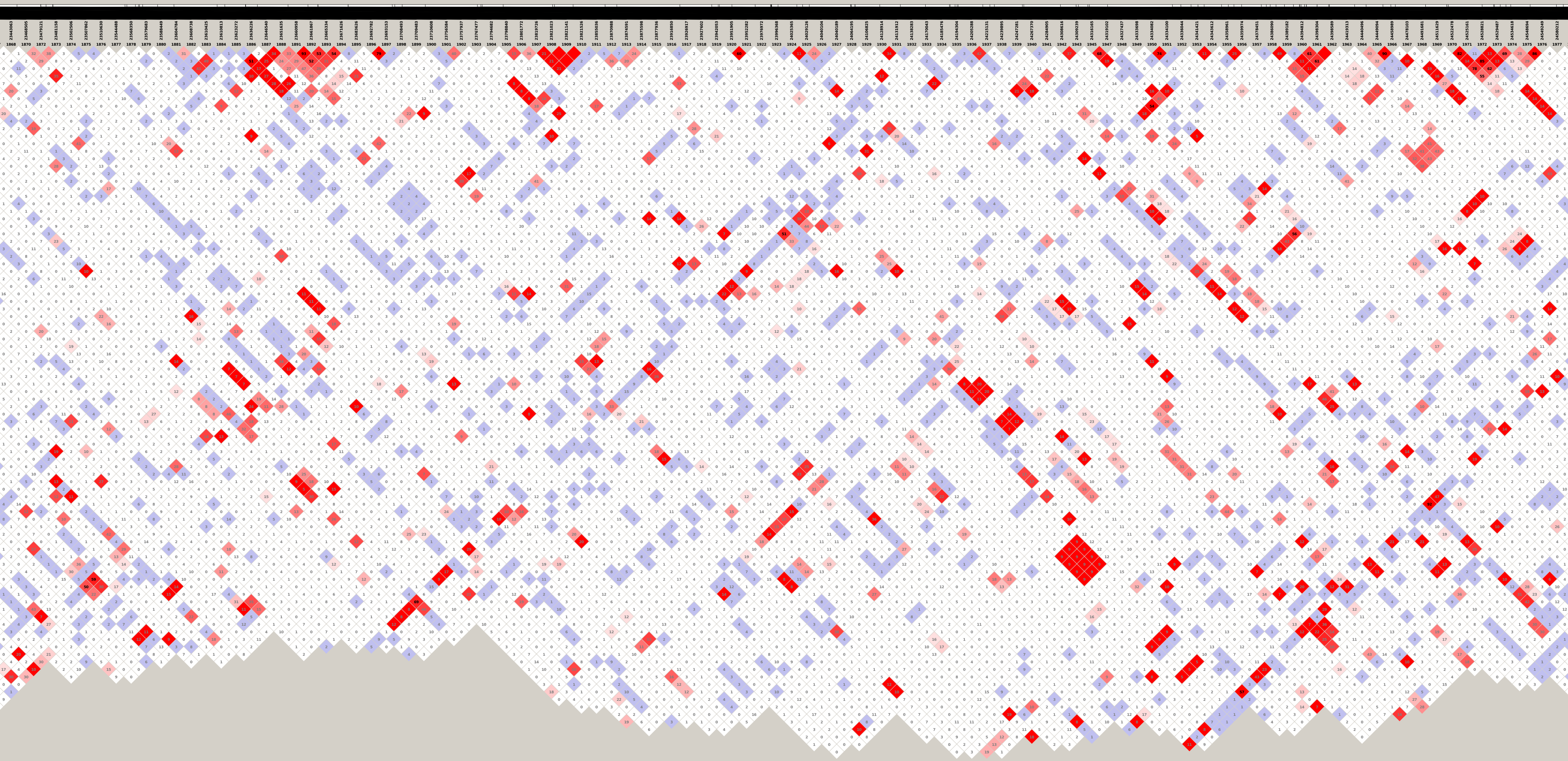

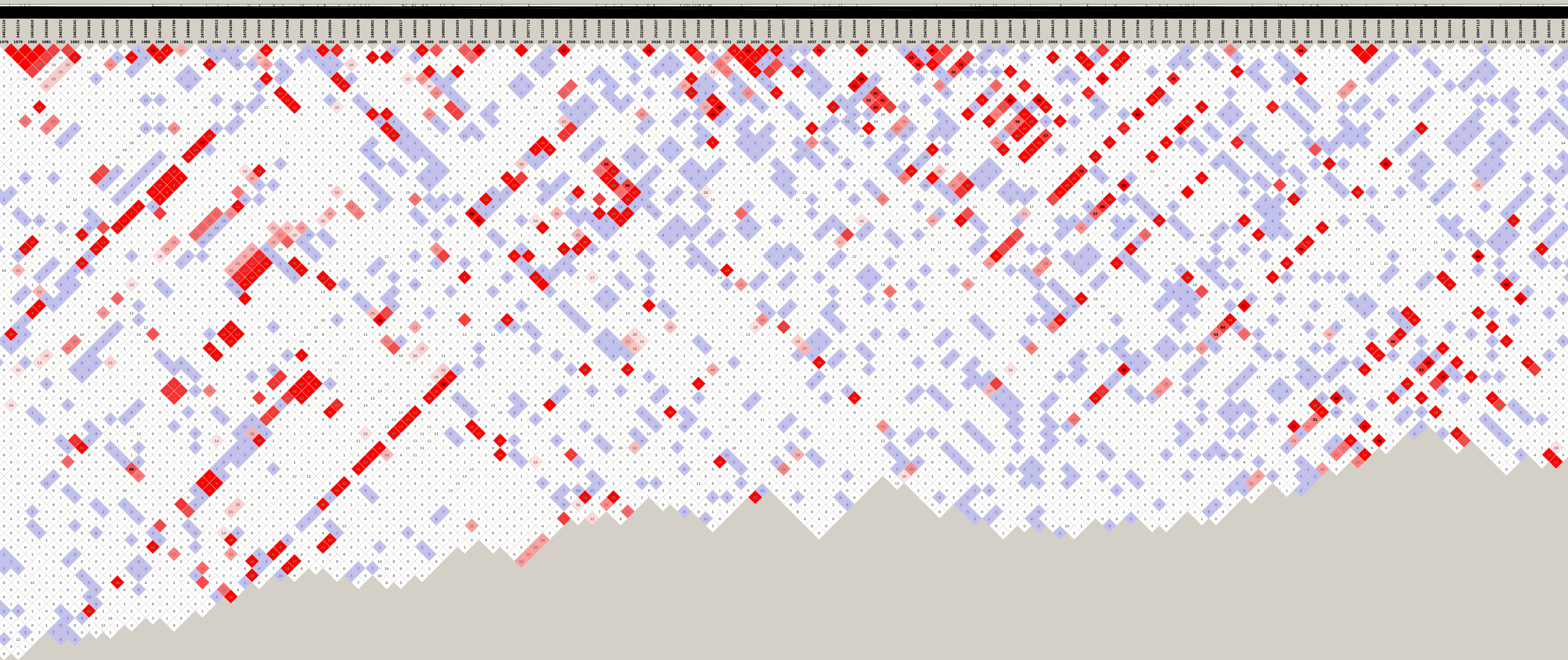

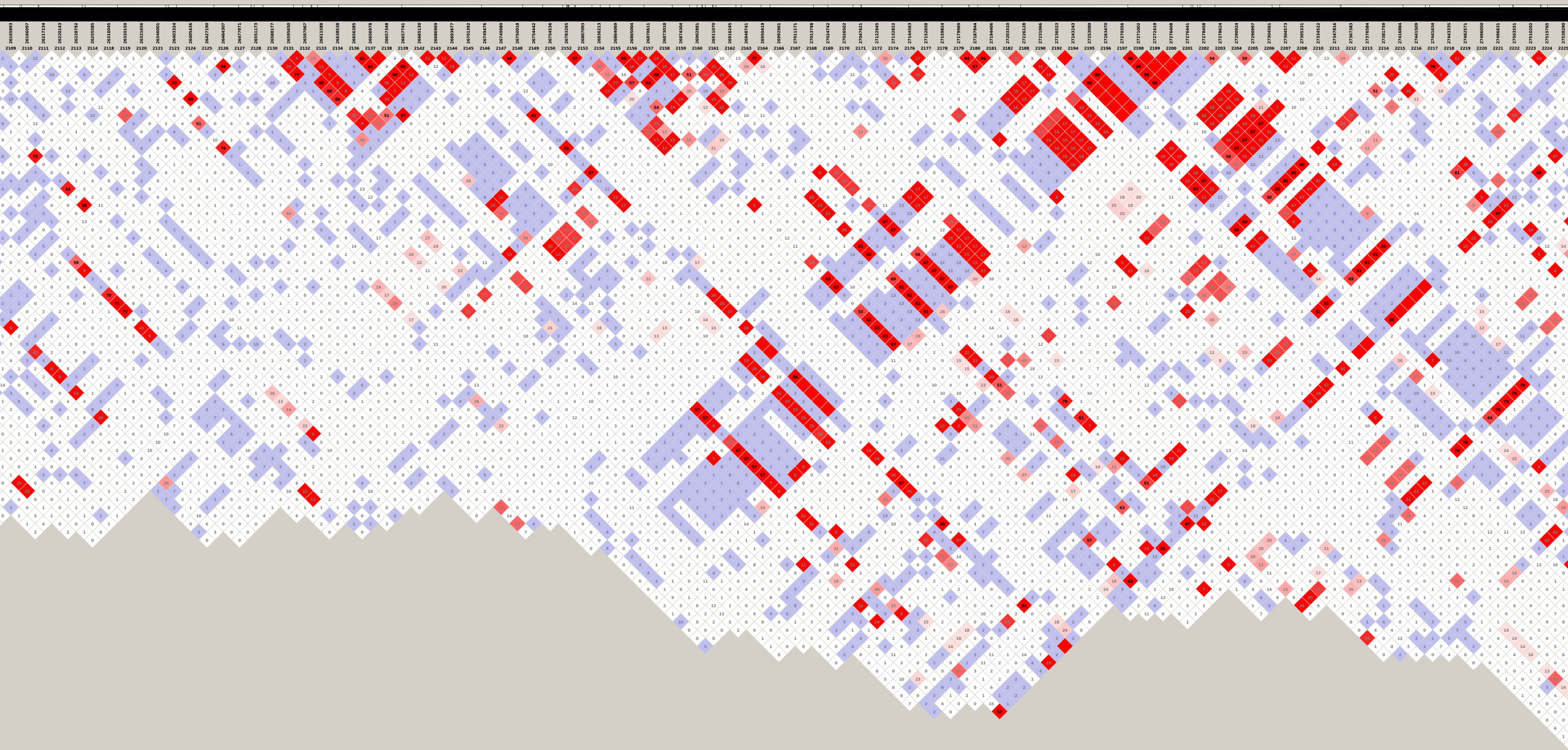

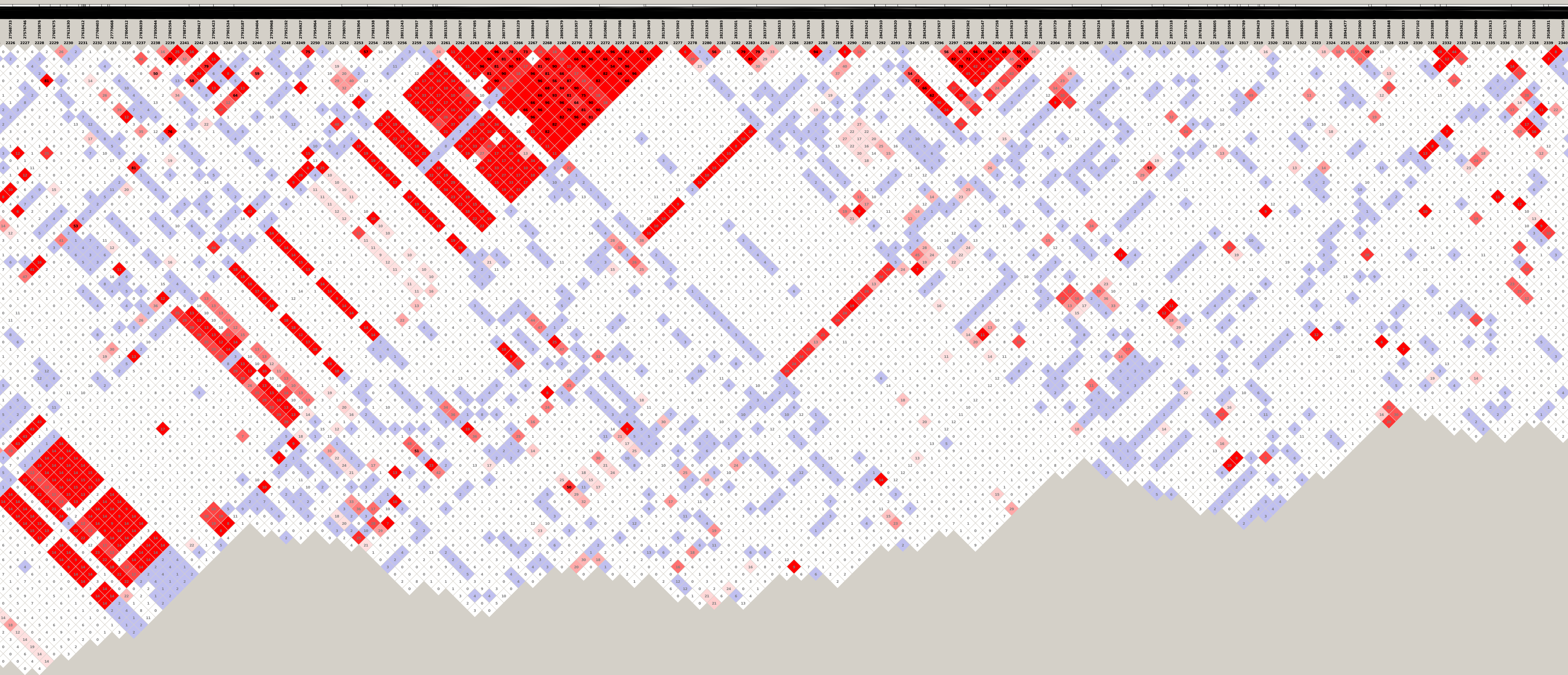

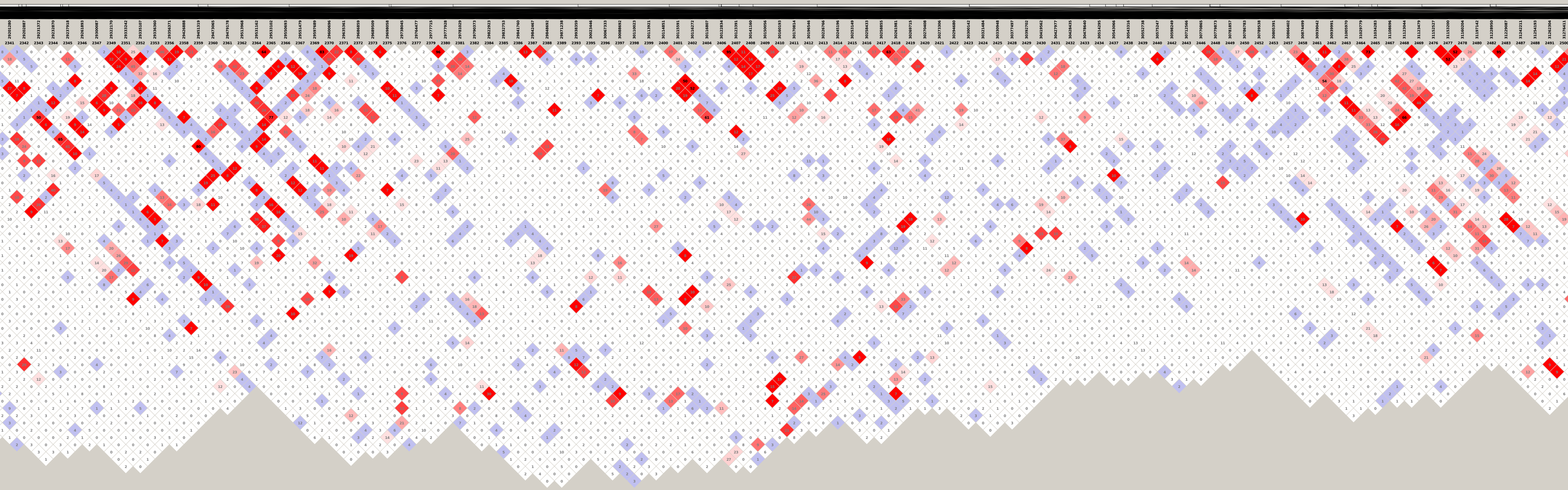

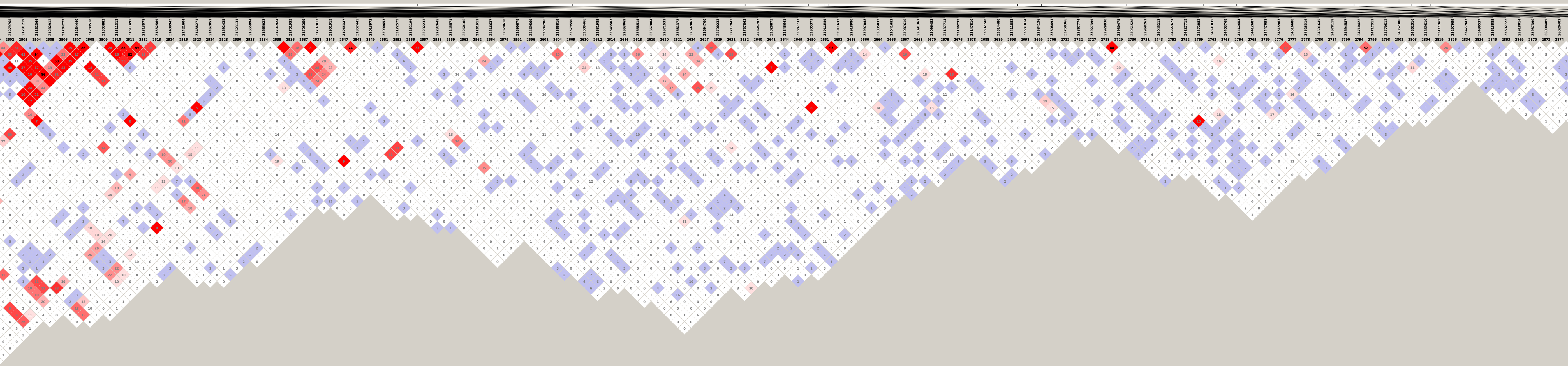

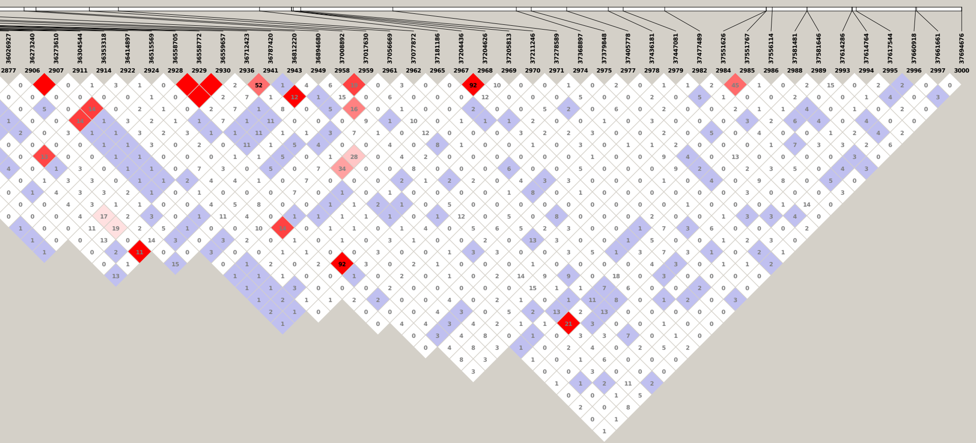
36026927 36273240 36273610 36304544 36353318 36414897 36515569 36558705 36558772 36559657 36712423 36787420 36812220 36894680 37008892 37017630 37056669 37077872 37181186 37204436 37204626 37205813 37211246 37278589 37368897 37379848 37405778 37436181 37447081 37477489 37551626 37551767 37556114 37581481 37581646 37614286 37614764 37617544 37660918 37661661 37694676
2877 2906 2907 2911 2914 2922 2924 2928 2929 2930 2936 2941 2943 2949 2958 2959 2961 2962 2965 2967 2968 2969 2970 2971 2974 2975 2977 2978 2979 2982 2984 2985 2986 2988 2989 2993 2994 2995 2996 2997 3000

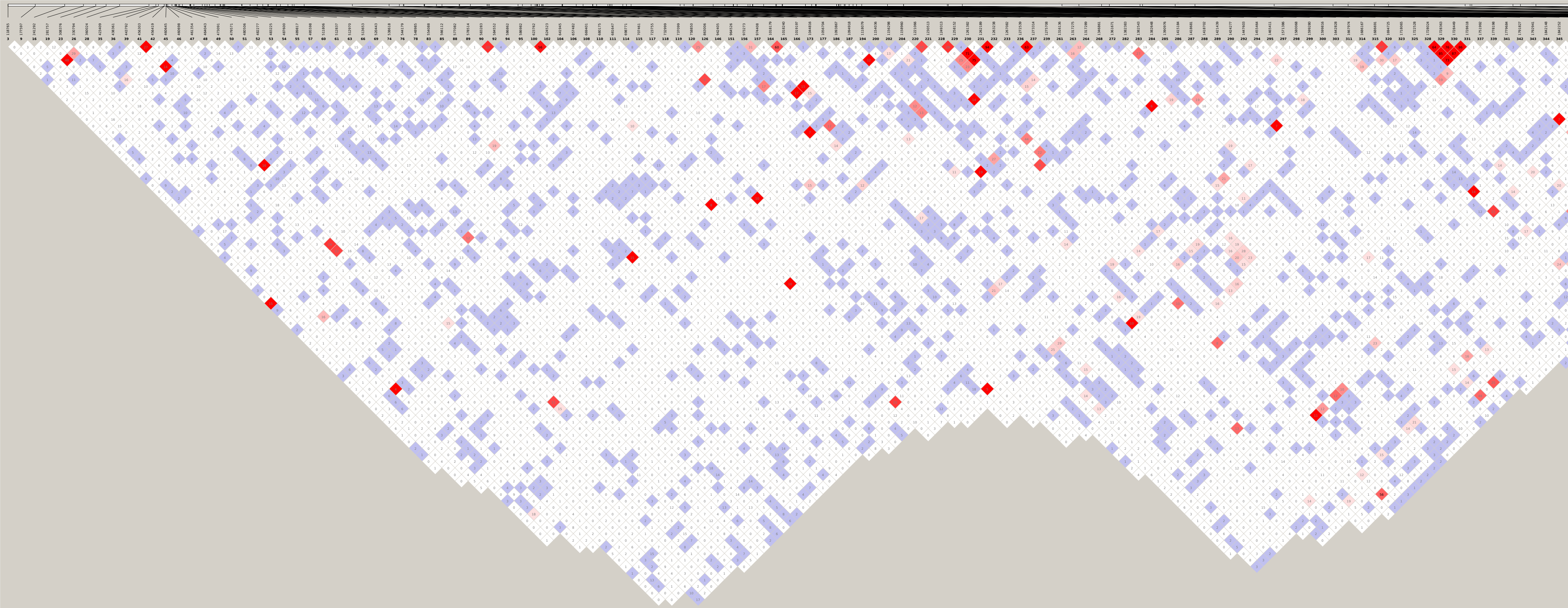

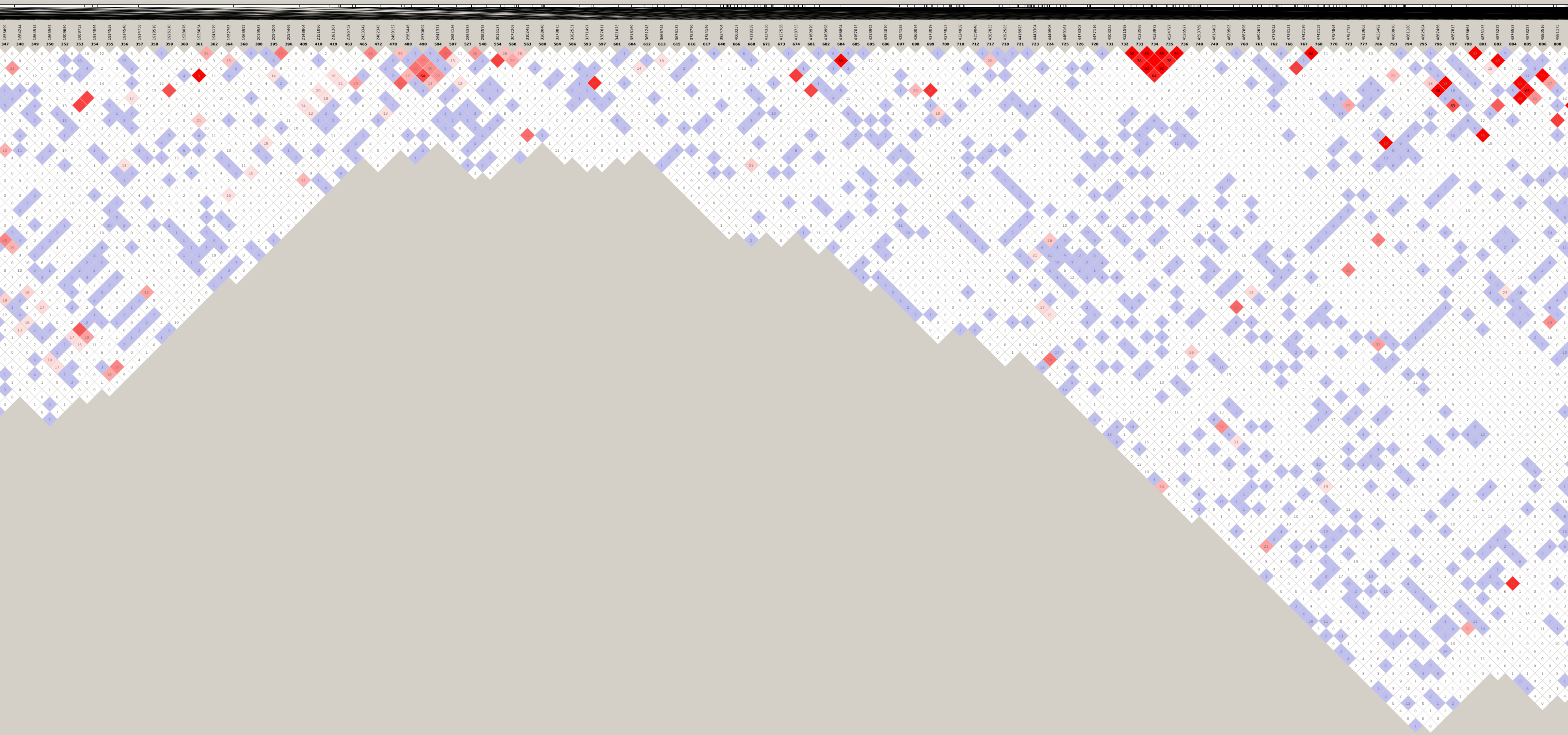

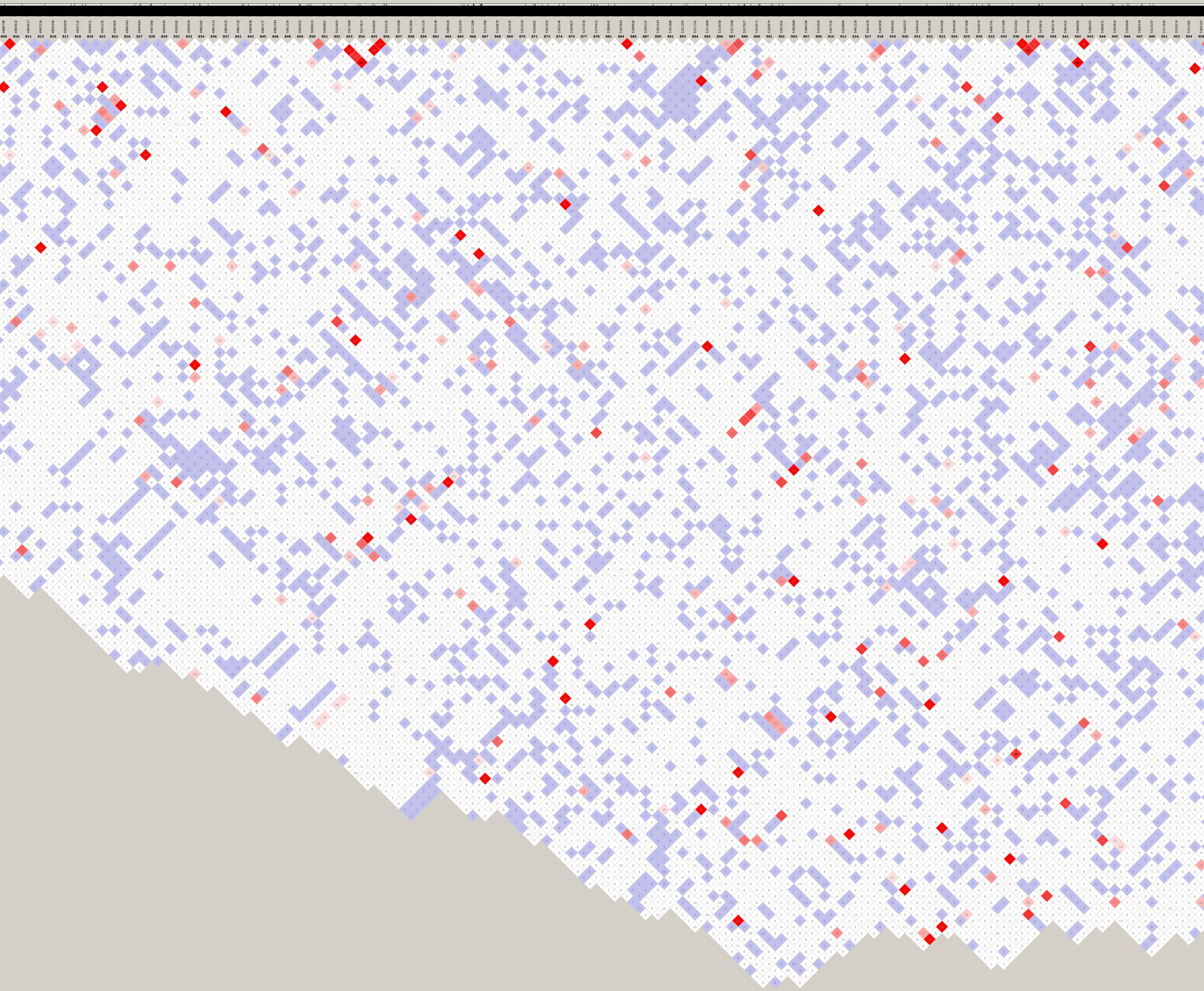

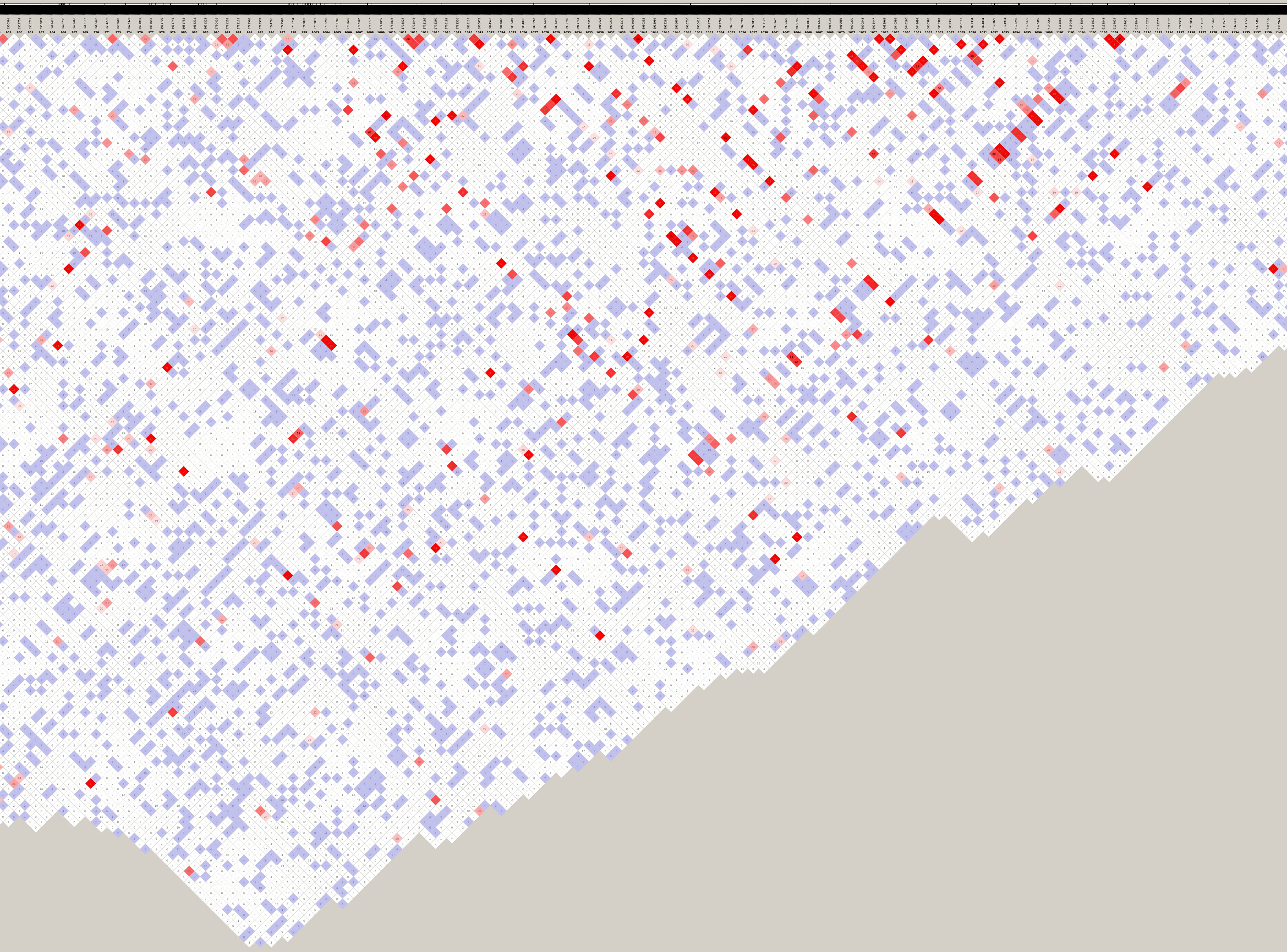

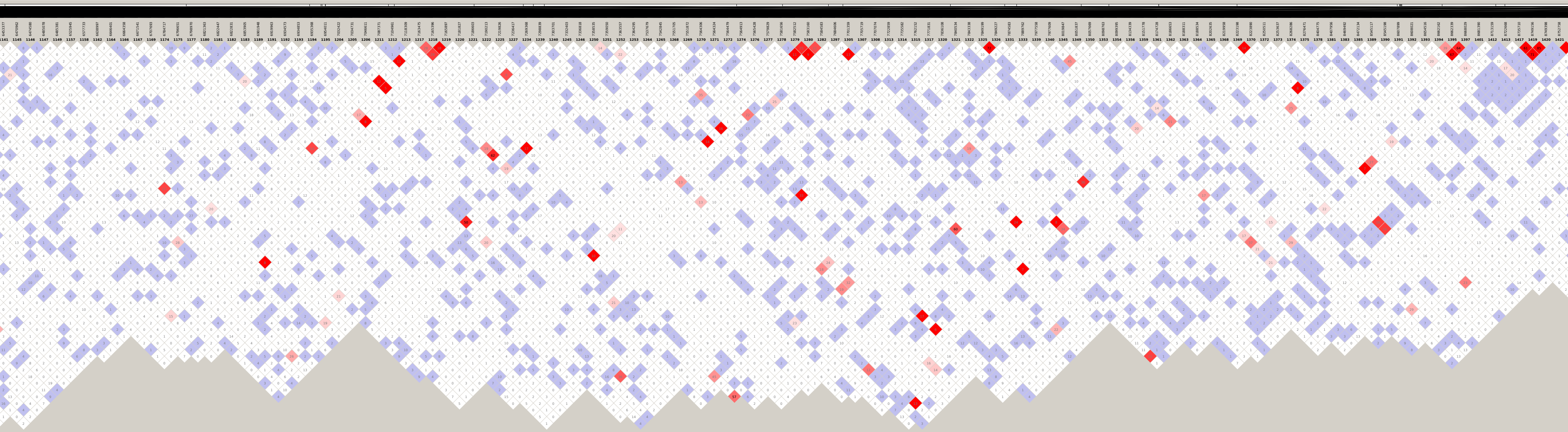

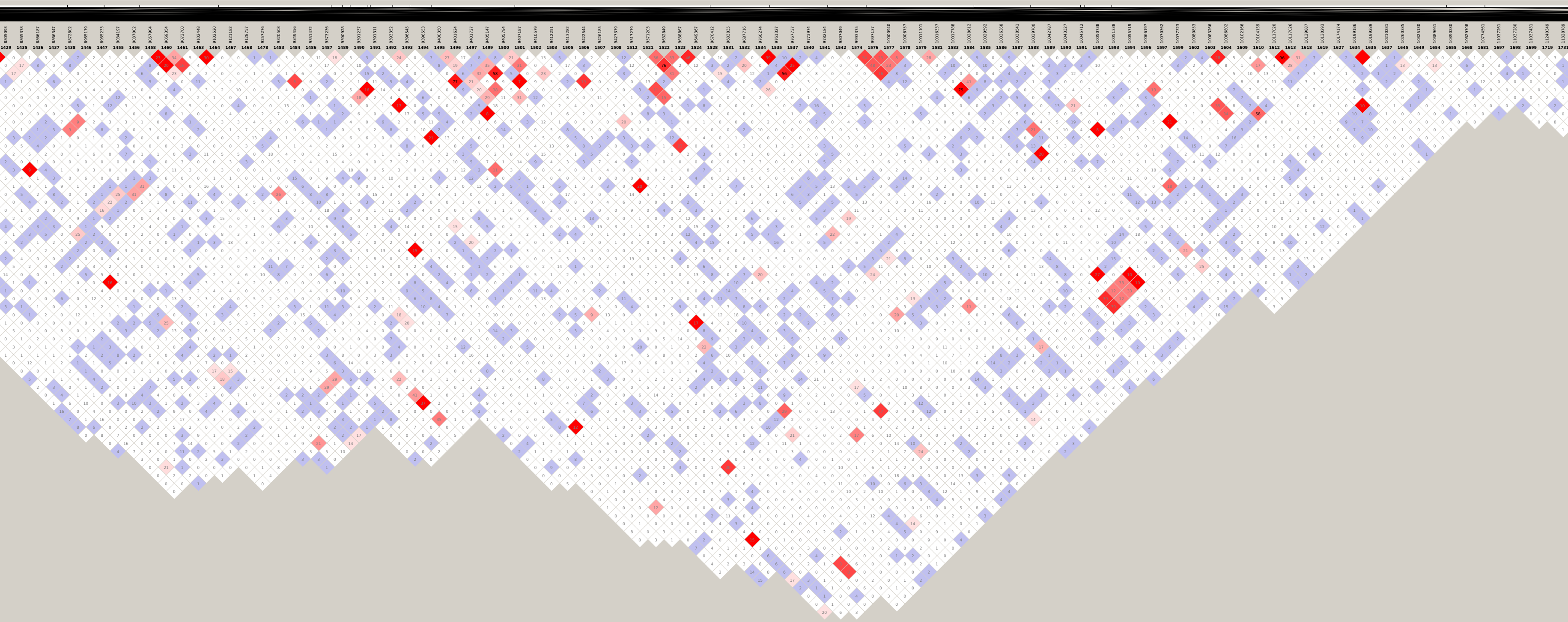

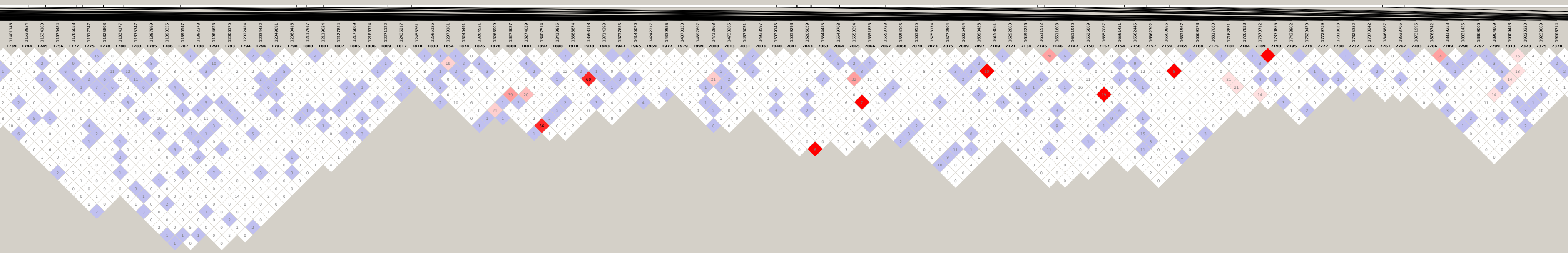

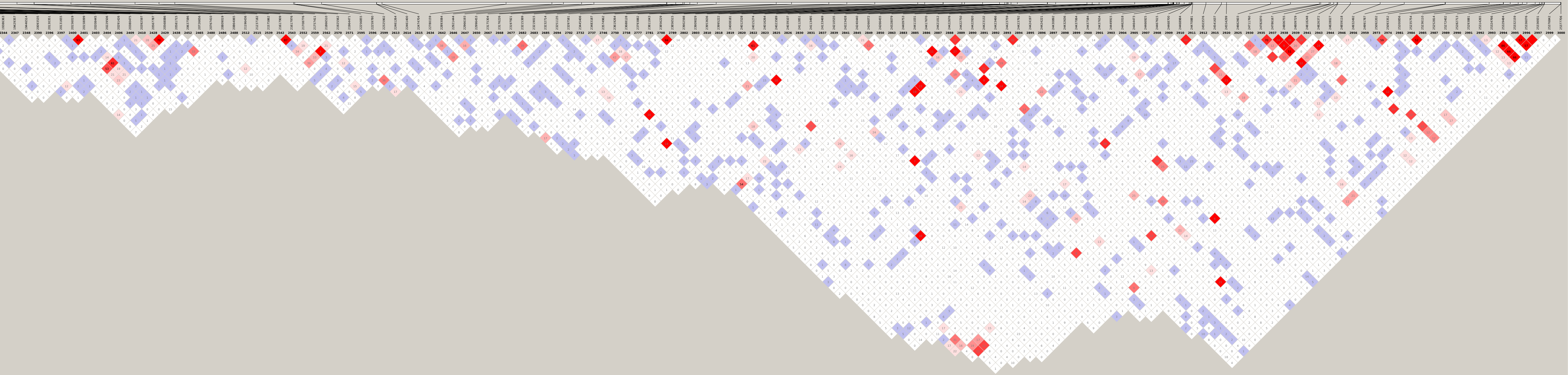

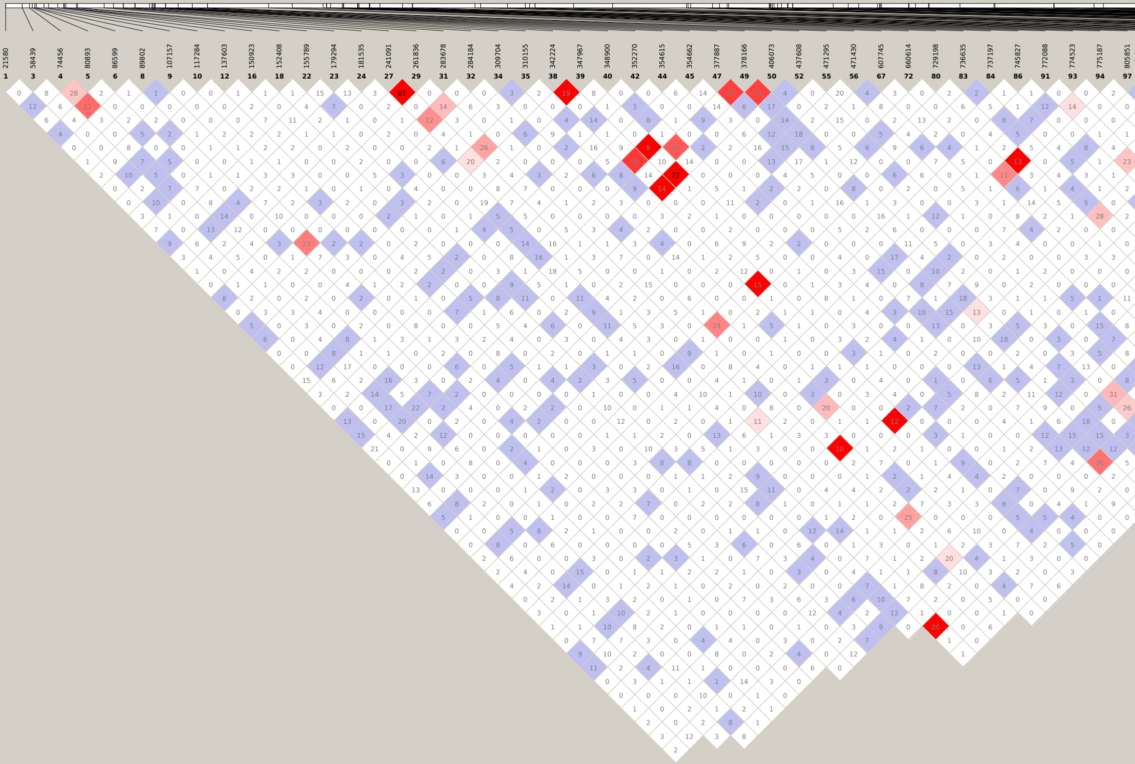
21580 58439 74456 80893 86599 89802 107157 117284 137603 150923 152408 155789 179294 181535 241091 261836 283678 284184 309704 310155 342224 347967 348900 352270 354615 354662 377887 378166 406073 437608 471295 471430 607745 660614 729198 736635 737197 745827 772088 774523 775187 805851
1 3 4 5 6 8 9 10 12 16 18 22 23 24 27 29 31 32 34 35 38 39 40 42 44 45 47 49 50 52 55 56 67 72 80 83 84 86 91 93 94 97

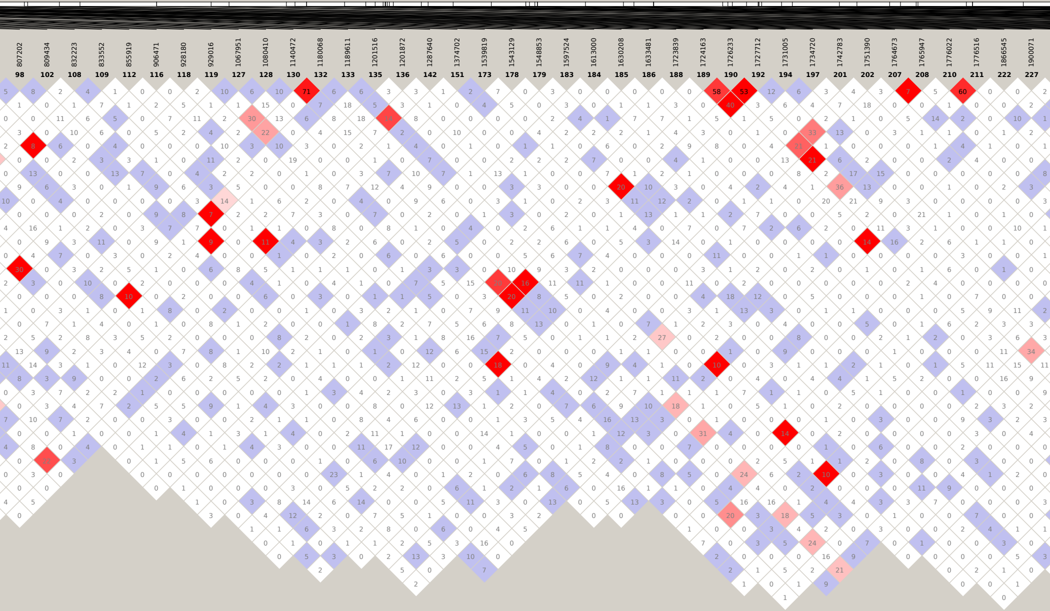
807202 98
809434 102
832223 108
833552 109
855919 112
906471 116
928180 118
929016 119
1067951 127
1080410 128
1140472 130
1180068 132
1189611 133
1201516 135
1201872 136
1287640 142
1374702 151
1539819 173
1543129 178
1548853 179
1597524 183
1613000 184
1630208 185
1633481 186
1723839 188
1724163 189
1726233 190
1727712 192
1731005 194
1734720 197
1742783 201
1751390 202
1764673 207
1765947 208
1776022 210
1776516 211
1866545 222
1900071 227

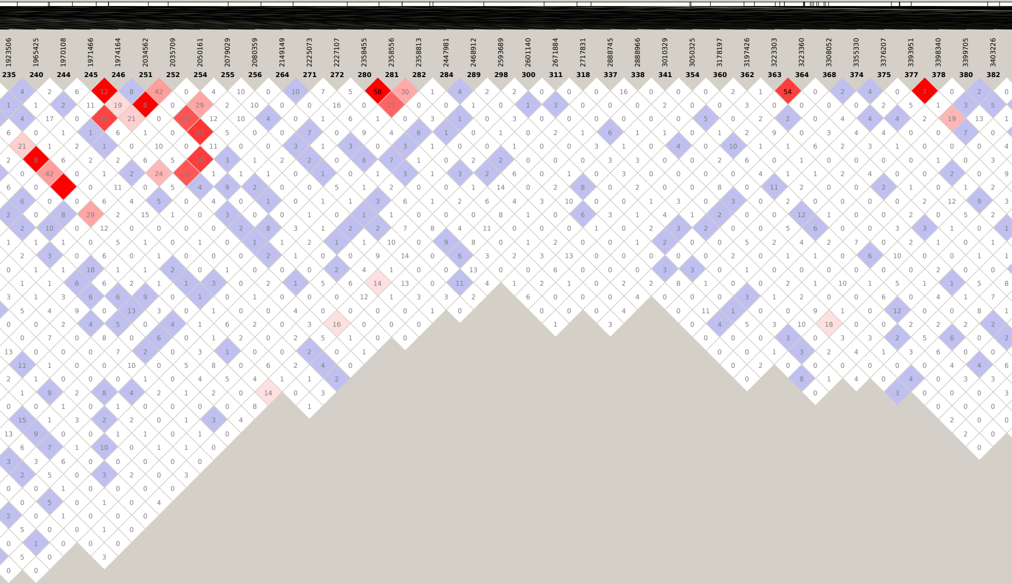
1923506 235
1965425 240
1970108 244
1971466 245
1974164 246
2034562 251
2035709 252
2050161 254
2079029 255
2080359 256
2149149 264
2225073 271
2227107 272
2358455 280
2358556 281
2358813 282
2447981 284
2468912 289
2593689 298
2601140 300
2671884 311
2717831 318
2888745 337
2888966 338
3010329 341
3050325 354
3178197 360
3197426 362
3223303 363
3223360 364
3308052 368
3355330 374
3376207 375
3393951 377
3398340 378
3399705 380
3403226 382

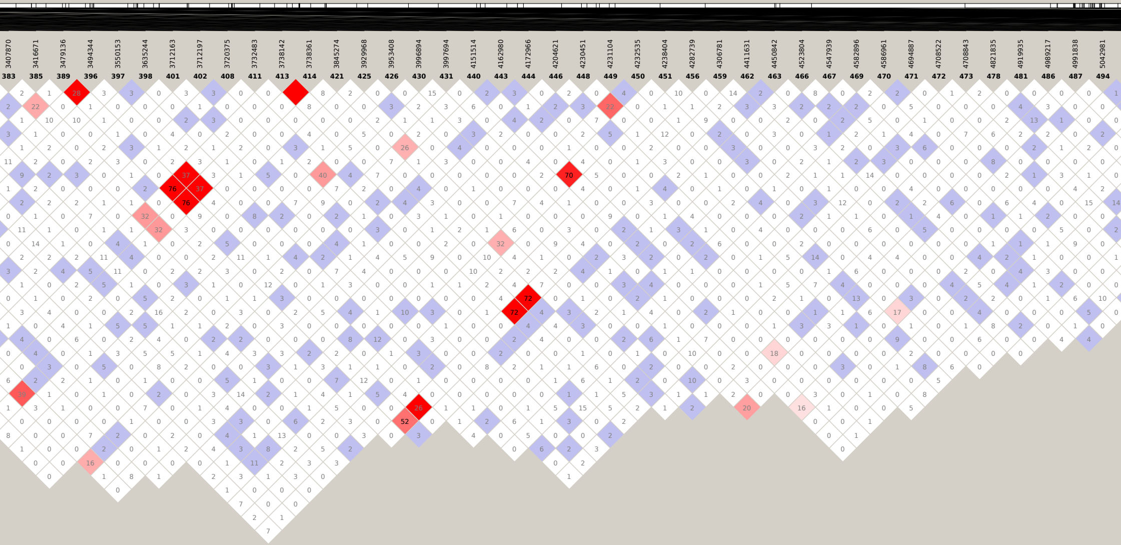
3407870
383
3416671
385
3479136
389
3494344
396
3550153
397
3635244
398
3712163
401
3712197
402
3720375
408
3732483
411
3738142
413
3738361
414
3845274
421
3929968
425
3953408
426
3996894
430
3997694
431
4151514
440
4162980
443
4172966
444
4204621
446
4230451
448
4231104
449
4232535
450
4238404
451
4282739
456
4306781
459
4411631
462
4450842
463
4523804
466
4547939
467
4582896
469
4586961
470
4694887
471
4708522
472
4708843
473
4821835
478
4919935
481
4989217
486
4991838
487
5042981
494

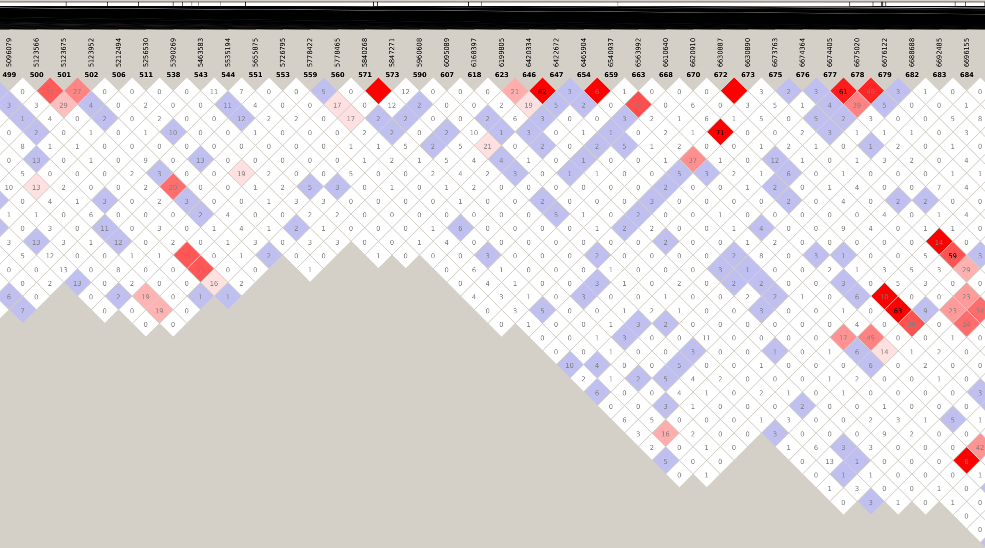
5096079
5123566
5123675
5123952
5212494
5256530
5390269
5463583
5535194
5655875
5726795
5778422
5778465
5840268
5847271
5960608
6095089
6168397
6199805
6420334
6422672
6465904
6540937
6563992
6610640
6620910
6630887
6630890
6673763
6674364
6674405
6675020
6676122
6688688
6692485
6696155
499
500
501
502
506
511
538
543
544
551
553
559
560
571
573
590
607
618
623
646
647
654
659
663
668
670
672
673
675
676
677
678
679
682
683
684

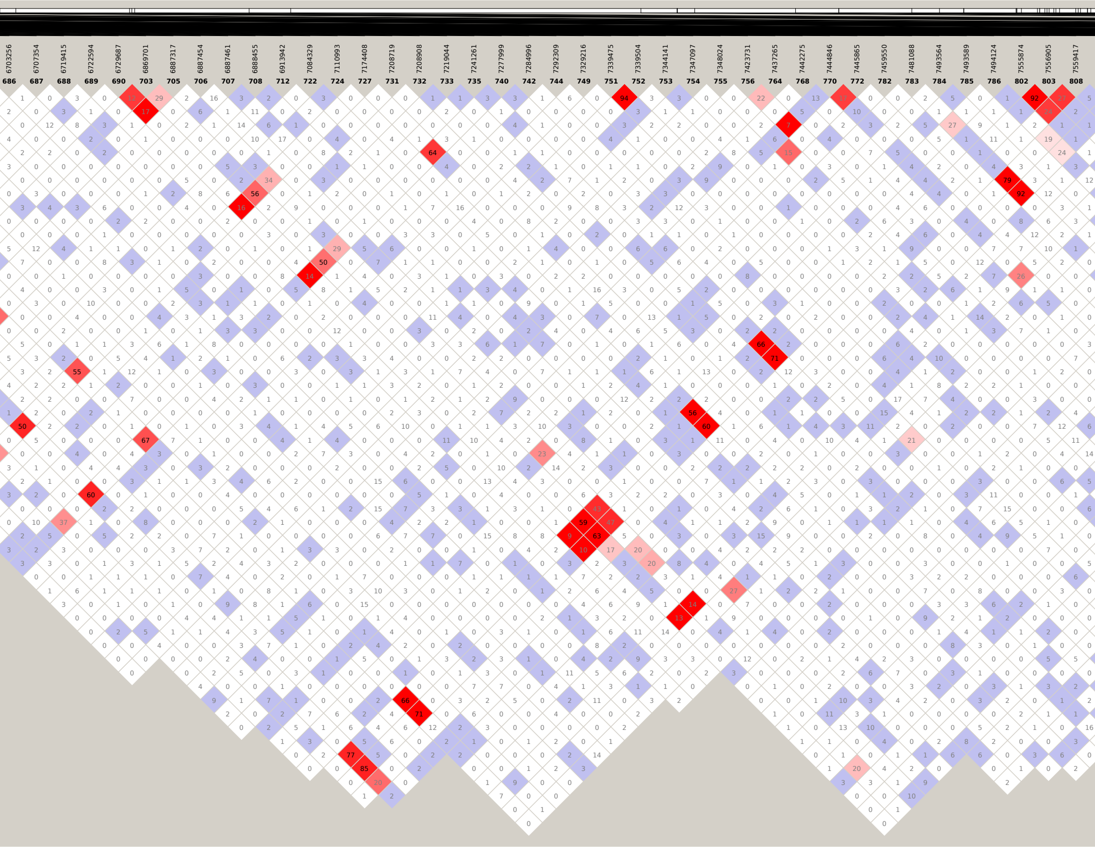
6703256
686
6707354
687
6719415
688
6722594
689
6729687
690
6869701
703
6887317
705
6887454
706
6887461
707
6888455
708
6913942
712
7084329
722
7110993
724
7174408
727
7208719
731
7208908
732
7219044
733
7241261
735
7277999
740
7284996
742
7292309
744
7329216
749
7339475
751
7339504
752
7344141
753
7347097
754
7348024
755
7423731
756
7437265
764
7442275
768
7444846
770
7445865
772
7459550
782
7481088
783
7493564
784
7493589
785
7494124
786
7555874
802
7556905
803
7559417
808

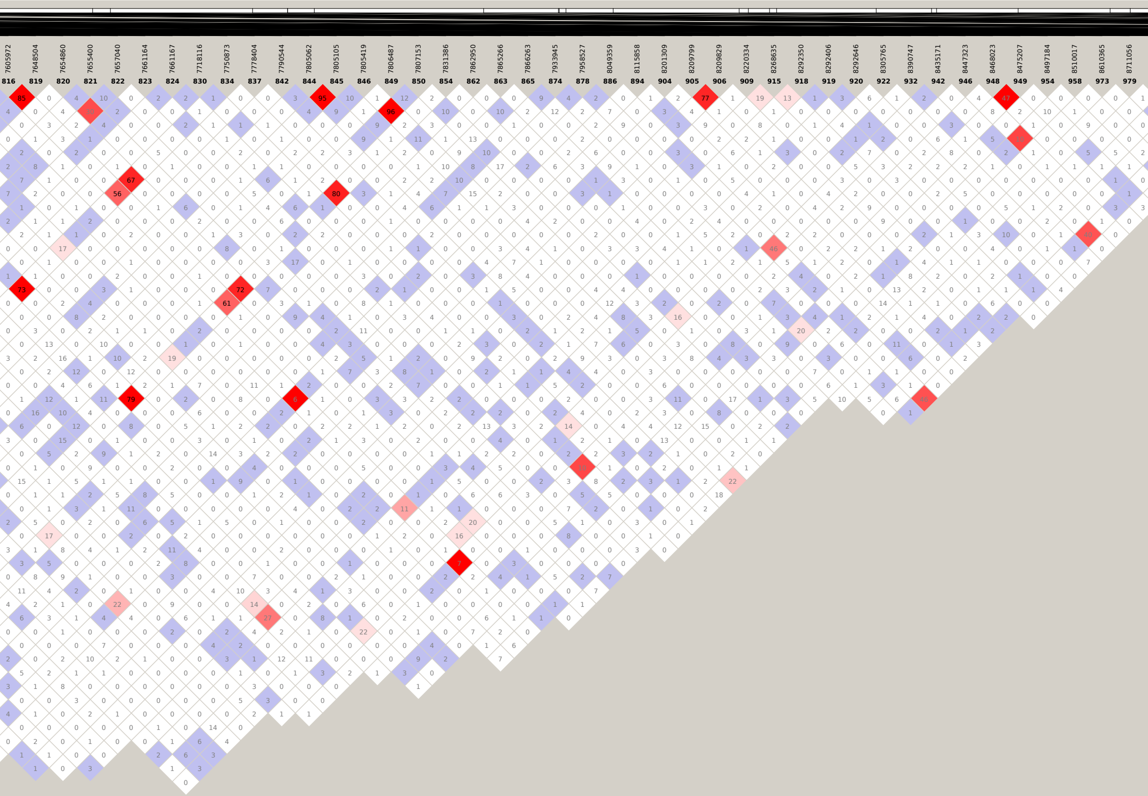
7605972 816
7648504 819
7654860 820
7655400 821
7657040 822
7661164 823
7661167 824
7718116 830
7750873 834
7778404 837
7790544 842
7805062 844
7805105 845
7805419 846
7806487 849
7807153 850
7831386 854
7862950 862
7865266 863
7866263 865
7933945 874
7958527 878
8049359 886
8115858 894
8201309 904
8209799 905
8209829 906
8220334 909
8268635 915
8292350 918
8292406 919
8292646 920
8305765 922
8390747 932
8435171 942
8447323 946
8468023 948
8475207 949
8497184 954
8510017 958
8610365 973
8711056 979

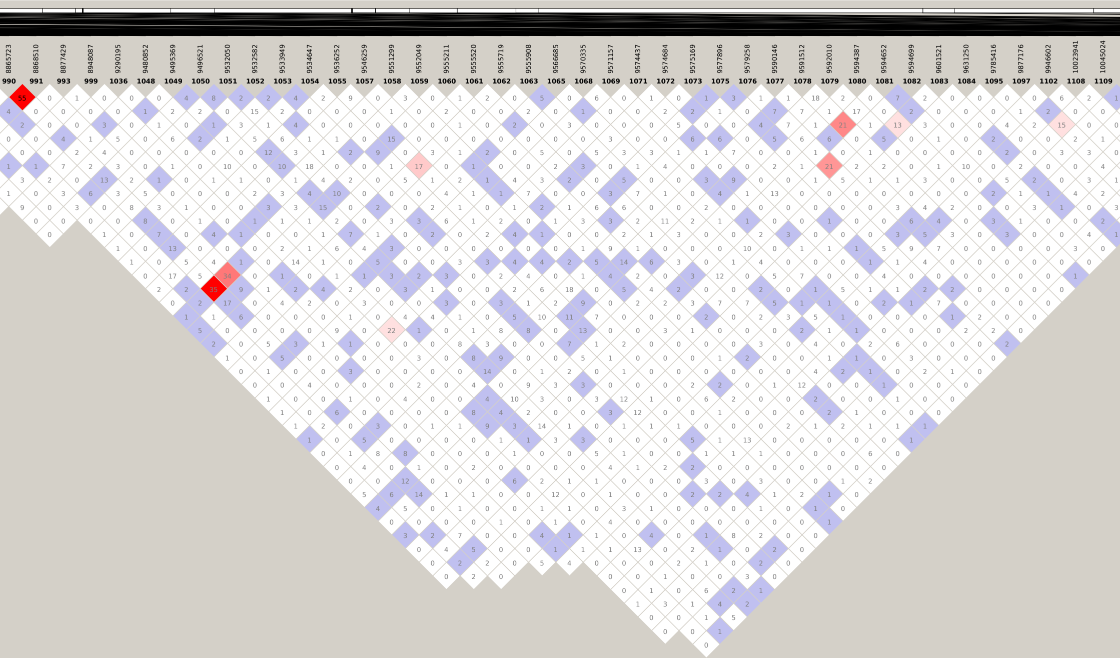
8865723 8868510 8877429 8948087 9290195 9480852 9495369 9496521 9532050 9532582 9533949 9534647 9536252 9546259 9551299 9552049 9555211 9555520 9555719 9555908 9566685 9570335 9571157 9574437 9574684 9575169 9577896 9579258 9590146 9591512 9592010 9594387 9594652 9594699 9601521 9631250 9785416 9877176 9946602 10023941 10045024
990 991 993 999 1036 1048 1049 1050 1051 1052 1053 1054 1055 1057 1058 1059 1060 1061 1062 1063 1065 1068 1069 1071 1072 1073 1075 1076 1077 1078 1079 1080 1081 1082 1083 1084 1095 1097 1102 1108 1109

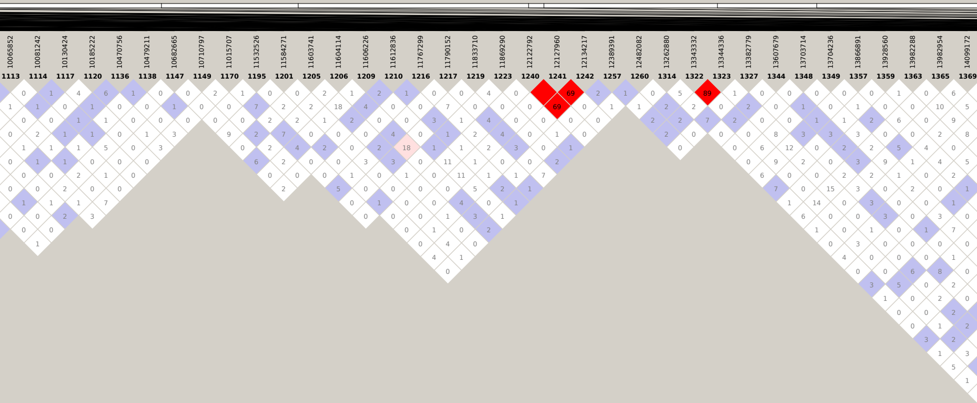
10065852 10081242 10130424 10185222 10470756 10479211 10682665 10710797 11015707 11532526 11584271 11603741 11604114 11606226 11612836 11767299 11790152 11833710 11869290 12122792 12127960 12134217 12389391 12482082 13262880 13343332 13344336 13382779 13607679 13703714 13704236 13866891 13928560 13982288 13982954 14099172
1113 1114 1117 1120 1136 1138 1147 1149 1170 1195 1201 1205 1206 1209 1210 1216 1217 1219 1223 1240 1241 1242 1257 1260 1314 1322 1323 1327 1344 1348 1349 1357 1359 1363 1365 1369

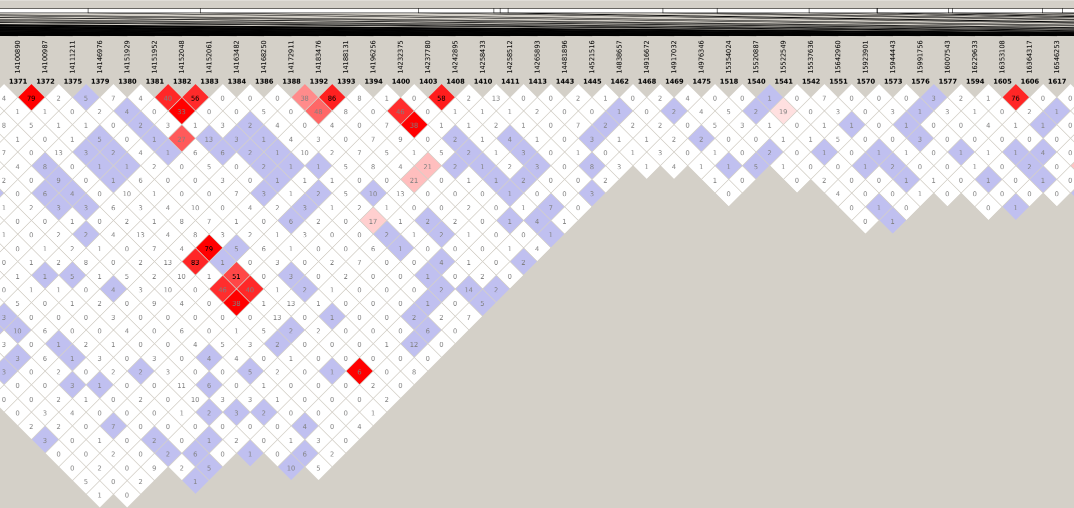
14100890
1371
14100987
1372
14111211
1375
14146976
1379
14151929
1380
14151952
1381
14152048
1382
14152061
1383
14163482
1384
14168250
1386
14172911
1388
14183476
1392
14188131
1393
14196256
1394
14232375
1400
14237780
1403
14242895
1408
14258433
1410
14258512
1411
14265893
1413
14481896
1443
14521516
1445
14838657
1462
14916672
1468
14917032
1469
14976346
1475
15354024
1518
15520887
1540
15522549
1541
15537636
1542
15642960
1551
15923901
1570
15944443
1573
15991756
1576
16007543
1577
16229633
1594
16353108
1605
16364317
1606
16546253
1617

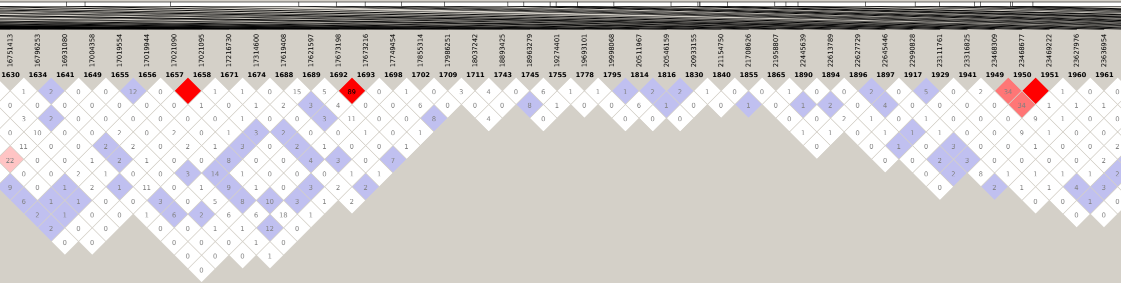
16751413
16796253
16931080
17004358
17019554
17019944
17021090
17021095
17216730
17314600
17619408
17621597
17673198
17673216
17749454
17855314
17986251
18037242
18893425
18963279
19274401
19693101
19998068
20511967
20546159
20933155
21154750
21708626
21958807
22445639
22613789
22627729
22645446
22990828
23111761
23316825
23468309
23468677
23469222
23627976
23636954
1630
1634
1641
1649
1655
1656
1657
1658
1671
1674
1688
1689
1692
1693
1698
1702
1709
1711
1743
1745
1755
1778
1795
1814
1816
1830
1840
1855
1865
1890
1894
1896
1897
1917
1929
1941
1949
1950
1951
1960
1961

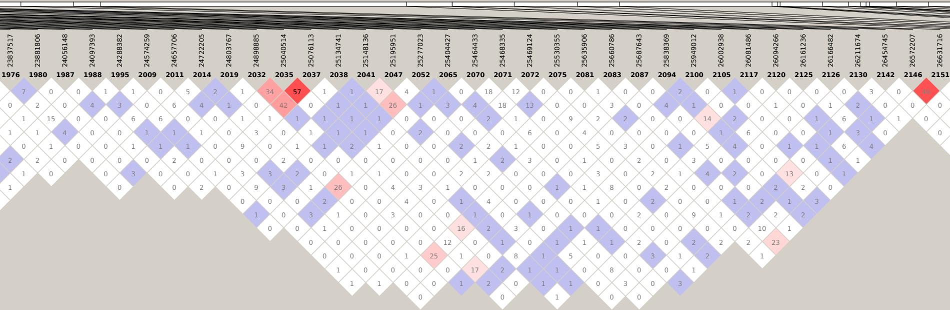
23837517 23881806 24056148 24097393 24288382 24574259 24657706 24722205 24803767 24898885 25040514 25076113 25134741 25148136 25195951 25277023 25404427 25464433 25468335 25469124 25530355 25635906 25660786 25687643 25838369 25949012 26002938 26081486 26094266 26161236 26166482 26211674 26454745 26572207 26631716
1976 1980 1987 1988 1995 2009 2011 2014 2019 2032 2035 2037 2038 2041 2047 2052 2065 2070 2071 2072 2075 2081 2083 2087 2094 2100 2105 2117 2120 2125 2126 2130 2142 2146 2151

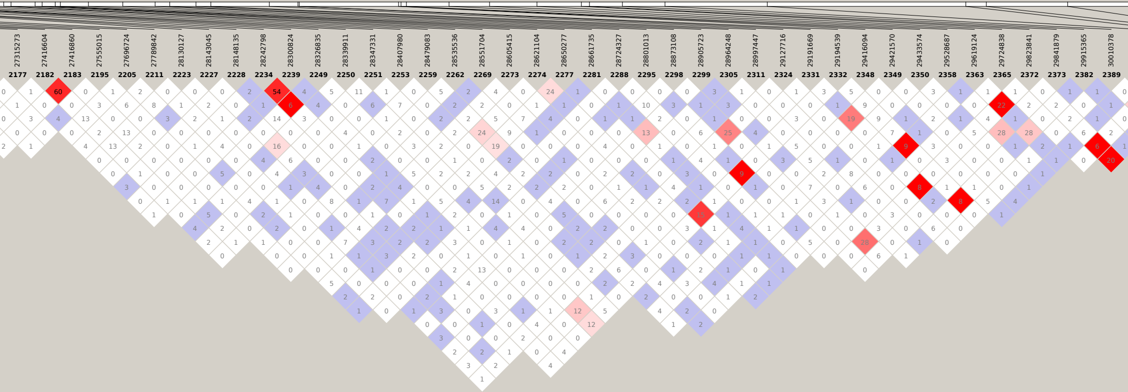
27315273 27416604 27416860 27555015 27696724 27789842 28130127 28143045 28148135 28242798 28300824 28326835 28339911 28347331 28407980 28479083 28535536 28551704 28605415 28621104 28650277 28661735 28724327 28801013 28873108 28905723 28964248 28997447 29127716 29191669 29194539 29416094 29421570 29433574 29528687 29619124 29724838 29823841 29841879 29915365 30010378
2177 2182 2183 2195 2205 2211 2223 2227 2228 2234 2239 2249 2250 2251 2253 2259 2262 2269 2273 2274 2277 2281 2288 2295 2298 2299 2305 2311 2324 2331 2332 2348 2349 2350 2358 2363 2365 2372 2373 2382 2389

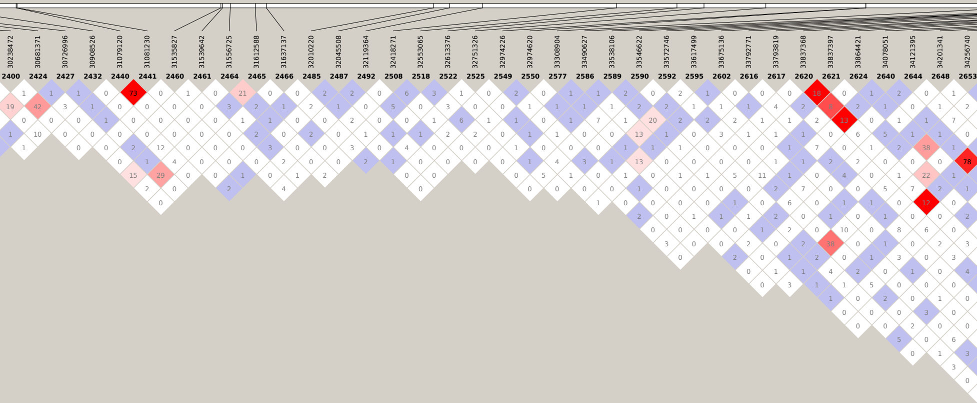

30238472
30681371
30726996
30908526
31079120
31081230
31535827
31539642
31556725
31612588
31637137
32010220
32045508
32119364
32418271
32553065
32613376
32751326
32974226
32974620
33308904
33490627
33538106
33546622
33572746
33617499
33675136
33792771
33793819
33837368
33837397
33864421
34078051
34121395
34201341
34256740
2400 2424 2427 2432 2440 2441 2460 2461 2464 2465 2466 2485 2487 2492 2508 2518 2522 2525 2549 2550 2577 2586 2589 2590 2592 2595 2602 2616 2617 2620 2621 2624 2640 2644 2648 2653

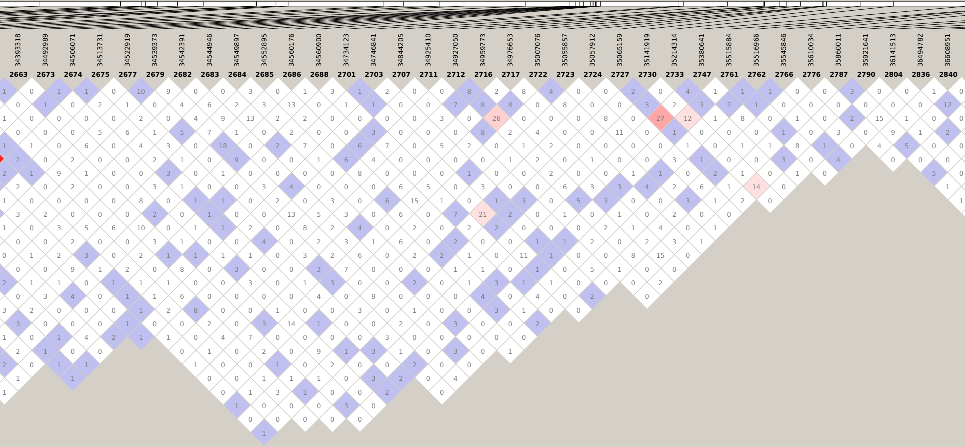
34393318
34492989
34506071
34513731
34522919
34539373
34542391
34544946
34549897
34552895
34560176
34560900
34734123
34746841
34844205
34925410
34927050
34959773
34976653
35007076
35055857
35057912
35065159
35141919
35214314
35380641
35515884
35516966
35545846
35610034
35860011
35921641
36141513
36494782
36608951
2663
2673
2674
2675
2677
2679
2682
2683
2684
2685
2686
2688
2701
2703
2707
2711
2712
2716
2717
2722
2723
2724
2727
2730
2733
2747
2761
2762
2766
2776
2787
2790
2804
2836
2840

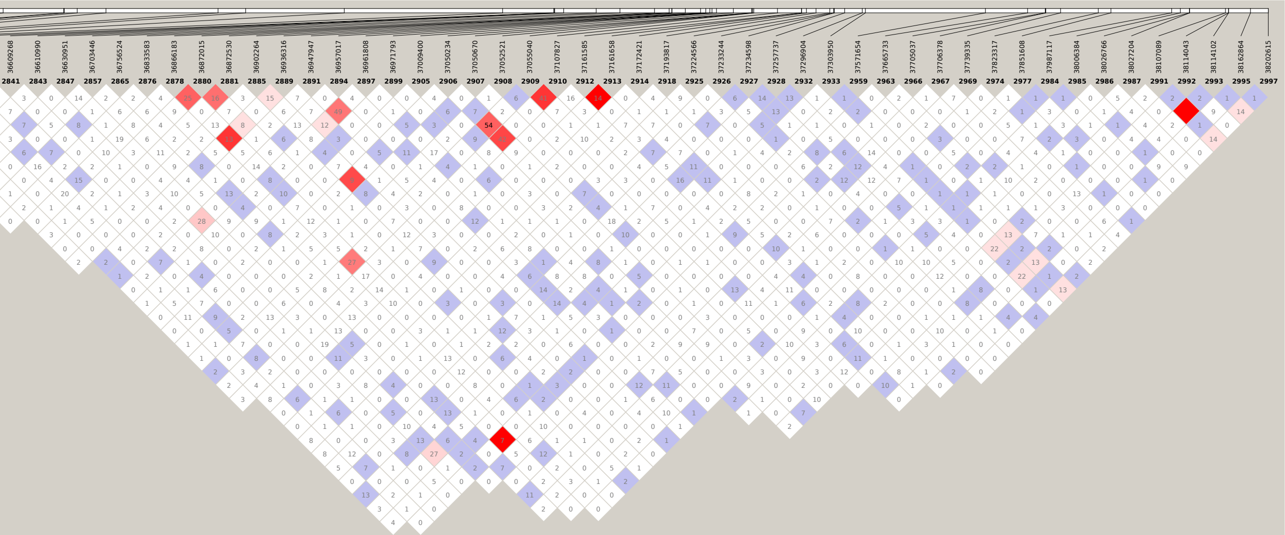
36609268 36610990 36630951 36703446 36756524 36833583 36866183 36872015 36872530 36902264 36936316 36947947 36957017 36961808 36971793 37009400 37050234 37050670 37052521 37055040 37107827 37161585 37161658 37172421 37193817 37224566 37233244 37234598 37257737 37296904 37303950 37571654 37665733 37705037 37706378 37739335 37823317 37851608 37987117 38006384 38026766 38027204 38107089 38114043 38114102 38162864 38202615
2841 2843 2847 2857 2865 2876 2878 2880 2881 2885 2889 2891 2894 2897 2899 2905 2906 2907 2908 2909 2910 2912 2913 2914 2918 2925 2926 2927 2928 2932 2933 2959 2963 2966 2967 2969 2974 2977 2984 2985 2986 2987 2991 2992 2993 2995 2997

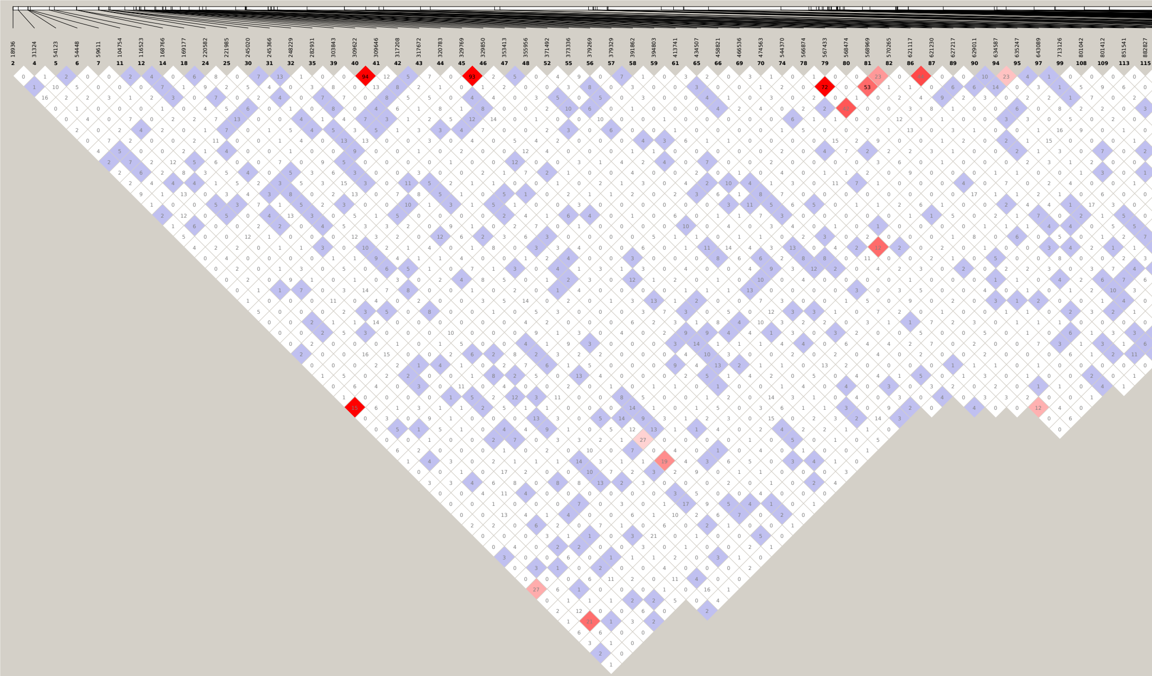

18936 2
31324 4
54123 5
54448 6
59611 7
104754 11
116523 12
168766 14
169177 18
220582 24
221985 25
245020 30
245366 31
248229 32
282931 35
303843 39
309622 40
309646 41
317208 42
317672 43
320783 44
329769 45
329850 46
353413 47
355956 48
371492 52
373336 55
379269 56
379329 57
391862 58
394803 59
413741 61
434507 65
458821 66
466536 69
474563 70
544370 74
566874 78
567433 79
568474 80
568969 81
570265 82
621117 86
621230 87
627217 89
629011 90
634587 94
635247 95
643089 97
713126 99
801042 108
801412 109
851541 113
882827 115

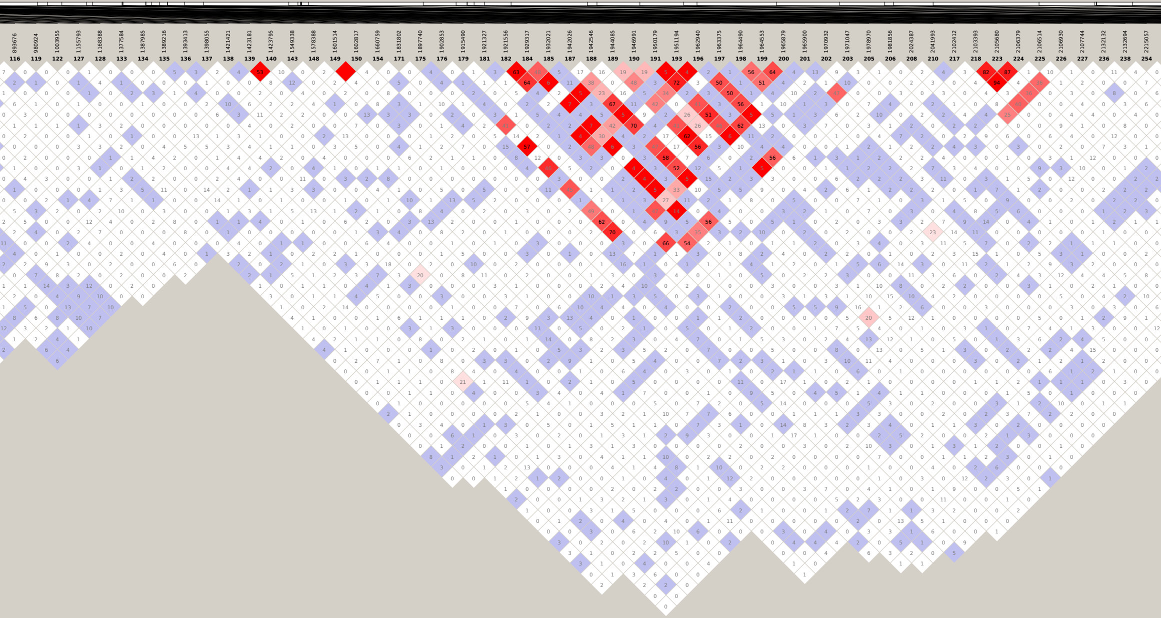

893676 116
980924 119
1003955 122
1155793 127
1168388 128
1377584 133
1387985 134
1389216 135
1393413 136
1398055 137
1421421 138
1423181 139
1423795 140
1549338 143
1578388 148
1601514 149
1602817 150
1660759 154
1831802 171
1897740 175
1902853 176
1915490 179
1921327 181
1921556 182
1929317 184
1932021 185
1942026 187
1942546 188
1944085 189
1946991 190
1950179 191
1951194 193
1962940 196
1963375 197
1964490 198
1964553 199
1965879 200
1965900 201
1970932 202
1971047 203
1978970 205
1981856 206
2024387 208
2041993 210
2102412 217
2103393 218
2105680 223
2106379 224
2106514 225
2106930 226
2107744 227
2132132 236
2132694 238
2215057 254

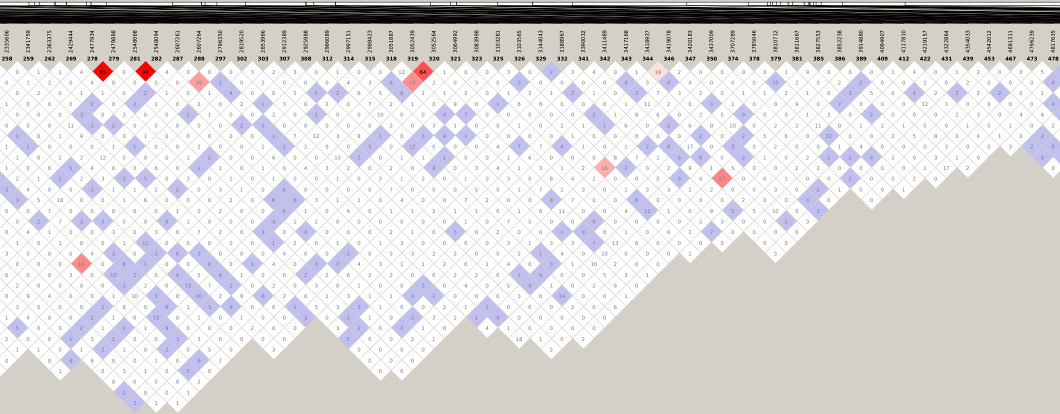
2335606 2341759 2363375 2429444 2477934 2479888 2548068 2548094 2607261 2607264 2796350 2819520 2853966 2912389 2925988 2996089 2997151 2998423 3051887 3052439 3052564 3064992 3083998 3103281 3103565 3144043 3188967 3390032 3411489 3417168 3418937 3419078 3420183 3437009 3707289 3785046 3810712 3811667 3827553 3852238 3914880 4094907 4117810 4219157 4322864 4354033 4542012 4691311 4796239 4817635
258 259 262 269 278 279 281 282 287 288 297 302 303 307 308 312 314 315 318 319 320 321 323 325 326 329 332 341 342 343 344 346 347 350 374 378 379 381 385 386 389 409 412 422 431 439 453 467 473 478

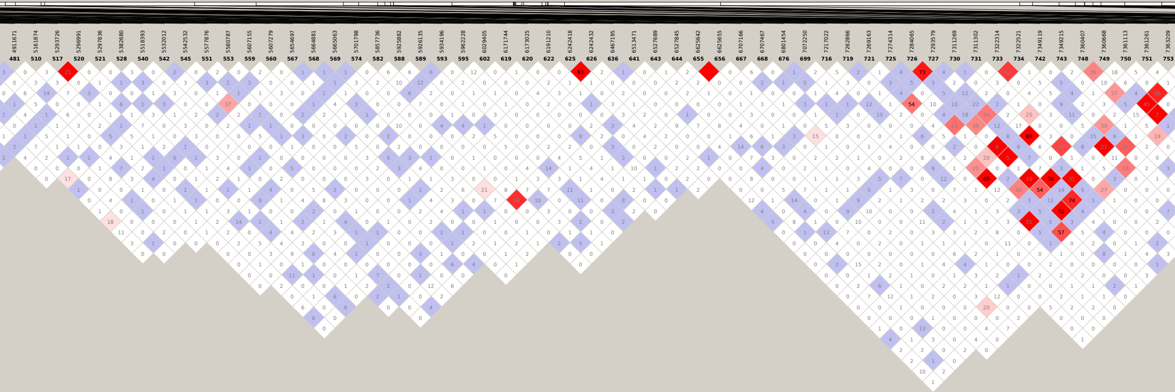
4911671 481
5161874 510
5293726 517
5296991 520
5297836 521
5382680 528
5518393 540
5532012 542
5542532 545
5577876 551
5580787 553
5607155 559
5607279 560
5654697 567
5664881 568
5665063 569
5701798 574
5857736 582
5925882 588
5926135 589
5934196 593
5962228 595
6029405 602
6171744 619
6173025 620
6191210 622
6242418 625
6242432 626
6467195 636
6513471 641
6527689 643
6527845 644
6625642 655
6625655 656
6707166 667
6707467 668
6801454 676
7072250 699
7217022 716
7262866 719
7269163 721
7274314 725
7284065 726
7293579 727
7311269 730
7311302 731
7322314 733
7322521 734
7349119 742
7349215 743
7360407 748
7360668 749
7361113 750
7361261 751
7363209 753

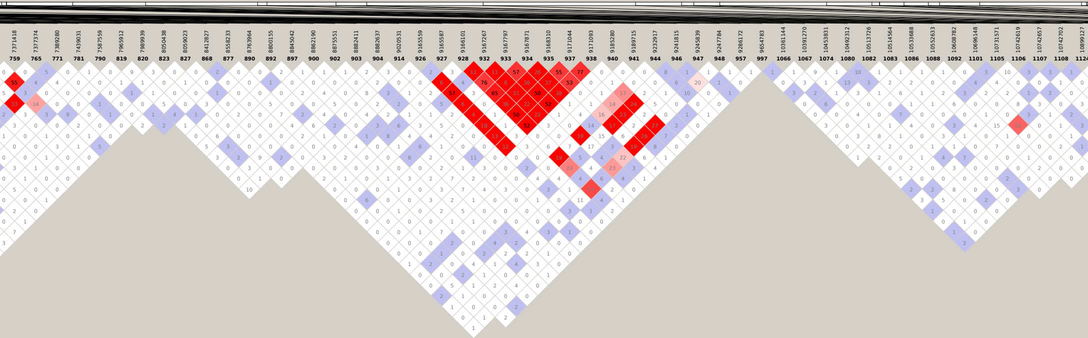

7371418 7377374 7389280 7439031 7587559 7965912 7989939 8050438 8059023 8412827 8558233 8763964 8800155 8845042 8862190 8875551 8882411 8882637 9020531 9165559 9165587 9166101 9167267 9167797 9167871 9168310 9171044 9171093 9185280 9189715 9232917 9241815 9245839 9247784 9286172 9654783 10361144 10391270 10453831 10492312 10513726 10514564 10533688 10552633 10608782 10696148 10731571 10742619 10742657 10742702 10899127
759 765 771 781 790 819 820 823 827 868 877 890 892 897 900 902 903 904 914 926 927 928 932 933 934 935 937 938 940 941 944 946 947 948 957 997 1066 1067 1074 1080 1082 1083 1086 1088 1092 1101 1105 1106 1107 1108 1124

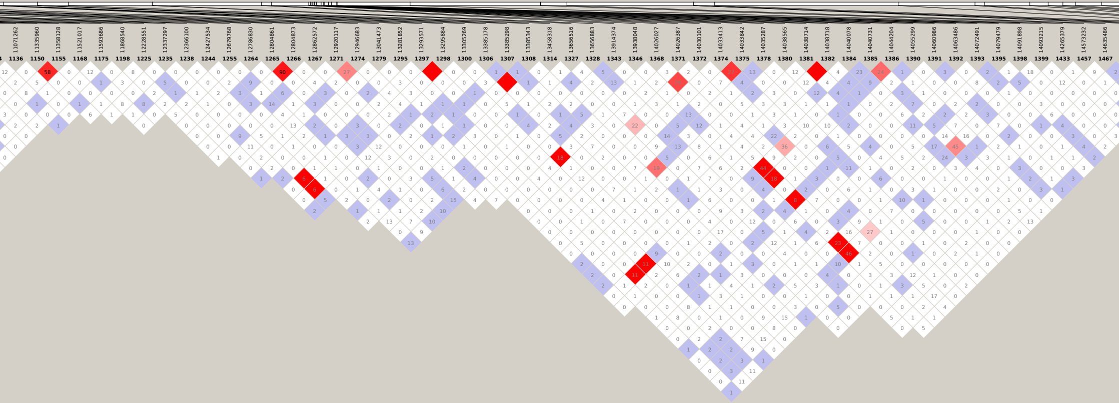
11071262
11335960
11358128
11521017
11593686
11868540
12228551
12337297
12366100
12427534
12679768
12786830
12804861
12804873
12862572
12920117
12946683
13041473
13281852
13293571
13295884
13305269
13385178
13385298
13385343
13458318
13656516
13656883
13914374
13938048
14026027
14026387
14030101
14033413
14033842
14035287
14038565
14038714
14038718
14040078
14040731
14044204
14055299
14060986
14063486
14072491
14079479
14091898
14093215
14265379
14573232
14635486
1136 1150 1155 1168 1175 1198 1225 1235 1238 1244 1255 1264 1265 1266 1267 1271 1274 1279 1295 1297 1298 1300 1306 1307 1308 1314 1327 1328 1343 1346 1368 1371 1372 1374 1375 1378 1380 1381 1382 1384 1385 1386 1390 1391 1392 1393 1395 1398 1399 1433 1457 1467

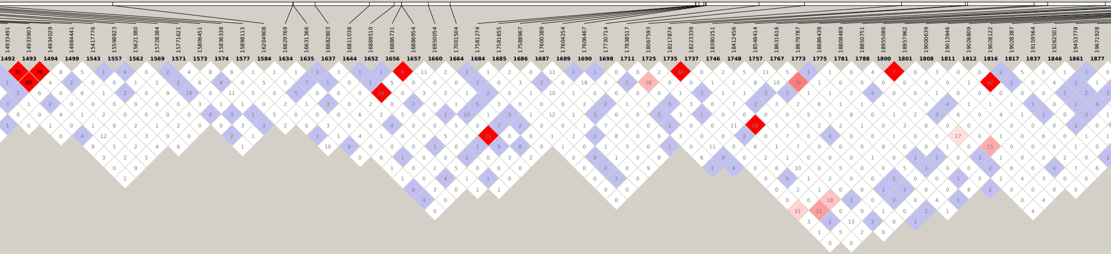
14933491 14933903 14934029 14984441 15417776 15598923 15621380 15728384 15771621 15806451 15836338 15898115 16204908 16629769 16631366 16682803 16811038 16869510 16886731 16886954 16950054 17001504 17581274 17581655 17588967 17600389 17604254 17606467 17730714 17838517 18067593 18217874 18223339 18380251 18412456 18548414 18631616 18679787 18686439 18808489 18850751 18955086 18957962 19000639 19013946 19026809 19028122 19028387 19159564 19262501 19453778 19671928
1492 1493 1494 1499 1543 1557 1562 1569 1571 1573 1574 1577 1584 1634 1635 1637 1644 1652 1656 1657 1660 1664 1684 1685 1686 1687 1689 1690 1698 1711 1725 1735 1737 1746 1748 1757 1767 1773 1775 1781 1788 1800 1801 1808 1811 1812 1816 1817 1837 1846 1861 1877

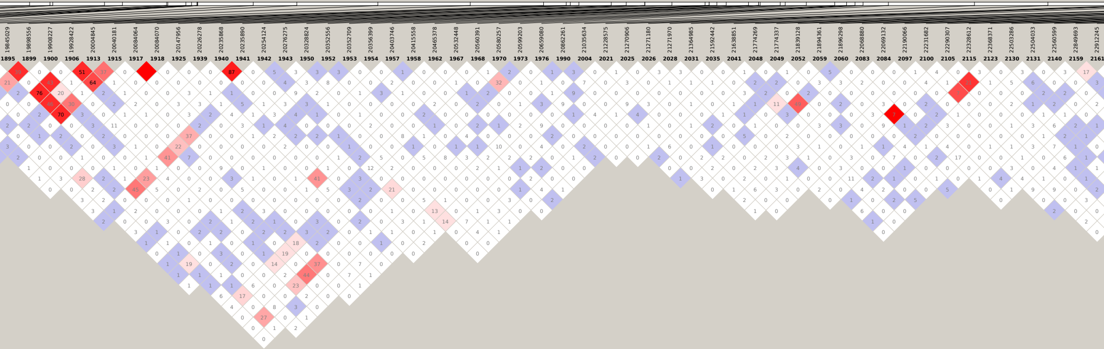
19845029 19898556 19908227 19928422 20004845 20040181 20084064 20084070 20147956 20226278 20235868 20235890 20254124 20276275 20328824 20352556 20352709 20356399 20403746 20415558 20465378 20532448 20560391 20580257 20599203 20659080 20862261 21035634 21228575 21270906 21271180 21271970 21364985 21592442 21638851 21774269 21774337 21839128 21894361 21896298 22068880 22069132 22190066 22231682 22290307 22328612 22368371 22503286 22504033 22560599 22849693 22912245
1895 1899 1900 1906 1913 1915 1917 1918 1925 1939 1940 1941 1942 1943 1950 1952 1953 1954 1957 1958 1962 1967 1968 1970 1973 1976 1990 2004 2021 2025 2026 2028 2031 2035 2041 2048 2049 2052 2059 2060 2083 2084 2097 2100 2105 2115 2123 2130 2131 2140 2159 2161

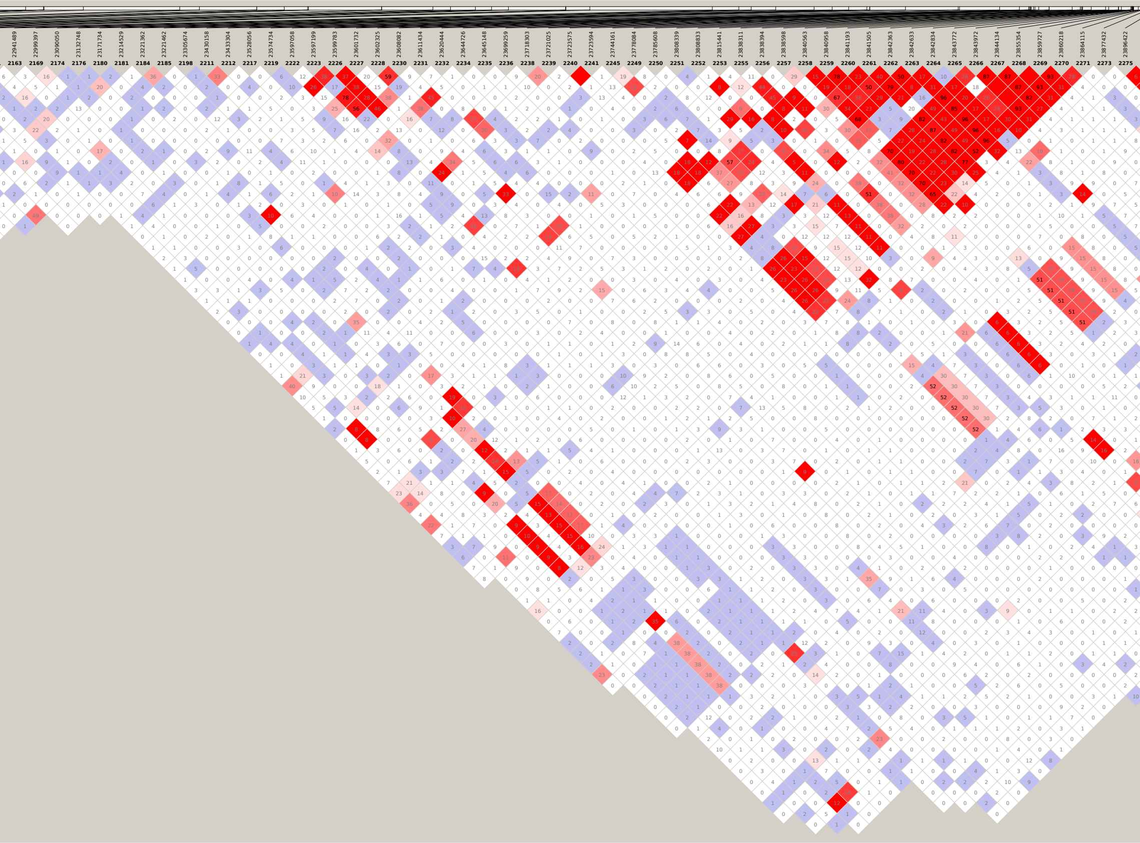
22941489
22999397
23090050
23132748
23171734
23214529
23221362
23221462
23305674
23430158
23433304
23528056
23574734
23597058
23597199
23599783
23601732
23602325
23608082
23611434
23620444
23644726
23645148
23699259
23718303
23721025
23723575
23723594
23744161
23778084
23785608
23808339
23808833
23815441
23838311
23838394
23838598
23840563
23840958
23841193
23841505
23842363
23842633
23842834
23843772
23843972
23844134
23855354
23859727
23860218
23864115
23877432
23896422
2163
2169
2174
2176
2180
2181
2184
2185
2198
2211
2212
2217
2219
2222
2223
2226
2227
2228
2230
2231
2232
2234
2235
2236
2238
2239
2240
2241
2245
2249
2250
2251
2252
2253
2255
2256
2257
2258
2259
2260
2261
2262
2263
2264
2265
2266
2267
2268
2269
2270
2271
2273
2275

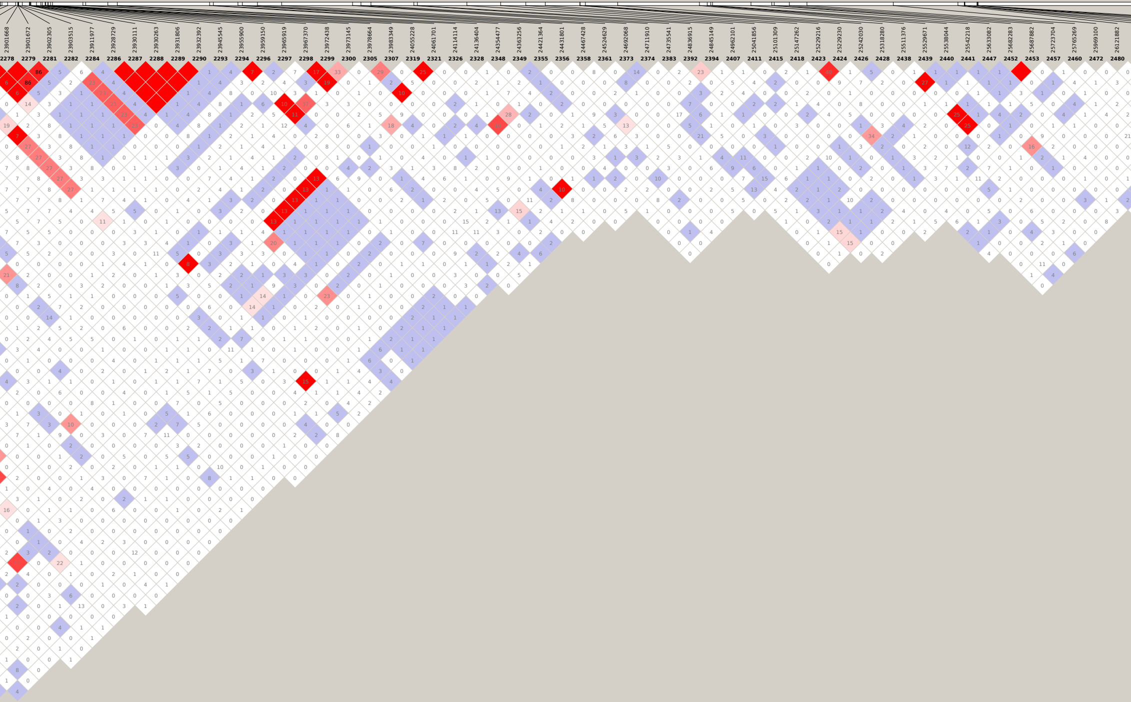
23901668 23901672 23902305 23903515 23911977 23928729 23930111 23930263 23931806 23932392 23945545 23955900 23959150 23965919 23967370 23972438 23973145 23978664 23983349 24055228 24061701 24114114 24136404 24354477 24363256 24421364 24431801 24467428 24524629 24692068 24711910 24735541 24836915 24845149 24962101 25041856 25101309 25147262 25229216 25229230 25242030 25318280 25511376 25529671 25538044 25542218 25633082 25682283 25687882 25723704 25765269 25969100 26121882
2278 2279 2281 2282 2284 2286 2287 2288 2289 2290 2293 2294 2296 2297 2298 2299 2300 2305 2307 2319 2321 2326 2328 2348 2349 2355 2356 2358 2361 2373 2374 2383 2392 2394 2407 2411 2415 2418 2423 2424 2426 2428 2438 2439 2440 2441 2447 2452 2453 2457 2460 2472 2480

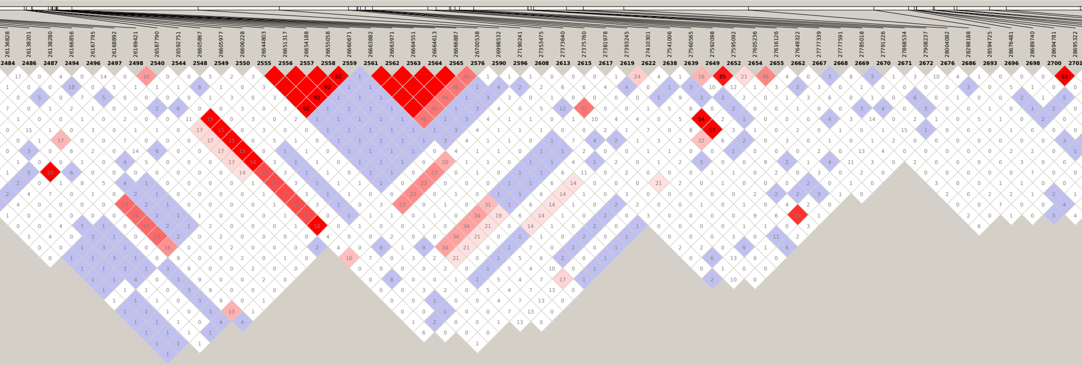

26136828 26138201 26138280 26166856 26167795 26168892 26169421 26587790 26592751 26605867 26605977 26606228 26644803 26651317 26654188 26655058 26660671 26663882 26663971 26664551 26664613 26666887 26700538 26998532 27190241 27353475 27373640 27375760 27381978 27393245 27410301 27541006 27560565 27592098 27595092 27605236 27616126 27649322 27777339 27777591 27785018 27791226 27868534 27908237 28004082 28298188 28594725 28676481 28689740 28694781 28695322
2484 2486 2487 2494 2496 2497 2498 2540 2544 2548 2549 2550 2555 2556 2557 2558 2559 2561 2562 2563 2564 2565 2576 2590 2596 2608 2613 2615 2617 2619 2622 2638 2639 2649 2652 2654 2655 2662 2667 2668 2669 2670 2671 2672 2676 2686 2693 2696 2698 2700 2701

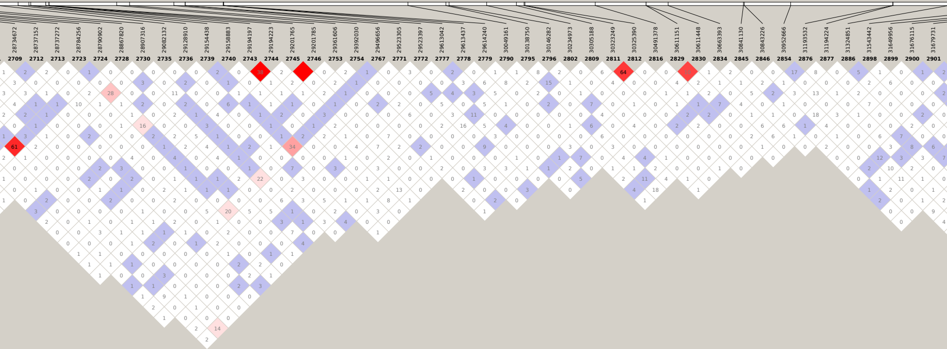
28734672 28737152 28737272 28784256 28790902 28867820 28907316 29082132 29128910 29154438 29158883 29194197 29194223 29201765 29201785 29361606 29392030 29496656 29523305 29523397 29613042 29613437 29614240 30049161 30138750 30146282 30234973 30305188 30323249 30325390 30491378 30611151 30611448 30663393 30841130 30843226 30952666 31193532 31194224 31324851 31543442 31649956 31676115 31679731
2709 2712 2713 2723 2724 2728 2730 2735 2736 2739 2740 2743 2744 2745 2746 2753 2754 2767 2771 2772 2777 2778 2779 2790 2795 2796 2802 2809 2811 2812 2816 2829 2830 2834 2845 2846 2854 2876 2877 2886 2898 2899 2900 2901

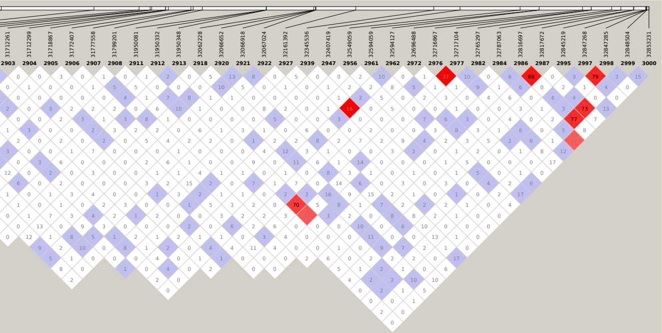
31712261
31712299
31718867
31772407
31777558
31799201
31950081
31950332
31950348
32062228
32066652
32066918
32067024
32161392
32345536
32407419
32549059
32594059
32594127
32696488
32716867
32717104
32765297
32787063
32816697
32817672
32845219
32847268
32847285
32848504
32853231
2903
2904
2905
2906
2907
2908
2911
2912
2913
2918
2920
2921
2922
2927
2939
2947
2956
2961
2962
2972
2976
2977
2982
2984
2986
2987
2995
2997
2998
2999
3000

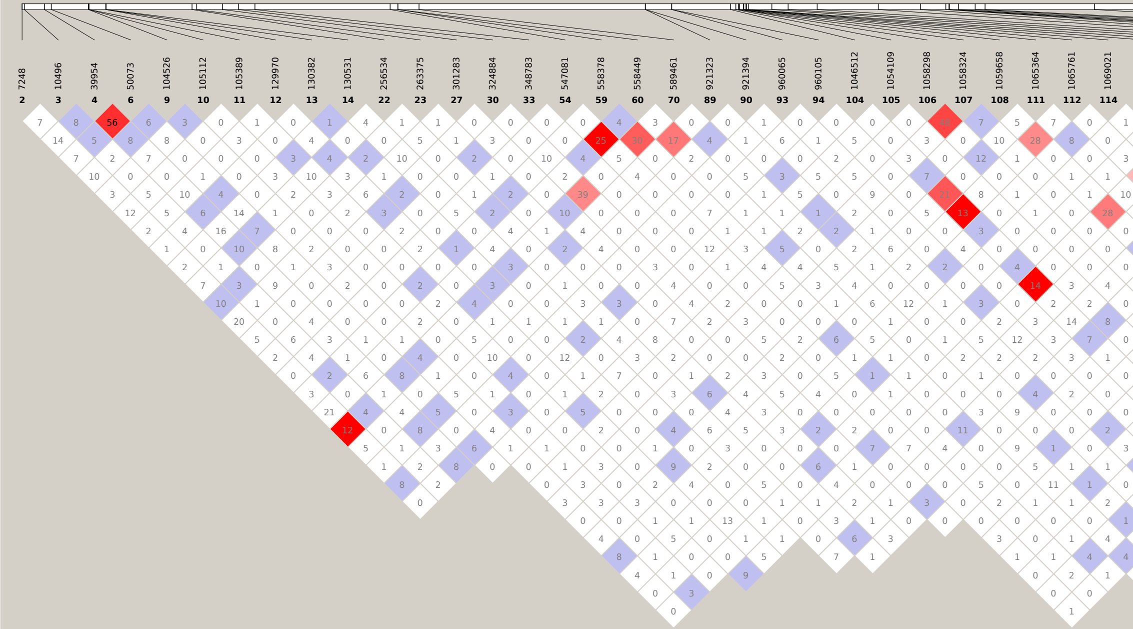

7248 10496 39954 50073 104526 105112 105389 129970 130382 130531 256534 263375 301283 324884 348783 547081 558378 558449 589461 921323 921394 960065 960105 1046512 1054109 1058298 1058324 1059658 1065364 1065761 1069021
2 3 4 6 9 10 11 12 13 14 22 23 27 30 33 54 59 60 70 89 90 93 94 104 105 106 107 108 111 112 114

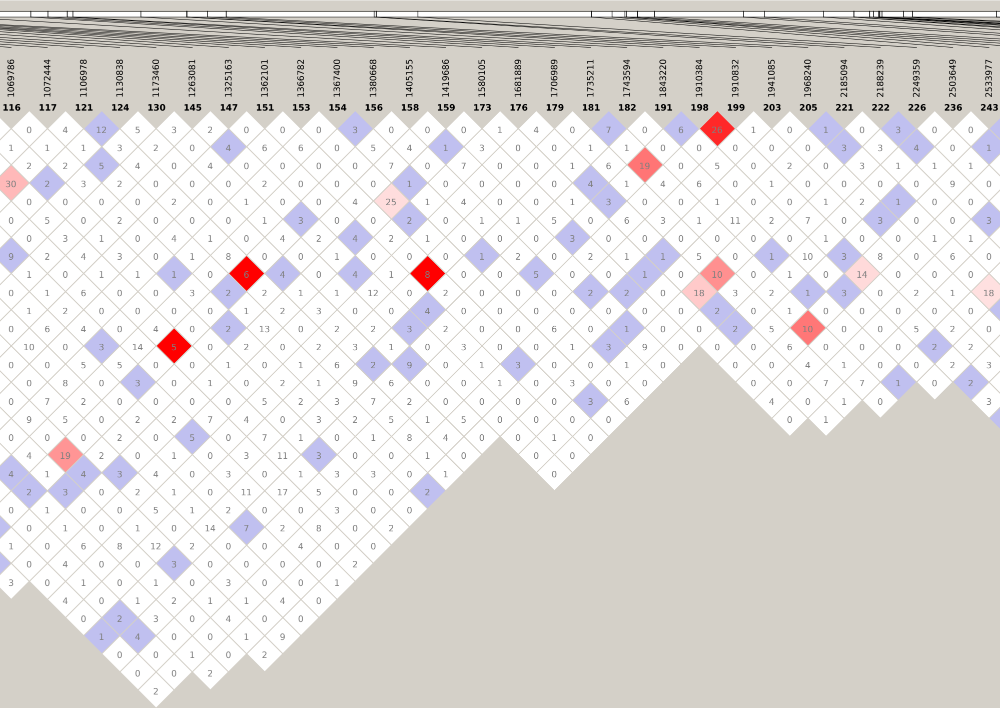
1069786 1072444 1106978 1130838 1173460 1263081 1325163 1362101 1366782 1367400 1380668 1405155 1419686 1580105 1681889 1706989 1735211 1743594 1843220 1910384 1910832 1941085 1968240 2185094 2188239 2249359 2503649 2533977
116 117 121 124 130 145 147 151 153 154 156 158 159 173 176 179 181 182 191 198 199 203 205 221 222 226 236 243

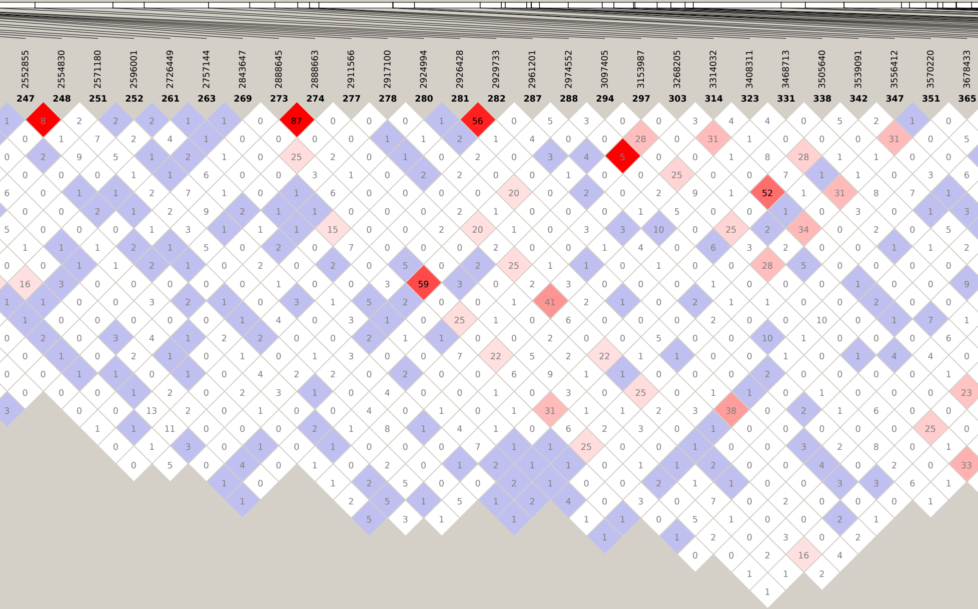
2552855
2554830
2571180
2596001
2726449
2757144
2843647
2888645
2888663
2911566
2917100
2924994
2926428
2929733
2961201
2974552
3097405
3153987
3268205
3314032
3408311
3468713
3505640
3539091
3556412
3570220
3678433
247
248
251
252
261
263
269
273
274
277
278
280
281
282
287
288
294
297
303
314
323
331
338
342
347
351
365

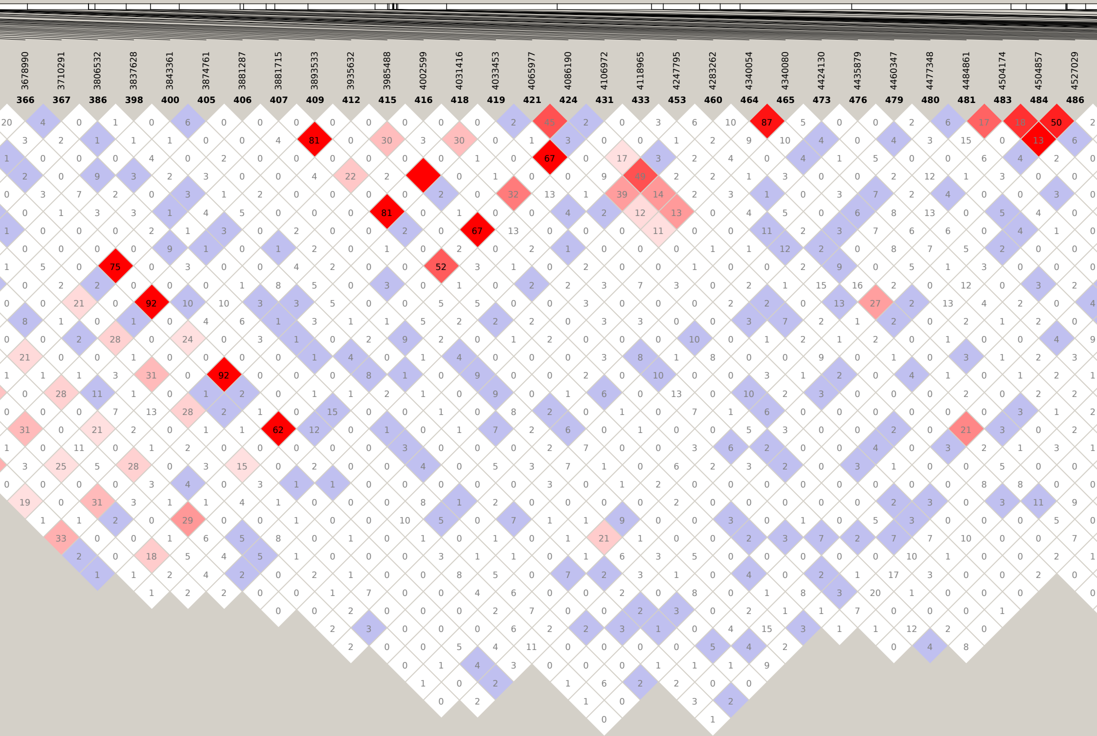
3678990
3710291
3806532
3837628
3843361
3874761
3881287
3881715
3893533
3935632
3985488
4002599
4031416
4033453
4065977
4086190
4106972
4118965
4247795
4283262
4340054
4340080
4424130
4435879
4460347
4477348
4484861
4504174
4504857
4527029
366
367
386
398
400
405
406
407
409
412
415
416
418
419
421
424
431
433
453
460
464
465
473
476
479
480
481
483
484
486

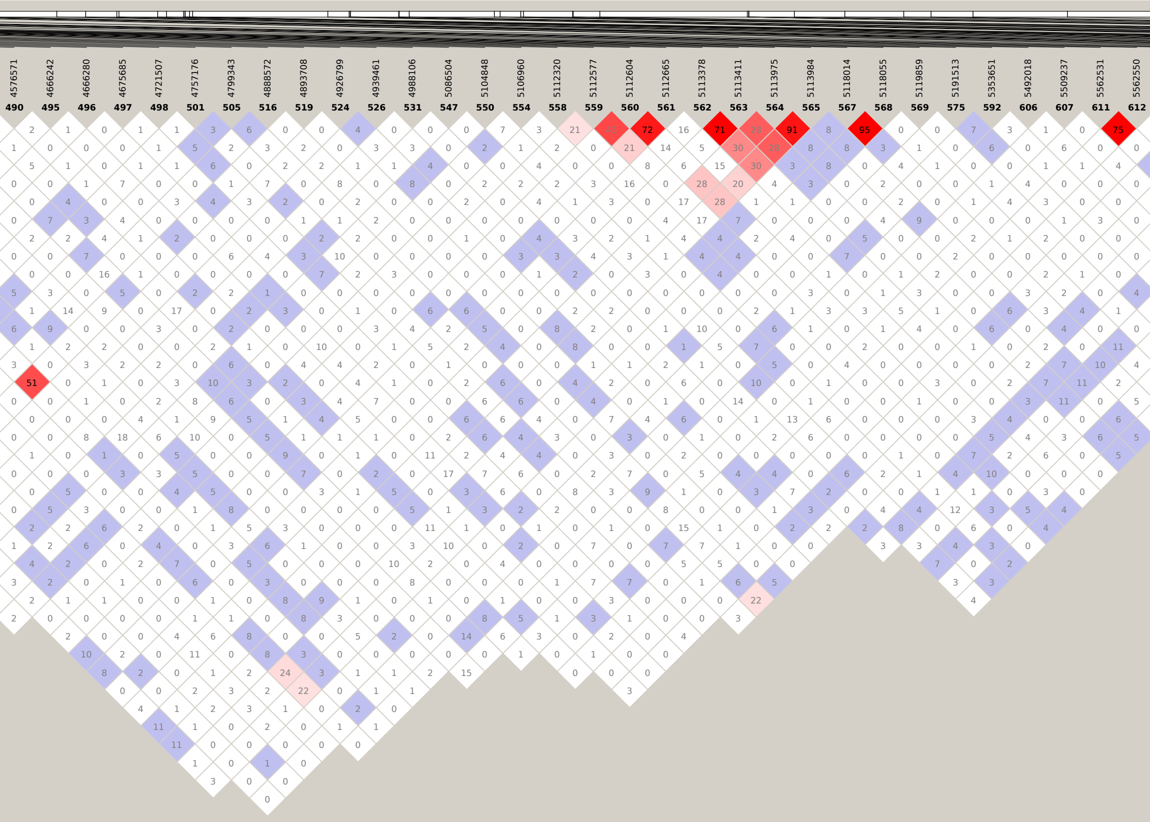
4576571 490
4666242 495
4666280 496
4675685 497
4721507 498
4757176 501
4799343 505
4888572 516
4893708 519
4926799 524
4939461 526
4988106 531
5086504 547
5104848 550
5106960 554
5112320 558
5112577 559
5112604 560
5112665 561
5113378 562
5113411 563
5113975 564
5113984 565
5118014 567
5118055 568
5119859 569
5191513 575
5353651 592
5492018 606
5509237 607
5562531 611
5562550 612

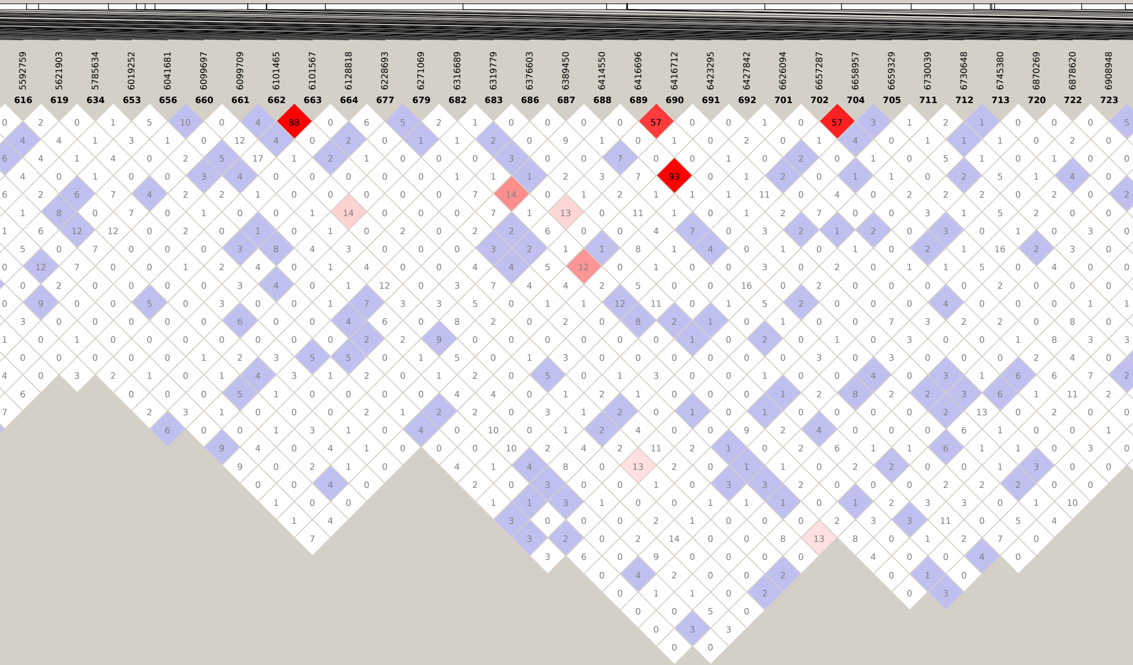
5592759
5621903
5785634
6019252
6041681
6099697
6099709
6101465
6101567
6128818
6228693
6271069
6316689
6319779
6376603
6389450
6414550
6416696
6416712
6423295
6427842
6626094
6657287
6658957
6659329
6730039
6730648
6745380
6870269
6878620
6908948
616
619
634
653
656
660
661
662
663
664
677
679
682
683
686
687
688
689
690
691
692
701
702
704
705
711
712
713
720
722
723

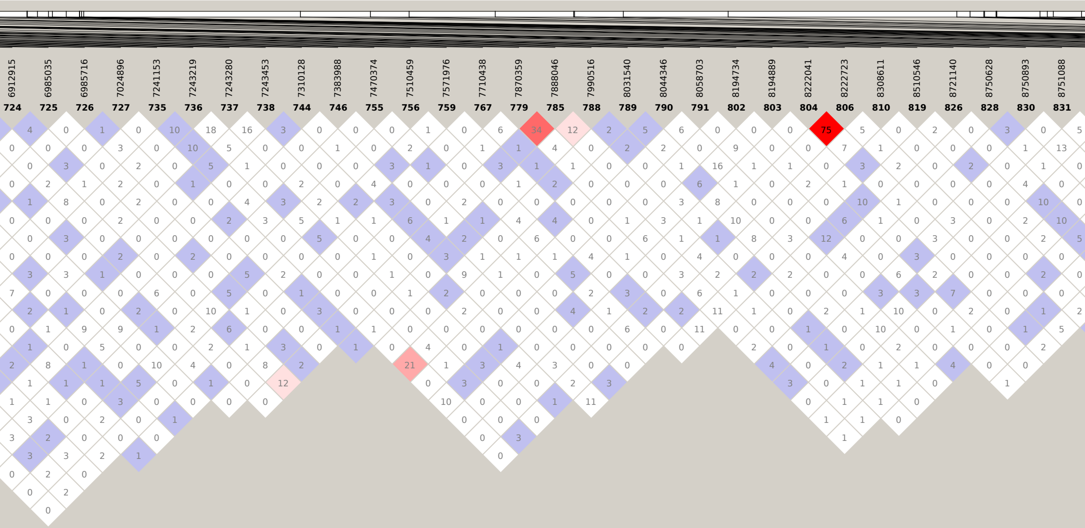

6912915 724
6985035 725
6985716 726
7024896 727
7241153 735
7243219 736
7243280 737
7243453 738
7310128 744
7383988 746
7470374 755
7510459 756
7571976 759
7710438 767
7870359 779
7888046 785
7990516 788
8031540 789
8044346 790
8058703 791
8194734 802
8194889 803
8222041 804
8222723 806
8308611 810
8510546 819
8721140 826
8750628 828
8750893 830
8751088 831

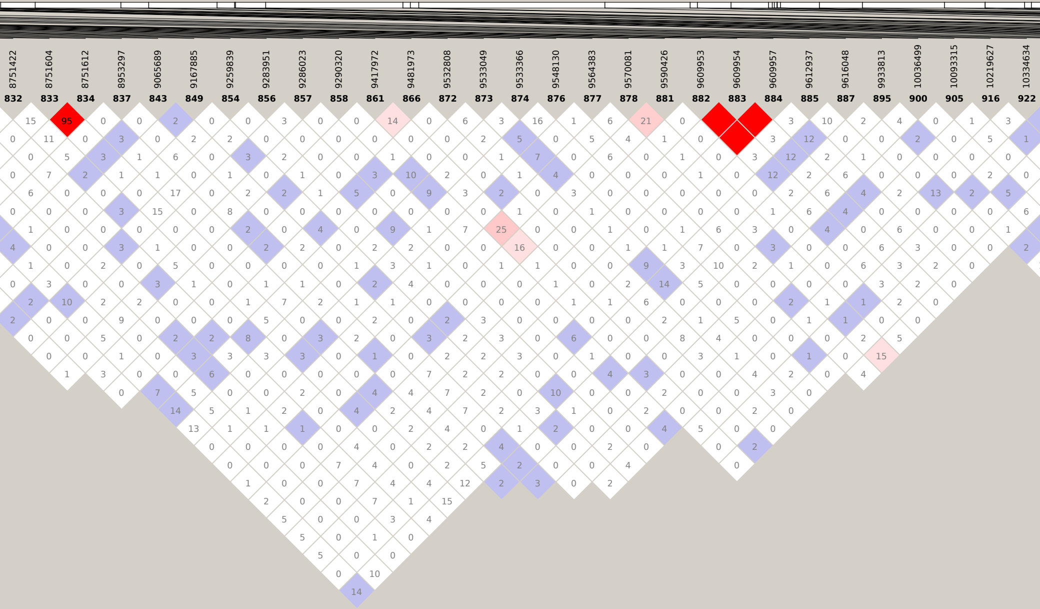
8751422 8751604 8751612 8953297 9065689 9167885 9259839 9283951 9286023 9290320 9417972 9481973 9532808 9533049 9533366 9548130 9564383 9570081 9590426 9609953 9609954 9609957 9612937 9616048 9933813 10036499 10093315 10219627 10334634
832 833 834 837 843 849 854 856 857 858 861 866 872 873 874 876 877 878 881 882 883 884 885 887 895 900 905 916 922

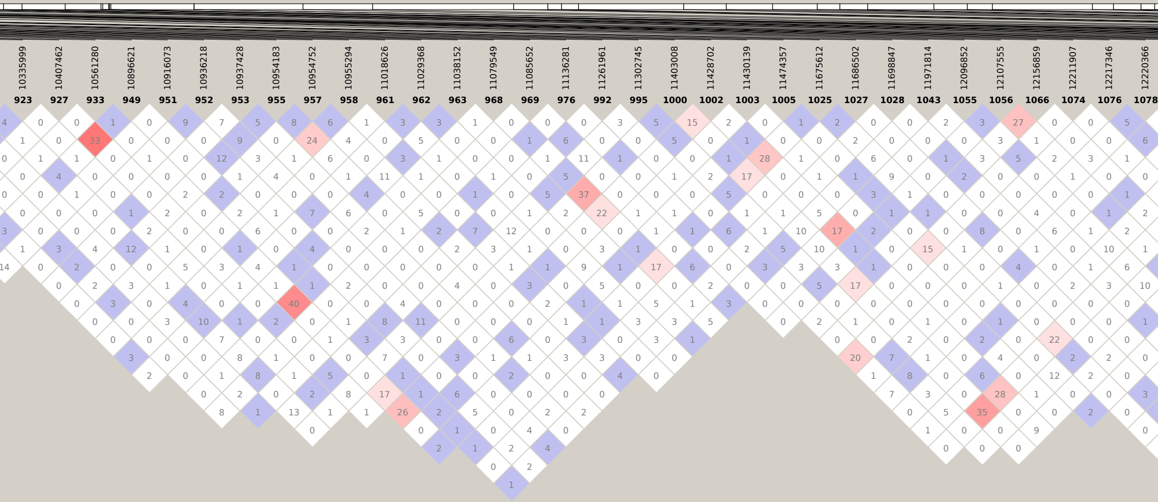

10335999
10407462
10561280
10896621
10916073
10936218
10937428
10954183
10954752
10955294
11018626
11029368
11038152
11079549
11085652
11136281
11261961
11302745
11403008
11428702
11430139
11474357
11675612
11686502
11698847
11971814
12096852
12107555
12156859
12211907
12217346
12220366
923
927
933
949
951
952
953
955
957
958
961
962
963
968
969
976
992
995
1000
1002
1003
1005
1025
1027
1028
1043
1055
1056
1066
1074
1076
1078

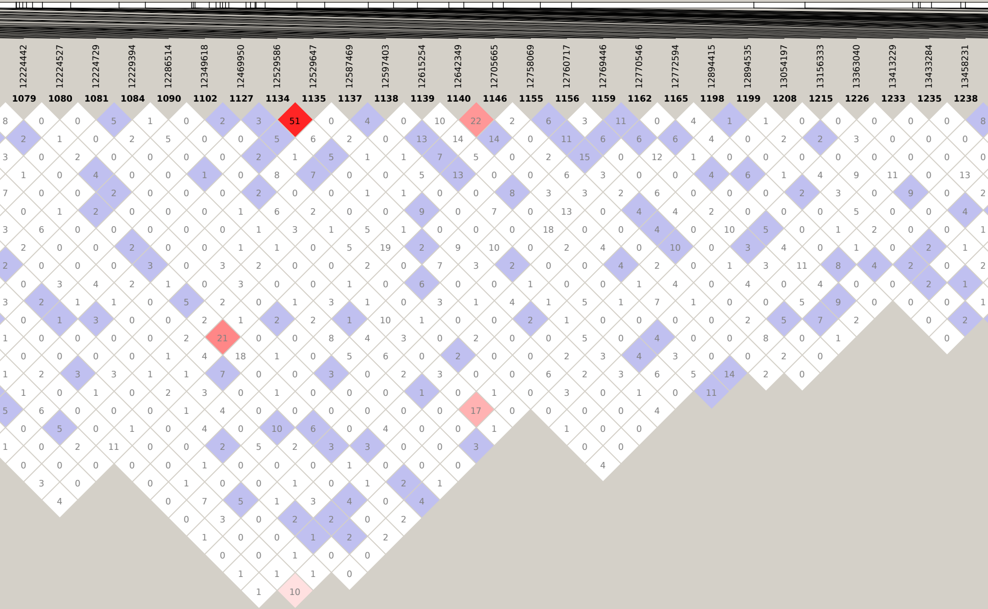
12224442
12224527
12224729
12229394
12286514
12349618
12469950
12529586
12529647
12587469
12597403
12615254
12642349
12705665
12758069
12760717
12769446
12770546
12772594
12894415
12894535
13054197
13156333
13363040
13413229
13433284
13458231
1079
1080
1081
1084
1090
1102
1127
1134
1135
1137
1138
1139
1140
1146
1155
1156
1159
1162
1165
1198
1199
1208
1215
1226
1233
1235
1238

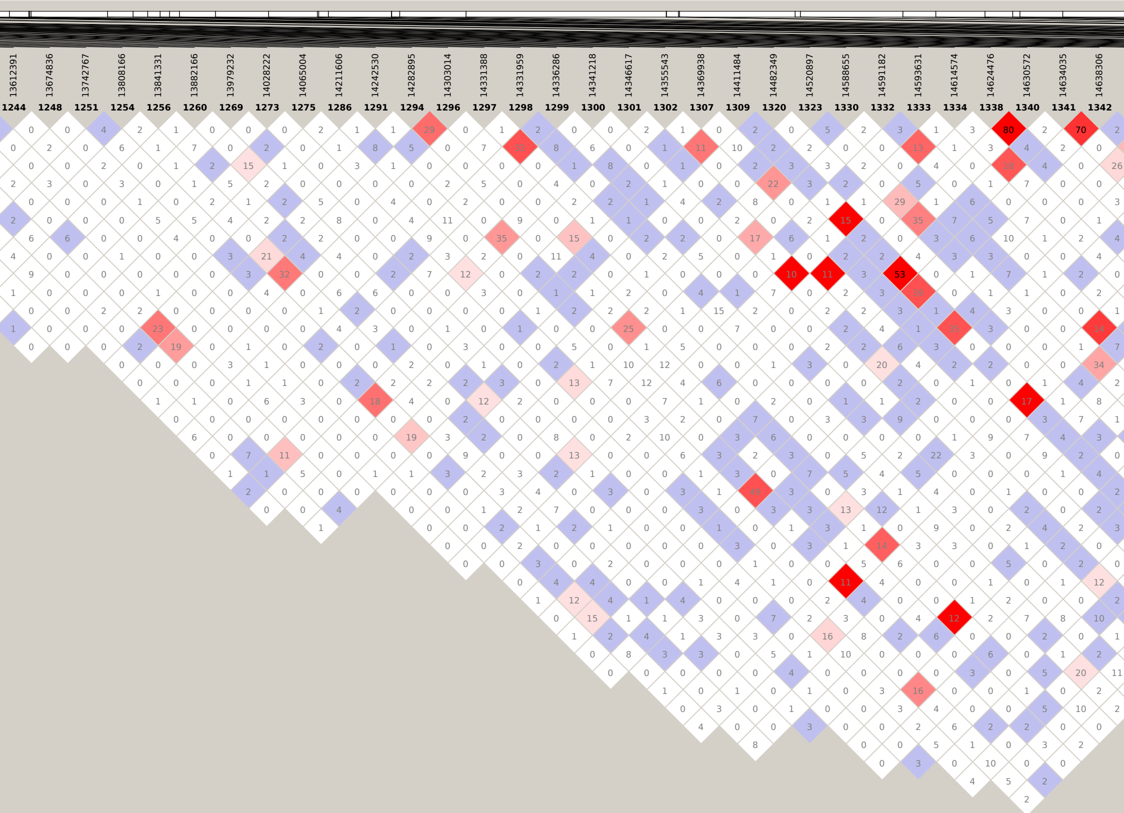
13612391
13674836
13742767
13808166
13841331
13882166
13979232
14028222
14065004
14211606
14242530
14282895
14303014
14331388
14331959
14336286
14341218
14346617
14355543
14369938
14411484
14482349
14520897
14588655
14591182
14593631
14614574
14624476
14630572
14634035
14638306
1244
1248
1251
1254
1256
1260
1269
1273
1275
1286
1291
1294
1296
1297
1298
1299
1300
1301
1302
1307
1309
1320
1323
1330
1332
1333
1334
1338
1340
1341
1342

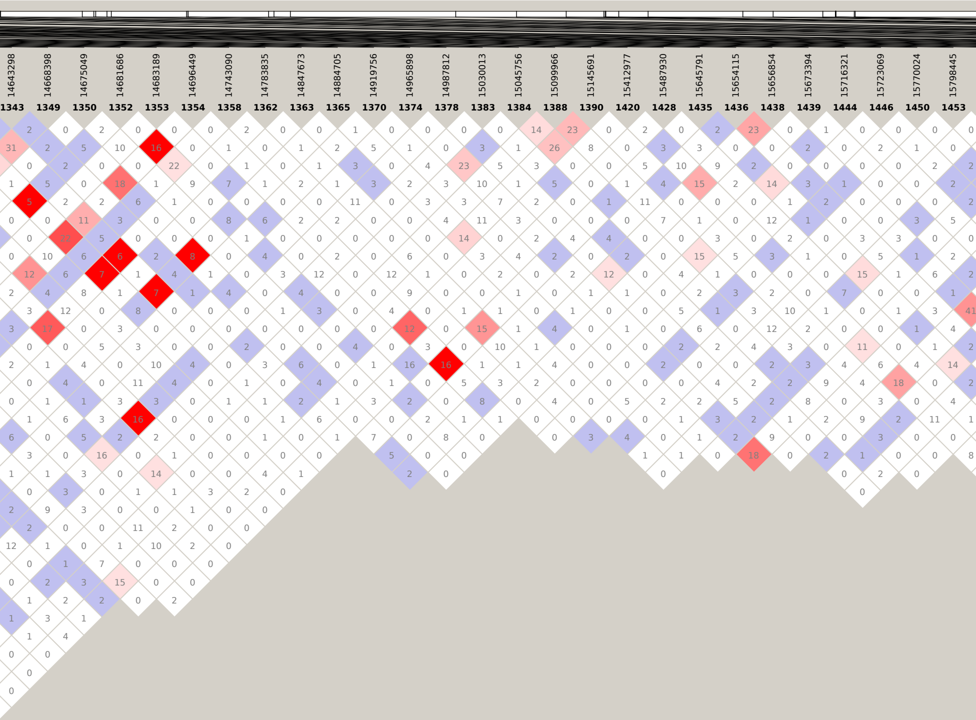
14643298
14668398
14675049
14681686
14683189
14696449
14743090
14783835
14847673
14884705
14919756
14965898
14987812
15030013
15045756
15099966
15145691
15412977
15487930
15645791
15654115
15656854
15673394
15716321
15723069
15770024
15798445
1343
1349
1350
1352
1353
1354
1358
1362
1363
1365
1370
1374
1378
1383
1384
1388
1390
1420
1428
1435
1436
1438
1439
1444
1446
1450
1453

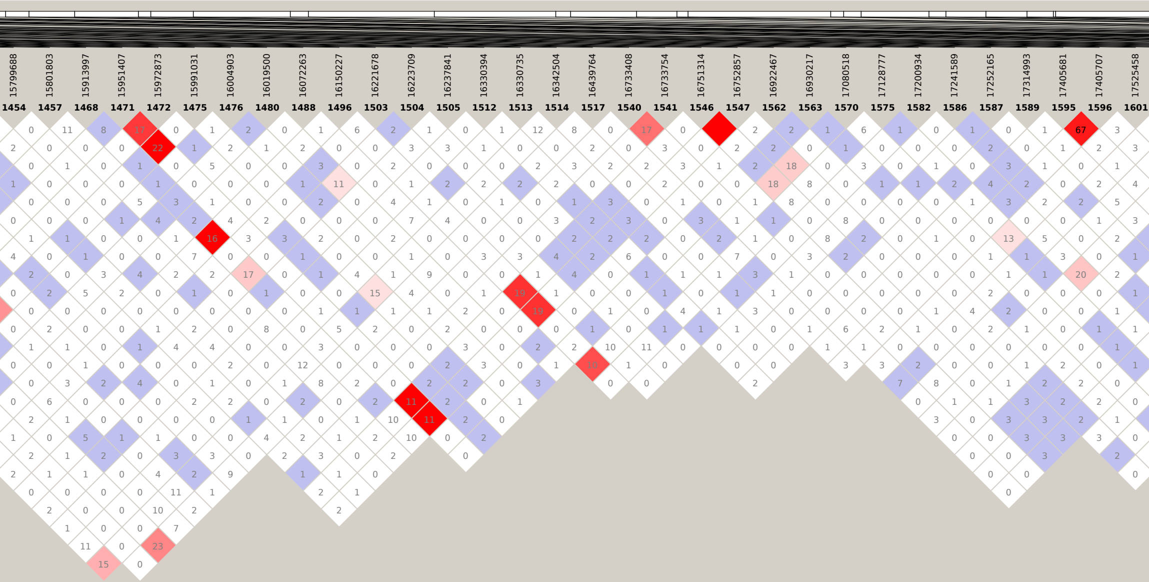
15799688 15801803 15913997 15951407 15972873 15991031 16004903 16019500 16072263 16150227 16221678 16223709 16237841 16330394 16330735 16342504 16439764 16733408 16733754 16751314 16752857 16922467 16930217 17080518 17128777 17200934 17241589 17252165 17314993 17405681 17405707 17525458
1454 1457 1468 1471 1472 1475 1476 1480 1488 1496 1503 1504 1505 1512 1513 1514 1517 1540 1541 1546 1547 1562 1563 1570 1575 1582 1586 1587 1589 1595 1596 1601

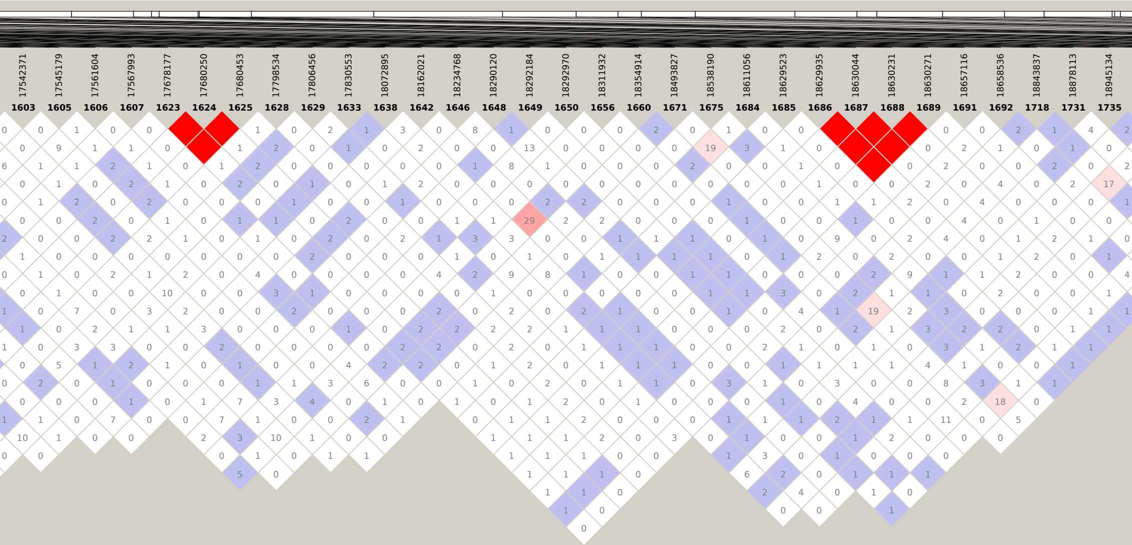

17542371 17545179 17561604 17567993 17678177 17680250 17680453 17798534 17806456 17830553 18072895 18162021 18234768 18290120 18292184 18292970 18311932 18354914 18493827 18538190 18611056 18629523 18629935 18630044 18630231 18630271 18657116 18658536 18843837 18878113 18945134
1603 1605 1606 1607 1623 1624 1625 1628 1629 1633 1638 1642 1646 1648 1649 1650 1656 1660 1671 1675 1684 1685 1686 1687 1688 1689 1691 1692 1718 1731 1735

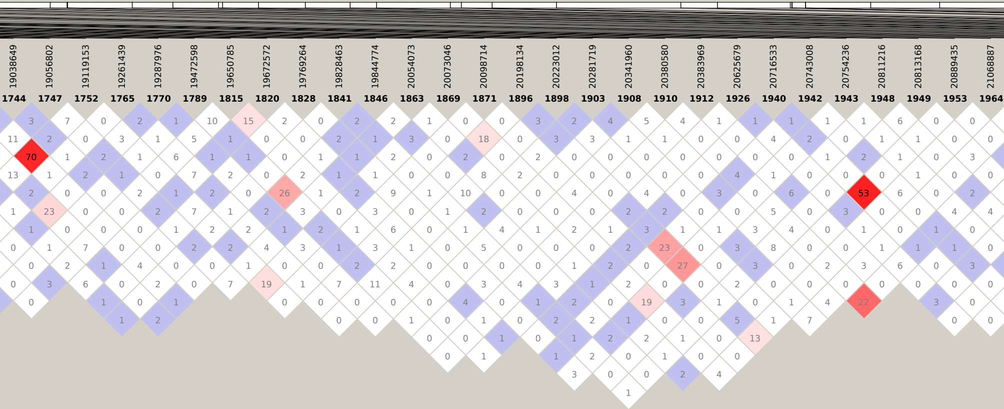
19038649
19056802
19119153
19261439
19287976
19472598
19650785
19672572
19769264
19828463
19844774
20054073
20073046
20098714
20198134
20223012
20281719
20341960
20380580
20383969
20625679
20716533
20743008
20754236
20811216
20813168
20889435
21068887
1744
1747
1752
1765
1770
1789
1815
1820
1828
1841
1846
1863
1869
1871
1896
1898
1903
1908
1910
1912
1926
1940
1942
1943
1948
1949
1953
1964

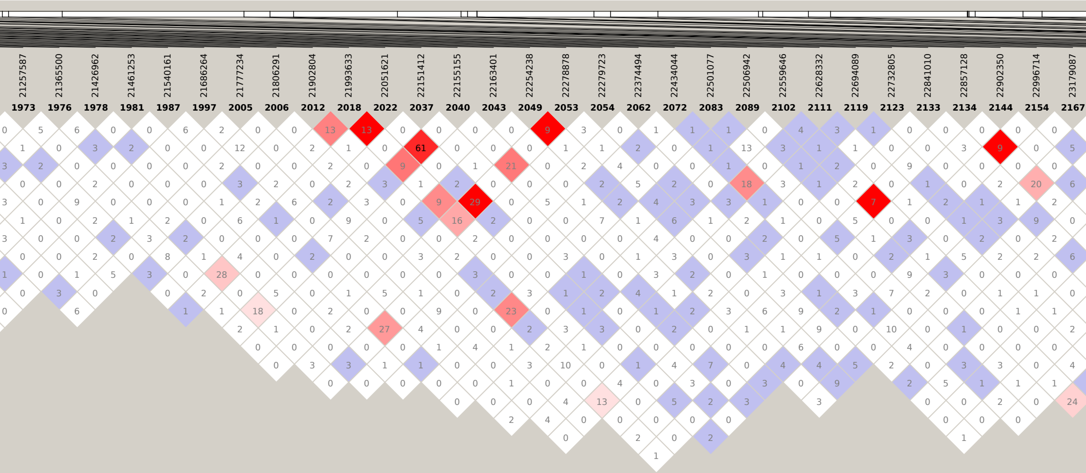
21257587 21365500 21426962 21461253 21540161 21686264 21777234 21806291 21902804 21993633 22051621 22151412 22155155 22163401 22254238 22278878 22279723 22374494 22434044 22501077 22506942 22559646 22628332 22694089 22732805 22841010 22857128 22902350 22996714 23179087
1973 1976 1978 1981 1987 1997 2005 2006 2012 2018 2022 2037 2040 2043 2049 2053 2054 2062 2072 2083 2089 2102 2111 2119 2123 2133 2134 2144 2154 2167

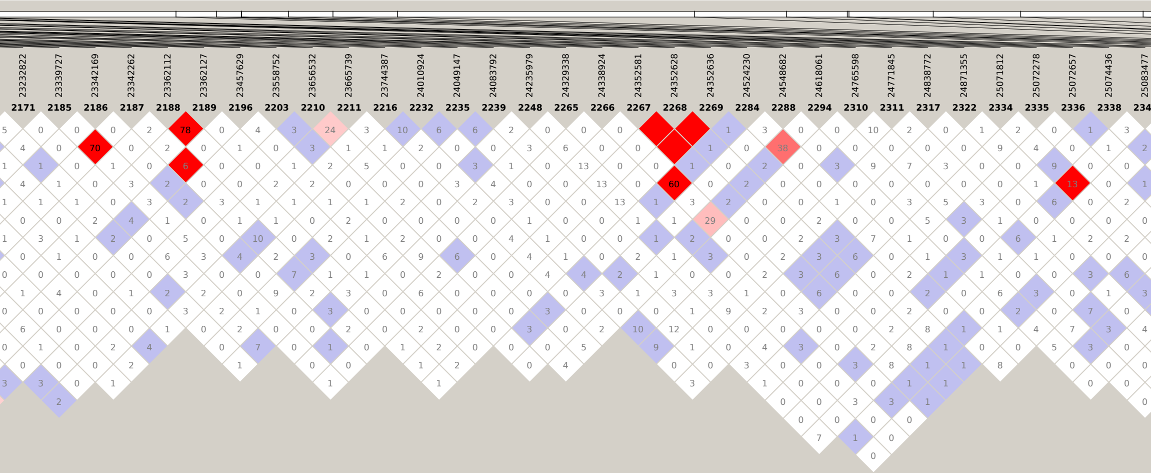
23232822 23339727 23342169 23342262 23362112 23362127 23457629 23558752 23656532 23665739 23744387 24010924 24049147 24083792 24235979 24329338 24338924 24352581 24352628 24352636 24524230 24548682 24618061 24765598 24771845 24838772 24871355 25071812 25072278 25072657 25074436 25083477
2171 2185 2186 2187 2188 2189 2196 2203 2210 2211 2216 2232 2235 2239 2248 2265 2266 2267 2268 2269 2284 2288 2294 2310 2311 2317 2322 2334 2335 2336 2338

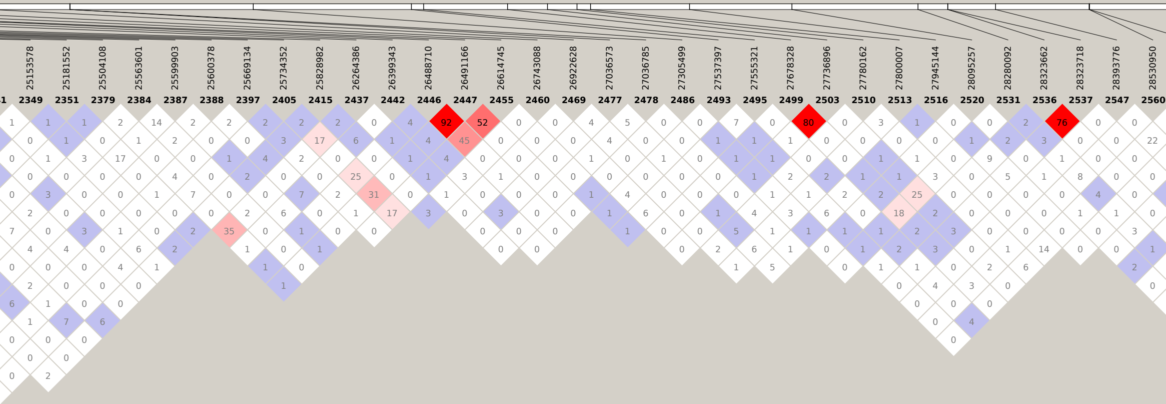

25153578
25181552
25504108
25563601
25599903
25600378
25669134
25734352
25828982
26264386
26399343
26488710
26491166
26614745
26743088
26922628
27036573
27036785
27305499
27537397
27555321
27678328
27736896
27780162
27800007
27945144
28095257
28280092
28323662
28323718
28393776
28530950
2349
2351
2379
2384
2387
2388
2397
2405
2415
2437
2442
2446
2447
2455
2460
2469
2477
2478
2486
2493
2495
2499
2503
2510
2513
2516
2520
2531
2536
2537
2547
2560

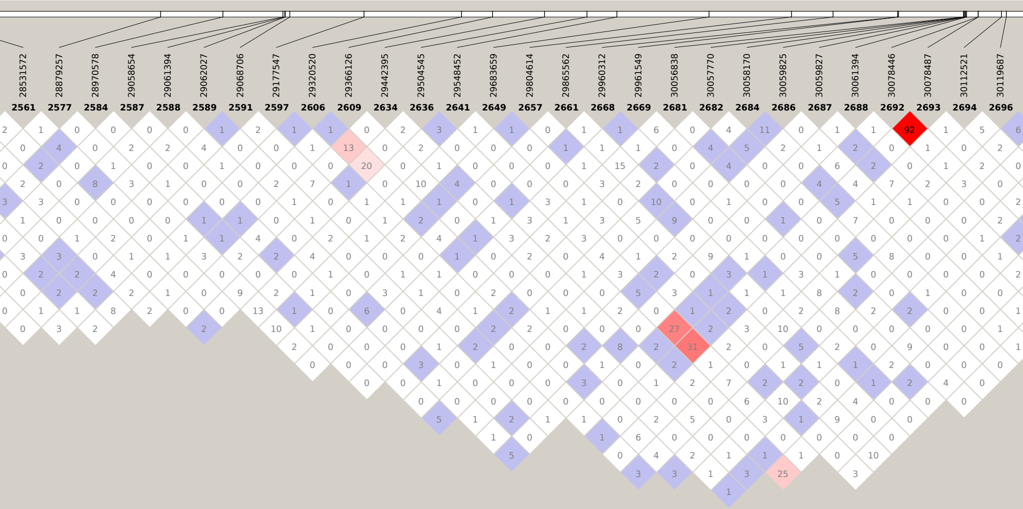
28531572 28879257 28970578 29058654 29061394 29062027 29068706 29177547 29320520 29366126 29442395 29504545 29548452 29683659 29804614 29865562 29960312 29961549 30056838 30057770 30058170 30059825 30059827 30061394 30078446 30078487 30112521 30119687
2561 2577 2584 2587 2588 2589 2591 2597 2606 2609 2634 2636 2641 2649 2657 2661 2668 2669 2681 2682 2684 2686 2687 2688 2692 2693 2694 2696

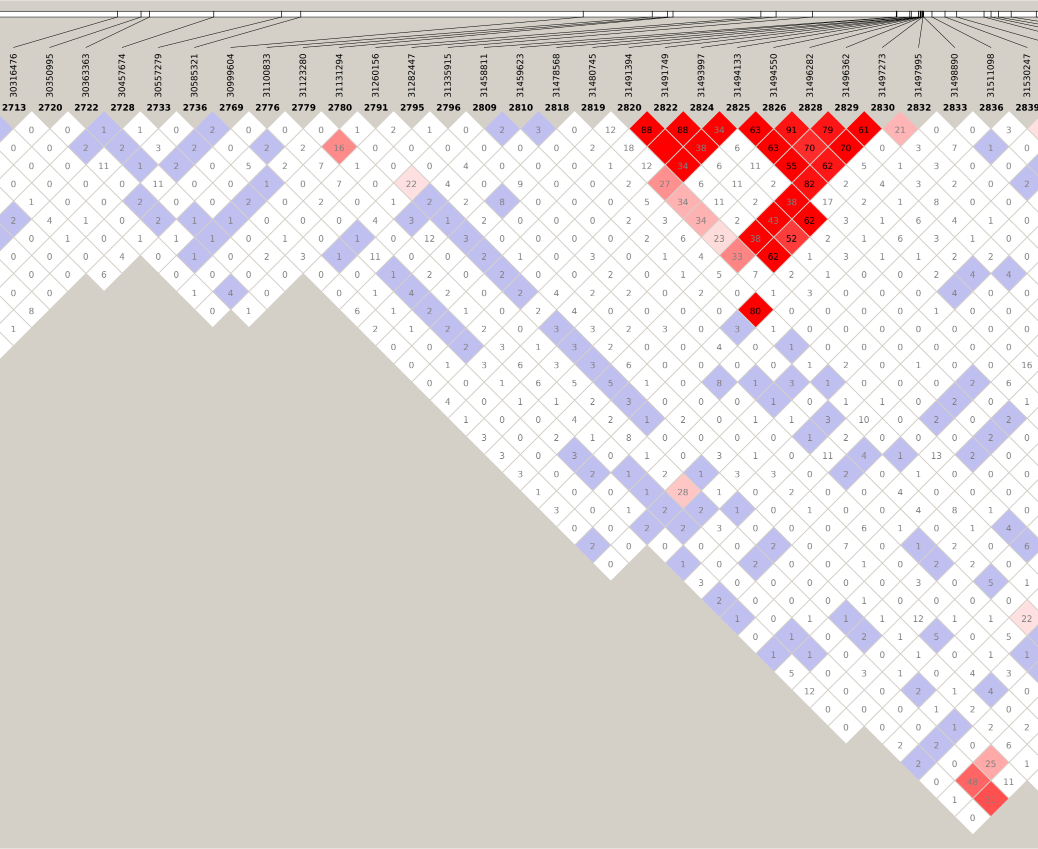
30316476
30350995
30363363
30457674
30557279
30585321
30999604
31100833
31123280
31131294
31260156
31282447
31335915
31458811
31459623
31478568
31480745
31491394
31491749
31493997
31494133
31494550
31496282
31496362
31497273
31497995
31498890
31511098
31530247
2713
2720
2722
2728
2733
2736
2769
2776
2779
2780
2791
2795
2796
2809
2810
2818
2819
2820
2822
2824
2825
2826
2828
2829
2830
2832
2833
2836
2839

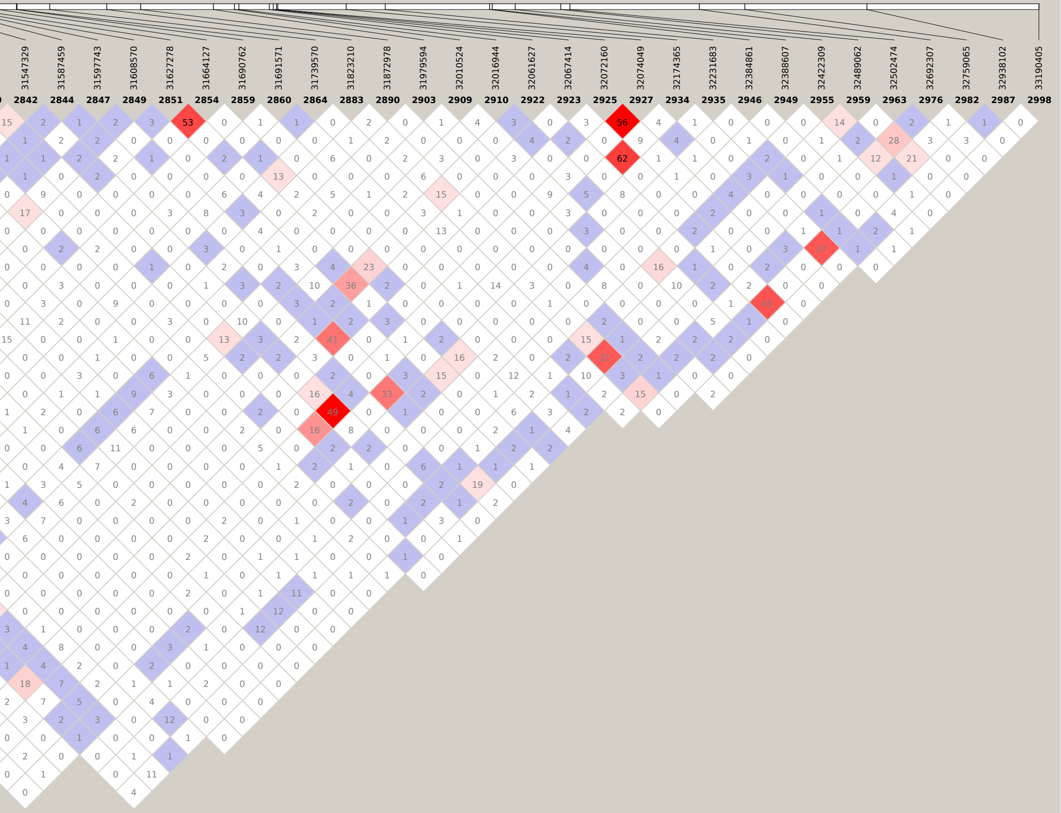

31547329
31587459
31597743
31608570
31627278
31664127
31690762
31691571
31739570
31823210
31872978
31979594
32010524
32016944
32061627
32067414
32072160
32074049
32174365
32231683
32384861
32388607
32422309
32489062
32502474
32692307
32759065
32938102
33190405
2842
2844
2847
2849
2851
2854
2859
2860
2864
2883
2890
2903
2909
2910
2922
2923
2925
2927
2934
2935
2946
2949
2955
2959
2963
2976
2982
2987
2998

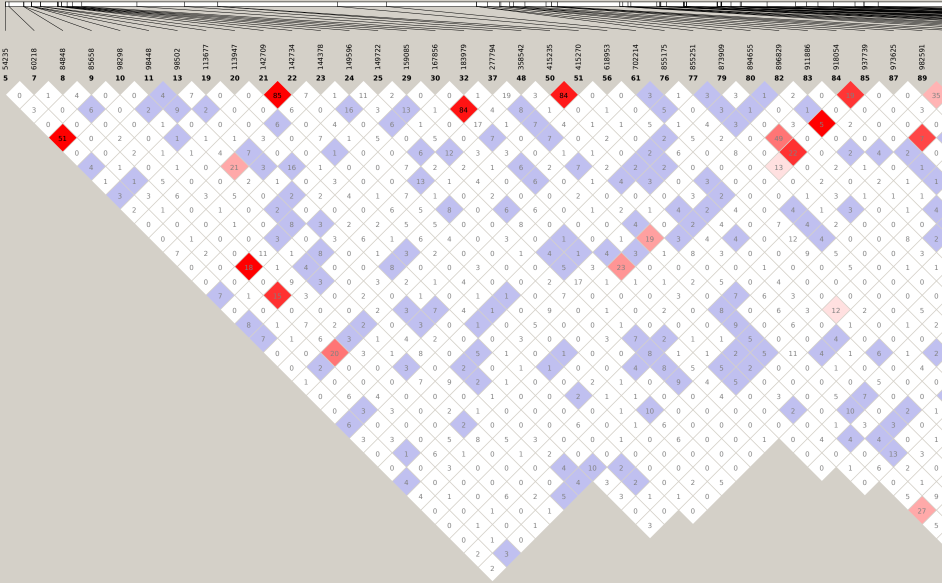

54235 60218 84848 85658 98298 98448 98502 113677 113947 142709 142734 144378 149596 149722 159085 167856 183979 277794 358542 415235 415270 618953 702214 855175 855251 873909 894655 896829 911886 918054 937739 973625 982591
5 7 8 9 10 11 13 19 20 21 22 23 24 25 29 30 32 37 48 50 51 56 61 76 77 79 80 82 83 84 85 87 89

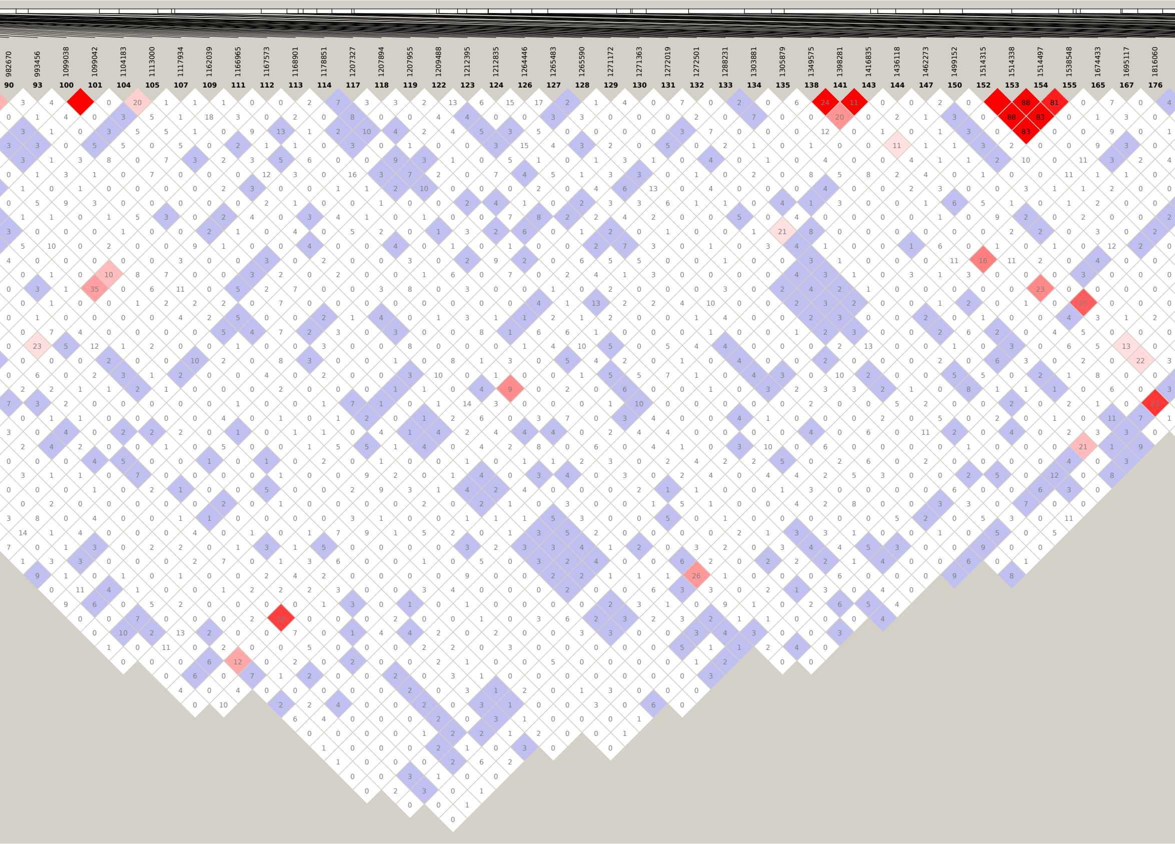

982670 993456 1099038 1099042 1104183 1113000 1117934 1162039 1166965 1167573 1168901 1178851 1207327 1207894 1207955 1209488 1212395 1212835 1264446 1265483 1265590 1271172 1271363 1272019 1272501 1288231 1303881 1305879 1349575 1398281 1416835 1436118 1462273 1499152 1514315 1514338 1514497 1538548 1674433 1695117 1816060
90 93 100 101 104 105 107 109 111 112 113 114 117 118 119 122 123 124 126 127 128 129 130 131 132 133 134 135 138 141 143 144 147 150 152 153 154 155 165 167 176

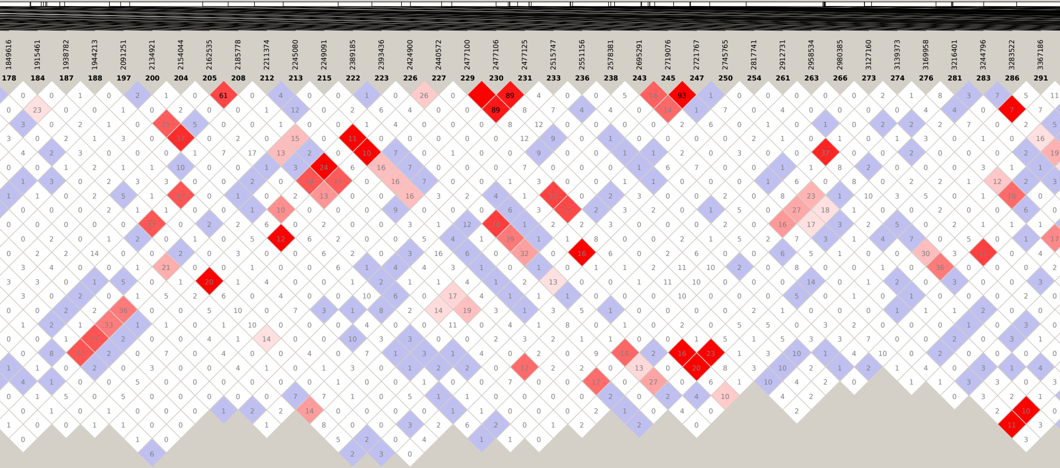

1849616
178
1915461
184
1938782
187
1944213
188
2091251
197
2134921
200
2154044
204
2162535
205
2185778
208
2211374
212
2245080
213
2249091
215
2389185
222
2393436
223
2424900
226
2440572
227
2477100
229
2477106
230
2477125
231
2515747
233
2551156
236
2578381
238
2695291
243
2719076
245
2721767
247
2745765
250
2817741
254
2912731
261
2958534
263
2980385
266
3127160
273
3139373
274
3169958
276
3216401
281
3244796
283
3283522
286
3367186
291

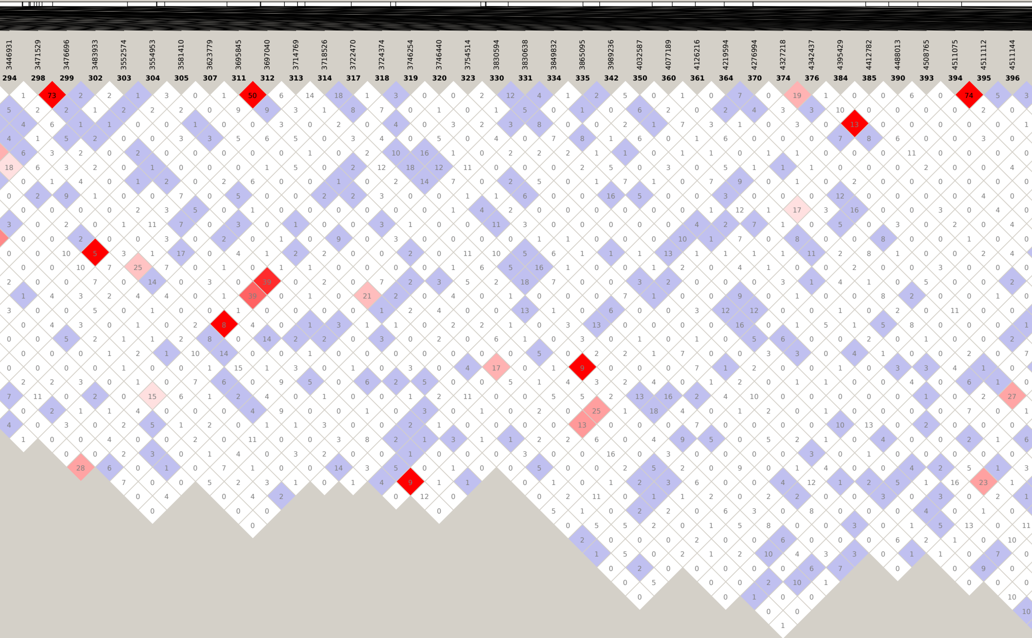
3446931
294
3471529
298
3476696
299
3483933
302
3552574
303
3554953
304
3581410
305
3623779
307
3695845
311
3697040
312
3714769
313
3718526
314
3722470
317
3724374
318
3746254
319
3746440
320
3754514
323
3830594
330
3830638
331
3849832
334
3865095
335
3989236
342
4032587
350
4077189
360
4126216
361
4219594
364
4276994
370
4327218
374
4342437
376
4395429
384
4412782
385
4488013
390
4508765
393
4511075
394
4511112
395
4511144
396

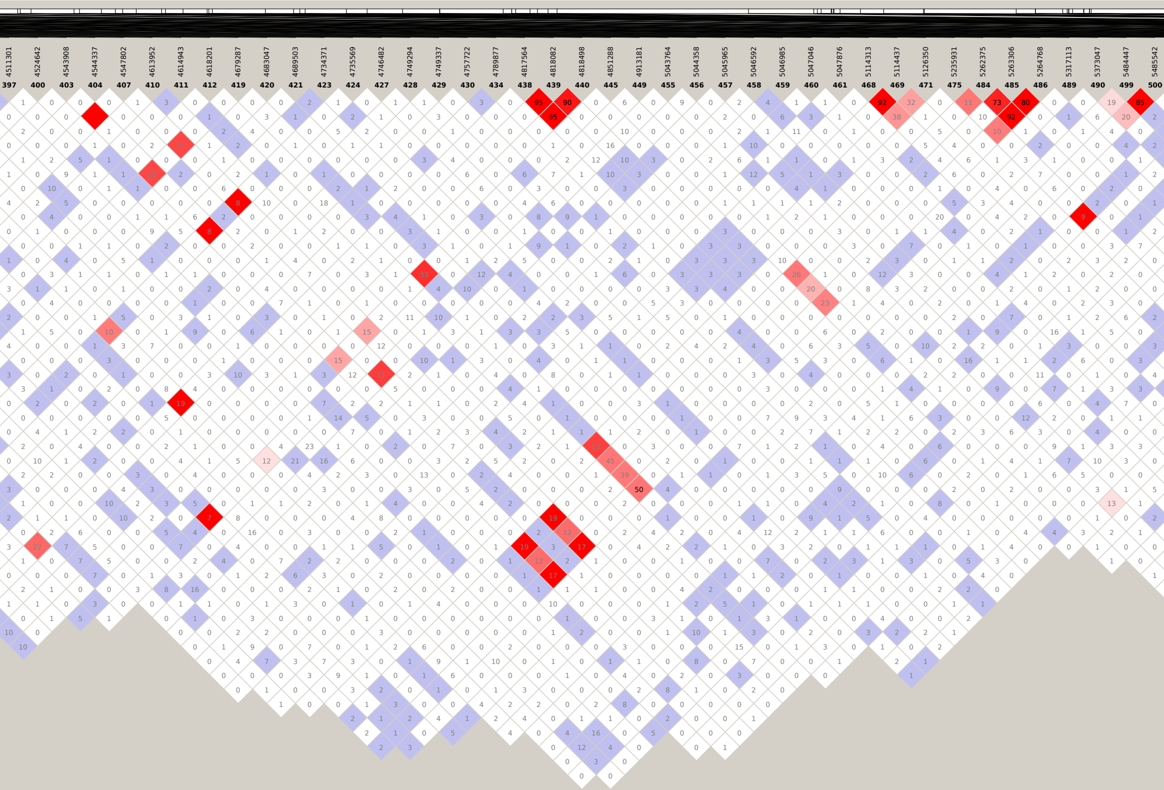
4511301
397
4524642
400
4543908
403
4544337
404
4547802
407
4613952
410
4614943
411
4618201
412
4679287
419
4683047
420
4689503
421
4734371
423
4735569
424
4746482
427
4749294
428
4749337
429
4757722
430
4789877
434
4817564
438
4818082
439
4818498
440
4851288
445
4913181
449
5043764
455
5044358
456
5045965
457
5046592
458
5046985
459
5047046
460
5047876
461
5114313
468
5114437
469
5126350
471
5235931
475
5262375
484
5263306
485
5264768
486
5317113
489
5373047
490
5484447
499
5485542
500

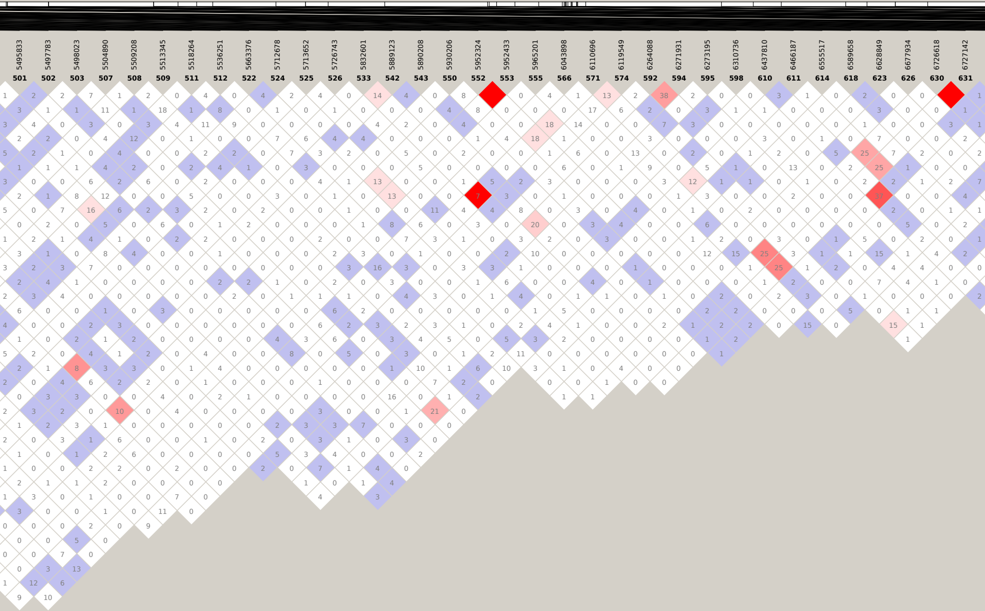

5495833 5497783 5498023 5504890 5509208 5513345 5518264 5536251 5663376 5712678 5713652 5726743 5832601 5889123 5890208 5930206 5952324 5952433 5965201 6043898 6110696 6119549 6264088 6271931 6273195 6310736 6437810 6466187 6555517 6589658 6628849 6677934 6726618 6727142
501 502 503 507 508 509 511 512 522 524 525 526 533 542 543 550 552 553 555 566 571 574 592 594 595 598 610 611 614 618 623 626 630 631

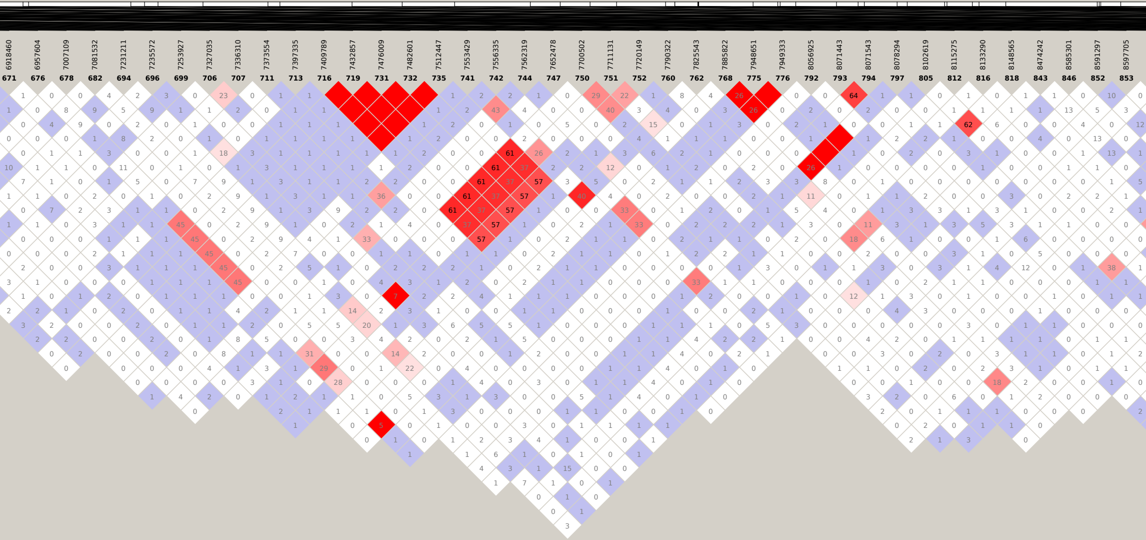
6918460
6957604
7007109
7081532
7231211
7235572
7253927
7327035
7336310
7373554
7397335
7409789
7432857
7476009
7482601
7512447
7553429
7556335
7562319
7652478
7700502
7711131
7720149
7790322
7825543
7885822
7948651
7949333
8056925
8071443
8071543
8078294
8102619
8115275
8133290
8148565
8474242
8585301
8591297
8597705
671 676 678 682 694 696 699 706 707 711 713 716 719 731 732 735 741 742 744 747 750 751 752 760 762 768 775 776 792 793 794 797 805 812 816 818 843 846 852 853

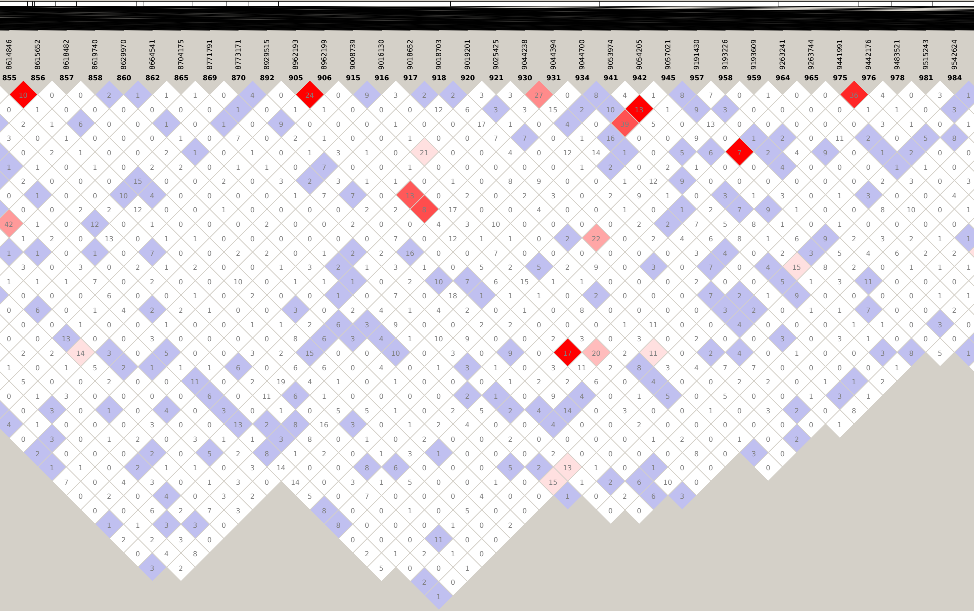

8614846 8615652 8618482 8619740 8629970 8664541 8704175 8771791 8773171 8929515 8962193 8962199 9008739 9016130 9018652 9018703 9019201 9025425 9044238 9044394 9044700 9053974 9054205 9057021 9191430 9193226 9193609 9263241 9263744 9441991 9442176 9483521 9515243 9542624
855 856 857 858 860 862 865 869 870 892 905 906 915 916 917 918 920 921 930 931 934 941 942 945 957 958 959 964 965 975 976 978 981 984

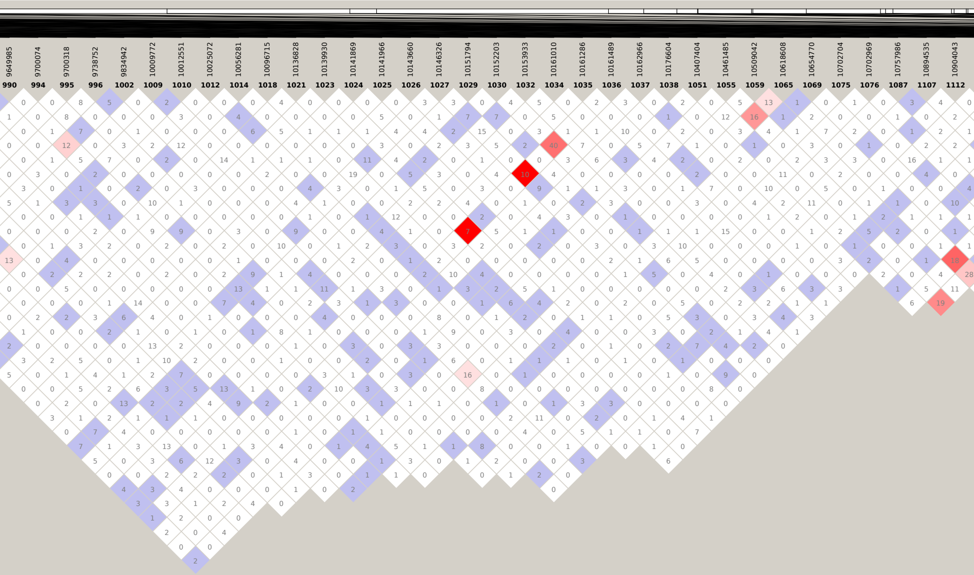

9649985
990
9700074
994
9700318
995
9738752
996
9834942
1002
10009772
1009
10012551
1010
10025072
1012
10056281
1014
10096715
1018
10136828
1021
10139930
1023
10141869
1024
10141966
1025
10143660
1026
10146326
1027
10151794
1029
10152203
1030
10153933
1032
10161010
1034
10161286
1035
10161489
1036
10162966
1037
10176604
1038
10407404
1051
10461485
1055
10509042
1059
10618608
1065
10654770
1069
10702704
1075
10702969
1076
10757986
1087
10894535
1107
10904043
1112

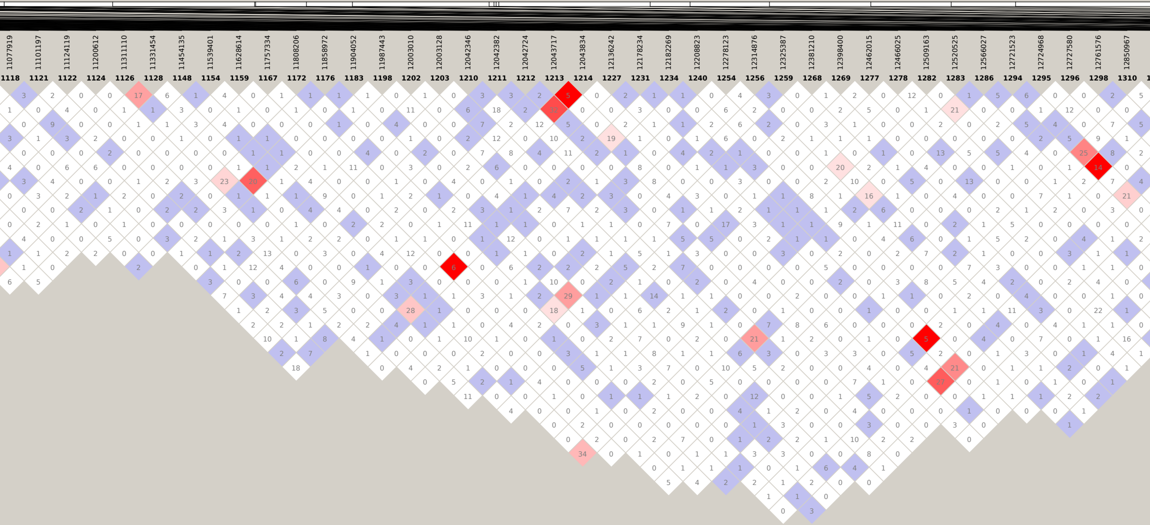
11077919
11101197
11124119
11200612
11311110
11331454
11454135
11539401
11628614
11757334
11808206
11858972
11904052
11987443
12003010
12003128
12042346
12042382
12042724
12043717
12043834
12136242
12178234
12182269
12208823
12278123
12314876
12325387
12381210
12398400
12462015
12466025
12509163
12520525
12566027
12721523
12724968
12727580
12761576
12850967
1118
1121
1122
1124
1126
1128
1148
1154
1159
1167
1172
1176
1183
1198
1202
1203
1210
1211
1212
1213
1214
1227
1231
1234
1240
1254
1256
1259
1268
1269
1277
1278
1282
1283
1286
1294
1295
1296
1298
1310

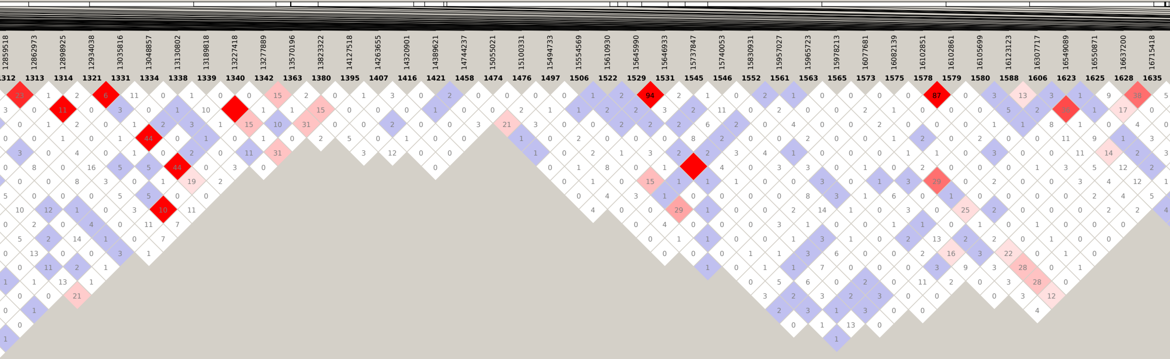
12859518
12862973
12898925
12934038
13035816
13048857
13130802
13189818
13227418
13277889
13570196
13823322
14127518
14263655
14320901
14389621
14744237
15055021
15100331
15494733
15554569
15610930
15645990
15646933
15737847
15740053
15830931
15957027
15965723
15978213
16077681
16082139
16102851
16102861
16105699
16123123
16307717
16549089
16550871
16637200
16715418
312
1313
1314
1321
1331
1334
1338
1339
1340
1342
1363
1380
1395
1407
1416
1421
1458
1474
1476
1497
1506
1522
1529
1531
1545
1546
1552
1561
1563
1565
1573
1575
1578
1579
1580
1588
1606
1623
1625
1628
1635

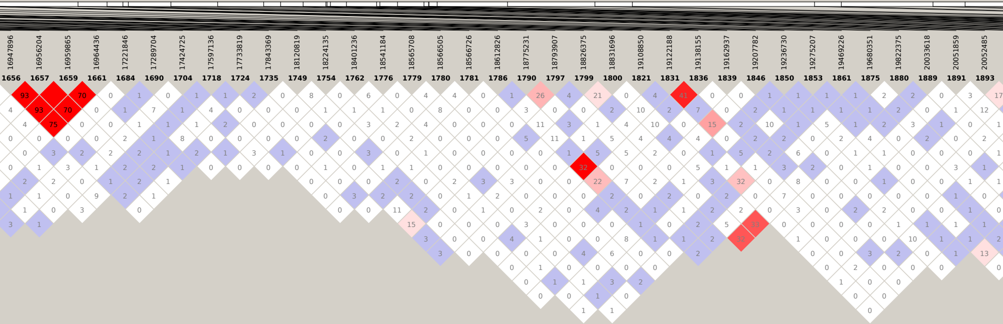
16947896
16956204
16959865
16964436
17221846
17289704
17424725
17597136
17733819
17843369
18120819
18224135
18401236
18541184
18565708
18566505
18566726
18612826
18775231
18793907
18826375
18831696
19108850
19122188
19138155
19162937
19207782
19236730
19275207
19469226
19680351
19822375
20033618
20051859
20052485
1656
1657
1659
1661
1684
1690
1704
1718
1724
1735
1749
1754
1762
1776
1779
1780
1781
1786
1790
1797
1799
1800
1821
1831
1836
1839
1846
1850
1853
1861
1875
1880
1889
1891
1893

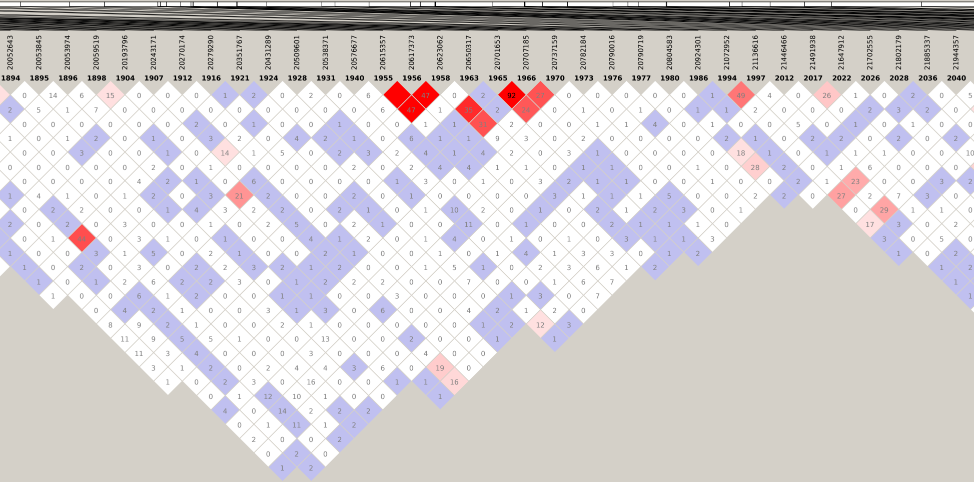
20052643 20053845 20053974 20059519 20193796 20243171 20270174 20279290 20351767 20431289 20509601 20538371 20576677 20615357 20617373 20623062 20650317 20701653 20707185 20737159 20782184 20790016 20790719 20804583 20924301 21072952 21136616 21446466 21491938 21647912 21702555 21802179 21885337 21944357
1894 1895 1896 1898 1904 1907 1912 1916 1921 1924 1928 1931 1940 1955 1956 1958 1963 1965 1966 1970 1973 1976 1977 1980 1986 1994 1997 2012 2017 2022 2026 2028 2036 2040

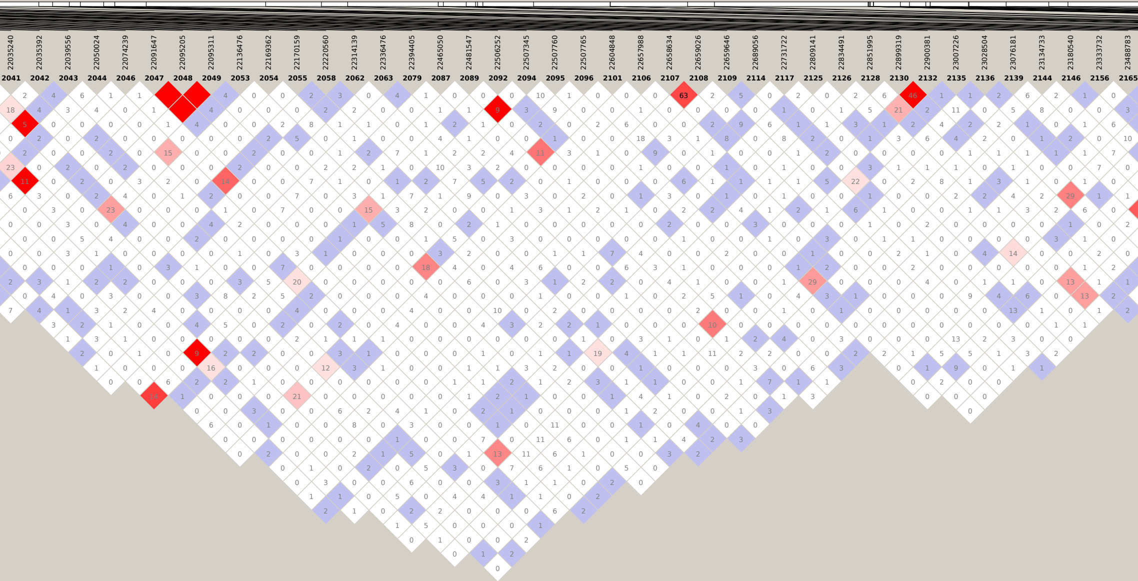
22035240 22035392 22039556 22050024 22074239 22091647 22095205 22095311 22136476 22169362 22170159 22220560 22314139 22336476 22394405 22465050 22481547 22506252 22507345 22507760 22507765 22604848 22657988 22658634 22659026 22659646 22689056 22731722 22809141 22834491 22851995 22899319 22900381 23007226 23028504 23076181 23134733 23180540 23333732 23488783
2041 2042 2043 2044 2046 2047 2048 2049 2053 2054 2055 2058 2062 2063 2079 2087 2089 2092 2094 2095 2096 2101 2106 2107 2108 2109 2114 2117 2125 2126 2128 2130 2132 2135 2136 2139 2144 2146 2156 2165

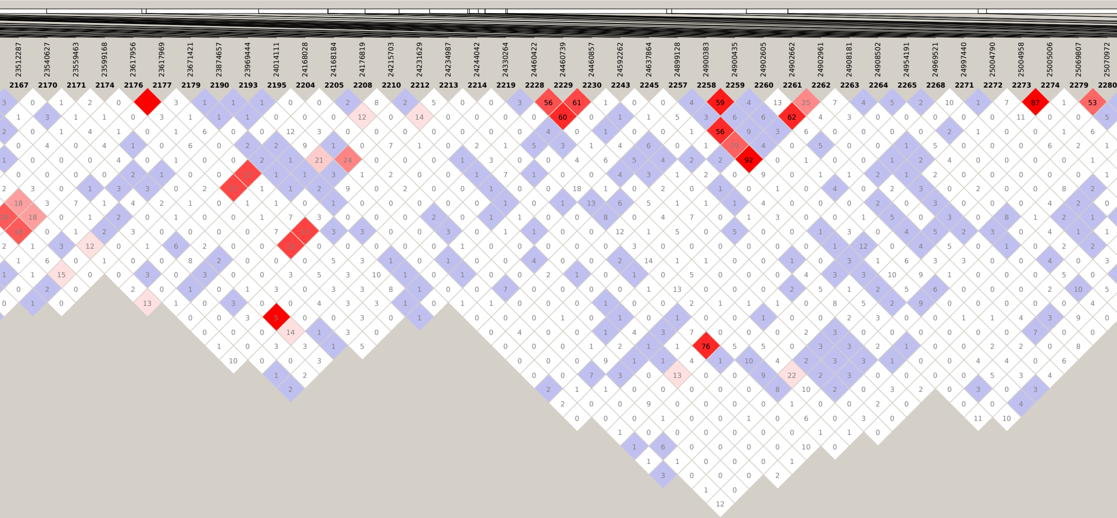
23512287 23540627 23559463 23599168 23617956 23617969 23671421 23874657 23969444 24014111 24168028 24168184 24176819 24215703 24231629 24234987 24244042 24330264 24460422 24460739 24460857 24592262 24637864 24899128 24900383 24900435 24902605 24902662 24902961 24908181 24908502 24954191 24969521 24997440 25004790 25004958 25005006 25069807 25070972
2167 2170 2171 2174 2176 2177 2179 2190 2193 2195 2204 2205 2208 2210 2212 2213 2214 2219 2228 2229 2230 2243 2245 2257 2258 2259 2260 2261 2262 2263 2264 2265 2268 2271 2272 2273 2274 2279 2280

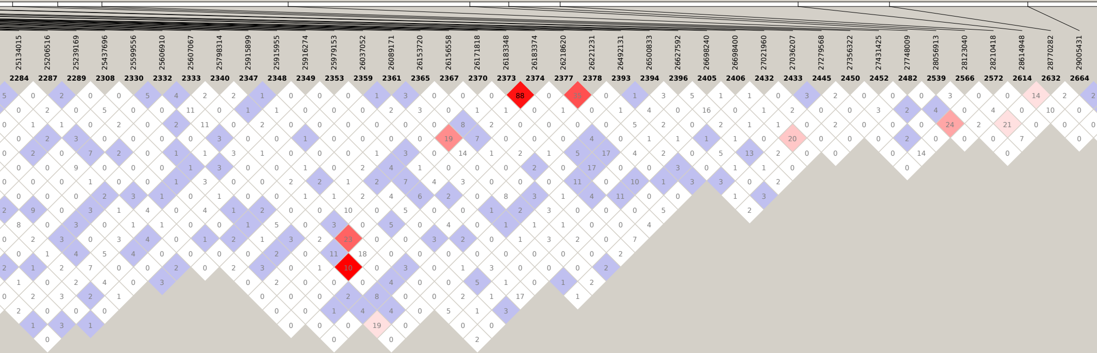

25134015
25206516
25239169
25437696
25599556
25606910
25607067
25798314
25915899
25915955
25916274
25979153
26037052
26089171
26153720
26156558
26171818
26183348
26183374
26218620
26221231
26492131
26500833
26627592
26698240
26698400
27021960
27036207
27279568
27356322
27431425
27748009
28056913
28123040
28210418
28614948
28770282
29005431
2284
2287
2289
2308
2330
2332
2333
2340
2347
2348
2349
2353
2359
2361
2365
2367
2370
2373
2374
2377
2378
2393
2394
2396
2405
2406
2432
2433
2445
2450
2452
2482
2539
2566
2572
2614
2632
2664

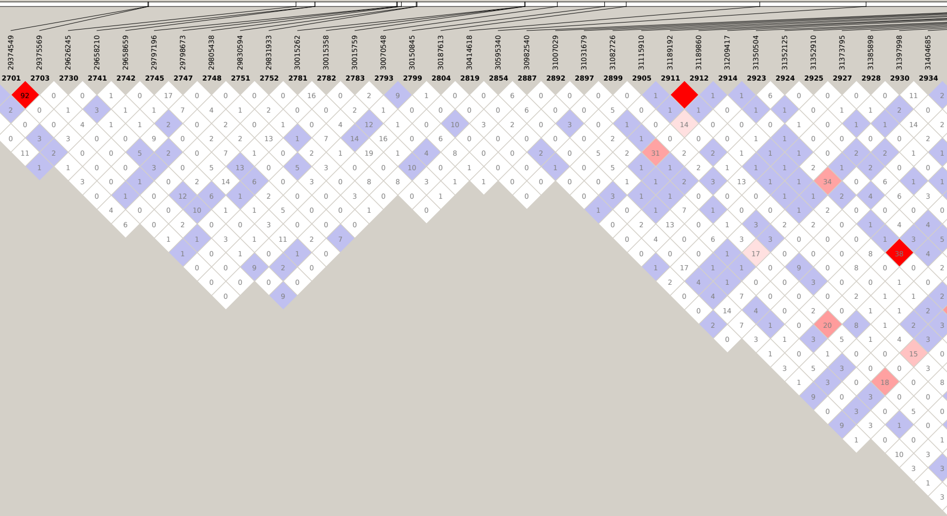
29374549
29375569
29626245
29658210
29658659
29797196
29798673
29805438
29830594
29831933
30015262
30015358
30015759
30070548
30150845
30187613
30414618
30595340
30982540
31007029
31031679
31082726
31115910
31189192
31189860
31209417
31350504
31352125
31352910
31373795
31385898
31397998
31404685
2701 2703 2730 2741 2742 2745 2747 2748 2751 2752 2781 2782 2783 2793 2799 2804 2819 2854 2887 2892 2897 2899 2905 2911 2912 2914 2923 2924 2925 2927 2928 2930 2934

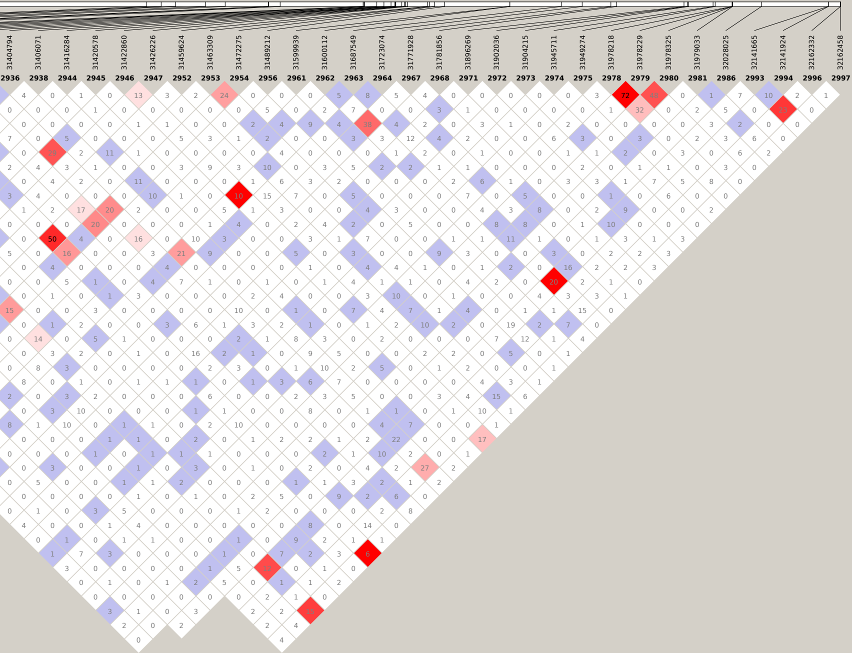
31404794 31406071 31416284 31420578 31422860 31426226 31459624 31463309 31472275 31489212 31599939 31600112 31687549 31723074 31771928 31781856 31896269 31902036 31904215 31945711 31949274 31978218 31978229 31978325 31979033 32028025 32141665 32141924 32162332 32162458
2936 2938 2944 2945 2946 2947 2952 2953 2954 2956 2961 2962 2963 2964 2967 2968 2971 2972 2973 2974 2975 2978 2979 2980 2981 2986 2993 2994 2996 2997

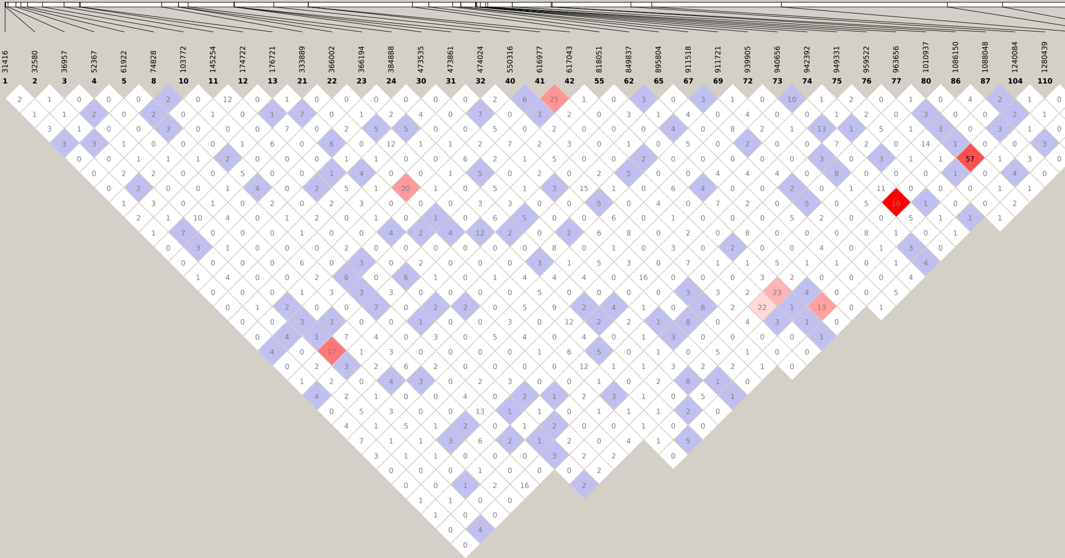

31416 1
32580 2
36957 3
52367 4
61922 5
74828 8
103772 10
145254 11
174722 12
176721 13
333889 21
366002 22
366194 23
384888 24
473535 30
473861 31
474024 32
550316 40
616977 41
617043 42
818051 55
849837 62
895804 65
911518 67
911721 69
939905 72
940656 73
942392 74
949331 75
959522 76
963656 77
1010937 80
1086150 86
1088048 87
1240084 104
1280439 110

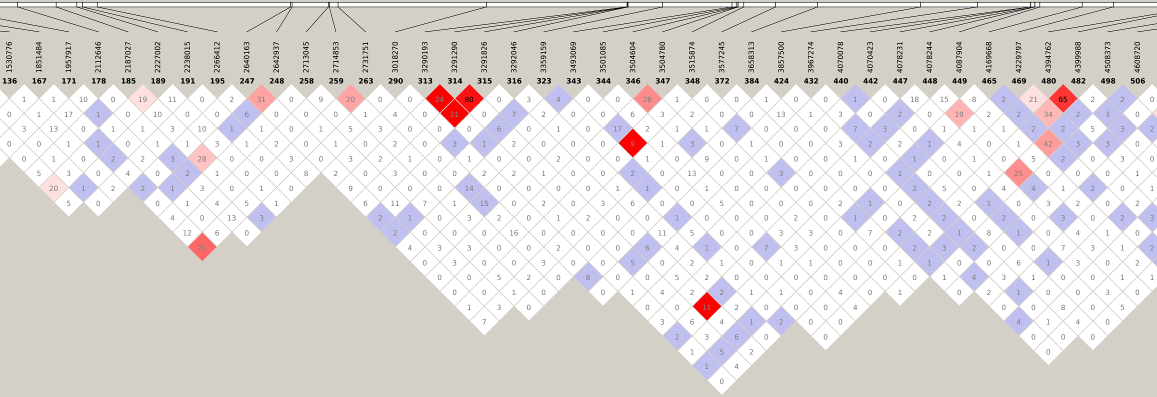
1530776
1851484
1957917
2112646
2187027
2227002
2238015
2266412
2640163
2642937
2713045
2714853
2731751
3018270
3290193
3291290
3291826
3292046
3359159
3493069
3501085
3504604
3504780
3515874
3577245
3658313
3857500
3967274
4070078
4070423
4078231
4078244
4087904
4169668
4229797
4394762
4399988
4508373
4608720
136
167
171
178
185
189
191
195
247
248
258
259
263
290
313
314
315
316
323
343
344
346
347
348
372
384
424
432
440
442
447
448
449
465
469
480
482
498
506

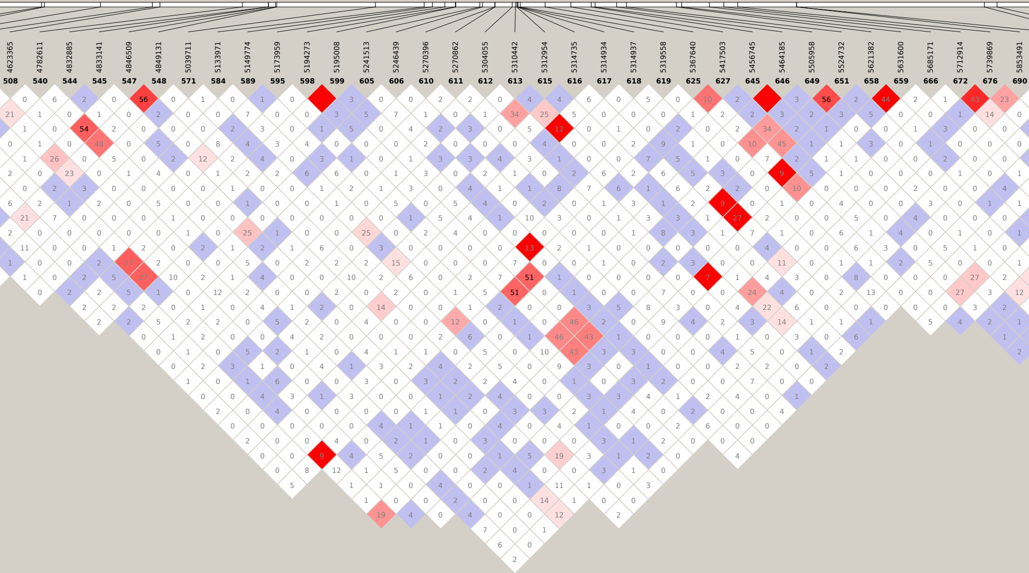

4623365 4782611 4832885 4833141 4846509 4849131 5039711 5133971 5149774 5173959 5194273 5195008 5241513 5246439 5270396 5270862 5304055 5310442 5312954 5314735 5314934 5314937 5319558 5367640 5417503 5456745 5464185 5505958 5524732 5621382 5631600 5685171 5712914 5739869 5853491
508 540 544 545 547 548 571 584 589 595 598 599 605 606 610 611 612 613 615 616 617 618 619 625 627 645 646 649 651 658 659 666 672 676 690

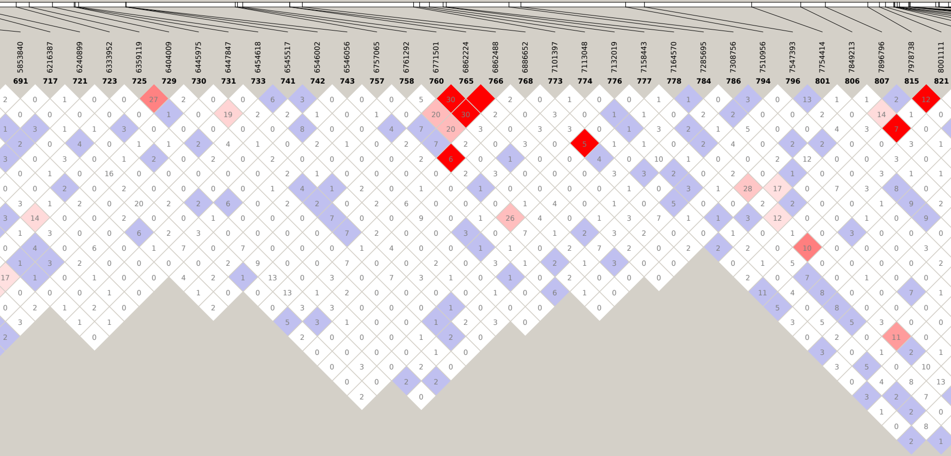
5853840 6216387 6240899 6333952 6359119 6404009 6445975 6447847 6454618 6545517 6546002 6546056 6757065 6761292 6771501 6862224 6862488 6886652 7101397 7113048 7132019 7158443 7164570 7285695 7308756 7510956 7547393 7754414 7849213 7896796 7978738 8001111
691 717 721 723 725 729 730 731 733 741 742 743 757 758 760 765 766 768 773 774 776 777 778 784 786 794 796 801 806 807 815 821

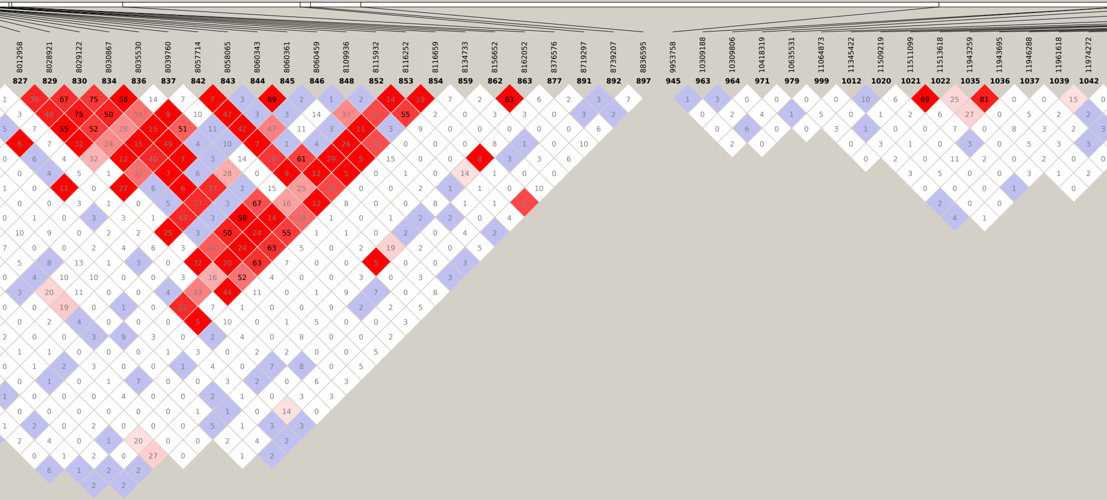

8012958 8028921 8029122 8030867 8035530 8039760 8057714 8058065 8060343 8060361 8060459 8109936 8115932 8116252 8116659 8134733 8156652 8162052 8376576 8719297 8739207 8836595 9953758 10309188 10309806 10418319 10635531 11064873 11345422 11509219 11511099 11513618 11943259 11943695 11946288 11961618 11974272
827 829 830 834 836 837 842 843 844 845 846 848 852 853 854 859 862 863 877 891 892 897 945 963 964 971 979 999 1012 1020 1021 1022 1035 1036 1037 1039 1042

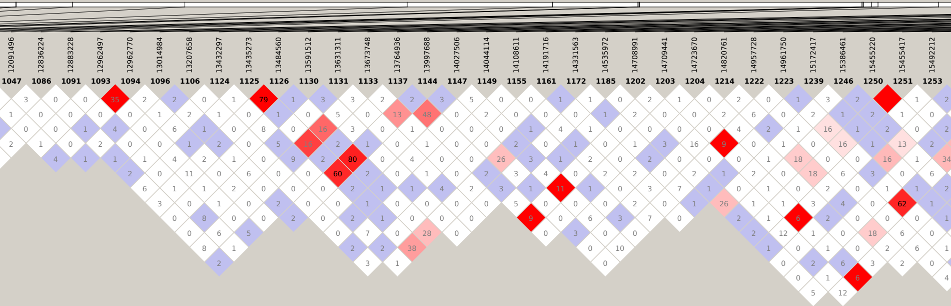
12091496 12836224 12883228 12962497 12962770 13014984 13207658 13432297 13435273 13484560 13591512 13631311 13673748 13764936 13997688 14027506 14044114 14108611 14191716 14331563 14535972 14708991 14709441 14723670 14820761 14957728 14961750 15172417 15386461 15455220 15455417 15492212
1047 1086 1091 1093 1094 1096 1106 1124 1125 1126 1130 1131 1133 1137 1144 1147 1149 1155 1161 1172 1185 1202 1203 1204 1214 1222 1223 1239 1246 1250 1251 1253

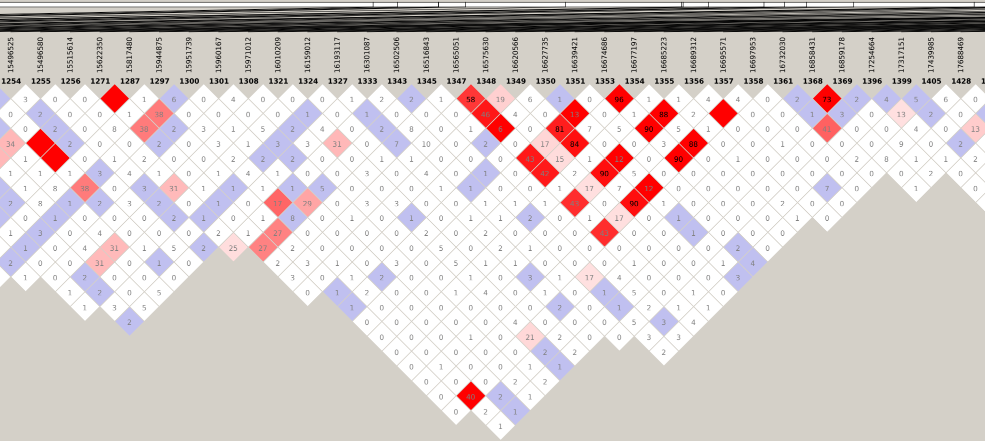

15496525
15496580
15515614
15622350
15817480
15944875
15951739
15960167
15971012
16010209
16159012
16193117
16301087
16502506
16516843
16565051
16575630
16620566
16627735
16639421
16674686
16677197
16685223
16689312
16695571
16697953
16732030
16858431
16859178
17254664
17317151
17439985
17688469
1254
1255
1256
1271
1287
1297
1300
1301
1308
1321
1324
1327
1333
1343
1345
1347
1348
1349
1350
1351
1353
1354
1355
1356
1357
1358
1361
1368
1369
1396
1399
1405
1428

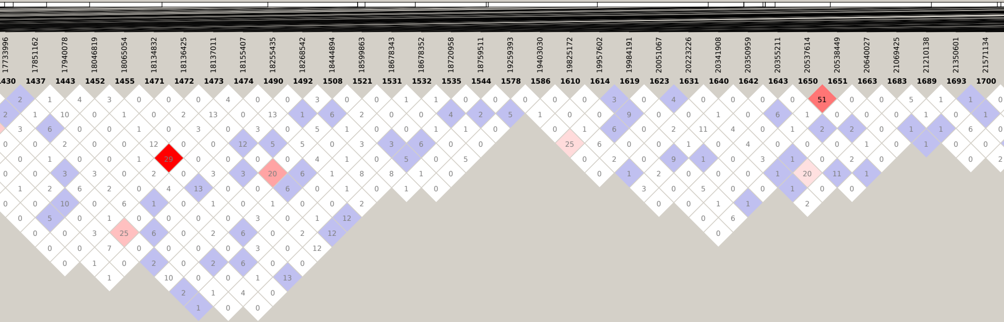
17733996
17851162
17940078
18046819
18065054
18134832
18136425
18137011
18155407
18255435
18268542
18444894
18599863
18678343
18678352
18720958
18759511
19259393
19403030
19825172
19957602
19984191
20051067
20223226
20341908
20350959
20355211
20537614
20538449
20640027
21069425
21210138
21350601
21571134
430 1437 1443 1452 1455 1471 1472 1473 1474 1490 1492 1508 1521 1531 1532 1535 1544 1578 1586 1610 1614 1619 1623 1631 1640 1642 1643 1650 1651 1663 1683 1689 1693 1700

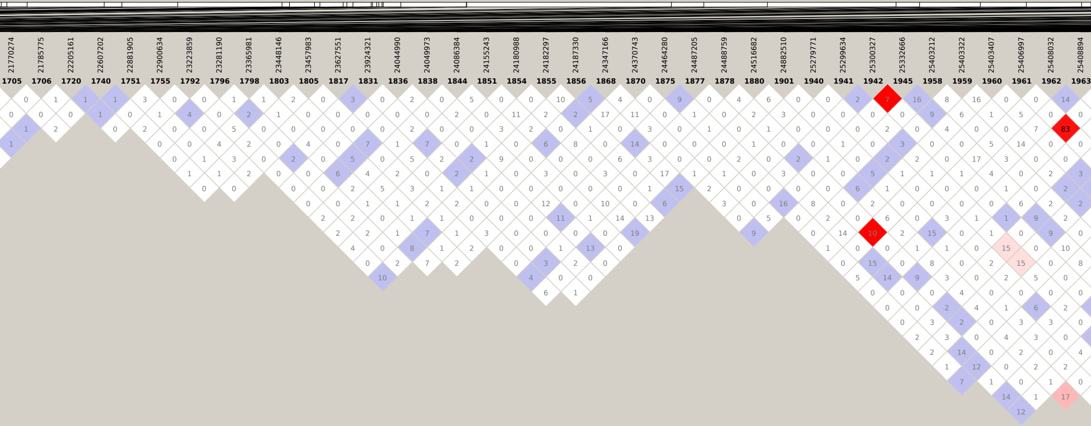
21770274 21785775 22205161 22607202 22881905 22900634 23223859 23281190 23365981 23448146 23457983 23627551 23924321 24044990 24049973 24086384 24155243 24180988 24182297 24187330 24347166 24370743 24464280 24487205 24488759 24516682 24882510 25279771 25299634 25300327 25332666 25403212 25403322 25403407 25406997 25408032 25408894
1705 1706 1720 1740 1751 1755 1792 1796 1798 1803 1805 1817 1831 1836 1838 1844 1851 1854 1855 1856 1868 1870 1875 1877 1878 1880 1901 1940 1941 1942 1945 1958 1959 1960 1961 1962 1963

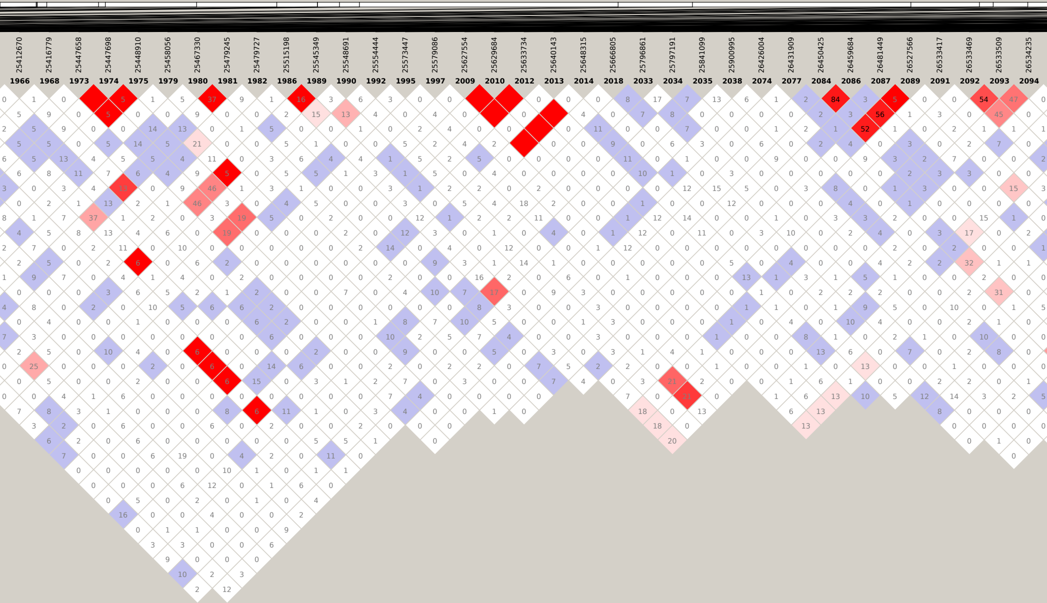

25412670 1966
25416779 1968
25447658 1973
25447698 1974
25448910 1975
25458056 1979
25467330 1980
25479245 1981
25479727 1982
25515198 1986
25545349 1989
25548691 1990
25554444 1992
25573447 1995
25579086 1997
25627554 2009
25629684 2010
25633734 2012
25640143 2013
25648315 2014
25666805 2018
25796861 2033
25797191 2034
25841099 2035
25900995 2038
26426004 2074
26431909 2077
26450425 2084
26459684 2086
26481449 2087
26527566 2089
26533417 2091
26533469 2092
26533509 2093
26534235 2094

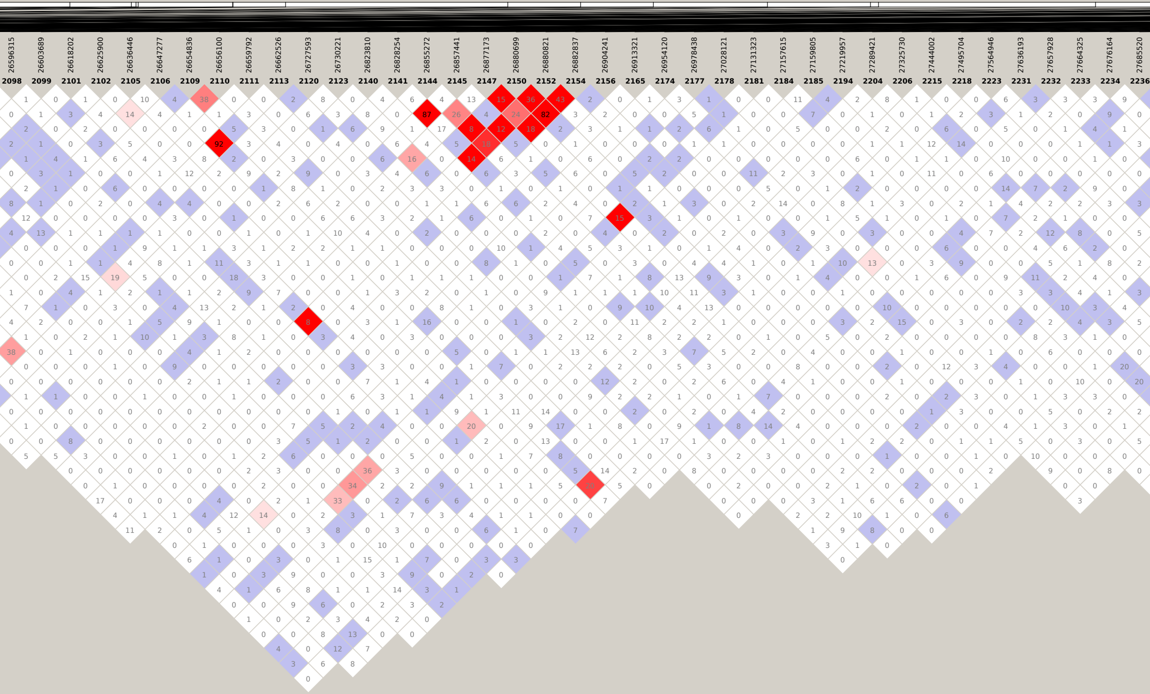
26596315
26603689
26618202
26625900
26636446
26647277
26654836
26656100
26659792
26662526
26727593
26730221
26823810
26828254
26855272
26857441
26877173
26880699
26880821
26882837
26904241
26913321
26954120
26978438
27028121
27131323
27157615
27159805
27219957
27289421
27325730
27444002
27495704
27564946
27636193
27657928
27664325
27676164
27685520
2098 2099 2101 2102 2105 2106 2109 2110 2111 2113 2120 2123 2140 2141 2144 2145 2147 2150 2152 2154 2156 2165 2174 2177 2178 2181 2184 2185 2194 2204 2206 2215 2218 2223 2231 2232 2233 2234 2236

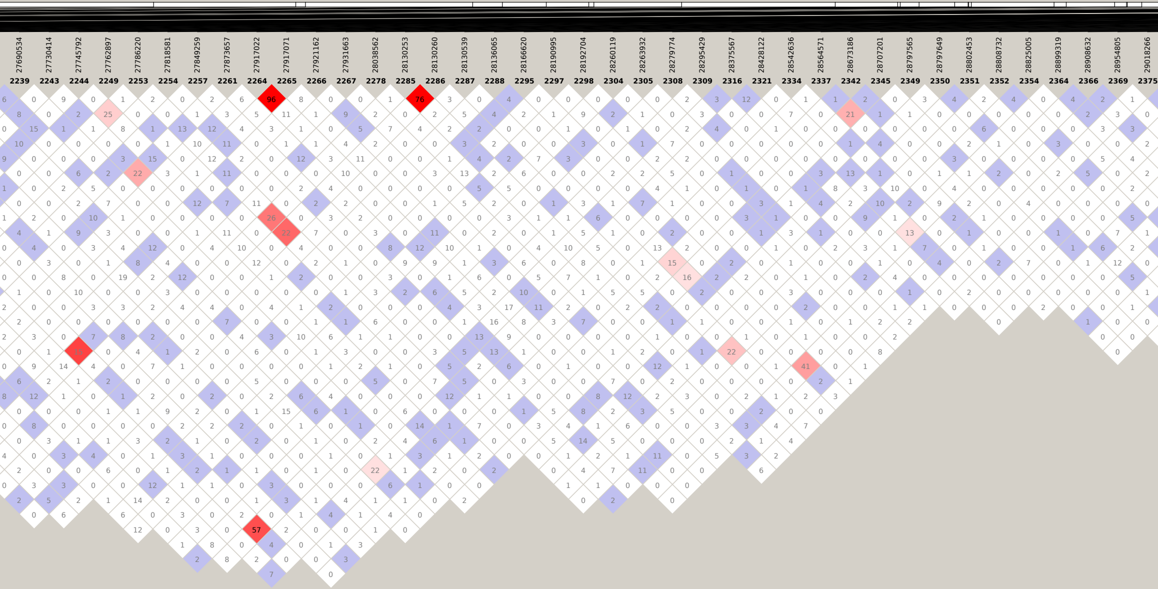
27690534
27730414
27745792
27762897
27786220
27818581
27849259
27873657
27917022
27917071
27921162
27931663
28038562
28130253
28130260
28130539
28136065
28166620
28190995
28192704
28260119
28263932
28279774
28295429
28375567
28428122
28542636
28564571
28673186
28707201
28797565
28797649
28802453
28808732
28825005
28899319
28908632
28954805
29018266
2239 2243 2244 2249 2253 2254 2257 2261 2264 2265 2266 2267 2278 2285 2286 2287 2288 2295 2297 2298 2304 2305 2308 2309 2316 2321 2334 2337 2342 2345 2349 2350 2351 2352 2354 2364 2366 2369 2375

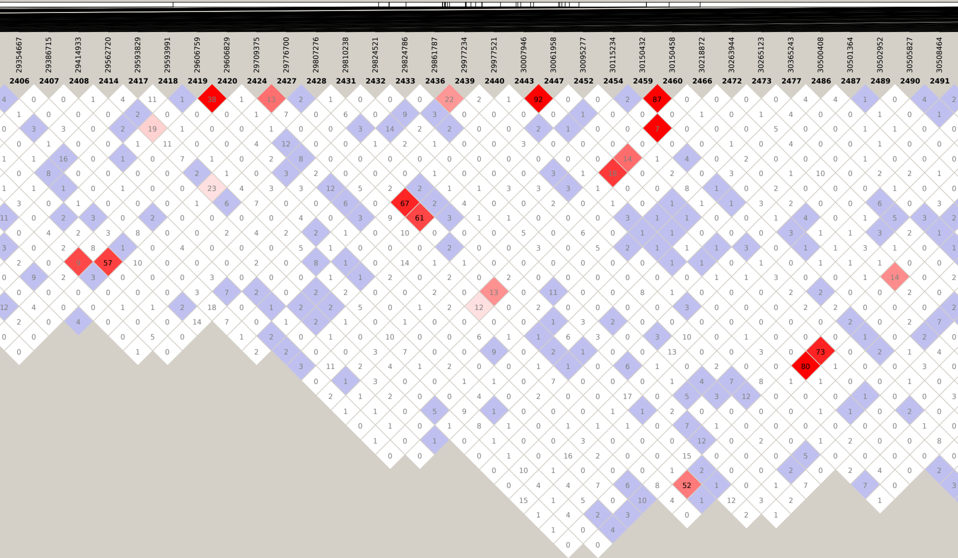

29354667 29386715 29414933 29562720 29593829 29593991 29606759 29606829 29709375 29776700 29807276 29810238 29824521 29824786 29861787 29977234 29977521 30007946 30061958 30095277 30115234 30150432 30150458 30218872 30263944 30265123 30365243 30500408 30501364 30502952 30505827 30508464
2406 2407 2408 2414 2417 2418 2419 2420 2424 2427 2428 2431 2432 2433 2436 2439 2440 2443 2447 2452 2454 2459 2460 2466 2472 2473 2477 2486 2487 2489 2490 2491

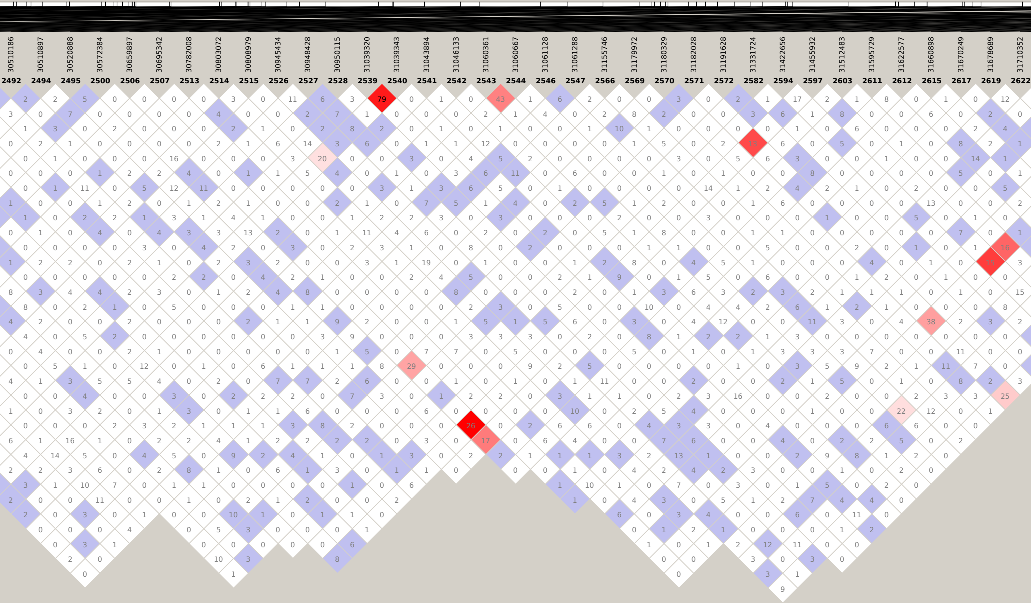
30510186 30510897 30520888 30572384 30659897 30695342 30782008 30803072 30808979 30945434 30948428 30950115 31039320 31039343 31043894 31046133 31060361 31060667 31061128 31061288 31155746 31179972 31180329 31182028 31191628 31331724 31422656 31455932 31512483 31595729 31622577 31660898 31670249 31678689 31710352
2492 2494 2495 2500 2506 2507 2513 2514 2515 2526 2527 2528 2539 2540 2541 2542 2543 2544 2546 2547 2566 2569 2570 2571 2572 2582 2594 2597 2603 2611 2612 2615 2617 2619 2622

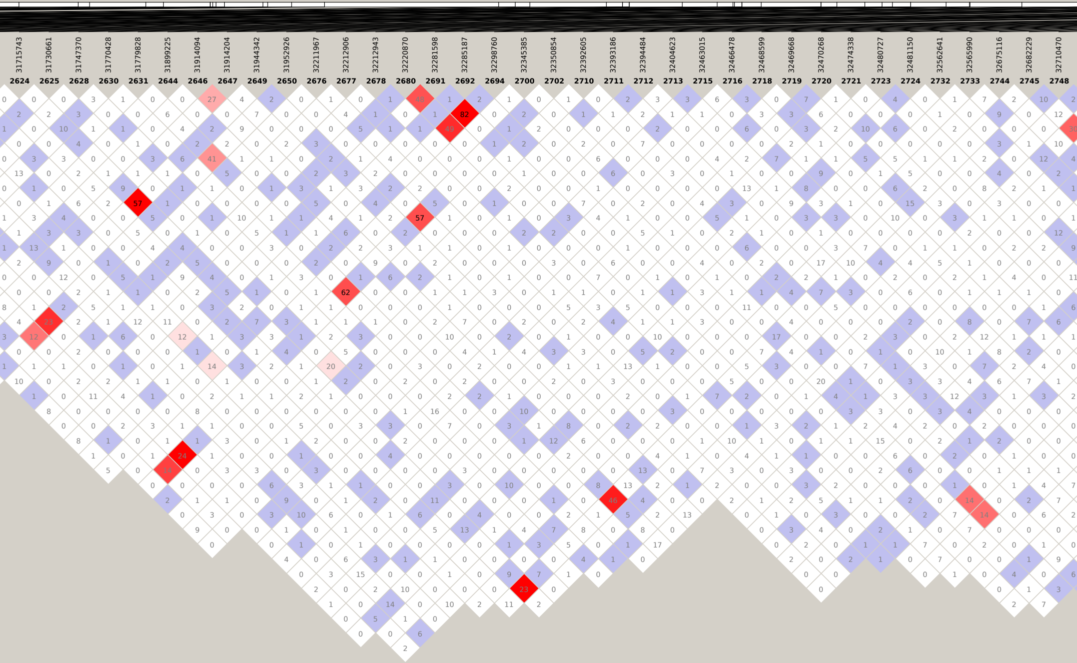

31715743 31730661 31747370 31770428 31779828 31899225 31914094 31914204 31944342 31952926 32211967 32212906 32212943 32220870 32281598 32285187 32298760 32345385 32350854 32392605 32393186 32394484 32404623 32463015 32466478 32468599 32469668 32470268 32474338 32480727 32481150 32562641 32565990 32675116 32682229 32710470
2624 2625 2628 2630 2631 2644 2646 2647 2649 2650 2676 2677 2678 2680 2691 2692 2694 2700 2702 2710 2711 2712 2713 2715 2716 2718 2719 2720 2721 2723 2724 2732 2733 2744 2745 2748

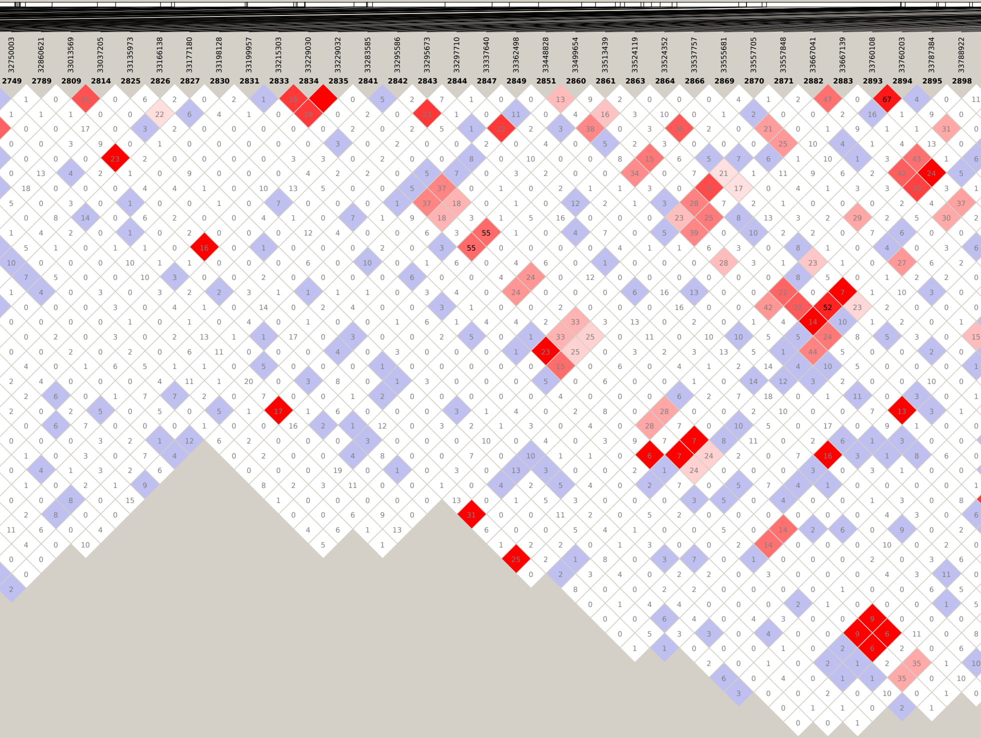
32750003
2749
32860621
2789
33013569
2809
33037205
2814
33135973
2825
33166138
2826
33177180
2827
33198128
2830
33199957
2831
33215303
2833
33229030
2834
33229032
2835
33283585
2841
33295586
2842
33295673
2843
33297710
2844
33337640
2847
33362498
2849
33448828
2851
33499654
2860
33513439
2861
33524119
2863
33524352
2864
33537757
2866
33555681
2869
33557705
2870
33557848
2871
33667041
2882
33667139
2883
33760108
2893
33760203
2894
33787384
2895
33788922
2898

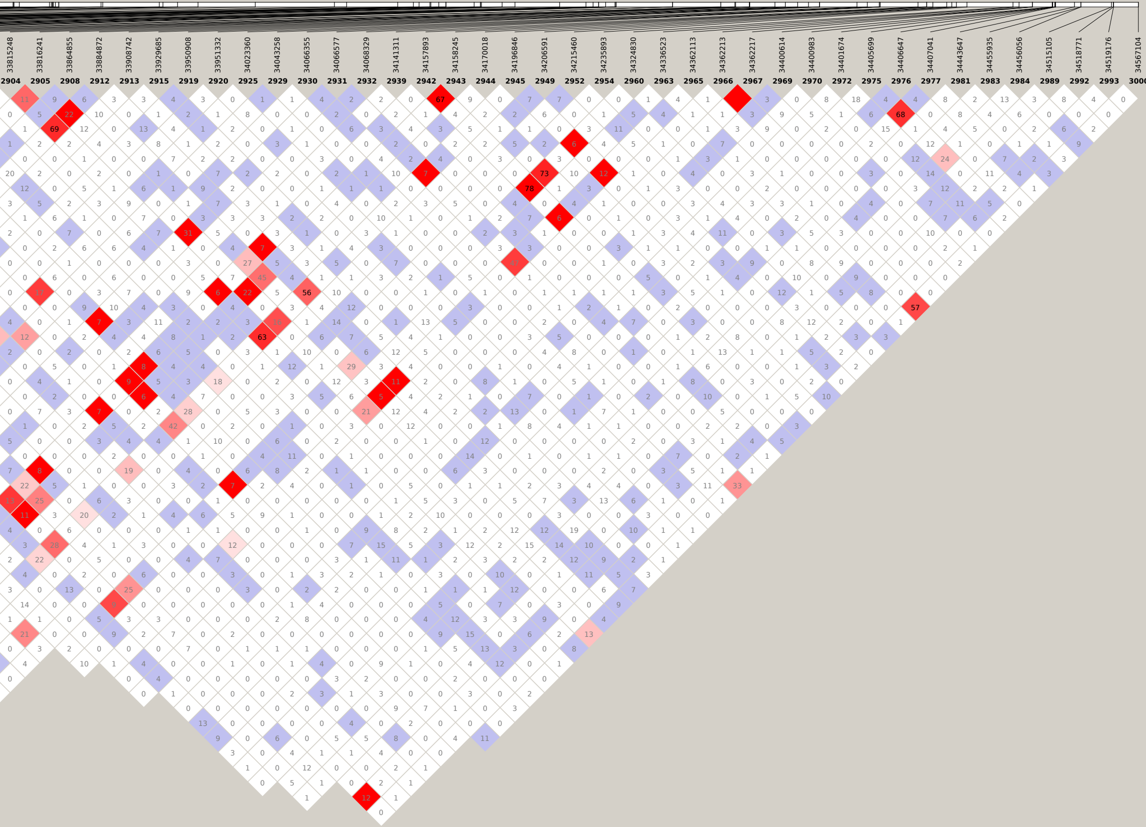
33815248
33816241
33864855
33884872
33908742
33929685
33950908
33951332
34023360
34043258
34066355
34066577
34068329
34141311
34157893
34158245
34170018
34196846
34206591
34215460
34235893
34324830
34336523
34362113
34362213
34362217
34400614
34400983
34401674
34405699
34406647
34407041
34443647
34455935
34456056
34515105
34518771
34519176
34567104
2904 2905 2908 2912 2913 2915 2919 2920 2925 2929 2930 2931 2932 2939 2942 2943 2944 2945 2949 2952 2954 2960 2963 2965 2966 2967 2969 2970 2972 2975 2976 2977 2981 2983 2984 2989 2992 2993 3000

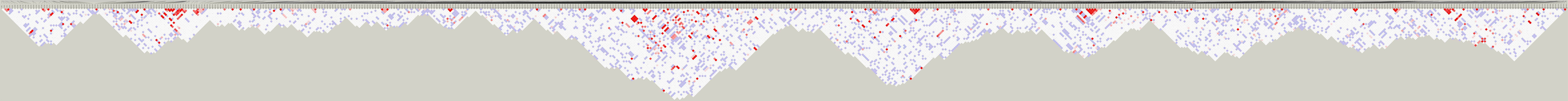

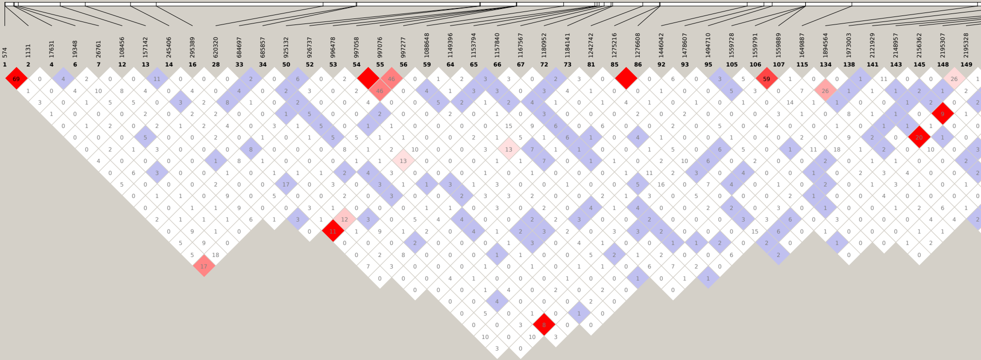
574 1131 17631 19348 26761 108456 157142 245406 295389 620320 684697 685857 925132 926737 996478 997058 997076 997277 1088648 1149396 1153794 1157840 1167567 1180952 1184141 1242742 1275216 1276608 1446042 1478607 1494710 1559728 1559791 1559889 1649887 1894564 1973003 2121929 2148957 2156362 2195307 2195328
1 2 4 6 7 12 13 14 16 28 33 34 50 52 53 54 55 56 59 64 65 66 67 72 73 81 85 86 92 93 95 105 106 107 115 134 138 141 143 145 148 149

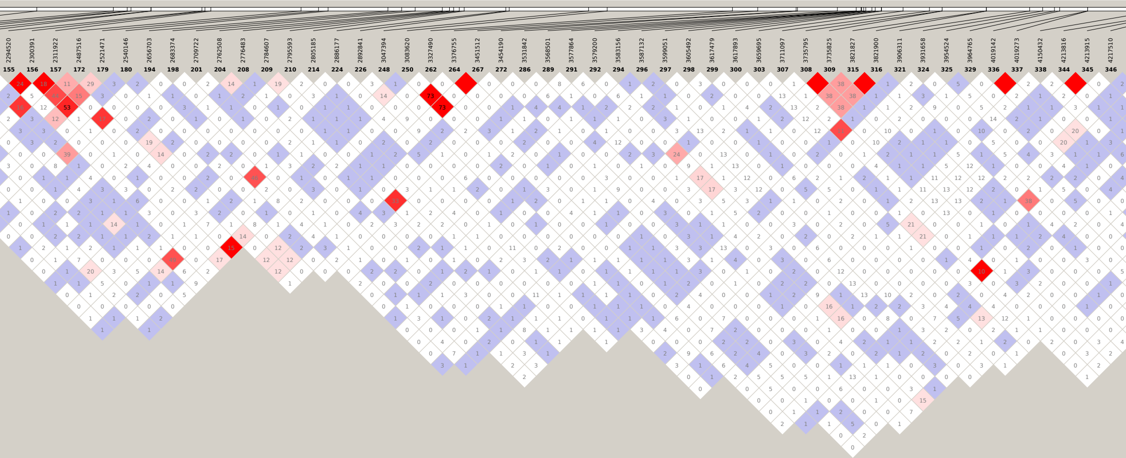
2294520 155
2300391 156
2311922 157
2487516 172
2521471 179
2540146 180
2656703 194
2683374 198
2709722 201
2762508 204
2776483 208
2784607 209
2795593 210
2805185 214
2886177 224
2892841 226
3047394 248
3083620 250
3327490 262
3376755 264
3451512 267
3454190 272
3531842 286
3568501 289
3577864 291
3579200 292
3583156 294
3587132 296
3599051 297
3605492 298
3617479 299
3617893 300
3659695 303
3711097 307
3735795 308
3735825 309
3821827 315
3821900 316
3906311 321
3931658 324
3954524 325
3964765 329
4019142 336
4019273 337
4150432 338
4213816 344
4213915 345
4217510 346

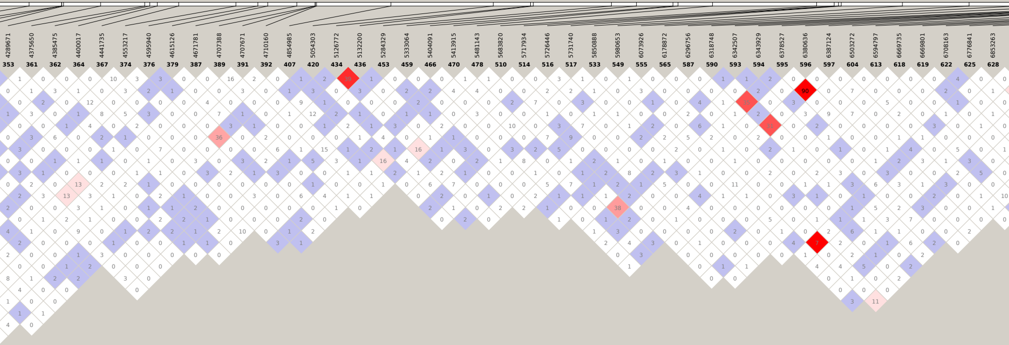

4289671
353
4375650
361
4385475
362
4400017
364
4441735
367
4553217
374
4595940
376
4615126
379
4671781
387
4707388
389
4707671
391
4710160
392
4854985
407
5054303
420
5126772
434
5132200
436
5284329
453
5333064
459
5404091
466
5413915
470
5481143
478
5683820
510
5717934
514
5726446
516
5731740
517
5850888
533
5980653
549
6073926
555
6178872
565
6296756
587
6318748
590
6342507
593
6343929
594
6378527
595
6380636
596
6387124
597
6503272
604
6594797
613
6669735
618
6669801
619
6708163
622
6776841
625
6853263
628

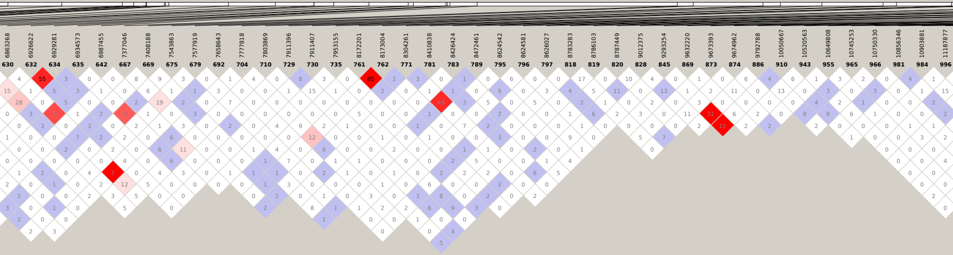
6863268 6926622 6929281 6934573 6987455 7377046 7408188 7543863 7577919 7658643 7777818 7803869 7911396 7911407 7953155 8172201 8173004 8304261 8410838 8426424 8472461 8624542 8624581 8626027 8783283 8786103 8787449 9012375 9293254 9632220 9673393 9674962 9792788 10050667 10520563 10649808 10745253 10750330 10856346 10903881 11187877
630 632 634 635 642 667 669 675 679 692 704 710 729 730 735 761 762 771 781 783 789 795 796 797 818 819 820 828 845 869 873 874 886 910 943 955 965 966 981 984 996

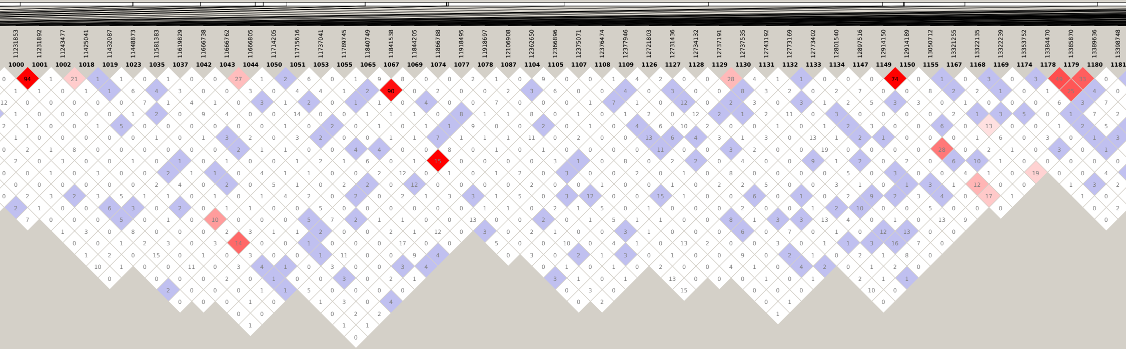
11231853 11231892 11243477 11425041 11432087 11448873 11581383 11619829 11666738 11666762 11666805 11714205 11715616 11737041 11789745 11840749 11841538 11844205 11866788 11918495 11918697 12106908 12362650 12366896 12375071 12376474 12377946 12721803 12731436 12734132 12737191 12737535 12743192 12773169 12773402 12801540 12897516 12914150 12914189 13050712 13321255 13322135 13322239 13353752 13384470 13385870 13389636 13398748
1000 1001 1002 1018 1019 1023 1035 1037 1042 1043 1044 1050 1051 1053 1055 1065 1067 1069 1074 1077 1078 1087 1104 1105 1107 1108 1109 1126 1127 1128 1129 1130 1131 1132 1133 1134 1147 1149 1150 1155 1167 1168 1169 1174 1178 1179 1180 1181

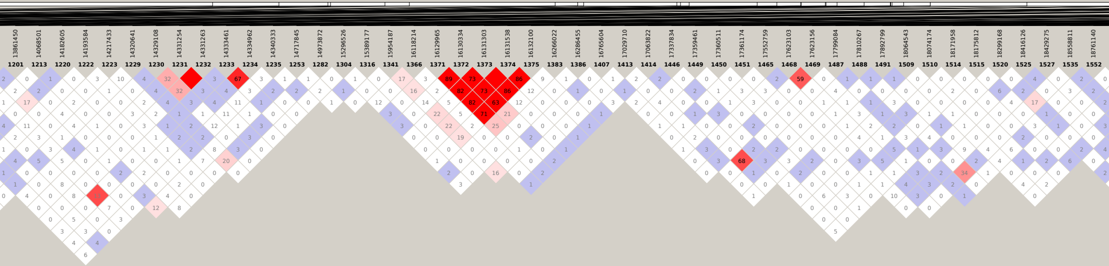
13861450 1201
14068501 1213
14182605 1220
14193584 1222
14217433 1223
14320641 1229
14329108 1230
14331254 1231
14331263 1232
14333461 1233
14334962 1234
14340333 1235
14717845 1253
14973872 1282
15296526 1304
15389177 1316
15954187 1341
16118214 1366
16129965 1371
16130334 1372
16131303 1373
16131538 1374
16132100 1375
16266022 1383
16286455 1386
16765604 1407
17029710 1413
17063822 1414
17337834 1446
17359461 1449
17360511 1450
17361174 1451
17552759 1465
17623103 1468
17623156 1469
17799084 1487
17810267 1488
17892799 1491
18064543 1509
18074174 1510
18171958 1514
18175812 1515
18299168 1520
18416126 1525
18429275 1527
18558811 1535
18761140 1552

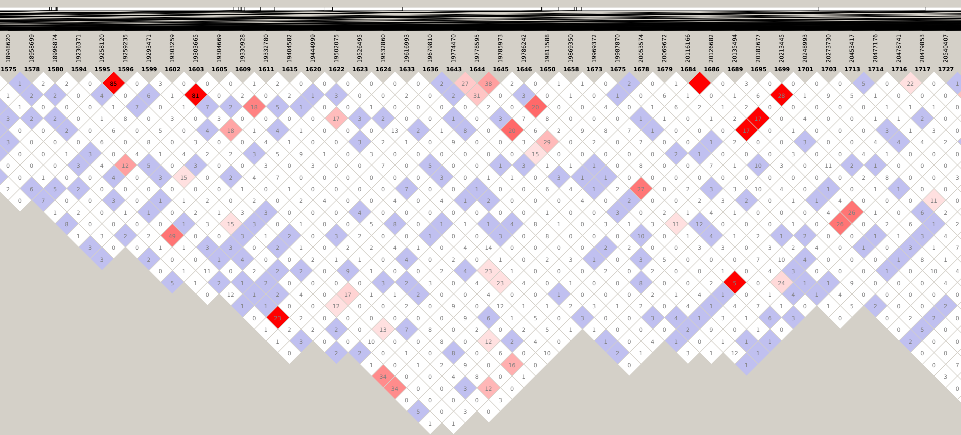
18948620 18958699 18996874 19236371 19258120 19259235 19293471 19303259 19303665 19304669 19330928 19332780 19404582 19444999 19502075 19526495 19532860 19616993 19679810 19774470 19778595 19785973 19786242 19811588 19869350 19969372 19987870 20053574 20069672 20116166 20126682 20135494 20182677 20213445 20248993 20273730 20453417 20477176 20478741 20479853 20540407
1575 1578 1580 1594 1595 1596 1599 1602 1603 1605 1609 1611 1615 1620 1622 1623 1624 1633 1636 1643 1644 1645 1646 1650 1658 1673 1675 1678 1679 1684 1686 1689 1695 1699 1701 1703 1713 1714 1716 1717 1727

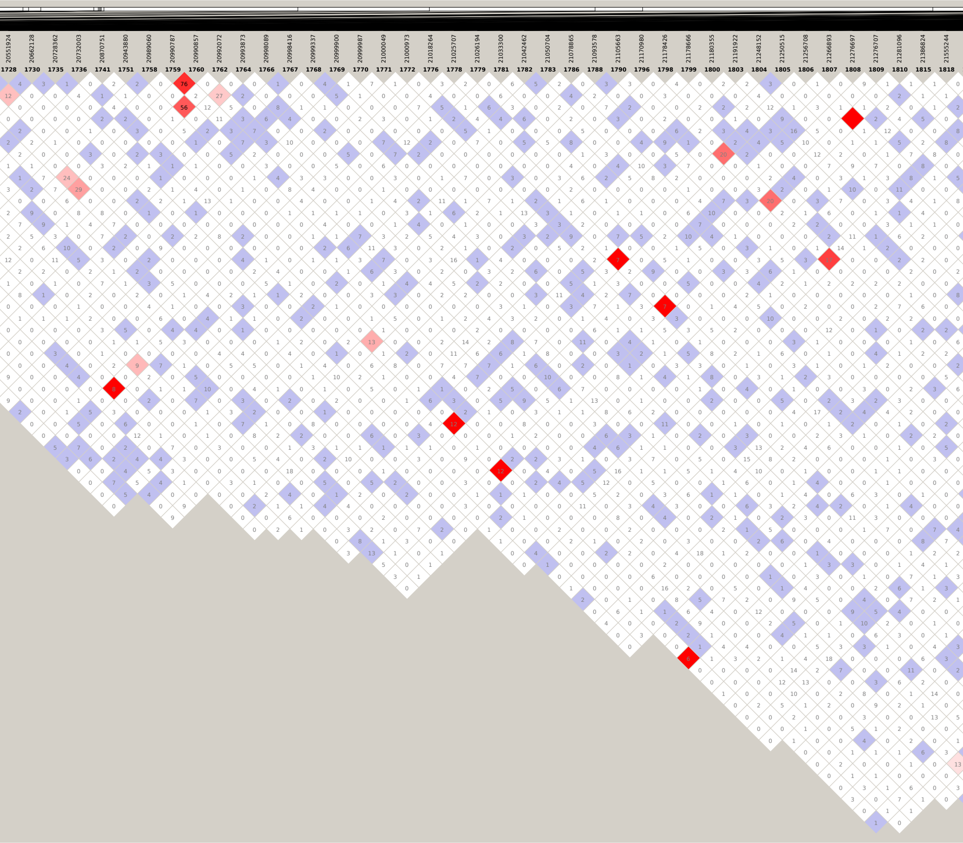
20551924 1728
20662128 1730
20728362 1735
20732003 1736
20870751 1741
20943880 1751
20989060 1758
20990787 1759
20990857 1760
20992072 1762
20993873 1764
20998089 1766
20998416 1767
20999337 1768
20999900 1769
20999987 1770
21000049 1771
21000973 1772
21018264 1776
21025707 1778
21026194 1779
21033300 1781
21042462 1782
21050704 1783
21078865 1786
21093578 1788
21105663 1790
21170980 1796
21178426 1798
21178666 1799
21180355 1800
21191922 1803
21248152 1804
21250515 1805
21256708 1806
21266893 1807
21276697 1808
21276707 1809
21281096 1810
21386824 1815
21555244 1818

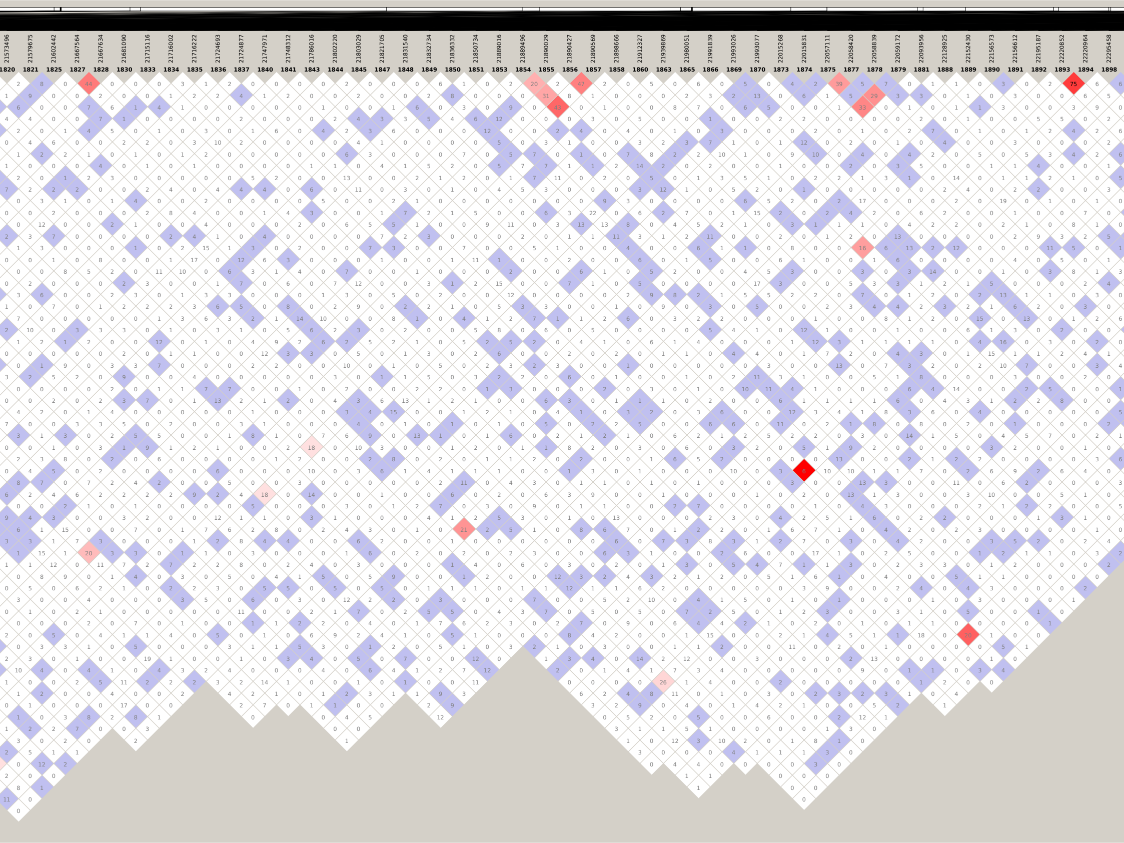

21573496 21579675 21602442 21667564 21667634 21681090 21715116 21716002 21716222 21724693 21724877 21747971 21748312 21786016 21802220 21803029 21821705 21831540 21832734 21836332 21850734 21889016 21889496 21890029 21890427 21890569 21898666 21912327 21939869 21980051 21991839 21993026 21993077 22015268 22015831 22057111 22058420 22058839 22059172 22093956 22128925 22152430 22156573 22156612 22195187 22220852 22220964 22295458
1820 1821 1825 1827 1828 1830 1833 1834 1835 1836 1837 1840 1841 1843 1844 1845 1847 1848 1849 1850 1851 1853 1854 1855 1856 1857 1858 1860 1863 1865 1866 1869 1870 1873 1874 1875 1877 1878 1879 1881 1888 1889 1890 1891 1892 1893 1894 1898

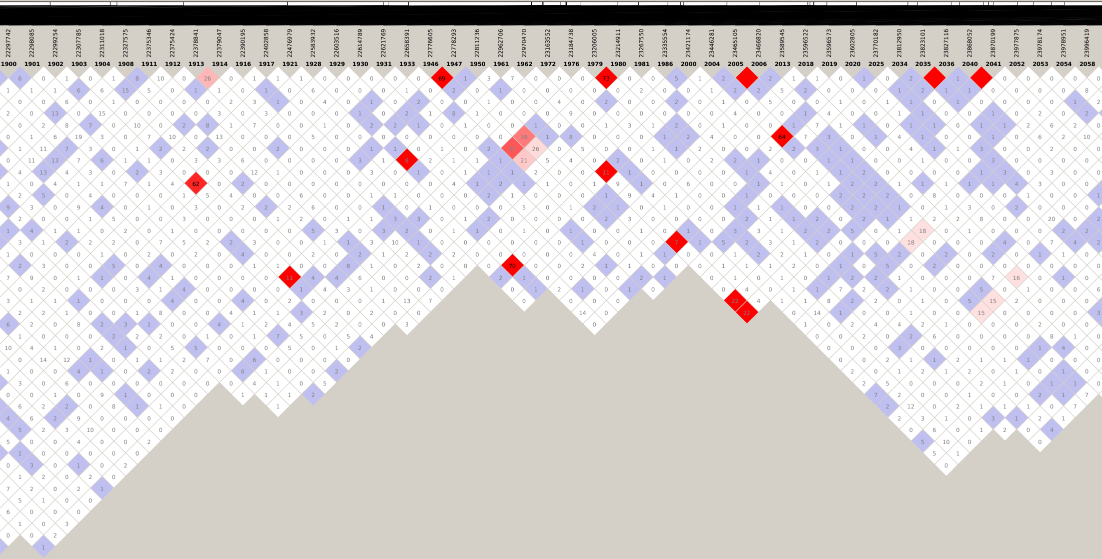
22297742 1900
22298085 1901
22299254 1902
22307785 1903
22311018 1904
22327575 1908
22375346 1911
22375424 1912
22378841 1913
22379047 1914
22390195 1916
22402858 1917
22476979 1921
22583932 1928
22603516 1929
22614789 1930
22621769 1931
22658391 1933
22776605 1946
22778293 1947
22811236 1950
22962706 1961
22970470 1962
23163552 1972
23184738 1976
23206005 1979
23214911 1980
23267550 1981
23335554 1986
23421174 2000
23446281 2004
23465105 2005
23466820 2006
23589545 2013
23596522 2018
23596573 2019
23602805 2020
23770182 2025
23812950 2034
23823101 2035
23827116 2036
23868052 2040
23870199 2041
23977875 2052
23978174 2053
23978951 2054
23996419 2058

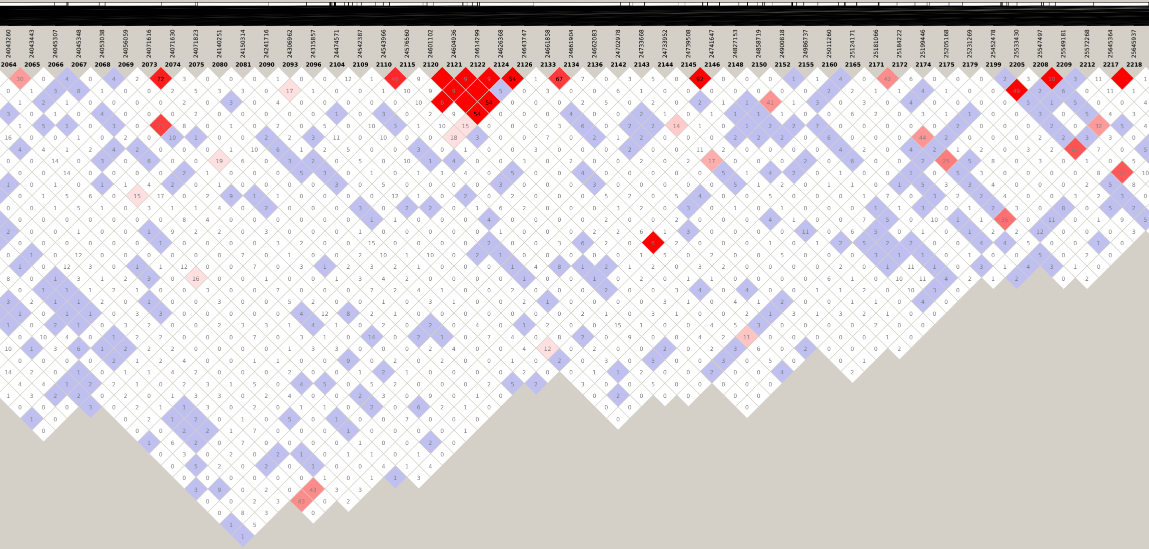
24043260 2064
24043443 2065
24045307 2066
24045348 2067
24053038 2068
24056059 2069
24071616 2073
24071630 2074
24071823 2075
24140251 2080
24150314 2081
24241716 2090
24306962 2093
24315857 2096
24474571 2104
24542387 2109
24543966 2110
24576560 2115
24601102 2120
24604936 2121
24614299 2122
24626368 2124
24643747 2126
24661858 2133
24661904 2134
24662083 2136
24702978 2142
24733668 2143
24733952 2144
24739508 2145
24741647 2146
24827153 2148
24858719 2150
24900818 2152
24986737 2155
25011260 2160
25124171 2165
25181066 2171
25184222 2172
25199446 2174
25205168 2175
25231269 2179
25452478 2199
25533430 2205
25547497 2208
25549181 2209
25572268 2212
25645364 2217
25645937 2218

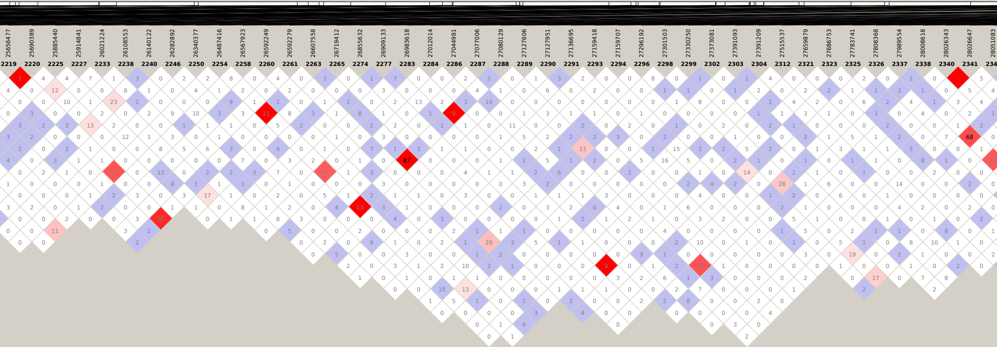
25656477 25690389 25885440 25914841 26021224 26108553 26140122 26282892 26340377 26487416 26567923 26592249 26592279 26607558 26719412 26855632 26909133 26983618 27012014 27044981 27077006 27080129 27127606 27127951 27136695 27159418 27159707 27296192 27301503 27330250 27373081 27391093 27391209 27515537 27659879 27686753 27783741 27808368 27989554 28008618 28026343 28026647 28051083
2219 2220 2225 2227 2233 2238 2240 2246 2250 2254 2258 2260 2261 2263 2265 2274 2277 2283 2284 2286 2287 2288 2289 2290 2291 2293 2294 2296 2298 2299 2302 2303 2304 2312 2321 2323 2325 2326 2337 2338 2340 2341

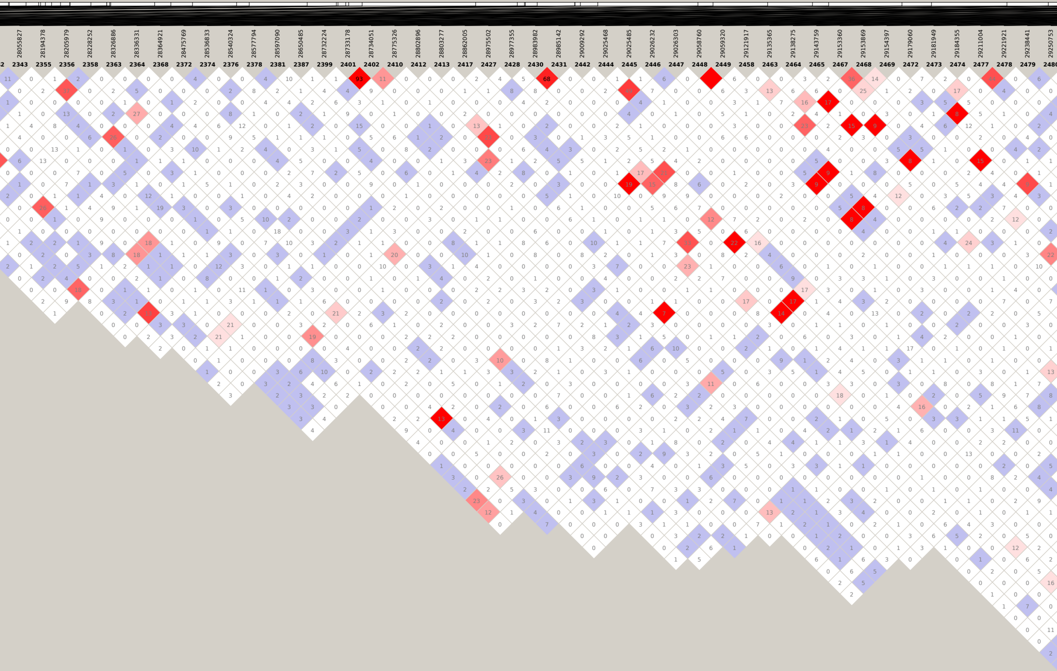

28055827
28194378
28205979
28228252
28326886
28336331
28364921
28475769
28536833
28540324
28577794
28597090
28650485
28732224
28733178
28734051
28775326
28802896
28803277
28862005
28975502
28977355
28983982
28985142
29009292
29025468
29025485
29026232
29026303
29058760
29059320
29121917
29135365
29138275
29143759
29153360
29153869
29154397
29179060
29181949
29184355
29211004
29221921
29238841
29250753
2343 2355 2356 2358 2363 2364 2368 2372 2374 2376 2378 2381 2387 2399 2401 2402 2410 2412 2413 2417 2427 2428 2430 2431 2442 2444 2445 2446 2447 2448 2449 2458 2463 2464 2465 2467 2468 2469 2472 2473 2474 2477 2478 2479 2480

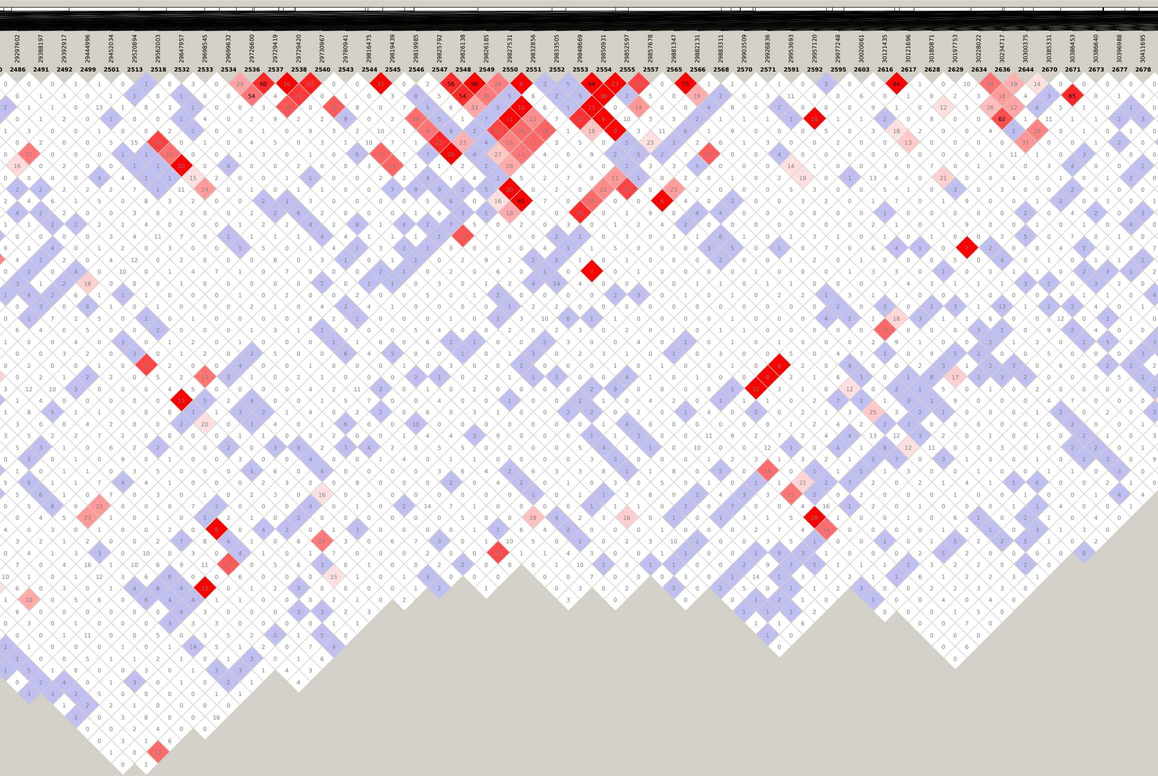
29297602 29388197 29392917 29444996 29452034 29520894 29562003 29647957 29698545 29699632 29726600 29729419 29729420 29730967 29780941 29816475 29819439 29819985 29825792 29826138 29826185 29827531 29832856 29833505 29848669 29850931 29852597 29857678 29881347 29882131 29883311 29903509 29926836 29953693 29957120 29977248 30020061 30121435 30121696 30180871 30197753 30228022 30234717 30300375 30385331 30386453 30386640 30396988 30411695
2486 2491 2492 2499 2501 2513 2518 2532 2533 2534 2536 2537 2538 2540 2543 2544 2545 2546 2547 2548 2549 2550 2551 2552 2553 2554 2555 2557 2565 2566 2568 2570 2571 2591 2592 2595 2603 2616 2617 2628 2629 2634 2636 2644 2670 2671 2673 2677 2678

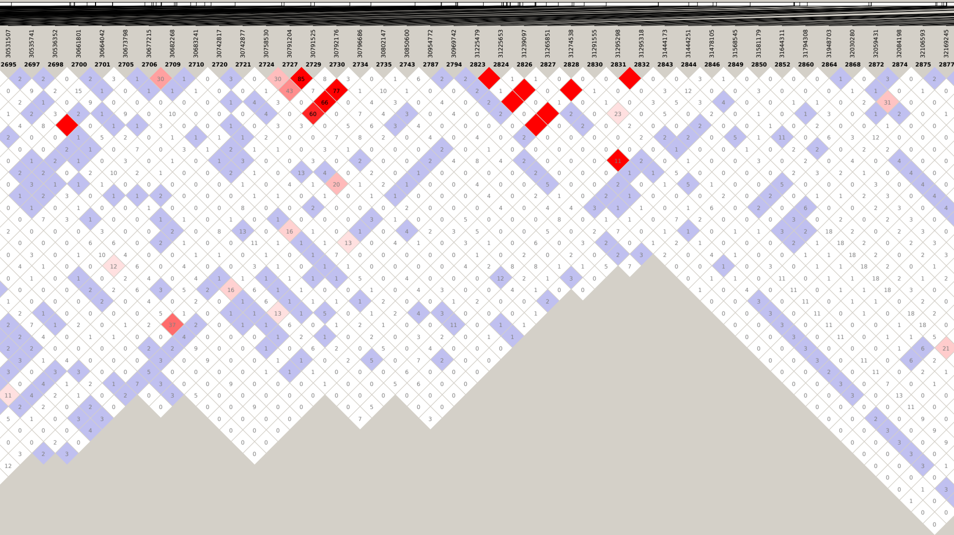
30531507 30535741 30536352 30661801 30664042 30673798 30677215 30682268 30683241 30742817 30742877 30758530 30791204 30791525 30792176 30796686 30802147 30850600 30954772 30969742 31225479 31225653 31239097 31265851 31274538 31291555 31295298 31295318 31444173 31444251 31478105 31568545 31581179 31644311 31794308 31948703 32030280 32059431 32084198 32106593 32169245
2695 2697 2698 2700 2701 2705 2706 2709 2710 2720 2721 2724 2727 2729 2730 2734 2735 2743 2787 2794 2823 2824 2826 2827 2828 2830 2831 2832 2843 2844 2846 2849 2850 2852 2860 2864 2868 2872 2874 2875 2877

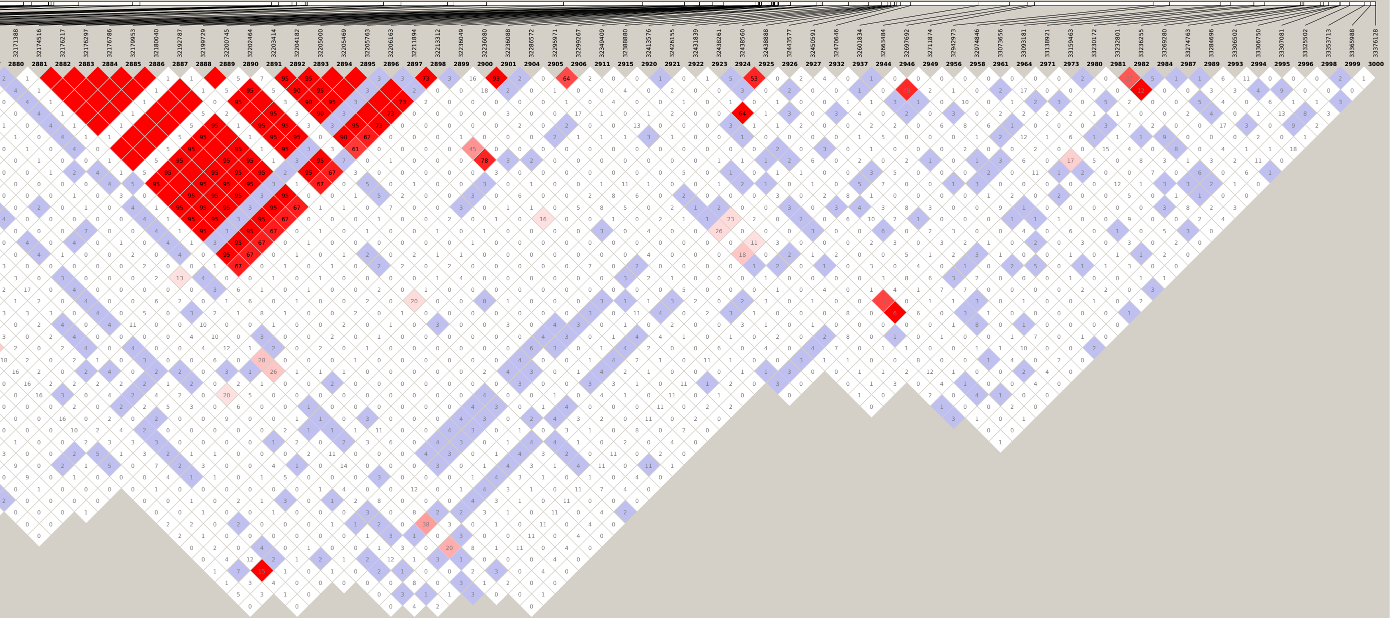

32171388 32174516 32176217 32176297 32176786 32179953 32180040 32192787 32199729 32200745 32202464 32203414 32204182 32205000 32205469 32205763 32206163 32211894 32213312 32236049 32236080 32236088 32286572 32295971 32299267 32349409 32388880 32413576 32426155 32431839 32438261 32438560 32438888 32443577 32450591 32470646 32601834 32663484 32697692 32711874 32942973 32974846 33073656 33093181 33138921 33159463 33230172 33232801 33236255 33269280 33274763 33284696 33306502 33306750 33307081 33325502 33353713 33365988 33376128
2880 2881 2882 2883 2884 2885 2886 2887 2888 2889 2890 2891 2892 2893 2894 2895 2896 2897 2898 2899 2900 2901 2904 2905 2906 2911 2915 2920 2921 2922 2923 2924 2925 2926 2927 2932 2937 2944 2946 2949 2956 2958 2961 2964 2971 2973 2980 2981 2982 2984 2987 2989 2993 2994 2995 2996 2998 2999 3000

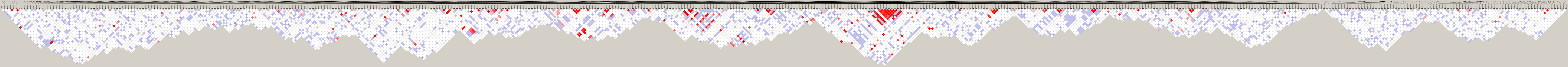

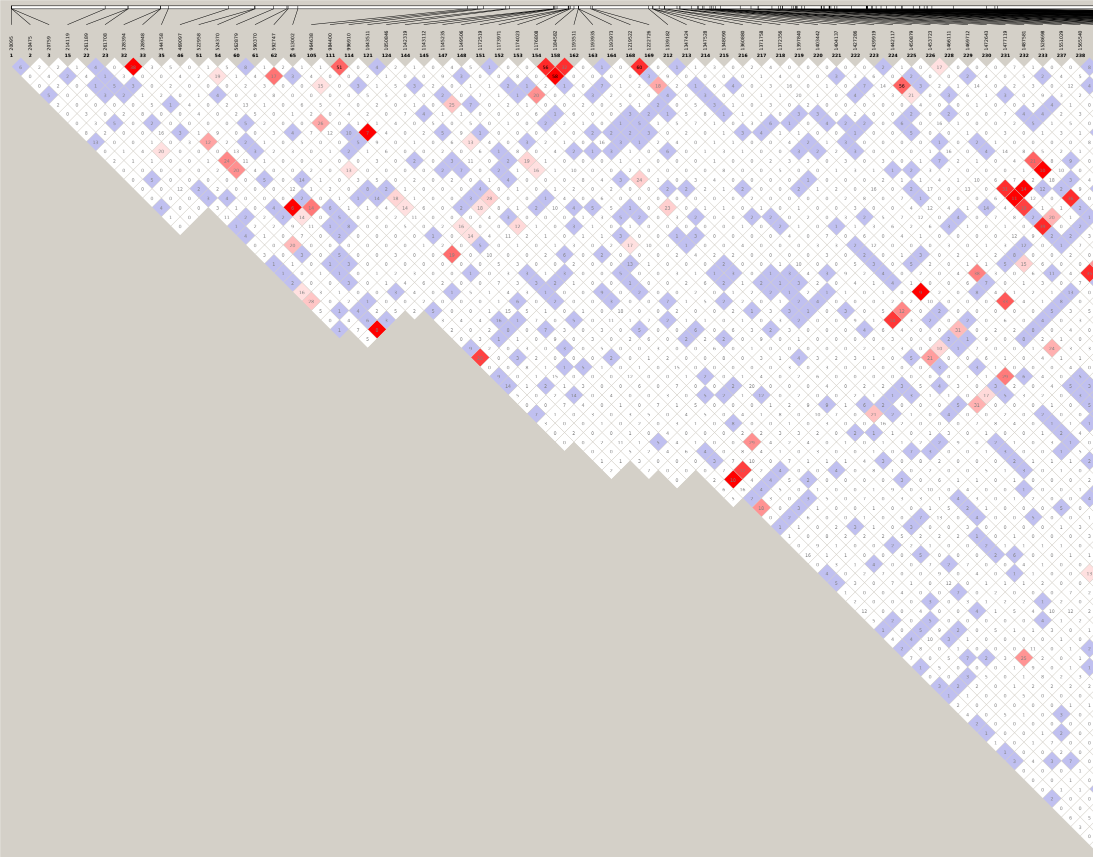
20095 20475 20759 214119 261189 261708 328394 328948 344758 469097 522958 524370 562879 590370 592747 613002 964638 984400 996910 1043511 1050846 1142319 1143112 1145235 1149506 1172519 1173971 1174023 1176808 1184582 1193511 1193935 1193973 1219522 1222726 1339182 1347424 1347528 1348090 1360880 1371758 1372356 1397840 1403442 1404137 1427286 1439919 1442117 1450879 1453723 1466111 1469712 1472643 1477119 1487581 1528698 1551029 1565540
1 2 3 15 22 23 32 33 35 46 51 54 60 61 62 65 105 111 114 121 124 144 145 147 148 151 152 153 154 158 162 163 164 168 169 212 213 214 215 216 217 218 219 220 221 222 223 224 225 226 228 229 230 231 232 233 237 238

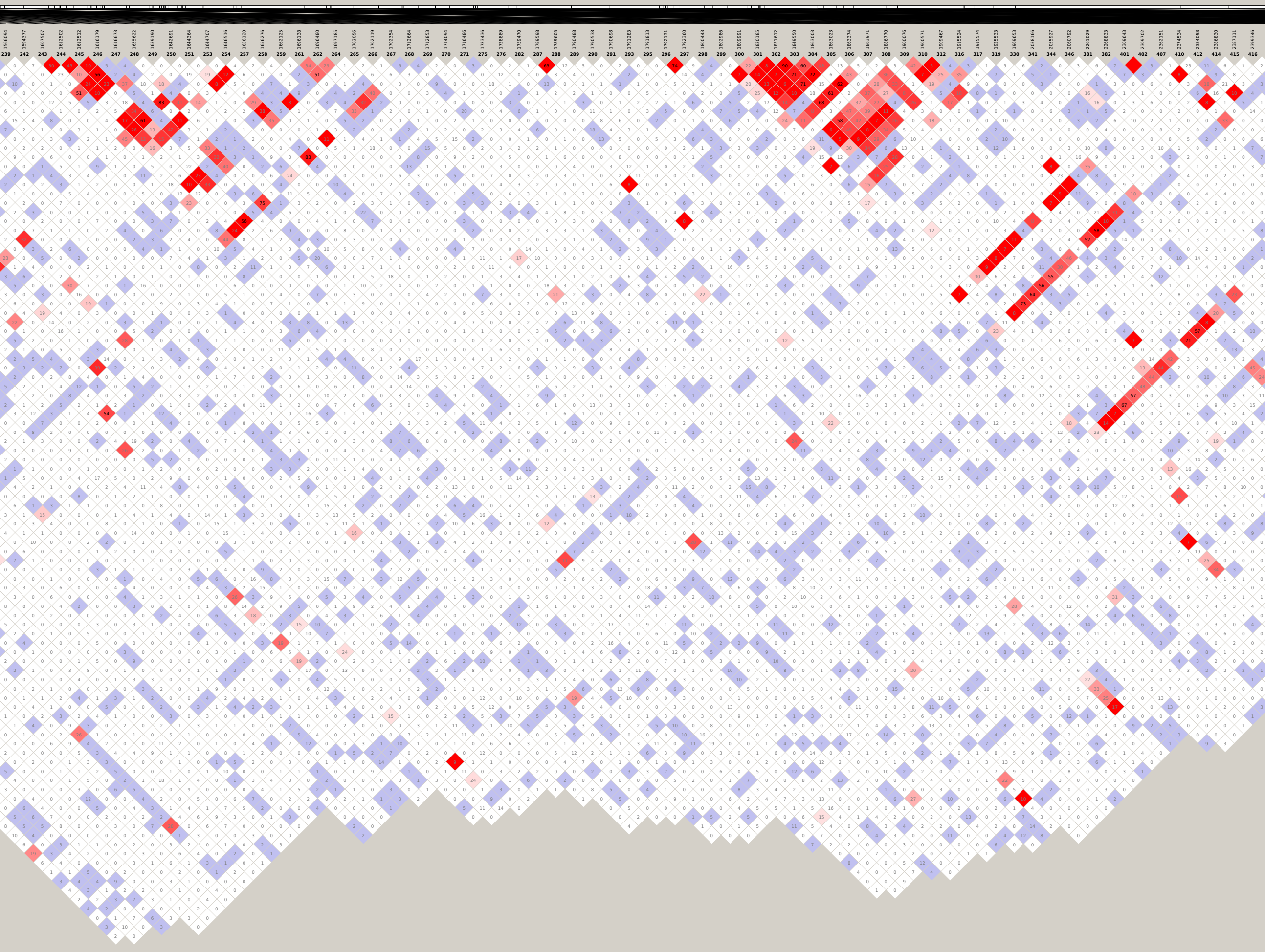

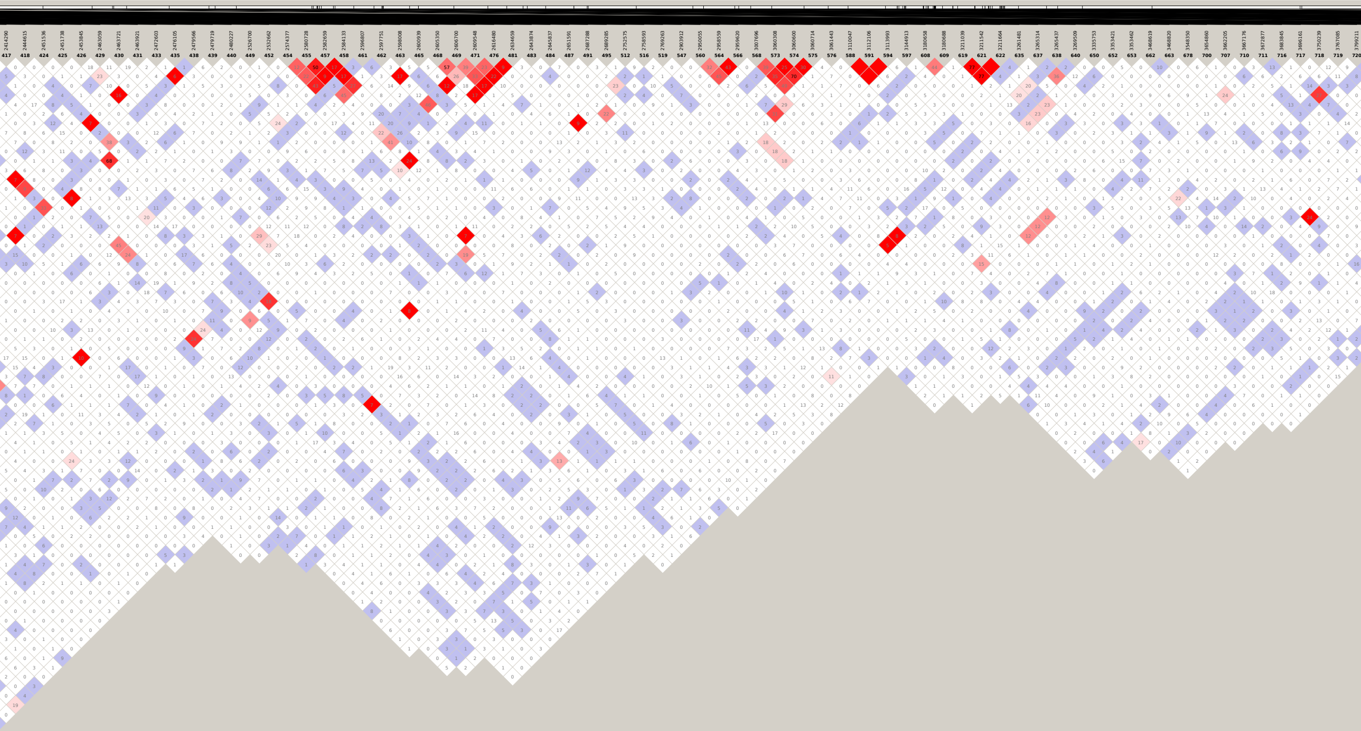

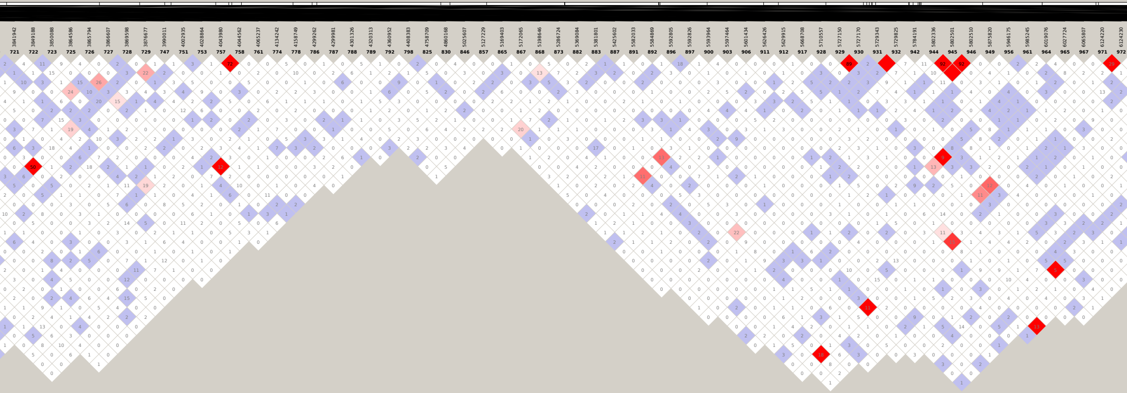
3841942 3849188 3850088 3864586 3865794 3866607 3869598 3876677 3990011 4002935 4028864 4043980 4044562 4065237 4134242 4158749 4299262 4299981 4301326 4320313 4360952 4408383 4758709 4860168 5025607 5127229 5169403 5172065 5198646 5286724 5369084 5381801 5425602 5582033 5584869 5592805 5592826 5593964 5597464 5601434 5626426 5629915 5668708 5710557 5727150 5727170 5729343 5729825 5784191 5802336 5802501 5802510 5875820 5946175 5985245 6019076 6027724 6065807 6124220 6124230
721 722 723 725 726 727 728 729 747 751 753 757 758 761 774 778 786 787 788 789 792 798 825 830 846 857 865 867 868 873 882 883 887 891 892 896 897 900 903 906 911 912 917 928 929 930 931 932 942 944 945 946 949 956 961 964 965 967 971 972

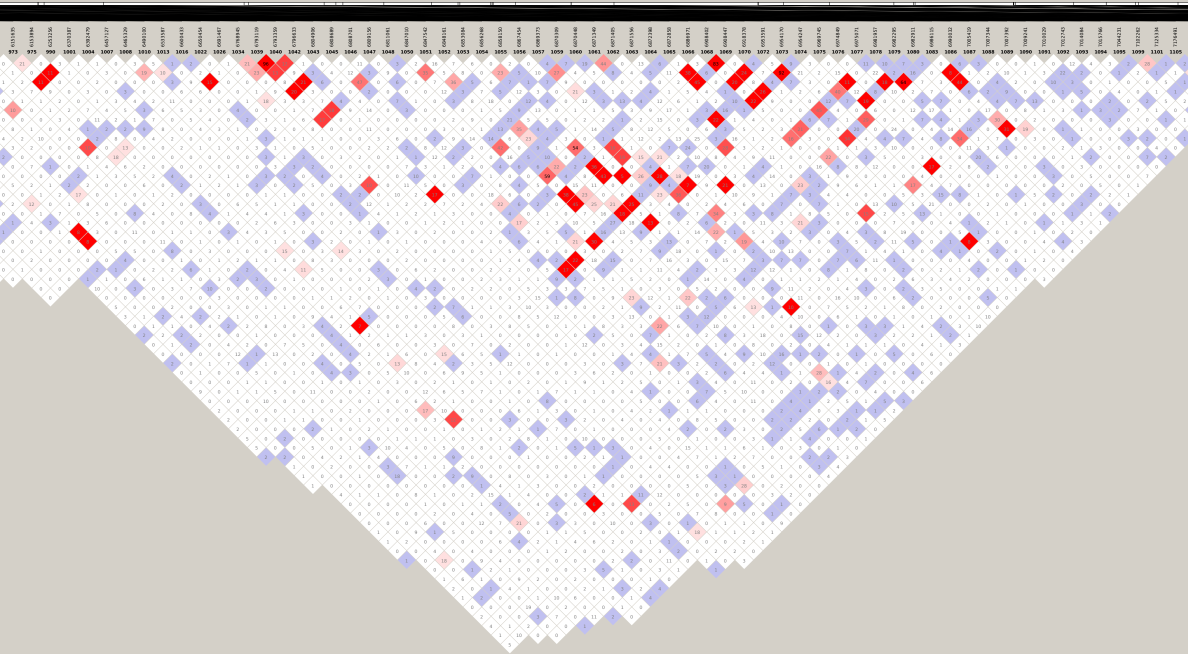

6151635 6153894 6253256 6370387 6392479 6457127 6465329 6490100 6533587 6600433 6650454 6691467 6768945 6793119 6793359 6796633 6804906 6808689 6808701 6809156 6811061 6847010 6847542 6848161 6853084 6856268 6858150 6867454 6869373 6870309 6870448 6871349 6871405 6871556 6872398 6872858 6886971 6908402 6908447 6918378 6953591 6954170 6954247 6969745 6974849 6975071 6981957 6982295 6982911 6986115 6990032 7005419 7007344 7007392 7009241 7010029 7012743 7014084 7015766 7044231 7102262 7125334 7176491
973 975 990 1001 1004 1007 1008 1010 1013 1016 1022 1026 1034 1039 1040 1042 1043 1045 1046 1047 1048 1050 1051 1052 1053 1054 1055 1056 1057 1059 1060 1061 1062 1063 1064 1065 1066 1068 1069 1070 1072 1073 1074 1075 1076 1077 1078 1079 1080 1083 1086 1087 1088 1089 1090 1091 1092 1093 1094 1095 1099 1101 1105

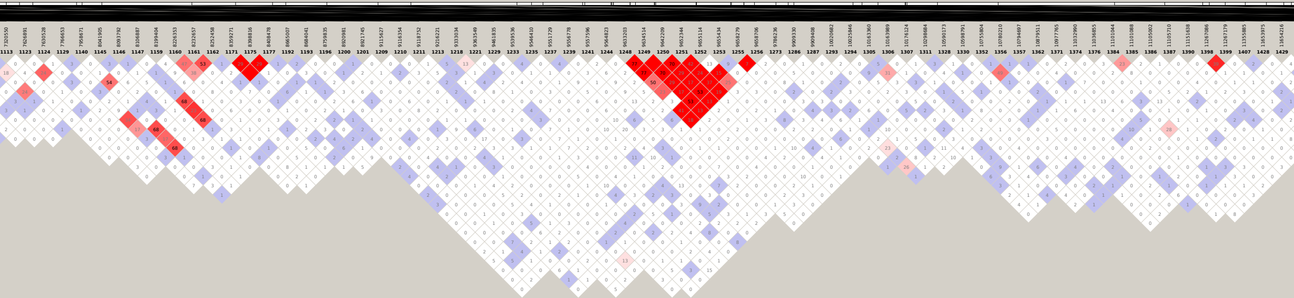

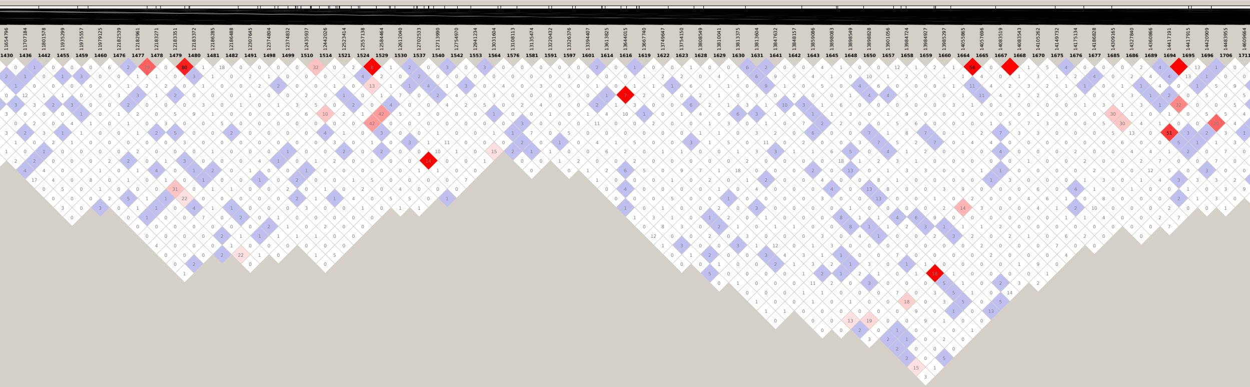

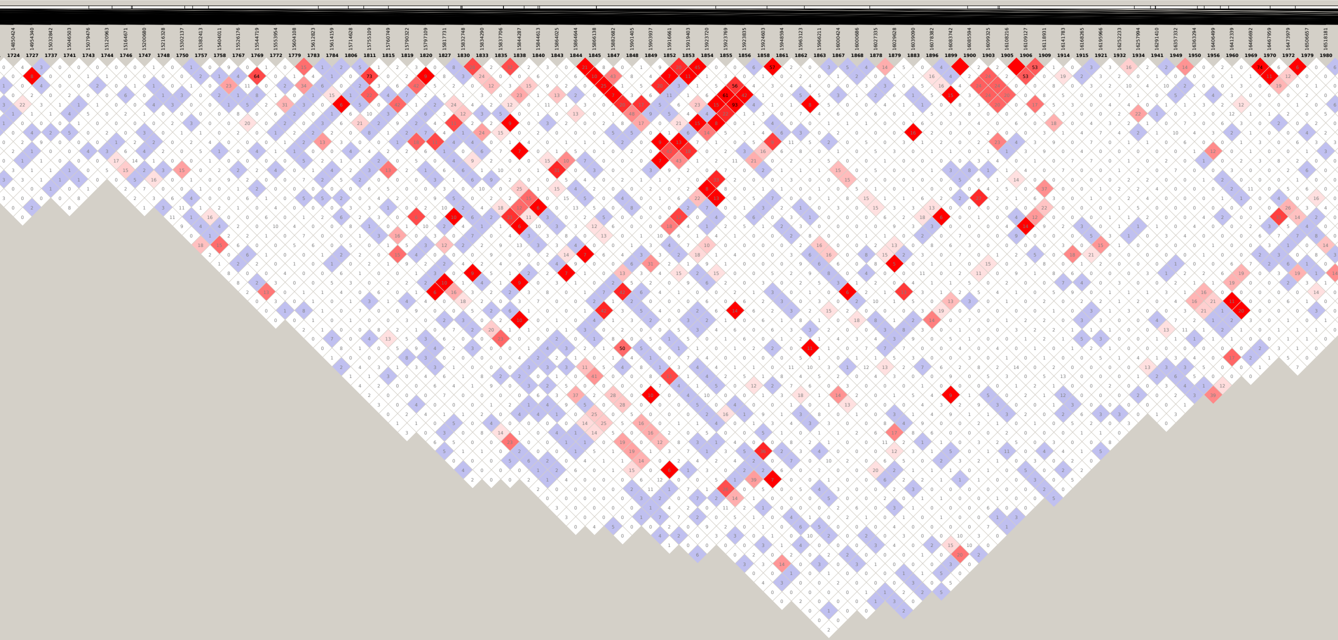

14850424
14954340
15032842
15046503
15079476
15120963
15164671
15200680
15216328
15302137
15382413
15404011
15526176
15544719
15553954
15604108
15612823
15614159
15714628
15755109
15760749
15790322
15797109
15817731
15832748
15834290
15837706
15844287
15844613
15864025
15864644
15866138
15882682
15901405
15903937
15916661
15919403
15923720
15923769
15923835
15924603
15948594
15963123
15966211
16000424
16000686
16027335
16029628
16030090
16078382
16083742
16085594
16099325
16108216
16109127
16118931
16141783
16168265
16195966
16252233
16257994
16291410
16357332
16363294
16406499
16412339
16466692
16467959
16473979
16506657
16518181
1724 1727 1737 1741 1743 1744 1746 1747 1748 1750 1757 1758 1767 1769 1772 1779 1783 1784 1806 1811 1815 1819 1820 1827 1830 1833 1835 1838 1840 1843 1844 1845 1847 1848 1849 1852 1853 1854 1855 1856 1858 1861 1862 1863 1867 1868 1878 1879 1883 1896 1899 1900 1903 1905 1906 1909 1914 1915 1921 1932 1934 1941 1949 1950 1956 1960 1969 1970 1972 1979 1980

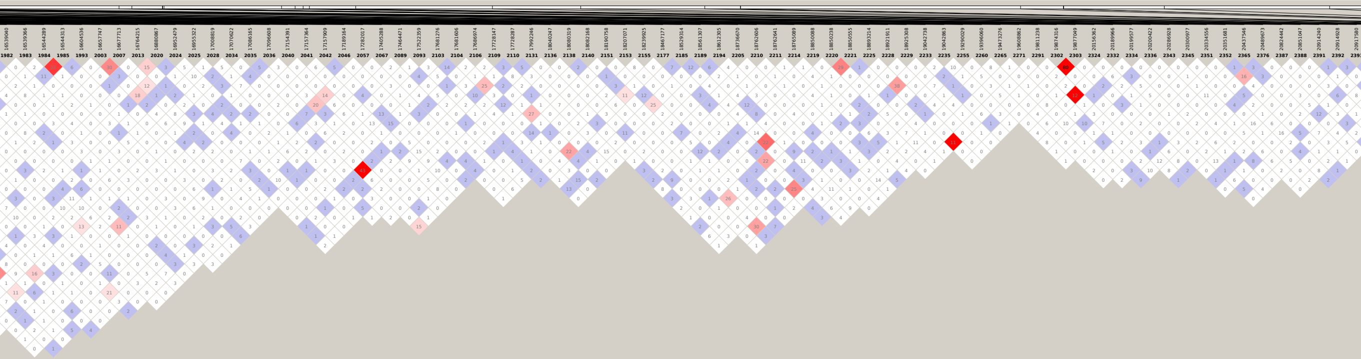

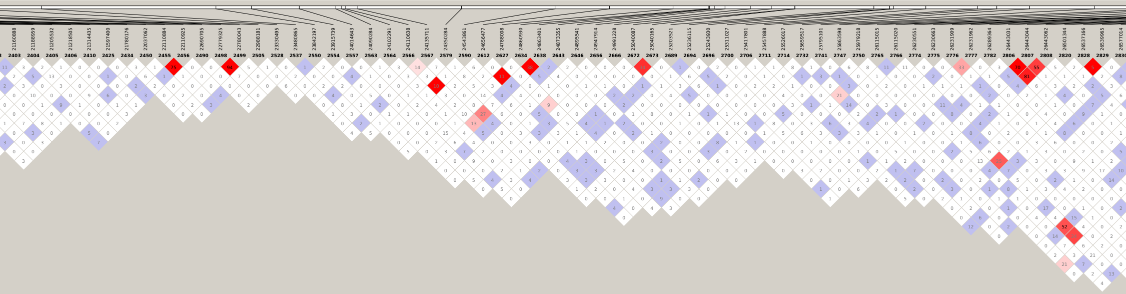

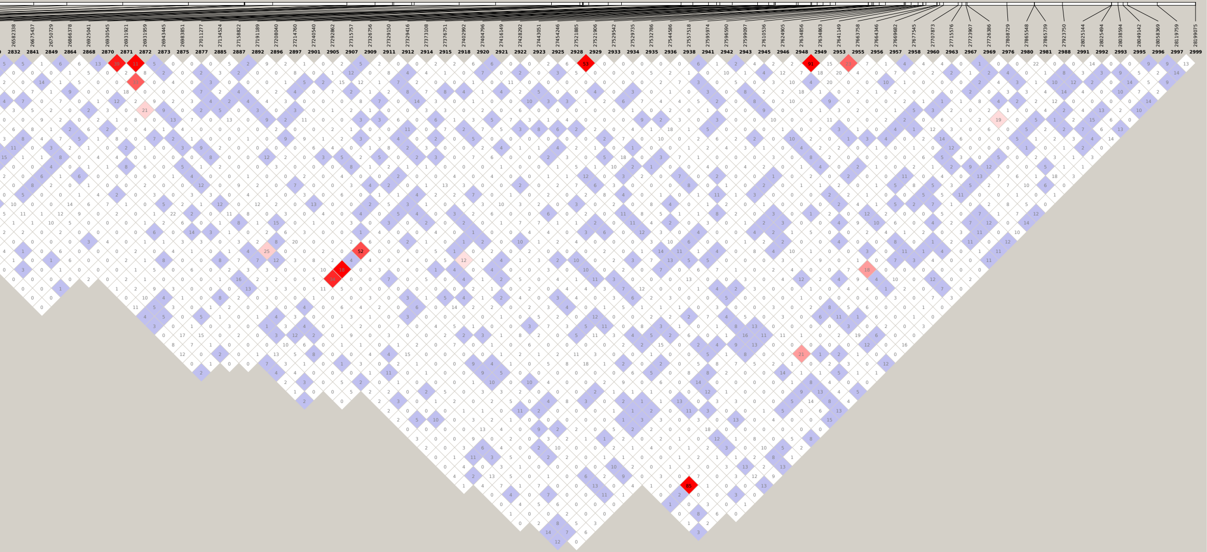
2832 2841 2849 2864 2868 2870 2871 2872 2873 2875 2877 2885 2887 2892 2896 2897 2901 2905 2907 2909 2911 2912 2914 2915 2918 2920 2921 2922 2923 2925 2928 2929 2933 2934 2935 2936 2938 2941 2942 2943 2945 2946 2948 2949 2953 2955 2956 2957 2958 2960 2963 2967 2969 2976 2980 2981 2988 2991 2992 2993 2995 2996 2997 2999

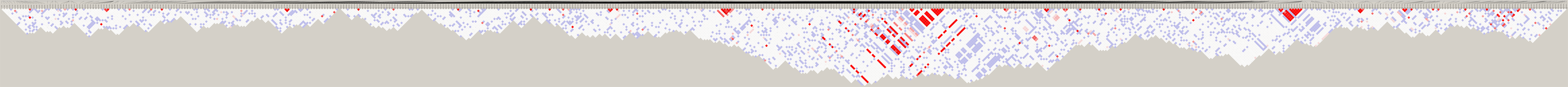

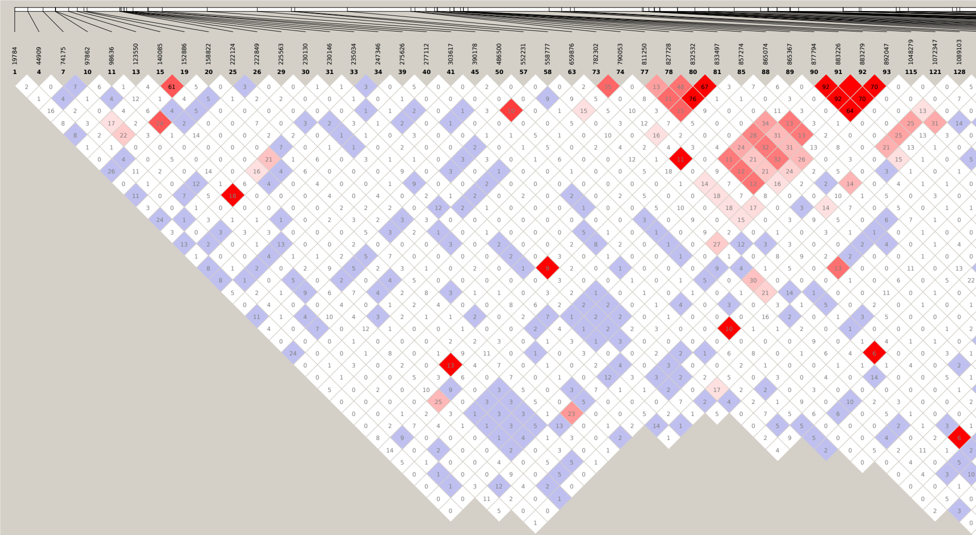
19784 44909 74175 97862 98636 123550 140085 152886 158822 222124 222849 225563 230130 230146 235034 247346 275626 277112 303617 390178 486500 552231 558777 659876 782302 790053 811250 827728 832532 833497 857274 865074 865367 877794 883226 883279 892047 1048279 1072347 1089103
1 4 7 10 11 13 15 19 20 25 26 29 30 31 33 34 39 40 41 45 50 57 58 63 73 74 77 78 80 81 85 88 89 90 91 92 93 115 121 128

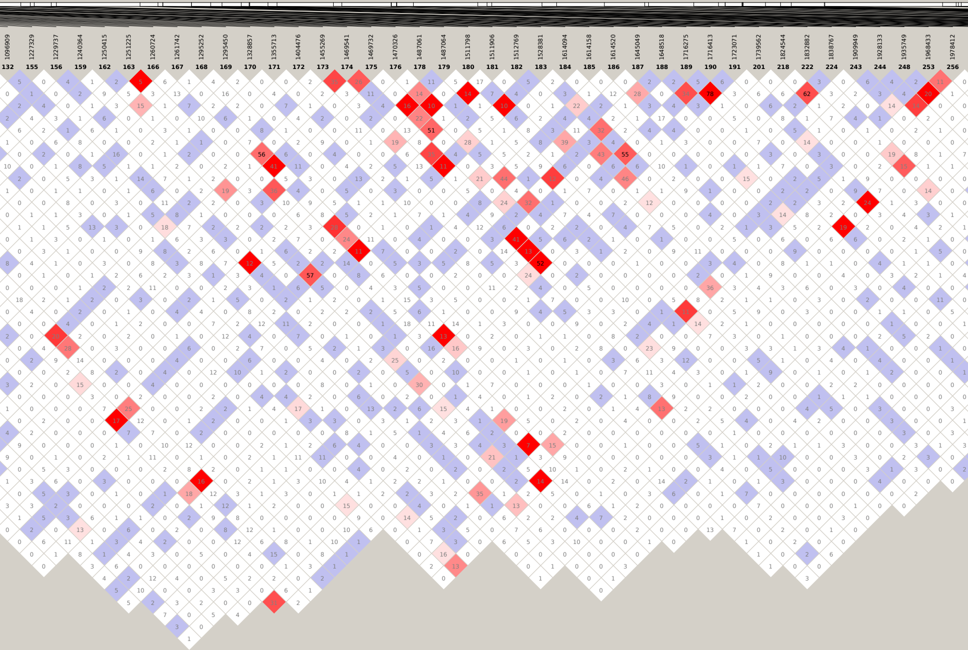
1096909
1227329
1229737
1240364
1250415
1251225
1260724
1261742
1295252
1295450
1328857
1355713
1404476
1455269
1469541
1469732
1470326
1487061
1487064
1511798
1511906
1512769
1528381
1614094
1614158
1614520
1645049
1648518
1716275
1716413
1723071
1739562
1824544
1832882
1838767
1909949
1928133
1935749
1968433
1978412
132 155 156 159 162 163 166 167 168 169 170 171 172 173 174 175 176 178 179 180 181 182 183 184 185 186 187 188 189 190 191 201 218 222 224 243 244 248 253 256

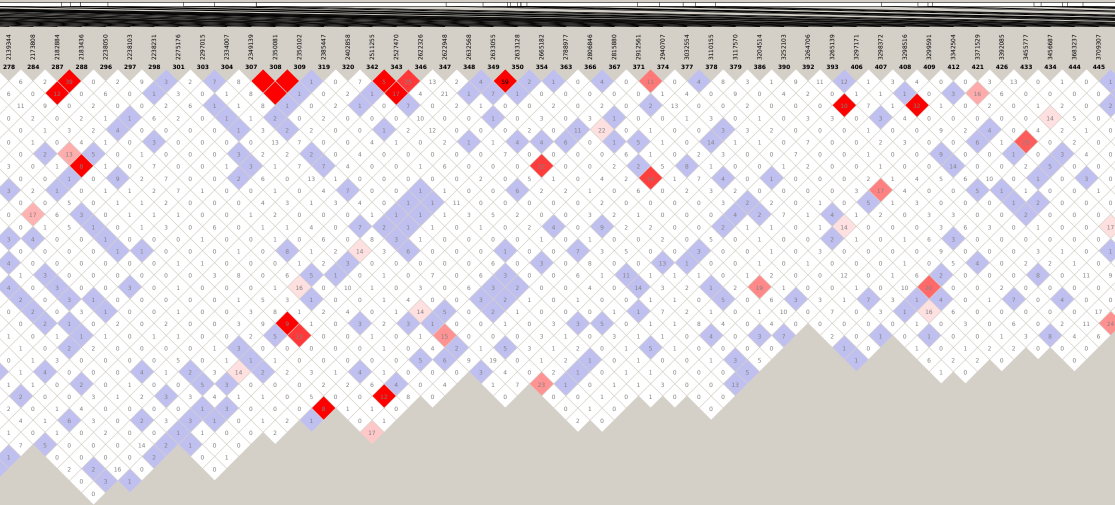

2139344 278
2173808 284
2182884 287
2183436 288
2238050 296
2238103 297
2238231 298
2275176 301
2297015 303
2334007 304
2349139 307
2350081 308
2350102 309
2385447 319
2402858 320
2511255 342
2527470 343
2622326 346
2622948 347
2632568 348
2633055 349
2633128 350
2665182 354
2788977 363
2806846 366
2815880 367
2912561 371
2940707 374
3032554 377
3110155 378
3117570 379
3204514 386
3252103 390
3264706 392
3265139 393
3297171 406
3298372 407
3298516 408
3299591 409
3342504 412
3371529 421
3392085 426
3455777 433
3456687 434
3683237 444
3709307 445

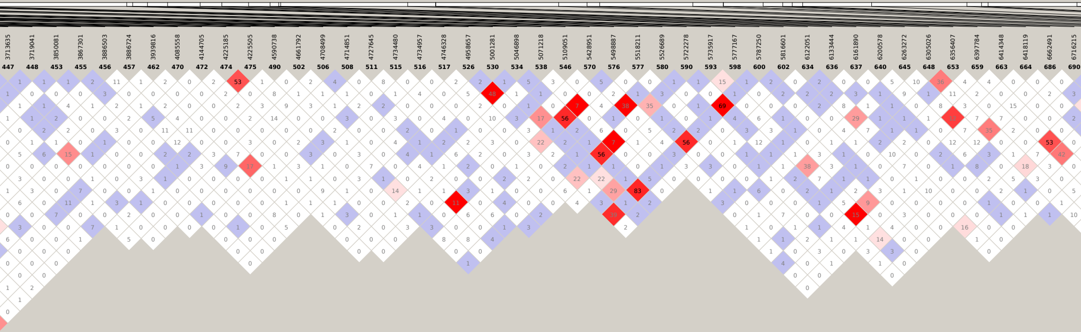

3713635 447
3719041 448
3850081 453
3867301 455
3886503 456
3886724 457
3939816 462
4085558 470
4144705 472
4225185 474
4225505 475
4590738 490
4661792 502
4708499 506
4714851 508
4727645 511
4734480 515
4734957 516
4746328 517
4958657 526
5001281 530
5046898 534
5071218 538
5109051 546
5428951 570
5498887 576
5518211 577
5526689 580
5722278 590
5735917 593
5777167 598
5787250 600
5816601 602
6122051 634
6133444 636
6161890 637
6200578 640
6263272 645
6305026 648
6356407 653
6397784 659
6414348 663
6418119 664
6662491 686
6716215 690

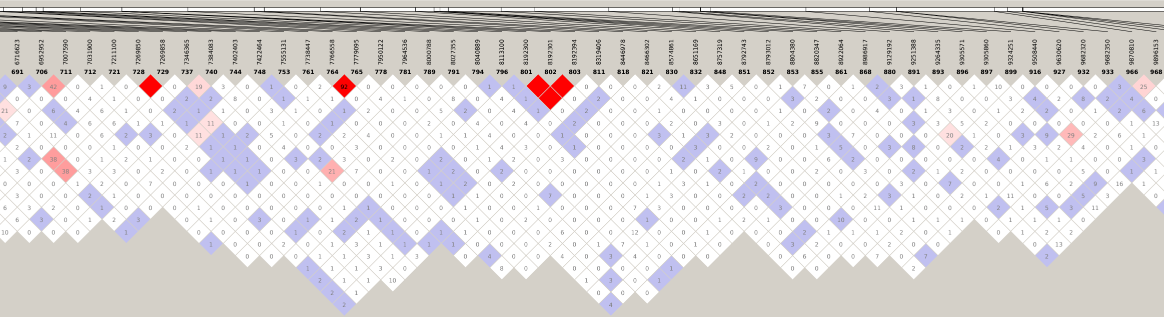
6716623 6952952 7007590 7031900 7211100 7269850 7269858 7346365 7384083 7402403 7422464 7555131 7738447 7766558 7779095 7950122 7964536 8000788 8027355 8040889 8113100 8192300 8192301 8192394 8319406 8446978 8466302 8574861 8651169 8757319 8792743 8793012 8804380 8820347 8922064 8986917 9129192 9251388 9264335 9305571 9305860 9324251 9508440 9630620 9682320 9682350 9870810 9896153
691 706 711 712 721 728 729 737 740 744 748 753 761 764 765 778 781 789 791 794 796 801 802 803 811 818 821 830 832 848 851 852 853 855 861 868 880 891 893 896 897 899 916 927 932 933 966 968

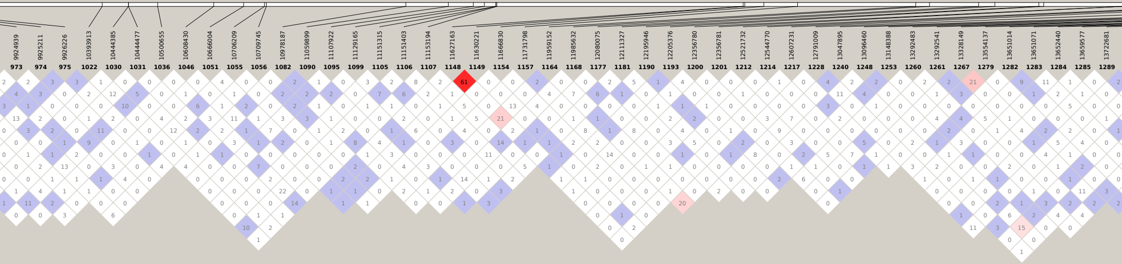
9924939
9925211
9926226
10393913
10444385
10444477
10500655
10608430
10666004
10706209
10709745
10978187
11059899
11107922
11129165
11151315
11151403
11153194
11627163
11630221
11666830
11731798
11959152
11985632
12080075
12111327
12195946
12205376
12356780
12356781
12521732
12544770
12607231
12791009
13047895
13096460
13148388
13292483
13292541
13328149
13554137
13651014
13651071
13652440
13659577
13722681
973 974 975 1022 1030 1031 1036 1046 1051 1055 1056 1082 1090 1095 1099 1105 1106 1107 1148 1149 1154 1157 1164 1168 1177 1181 1190 1193 1200 1201 1212 1214 1217 1228 1240 1248 1253 1260 1261 1267 1279 1282 1283 1284 1285 1289

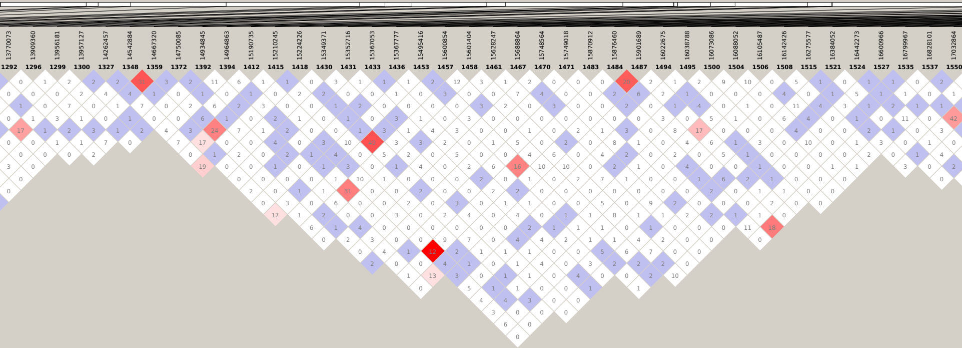
13770073 1292
13909360 1296
13956181 1299
13957127 1300
14262457 1327
14542884 1348
14667320 1359
14750085 1372
14934845 1392
14964863 1394
15190735 1412
15210245 1415
15224226 1418
15349371 1430
15352716 1431
15367053 1433
15367777 1436
15495416 1453
15600854 1457
15601404 1458
15628247 1461
15688864 1467
15748564 1470
15749018 1471
15870912 1483
15876460 1484
15901689 1487
16022675 1494
16038788 1495
16073086 1500
16088052 1504
16105487 1506
16142426 1508
16275577 1515
16384052 1521
16442273 1524
16600966 1527
16799967 1535
16828101 1537
17032864 1550

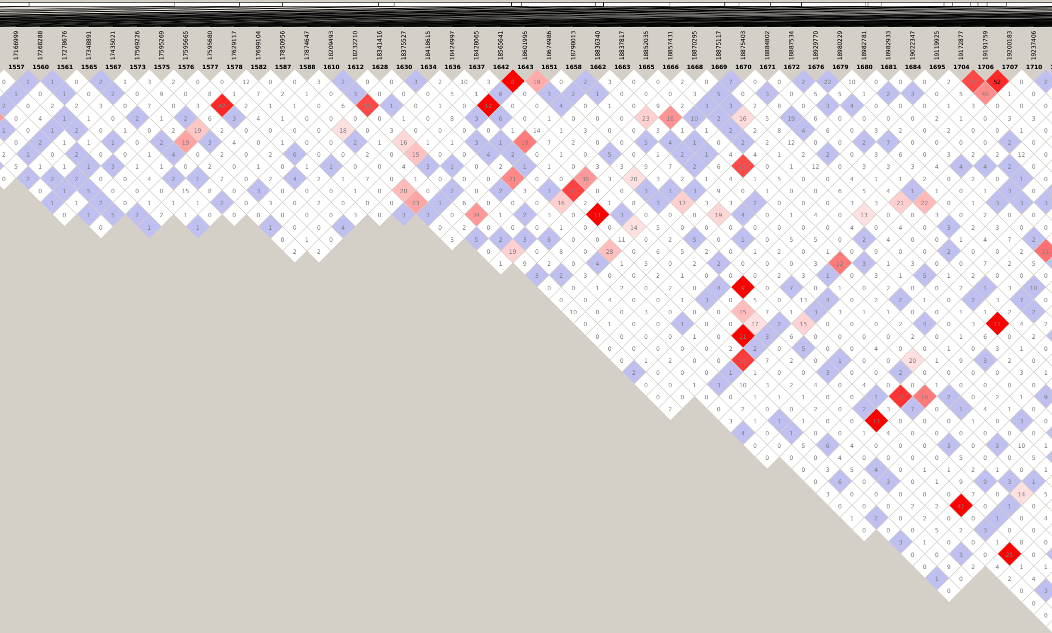
17166999 17268288 17278676 17348891 17435021 17569226 17595269 17595665 17595680 17629117 17699104 17850956 17874647 18209493 18232210 18341416 18375527 18418615 18424997 18428065 18565641 18601995 18674986 18798013 18836340 18837817 18852035 18857431 18870295 18875117 18875403 18884802 18887534 18929770 18980229 18982781 18982933 19022347 19119925 19172877 19191759 19200183 19237406
1557 1560 1561 1565 1567 1573 1575 1576 1577 1578 1582 1587 1588 1610 1612 1628 1630 1634 1636 1637 1642 1643 1651 1658 1662 1663 1665 1666 1668 1669 1670 1671 1672 1676 1679 1680 1681 1684 1695 1704 1706 1707 1710

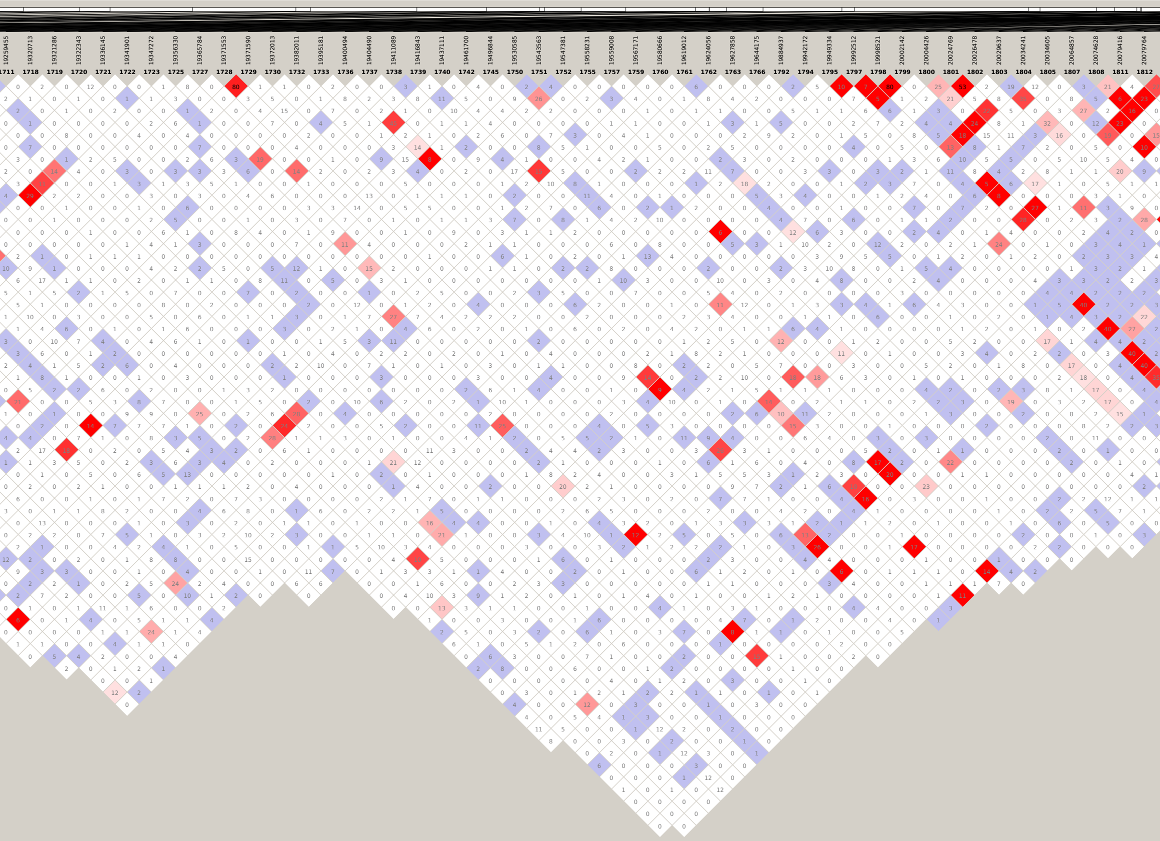

19259455
19320713
19321286
19322343
19336145
19341901
19347272
19356330
19365784
19371553
19371590
19372013
19382011
19395181
19400494
19404490
19411089
19416843
19437111
19461700
19496844
19530585
19543563
19547381
19558231
19559008
19567171
19580666
19619012
19624056
19627858
19644175
19884937
19942172
19949334
19992512
19998521
20002142
20004426
20024769
20026478
20029637
20034241
20034605
20064857
20074628
20079416
20079764
1711
1718
1719
1720
1721
1722
1723
1725
1727
1728
1729
1730
1732
1733
1736
1737
1738
1739
1740
1742
1745
1750
1751
1752
1755
1757
1759
1760
1761
1762
1763
1766
1792
1794
1795
1797
1798
1799
1800
1801
1802
1803
1804
1805
1807
1808
1811
1812

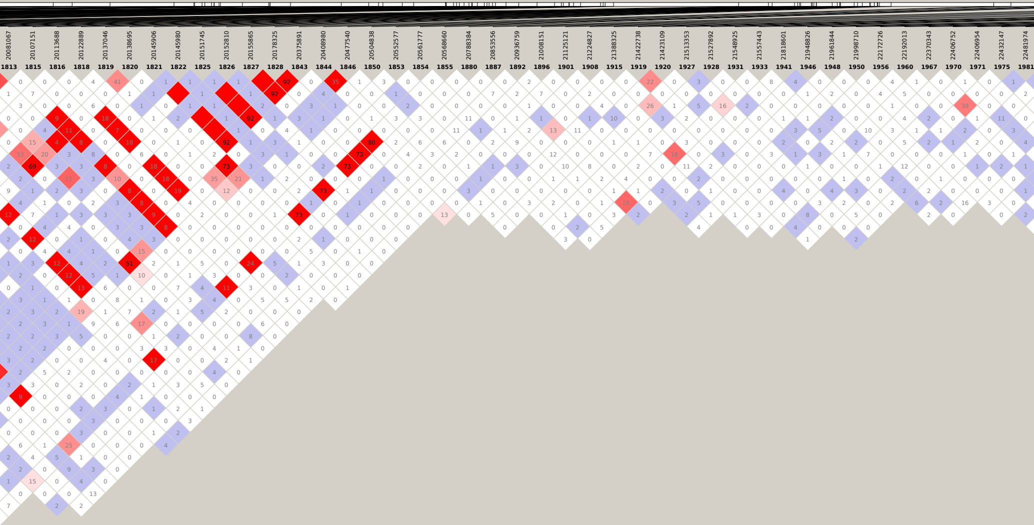
20081067 1813
20107151 1815
20113688 1816
20122889 1818
20137046 1819
20138695 1820
20145906 1821
20145980 1822
20151745 1825
20152810 1826
20155865 1827
20178325 1828
20375891 1843
20408980 1844
20477540 1846
20504838 1850
20552577 1853
20561777 1854
20568660 1855
20788384 1880
20853556 1887
20936759 1892
21008151 1896
21125121 1901
21224827 1908
21388325 1915
21422738 1919
21423109 1920
21513353 1927
21527892 1928
21548925 1931
21557443 1933
21818601 1941
21948826 1946
21961844 1948
21998710 1950
22172726 1956
22192013 1960
22370343 1967
22406752 1970
22406954 1971
22432147 1975
22481974 1981

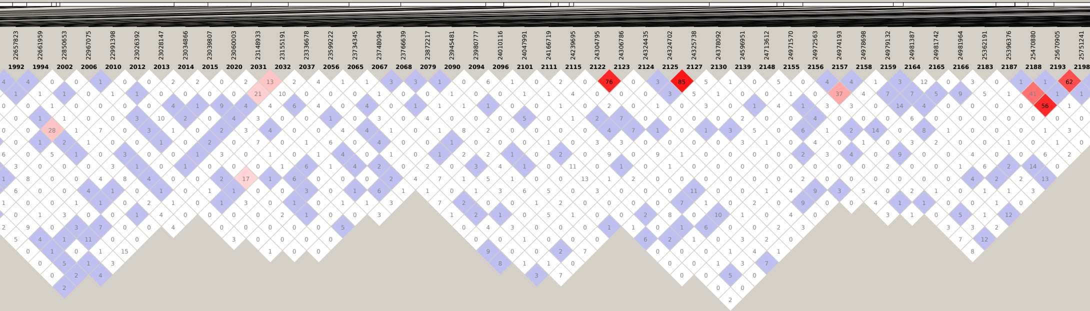

22657823
22661959
22850653
22967075
22991398
23026392
23028147
23034866
23039807
23060003
23148933
23155191
23336678
23599222
23734345
23748094
23766639
23872217
23945481
23980777
24010116
24047991
24166719
24239695
24304795
24306786
24324435
24324702
24325738
24378092
24596951
24713612
24971570
24972563
24974193
24978698
24979132
24981387
24981742
24981964
25362191
25396376
25470880
25670905
25751241
1992 1994 2002 2006 2010 2012 2013 2014 2015 2020 2031 2032 2037 2056 2065 2067 2068 2079 2090 2094 2096 2101 2111 2115 2122 2123 2124 2125 2127 2130 2139 2148 2155 2156 2157 2158 2159 2164 2165 2166 2183 2187 2188 2193 2198

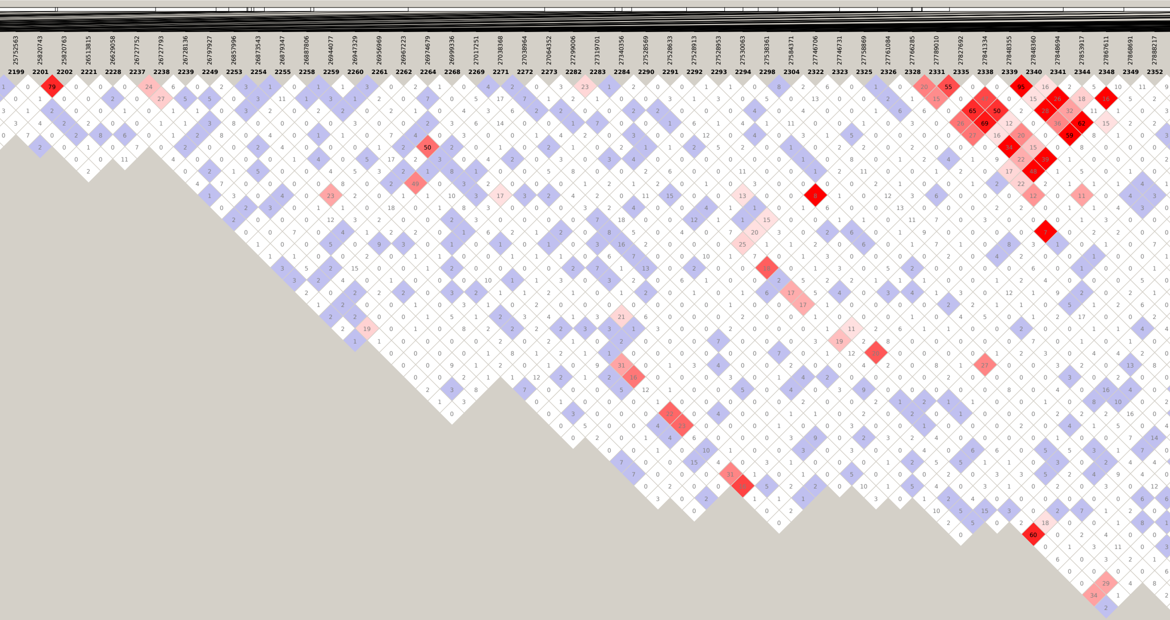

25752563 2199
25820743 2201
25820763 2202
26513815 2221
26629058 2228
26727752 2237
26727793 2238
26728136 2239
26797927 2249
26857996 2253
26873543 2254
26879347 2255
26887806 2258
26944077 2259
26947329 2260
26956969 2261
26967223 2262
26974679 2264
26999336 2268
27017251 2269
27038368 2271
27038964 2272
27064352 2273
27299006 2282
27319701 2283
27340356 2284
27528569 2290
27528633 2291
27528913 2292
27528953 2293
27530063 2294
27538361 2298
27584371 2304
27746706 2322
27746731 2323
27758869 2325
27761084 2326
27766285 2328
27789010 2331
27827692 2335
27841334 2338
27848355 2339
27848360 2340
27848694 2341
27853917 2344
27867611 2348
27868691 2349
27888217 2352

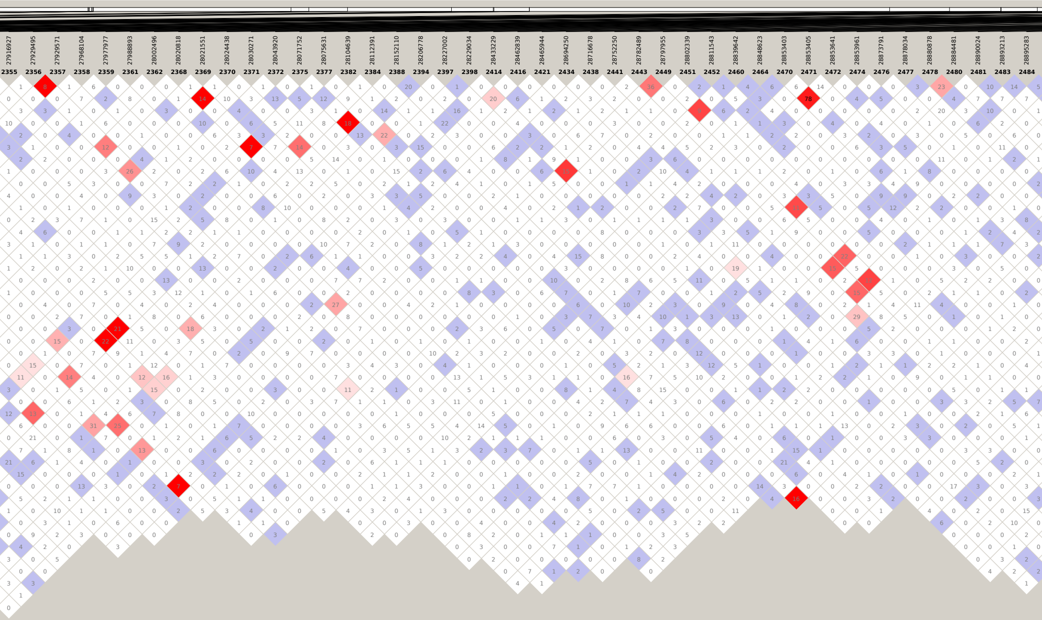
27916927 2355
27929495 2356
27929571 2357
27968104 2358
27977977 2359
27988893 2361
28002496 2362
28020818 2368
28021551 2369
28024438 2370
28030271 2371
28043920 2372
28071752 2375
28075631 2377
28104639 2382
28112391 2384
28152110 2388
28206778 2394
28227002 2397
28229034 2398
28433229 2414
28462839 2416
28465944 2421
28694250 2434
28716678 2438
28752250 2441
28782489 2443
28797955 2449
28802339 2451
28811543 2452
28839642 2460
28848623 2464
28853403 2470
28853405 2471
28853641 2472
28853961 2474
28873791 2476
28878034 2477
28880878 2478
28884481 2480
28890024 2481
28893213 2483
28895283 2484

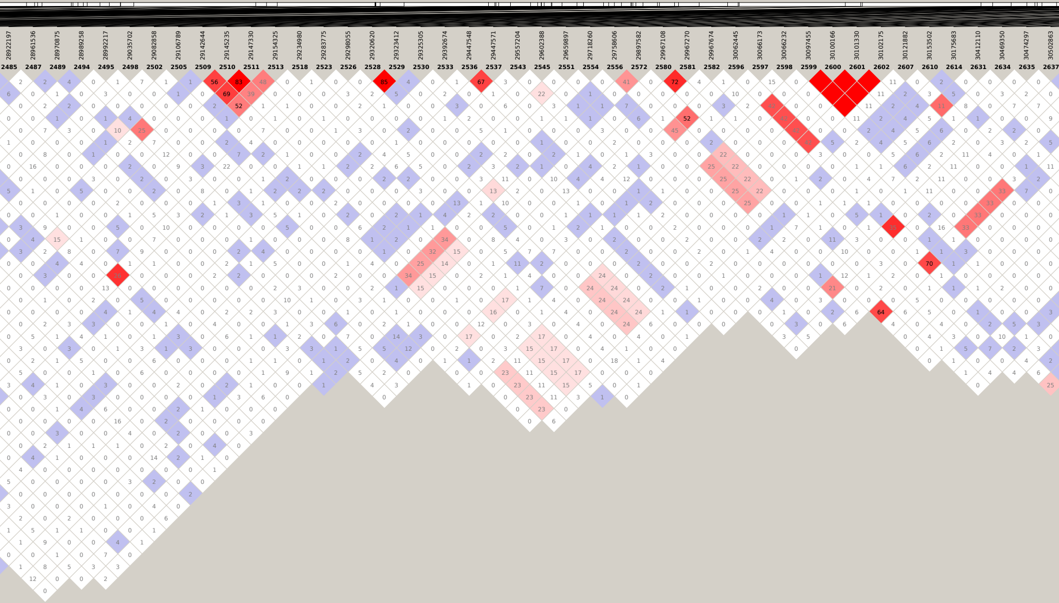
28922197 28961536 28970875 28989258 28992217 29035702 29082858 29106789 29142644 29145235 29147330 29154325 29234980 29283775 29298055 29320620 29323412 29325305 29392674 29447548 29447571 29557204 29602388 29659897 29718260 29758606 29897582 29967108 29967270 29967674 30062445 30066173 30066232 30097455 30100166 30101330 30102175 30121882 30153502 30175683 30412110 30469350 30474297 30502863
2485 2487 2489 2494 2495 2498 2502 2505 2509 2510 2511 2513 2518 2523 2526 2528 2529 2530 2533 2536 2537 2543 2545 2551 2554 2556 2572 2580 2581 2582 2596 2597 2598 2599 2600 2601 2602 2607 2610 2614 2631 2634 2635 2637

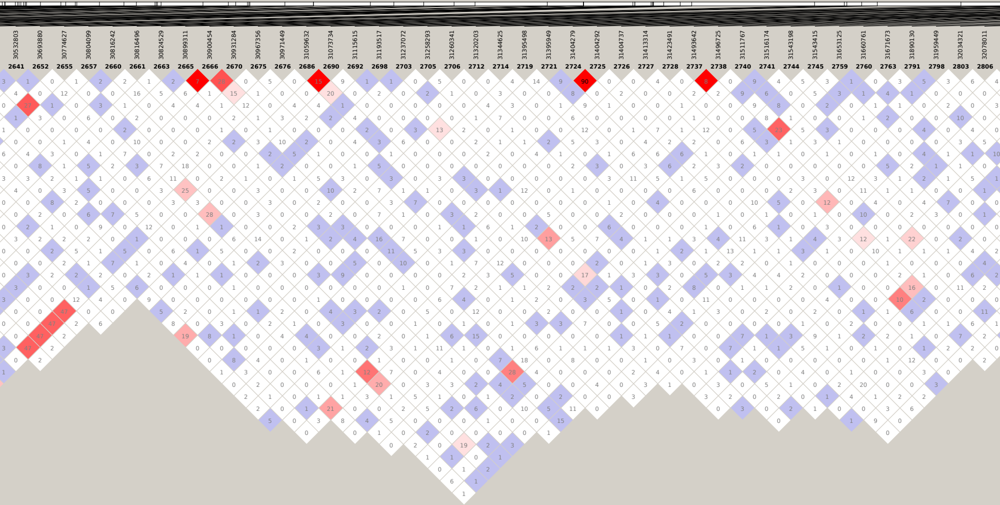
30532803
30693880
30774627
30804099
30816242
30816496
30824529
30899311
30900454
30931284
30967356
30971449
31059632
31073734
31115615
31193517
31237072
31258293
31260341
31320203
31344625
31395498
31395949
31404279
31404292
31404737
31413314
31423491
31493642
31496725
31511767
31516174
31543198
31543415
31653125
31660761
31671673
31890130
31959449
32034321
32078011
2641 2652 2655 2657 2660 2661 2663 2665 2666 2670 2675 2676 2686 2690 2692 2698 2703 2705 2706 2712 2714 2719 2721 2724 2725 2726 2727 2728 2737 2738 2740 2741 2744 2745 2759 2760 2763 2791 2798 2803 2806

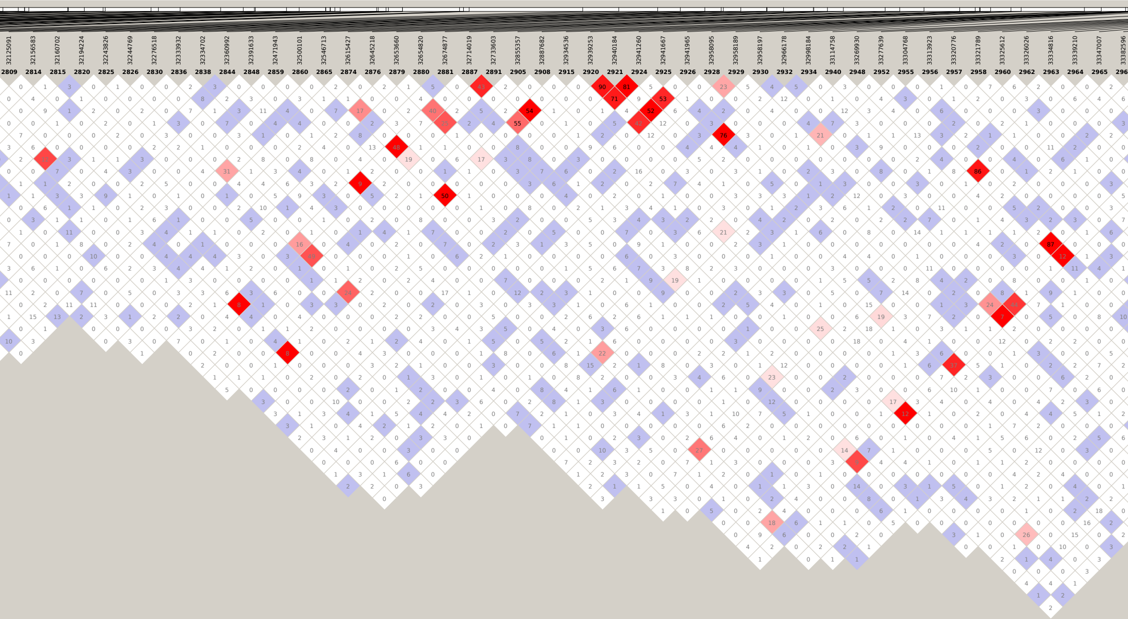
32125091 2809
32156583 2814
32160702 2815
32194224 2820
32243826 2825
32244769 2826
32276518 2830
32333932 2836
32334702 2838
32360992 2844
32391633 2848
32471943 2859
32500101 2860
32546713 2865
32615427 2874
32645218 2876
32653660 2879
32654820 2880
32674877 2881
32714019 2887
32733603 2891
32855357 2905
32887682 2908
32934536 2915
32939253 2920
32940184 2921
32941260 2924
32941667 2925
32941965 2926
32958095 2928
32958189 2929
32958197 2930
32966178 2932
32998184 2934
33114758 2940
33269930 2948
33277639 2952
33304768 2955
33313923 2956
33320776 2957
33321789 2958
33325612 2960
33326026 2962
33334816 2963
33339210 2964
33347007 2965
33382596 296

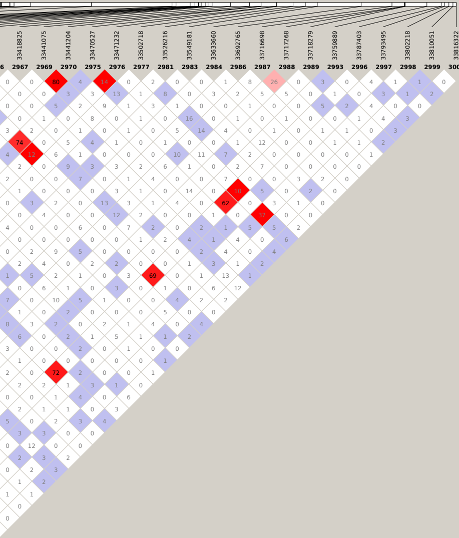

33418825
33441075
33441204
33470527
33471232
33502718
33526216
33549181
33633660
33692765
33716698
33717268
33718279
33759889
33787403
33793495
33802218
33810051
33816322
6 2967 2969 2970 2975 2976 2977 2981 2983 2984 2986 2987 2988 2989 2993 2996 2997 2998 2999 3000

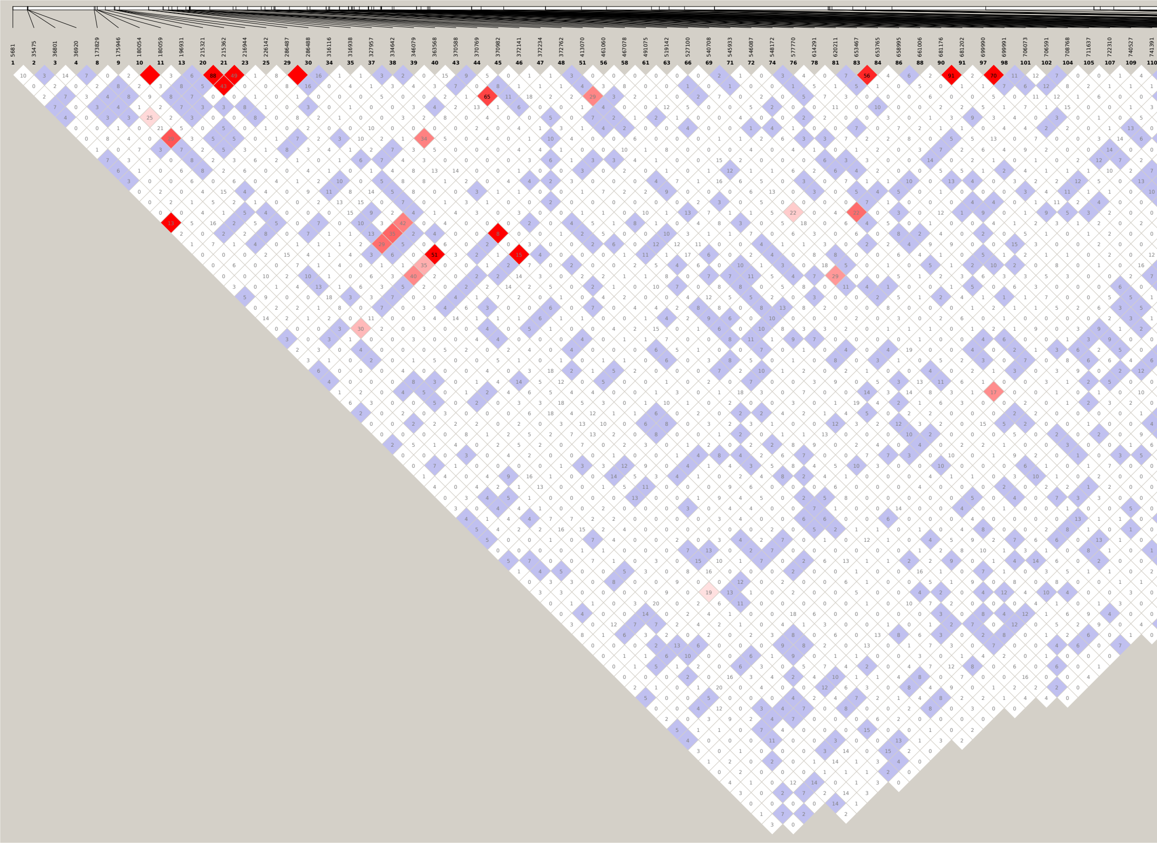
5681 1
35475 2
36801 3
36920 4
173829 8
175946 9
180054 10
180059 11
196931 13
215321 20
215362 21
216944 23
226142 25
286487 29
286488 30
316116 34
316938 35
327957 37
334642 38
346079 39
363568 40
370588 43
370769 44
370982 45
372141 46
372234 47
372762 48
413070 51
461060 56
467078 58
491075 61
519142 63
527100 66
540708 69
545933 71
546087 72
548172 74
577770 76
614291 78
620211 81
653467 83
653765 84
658995 86
661006 88
681176 90
681202 91
699990 97
699991 98
706073 101
706591 102
708768 104
711637 105
722310 107
740527 109
741391 110

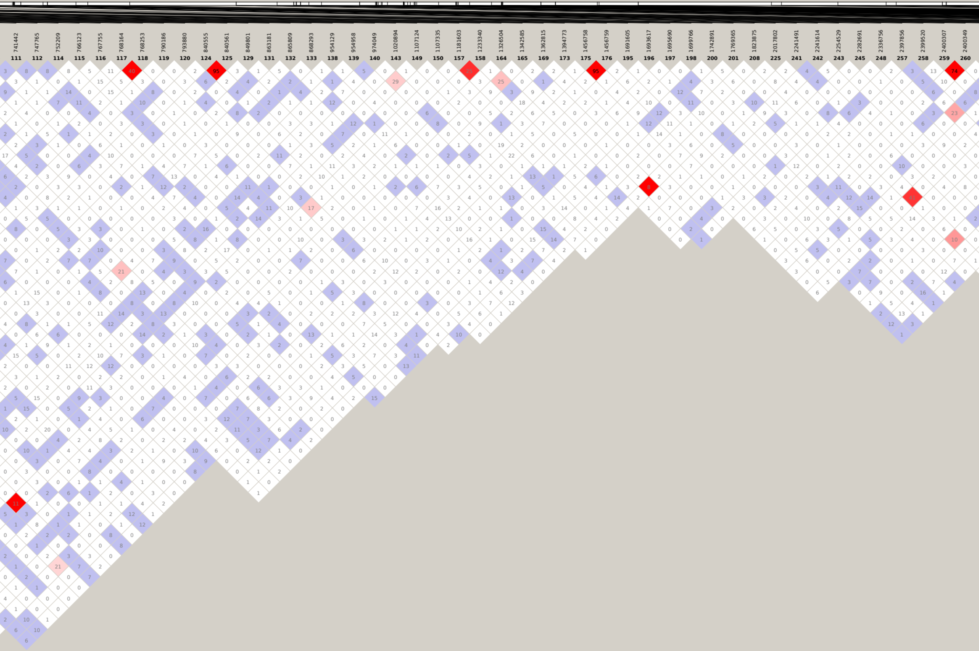
741442 747765 752209 766123 767755 768164 768253 790186 793880 840555 840561 849801 863181 865809 868293 954129 954958 974049 1020894 1107124 1107335 1181603 1233340 1326504 1342585 1362815 1394773 1456758 1456759 1691605 1693617 1695690 1699766 1742891 1769365 1823875 2017802 2241491 2241614 2254529 2282691 2336756 2397856 2399520 2400307 2400349
111 112 114 115 116 117 118 119 120 124 125 129 131 132 133 138 139 140 143 149 150 157 158 164 165 169 173 175 176 195 196 197 198 200 201 208 225 241 242 243 245 248 257 258 259 260

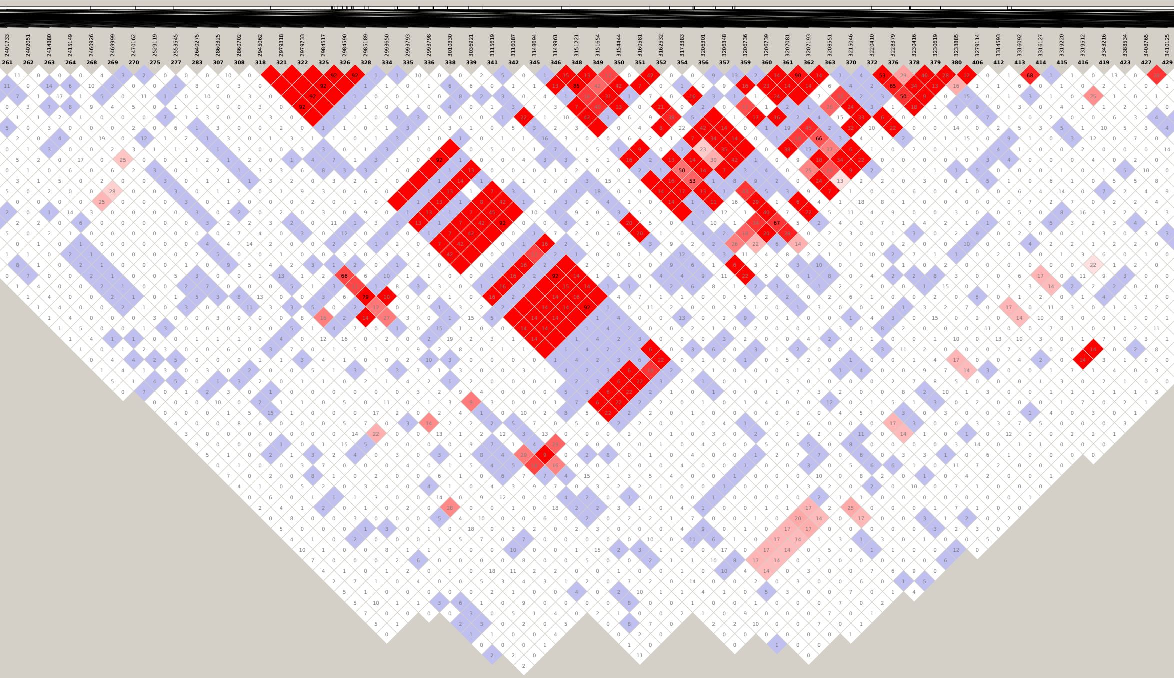
2401733
2402051
2414880
2415149
2460926
2469999
2470162
2529119
2553545
2640275
2860325
2860702
2945062
2979318
2979733
2984517
2984590
2985189
2993650
2993793
2993798
3010830
3036921
3115619
3116087
3148694
3149961
3151221
3151654
3154444
3160581
3162532
3173383
3206301
3206348
3206736
3206739
3207081
3207193
3208551
3215046
3220410
3228379
3230416
3230619
3233885
3279114
3314593
3316092
3316127
3319220
3319512
3343216
3388534
3408765
3410125
261 262 263 264 268 269 270 275 277 283 307 308 318 321 322 325 326 328 334 335 336 338 339 341 342 345 346 348 349 350 351 352 354 356 357 359 360 361 362 363 370 372 376 378 379 380 406 412 413 414 415 416 419 423 427 429

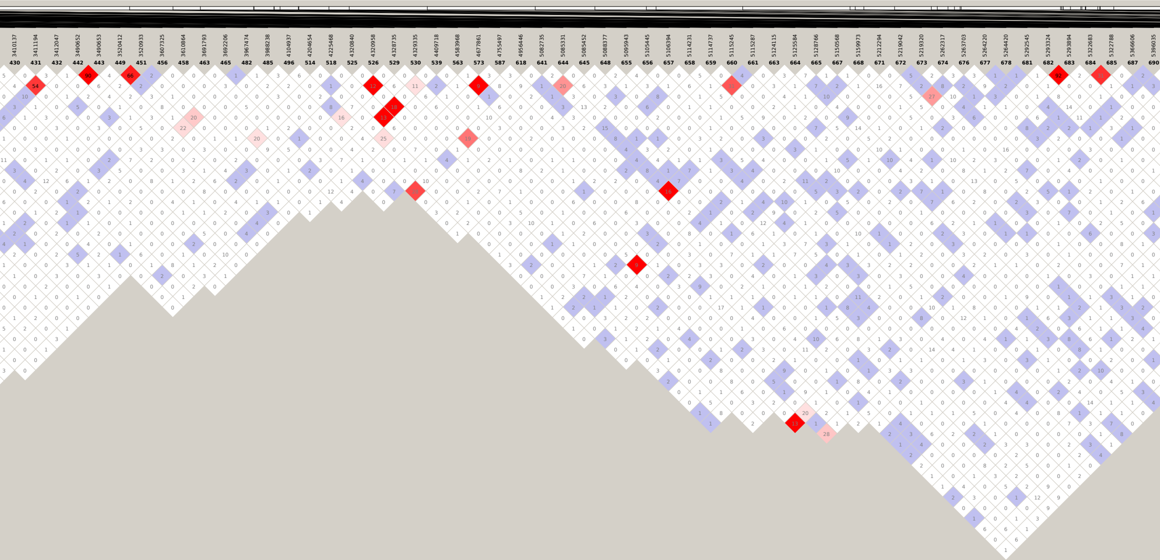
3410137 3411194 3412047 3490652 3490653 3520412 3520933 3607325 3610864 3691793 3692206 3967474 3988238 4104937 4204654 4225468 4320840 4320958 4328735 4329335 4409718 4583968 4677861 4755497 4956446 5082735 5085331 5085452 5088377 5095943 5105445 5106394 5114231 5114737 5115245 5115287 5124115 5125584 5128766 5150568 5159973 5212294 5219042 5219320 5262317 5263703 5264220 5264420 5292545 5293324 5293894 5322683 5322788 5366606 5396035
430 431 432 442 443 449 451 456 458 463 465 482 485 496 514 518 525 526 529 530 539 563 573 587 618 641 644 645 648 655 656 657 658 659 660 661 663 664 665 667 668 671 672 673 674 676 677 678 681 682 683 684 685 687 690

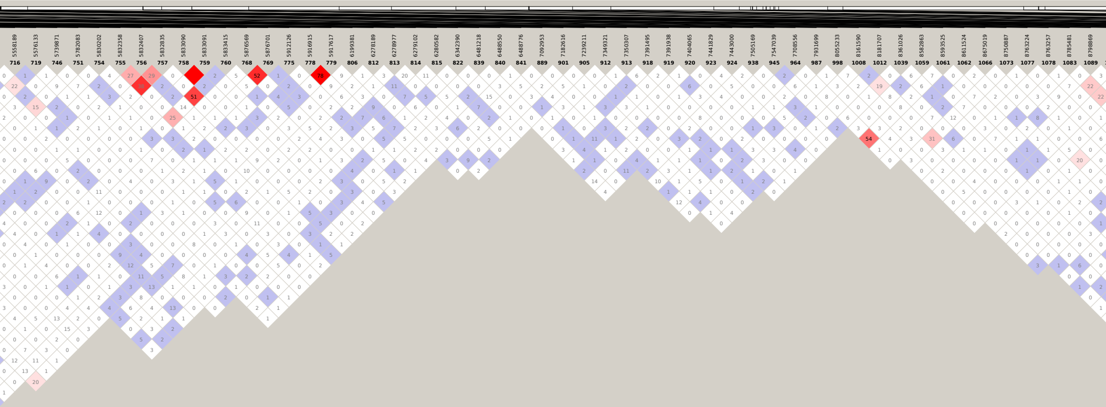

5558189 5576133 5739871 5782083 5830202 5832358 5832407 5832835 5833090 5833091 5833415 5876569 5876701 5912126 5916915 5917617 6199381 6278189 6278977 6279102 6280582 6342390 6481218 6488550 6488776 7092953 7182616 7239211 7349321 7350307 7391495 7391938 7404065 7441829 7443000 7505169 7547039 7708856 7931699 8055233 8161590 8181707 8361026 8582863 8593525 8611524 8675019 8750887 8763224 8763257 8785481 8798869
716 719 746 751 754 755 756 757 758 759 760 768 769 775 778 779 806 812 813 814 815 822 839 840 841 889 901 905 912 913 918 919 920 923 924 938 945 964 987 998 1008 1012 1039 1059 1061 1062 1066 1073 1077 1078 1083 1089

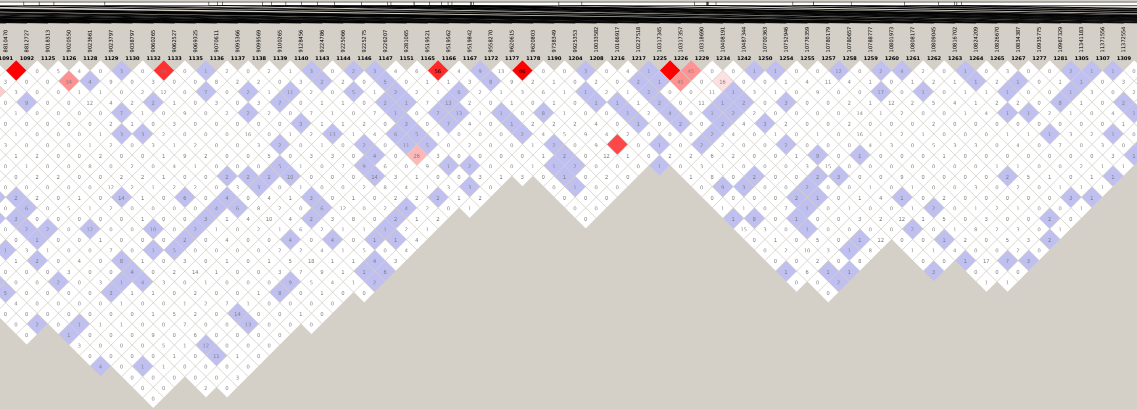
8810470 8812727 9018313 9020550 9023661 9023797 9038797 9060265 9062527 9069325 9070611 9095366 9099569 9100265 9128456 9224786 9225066 9225275 9226207 9281065 9519521 9519562 9519842 9558270 9620615 9620803 9738349 9925353 10033582 10166917 10227518 10317345 10317357 10318690 10408191 10487344 10700363 10752946 10776359 10780179 10780657 10788777 10801973 10808177 10809045 10816702 10824209 10826670 10834387 10935775 10967329 11341183 11371556 11372554
1091 1092 1125 1126 1128 1129 1130 1132 1133 1135 1136 1137 1138 1139 1140 1143 1144 1146 1147 1151 1165 1166 1167 1172 1177 1178 1190 1204 1208 1216 1217 1225 1226 1229 1234 1242 1250 1254 1255 1257 1258 1259 1260 1261 1262 1263 1264 1265 1267 1277 1281 1305 1307 1309

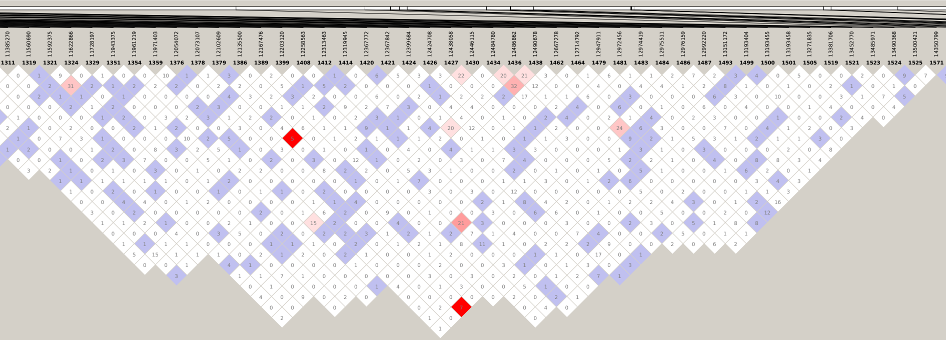
11385270 11560690 11592375 11622866 11728197 11943375 11961219 11971403 12054072 12073107 12102609 12135500 12167476 12203120 12258563 12313463 12319945 12367772 12367842 12399684 12424708 12438058 12446115 12484780 12486862 12490678 12667278 12714792 12947911 12972456 12974419 12975511 12976159 12992220 13151172 13193404 13193455 13193458 13271835 13381706 13452770 13485971 13490368 13500421 14350799
1311 1319 1321 1324 1329 1351 1354 1359 1376 1378 1379 1386 1389 1399 1408 1412 1414 1420 1421 1424 1426 1427 1430 1434 1436 1438 1462 1464 1479 1481 1483 1484 1486 1487 1493 1499 1500 1501 1505 1519 1521 1523 1524 1525 1571

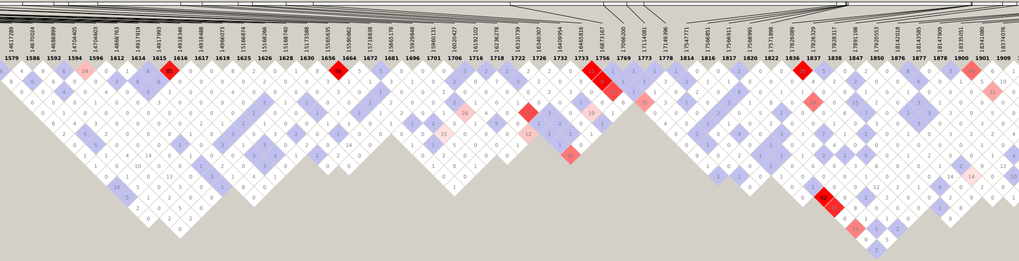
14617289
14670024
14688899
14704405
14704603
14868763
14917919
14917993
14918346
14918488
14966073
15166874
15168266
15168740
15173588
15565635
15580862
15718838
15865178
15929848
15960131
16020427
16192102
16236278
16310739
16340307
16409954
16465816
16873167
17066200
17114081
17149396
17547771
17566851
17566911
17568995
17571898
17826089
17826329
17828317
17891198
17920553
18142018
18143585
18147909
18335051
18341080
18374076
1579
1586
1592
1594
1596
1612
1614
1615
1616
1617
1619
1625
1626
1628
1630
1656
1664
1672
1681
1696
1701
1706
1716
1718
1722
1726
1732
1733
1756
1769
1773
1778
1814
1816
1817
1820
1822
1836
1837
1838
1847
1850
1876
1877
1878
1900
1901
1909

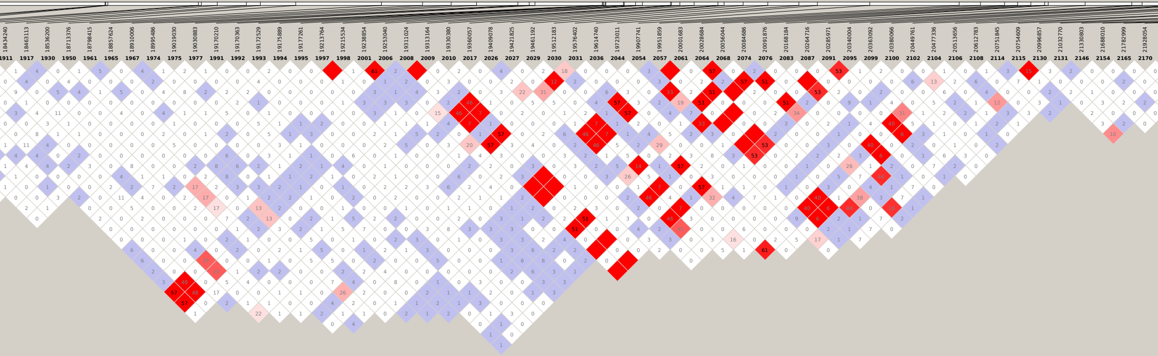
18463113 18536200 18713376 18798415 18857624 18910006 18995486 19019030 19030883 19170210 19170363 19172529 19175889 19177261 19213764 19215534 19238854 19253040 19311024 19313164 19330380 19360057 19409078 19421825 19461192 19512183 19576402 19614740 19723011 19907741 19951859 20001683 20028684 20056044 20084686 20091876 20168184 20264716 20285971 20340004 20363092 20380066 20449761 20477336 20513656 20612783 20751845 20754609 20996857 21032770 21330803 21688010 21782999 21928054
1911 1917 1930 1950 1961 1965 1967 1974 1975 1977 1991 1992 1993 1994 1995 1997 1998 2001 2006 2008 2009 2010 2017 2026 2027 2029 2030 2031 2036 2044 2054 2057 2061 2064 2068 2074 2076 2083 2087 2091 2095 2099 2100 2102 2104 2106 2108 2114 2115 2130 2131 2146 2154 2165 2170

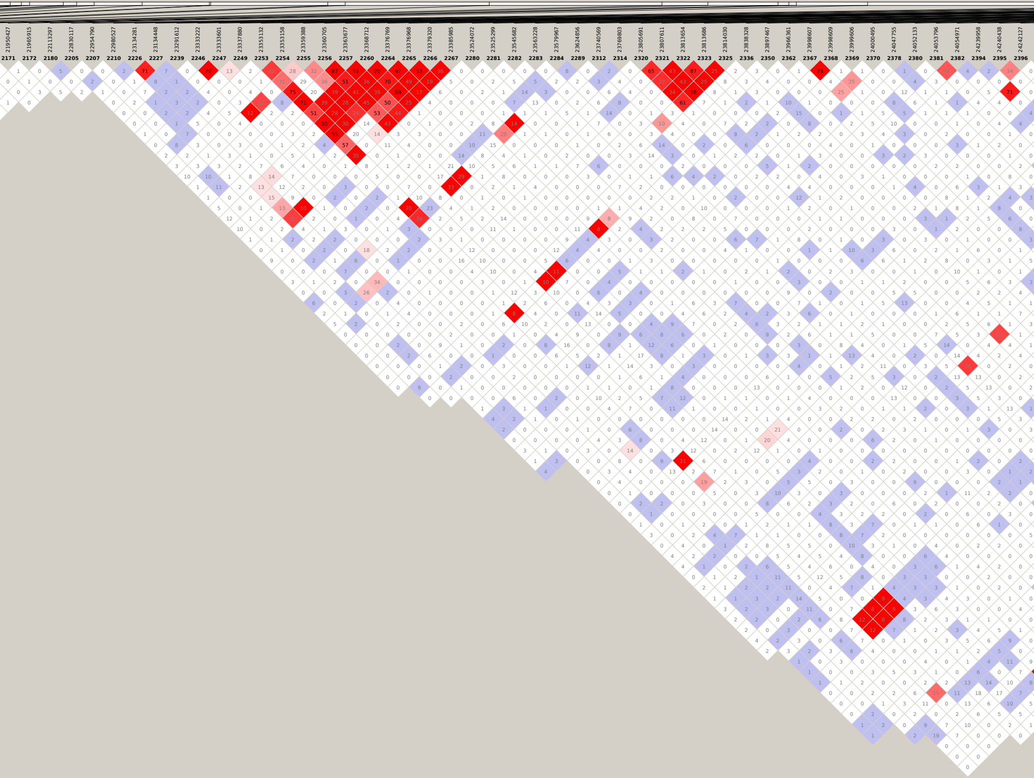
21950427
21965915
22113297
22830117
22954790
22980527
23134281
23134448
23291612
23333222
23333601
23337880
23353132
23353158
23359388
23360705
23363677
23368712
23376769
23376968
23379320
23385985
23524072
23525299
23545682
23563228
23579967
23624856
23740569
23769803
23805691
23807611
23813654
23813686
23814030
23838328
23897467
23966361
23998607
23998609
23999606
24000495
24047755
24052133
24053796
24054971
24239958
24240438
24242127
2171
2172
2180
2205
2207
2210
2226
2227
2239
2246
2247
2249
2253
2254
2255
2256
2257
2260
2264
2265
2266
2267
2280
2281
2282
2283
2284
2289
2312
2314
2320
2321
2322
2323
2325
2336
2350
2362
2367
2368
2369
2370
2378
2380
2381
2382
2394
2395
2396

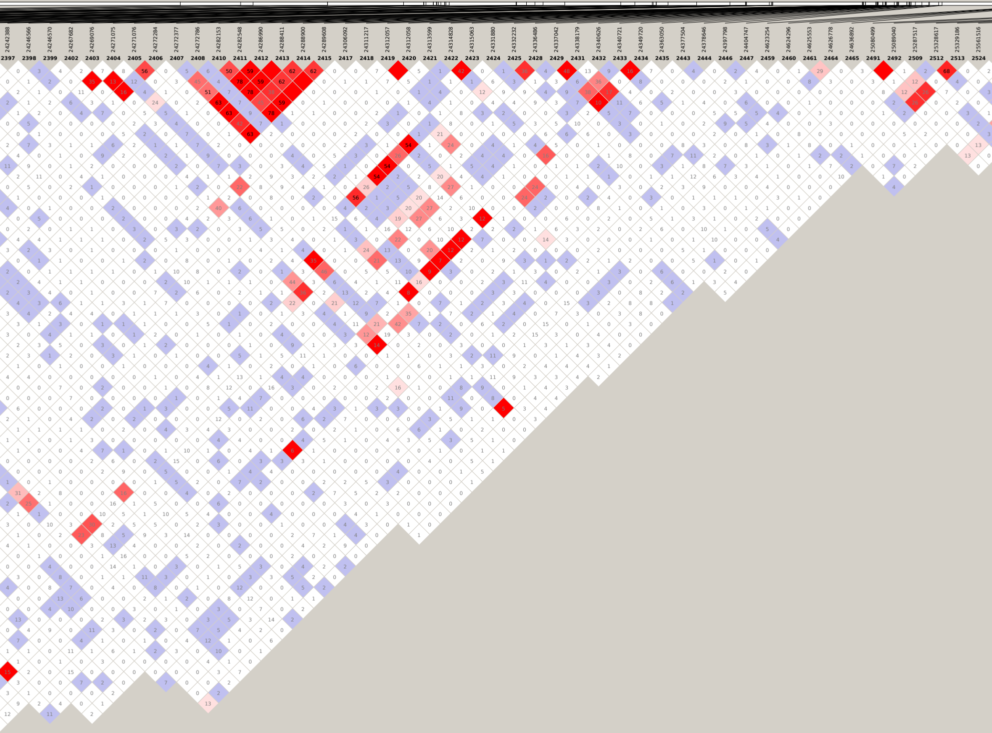
24242388 24246566 24246570 24267682 24269076 24271075 24271076 24272284 24272377 24272786 24282153 24282548 24286990 24288411 24288900 24289608 24306092 24311217 24312057 24312058 24313599 24314828 24315063 24331880 24332232 24336486 24337042 24338179 24340626 24340721 24349720 24363050 24377504 24378646 24397798 24404747 24623254 24624296 24625553 24626778 24636892 25080499 25089040 25287517 25328617 25329186 25561516
2397 2398 2399 2402 2403 2404 2405 2406 2407 2408 2410 2411 2412 2413 2414 2415 2417 2418 2419 2420 2421 2422 2423 2424 2425 2428 2429 2431 2432 2433 2434 2435 2443 2444 2446 2447 2459 2460 2461 2464 2465 2491 2492 2509 2512 2513 2524

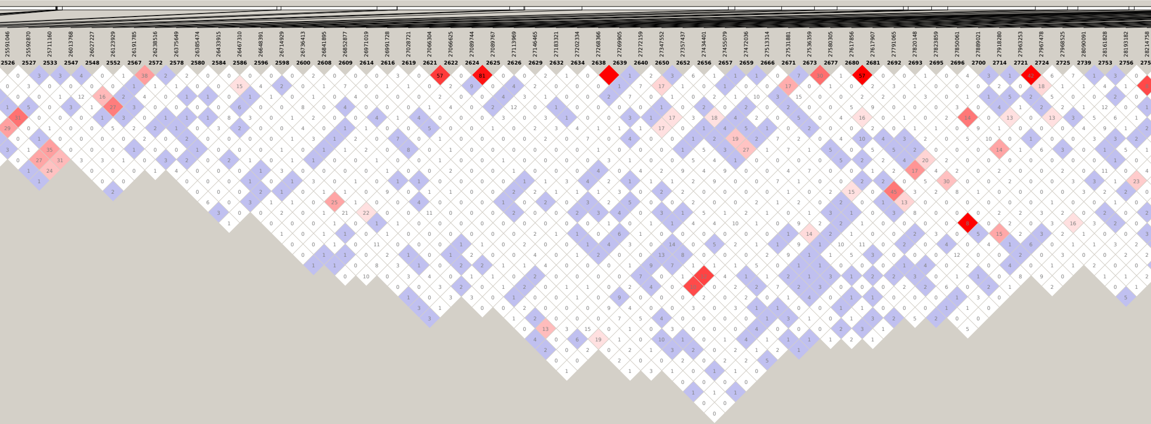
25591046 25592870 25711160 26013768 26027227 26123929 26191785 26238516 26375649 26385474 26433915 26467310 26648391 26714929 26736413 26841895 26852877 26971019 26991728 27028721 27066304 27066625 27089744 27089767 27113969 27146465 27183321 27202334 27268366 27269905 27272159 27347552 27357437 27434401 27455079 27472036 27513314 27531881 27536359 27580305 27617856 27617907 27791065 27820148 27823859 27850061 27889021 27918280 27963253 27967478 27968525 28090091 28161828 28193182 28214758
2526 2527 2533 2547 2548 2552 2567 2572 2578 2580 2584 2586 2596 2598 2600 2608 2609 2614 2616 2619 2621 2622 2624 2625 2626 2629 2632 2634 2638 2639 2640 2650 2652 2656 2657 2659 2666 2671 2673 2677 2680 2681 2692 2693 2695 2696 2700 2714 2721 2724 2725 2739 2753 2756

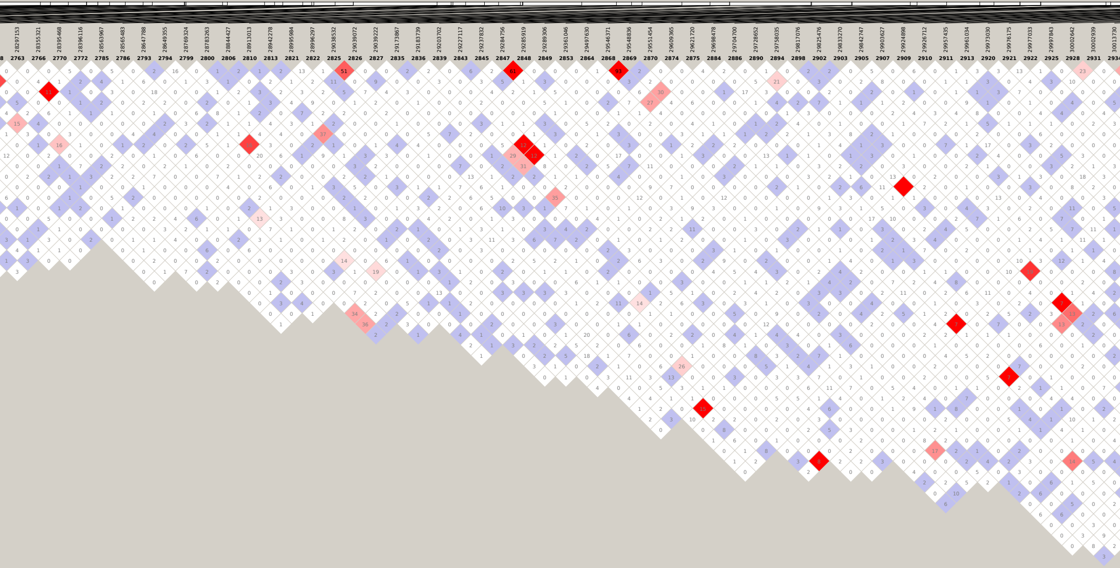
28297153 28355321 28395468 28396116 28563967 28565483 28647788 28649355 28769324 28783263 28844427 28913013 28942278 28995984 28996297 29038532 29039072 29039222 29173867 29183739 29203702 29227117 29237832 29284756 29285919 29289306 29361046 29497630 29546371 29548836 29551454 29609365 29621720 29698478 29704700 29728652 29758035 29817076 29825476 29833270 29842747 29903627 29924898 29926712 29957435 29961034 29973030 29976175 29977033 29997843 30002642 30002939 30013730
2763 2766 2770 2772 2785 2786 2793 2794 2799 2800 2806 2810 2813 2821 2822 2825 2826 2827 2835 2836 2839 2843 2845 2847 2848 2849 2853 2864 2868 2869 2870 2874 2875 2884 2886 2890 2894 2898 2902 2903 2905 2907 2909 2910 2911 2913 2920 2921 2922 2925 2928 2931 2934

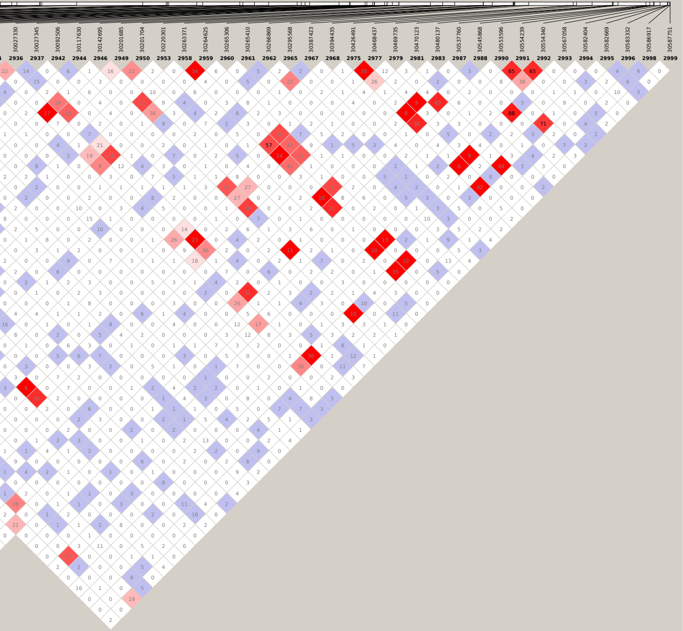

30027330
30027345
30092506
30117630
30142695
30201685
30201704
30220301
30263371
30264625
30265306
30265410
30266869
30295568
30387423
30394435
30426491
30468437
30469735
30470123
30480137
30537760
30545868
30553596
30554239
30554340
30567058
30582404
30582669
30583332
30586917
30587751
2936 2937 2942 2944 2946 2949 2950 2953 2958 2959 2960 2961 2962 2965 2967 2968 2975 2977 2979 2981 2983 2987 2988 2990 2991 2992 2993 2994 2995 2996 2998 2999

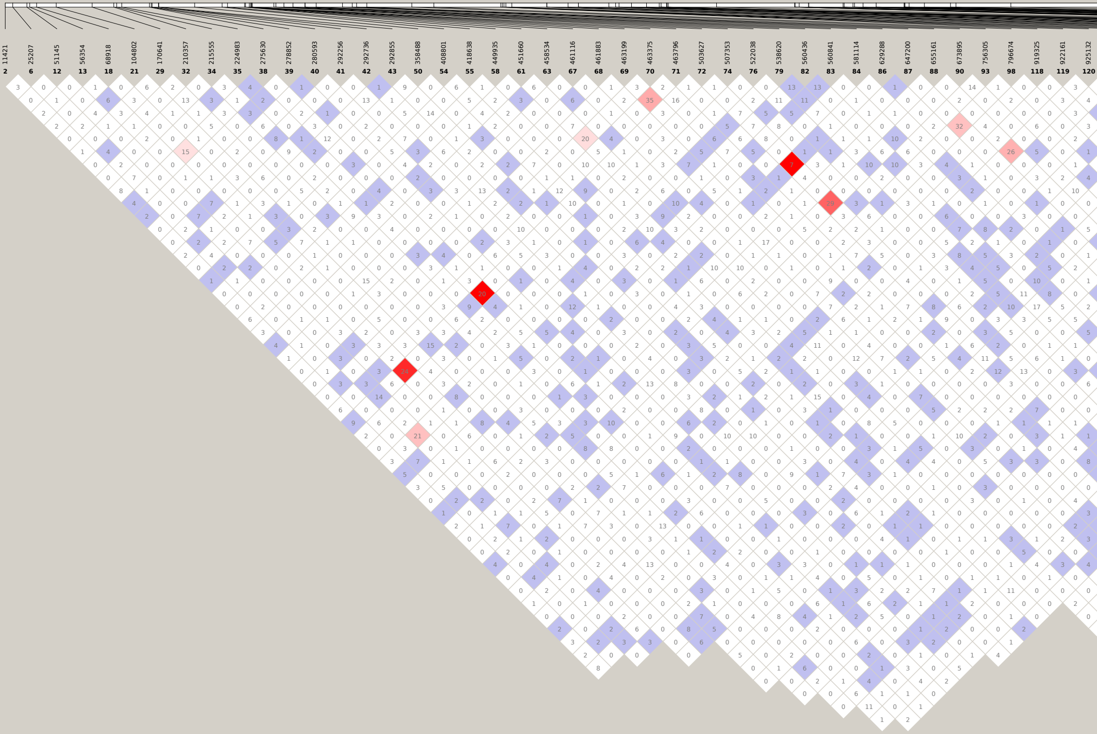

11421
25207
51145
56354
68918
104802
170641
210357
215555
224983
275630
278852
280593
292256
292736
292855
358488
408801
418638
449935
451660
458534
461116
461883
463199
463375
463796
503627
507353
522038
538620
560436
560841
581114
629288
647200
655161
673895
756305
796674
919325
922161
925132
2 6 12 13 18 21 29 32 34 35 38 39 40 41 42 43 50 54 55 58 61 63 67 68 69 70 71 72 74 76 79 82 83 84 86 87 88 90 93 98 118 119 120

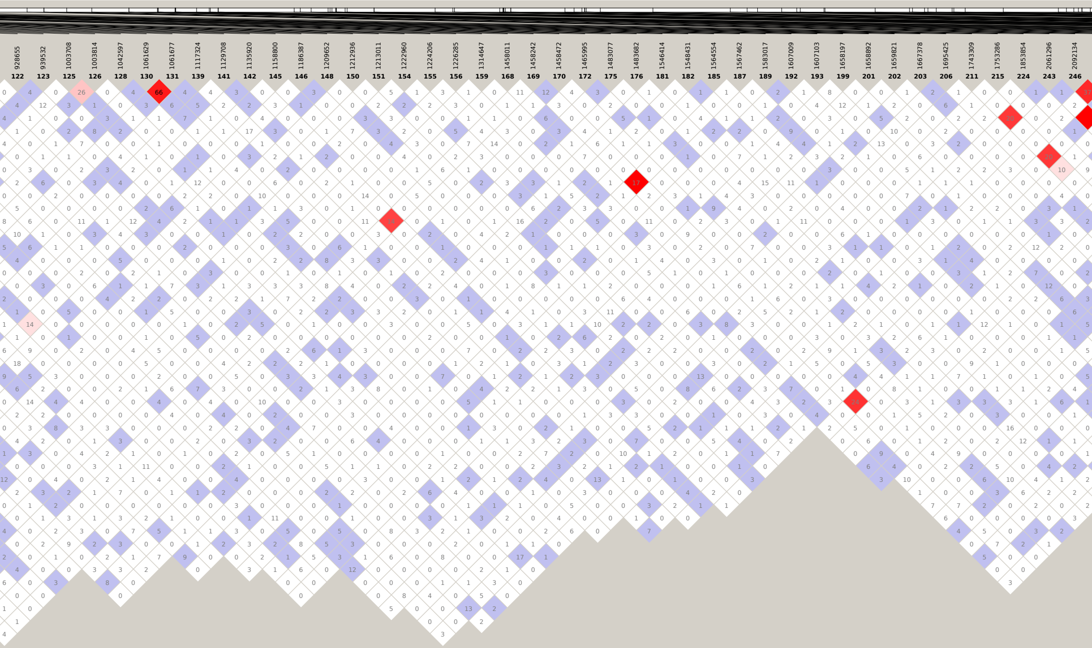

928655 122
939532 123
1003708 125
1003814 126
1042597 128
1061629 130
1061677 131
1117324 139
1129708 141
1135920 142
1158800 145
1186387 146
1209652 148
1212936 150
1213011 151
1222960 154
1224206 155
1226285 156
1314647 159
1458011 168
1458242 169
1458472 170
1465995 172
1483077 175
1483682 176
1546414 181
1548431 182
1564554 185
1567462 187
1583017 189
1607009 192
1607103 193
1658197 199
1658892 201
1659821 202
1667378 203
1695425 206
1743309 211
1753286 215
1853854 224
2061296 243
2092134 246

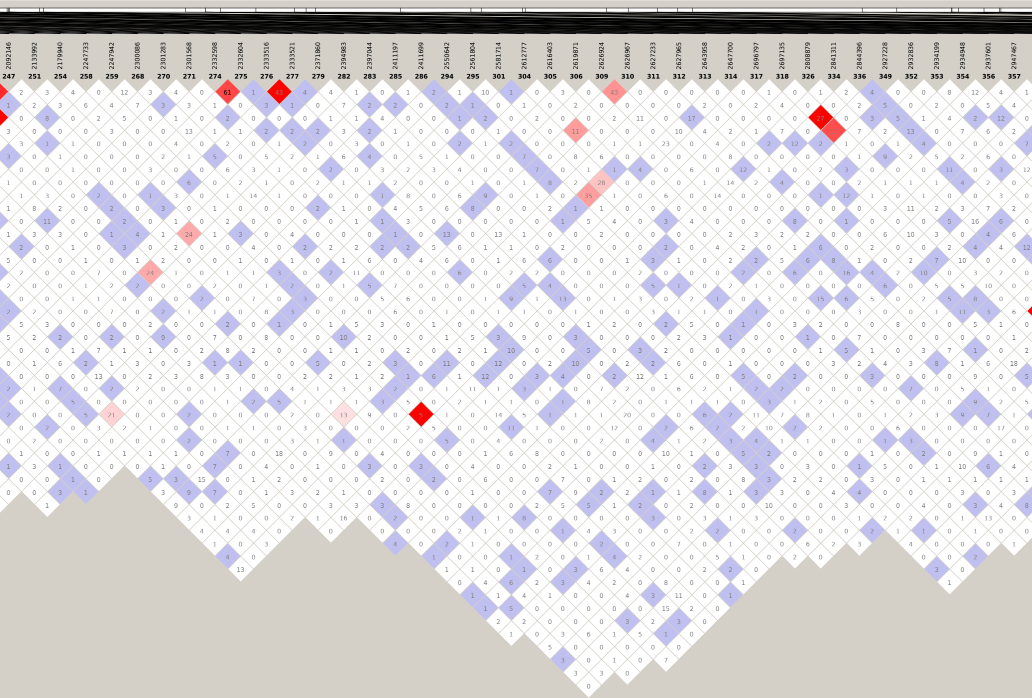
2092146
247
2133992
251
2179940
254
2247733
258
2247942
259
2300086
268
2301283
270
2301568
271
2332598
274
2332604
275
2333516
276
2333521
277
2371860
279
2394983
282
2397044
283
2411197
285
2411699
286
2550642
294
2561804
295
2581714
301
2612777
304
2616403
305
2619871
306
2626924
309
2626967
310
2627233
311
2627965
312
2643958
313
2647700
314
2696797
317
2697135
318
2808879
326
2841311
334
2844396
336
2927228
349
2932836
352
2934199
353
2934948
354
2937601
356
2947467
357

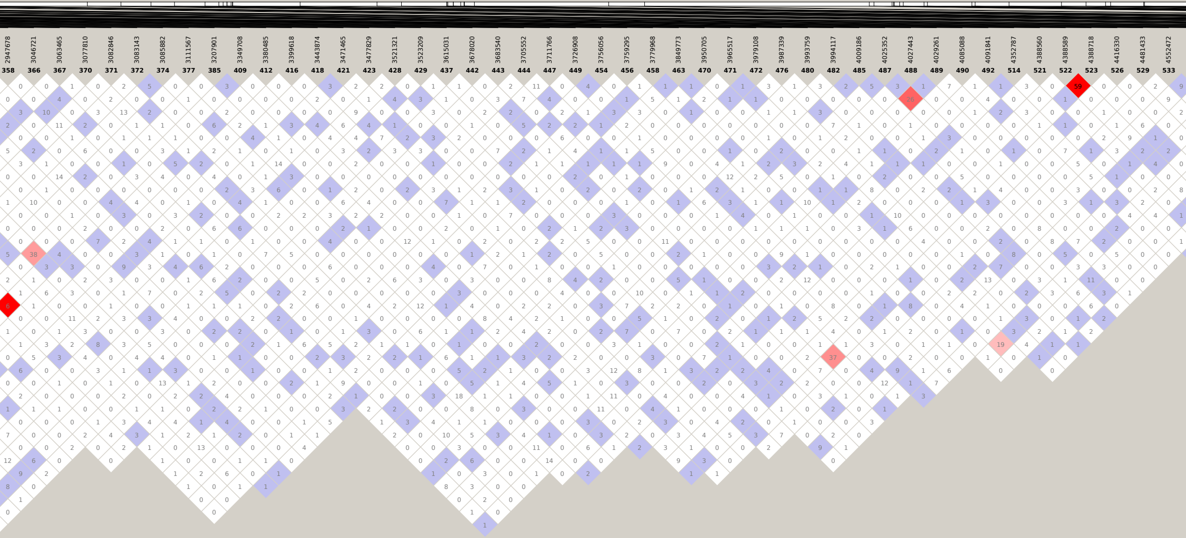

2947678 3046721 3063465 3077810 3082846 3083143 3085882 3111567 3207901 3349708 3380485 3399618 3443874 3471465 3477829 3521321 3523209 3615031 3678020 3683540 3705552 3711766 3726908 3756056 3759295 3779968 3849773 3950705 3965517 3979108 3987339 3993759 3994117 4009186 4025352 4027443 4029261 4085088 4091841 4352787 4388560 4388589 4388718 4416330 4481433 4552472
358 366 367 370 371 372 374 377 385 409 412 416 418 421 423 428 429 437 442 443 444 447 449 454 456 458 463 470 471 472 476 480 482 485 487 488 489 490 492 514 521 522 523 526 529 533

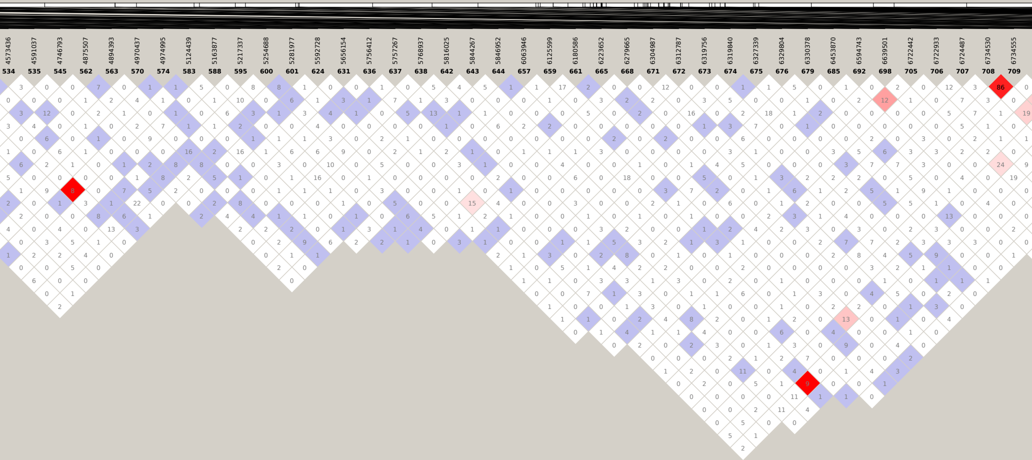
4573436 4591037 4746793 4875507 4894393 4970437 4974995 5124439 5163877 5217337 5254688 5281977 5592728 5656154 5756412 5757267 5768937 5816025 5844267 5846952 6063946 6125599 6180586 6223652 6279665 6304987 6312787 6319756 6319840 6327339 6329804 6330378 6453870 6594743 6639501 6722442 6722933 6724487 6734530 6734555
534 535 545 562 563 570 574 583 588 595 600 601 624 631 636 637 638 642 643 644 657 659 661 665 668 671 672 673 674 675 676 679 685 692 698 705 706 707 708 709

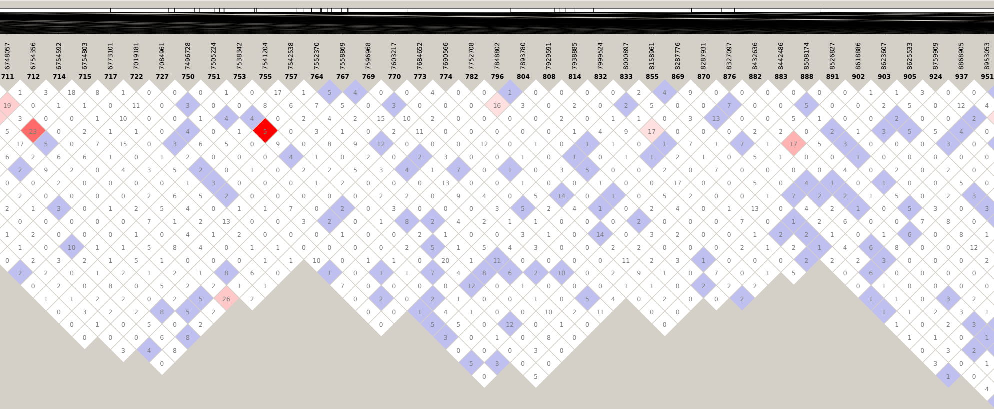

6748057 711
6754356 712
6754592 714
6754803 715
6773101 717
7019181 722
7084961 727
7496728 750
7505224 751
7538342 753
7541204 755
7542538 757
7552370 764
7558869 767
7596968 769
7603217 770
7684652 773
7690566 774
7752708 782
7848802 796
7893780 804
7929591 808
7938885 814
7999524 832
8000897 833
8158961 855
8287776 869
8287931 870
8327097 876
8432636 882
8442486 883
8508174 888
8526827 891
8618886 902
8623607 903
8625533 905
8759909 924
8868905 937
8953053 951

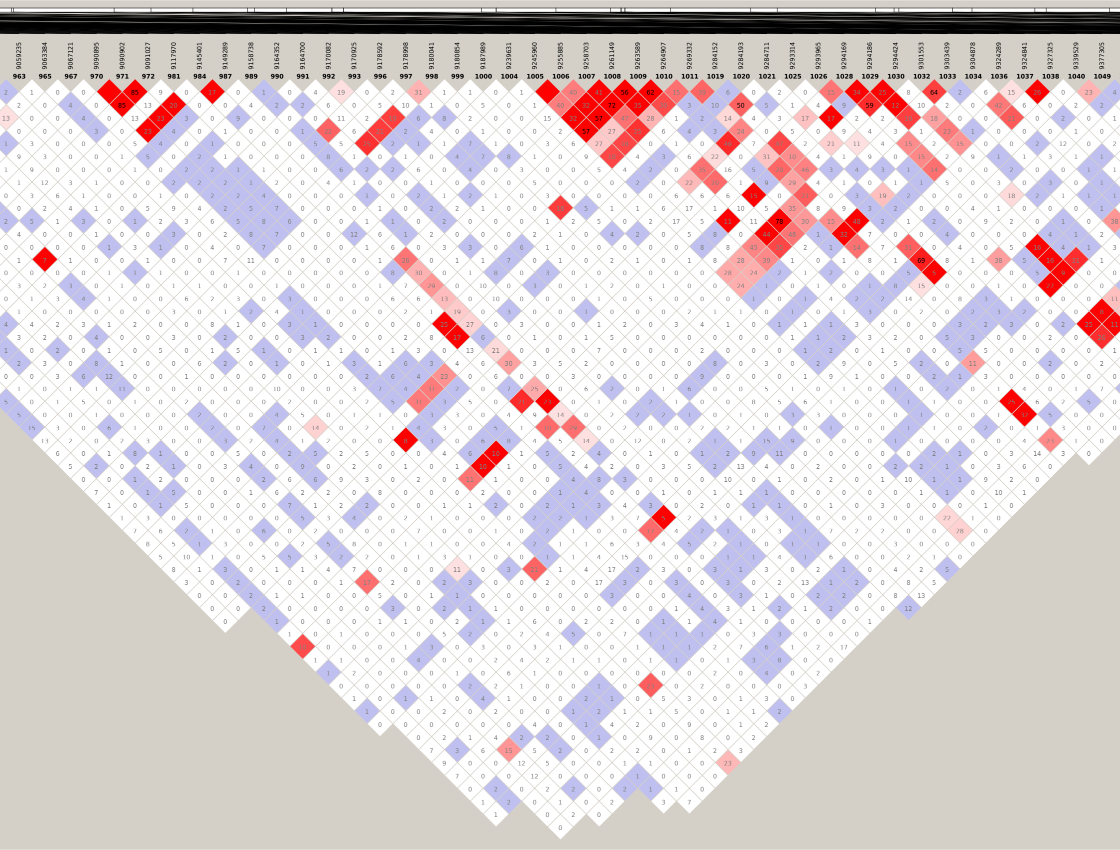
9059235 963
9063384 965
9067121 967
9090895 970
9090902 971
9091027 972
9117970 981
9145401 984
9149289 987
9158738 989
9164352 990
9164700 991
9170082 992
9170925 993
9178592 996
9178998 997
9180041 998
9180854 999
9187989 1000
9239631 1004
9245960 1005
9255885 1006
9258703 1007
9261149 1008
9263589 1009
9264907 1010
9269332 1011
9284152 1019
9284193 1020
9284711 1021
9293314 1025
9293965 1026
9294169 1028
9294186 1029
9294424 1030
9301553 1032
9303439 1033
9304878 1034
9324289 1036
9324841 1037
9327325 1038
9339529 1040
9377305 1049

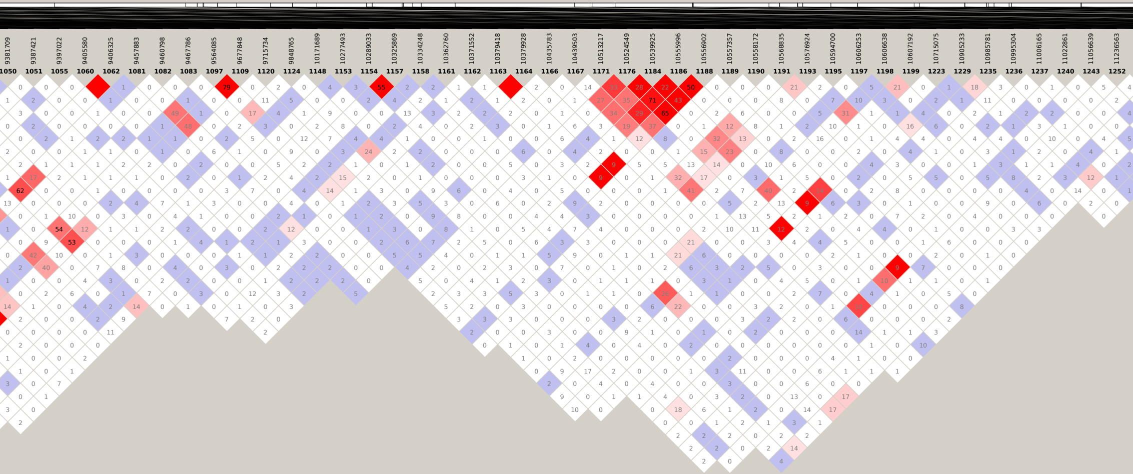
9381709 9387421 9397022 9405580 9406325 9457883 9460798 9467786 956408 9677848 9715734 9848765 10171689 10277493 10289033 10325869 10334248 10362760 10371552 10379418 10379928 10435783 10439503 10513217 10524549 10539925 10555996 10556902 10557357 10558172 10568835 10576924 10594700 10606253 10606638 10607192 10715075 10905233 10985781 10995304 11006165 11022861 11056639 11236563
1050 1051 1055 1060 1062 1081 1082 1083 1097 1109 1120 1124 1148 1153 1154 1157 1158 1161 1162 1163 1164 1166 1167 1171 1176 1184 1186 1188 1189 1190 1191 1193 1195 1197 1198 1199 1223 1229 1235 1236 1237 1240 1243 1252

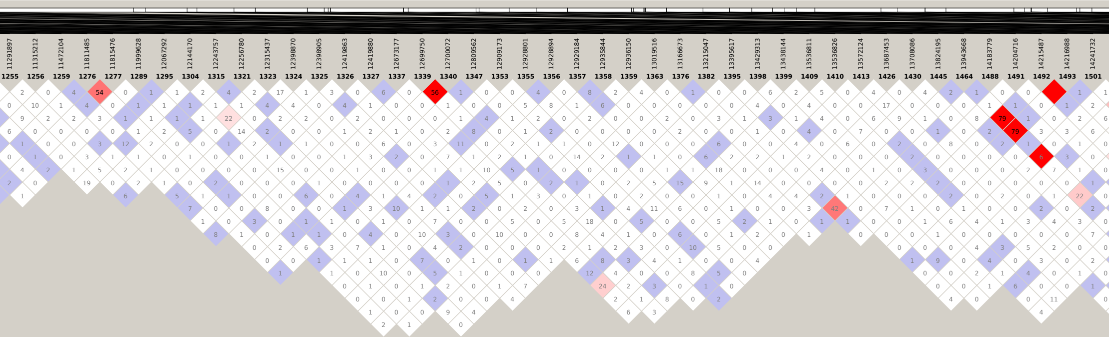

11291897
11315212
11472104
11811485
11815476
11999628
12067292
12144170
12243757
12256780
12315437
12398870
12398905
12419863
12419880
12673177
12699750
12700072
12809562
12909173
12928801
12928894
12929184
12935844
12936150
13019516
13166673
13215047
13395617
13429313
13438144
13536811
13536826
13572124
13687453
13708086
13824195
13943668
14183779
14204716
14215487
14216988
14241732
1255
1256
1259
1276
1277
1289
1295
1304
1315
1321
1323
1324
1325
1326
1327
1337
1339
1340
1347
1353
1355
1356
1357
1358
1359
1363
1376
1382
1395
1398
1399
1409
1410
1413
1426
1430
1445
1464
1488
1491
1492
1493
1501

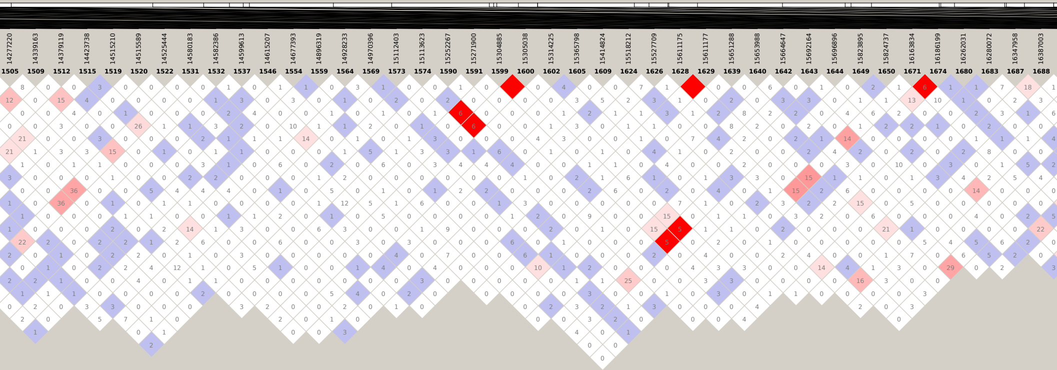
14277220
1505
14339163
1509
14379119
1512
14423738
1515
14515210
1519
14515589
1520
14525444
1522
14580183
1531
14582386
1532
14599613
1537
14615207
1546
14677393
1554
14896319
1559
14928233
1564
14970396
1569
15112403
1573
15113623
1574
15252267
1590
15271900
1591
15304885
1599
15305038
1600
15314225
1602
15365798
1605
15414824
1609
15518212
1624
15527709
1626
15611175
1628
15611177
1629
15651288
1639
15653988
1640
15664647
1642
15692164
1643
15696896
1644
15823895
1649
15824737
1650
16163834
1671
16186199
1674
16262031
1680
16280072
1683
16347958
1687
16387003
1688

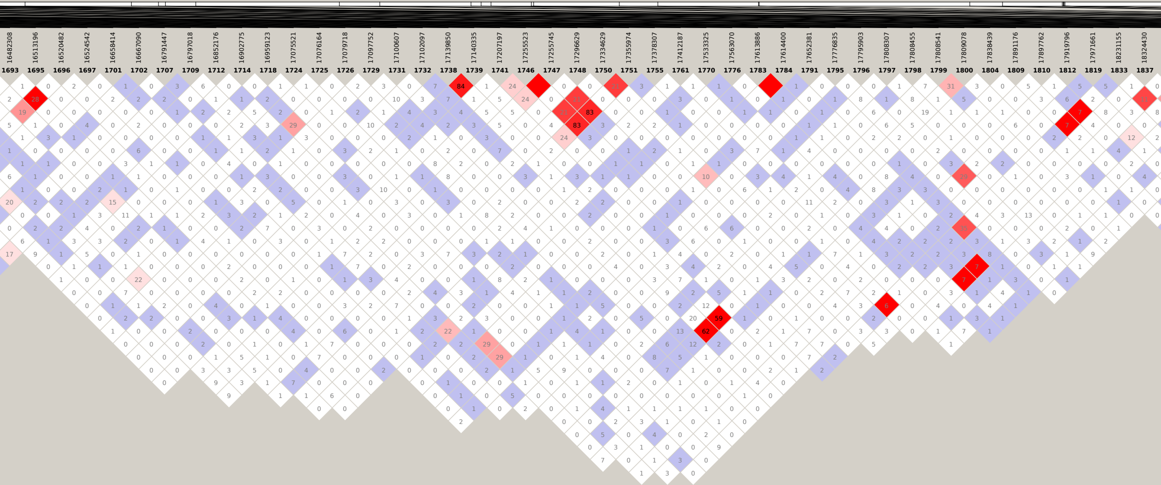
16482308 16513196 16520482 16524542 16658414 16667090 16791447 16797018 16852176 16902775 16959123 17075521 17076164 17079718 17097752 17100607 17102097 17139850 17140335 17207197 17255523 17255745 17296629 17334629 17355974 17378307 17412187 17533325 17563070 17613886 17614400 17652381 17776835 17795903 17808307 17808455 17808541 17809078 17838439 17891176 17897762 17919796 17971661 18231155 18324430
1693 1695 1696 1697 1701 1702 1707 1709 1712 1714 1718 1724 1725 1726 1729 1731 1732 1738 1739 1741 1746 1747 1748 1750 1751 1755 1761 1770 1776 1783 1784 1791 1795 1796 1797 1798 1799 1800 1804 1809 1810 1812 1819 1833 1837

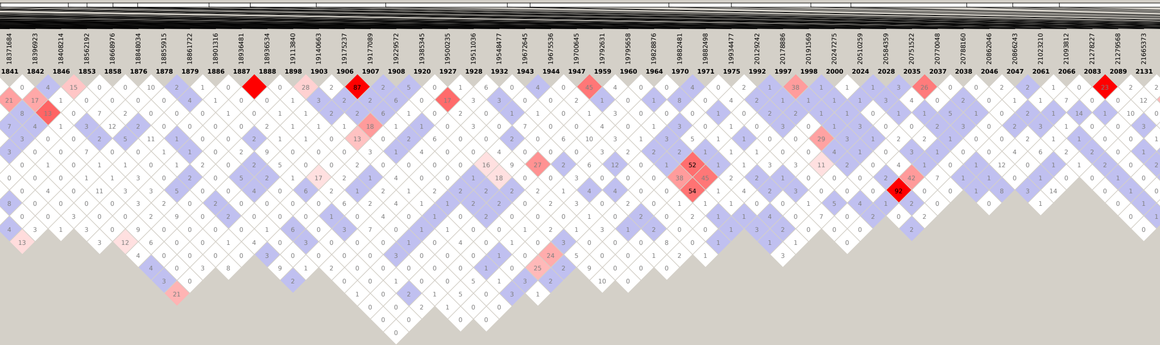
18371684 1841
18396923 1842
18408214 1846
18562192 1853
18668976 1858
18848034 1876
18855915 1878
18861722 1879
18901316 1886
18936481 1887
18936534 1888
19113840 1898
19140663 1903
19175237 1906
19177089 1907
19229572 1908
19385345 1920
19500235 1927
19511036 1928
19548477 1932
19672645 1943
19675536 1944
19700645 1947
19792631 1959
19795658 1960
19828876 1964
19882481 1970
19882498 1971
19934477 1975
20129242 1992
20178886 1997
20191569 1998
20247275 2000
20510259 2024
20584359 2028
20751522 2035
20770048 2037
20788160 2038
20862046 2046
20866243 2047
21023210 2061
21093812 2066
21278227 2083
21279568 2089
21665373 2131

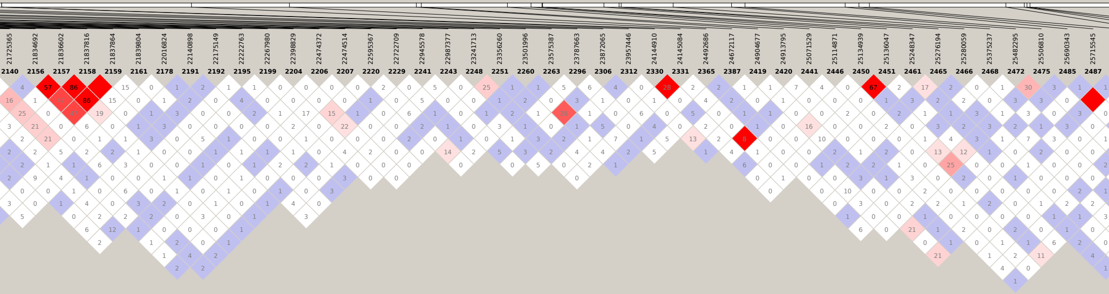
21725365
21834692
21836602
21837816
21837864
21839804
22016824
22140898
22175149
22222763
22267980
22398829
22474372
22474514
22595367
22722709
22945578
22987377
23241713
23356260
23501996
23575387
23787663
23872065
23957446
24144910
24145084
24492686
24672117
24904677
24913795
25071529
25114871
25134939
25136047
25248347
25276194
25280059
25375237
25482295
25506810
25690343
25715545
2140 2156 2157 2158 2159 2161 2178 2191 2192 2195 2199 2204 2206 2207 2220 2229 2241 2243 2248 2251 2260 2263 2296 2306 2312 2330 2331 2365 2387 2419 2420 2441 2446 2450 2451 2461 2465 2466 2468 2472 2475 2485 2487

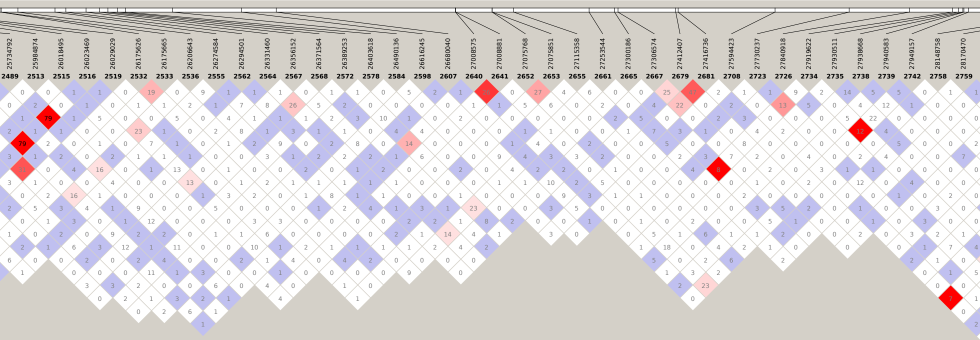
25734792 25984874 26018495 26023469 26029029 26175626 26175665 26206643 26274584 26294501 26331460 26356152 26371564 26389253 26403618 26490136 26616245 26680040 27008575 27008881 27075768 27075851 27115358 27253544 27300186 27306574 27412407 27416736 27594423 27730237 27840918 27919622 27930511 27938668 27940583 27949157 28148758 28170470
2489 2513 2515 2516 2519 2532 2533 2536 2555 2562 2564 2567 2568 2572 2578 2584 2598 2607 2640 2641 2652 2653 2655 2661 2665 2667 2679 2681 2708 2723 2726 2734 2735 2738 2739 2742 2758 2759

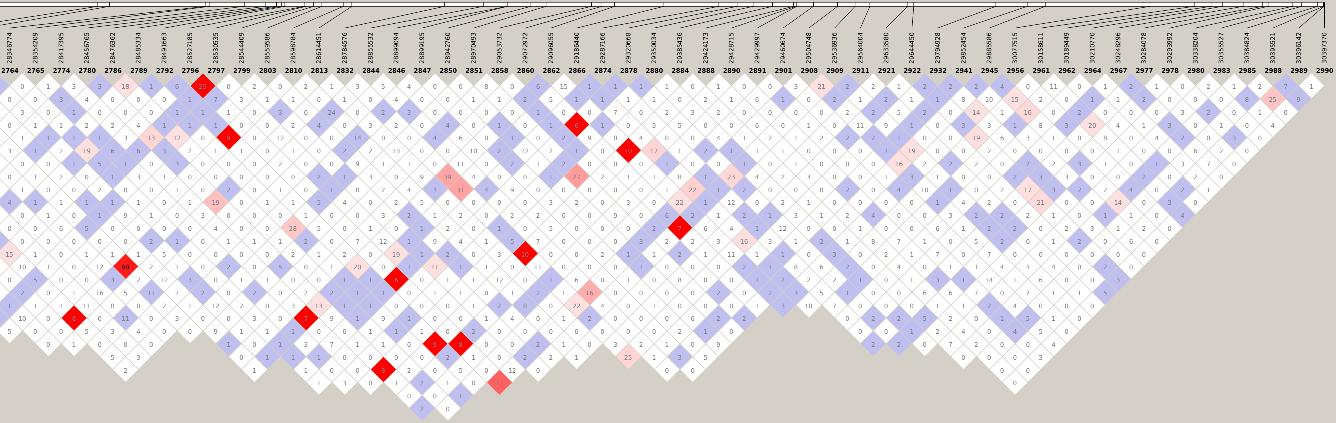
28346774
28354209
28417395
28456765
28476362
28485334
28491663
28527185
28530535
28544409
28559586
28598784
28614451
28784576
28855532
28899094
28899195
28942760
28970493
29053732
29072972
29096055
29186640
29287166
29320668
29350034
29385436
29424173
29428715
29429997
29460674
29504748
29536936
29564004
29633580
29644450
29794928
29852454
29885586
30077515
30158611
30189449
30210770
30248296
30284078
30293992
30338204
30355527
30384824
30395521
30396142
30397370
2764 2765 2774 2780 2786 2789 2792 2796 2797 2799 2803 2810 2813 2832 2844 2846 2847 2850 2851 2858 2860 2862 2866 2874 2878 2880 2884 2888 2890 2891 2901 2908 2909 2911 2921 2922 2932 2941 2945 2956 2961 2962 2964 2967 2977 2978 2980 2983 2985 2988 2989 2990

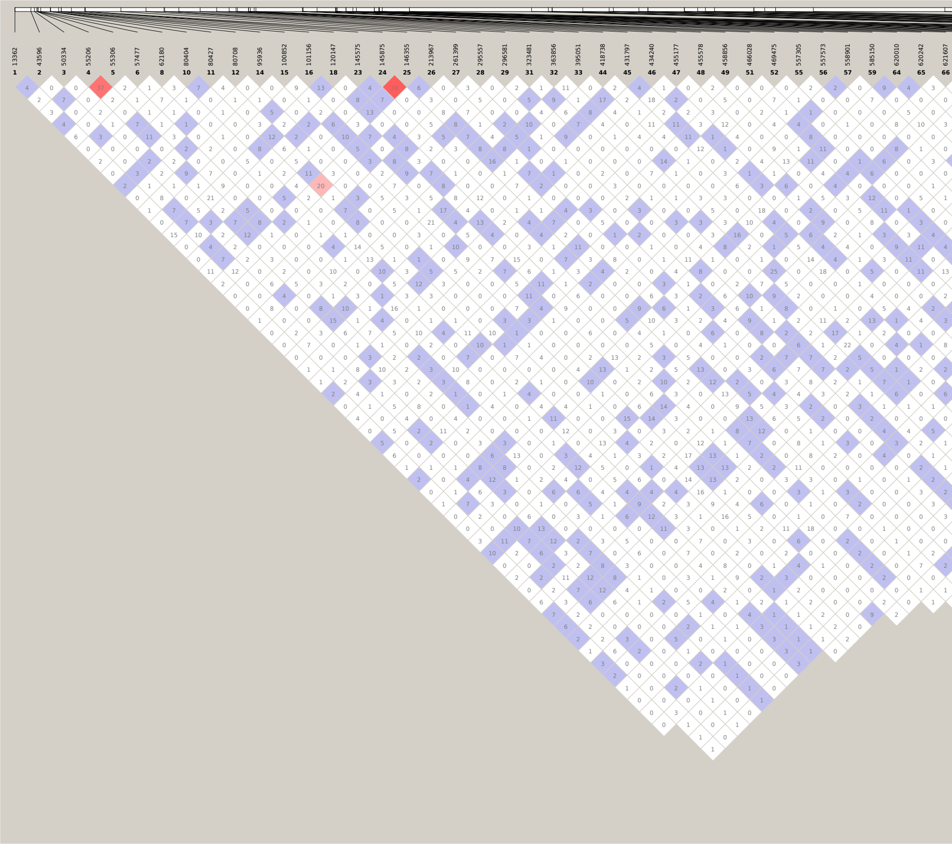
13362 1
43596 2
50334 3
55206 4
55306 5
57477 6
62180 8
80404 10
80427 11
80708 12
95936 14
100852 15
101156 16
120147 18
145575 23
145875 24
146355 25
213967 26
261399 27
295557 28
296581 29
323481 31
363856 32
395051 33
418738 44
431797 45
434240 46
455177 47
455578 48
458856 49
466028 51
469475 52
557305 55
557573 56
558901 57
585150 59
620010 64
620242 65
621607 66

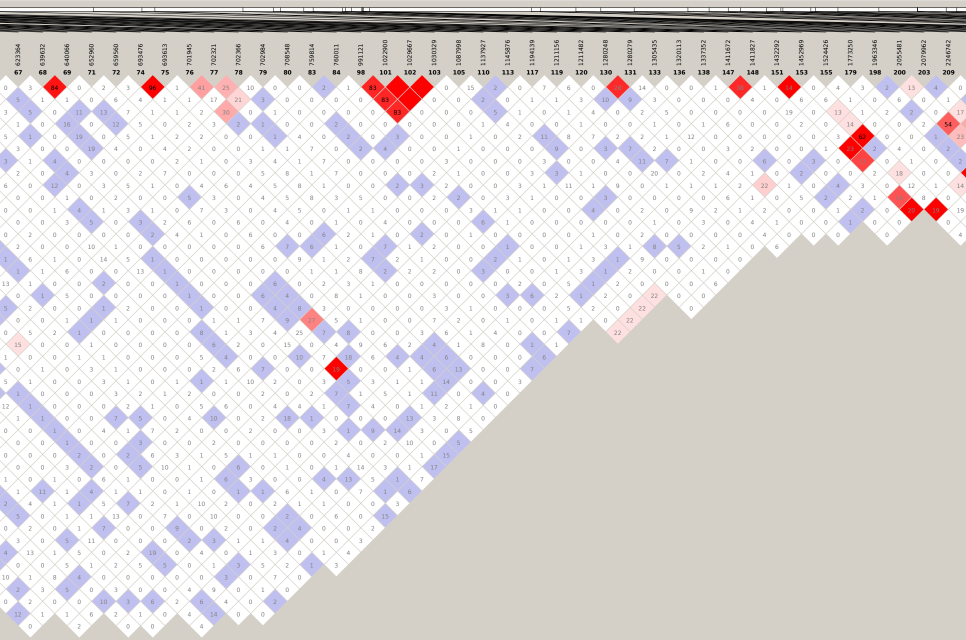
623364 67
639632 68
640066 69
652960 71
659560 72
693476 74
693613 75
701945 76
702321 77
702366 78
702984 79
708548 80
759814 83
760011 84
991121 98
1022900 101
1029667 102
1030329 103
1087998 105
1137927 110
1145876 113
1194139 117
1211156 119
1211482 120
1280248 130
1280279 131
1305435 133
1320113 136
1337352 138
1411672 147
1411827 148
1432292 151
1452969 153
1524426 155
1773250 179
1963346 198
2055481 200
2079962 203
2246742 209

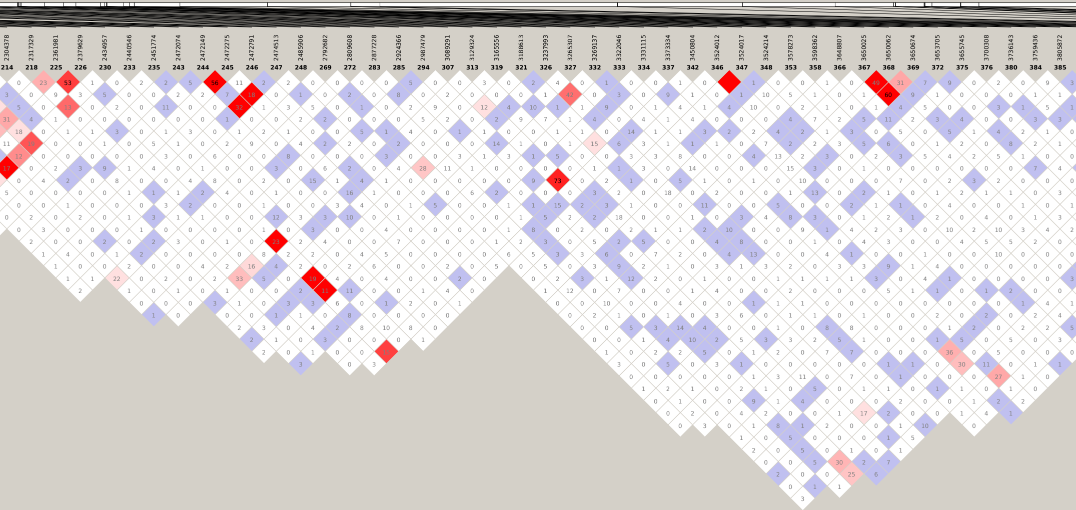
2304378 214
2317329 218
2361981 225
2379629 226
2434957 230
2440546 233
2451774 235
2472074 243
2472149 244
2472275 245
2472791 246
2474513 247
2485906 248
2792682 269
2809608 272
2877228 283
2924366 285
2987479 294
3089291 307
3129324 313
3165556 319
3188613 321
3237993 326
3265307 327
3269137 332
3322046 333
3331115 334
3373334 337
3450804 342
3524012 346
3524017 347
3524214 348
3578273 353
3598362 358
3648807 366
3650025 367
3650062 368
3650674 369
3653705 372
3655745 375
3700308 376
3736143 380
3759436 384
3805872 385

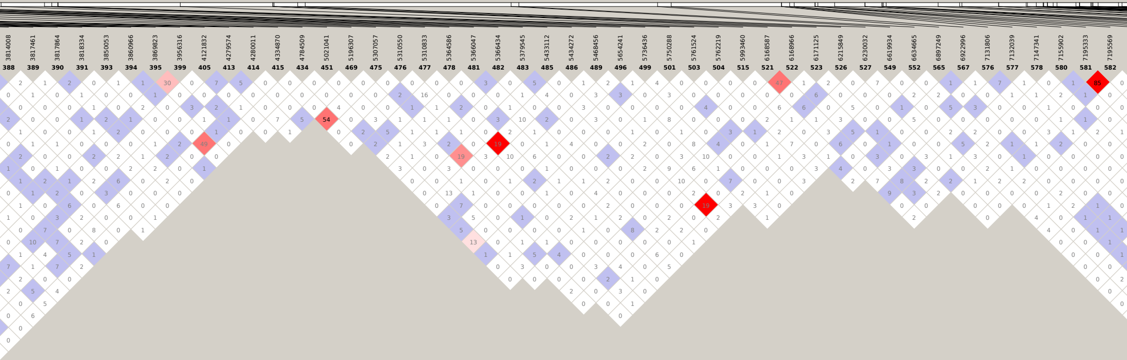
3814008 3817461 3817864 3818334 3850053 3860966 3869823 3956316 4121832 4279574 4280011 4334870 4784509 5021041 5196307 5307057 5310550 5310833 5364586 5366047 5366434 5379545 5433112 5434272 5468456 5654241 5736436 5750288 5761524 5762219 5993460 6168587 6168966 6171125 6215849 6230032 6619934 6634665 6897249 6922996 7131806 7132039 7147341 7155902 7195333 7195569
388 389 390 391 393 394 395 399 405 413 414 415 434 451 469 475 476 477 478 481 482 483 485 486 489 496 499 501 503 504 515 521 522 523 526 527 549 552 565 567 576 577 578 580 581 582

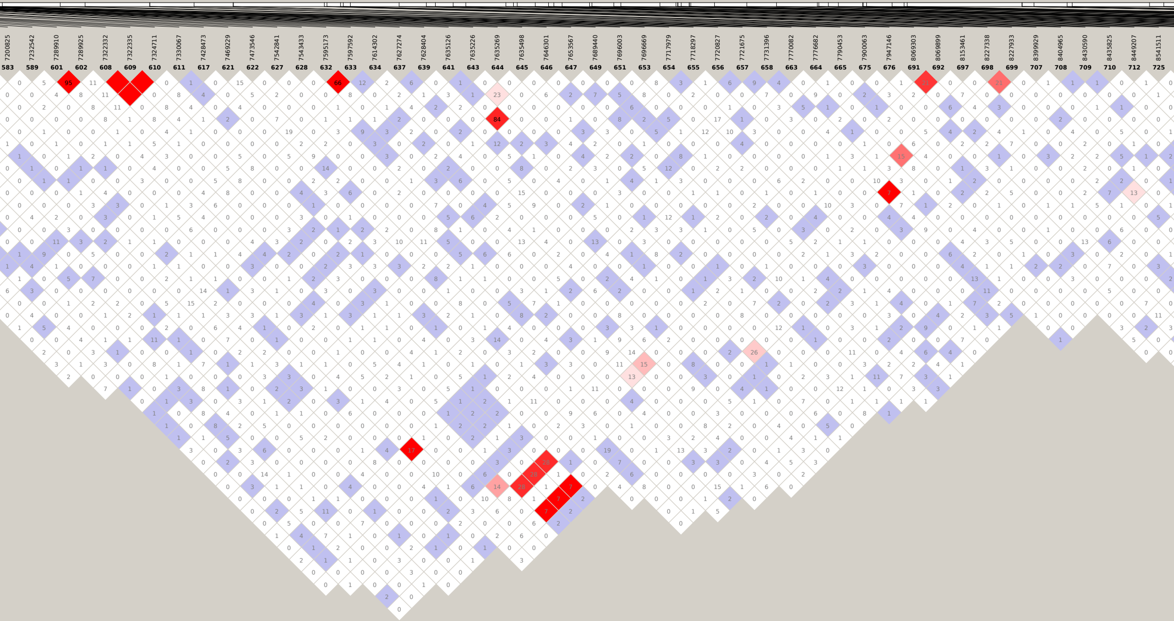
7200825
583
7232542
589
7289910
601
7289925
602
7322332
608
7322335
609
7324711
610
7330067
611
7428473
617
7469229
621
7473546
622
7542841
627
7543433
628
7595173
632
7597592
633
7614302
634
7627274
637
7628404
639
7635126
641
7635226
643
7635269
644
7635498
645
7646301
646
7653567
647
7689440
649
7696003
651
7696669
653
7717979
654
7718297
655
7720827
656
7721675
657
7731396
658
7770082
663
7776682
664
7790453
665
7900063
675
7947146
676
8069303
691
8069899
692
8153461
697
8227338
698
8227933
699
8399929
707
8404965
708
8430590
709
8435825
710
8449207
712
8541511
725

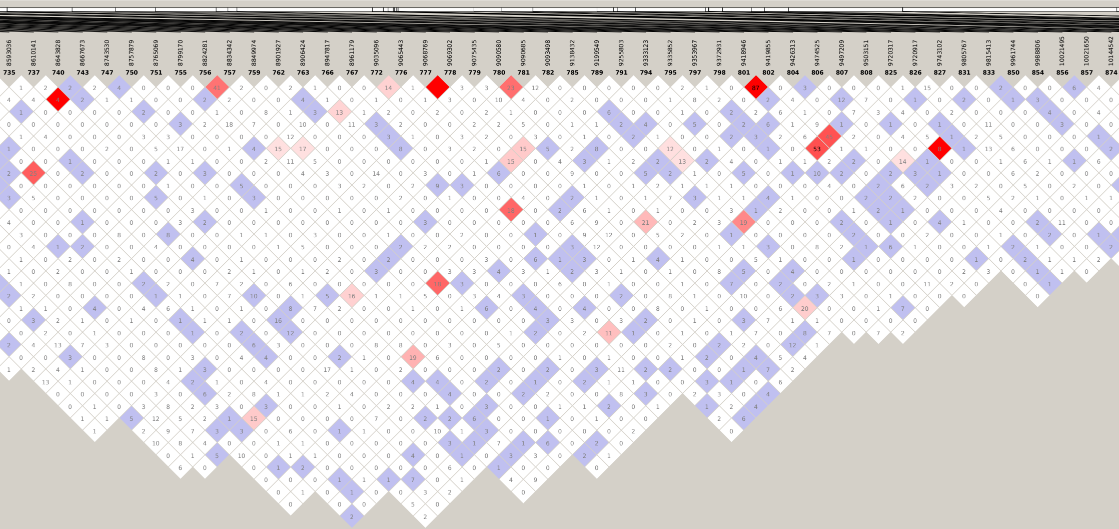

8593036
8610141
8643828
8667673
8743530
8757879
8765069
8799170
8824281
8834342
8849974
8901927
8906424
8947817
8961179
9035096
9065443
9068769
9069302
9075435
9090580
9090685
9093498
9138432
9199549
9255803
9333123
9335852
9353967
9372931
9418946
9419855
9426313
9474525
9497209
9503151
9720317
9720917
9743102
9805767
9815413
9961744
9988806
10021495
10021650
10144542
735
737
740
743
747
750
751
755
756
757
759
762
763
766
767
772
776
777
778
779
780
781
782
785
789
791
794
795
797
798
801
802
804
806
807
808
825
826
827
831
833
850
854
856
857
874

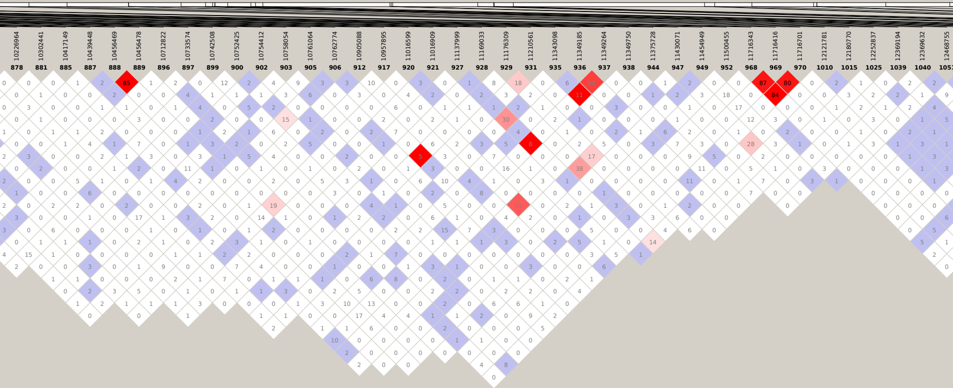

10226964
10302441
10417149
10439448
10456469
10456478
10712822
10733574
10742508
10752425
10754412
10758054
10761064
10762774
10905088
10957895
11016599
11016909
11137999
11169033
11176309
11210561
11343098
11349185
11349264
11349750
11375728
11430071
11454949
11500455
11716343
11716416
11716701
12121781
12180770
12252837
12369194
12369632
12468755
878
881
885
887
888
889
896
897
899
900
902
903
905
906
912
917
920
921
927
928
929
931
935
936
937
938
944
947
949
952
968
969
970
1010
1015
1025
1039
1040

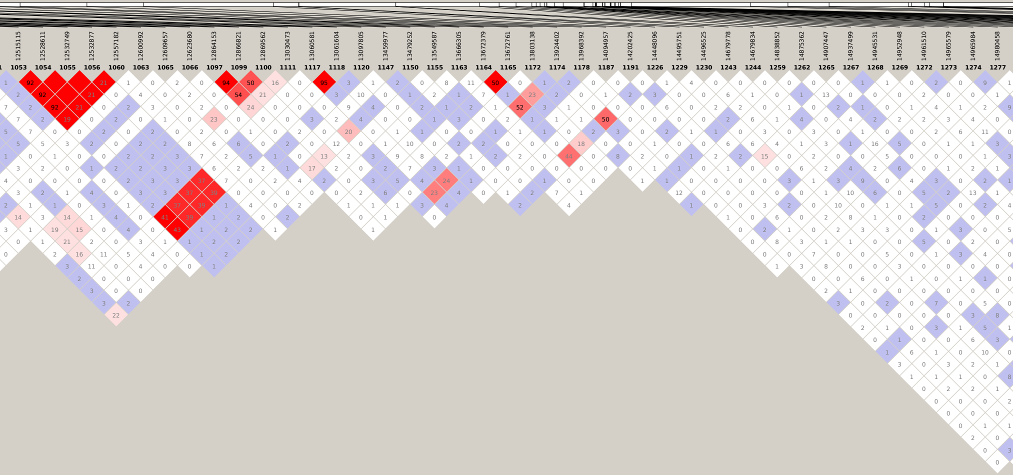
12515115
12528611
12532749
12532877
12557182
12600992
12609657
12623680
12864153
12866821
12869562
13030473
13060581
13061604
13097805
13459977
13479252
13549587
13666305
13672379
13672761
13803138
13924402
13968392
14094957
14202425
14448096
14495751
14496525
14679778
14679834
14838852
14875362
14907447
14937499
14945531
14952948
14961510
14965579
14965984
14980458
1053
1054
1055
1056
1060
1063
1065
1066
1097
1099
1100
1111
1117
1118
1120
1147
1150
1155
1163
1164
1165
1172
1174
1178
1187
1191
1226
1229
1230
1243
1244
1259
1262
1265
1267
1268
1269
1272
1273
1274
1277

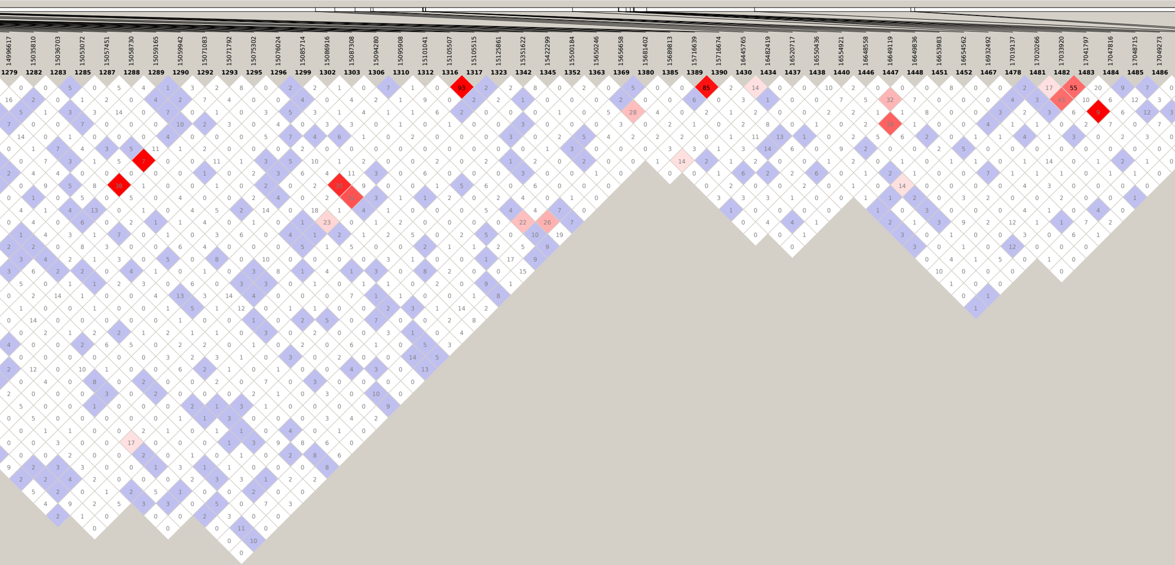
14996617
15035810
15036703
15053072
15057451
15058730
15059165
15059942
15071083
15071792
15075302
15076024
15085714
15086916
15087308
15094280
15095908
15101041
15105507
15105515
15125861
15351622
15422299
15500184
15650246
15656658
15681402
15689813
15716639
15716674
16445765
16482419
16520717
16550436
16554921
16648558
16649119
16649836
16653983
16654562
16932492
17019137
17020266
17033920
17041797
17047816
17048715
17049273
1279 1282 1283 1285 1287 1288 1289 1290 1292 1293 1295 1296 1299 1302 1303 1306 1310 1312 1316 1317 1323 1342 1345 1352 1363 1369 1380 1385 1389 1390 1430 1434 1437 1438 1440 1446 1447 1448 1451 1452 1467 1478 1481 1482 1483 1484 1485 1486

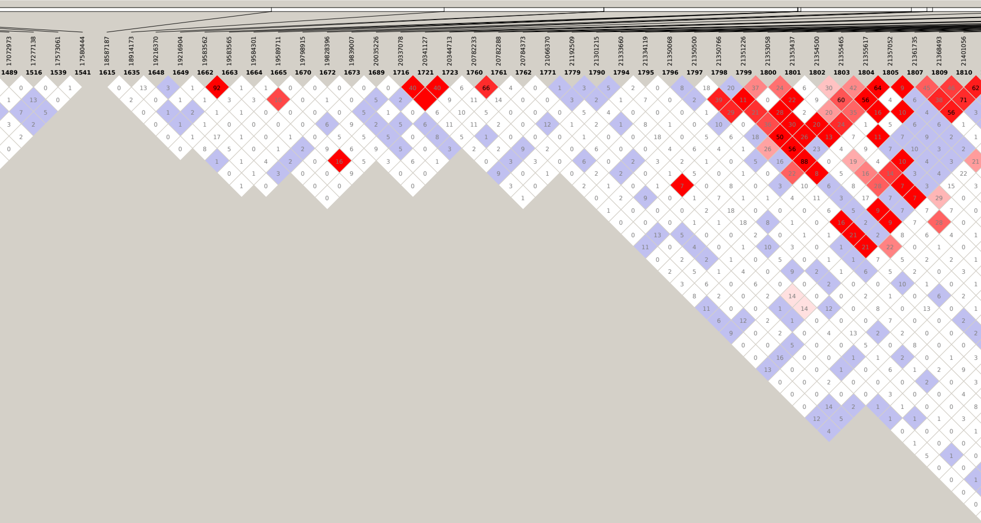

17072973 17277138 17573061 17580444 18587187 18914173 19216370 19216904 19583562 19583565 19584301 19589711 19798915 19828396 19839007 20035226 20337078 20341127 20344713 20782233 20782288 20784373 21066370 21192509 21301215 21333660 21334119 21350068 21350500 21350766 21351226 21353058 21353437 21354500 21355465 21355617 21357052 21361735 21368493 21401056
1489 1516 1539 1541 1615 1635 1648 1649 1662 1663 1664 1665 1670 1672 1673 1689 1716 1721 1723 1760 1761 1762 1771 1779 1790 1794 1795 1796 1797 1798 1799 1800 1801 1802 1803 1804 1805 1807 1809 1810

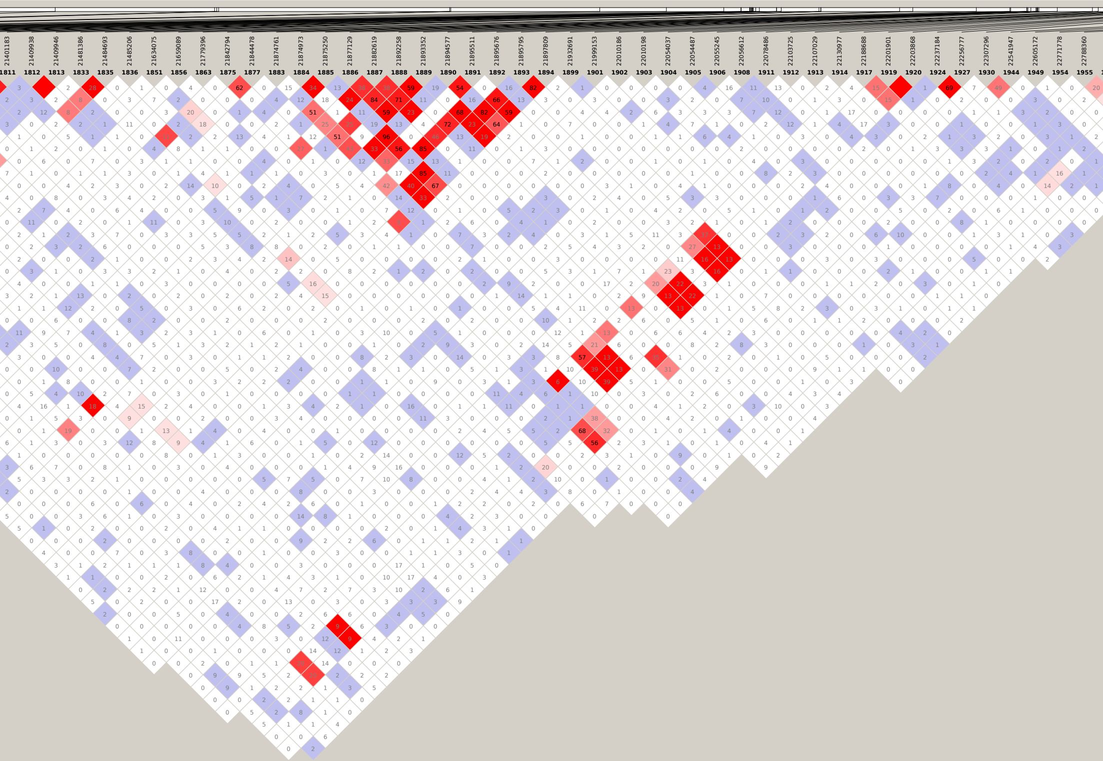
21401183 21409938 21409946 21481386 21484693 21485206 21634075 21659089 21779396 21842794 21844478 21874761 21874973 21875250 21877129 21882619 21892258 21893352 21894577 21895511 21895676 21895795 21897809 21932691 21999153 22010186 22010198 22054037 22054487 22055245 22056612 22078486 22103725 22107029 22130977 22188688 22201901 22203868 22237184 22256777 22307296 22541947 22605172 22771778 22788360
1811 1812 1813 1833 1835 1836 1851 1856 1863 1875 1877 1883 1884 1885 1886 1887 1888 1889 1890 1891 1892 1893 1894 1899 1901 1902 1903 1904 1905 1906 1908 1911 1912 1913 1914 1917 1919 1920 1924 1927 1930 1944 1949 1954 1955

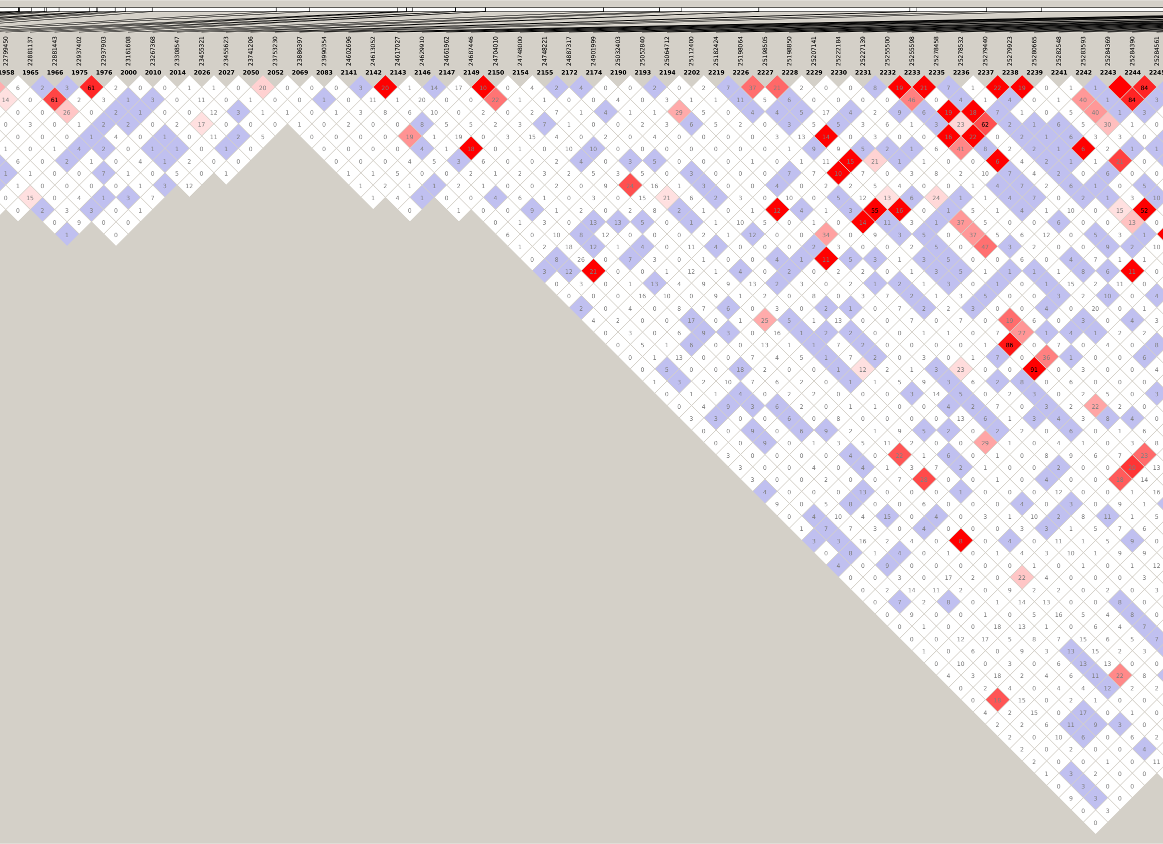
22799450
22881137
22881443
22937402
22937903
23161608
23267368
23308547
23455321
23455623
23741206
23753230
23886397
23990354
24602696
24613052
24617027
24629910
24661962
24687446
24704010
24748000
24748221
24887317
24901999
25032403
25052840
25064712
25112400
25182424
25198064
25198505
25198850
25207141
25222184
25227139
25255500
25255598
25278458
25278532
25279440
25279923
25280665
25282548
25283593
25284369
25284390
25284561
1958
1965
1966
1975
1976
2000
2010
2014
2026
2027
2050
2052
2069
2083
2141
2142
2143
2146
2147
2149
2150
2154
2155
2172
2174
2190
2193
2194
2202
2219
2226
2227
2228
2229
2230
2231
2232
2233
2235
2236
2237
2238
2239
2241
2242
2243
2244

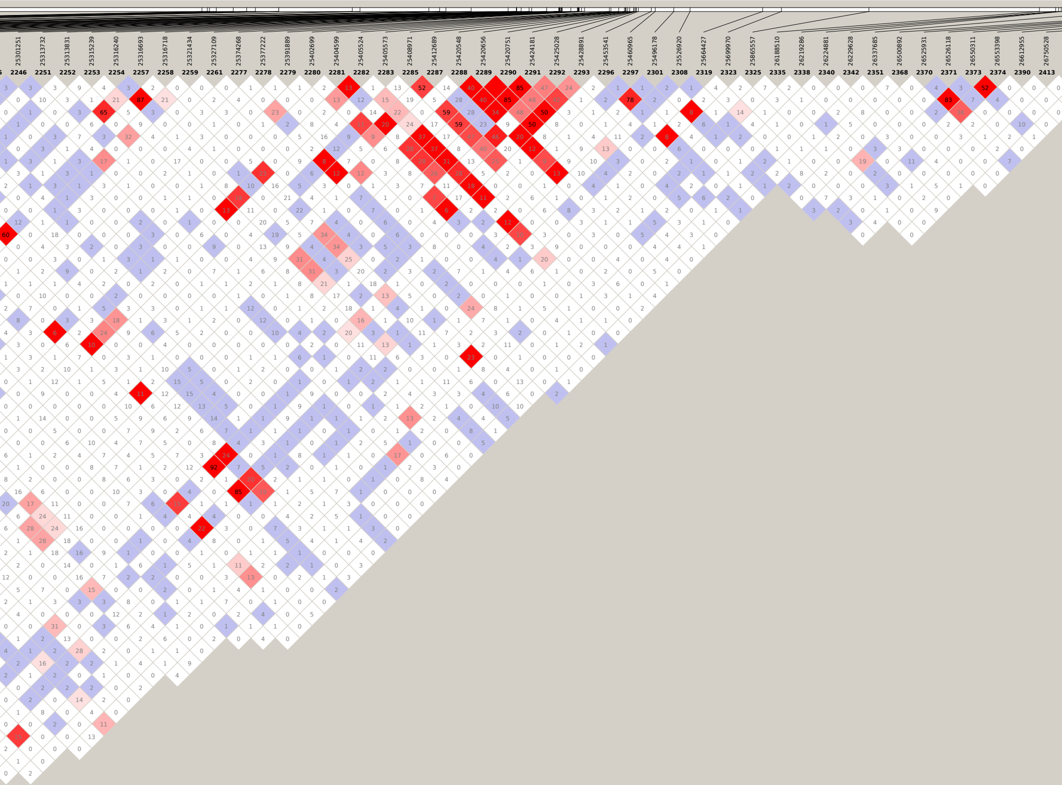
25301251
25313732
25313831
25315239
25316240
25316693
25316718
25321434
25327109
25374268
25377222
25391889
25402699
25404599
25405524
25405573
25408971
25412689
25420548
25420656
25420751
25424181
25425028
25428891
25453541
25460965
25496178
25526920
25664427
25699970
25865557
26188510
26219286
26224881
26229628
26337685
26500892
26525931
26526118
26550311
26553398
26612955
26750528
2246
2251
2252
2253
2254
2257
2258
2259
2261
2277
2278
2279
2280
2281
2282
2283
2285
2287
2288
2289
2290
2291
2292
2293
2296
2297
2301
2308
2319
2323
2325
2335
2338
2340
2342
2351
2368
2370
2371
2373
2374
2390
2413

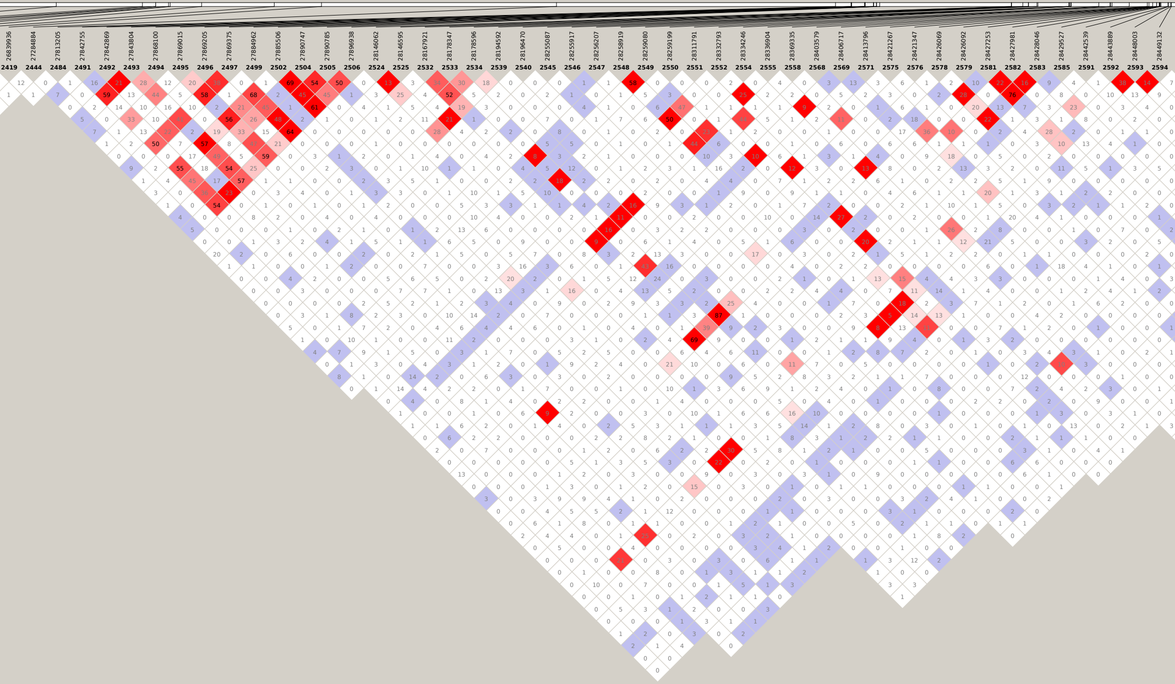

26839936
27284884
27813205
27842755
27842869
27843804
27868100
27869015
27869205
27869375
27884962
27885506
27890747
27890785
27896938
28146062
28146595
28167921
28178347
28178596
28194592
28196470
28255087
28255917
28256207
28258919
28259080
28259199
28311791
28332793
28334246
28336904
28369335
28403579
28406717
28413796
28421267
28421347
28426069
28426092
28427253
28427981
28428046
28429527
28442539
28443889
28448003
28449132
2419
2444
2484
2491
2492
2493
2494
2495
2496
2497
2499
2502
2504
2505
2506
2524
2525
2532
2533
2534
2539
2540
2545
2546
2547
2548
2549
2550
2551
2552
2554
2555
2558
2568
2569
2571
2575
2576
2578
2579
2581
2582
2583
2585
2591
2592
2593
2594

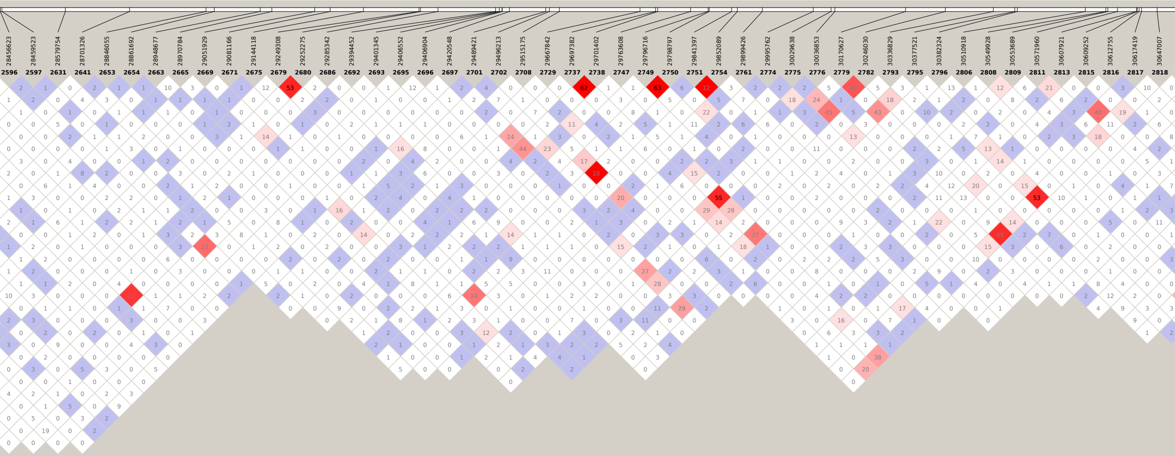
28456623
2596
28459523
2597
28579754
2631
28701326
2641
28846055
2653
28861692
2654
28948677
2663
28970784
2665
29051929
2669
29081166
2671
29144118
2675
29249308
2679
29252275
2680
29285342
2686
29394452
2692
29401345
2693
29406552
2695
29406904
2696
29420548
2697
29489421
2701
29496213
2702
29515175
2708
29667842
2729
29697382
2737
29701402
2738
29763608
2747
29796716
2749
29798797
2750
29841397
2751
29852089
2754
29899426
2761
29995762
2774
30029638
2775
30036853
2776
30170627
2779
30246030
2782
30336829
2793
30377521
2795
30382324
2796
30510918
2806
30549928
2808
30553689
2809
30571960
2811
30607921
2813
30609252
2815
30612755
2816
30617419
2817
30647007
2818

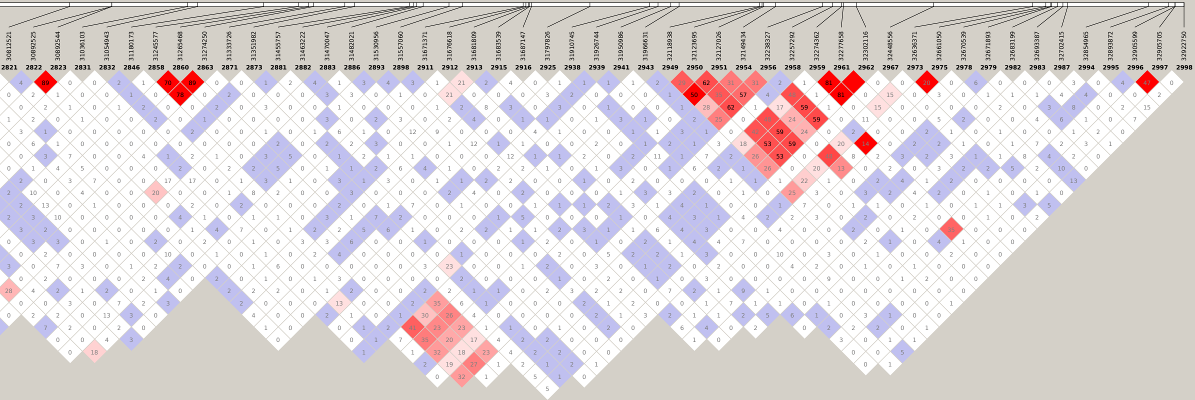
30812521
30892525
30892544
31036103
31054943
31180173
31245577
31265468
31274250
31333726
31351982
31455757
31463222
31470047
31482021
31530956
31557060
31671371
31676618
31681809
31683539
31687147
31797826
31910745
31926744
31950986
31966631
32118938
32123695
32127026
32149434
32238327
32257292
32274362
32277658
32302116
32448556
32636371
32661050
32670539
32671893
32683199
32693387
32702415
32854965
32893872
32905599
32905705
32922750
2821 2822 2823 2831 2832 2846 2858 2860 2863 2871 2873 2881 2882 2883 2886 2893 2898 2911 2912 2913 2915 2916 2925 2938 2939 2941 2943 2949 2950 2951 2954 2956 2958 2959 2961 2962 2967 2973 2975 2978 2979 2982 2983 2987 2994 2995 2996 2997 2998

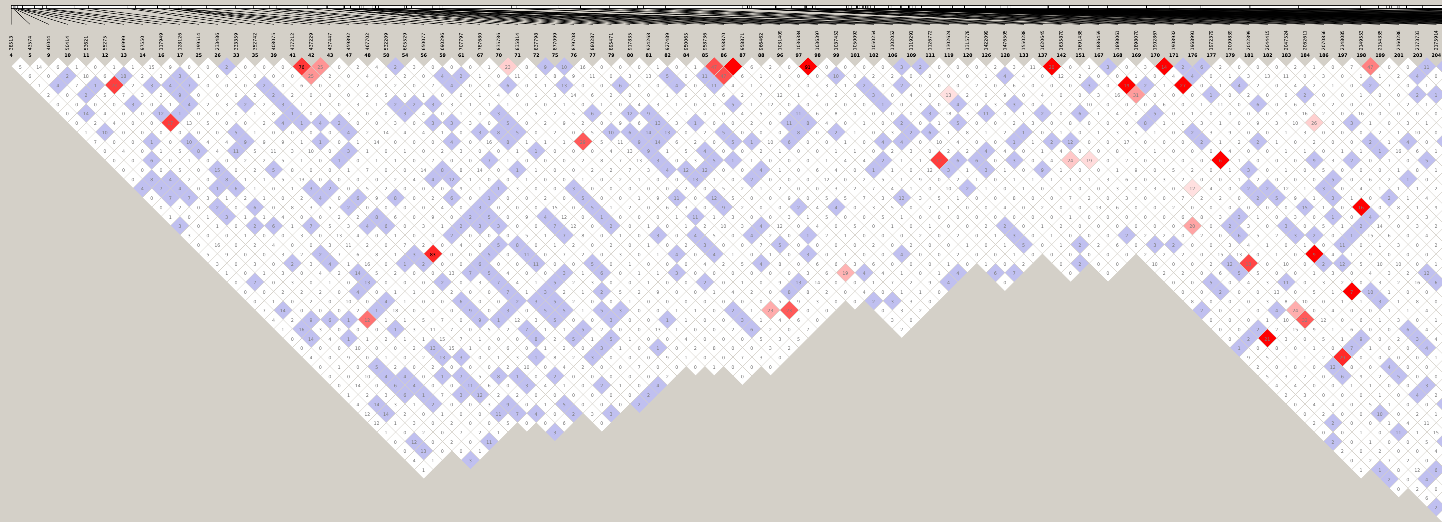

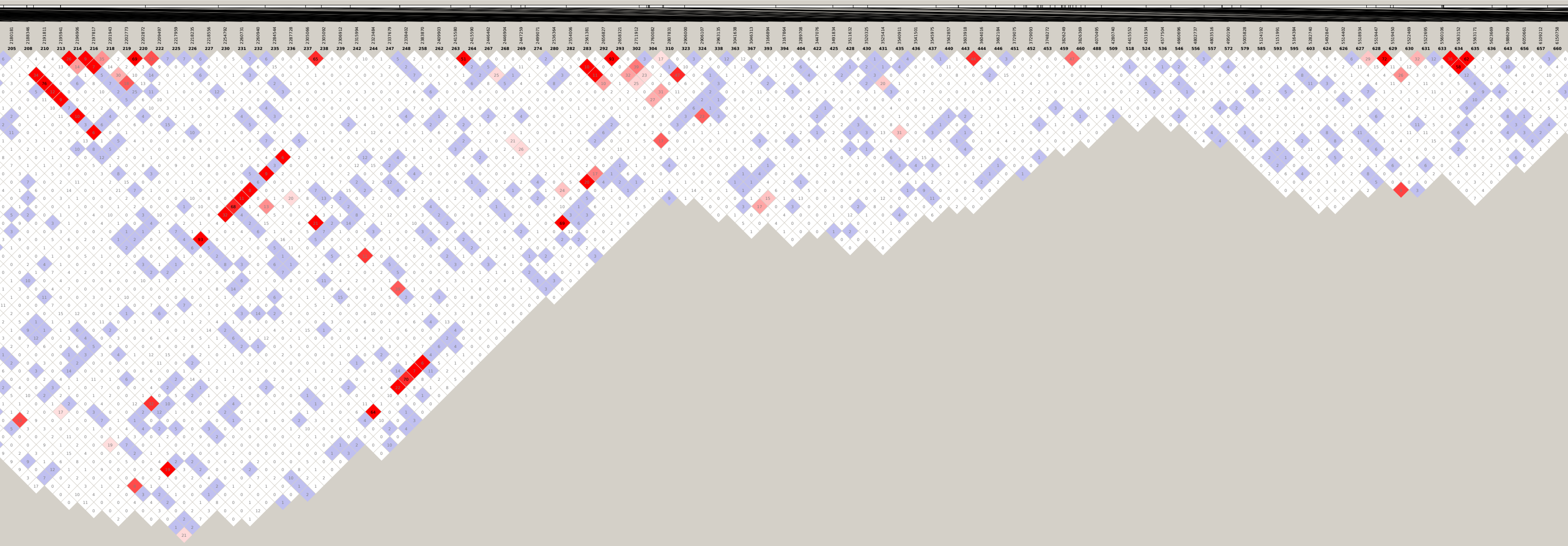

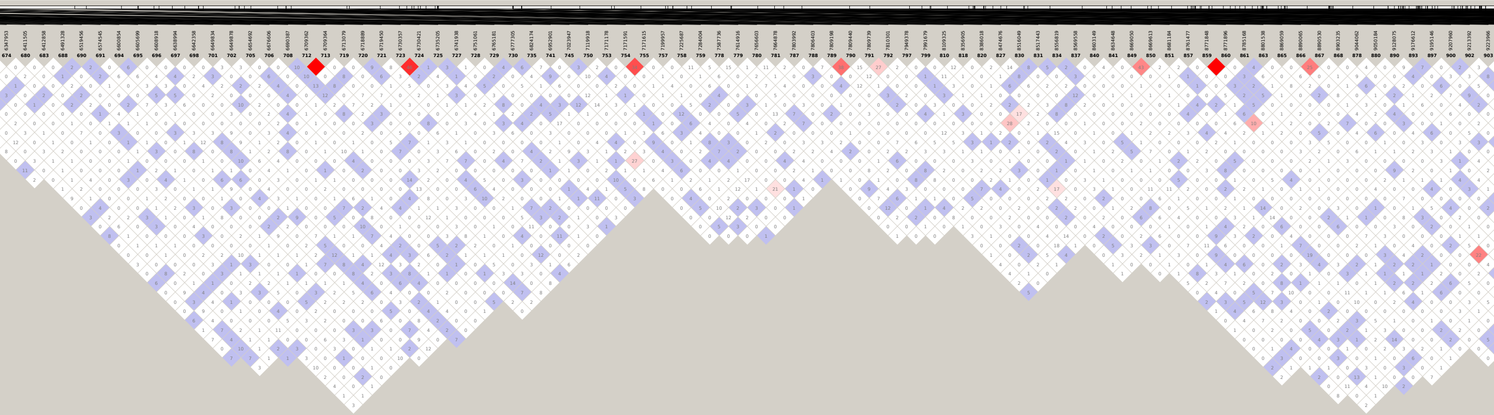

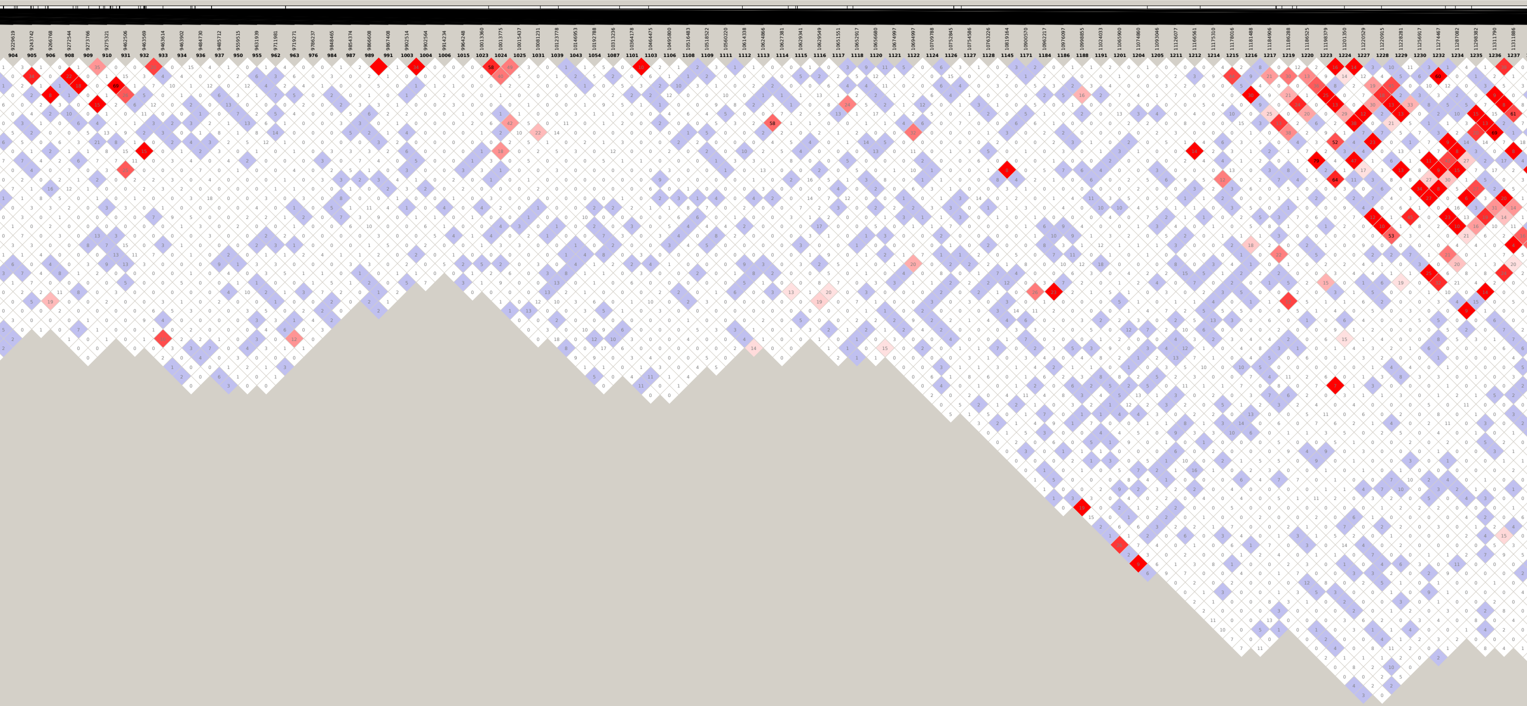

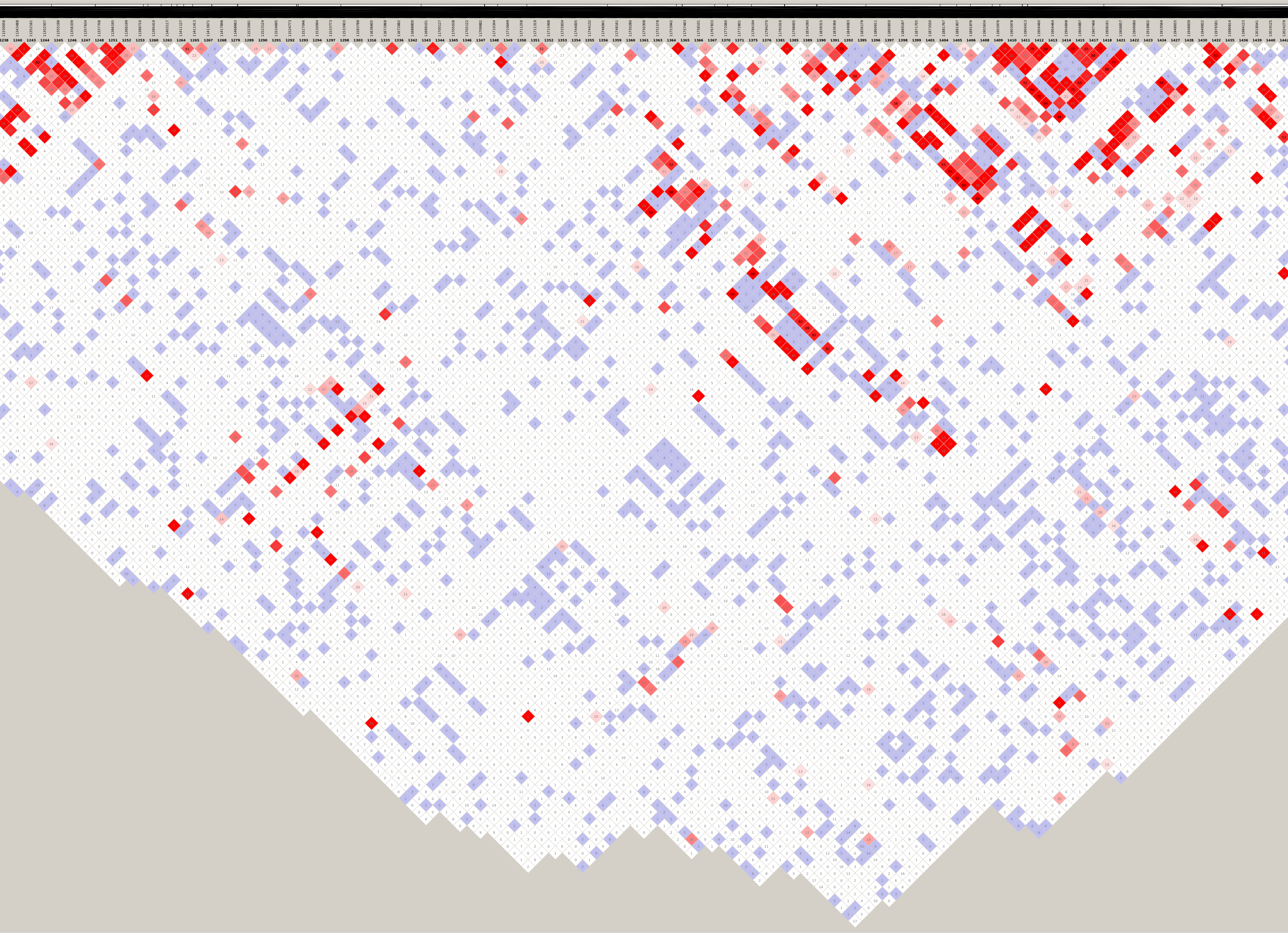

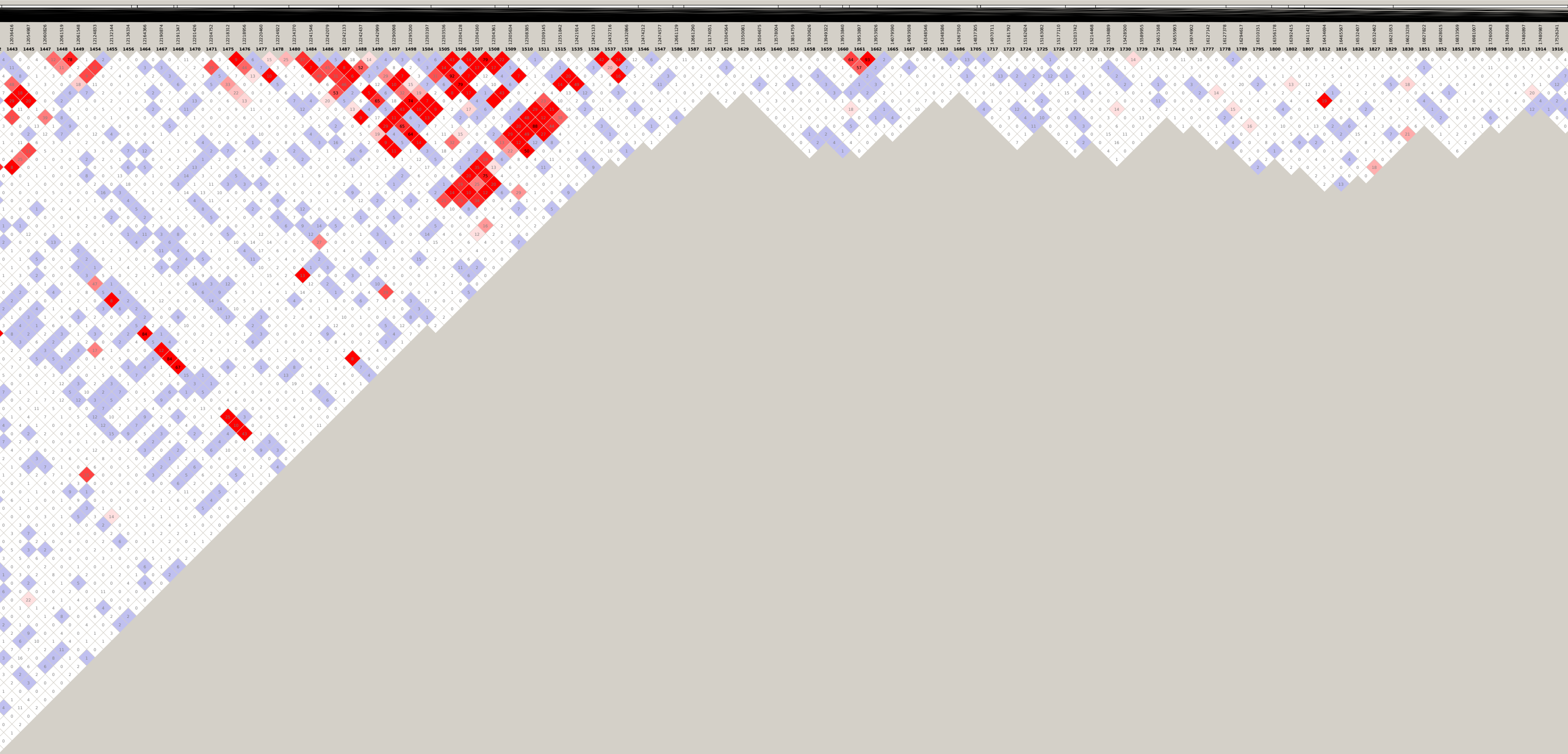

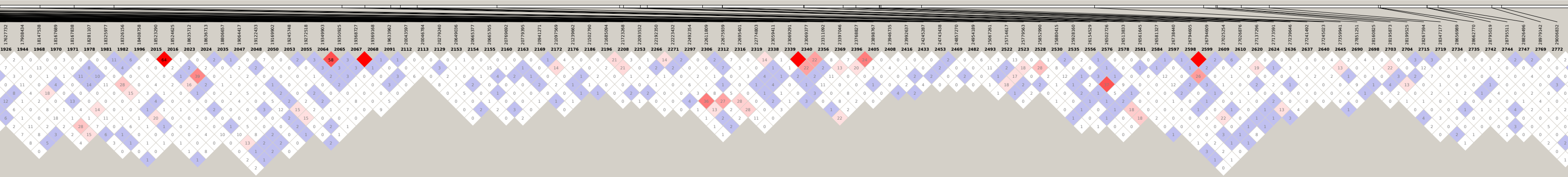

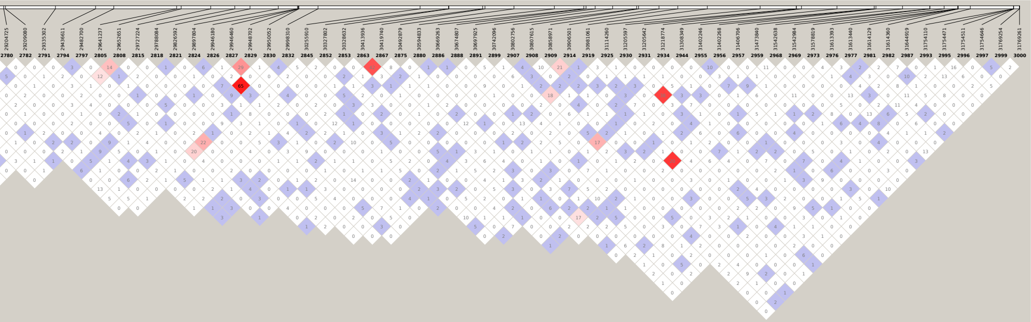

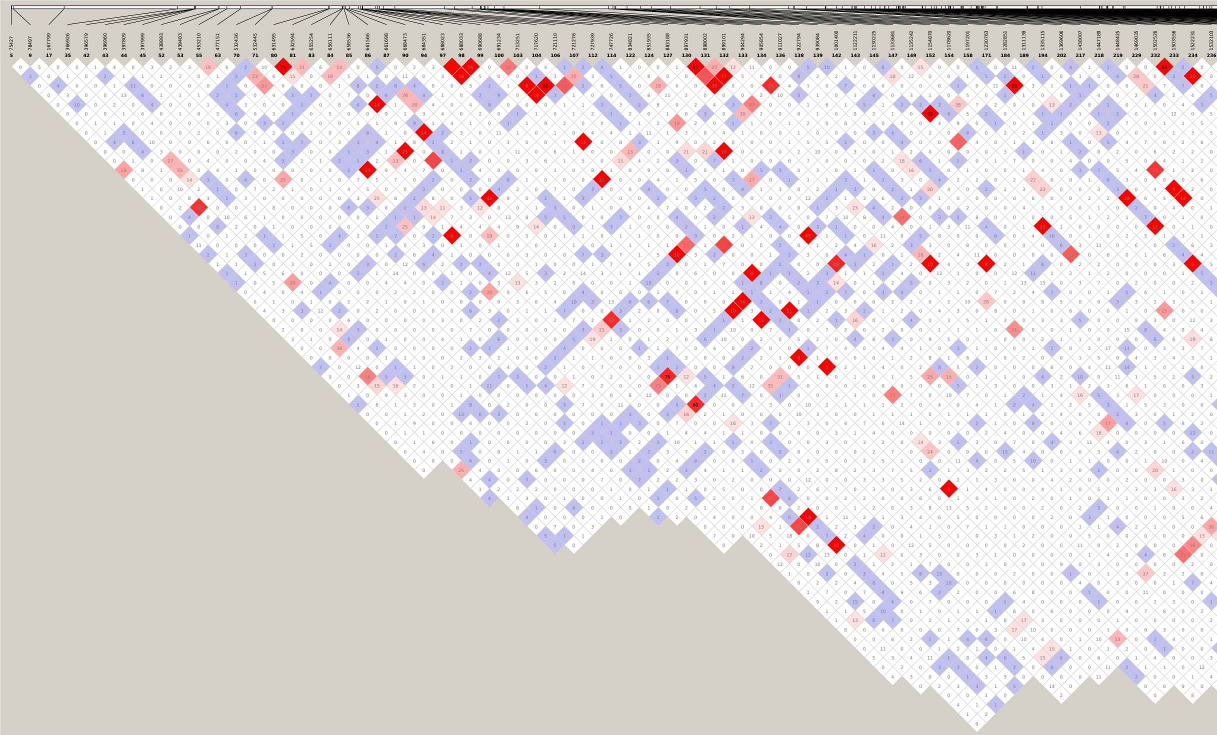
75427 5
78497 9
167799 17
366926 35
396579 42
396960 43
397809 44
397999 45
438893 52
439483 53
453210 55
477151 63
532436 70
532445 71
631495 80
632594 81
655254 83
656111 84
658536 85
661566 86
661698 87
668473 90
684351 94
688023 97
688033 98
690688 99
691234 100
713251 103
717620 104
721110 106
721276 107
727939 112
747726 114
834821 122
851935 124
883188 127
897931 130
898002 131
899101 132
904294 133
905854 134
911027 136
922794 138
939084 139
1001400 142
1122211 143
1130225 145
1133681 147
1135242 149
1154870 152
1178620 154
1197201 158
1230763 171
1282851 184
1311139 189
1335115 194
1369606 202
1438007 217
1447189 218
1448425 219
1469035 229
1502526 232
1503556 233
1522231 234
1532103 236

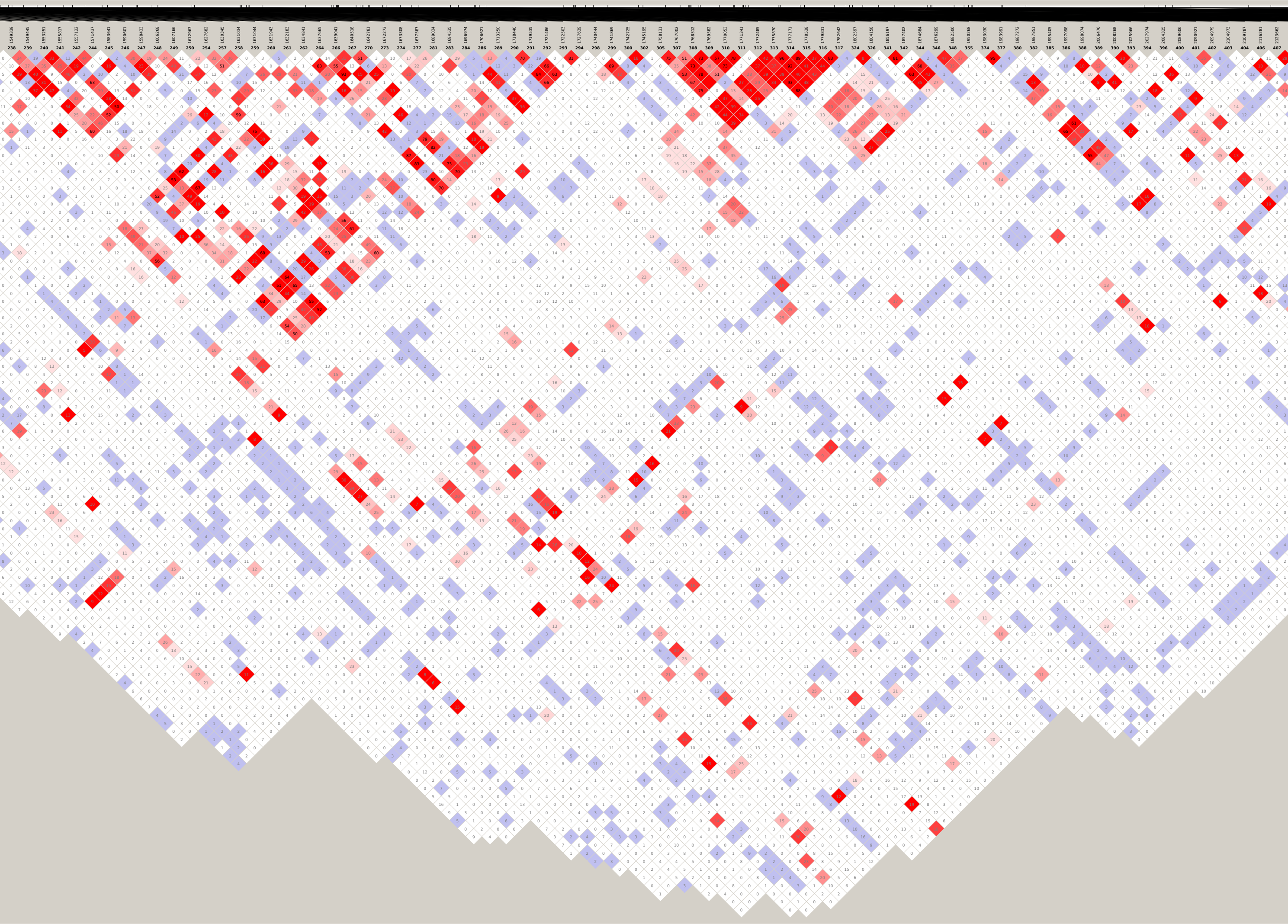

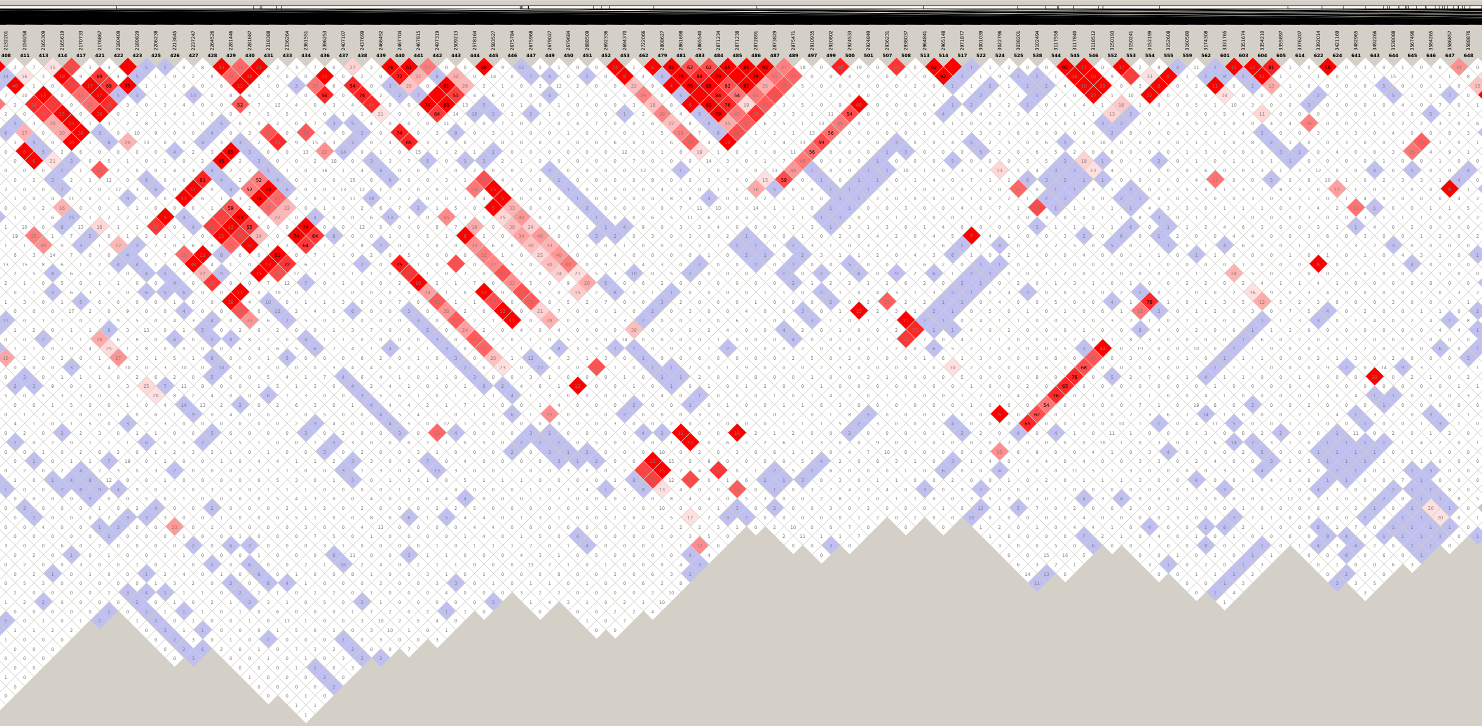
408 411 413 414 417 421 422 423 425 426 427 428 429 430 431 433 434 436 437 438 439 440 441 442 443 444 445 446 447 449 450 451 452 453 462 479 481 483 484 485 486 487 489 497 499 500 501 507 508 513 514 517 522 524 525 538 544 545 546 552 553 554 555 559 562 601 603 604 605 614 622 624 641 643 644 645 646 647 648

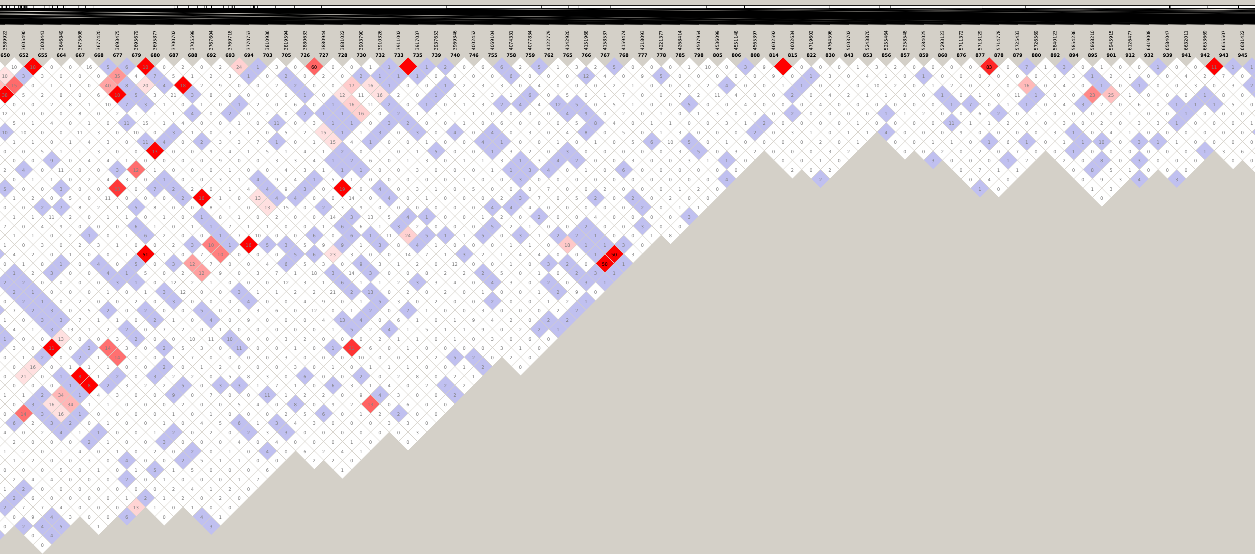
3589922 3605490 3608441 3646849 3675608 3677420 3693475 3695679 3695877 3700702 3705599 3767604 3769718 3770753 3810936 3819594 3880633 3880944 3881022 3903790 3910326 3911002 3917037 3937653 3969346 4002452 4069104 4074331 4077834 4122779 4142920 4151968 4158537 4159474 4218093 4221377 4268414 4507954 4536099 4551148 4565397 4602592 4602634 4719602 4764596 5003702 5243870 5255464 5258548 5284025 5293123 5711372 5713129 5714778 5725433 5726569 5840123 5854236 5868210 5945915 6126477 6419008 6584047 6632011 6653669 6655507 6681422
650 652 655 664 667 668 677 679 680 687 688 692 693 694 703 705 726 727 728 730 732 733 735 739 740 746 755 758 759 762 765 766 767 768 777 778 785 798 805 806 808 814 815 822 830 843 853 856 857 859 860 876 877 878 879 880 892 894 895 901 912 932 939 941 942 943 945

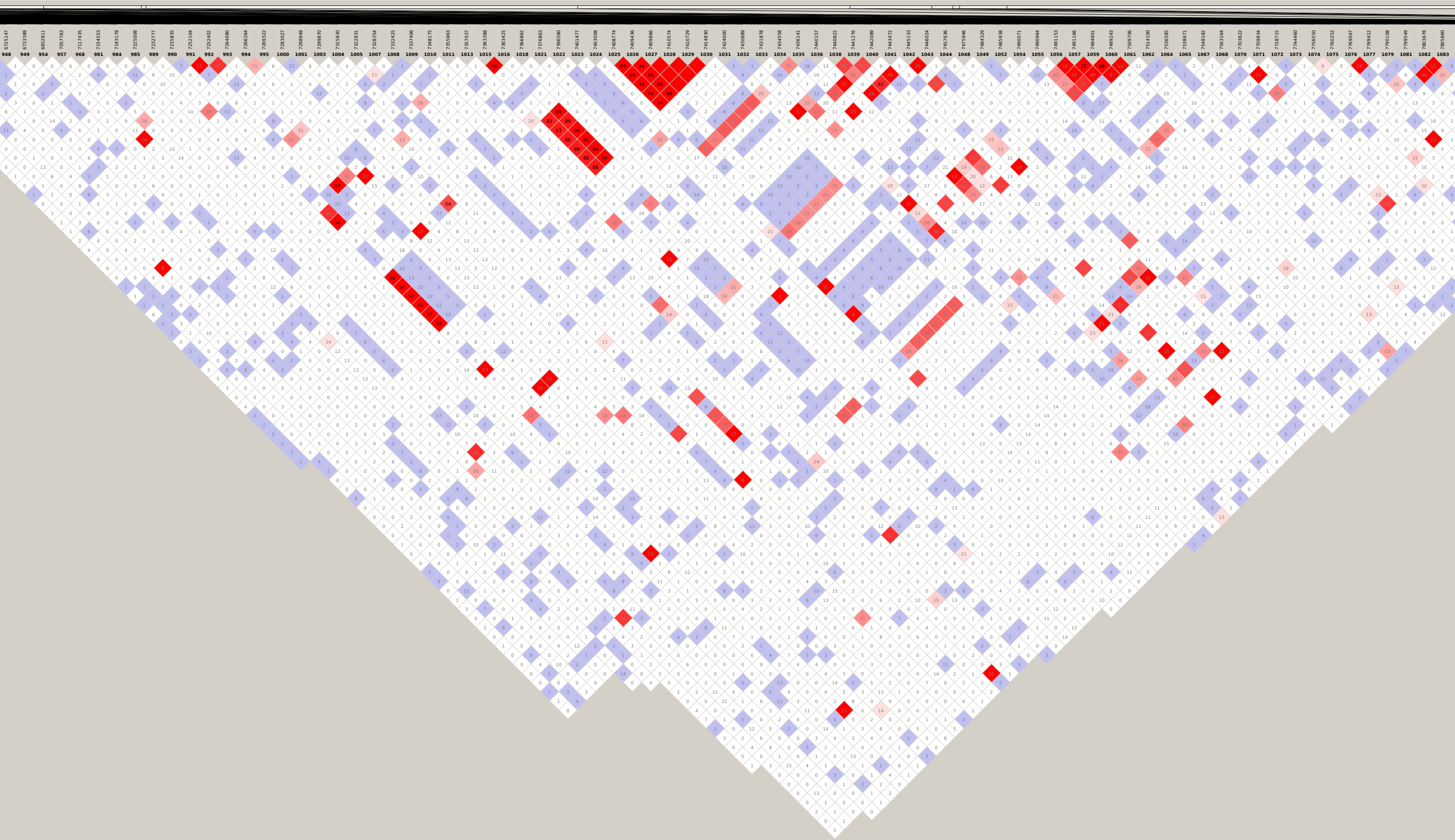

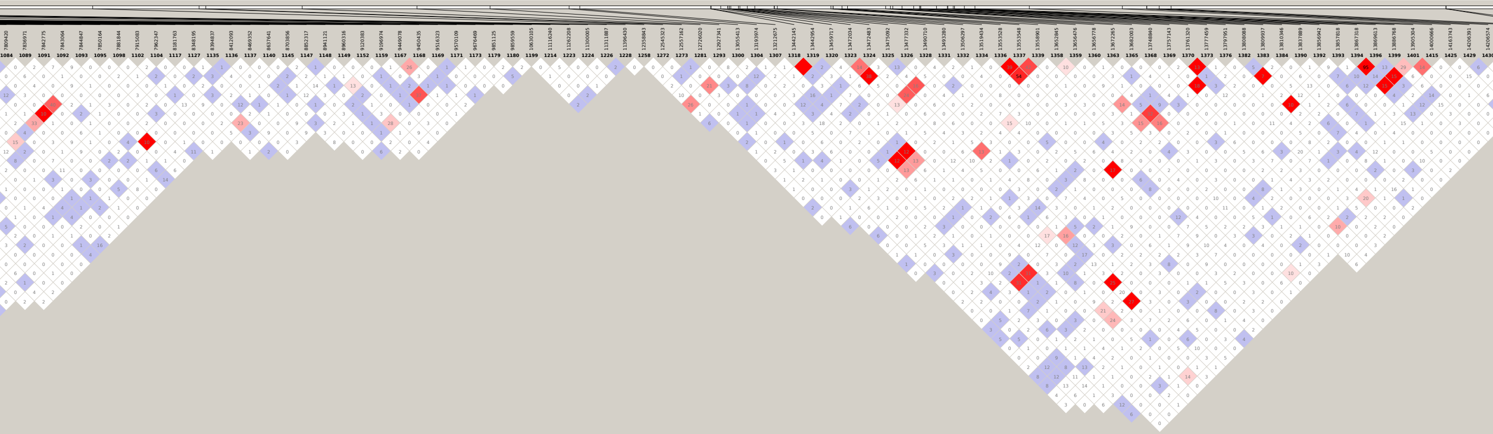

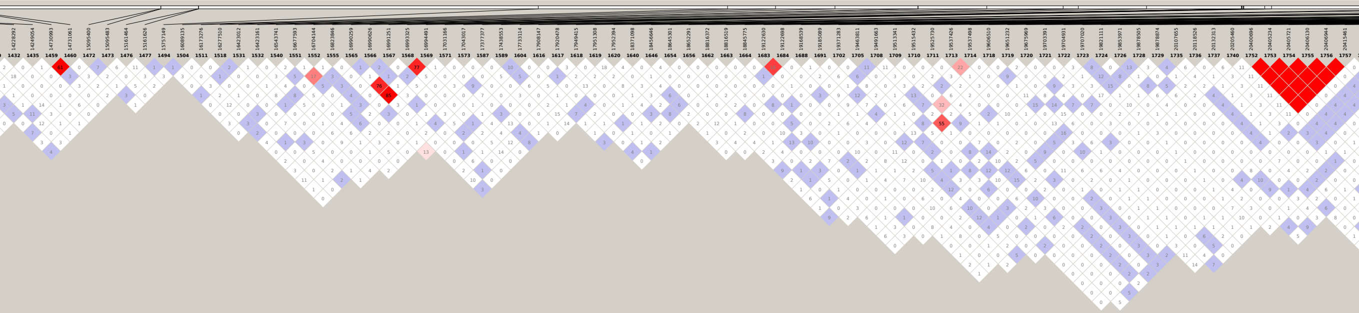

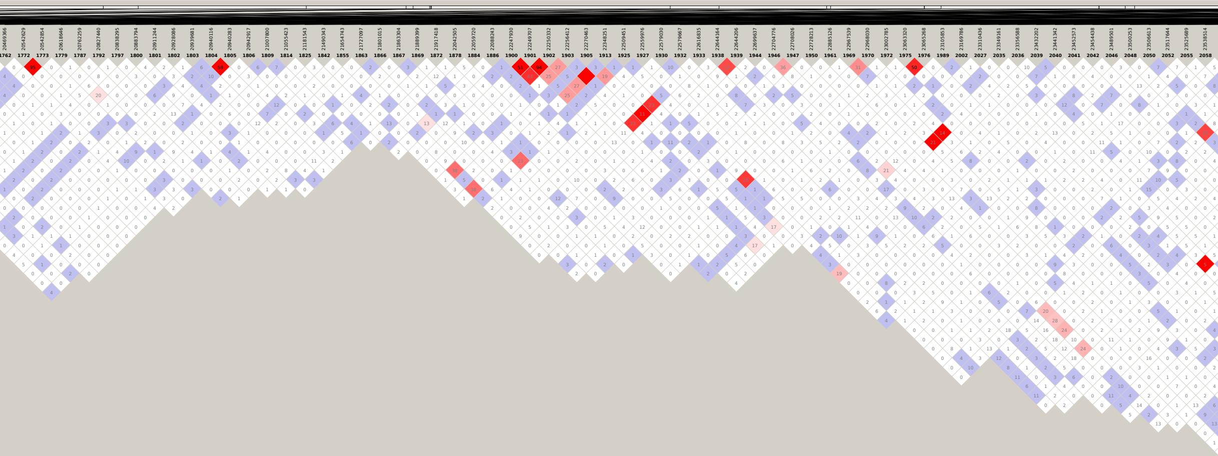

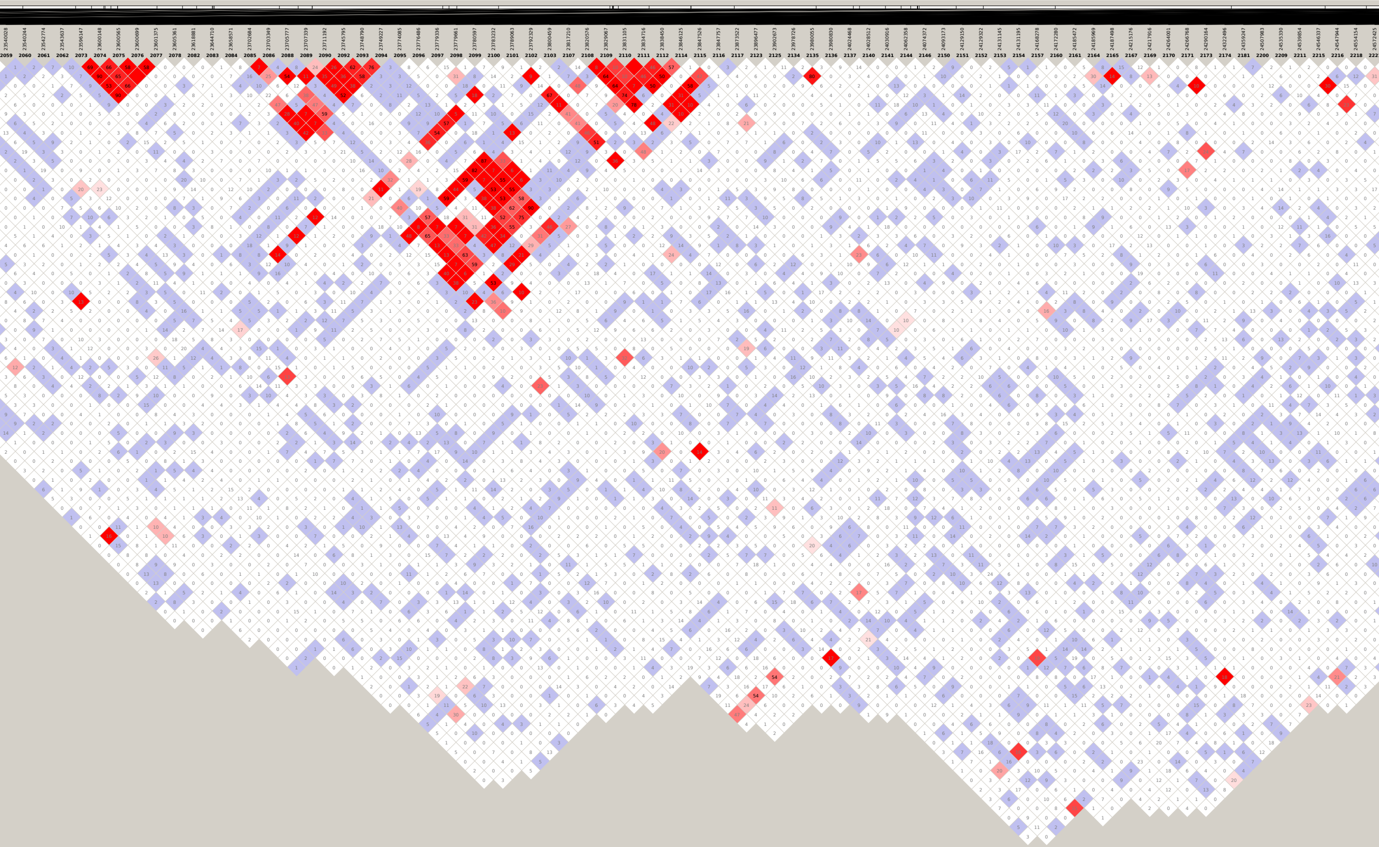

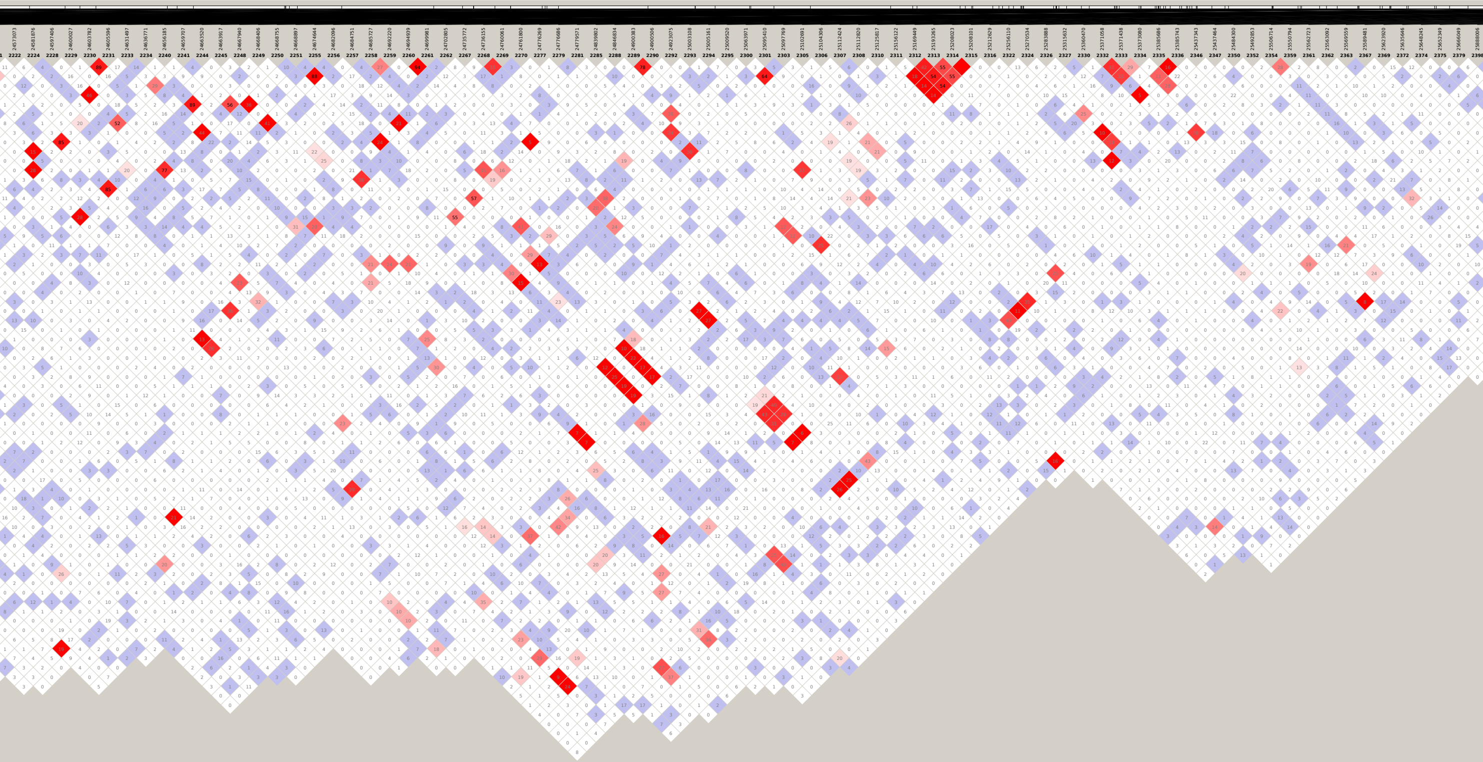

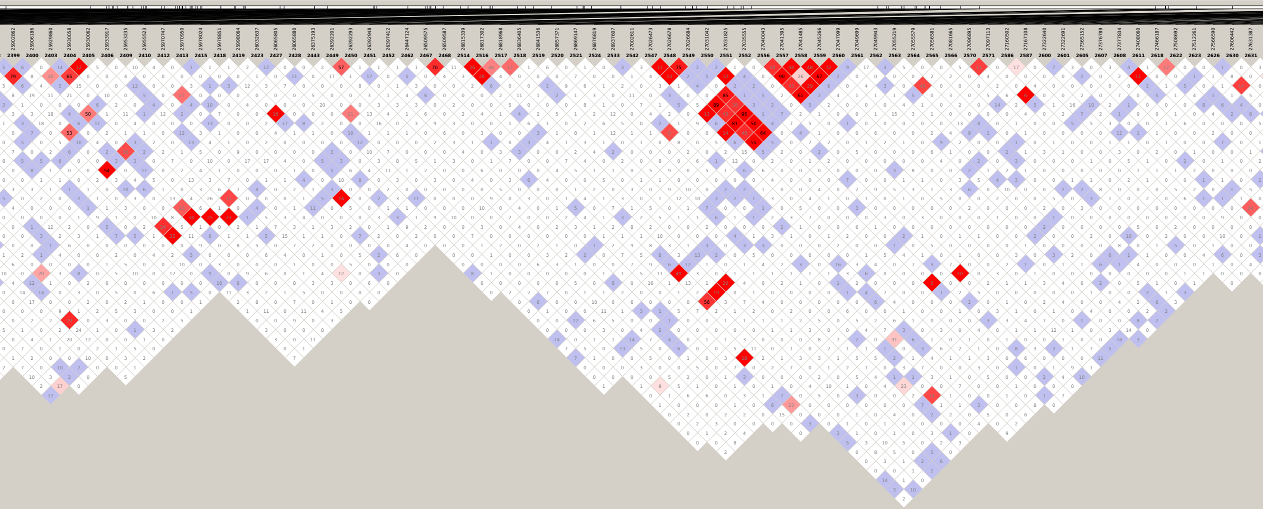
25902862 25906186 25929860 25930058 25930062 25933917 25953235 25955523 25970747 25970950 25978024 25978851 25998064 26032637 26065805 26065880 26375193 26392201 26392293 26392948 26397412 26447124 26509575 26509587 26815339 26817302 26818968 26836405 26841536 26857371 26869147 26876819 26937607 27002611 27026473 27026678 27026684 27031042 27031825 27035555 27040043 27041395 27041485 27045266 27047999 27049699 27049943 27055219 27055579 27056581 27081665 27096895 27097113 27160502 27167108 27322640 27322691 27365152 27376789 27377834 27408060 27466187 27508692 27512261 27566590 27606442 27631387
2399 2400 2403 2404 2405 2406 2409 2410 2412 2413 2415 2418 2419 2423 2427 2428 2443 2449 2450 2451 2452 2462 2467 2468 2514 2516 2517 2518 2519 2520 2521 2524 2533 2542 2547 2548 2549 2550 2551 2552 2556 2557 2558 2559 2560 2561 2562 2563 2564 2565 2566 2570 2571 2586 2587 2600 2601 2605 2607 2608 2611 2618 2622 2623 2626 2630 2631

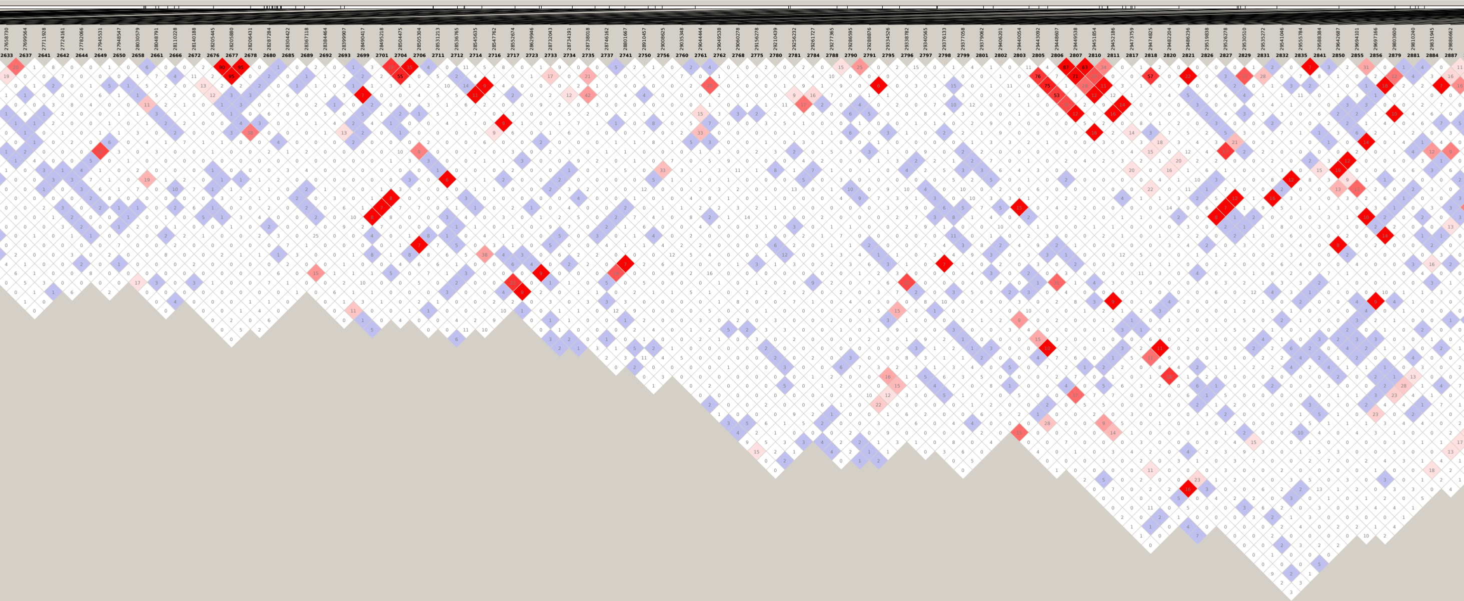

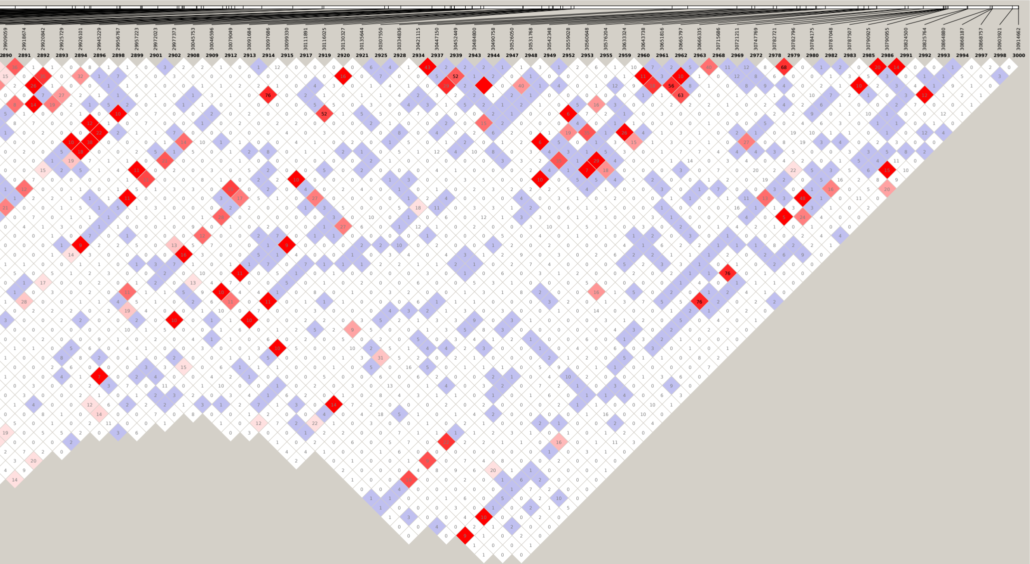
29909059 29918674 29920842 29925729 29926101 29945229 29956767 29957223 29972023 29977373 30045753 30046596 30079049 30091684 30097686 30099330 30111891 30116025 30130327 30135644 30307550 30334836 30421115 30447150 30452449 30484800 30490758 30526050 30531768 30542348 30559028 30560648 30576204 30633324 30643738 30651816 30665797 30666335 30715686 30721211 30747769 30782721 30782796 30784175 30787048 30787507 30790925 30790955 30824500 30825764 30864880 30868187 30868757 30903921 30914662
2890 2891 2892 2893 2894 2896 2898 2899 2901 2902 2908 2909 2912 2913 2914 2915 2917 2919 2920 2921 2925 2928 2934 2937 2939 2943 2944 2947 2948 2949 2952 2953 2955 2959 2960 2961 2962 2963 2968 2969 2972 2978 2979 2980 2982 2983 2985 2986 2991 2992 2993 2994 2997 2998 3000

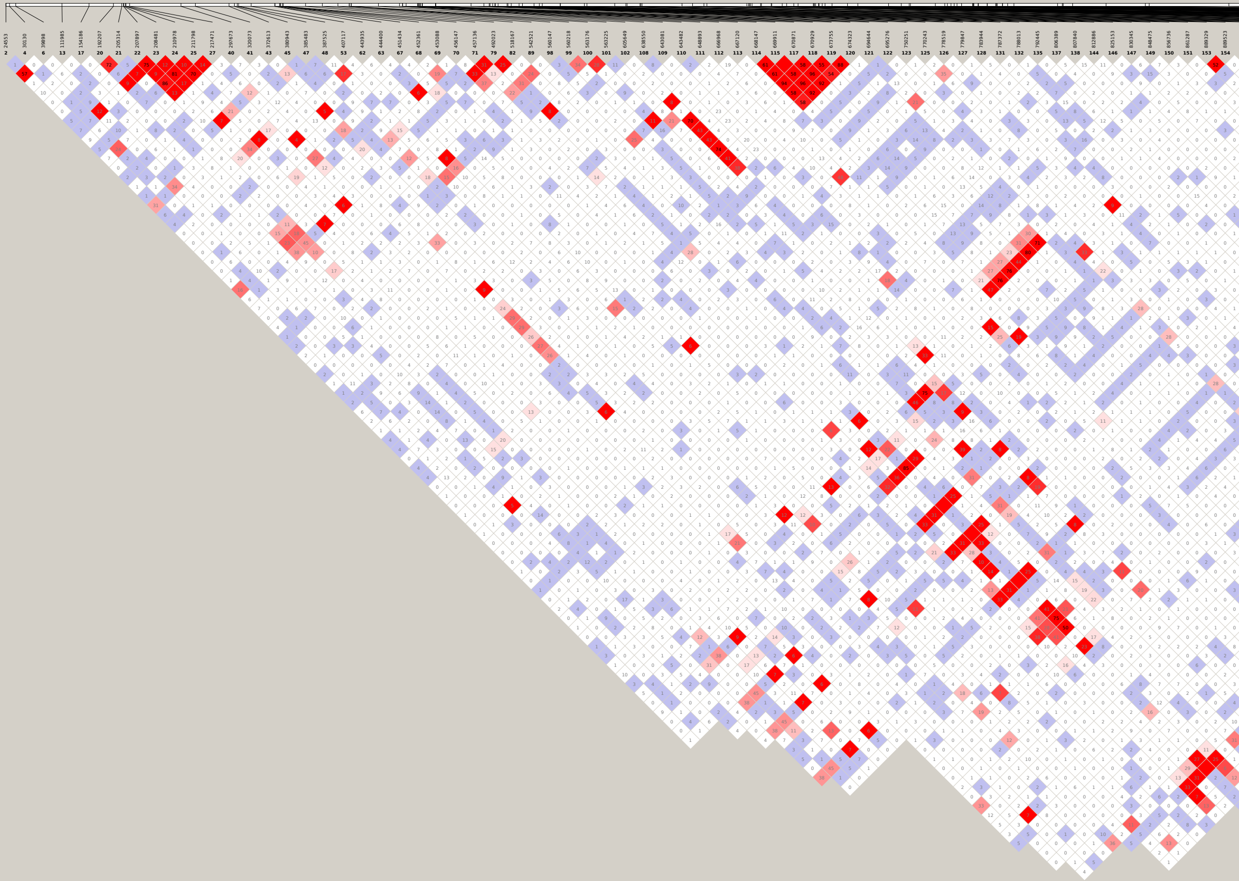

24553 2
30130 4
39898 6
111985 13
154186 17
192207 20
205314 21
207897 22
208481 23
210978 24
211798 25
217471 27
297673 40
320073 41
372613 43
380943 45
385483 47
387525 48
407117 53
443935 62
444400 63
451434 67
452361 68
453088 69
456147 70
457136 71
492023 79
518167 82
542521 89
560147 98
560218 99
563176 100
563225 101
605649 102
638550 108
643081 109
643482 110
648893 111
666968 112
667120 113
668147 114
669911 115
670871 117
670929 118
673755 119
674323 120
694644 121
695276 122
750251 123
770243 125
778519 126
779847 127
783944 128
787372 131
788013 132
792445 135
806389 137
807840 138
812886 144
825153 146
830345 147
848475 149
856736 150
861287 151
889329 153
889523 154

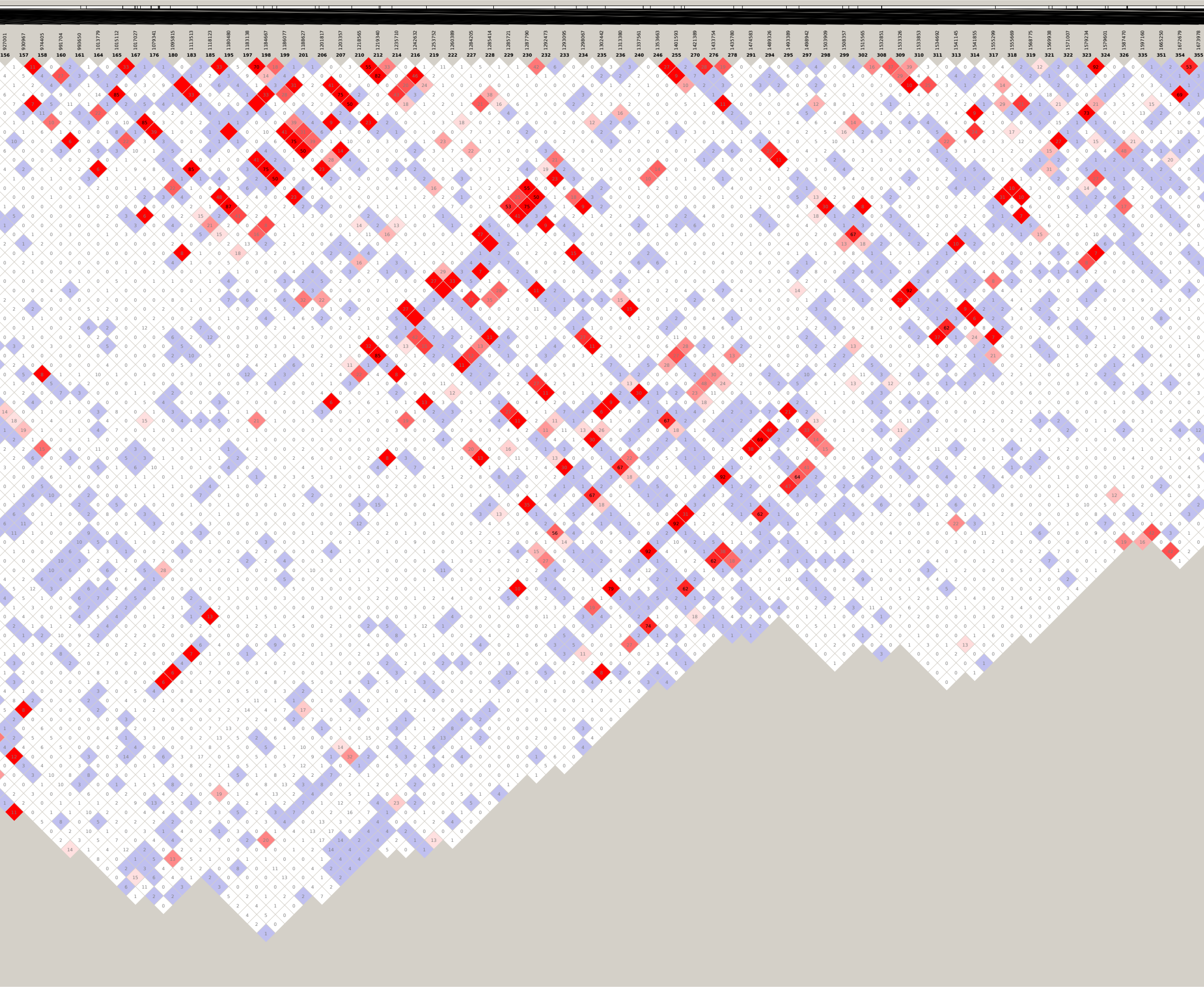
927001 939967 974405 991704 993650 1013779 1015112 1017027 1079341 1095815 1113513 1118123 1180480 1183138 1184667 1186077 1188627 1201817 1203357 1218565 1219340 1235710 1242632 1253752 1260389 1284205 1285414 1285721 1287790 1292473 1293095 1298067 1302442 1313380 1337561 1353663 1401593 1421389 1433754 1435780 1474583 1489326 1493389 1498942 1503909 1508357 1515565 1532851 1533326 1533853 1534692 1541145 1541855 1555299 1555669 1568775 1569938 1571007 1579234 1579601 1587470 1597160 1665250 1672679 1673978
156 157 158 160 161 164 165 167 176 180 183 185 195 197 198 199 201 206 207 210 212 214 216 219 222 227 228 229 230 232 233 234 235 236 240 246 255 270 276 278 291 294 295 297 298 299 302 308 309 310 311 313 314 317 318 319 321 322 323 324 326 335 351 354 355

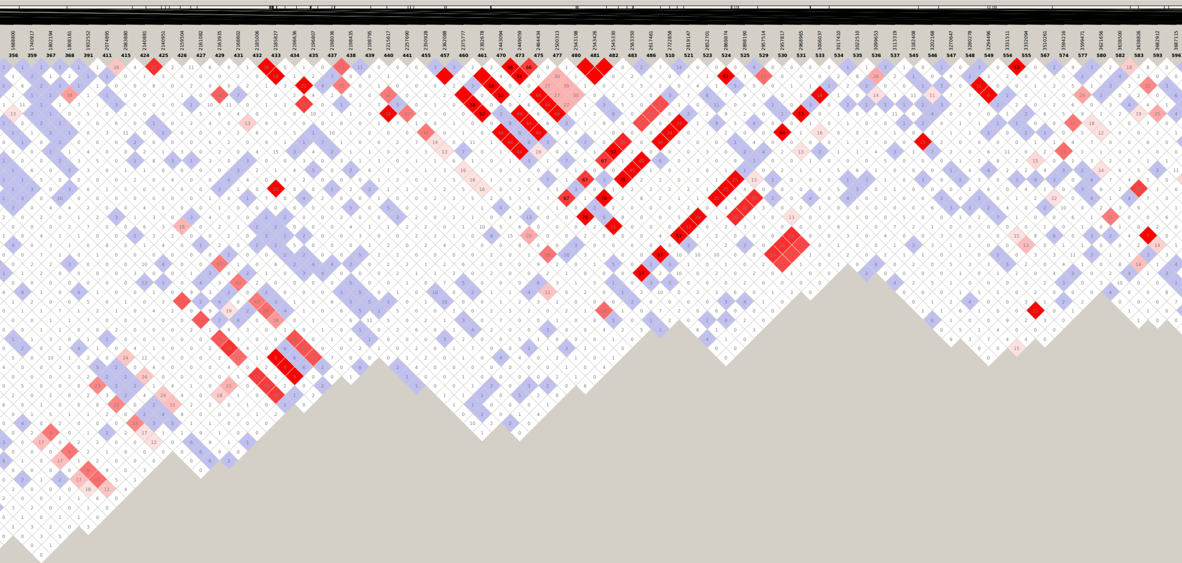

1688800 356
1740917 359
1802194 367
1808161 368
1932552 391
2074895 411
2083880 415
2140881 424
2140951 425
2159504 426
2161082 427
2163935 429
2168802 431
2185006 432
2185827 433
2196636 434
2196807 435
2198036 437
2198435 438
2198795 439
2215617 440
2257690 441
2350928 455
2362088 457
2375777 460
2382678 461
2443094 470
2449059 473
2464434 475
2500313 477
2543198 480
2543426 481
2545330 482
2563350 483
2617461 486
2722858 510
2819147 521
2852701 523
2869874 524
2898190 525
2957514 529
2957817 530
2968965 531
3006037 533
3017410 534
3022510 535
3099653 536
3113319 537
3182408 545
3202168 546
3270647 547
3280278 548
3294496 549
3331511 554
3332094 555
3510261 567
3594216 574
3599471 577
3621656 580
3638500 582
3638826 583
3682912 593
3687115 596

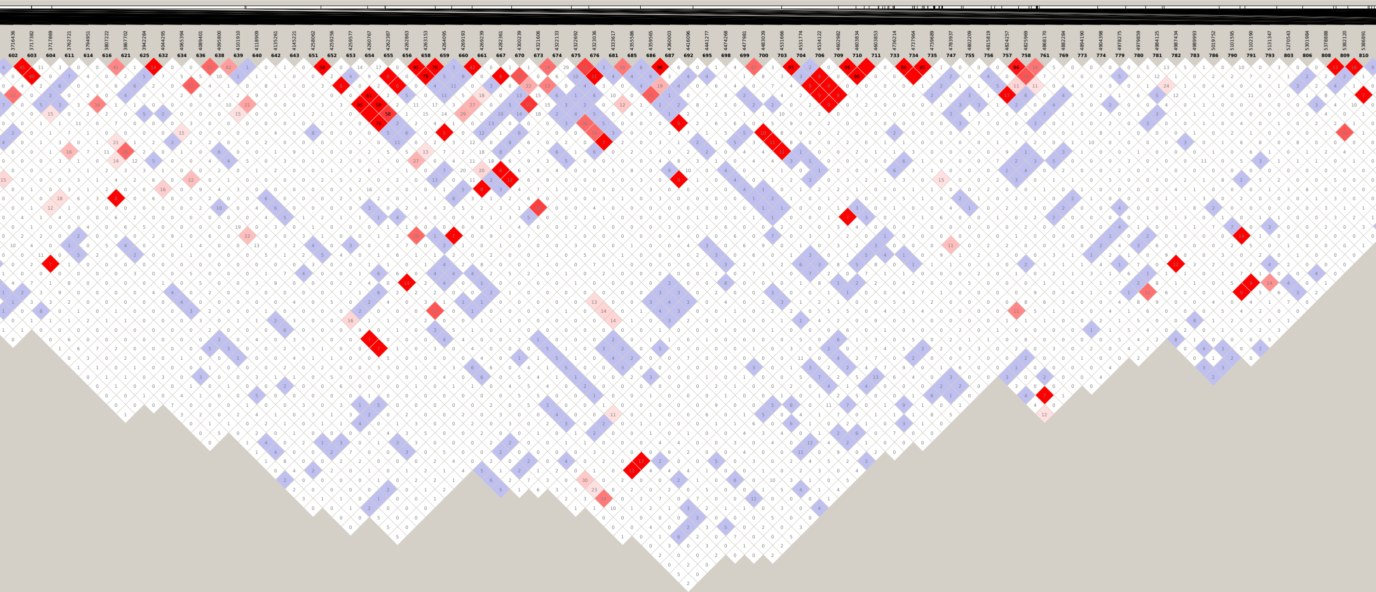
3716436 3717382 3717869 3762721 3794951 3807222 3867702 3942284 4044295 4065394 4089401 4095800 4101910 4118909 4135261 4145221 4258062 4259256 4259577 4260767 4262387 4262863 4263153 4264095 4269193 4269239 4282361 4300239 4321606 4322133 4322692 4323036 4333617 4355586 4359565 4360003 4416096 4441277 4474268 4477981 4483039 4531666 4531774 4534122 4602982 4603834 4603853 4736214 4737964 4739689 4783937 4802209 4815819 4824257 4825969 4868170 4882284 4894190 4904398 4978275 4979859 4984125 4987434 4989993 5019752 5101595 5102190 5131347 5270543 5301984 5378888 5382120 5386891
602 603 604 611 614 616 621 625 632 634 636 638 639 640 642 643 651 652 653 654 655 656 658 659 660 661 667 670 673 674 675 676 681 685 686 687 692 695 698 699 700 703 704 706 709 710 711 733 734 735 747 755 756 757 758 761 769 773 774 779 780 781 782 783 786 790 791 793 803 806 808 809 810

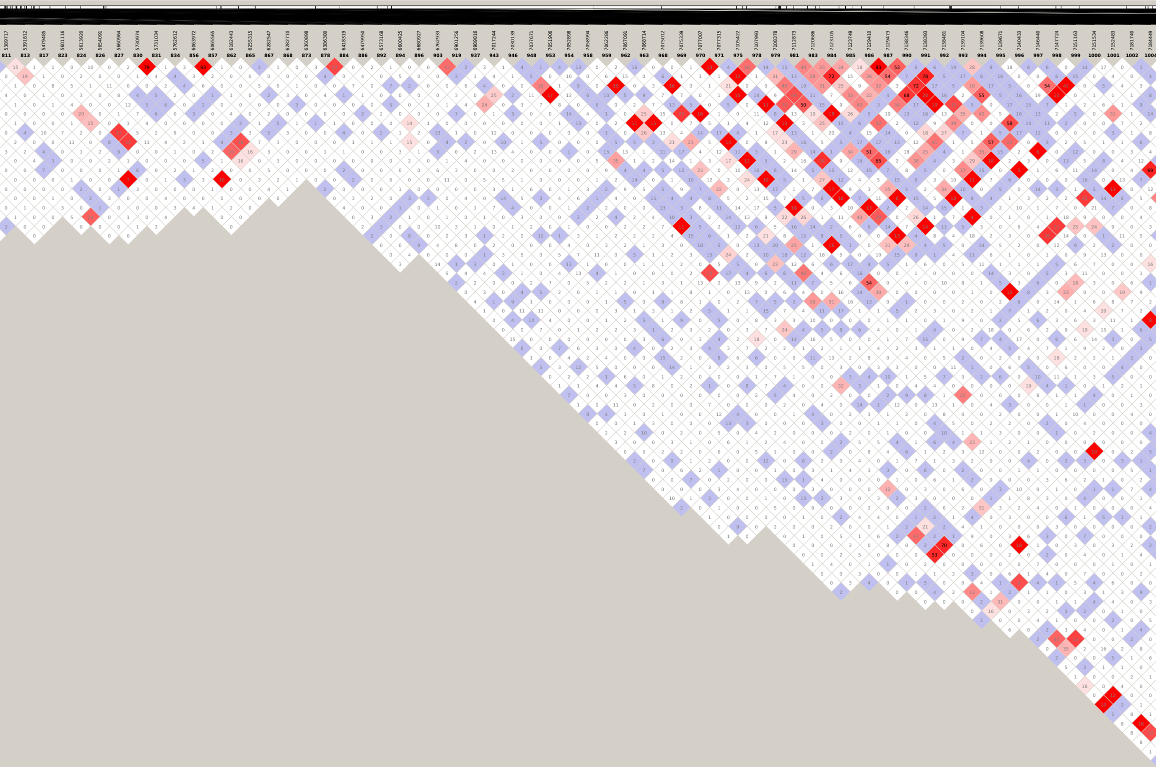

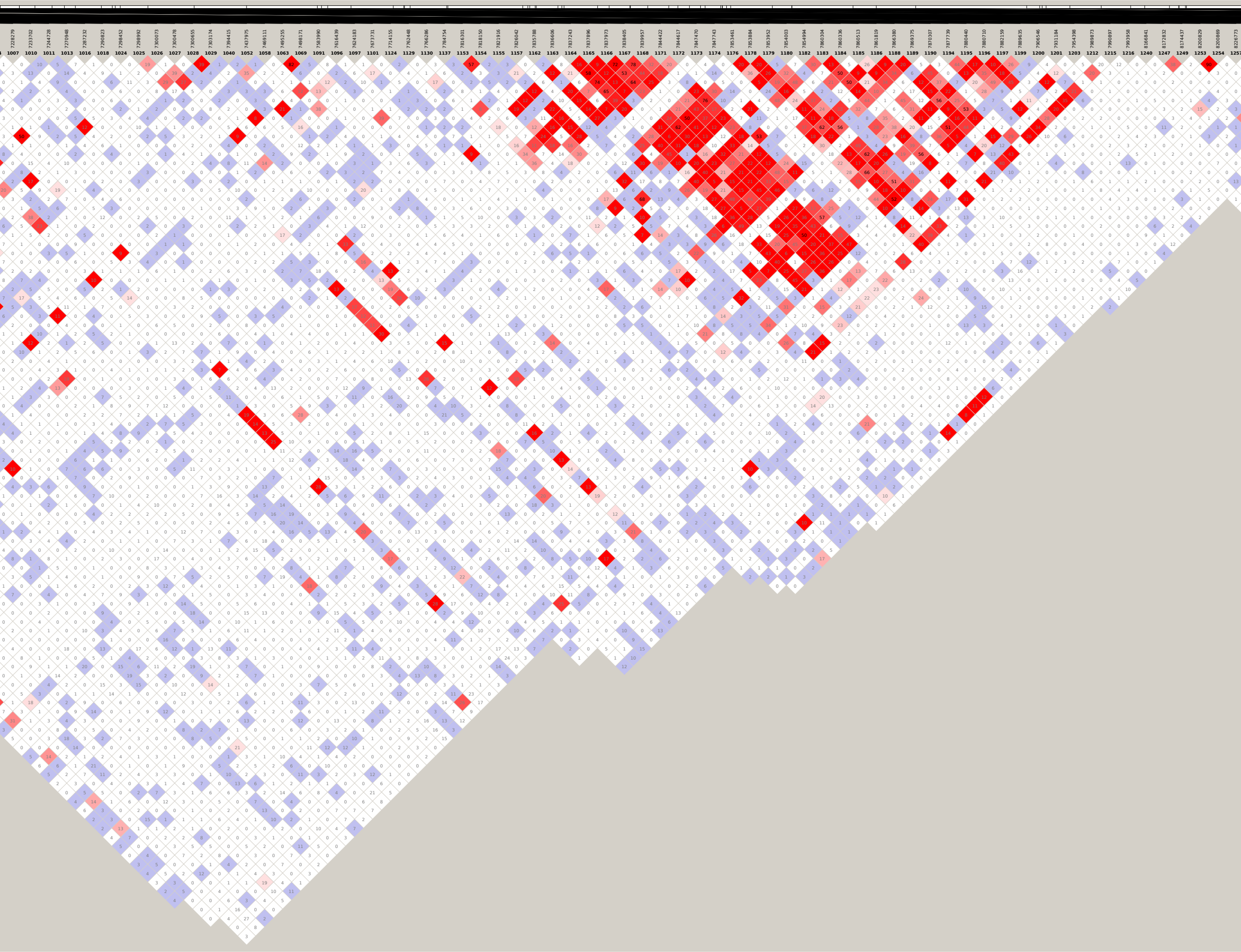

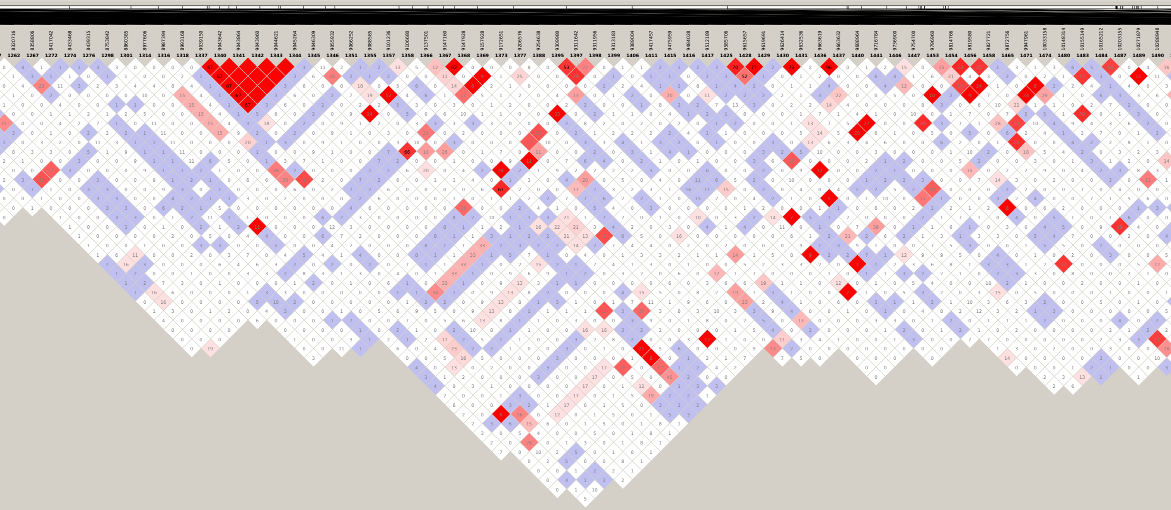
8320716 8358806 8417042 8433468 8439315 8753842 8860385 8977606 8987394 8993168 9039150 9043642 9043864 9043960 9044621 9045264 9046309 9055932 9066252 9088585 9101236 9106680 9137501 9147160 9147928 9157928 9172651 9206576 9254638 9309980 9311642 9311956 9313183 9389004 9417457 9475959 9484028 9512189 9585706 9615657 9619691 9626414 9632536 9663619 9663632 9688964 9716784 9736900 9754700 9796960 9814766 9819580 9827721 9872756 9947961 10033158 10148314 10155149 10165212 10203155 10271879 10288948
1262 1267 1272 1274 1276 1298 1301 1314 1316 1318 1337 1340 1341 1342 1343 1344 1345 1346 1351 1355 1357 1358 1366 1367 1368 1369 1373 1377 1388 1395 1397 1398 1399 1406 1411 1415 1416 1417 1425 1428 1429 1430 1431 1436 1437 1440 1441 1446 1447 1453 1454 1456 1458 1465 1471 1474 1480 1483 1484 1487 1489 1490

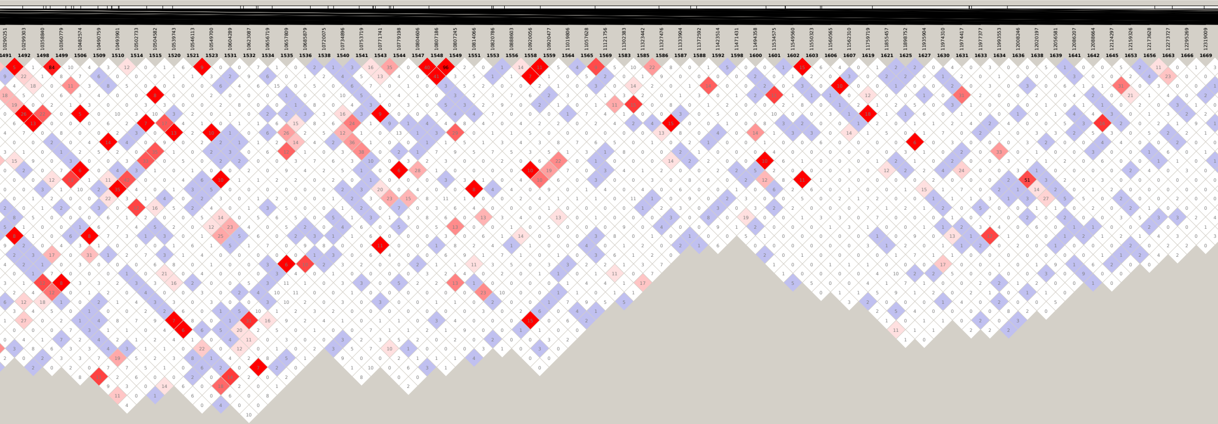
10290251
10299303
10358840
10360779
10482574
10490759
10493901
10502733
10504582
10539743
10546113
10549700
10604289
10623087
10656719
10657809
10685879
10720075
10734896
10753719
10771741
10779198
10804606
10807186
10807245
10814066
10820786
10888603
10920056
10920477
11019806
11057628
11121756
11302383
11323442
11327476
11333904
11372592
11423514
11471431
11494358
11534575
11549560
11550323
11560565
11562310
11759719
11855457
11898752
11935904
11974310
11974417
11977377
11993553
12008246
12020197
12056581
12086207
12088664
12124297
12159326
12173628
12273727
12295269
12319009
1491 1492 1498 1499 1506 1509 1510 1514 1515 1520 1521 1523 1531 1532 1534 1535 1536 1538 1540 1541 1543 1544 1547 1548 1549 1551 1553 1556 1558 1559 1564 1565 1569 1583 1585 1586 1587 1588 1592 1598 1600 1601 1602 1603 1606 1607 1619 1621 1625 1627 1630 1631 1633 1634 1636 1638 1639 1641 1642 1645 1653 1656 1663 1666 1669

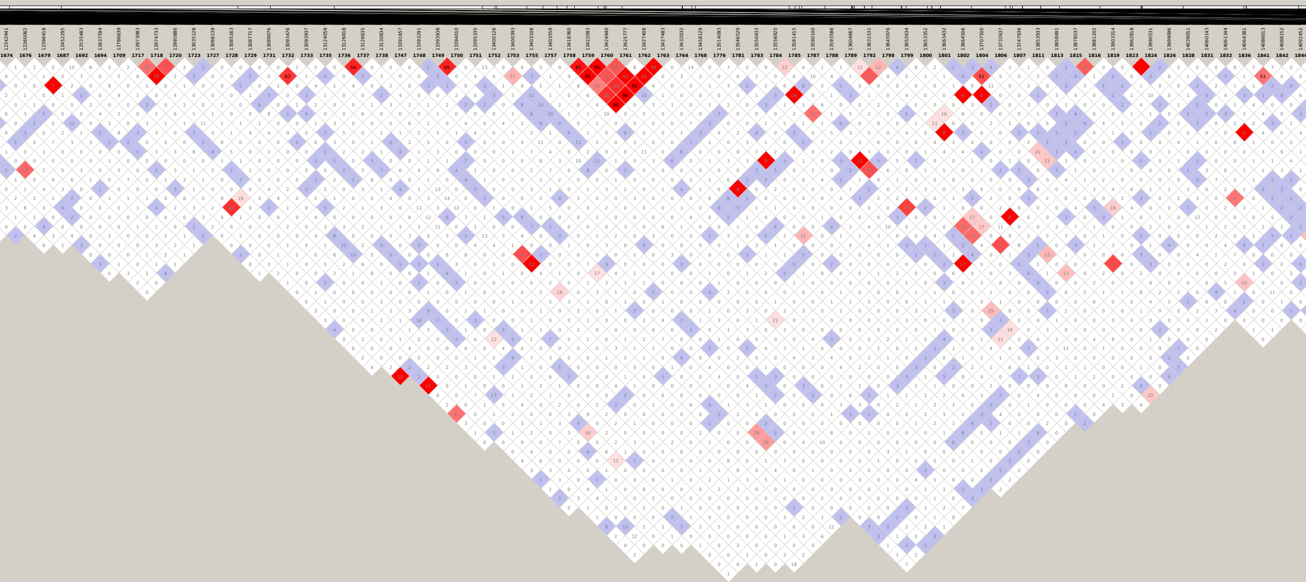

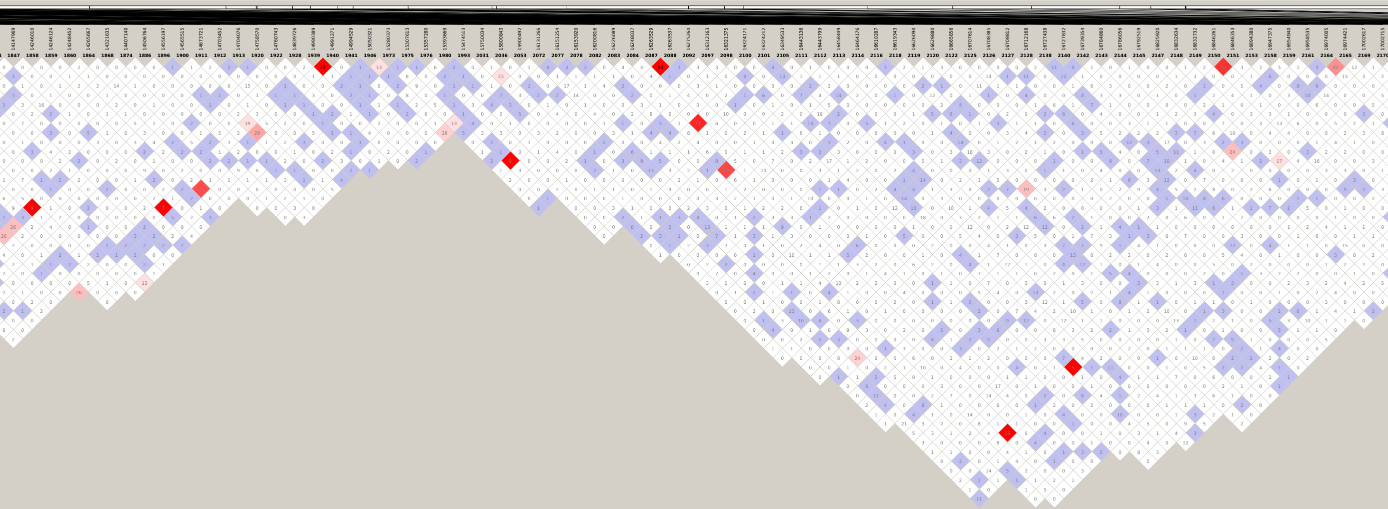

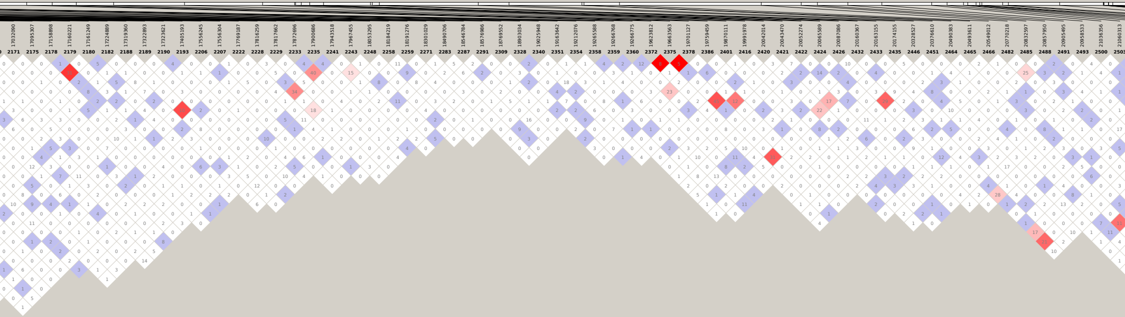
2171 2175 2178 2179 2180 2182 2188 2189 2190 2193 2206 2207 2222 2228 2229 2233 2235 2241 2243 2248 2258 2259 2271 2283 2287 2291 2309 2328 2340 2351 2354 2358 2359 2360 2372 2375 2378 2386 2401 2416 2420 2421 2422 2424 2426 2432 2433 2435 2446 2451 2464 2465 2466 2482 2485 2488 2491 2493 2500

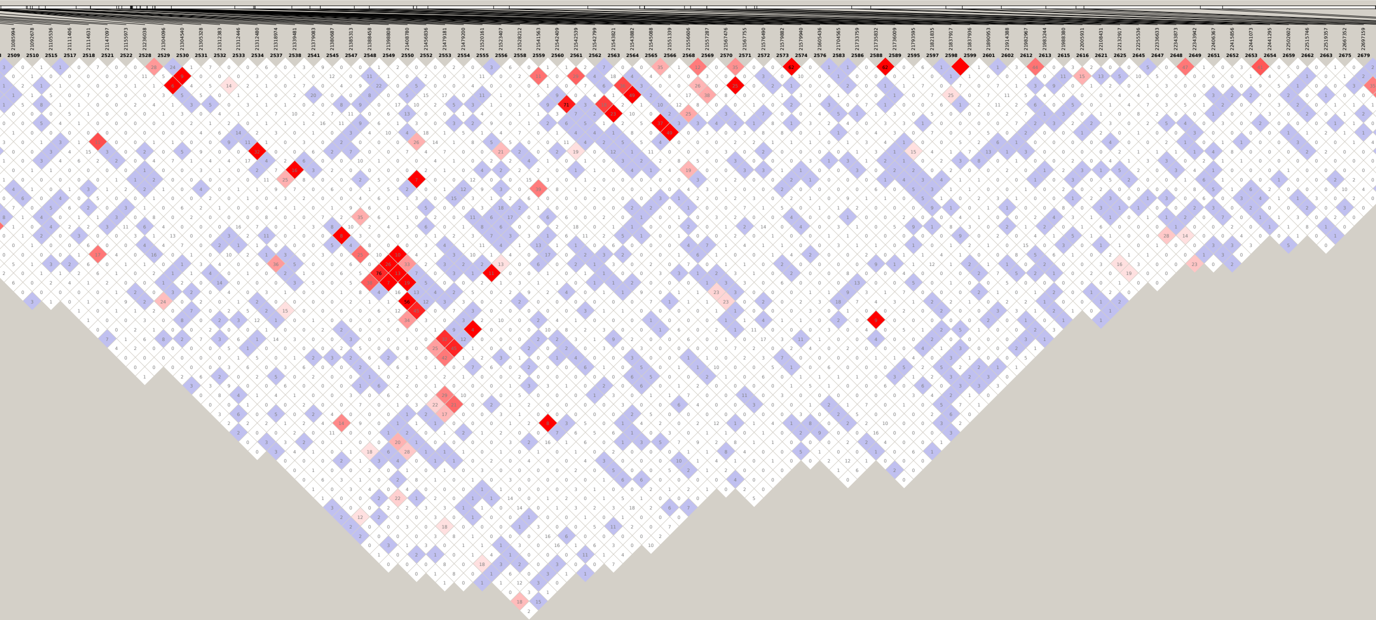

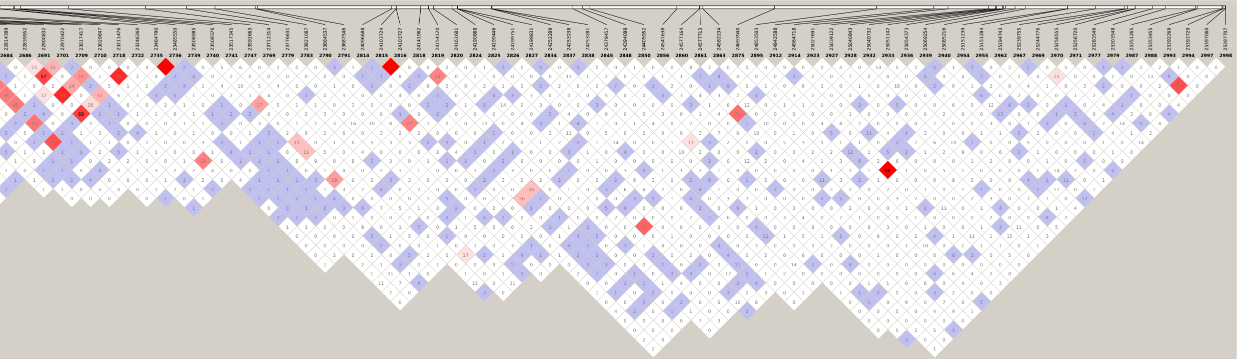
2684 2686 2691 2701 2709 2710 2718 2722 2735 2736 2739 2740 2741 2747 2769 2779 2783 2790 2791 2814 2815 2816 2818 2819 2820 2824 2825 2826 2827 2834 2837 2838 2845 2848 2850 2856 2860 2861 2863 2875 2895 2912 2914 2923 2927 2928 2932 2933 2936 2939 2940 2954 2955 2962 2967 2969 2970 2971 2977 2979 2987 2988 2993 2994 2997 2998

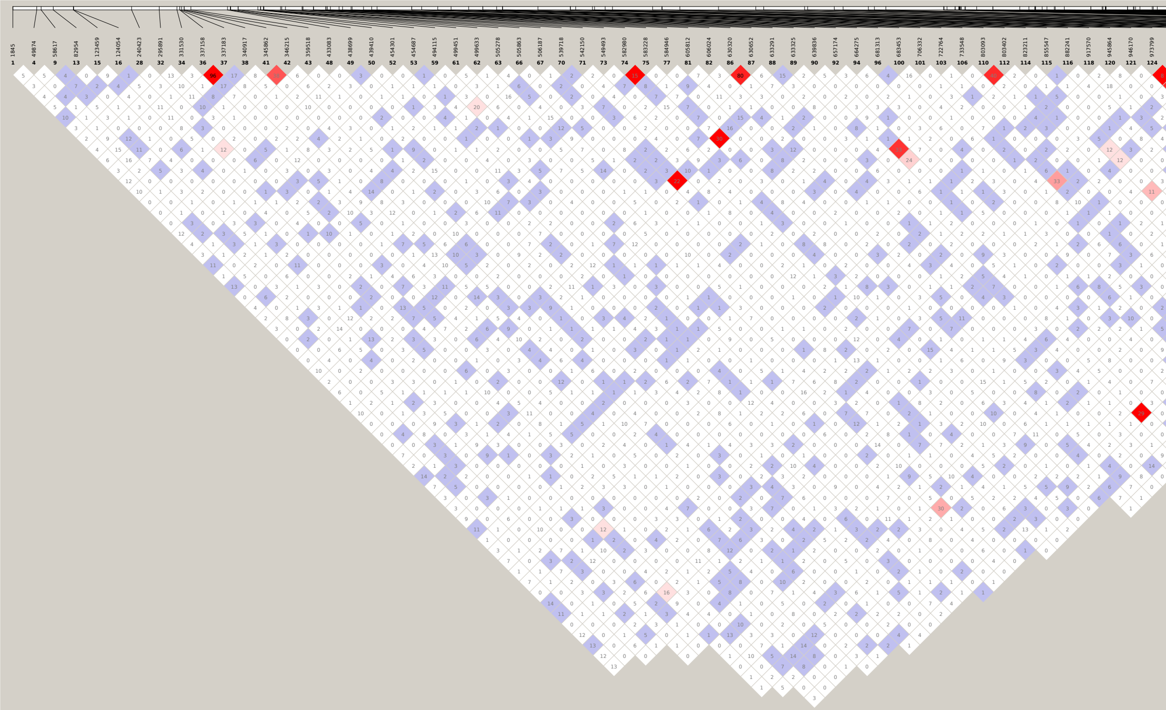

1845 1
49874 4
58617 9
82954 13
123459 15
124054 16
240423 28
295891 32
331530 34
337158 36
337183 37
340917 38
345862 41
346215 42
359518 43
433083 48
438699 49
439410 50
454301 52
454687 53
494115 59
499451 61
499633 62
505278 63
505863 66
506187 67
539718 70
542150 71
549493 73
582980 74
583228 75
584946 77
605812 81
606024 82
630320 86
630652 87
633291 88
633325 89
639836 90
657174 92
664275 94
681313 96
683453 100
706332 101
722764 103
733548 106
803093 110
803402 112
823211 114
855547 115
882241 116
917570 118
945864 120
946170 121
973799 124

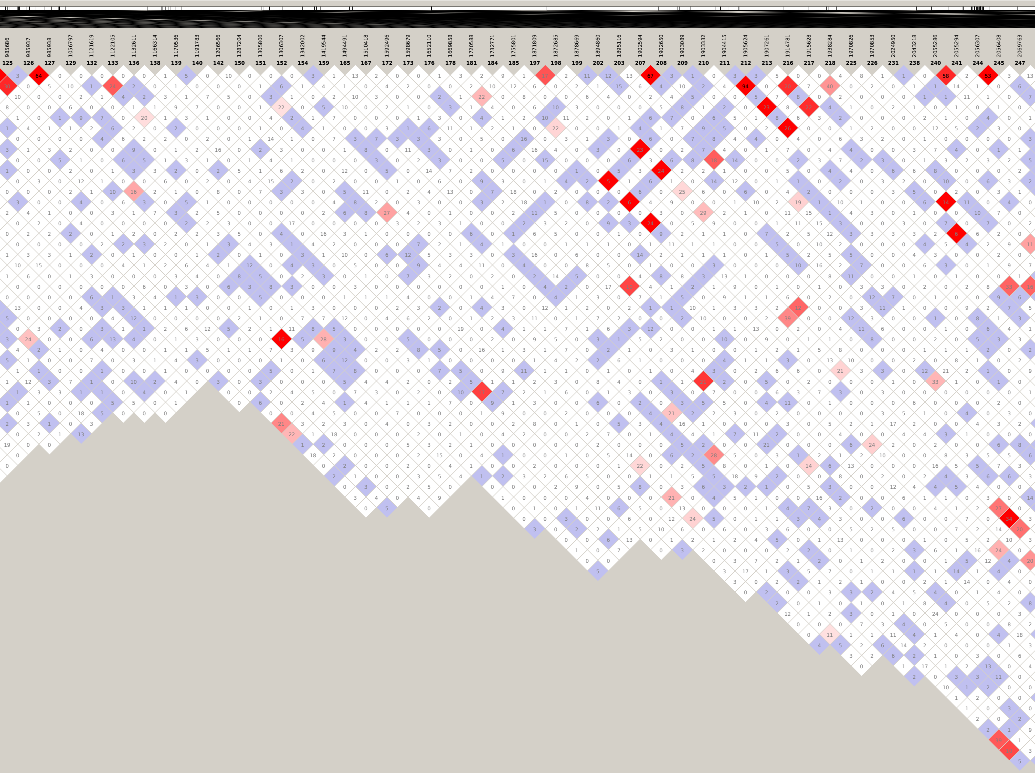

985686
985937
985938
1056797
1121619
1122105
1132611
1166314
1170536
1191783
1206566
1287204
1305806
1306307
1342002
1419544
1494491
1510418
1592496
1598679
1652110
1669858
1720588
1732771
1755801
1871809
1872685
1878669
1894860
1895116
1902594
1902650
1903089
1903332
1904415
1905624
1907261
1914781
1915628
1938284
1970826
1970853
2024950
2043218
2055286
2055294
2056307
2056408
2069763
125
126
127
129
132
133
136
138
139
140
142
150
151
152
154
159
165
167
172
173
176
178
181
184
185
197
198
199
202
203
207
208
209
210
211
212
213
216
217
218
225
226
231
238
240
241
244
245
247

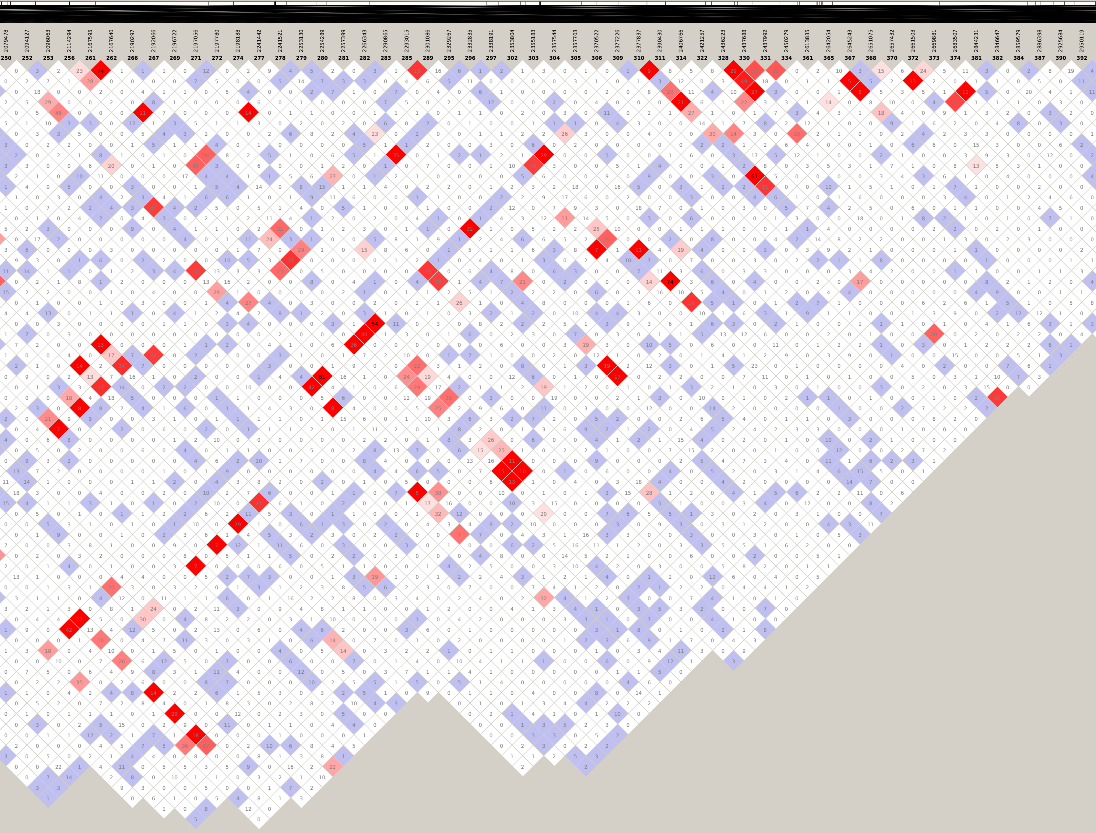

2079478 2094127 2096063 2114294 2167595 2167640 2190297 2192066 2196722 2197056 2197780 2198188 2241442 2241521 2253130 2254289 2257399 2266343 2290865 2293015 2301086 2329267 2332835 2338191 2353804 2355183 2357544 2357703 2370522 2377226 2377837 2390430 2406766 2421257 2436223 2437688 2437992 2450279 2613835 2642054 2645243 2651075 2657432 2661503 2669881 2683507 2844231 2846647 2859579 2886398 2925684 2950119
250 252 253 256 261 262 266 267 269 271 272 274 277 278 279 280 281 282 283 285 289 295 296 297 302 303 304 305 306 309 310 311 314 322 328 330 331 334 361 365 367 368 370 372 373 374 381 382 384 387 390 392

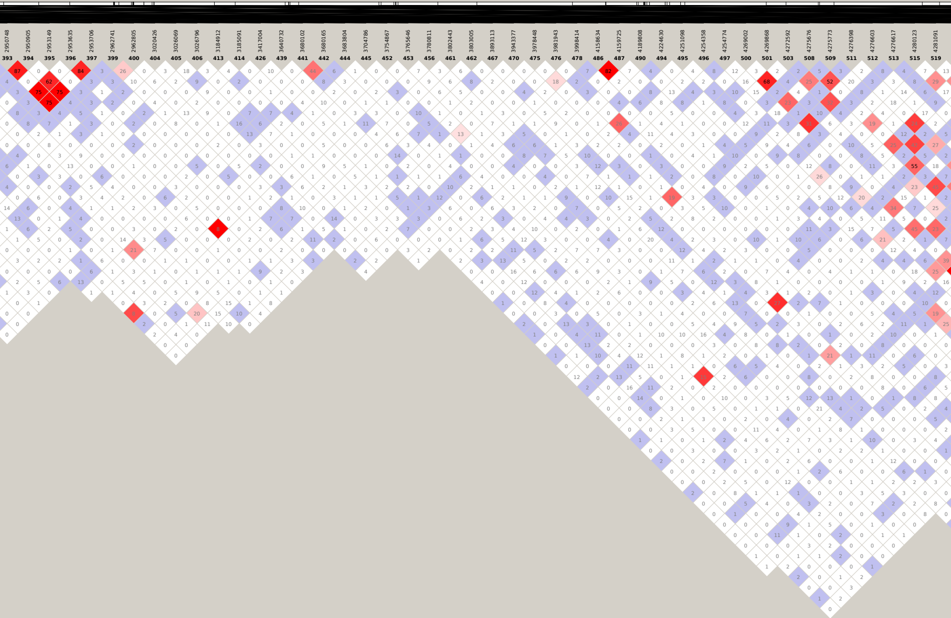

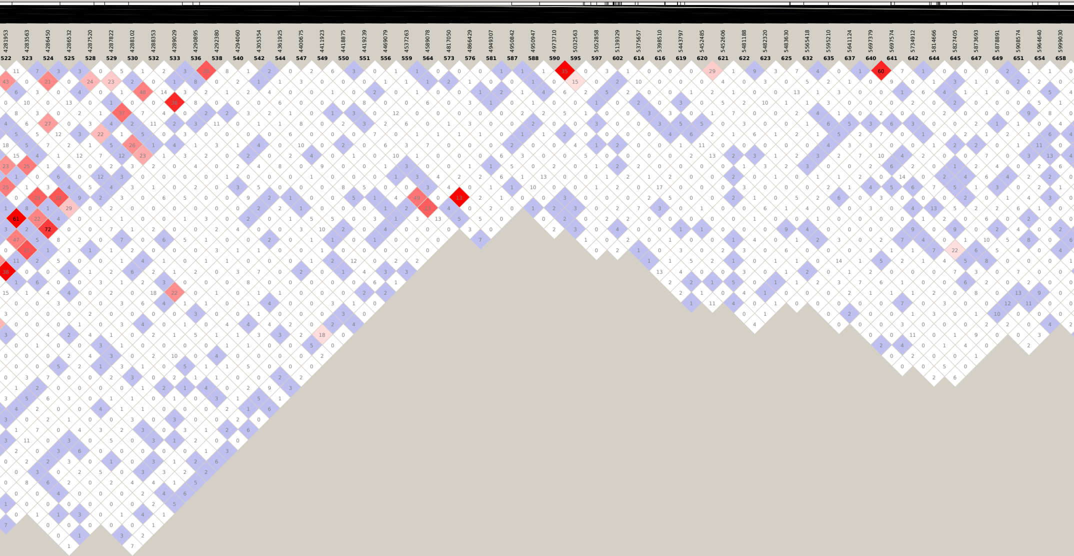

4281953 4283563 4286450 4286632 4287520 4287822 4288102 4288353 4289029 4290895 4292380 4294060 4305354 4361925 4400675 4411923 4418875 4419239 4469079 4537263 4589078 4817050 4866429 4949307 4950842 4950947 4973710 5032563 5052858 5139329 5375657 5398510 5443797 5452485 5452606 5481188 5482320 5483630 5565418 5595210 5641124 5697379 5697574 5734912 5814466 5827405 5873693 5878891 5908574 5964640 5999030
522 523 524 525 528 529 530 532 533 536 538 540 542 544 547 549 550 551 556 559 564 573 576 581 587 588 590 595 597 602 614 616 619 620 621 622 623 625 632 635 637 640 641 642 644 645 647 649 651 654 658

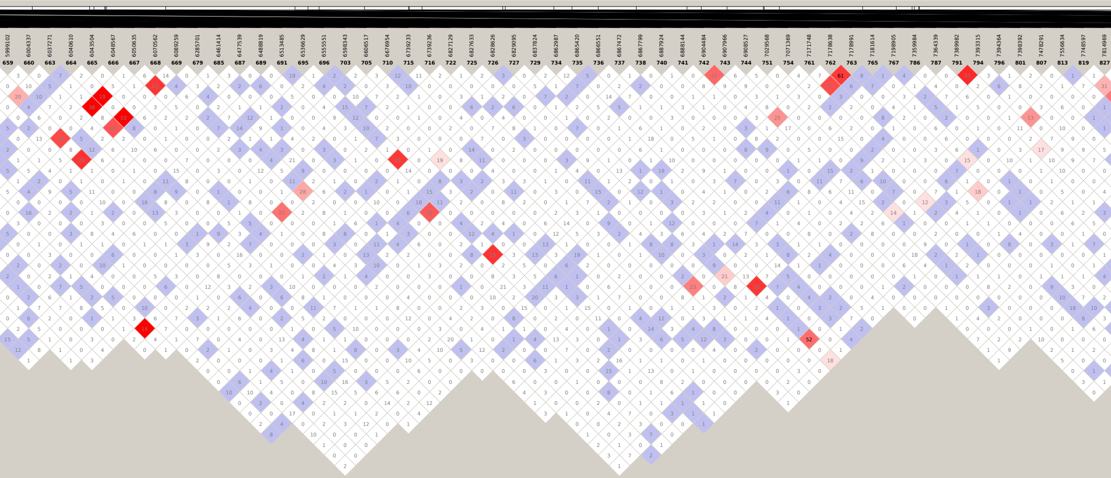
5999102 6004337 6037271 6040610 6043504 6048567 6050635 6070562 6089259 6285701 6461414 6477539 6488819 6513485 6536629 6555551 6598343 6606517 6676954 6739233 6739236 6827129 6827633 6828626 6829095 6837824 6862987 6865420 6866551 6867472 6867799 6887924 6888144 6904484 6907966 6908527 7029568 7071369 7171748 7178638 7178891 7181614 7198805 7359984 7364339 7389982 7393315 7394364 7398392 7478291 7556634 7748597 7814969
659 660 663 664 665 666 667 668 669 679 685 687 689 691 695 696 703 705 710 715 716 722 725 726 727 729 734 735 736 737 738 740 741 742 743 744 751 754 761 762 763 765 767 786 787 791 794 796 801 807 813 819 827

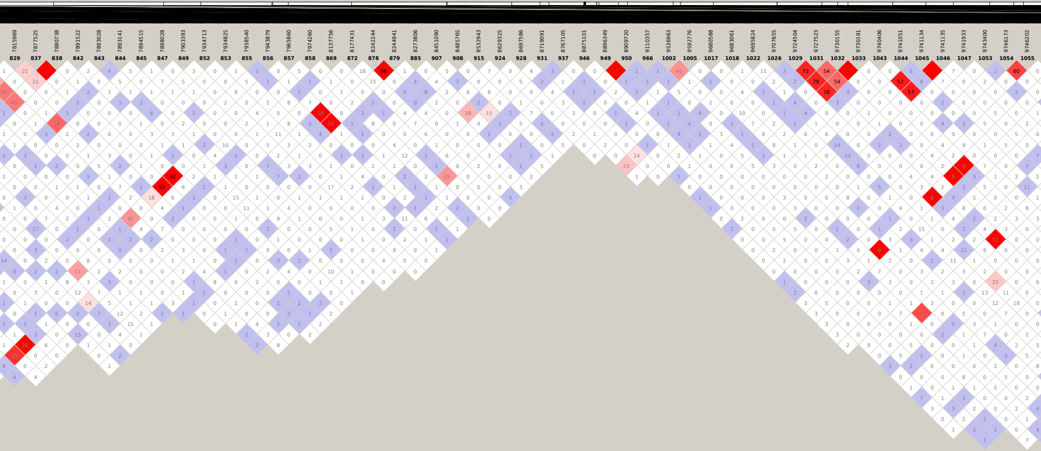
7815969 829
7877525 837
7880738 838
7891522 842
7893028 843
7893141 844
7894515 845
7898028 847
7903393 849
7934713 852
7934825 853
7938540 855
7943879 856
7965860 857
7974260 858
8137756 869
8177431 872
8242244 878
8244941 879
8273806 885
8451090 907
8485765 908
8532943 915
8629325 924
8697586 928
8719091 931
8767105 937
8875101 946
8896349 949
8909720 950
9110357 966
9536963 1002
9592776 1005
9680588 1017
9683061 1018
9695824 1022
9707655 1026
9724504 1029
9727523 1031
9730155 1032
9730191 1033
9740406 1043
9741051 1044
9741134 1045
9741135 1046
9741933 1047
9743400 1053
9746173 1054
9746202 1055

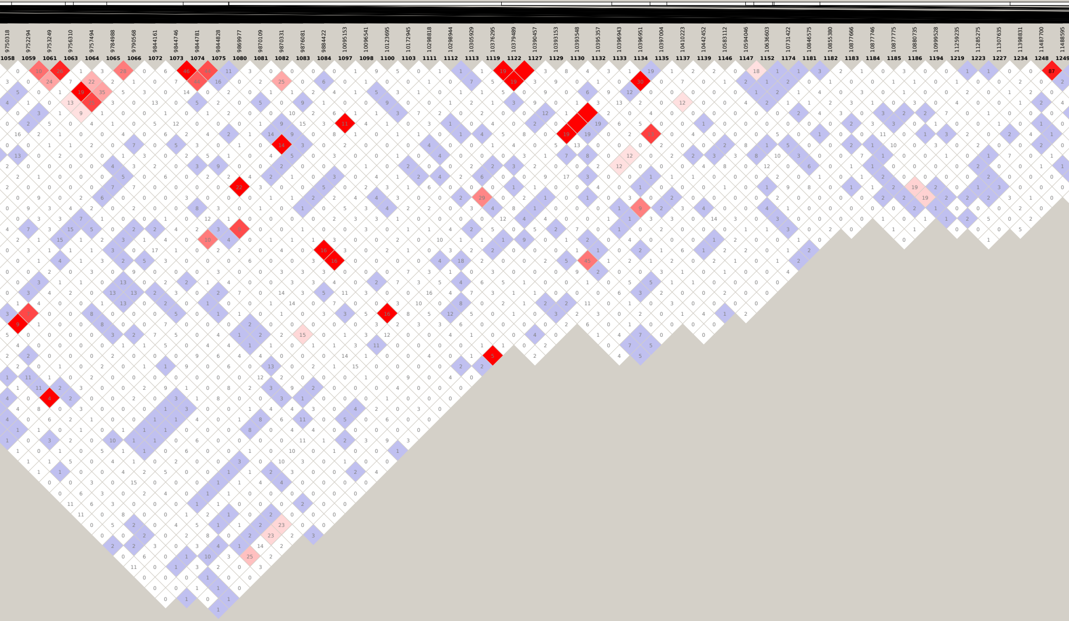
9750318 9752294 9753249 9756310 9757494 9784988 9790568 9844161 9844746 9844781 9844828 9869977 9870109 9870331 9876081 9884422 10095153 10096541 10123695 10172945 10298818 10298944 10305929 10376295 10379489 10390457 10393153 10393548 10395357 10396943 10396951 10397004 10410223 10442452 10583112 10594046 10636603 10731422 10846575 10855380 10877666 10877746 10877775 10880735 10999528 11259235 11285275 11307635 11398831 11487700 11488595
1058 1059 1061 1063 1064 1065 1066 1072 1073 1074 1075 1080 1081 1082 1083 1084 1097 1098 1100 1103 1111 1112 1113 1119 1122 1127 1129 1130 1132 1133 1134 1135 1137 1139 1146 1147 1152 1174 1181 1182 1183 1184 1185 1186 1194 1219 1223 1227 1234 1248 1249

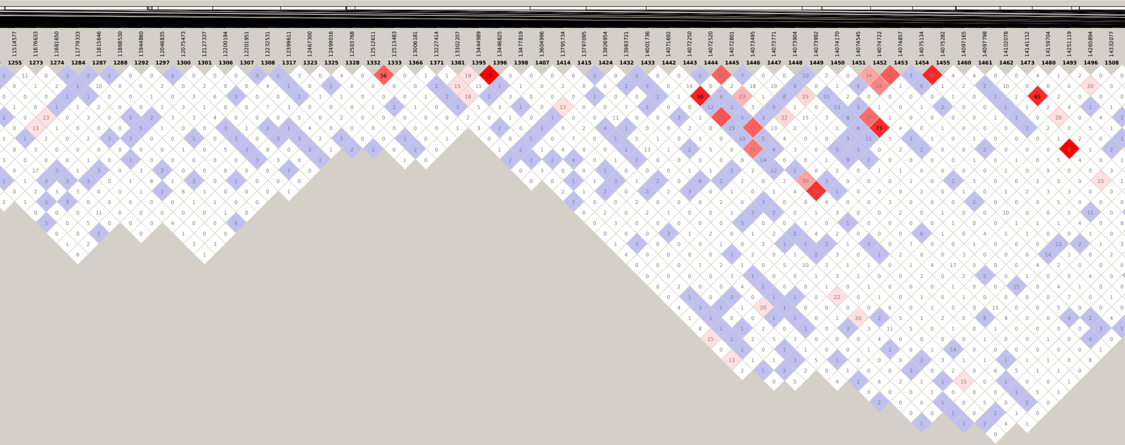
11514577 11676633 11681650 11779333 11815646 11868530 11944860 12046835 12075473 12127337 12200194 12201951 12232531 12399611 12467300 12499016 12503768 12512611 12513483 13006181 13227414 13302207 13344989 13446825 13477819 13604996 13795734 13797095 13926954 13983721 14001736 14071692 14072250 14072520 14072801 14073495 14073771 14073904 14073992 14074170 14074545 14074722 14074857 14075134 14075282 14097165 14097798 14102078 14141152 14159704 14251119 14265894 14332077
1255 1273 1274 1284 1287 1288 1292 1297 1300 1301 1306 1307 1308 1317 1323 1325 1328 1332 1333 1366 1371 1381 1395 1396 1398 1407 1414 1415 1424 1432 1433 1442 1443 1444 1445 1446 1447 1448 1449 1450 1451 1452 1453 1454 1455 1460 1461 1462 1473 1480 1493 1496 1508

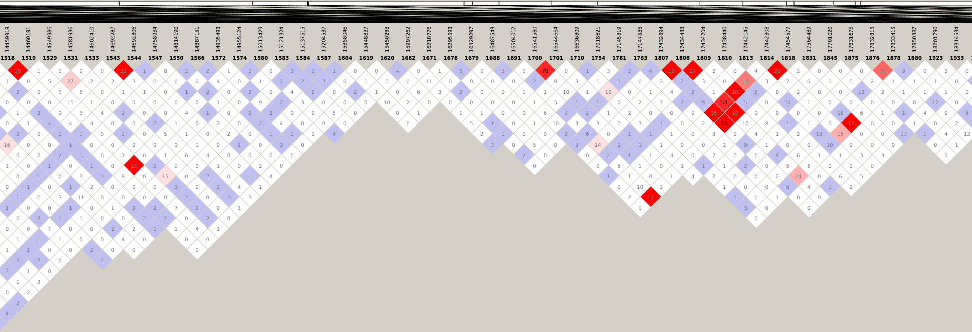

14459919 14460191 14549986 14581936 14602410 14692287 14692306 14758934 14814190 14887151 14935498 14955124 15013429 15121324 15137515 15204537 15358046 15448837 15450288 15997262 16218776 16295598 16329297 16487543 16504012 16541580 16544664 16636809 17018621 17145819 17147585 17432894 17434433 17434704 17438440 17442145 17442308 17454577 17564489 17701020 17831675 17832815 17833415 17850387 18201796 18314534
1518 1519 1529 1531 1533 1543 1544 1547 1550 1566 1572 1574 1580 1583 1584 1587 1604 1619 1620 1662 1671 1676 1679 1688 1691 1700 1701 1710 1754 1781 1783 1807 1808 1809 1810 1813 1814 1818 1831 1845 1875 1876 1878 1880 1923 1933

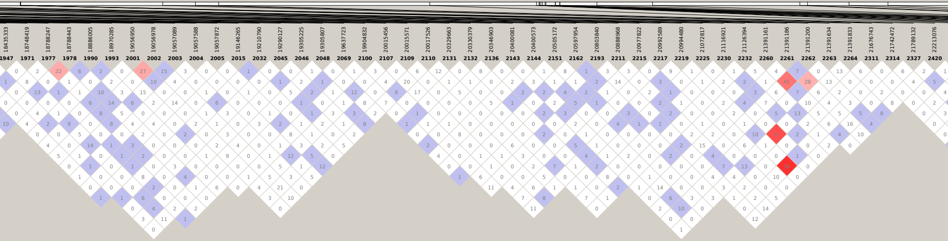
18435333 18748419 18788247 18788443 18886005 18970285 19056950 19056978 19057089 19057588 19057872 19146265 19210790 19290127 19305225 19305807 19637723 19904832 20015456 20015571 20017526 20329663 20330379 20346903 20400081 20400573 20505172 20597954 20803840 20888968 20977822 20992589 20994480 21072817 21116921 21126394 21391161 21391186 21391200 21391634 21391833 21676743 21742472 21789132 22213076
1947 1971 1977 1978 1990 1993 2001 2002 2003 2004 2005 2015 2032 2045 2046 2048 2069 2100 2107 2109 2110 2131 2132 2136 2143 2144 2151 2162 2193 2211 2215 2217 2219 2225 2230 2232 2260 2261 2262 2263 2264 2311 2314 2327 2420

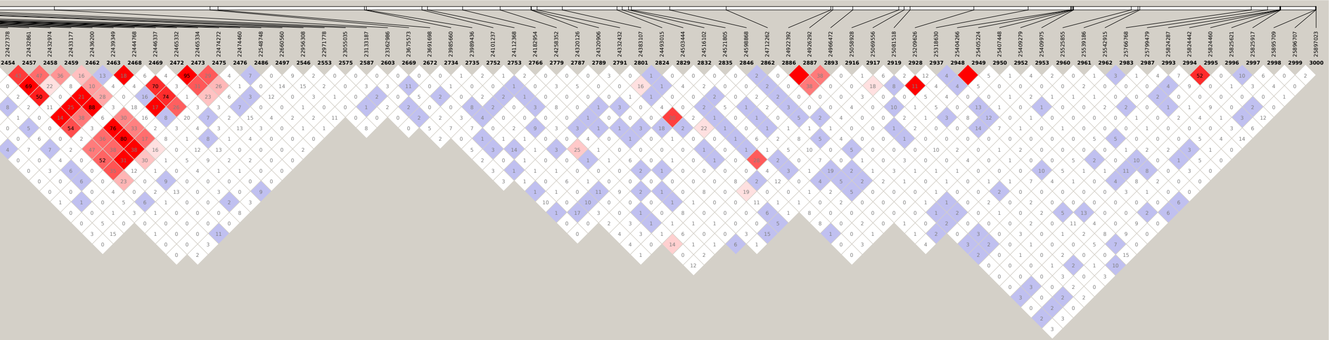
22427378 22432861 22432974 22433177 22436200 22439349 22444768 22446337 22465332 22465334 22474272 22474460 22548748 22660560 22956308 22971778 23055035 23133187 23362986 23675573 23691698 23985660 23989436 24101237 24112368 24182954 24258352 24320126 24320906 24332432 24383107 24493015 24503444 24516102 24521805 24598868 24712262 24922392 24926292 24966472 25058928 25069556 25081518 25209626 25318630 25404266 25405224 25407448 25409279 25409975 25525855 25539186 25542915 25766768 25799479 25824287 25824442 25824460 25825621 25825917 25895709 25896707 25897023
2454 2457 2458 2459 2462 2463 2468 2469 2472 2473 2475 2476 2486 2497 2546 2553 2575 2587 2603 2669 2672 2734 2735 2752 2753 2766 2779 2787 2789 2791 2801 2824 2829 2832 2835 2846 2862 2886 2887 2893 2916 2917 2919 2928 2937 2948 2949 2950 2952 2953 2960 2961 2962 2983 2987 2993 2994 2995 2996 2997 2998 2999 3000

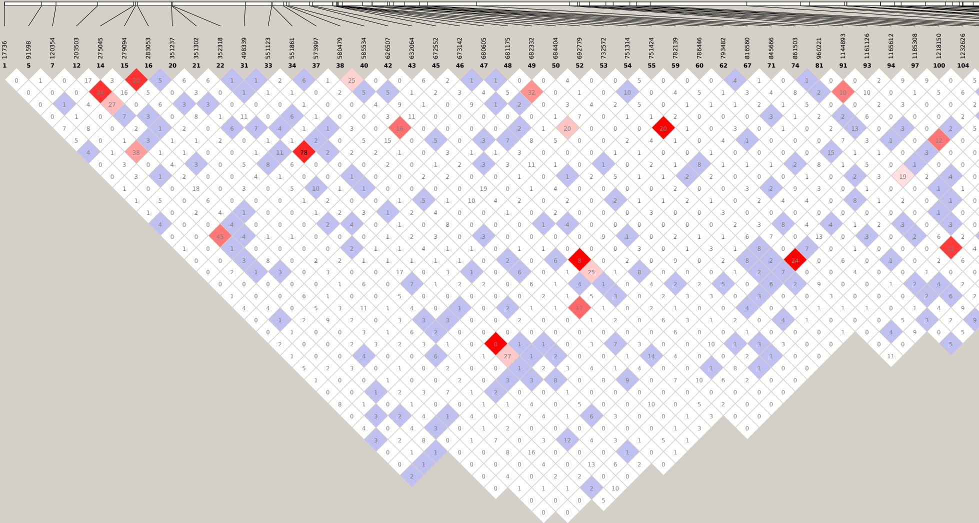

17736 1
91598 5
120354 7
203503 12
275045 14
279094 15
283053 16
351237 20
351302 21
352318 22
498339 31
551123 33
551861 34
573997 37
580479 38
585534 40
626507 42
632064 43
672552 45
673142 46
680605 47
681175 48
682332 49
684404 50
692779 52
732572 53
751314 54
751424 55
782139 59
786446 60
793482 62
816560 67
845666 71
861503 74
960221 81
1144893 91
1161126 93
1165612 94
1185308 97
1218150 100
1232626 104

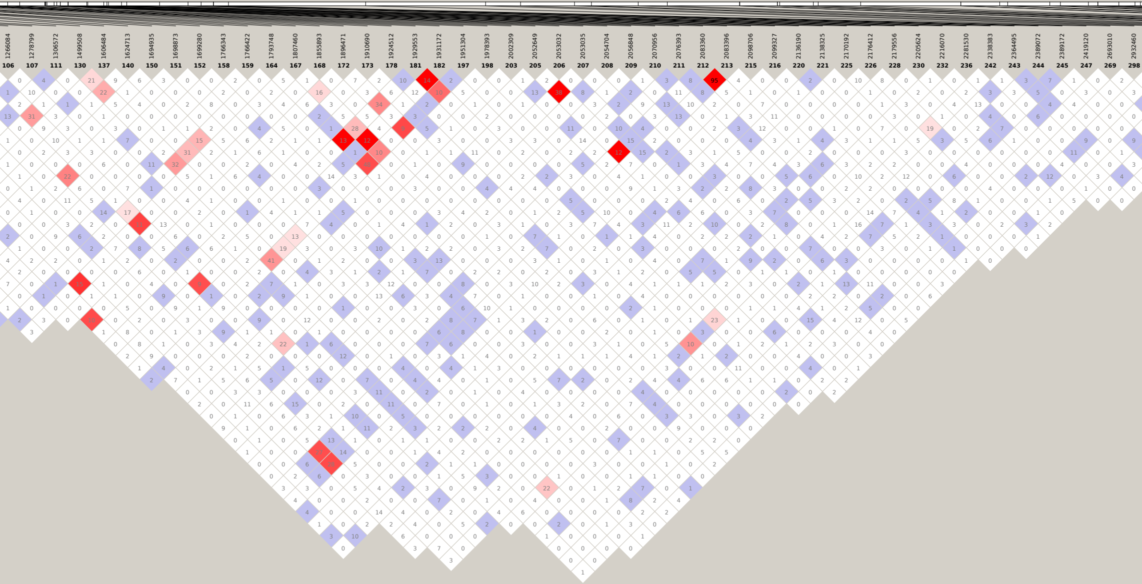

1266084 106
1278799 107
1306572 111
1499508 130
1606484 137
1624713 140
1694935 150
1698873 151
1699280 152
1766343 158
1766422 159
1793748 164
1807460 167
1855893 168
1896471 172
1910690 173
1924512 178
1929553 181
1931172 182
1951304 197
1978393 198
2002309 203
2052649 205
2053032 206
2053035 207
2054704 208
2056848 209
2070956 210
2076393 211
2083360 212
2083396 213
2098706 215
2099327 216
2136190 220
2138325 221
2170192 225
2176412 226
2179556 228
2205624 230
2216070 232
2281530 236
2338383 242
2364495 243
2389072 244
2389172 245
2419120 247
2693010 269
2932460 298

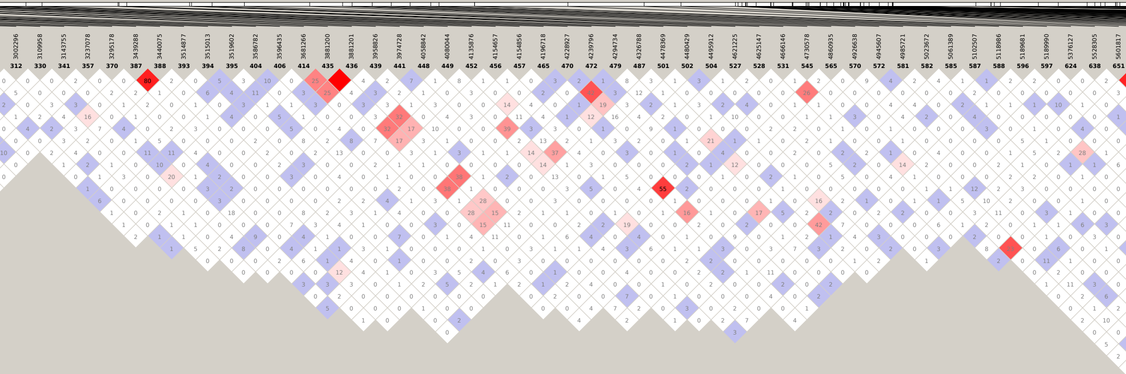
3002296
3109958
3143755
3237078
3295178
3439288
3440075
3514877
3515013
3519602
3586782
3596435
3681266
3881200
3881201
3958826
3974728
4058842
4080044
4135876
4154657
4154856
4196718
4228927
4239796
4294734
4326788
4478369
4480429
4495912
4621225
4625147
4666146
4730578
4860935
4926638
4945607
4985721
5023672
5061389
5102507
5118986
5189681
5189990
5376127
5528305
5601817
312
330
341
357
370
387
388
393
394
395
404
406
414
435
436
439
441
448
449
452
456
457
465
470
472
479
487
501
502
504
527
528
531
545
565
570
572
581
582
585
587
588
596
597
624
638
651

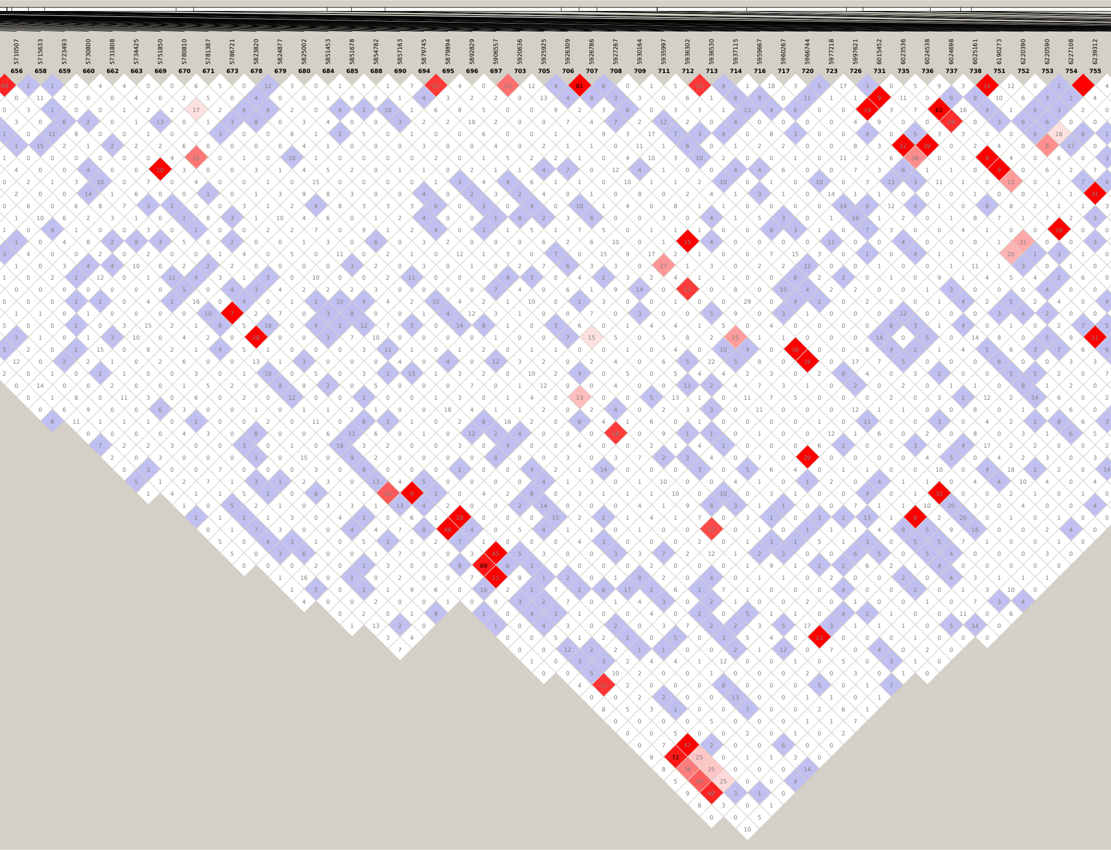

5710507
656
5715633
658
5723493
659
5730800
660
5731808
662
5734425
663
5751850
669
5780810
670
5781387
671
5786721
673
5823820
678
5824877
679
5825002
680
5851453
684
5851678
685
5854782
688
5857163
690
5879745
694
5879894
695
5892829
696
5906557
697
5920636
703
5925925
705
5926309
706
5926786
707
5927267
708
5930164
709
5935997
711
5936302
712
5936530
713
5937115
714
5959667
716
5960267
717
5966744
720
5977218
723
5997621
726
6015452
731
6023536
735
6024538
736
6024698
737
6025161
738
6190273
751
6220390
752
6220590
753
6227108
754
6239312
755

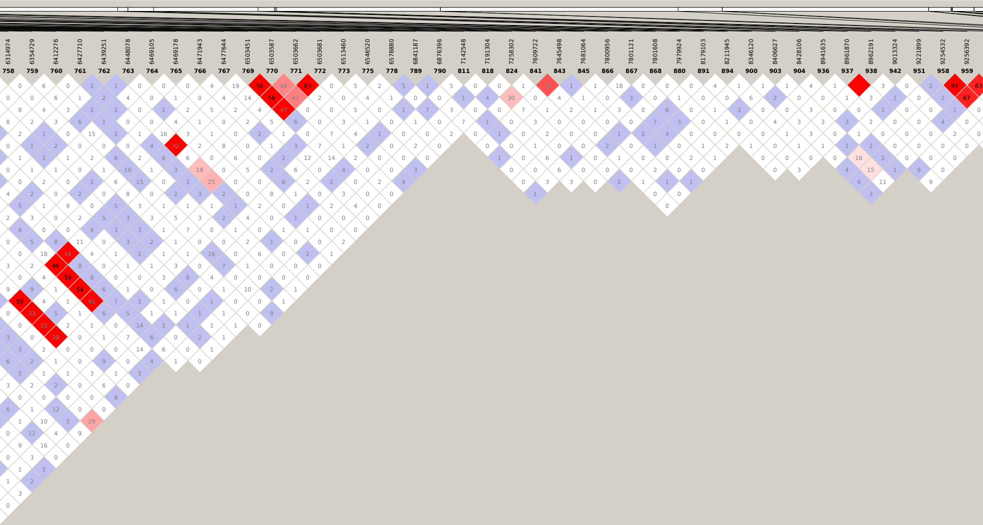
6314974 6354729 6412276 6427710 6439251 6448078 6469105 6469178 6471943 6477644 6503451 6503587 6503662 6503681 6513460 6546520 6578880 6841187 6876396 7142548 7191304 7258302 7609722 7645498 7681064 7800956 7801121 7801608 7979924 8179103 8211945 8346120 8406627 8428106 8941635 8961870 8962191 9013324 9221899 9254532 9256392
758 759 760 761 762 763 764 765 766 767 769 770 771 772 773 775 778 789 790 811 818 824 841 843 845 866 867 868 880 891 894 900 903 904 936 937 938 942 951 958 959

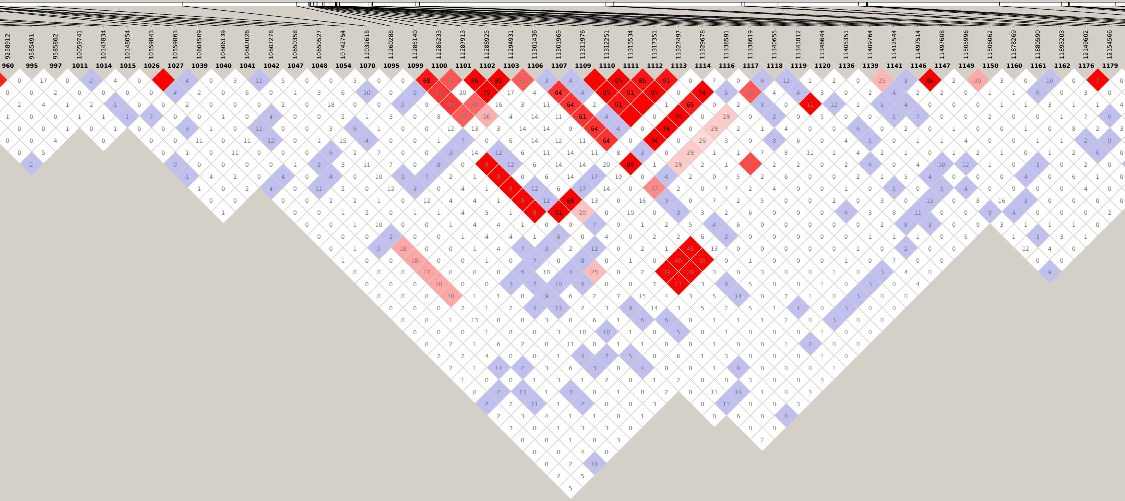
9258912 9585491 9585862 10059741 10147834 10148054 10559843 10559863 10604509 10606139 10607026 10607278 10650358 10650527 10742754 11032618 11260288 11285140 11286233 11287913 11288925 11294931 11301436 11301969 11311976 11312251 11315534 11317351 11327497 11329678 11338591 11338619 11340655 11341812 11346644 11405351 11409764 11412544 11497514 11497608 11505996 11506062 11878269 11880590 11893203 12149602 12154566
960 995 997 1011 1014 1015 1026 1027 1039 1040 1041 1042 1047 1048 1054 1070 1095 1099 1100 1101 1102 1103 1106 1107 1109 1110 1111 1112 1113 1114 1116 1117 1118 1119 1120 1136 1139 1141 1146 1147 1149 1150 1160 1161 1162 1176 1179

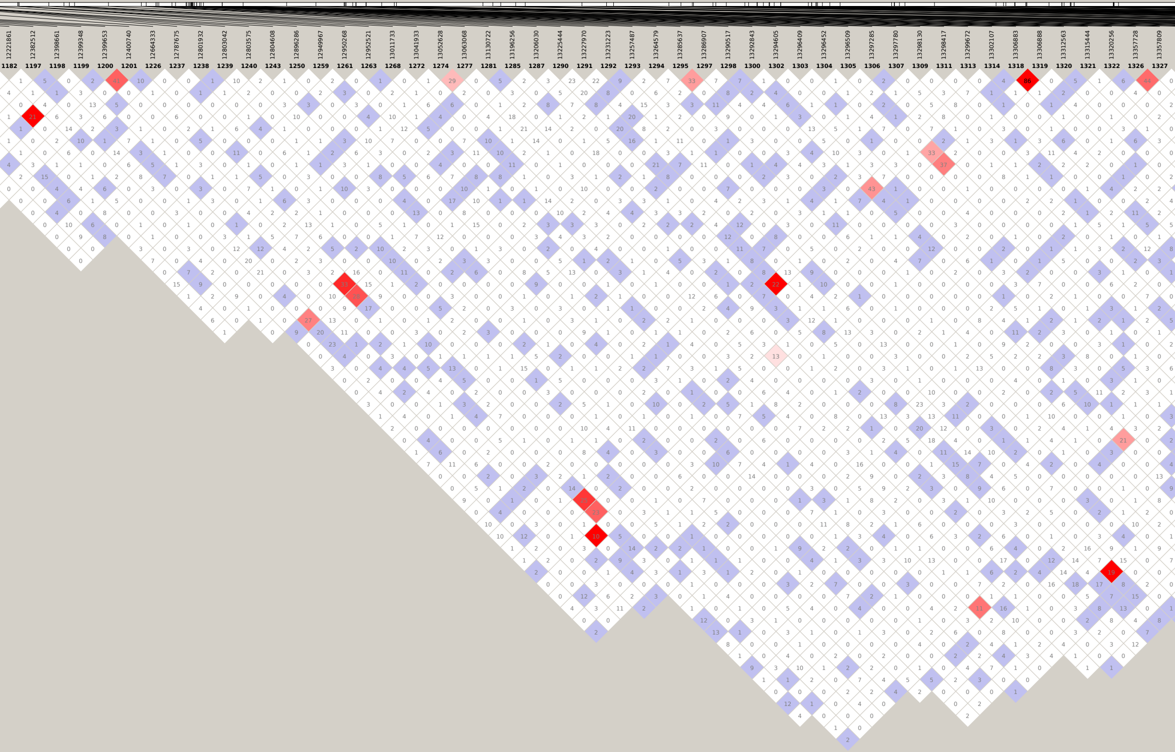
12221861 12382512 12398661 12399348 12399653 12400740 12664333 12787675 12801932 12803042 12803575 12804608 12896286 12949967 12950268 12952521 13011733 13041933 13052628 13063068 13130722 13196256 13206030 13225444 13227970 13231223 13257487 13264579 13285637 13286907 13290517 13292843 13294605 13296409 13296452 13296509 13297285 13297780 13298130 13298417 13299672 13302107 13306883 13306888 13312563 13315444 13320256 13357728 13357809
1182 1197 1198 1199 1200 1201 1226 1237 1238 1239 1240 1243 1250 1259 1261 1263 1268 1272 1274 1277 1281 1285 1287 1290 1291 1292 1293 1294 1295 1297 1298 1300 1302 1303 1304 1305 1306 1307 1309 1311 1313 1314 1318 1319 1320 1321 1322 1326 1327

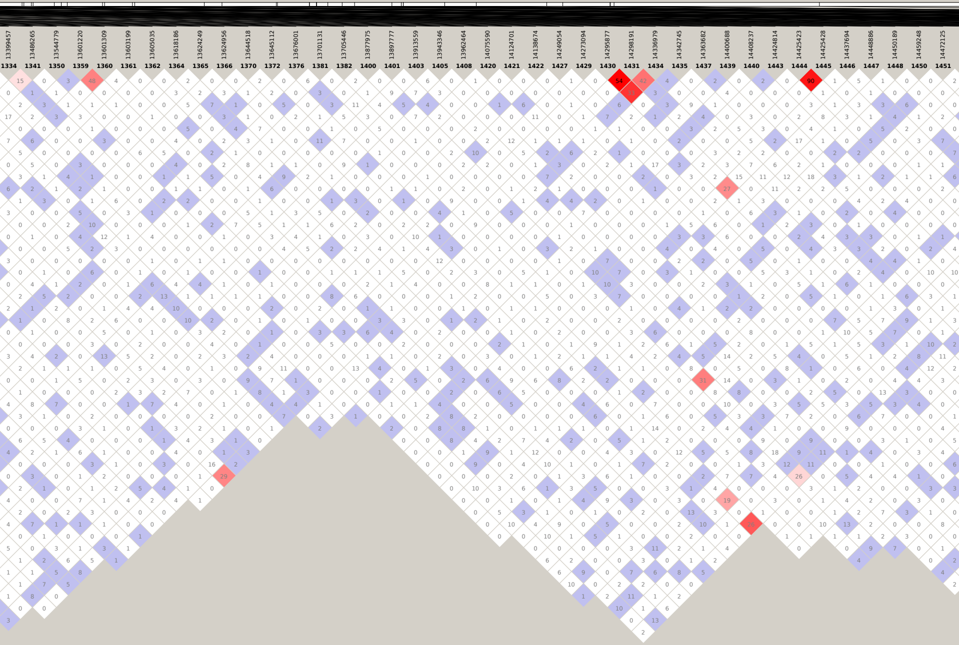
13399457
13486265
13544779
13601220
13601309
13603199
13605035
13618186
13624249
13624956
13644518
13645112
13676001
13701131
13705446
13877975
13897777
13913559
13943346
13962464
14075590
14124701
14138674
14249054
14273094
14295877
14298191
14336979
14342745
14363682
14400688
14408237
14424814
14425423
14425428
14437694
14448886
14450189
14459248
14472125
1334 1341 1350 1359 1360 1361 1362 1364 1365 1366 1370 1372 1376 1381 1382 1400 1401 1403 1405 1408 1420 1421 1422 1427 1429 1430 1431 1434 1435 1437 1439 1440 1443 1444 1445 1446 1447 1448 1450 1451

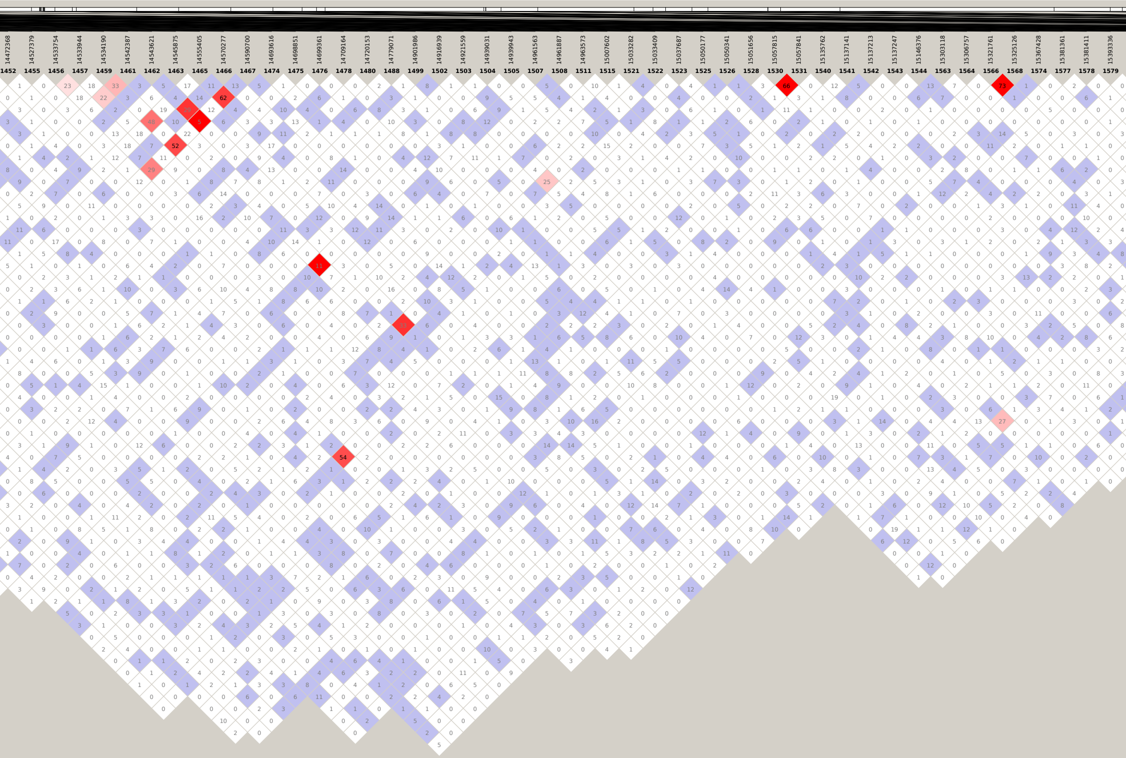
14472368
14527379
14533754
14533944
14534190
14542387
14543621
14545875
14555405
14570277
14590700
14693616
14698851
14699361
14709164
14720153
14779071
14901986
14916939
14921559
14939031
14939943
14961563
14961887
14963573
15007602
15033282
15033409
15037687
15050177
15050341
15051656
15057815
15057841
15135762
15137141
15137213
15137247
15146376
15303118
15306757
15321761
15325126
15367428
15381361
15381411
15393336
1452 1455 1456 1457 1459 1461 1462 1463 1465 1466 1467 1474 1475 1476 1478 1480 1488 1499 1502 1503 1504 1505 1507 1508 1511 1515 1521 1522 1523 1525 1526 1528 1530 1531 1540 1541 1542 1543 1544 1563 1564 1566 1568 1574 1577 1578 1579

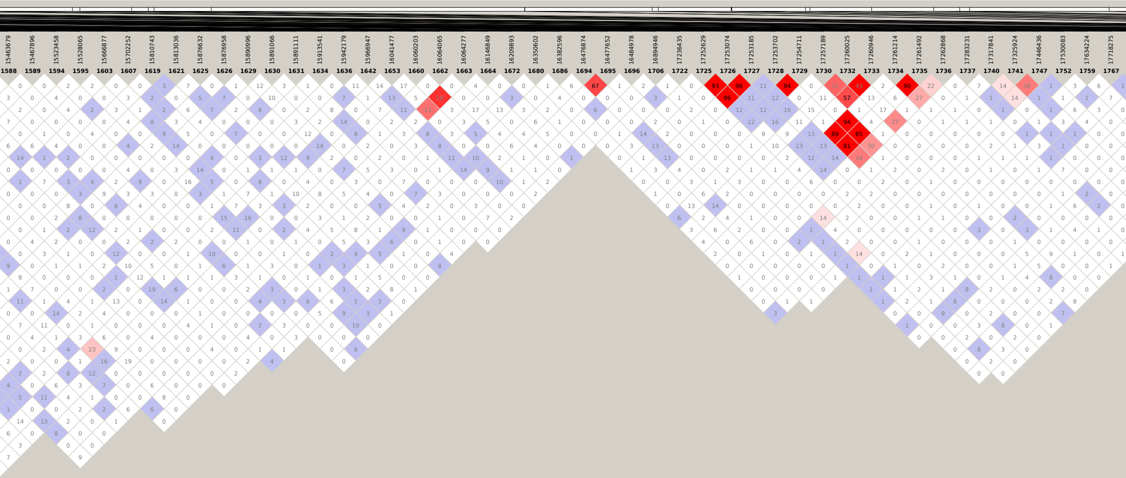

15463679 1588
15467896 1589
15523458 1594
15528065 1595
15666877 1603
15702252 1607
15810743 1619
15813036 1621
15876632 1625
15876958 1626
15890996 1629
15891066 1630
15891111 1631
15913541 1634
15942179 1636
15966947 1642
16041477 1653
16060203 1660
16064065 1662
16064277 1663
16146849 1664
16209893 1672
16350602 1680
16382596 1686
16476874 1694
16477652 1695
16484978 1696
16894946 1706
17236435 1722
17252629 1725
17253074 1726
17253185 1727
17253702 1728
17254711 1729
17257189 1730
17260025 1732
17260946 1733
17261214 1734
17261492 1735
17262868 1736
17283231 1737
17317841 1740
17325924 1741
17446436 1747
17530083 1752
17634224 1759
17718275 1767

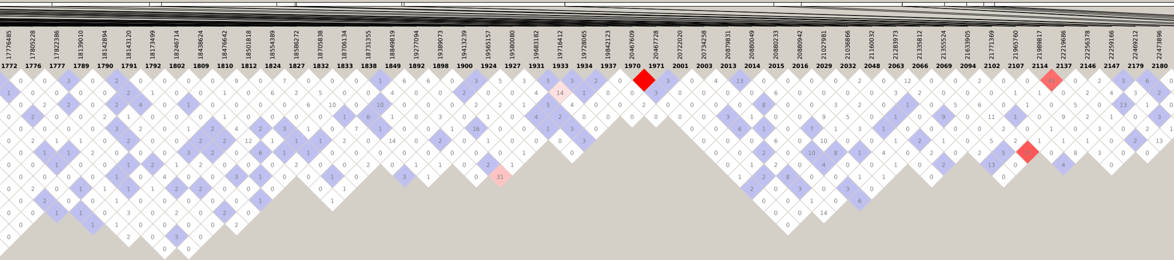
17776485 17805228 17822386 18139010 18142894 18143120 18173499 18246714 18438624 18476642 18501818 18554389 18586272 18705838 18706134 18731355 18849819 19277094 19389073 19413239 19565157 19580080 19683182 19716412 19728065 19842123 20467609 20467728 20722020 20734258 20879831 20880049 20880233 20880942 21027981 21036866 21160032 21283973 21335812 21355524 21633905 21771369 21965760 21989817 22219686 22256378 22259166 22469212 22473896
1772 1776 1777 1789 1790 1791 1792 1802 1809 1810 1812 1824 1827 1832 1833 1838 1849 1892 1898 1900 1924 1927 1931 1933 1934 1937 1970 1971 2001 2003 2013 2014 2015 2016 2029 2032 2048 2063 2066 2069 2094 2102 2107 2114 2137 2146 2147 2179 2180

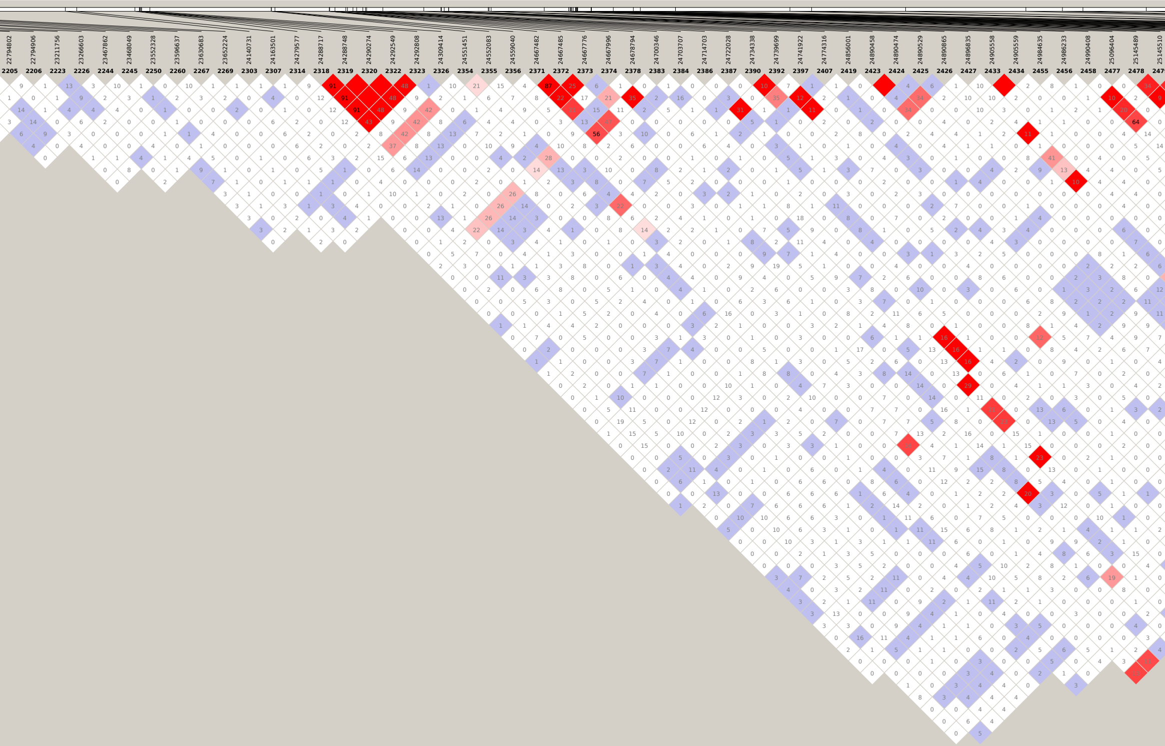
22794802 22794906 23211756 23266603 23467862 23468049 23552328 23596637 23630683 23652224 24140731 24163501 24279577 24288717 24288748 24290274 24292549 24292808 24309414 24551451 24552083 24559040 24667482 24667485 24667776 24667996 24678794 24700346 24703707 24714703 24722028 24734338 24739699 24741922 24774316 24856001 24890458 24890474 24890529 24890865 24896835 24905558 24905559 24984635 24986233 24990408 25096404 25145489 25145510
2205 2206 2223 2226 2244 2245 2250 2260 2267 2269 2303 2307 2314 2318 2319 2320 2322 2323 2326 2354 2355 2356 2371 2372 2373 2374 2378 2383 2384 2386 2387 2390 2392 2397 2407 2419 2423 2424 2425 2426 2427 2433 2434 2455 2456 2458 2477 2478

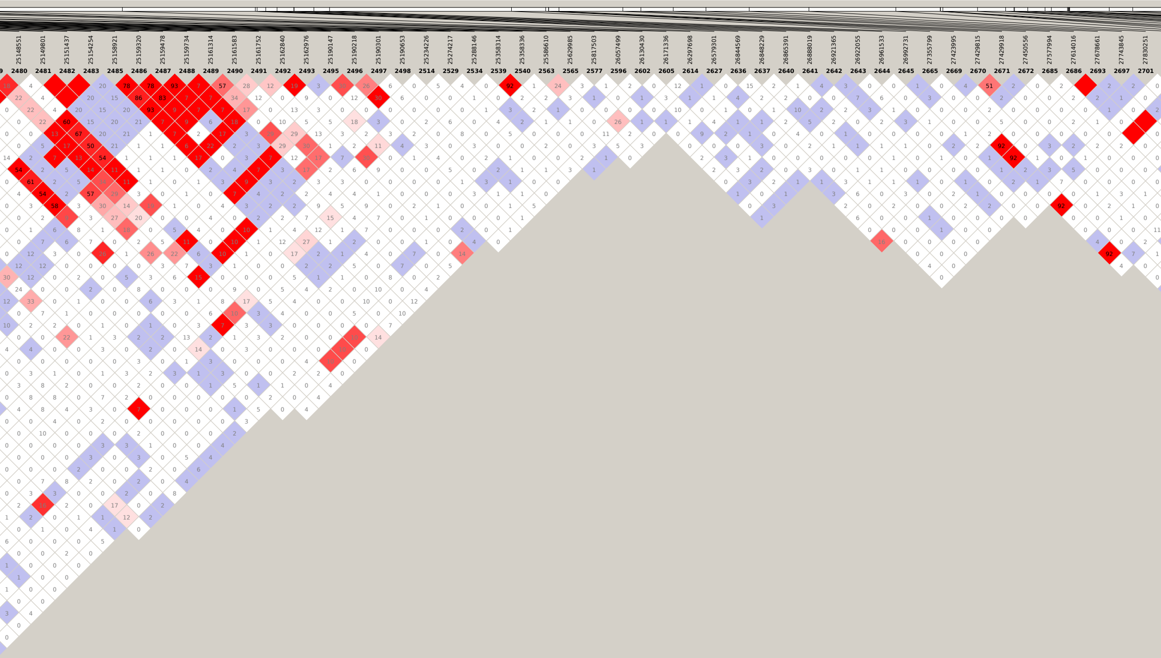
25148551
25149801
25151437
25154254
25158921
25159320
25159478
25159734
25161314
25161583
25161752
25162840
25162976
25190147
25190218
25190301
25190653
25234226
25274217
25288146
25358314
25358336
25586610
25629985
25817503
26057499
26130430
26171336
26297698
26579301
26844569
26848229
26865391
26888019
26921365
26922055
26961533
26992731
27355799
27423995
27429815
27429918
27450556
27577994
27614016
27678661
27743845
27830251
2480 2481 2482 2483 2485 2486 2487 2488 2489 2490 2491 2492 2493 2495 2496 2497 2498 2514 2529 2534 2539 2540 2563 2565 2577 2596 2602 2605 2614 2627 2636 2637 2640 2641 2642 2643 2644 2645 2665 2669 2670 2671 2672 2685 2686 2693 2697 2701

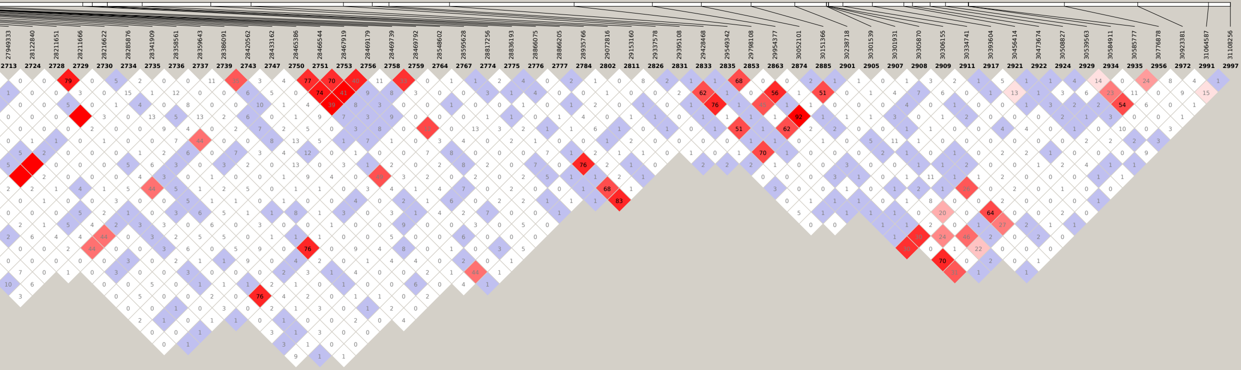
27949333 28122840 28211651 28211666 28216622 28285876 28341909 28358561 28359643 28386091 28420562 28433162 28465386 28466544 28467919 28469179 28469739 28469792 28548602 28595628 28817256 28836193 28866075 28866205 28935766 29072816 29153160 29337578 29395108 29428468 29549342 29798108 29954377 30052101 30151366 30238718 30301539 30301931 30305870 30306155 30334741 30393604 30456414 30473674 30508827 30539563 30584911 30585777 30776878 30923381 31064587 31108256
2713 2724 2728 2729 2730 2734 2735 2736 2737 2739 2743 2747 2750 2751 2753 2756 2758 2759 2764 2767 2774 2775 2776 2777 2784 2802 2811 2826 2831 2833 2835 2853 2863 2874 2885 2901 2905 2907 2908 2909 2911 2917 2921 2922 2924 2929 2934 2935 2956 2972 2991 2997

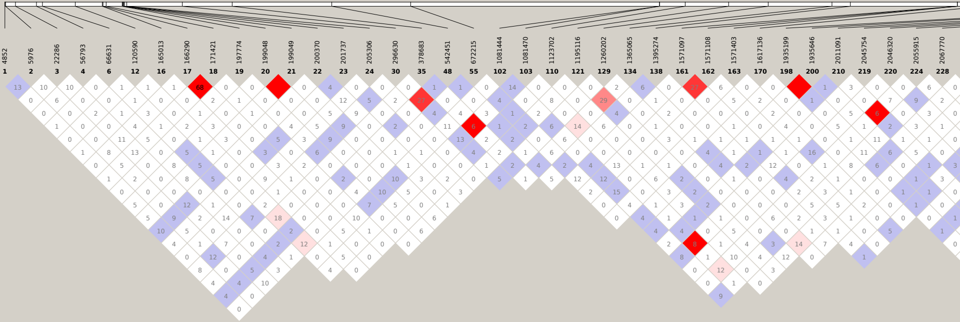

4852 5976 22286 56793 66631 120590 165013 166290 171421 197774 199048 199049 200370 201737 205306 296630 378683 542451 672215 1081444 1081470 1123702 1195116 1260202 1365065 1395274 1571097 1571108 1571403 1617136 1935199 1935646 2011091 2045754 2046320 2055915 2067770
1 2 3 4 6 12 16 17 18 19 20 21 22 23 24 30 35 48 55 102 103 110 121 129 134 138 161 162 163 170 198 200 210 219 220 224 228

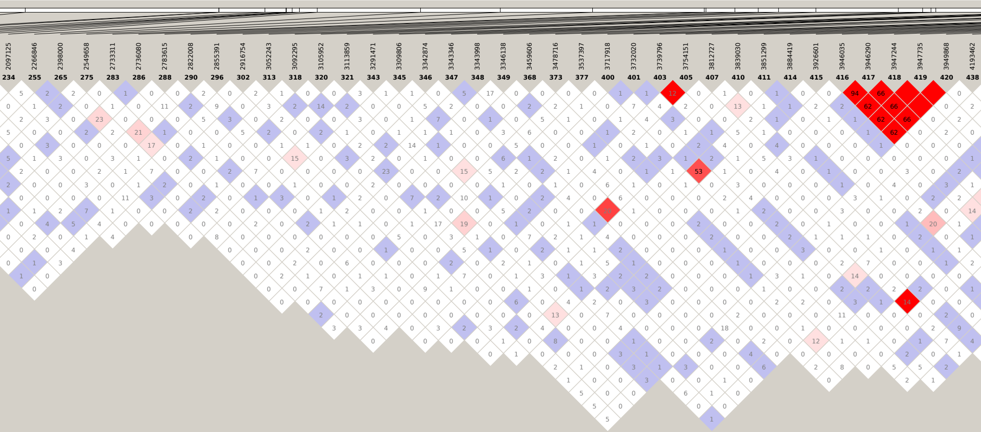
2097125 2266846 2398000 2549658 2733311 2736080 2783615 2822008 2855391 2916754 3052243 3092295 3105952 3113859 3291471 3309806 3342874 3343346 3343998 3346138 3459606 3478716 3537397 3717918 3732020 3739796 3754151 3812727 3839030 3851299 3884419 3926601 3946035 3946290 3947244 3947735 3949868 4193462
234 255 265 275 283 286 288 290 296 302 313 318 320 321 343 345 346 347 348 349 368 373 377 400 401 403 405 407 410 411 414 415 416 417 418 419 420 438

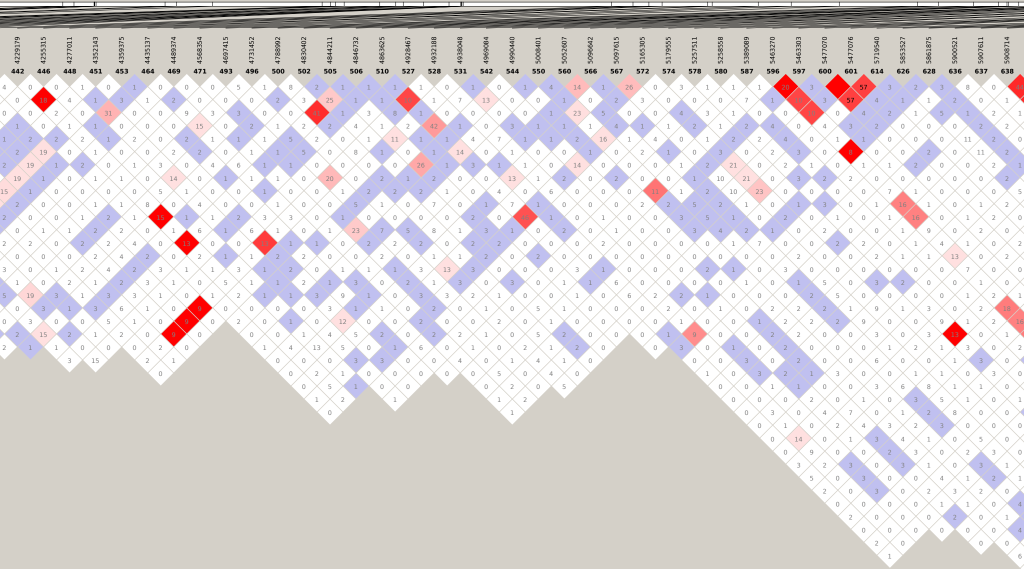
4229179
442
4255315
446
4277011
448
4352143
451
4359375
453
4435137
464
4489374
469
4568354
471
4697415
493
4731452
496
4788992
500
4830402
502
4844211
505
4846732
506
4863625
510
4928467
527
4932188
528
4938048
531
4969084
542
4990440
544
5008401
550
5052607
560
5096642
566
5097615
567
5165305
572
5179555
574
5257511
578
5258558
580
5389089
587
5463270
596
5463303
597
5477070
600
5477076
601
5719540
614
5853527
626
5861875
628
5900521
636
5907611
637
5908714
638

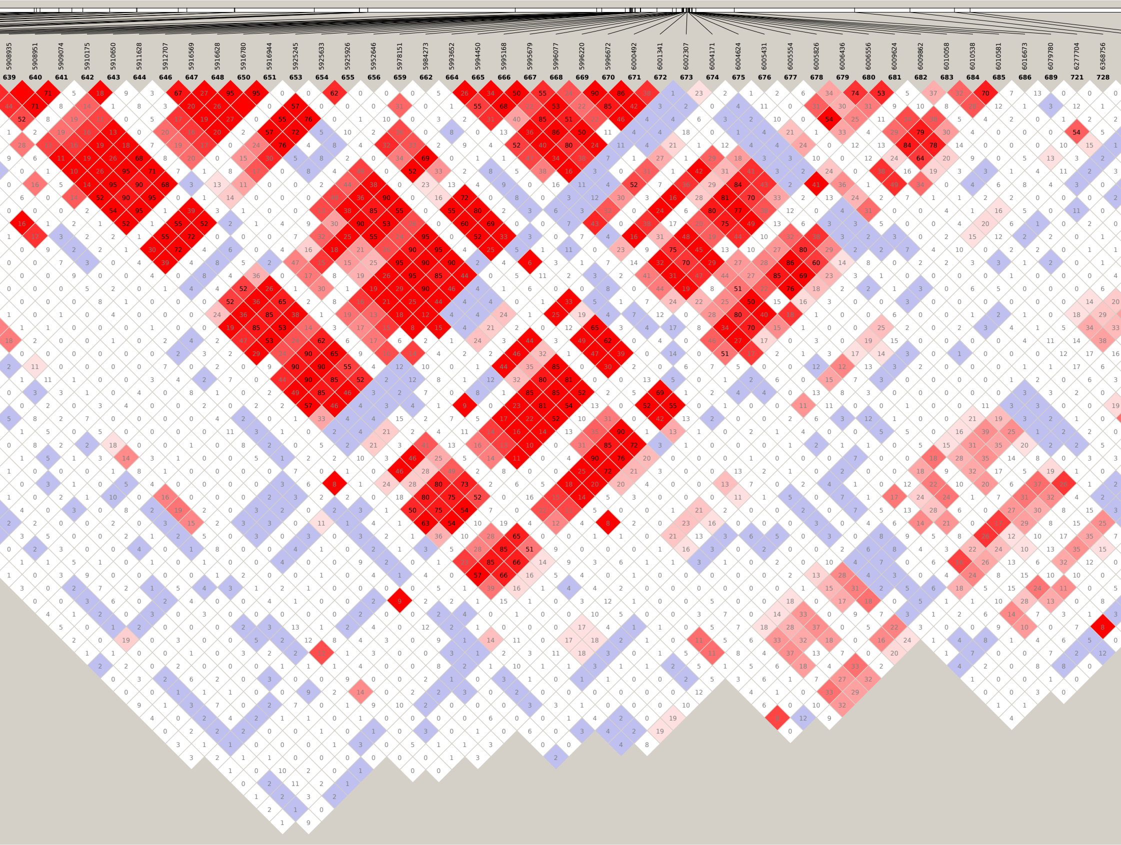
5908935 5908951 5909074 5910175 5910650 5911628 5912707 5916569 5916628 5916780 5916944 5925245 5925633 5925926 5952646 5978151 5984273 5993652 5994450 5995168 5995679 5996077 5996220 5996672 6000492 6001341 6002307 6004171 6004624 6005431 6005554 6005826 6006436 6006556 6009624 6009862 6010058 6010538 6010581 6016673 6079780 6277704 6368756
639 640 641 642 643 644 646 647 648 650 651 653 654 655 656 659 662 664 665 666 667 668 669 670 671 672 673 674 675 676 677 678 679 680 681 682 683 684 685 686 689 721 728

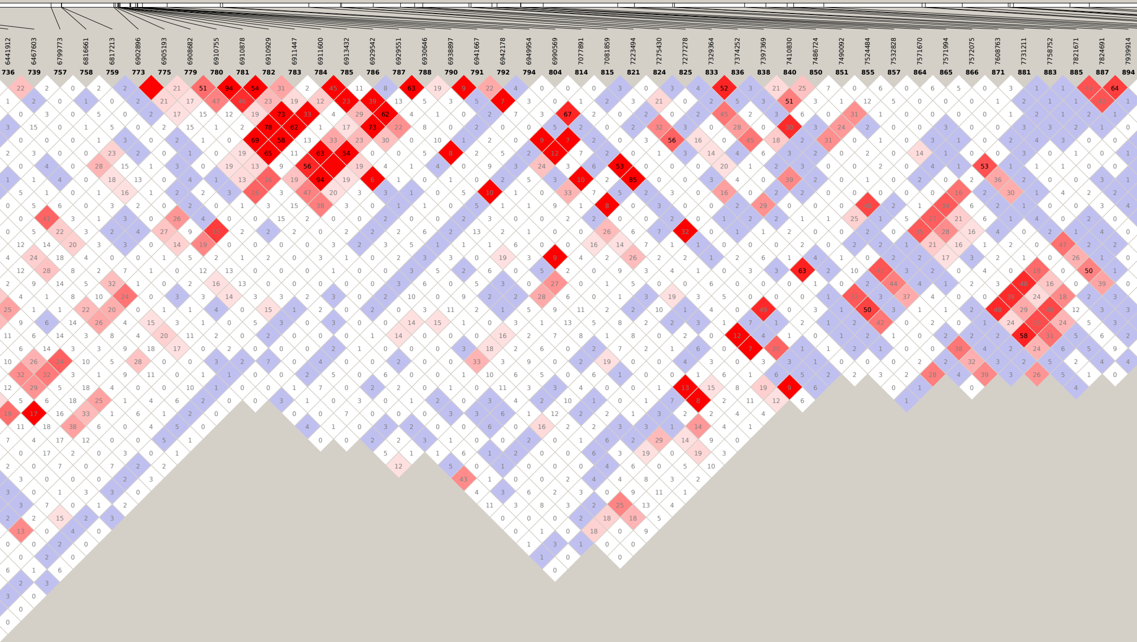
6441912
736
6467603
739
6799773
757
6816661
758
6817213
759
6902896
773
6905193
775
6908682
779
6910755
780
6910878
781
6910929
782
6911447
783
6911600
784
6913432
785
6929542
786
6929551
787
6930646
788
6938897
790
6941667
791
6942178
792
6949954
794
6990569
804
7077891
814
7081859
815
7223494
821
7275430
824
7277278
825
7329364
833
7374252
836
7397369
838
7410830
840
7486724
850
7490092
851
7524484
855
7532828
857
7571670
864
7571994
865
7572075
866
7608763
871
7731211
881
7758752
883
7821671
885
7824691
887
7939914
894

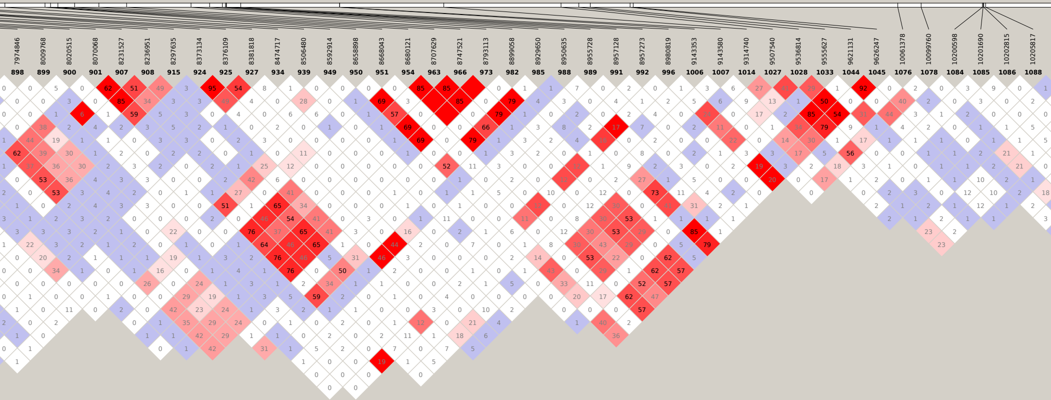

7974846 898
8009768 899
8020515 900
8070068 901
8231527 907
8236951 908
8297635 915
8373134 924
8376109 925
8381818 927
8474717 934
8506480 939
8592914 949
8658898 950
8668043 951
8680121 954
8707629 963
8747521 966
8793113 973
8899058 982
8929650 985
8950635 988
8955728 989
8957128 991
8957273 992
8980819 996
9143353 1006
9143580 1007
9314740 1014
9507540 1027
9536814 1028
9555627 1033
9621131 1044
9626247 1045
10061378 1076
10099760 1078
10200598 1084
10201690 1085
10202815 1086
10205817 1088

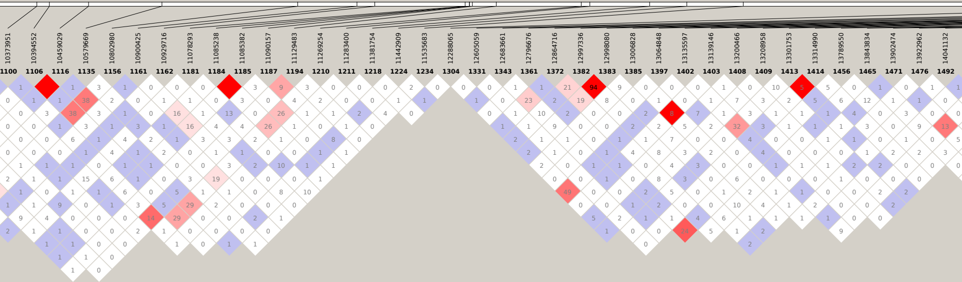
10373951
10394552
10459029
10579669
10802980
10900425
10929716
11078293
11085238
11085382
11090157
11129483
11269254
11283400
11381754
11442909
11535683
12288065
12605059
12683661
12796676
12864716
12997336
12998080
13006828
13064848
13135597
13139146
13200466
13208958
13301753
13314990
13789550
13843834
13902474
13922962
14041132
1100
1106
1116
1135
1156
1161
1162
1181
1184
1185
1187
1194
1210
1211
1218
1224
1234
1304
1331
1343
1361
1372
1382
1383
1385
1397
1402
1403
1408
1409
1413
1414
1456
1465
1471
1476
1492

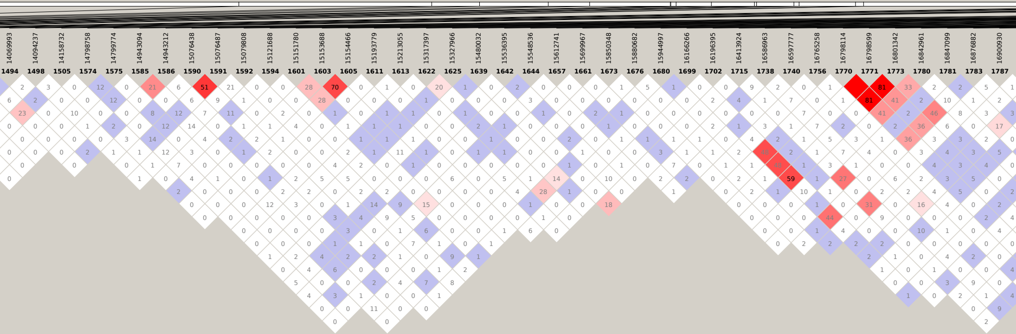

14069993 14094237 14158732 14798758 14799774 14943094 14943212 15076438 15076487 15079808 15121688 15151780 15153688 15154466 15193779 15213055 15317397 15327966 15480032 15536395 15548536 15612741 15699967 15850348 15880682 15944997 16166266 16196395 16413924 16586963 16597777 16765258 16798114 16798599 16801342 16842961 16847099 16876882 16900930
1494 1498 1505 1574 1575 1585 1586 1590 1591 1592 1594 1601 1603 1605 1611 1613 1622 1625 1639 1642 1644 1657 1661 1673 1676 1680 1699 1702 1715 1738 1740 1756 1770 1771 1773 1780 1781 1783 1787

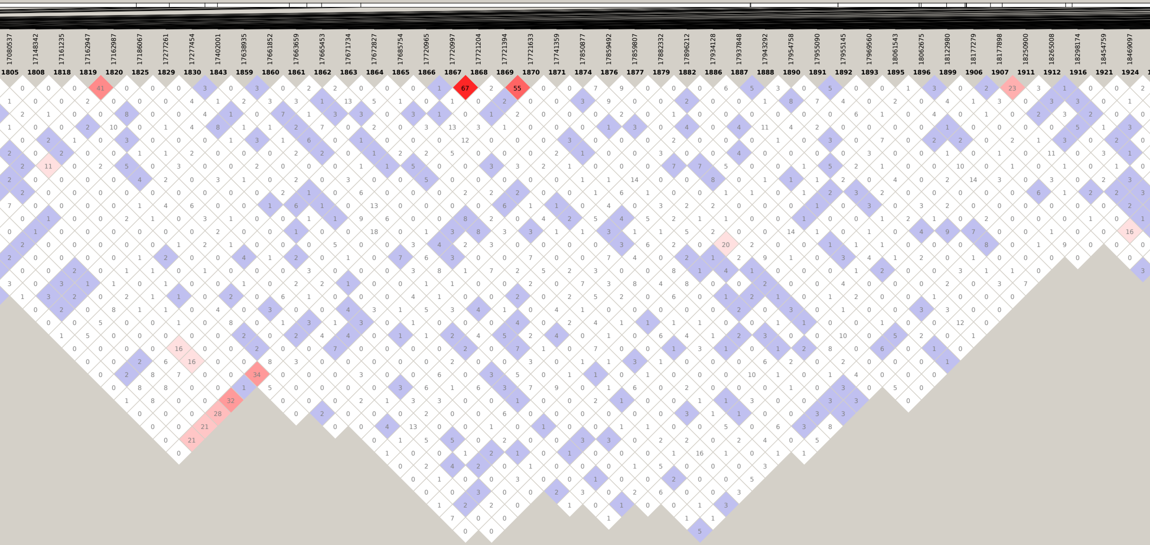
17080537 17148342 17161235 17162947 17162987 17186067 17277261 17277454 17402001 17638935 17661852 17663659 17665453 17671734 17672827 17685754 17720965 17720997 17721204 17721394 17721633 17741359 17850877 17859492 17859807 17882332 17896212 17934128 17937848 17943292 17954758 17955090 17955145 17969560 18061543 18062675 18122980 18177279 18177898 18250900 18265008 18298174 18454759 18469097
1805 1808 1818 1819 1820 1825 1829 1830 1843 1859 1860 1861 1862 1863 1864 1865 1866 1867 1868 1869 1870 1871 1874 1876 1877 1879 1882 1886 1887 1888 1890 1891 1892 1893 1895 1896 1899 1906 1907 1911 1912 1916 1921 1924

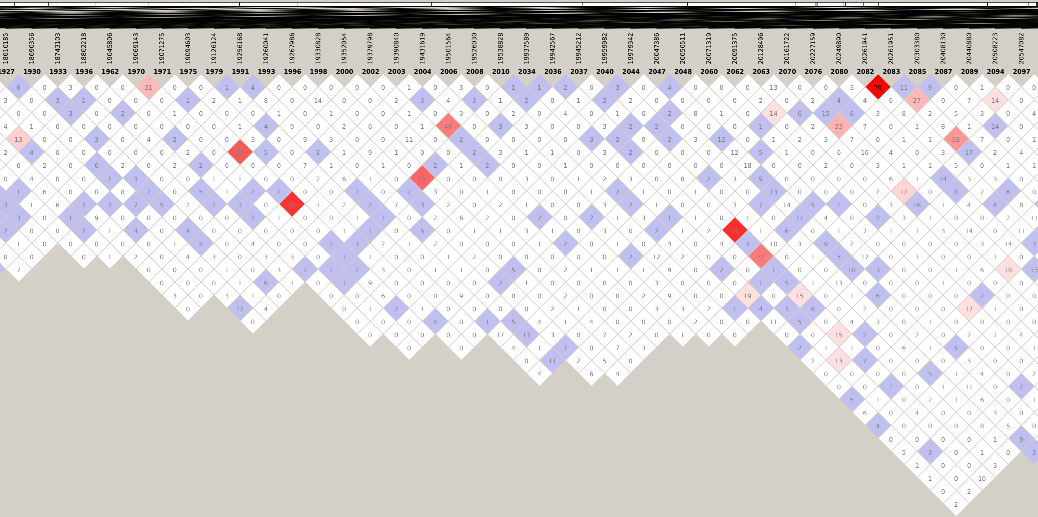
18610185 18690356 18743103 18802218 19045806 19069143 19071275 19094603 19126124 19256168 19260041 19267986 19330828 19352054 19379798 19390840 19431619 19501564 19526030 19538828 19937589 19942567 19945212 19959982 19979342 20047386 20050511 20071319 20091375 20128496 20161722 20227159 20249890 20261941 20261951 20303380 20408130 20440880 20508223 20547082
1927 1930 1933 1936 1962 1970 1971 1975 1979 1991 1993 1996 1998 2000 2002 2003 2004 2006 2008 2010 2034 2036 2037 2040 2044 2047 2048 2060 2062 2063 2070 2076 2080 2082 2083 2085 2087 2089 2094 2097

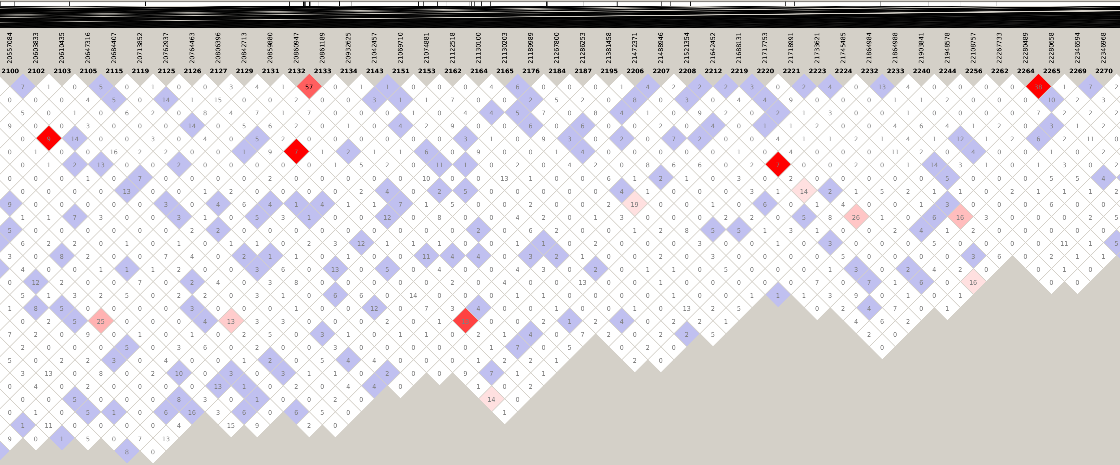
20557084
2100
20603833
2102
20610435
2103
20647316
2105
20684407
2115
20713852
2119
20762937
2125
20764463
2126
20806396
2127
20842713
2129
20859880
2131
20860947
2132
20861189
2133
20932625
2134
21042457
2143
21069710
2151
21074881
2153
21122518
2162
21130100
2164
21130203
2165
21189989
2176
21267800
2184
21286253
2187
21381458
2195
21472371
2206
21488946
2207
21521354
2208
21642452
2212
21688131
2219
21717753
2220
21718991
2221
21733621
2223
21745485
2224
21864984
2232
21864988
2233
21903841
2240
21948578
2244
22108757
2256
22267733
2262
22280489
2264
22280658
2265
22346594
2269
22346968
2270

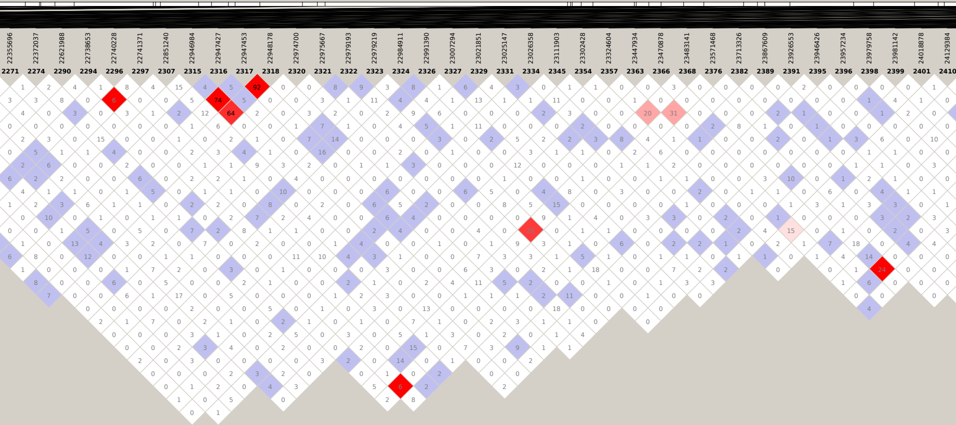
22355696 22372037 22621988 22738653 22740228 22741371 22851240 22946984 22947427 22947453 22948178 22974700 22975667 22979193 22979219 22984911 22991390 23007294 23021851 23025147 23026358 23111903 23302428 23324604 23447934 23470878 23483141 23571468 23713326 23867609 23926553 23946426 23957234 23979758 23981142 24018878 24129384
2271 2274 2290 2294 2296 2297 2307 2315 2316 2317 2318 2320 2321 2322 2323 2324 2326 2327 2329 2331 2334 2345 2354 2357 2363 2366 2368 2376 2382 2389 2391 2395 2396 2398 2399 2401 2410

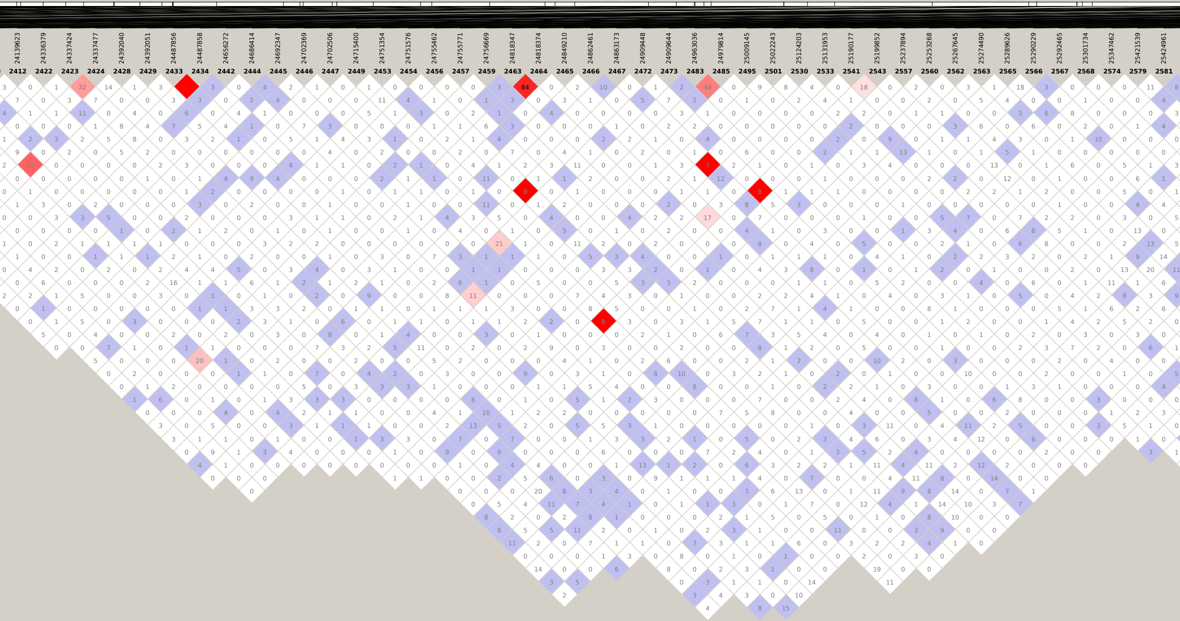
24139623 24336379 24337424 24337477 24392040 24392051 24487856 24487858 24656272 24686414 24692347 24702369 24702506 24715400 24751354 24751576 24755462 24755771 24756669 24818347 24818374 24849210 24862461 24863173 24909448 24909644 24963036 24979814 25009145 25022243 25124203 25131953 25190177 25199852 25237894 25253268 25267645 25274490 25289626 25290229 25292465 25301734 25347462 25421539 25424961
2412 2422 2423 2424 2428 2429 2433 2434 2442 2444 2445 2446 2447 2449 2453 2454 2456 2457 2459 2463 2464 2465 2466 2467 2472 2473 2483 2485 2495 2501 2530 2533 2541 2543 2557 2560 2562 2563 2565 2566 2567 2568 2574 2579 2581

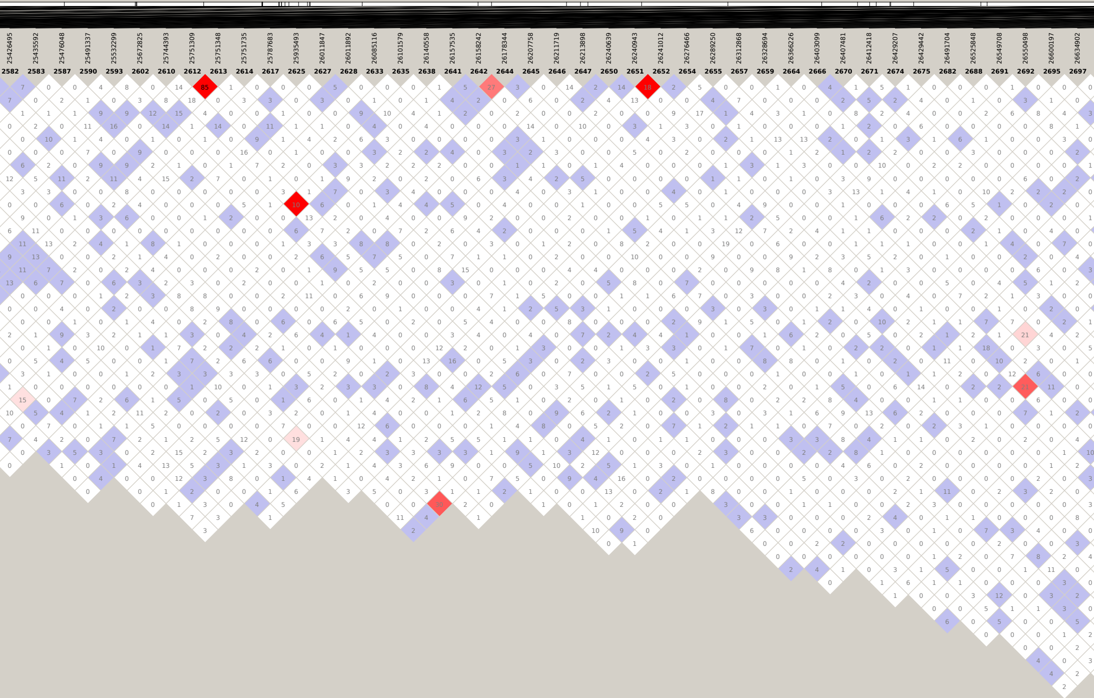

25426495 25435592 25476048 25491337 25532299 25672825 25744393 25751309 25751348 25751735 25787683 25935493 26011847 26011892 26085116 26101579 26140558 26157535 26158242 26178344 26207758 26211719 26213898 26240639 26240943 26241012 26276466 26289250 26312868 26328694 26366226 26403099 26407481 26412418 26429207 26429442 26491704 26525848 26549708 26550498 26600197 26634902
2582 2583 2587 2590 2593 2602 2610 2612 2613 2614 2617 2625 2627 2628 2633 2635 2638 2641 2642 2644 2645 2646 2647 2650 2651 2652 2654 2655 2657 2659 2664 2666 2670 2671 2674 2675 2682 2688 2691 2692 2695 2697

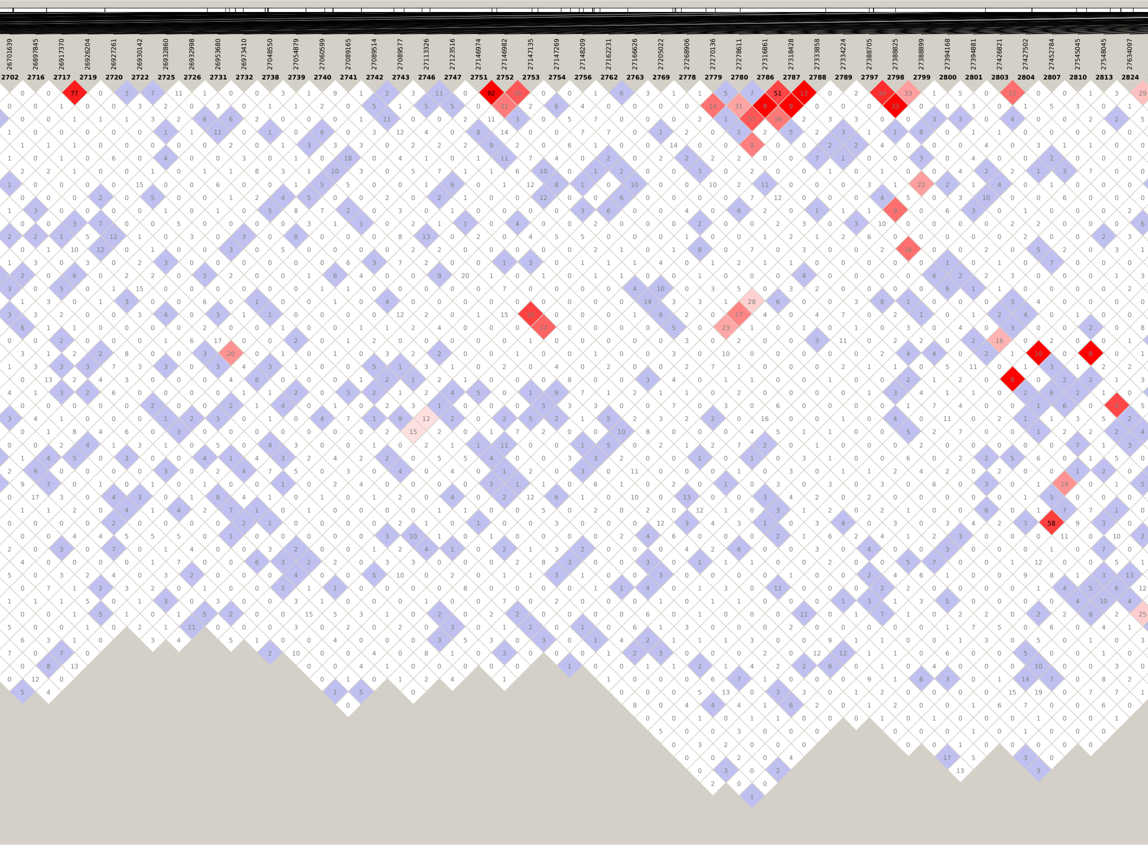
26701639
26897845
26917370
26926204
26927261
26930142
26932860
26932998
26953680
26973410
27048550
27054879
27060599
27089165
27089514
27089577
27113326
27123516
27146974
27146982
27147135
27147269
27148209
27162231
27166626
27205022
27268906
27270136
27278611
27316861
27318428
27333858
27334224
27388705
27388825
27388899
27394168
27394881
27426821
27427502
27452784
27545045
27548045
27634097
2702 2716 2717 2719 2720 2722 2725 2726 2731 2732 2738 2739 2740 2741 2742 2743 2746 2747 2751 2752 2753 2754 2756 2762 2763 2769 2778 2779 2780 2786 2787 2788 2789 2797 2798 2799 2800 2801 2803 2804 2807 2810 2813 2824

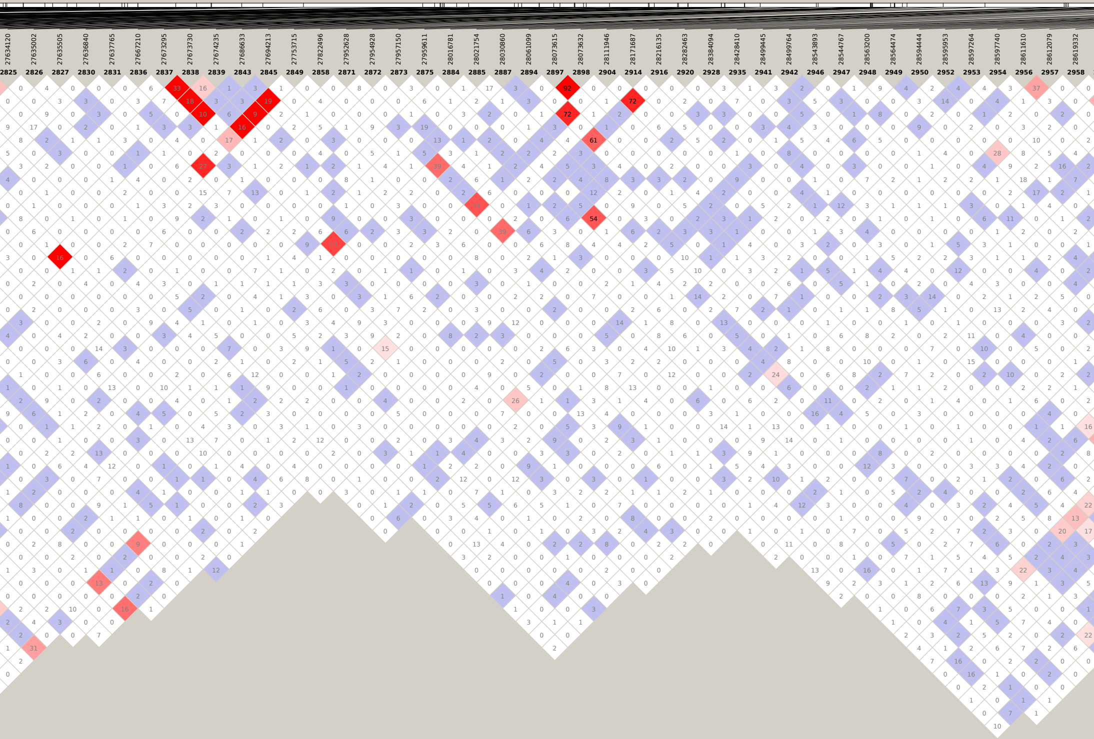
27634120
27635002
27635505
27636840
27637765
27667210
27673295
27673730
27674235
27686633
27694213
27753715
27822496
27952628
27954928
27957150
27959611
28016781
28021754
28030860
28061099
28073615
28073632
28111946
28171687
28216135
28282463
28384094
28428410
28499445
28499764
28543893
28544767
28563200
28564474
28594444
28595953
28597264
28597740
28611610
28612079
28619332
2825 2826 2827 2830 2831 2836 2837 2838 2839 2843 2845 2849 2858 2871 2872 2873 2875 2884 2885 2887 2894 2897 2898 2904 2914 2916 2920 2928 2935 2941 2942 2946 2947 2948 2949 2950 2952 2953 2954 2956 2957 2958

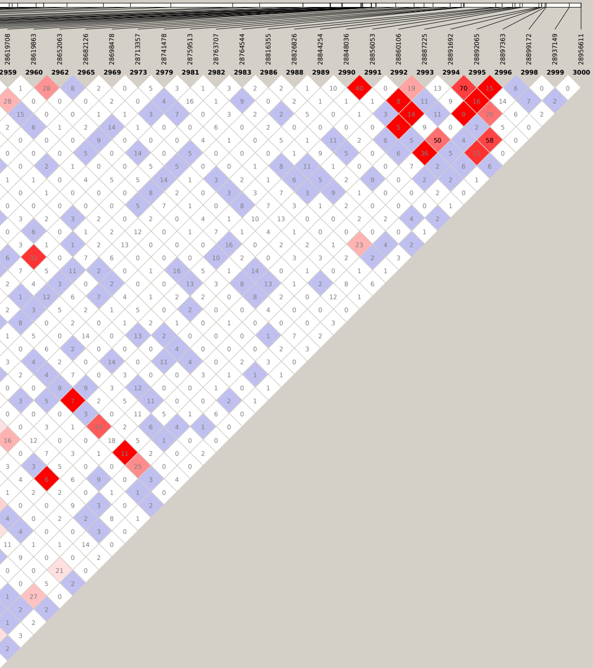

28619708
28619863
28652063
28682126
28698478
28713357
28741478
28759513
28763707
28764544
28816355
28826826
28844254
28848036
28856053
28860106
28887225
28891692
28892065
28897363
28899172
28937149
28956611
2959
2960
2962
2965
2969
2973
2979
2981
2982
2983
2986
2988
2989
2990
2991
2992
2993
2994
2995
2996
2998
2999
3000

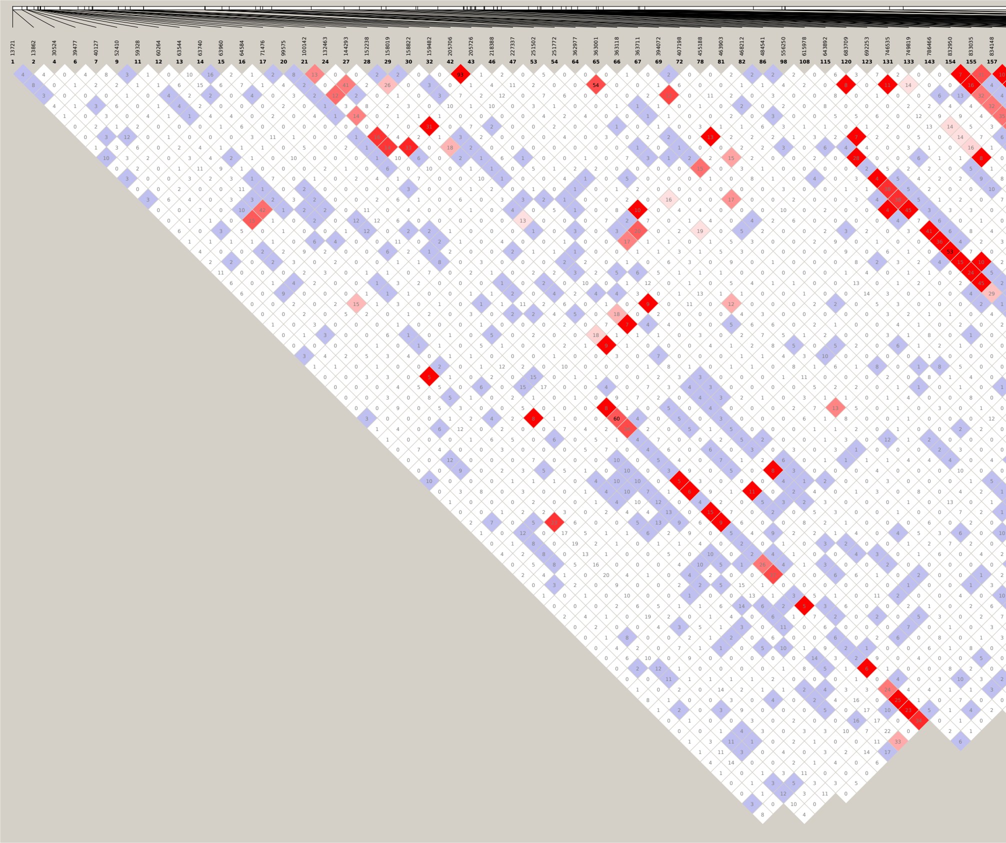

13721
13862
30524
39477
40127
52410
59328
60264
63544
63740
63960
64584
71476
99575
100142
132463
144293
152238
158019
158822
159482
205706
205726
218388
227337
251502
251772
362977
363001
363118
363711
394072
407198
455188
463903
468212
484541
556250
615978
643892
683709
692253
746535
749819
786466
832950
833035
834148
1 2 4 6 7 9 11 12 13 14 15 16 17 20 21 24 27 28 29 30 32 42 43 46 47 53 54 64 65 66 67 69 72 78 81 82 86 98 108 115 120 123 131 133 143 154 155 157

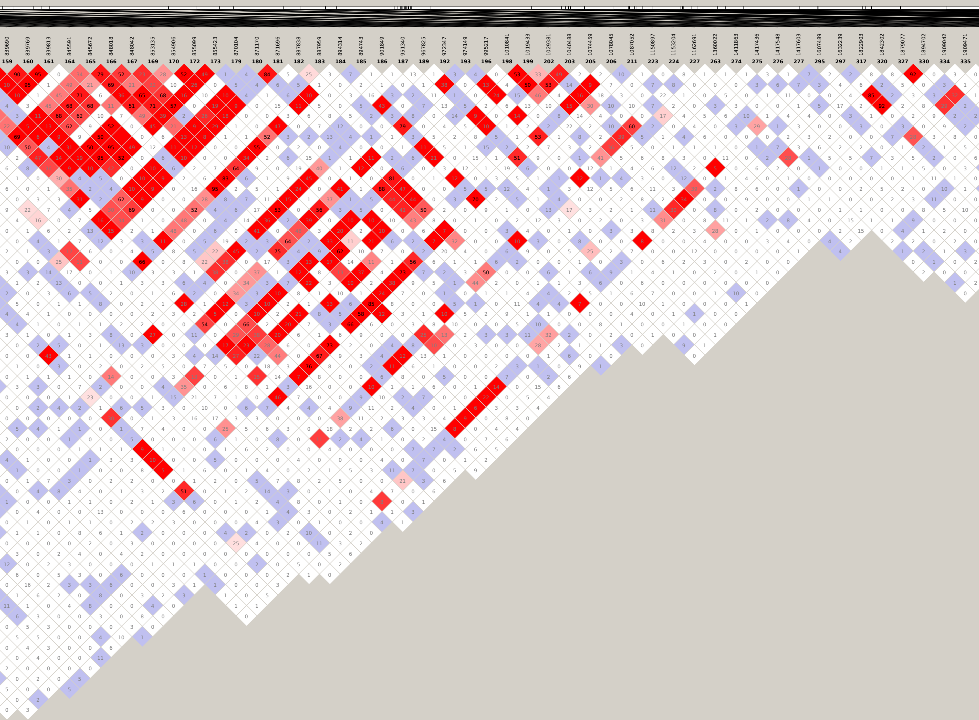
839690 839769 839813 845591 845672 848018 848042 853135 854906 855099 855423 870104 871170 871696 887838 887959 894314 894743 901849 951340 967825 972347 974149 995217 1010841 1019433 1029381 1040488 1074459 1078045 1087052 1150897 1153204 1162691 1360022 1411863 1417436 1417548 1417603 1607489 1632239 1822903 1842302 1879077 1894702 1909042 1909471
159 160 161 164 165 166 167 169 170 172 173 179 180 181 182 183 184 185 186 187 189 192 193 196 198 199 202 203 205 206 211 223 224 227 263 274 275 276 277 295 297 317 320 327 330 334 335

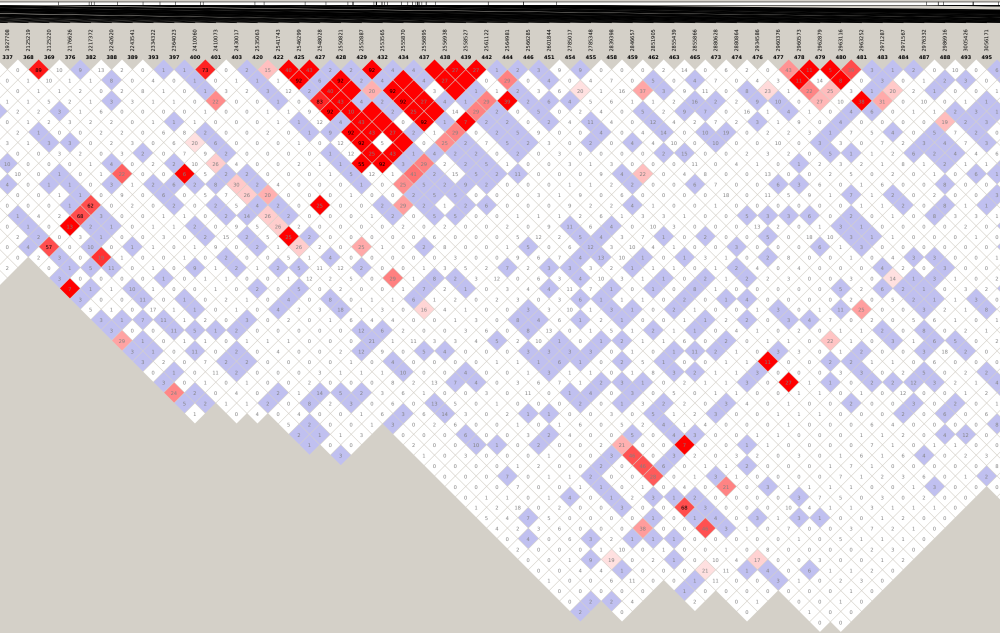

1927708
2125219
2125220
2176626
2217372
2242620
2243541
2334322
2364023
2410060
2410073
2430017
2535063
2541743
2546299
2548028
2550821
2552887
2553565
2555870
2556895
2556938
2558527
2561122
2564981
2566285
2601844
2785017
2785348
2839398
2846657
2851905
2855439
2855866
2880628
2880864
2934586
2960376
2960573
2962879
2963116
2963252
2971287
2971567
2976332
2986916
3005426
3056171
337 368 369 376 382 388 389 393 397 400 401 403 420 422 425 427 428 429 432 434 437 438 439 442 444 446 451 454 455 458 459 462 463 465 473 474 476 477 478 479 480 481 483 484 487 488 493 495

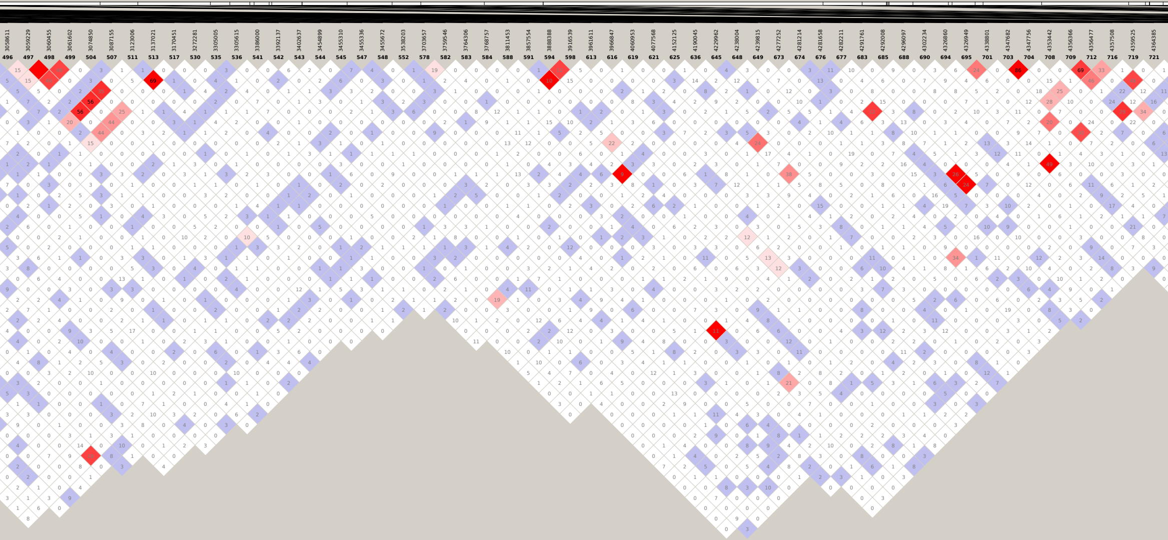
3058611
496
3059229
497
3060455
498
3061602
499
3074850
504
3087155
507
3123006
511
3137021
513
3170451
517
3272281
530
3305005
535
3305615
536
3386000
541
3392137
542
3402637
543
3454899
544
3455310
545
3455336
547
3455672
548
3538203
552
3703657
578
3759546
582
3764306
583
3768757
584
3811453
588
3857554
591
3888388
594
3916539
598
3961611
613
3966847
616
4060953
619
4077568
621
4152125
625
4190045
636
4229962
645
4238004
648
4239815
649
4277252
673
4281214
674
4281658
676
4282211
677
4291761
683
4292008
685
4296097
688
4302234
690
4326860
694
4326949
695
4338801
701
4347682
703
4347756
704
4353442
708
4356366
709
4356477
712
4357508
716
4359525
719
4364385
721

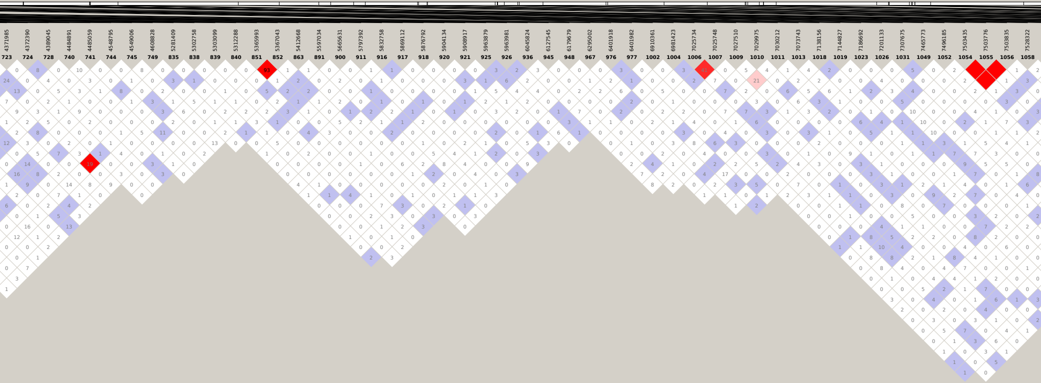

4371985 723
4372390 724
4389045 728
4484891 740
4485059 741
4548795 744
4549006 745
4608828 749
5281409 835
5302758 838
5303099 839
5312288 840
5365993 851
5367043 852
5412668 863
5597034 891
5665631 900
5797392 911
5832758 916
5869112 917
5876792 918
5904134 920
5908917 921
5963879 925
5963981 926
6045824 936
6127545 945
6179679 948
6295002 967
6401918 976
6401982 977
6910361 1002
6981423 1004
7025734 1006
7025748 1007
7027510 1009
7029975 1010
7030212 1011
7073743 1013
7138156 1018
7144827 1019
7186692 1023
7201133 1026
7307675 1031
7465773 1049
7496185 1052
7503435 1054
7503776 1055
7503835 1056
7528322 1058

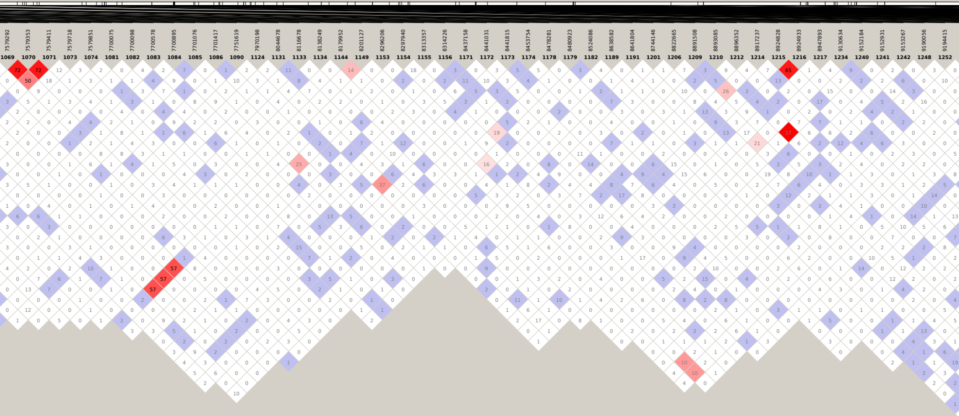
7579292 7579353 7579411 7579718 7579851 7700075 7700098 7700578 7700895 7701076 7701417 7751619 7970198 8044678 8116678 8138249 8179952 8201127 8296206 8297940 8313357 8314226 8437158 8441031 8441815 8453754 8478281 8480923 8524086 8638582 8641804 8744146 8822665 8891508 8893085 8896352 8917237 8924828 8924933 8947893 9130634 9152184 9152931 9153267 9190056 9194415
1069 1070 1071 1073 1074 1081 1082 1083 1084 1085 1086 1090 1124 1131 1133 1134 1144 1149 1153 1154 1155 1156 1171 1172 1173 1174 1178 1179 1182 1189 1191 1201 1206 1209 1210 1212 1214 1215 1216 1217 1234 1240 1241 1242 1248 1252

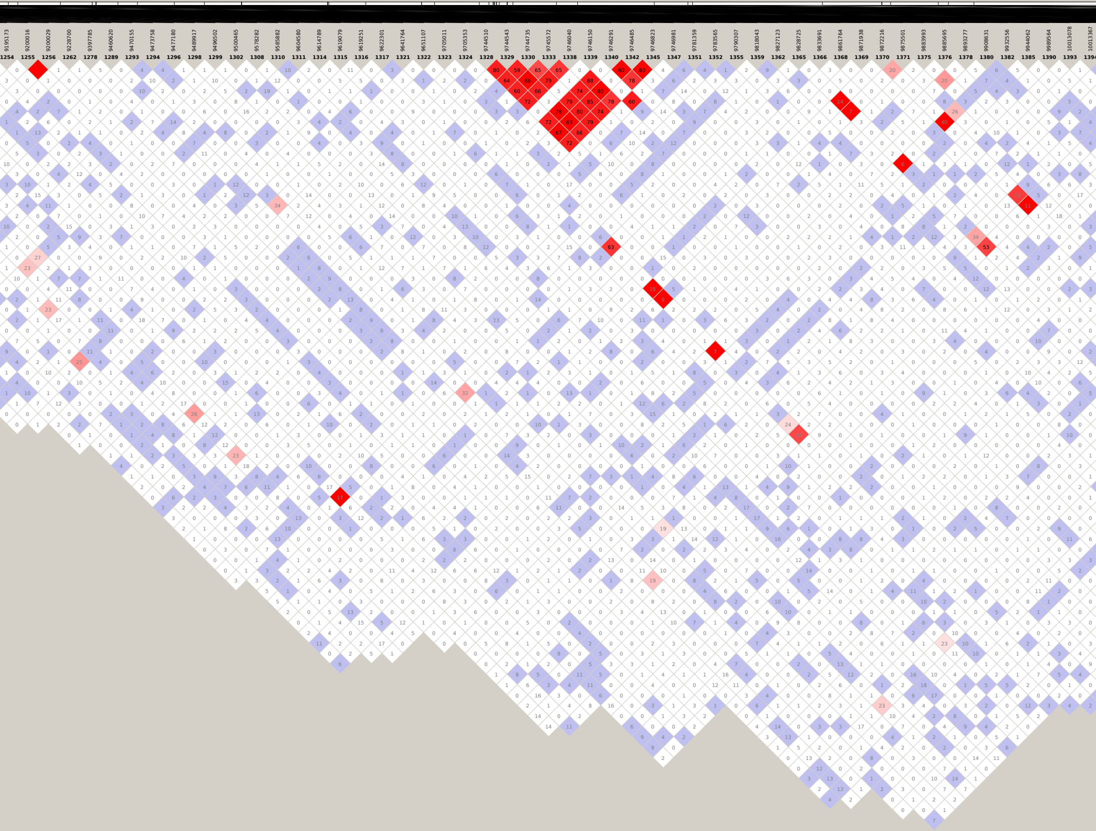
9195173
9200016
9200029
9228700
9397785
9460620
9470155
9473758
9477180
9489917
9496502
9500465
9578282
9585882
9604580
9614789
9619079
9619251
9622301
9641764
9651107
9705011
9705353
9744510
9744543
9744735
9745572
9746040
9746150
9746291
9746485
9746823
9746981
9781359
9783565
9790307
9818043
9827123
9828725
9833691
9861764
9871938
9872216
9875501
9883993
9885695
9893277
9908631
9932556
9944062
9989564
10013078
10013367
1254
1255
1256
1262
1278
1289
1293
1294
1296
1298
1299
1302
1308
1310
1311
1314
1315
1316
1317
1321
1322
1323
1324
1328
1329
1330
1333
1338
1339
1340
1342
1345
1347
1351
1352
1355
1359
1362
1365
1366
1368
1369
1370
1371
1375
1376
1378
1380
1382
1385
1390
1393
1394

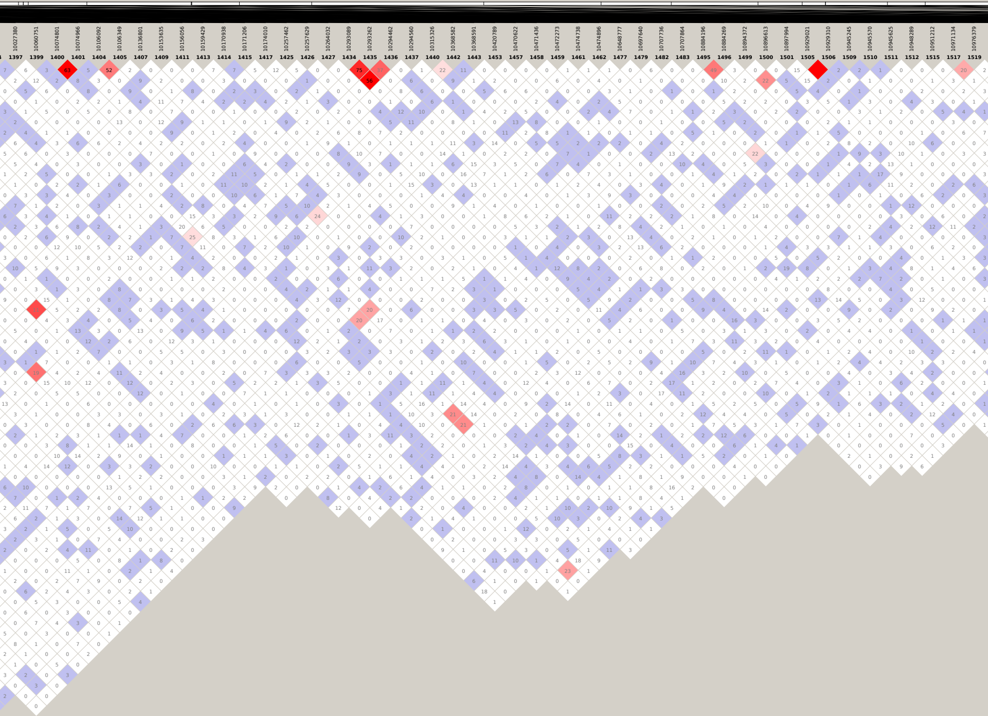
10027380 10060751 10074801 10074966 10106092 10106349 10136801 10153635 10156056 10159429 10170938 10171206 10174010 10257462 10257629 10264032 10293089 10293262 10294462 10294560 10315326 10368582 10368591 10420789 10470622 10471436 10472273 10474738 10474896 10648777 10697640 10707736 10707864 10884196 10884269 10894372 10896613 10897994 10929021 10929310 10945245 10945570 10945625 10948289 10951212 10971134 10976379
1397 1399 1400 1401 1404 1405 1407 1409 1411 1413 1414 1415 1417 1425 1426 1427 1434 1435 1436 1437 1440 1442 1443 1453 1457 1458 1459 1461 1462 1477 1479 1482 1483 1495 1496 1499 1500 1501 1505 1506 1509 1510 1511 1512 1515 1517 1519

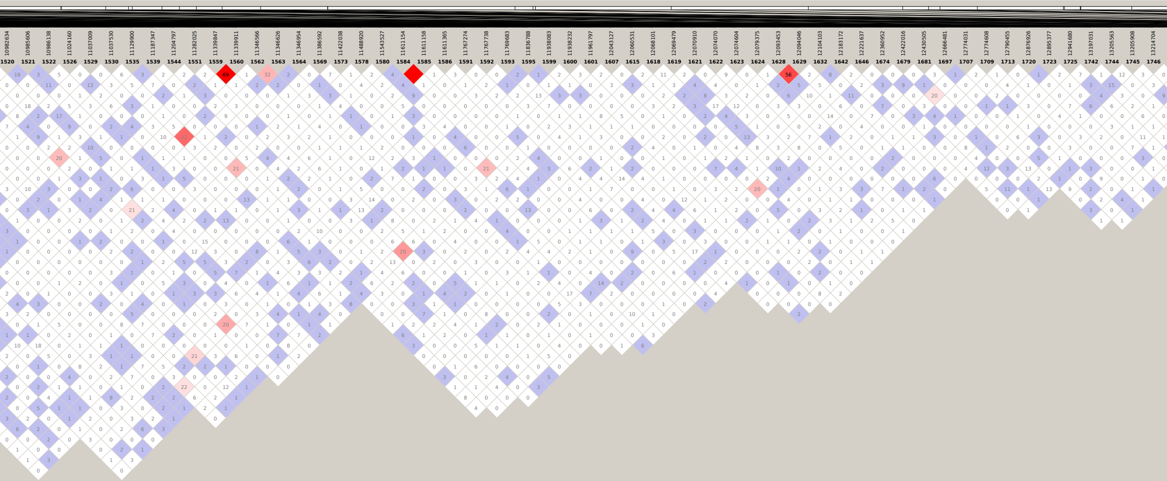
10982634 10985606 10986138 11024160 11037009 11037530 11129900 11187347 11204797 11282025 11339847 11339911 11346566 11346626 11346954 11386592 11422038 11488920 11543527 11611154 11611158 11611365 11767274 11767738 11769683 11836788 11938083 11938232 11961797 12043127 12060531 12068101 12069479 12070910 12074070 12074604 12079375 12093453 12094046 12104103 12183172 12221637 12360952 12422016 12430505 12666481 12774031 12774608 12790455 12876926 12895377 12941680 13197031 13205563 13205908 13214704
1520 1521 1522 1526 1529 1530 1535 1539 1544 1551 1559 1560 1562 1563 1564 1569 1573 1578 1580 1584 1585 1586 1591 1592 1593 1595 1599 1600 1601 1607 1615 1618 1619 1621 1622 1623 1624 1628 1629 1632 1642 1646 1674 1679 1681 1697 1707 1709 1713 1720 1723 1725 1742 1744 1745 1746

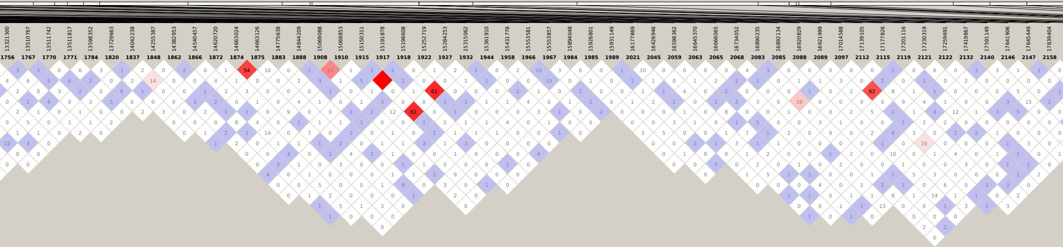
13321300 13510767 13511742 13511817 13598352 13729663 14042238 14255387 14382953 14540457 14620720 14663024 14663126 14775638 14844209 15069088 15069853 15150311 15191878 15198608 15252719 15284253 15315062 15361910 15431778 15553581 15553857 15894048 15926801 15931149 16177869 16426946 16598362 16645370 16666065 16734052 16890235 16892134 16920829 16921999 17014588 17139105 17177826 17201116 17230319 17259691 17419867 17591149 17641906 17645449 17839404
1756 1767 1770 1771 1784 1820 1837 1848 1862 1866 1872 1874 1875 1883 1888 1908 1910 1915 1917 1918 1922 1927 1932 1944 1958 1966 1967 1984 1985 1989 2021 2045 2059 2063 2065 2068 2083 2085 2088 2089 2097 2112 2115 2119 2121 2122 2132 2140 2146 2147 2158

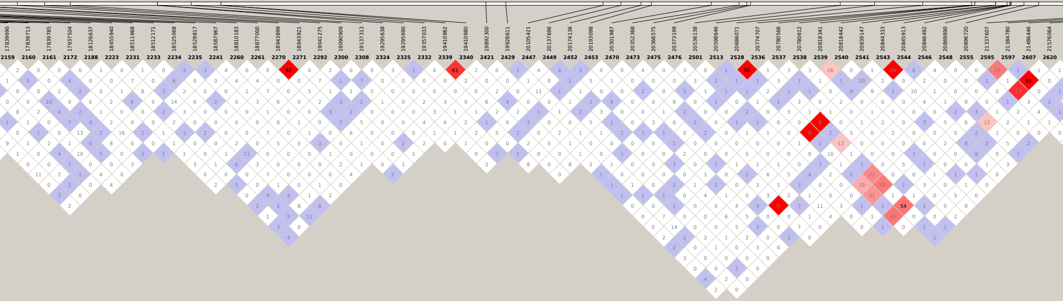
17839690 17839713 17839785 17937504 18126637 18455940 18511968 18512171 18525068 18529817 18587967 18810183 18877000 18943899 18943921 19041275 19090909 19137313 19295638 19295690 19357033 19410962 19410980 19892300 19928611 20105421 20137696 20174136 20193098 20301987 20352368 20366575 20373199 20536138 20598646 20686071 20774707 20780566 20780612 20818341 20818442 20839147 20844333 20845913 20846492 20869890 20896720 21337607 21384780 21486446 21576064
2159 2160 2161 2172 2188 2223 2231 2233 2234 2235 2241 2260 2261 2270 2271 2292 2300 2308 2324 2325 2332 2339 2340 2421 2429 2447 2449 2452 2453 2470 2473 2475 2476 2501 2513 2528 2536 2537 2538 2539 2540 2541 2543 2544 2546 2548 2555 2595 2597 2607 2620

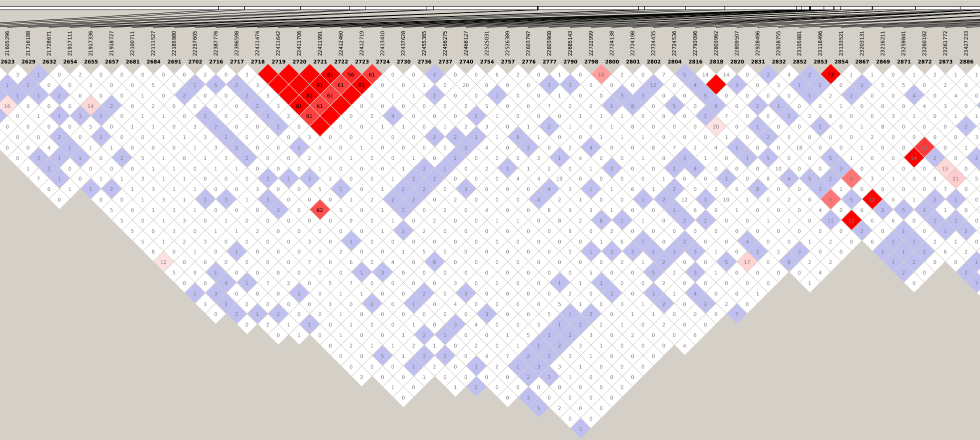
21605296 21716188 21728671 21917111 21917336 21918727 22100711 22111527 22185980 22257605 22387776 22396598 22411474 22411642 22411706 22411991 22412460 22412719 22413410 22437628 22455365 22456275 22468127 22525031 22526389 22603797 22603908 22685143 22722999 22724138 22724198 22724435 22724536 22793096 22803962 22809507 22928496 22928755 23105881 23118496 23133521 23203131 23216211 23259841 23260192 23261772 23427233
2623 2629 2632 2654 2655 2657 2681 2684 2691 2702 2716 2717 2718 2719 2720 2721 2722 2723 2724 2730 2736 2737 2740 2754 2757 2776 2777 2790 2798 2800 2801 2802 2804 2816 2818 2820 2831 2832 2852 2853 2854 2867 2869 2871 2872 2873 2886

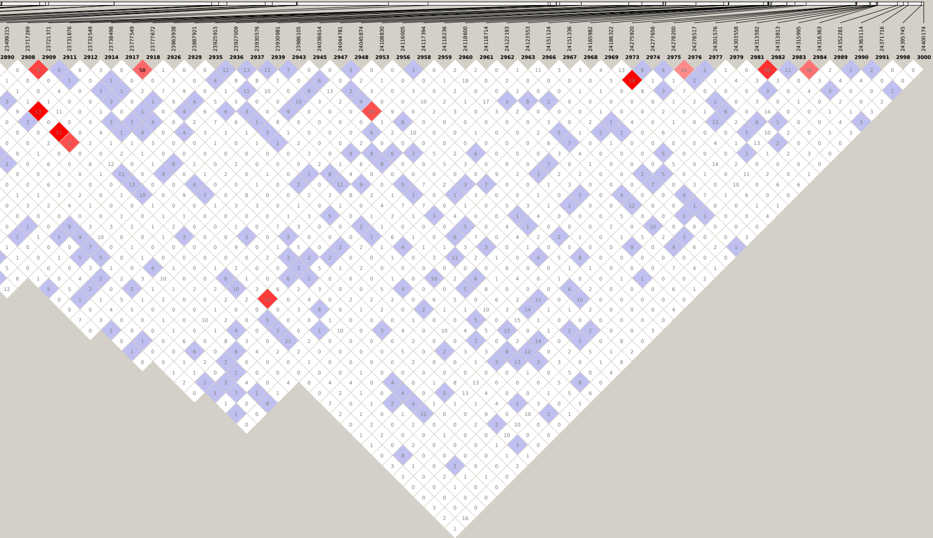
23499215 23717269 23721371 23731876 23732549 23738498 23777549 23777672 23863938 23887921 23925915 23927009 23930576 23930981 23986105 24036614 24044781 24045874 24108830 24116005 24117394 24118236 24118600 24118714 24122193 24123553 24151124 24151336 24165982 24186322 24275920 24277656 24278200 24278517 24301576 24303558 24313592 24313813 24315995 24316363 24352281 24363114 24371716 24395745 24400174
2890 2908 2909 2911 2912 2914 2917 2918 2926 2929 2935 2936 2937 2939 2943 2945 2947 2948 2953 2956 2958 2959 2960 2961 2962 2963 2966 2967 2968 2969 2973 2974 2975 2976 2977 2979 2981 2982 2983 2984 2989 2990 2991 2998 3000

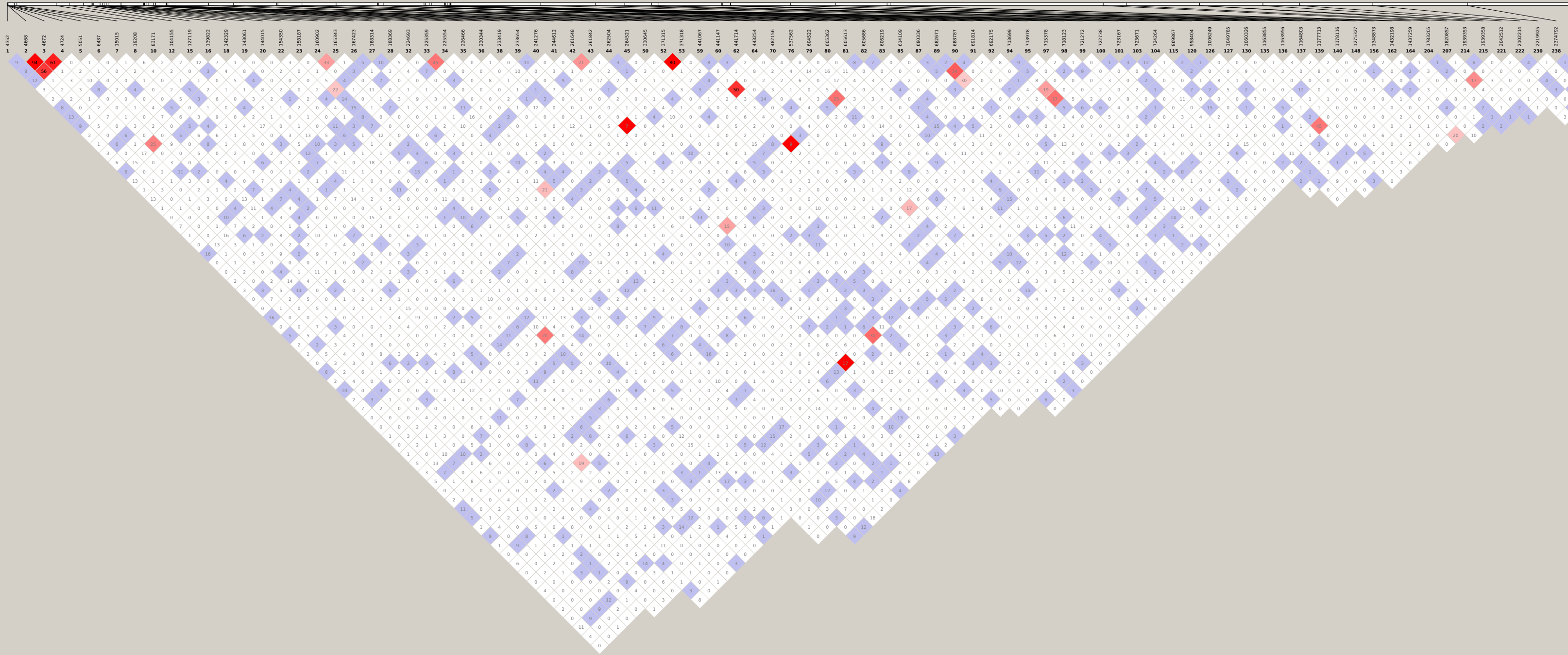

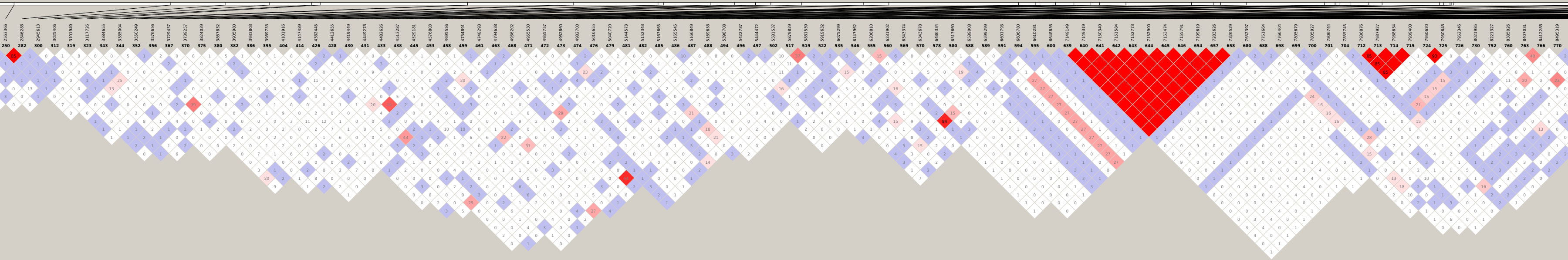

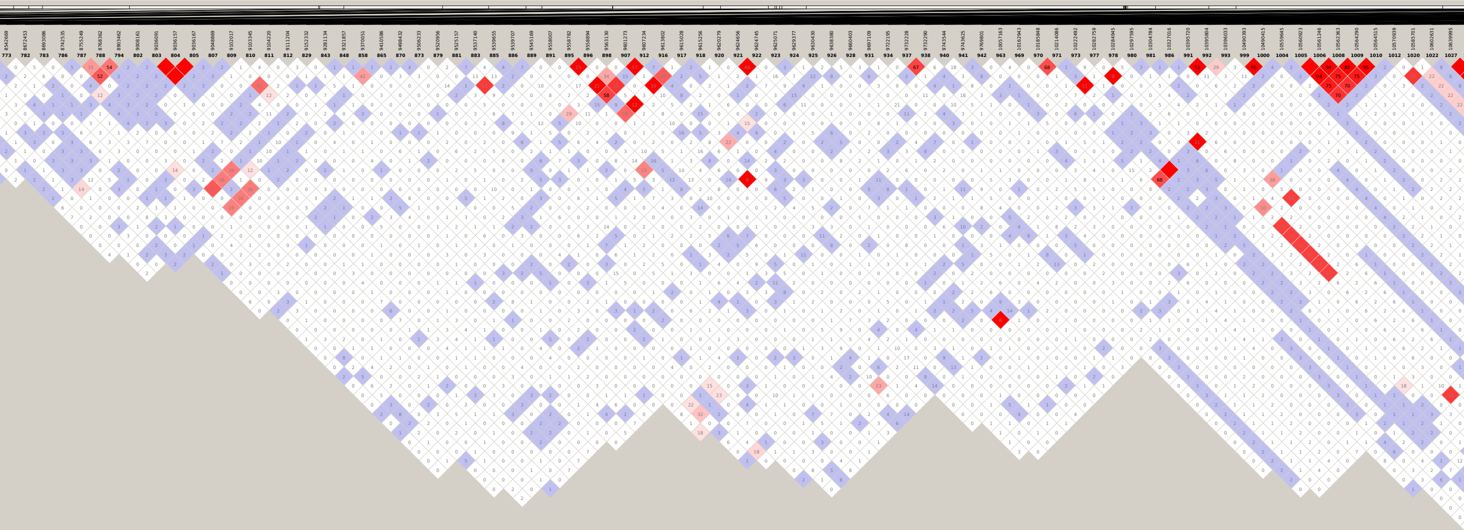

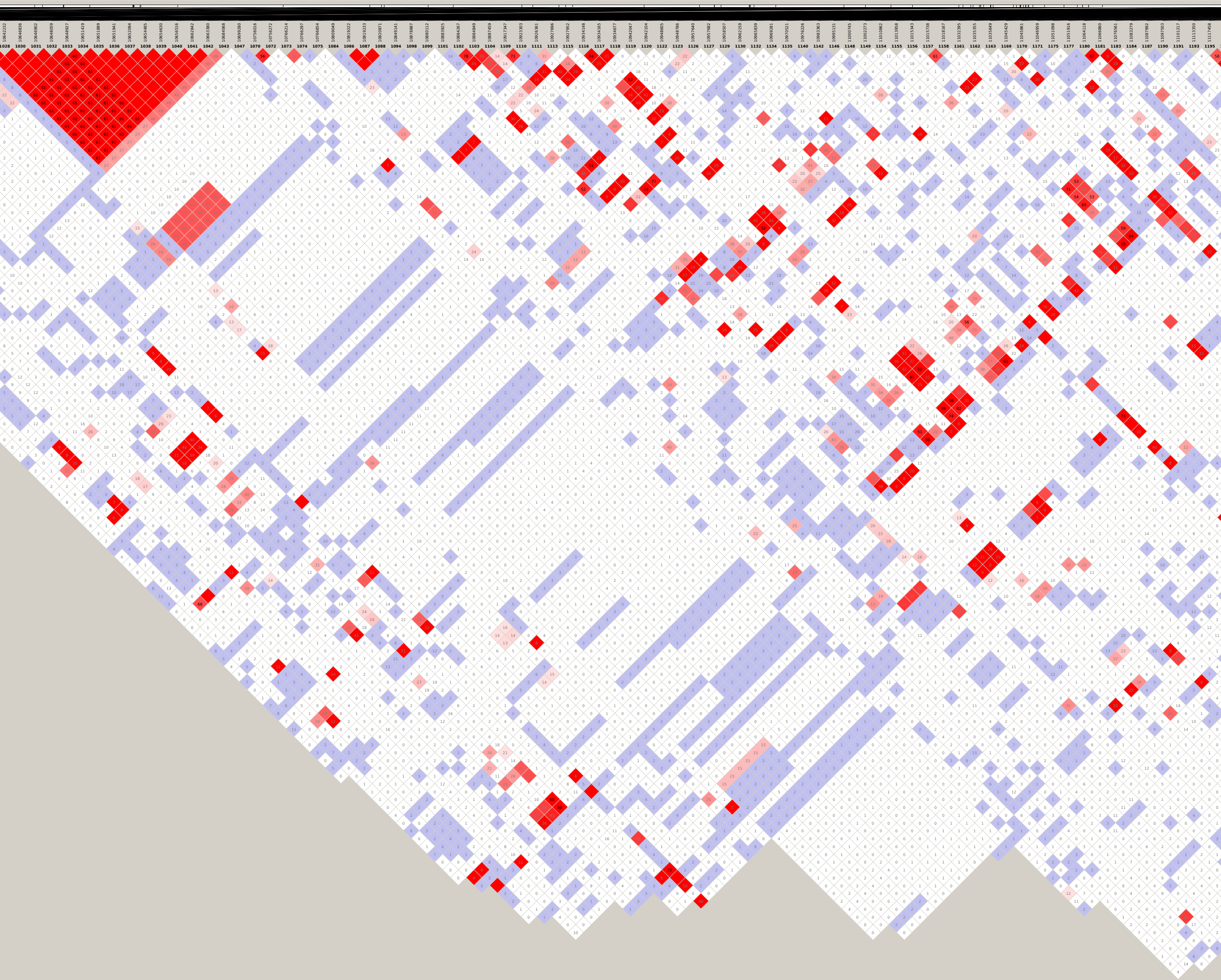

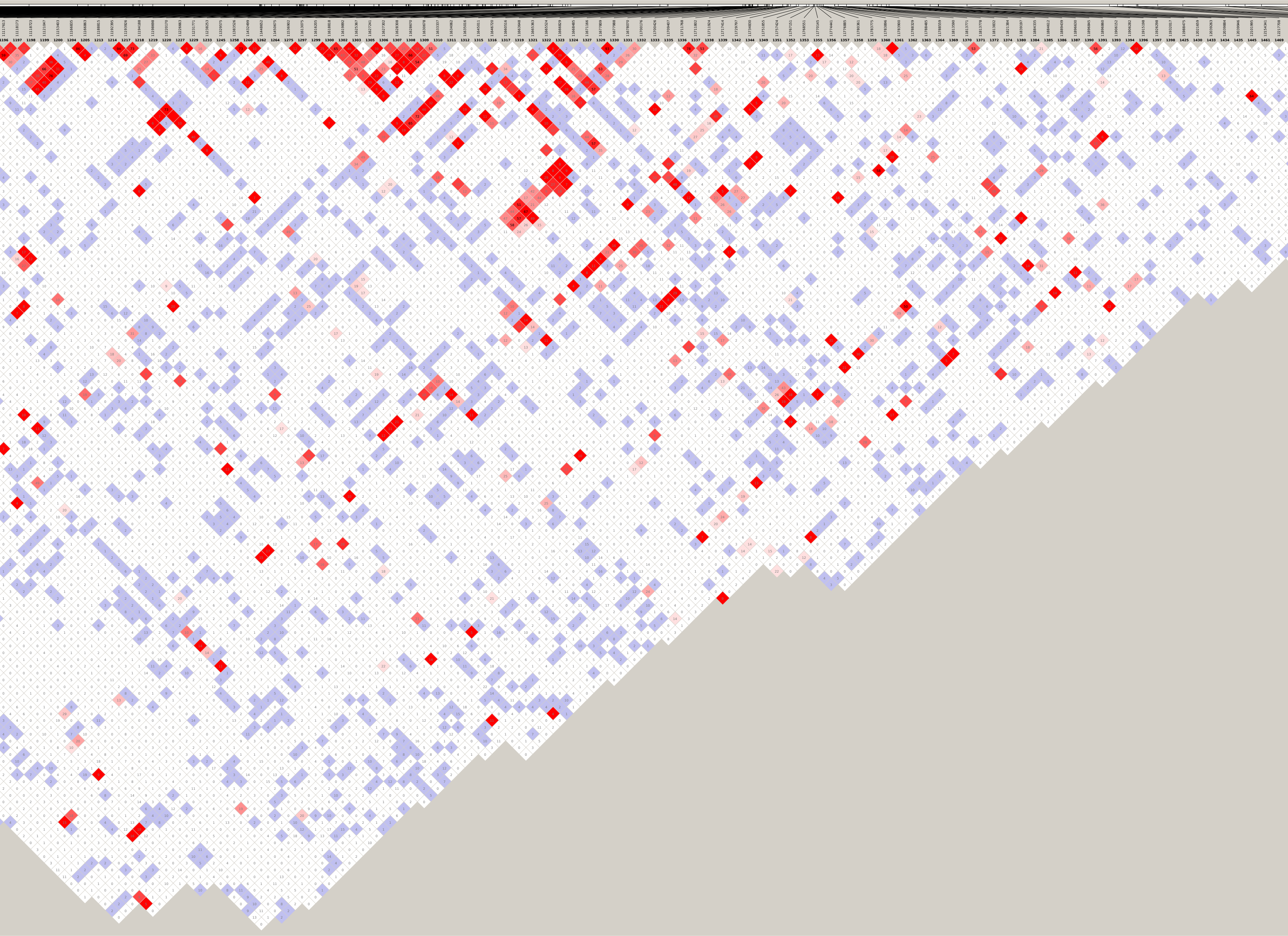

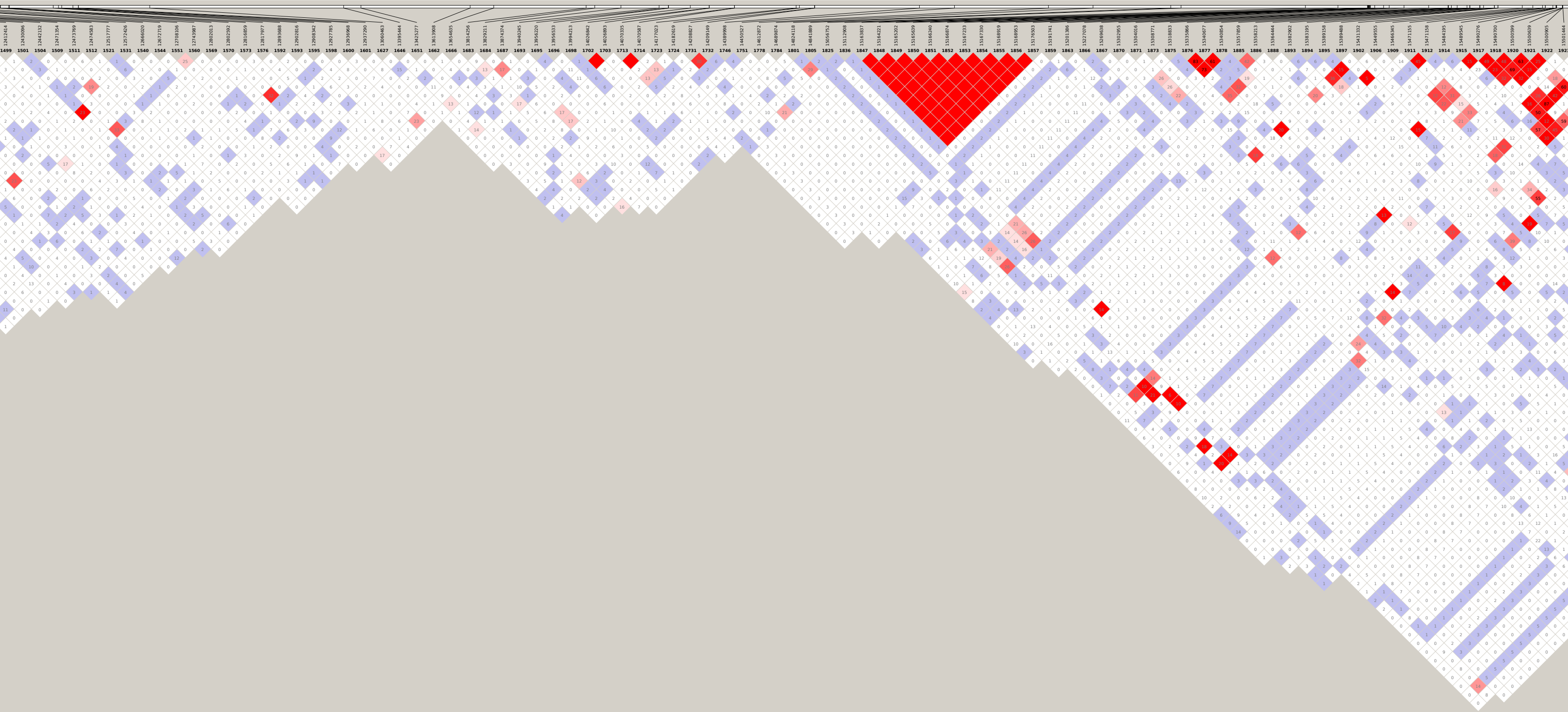

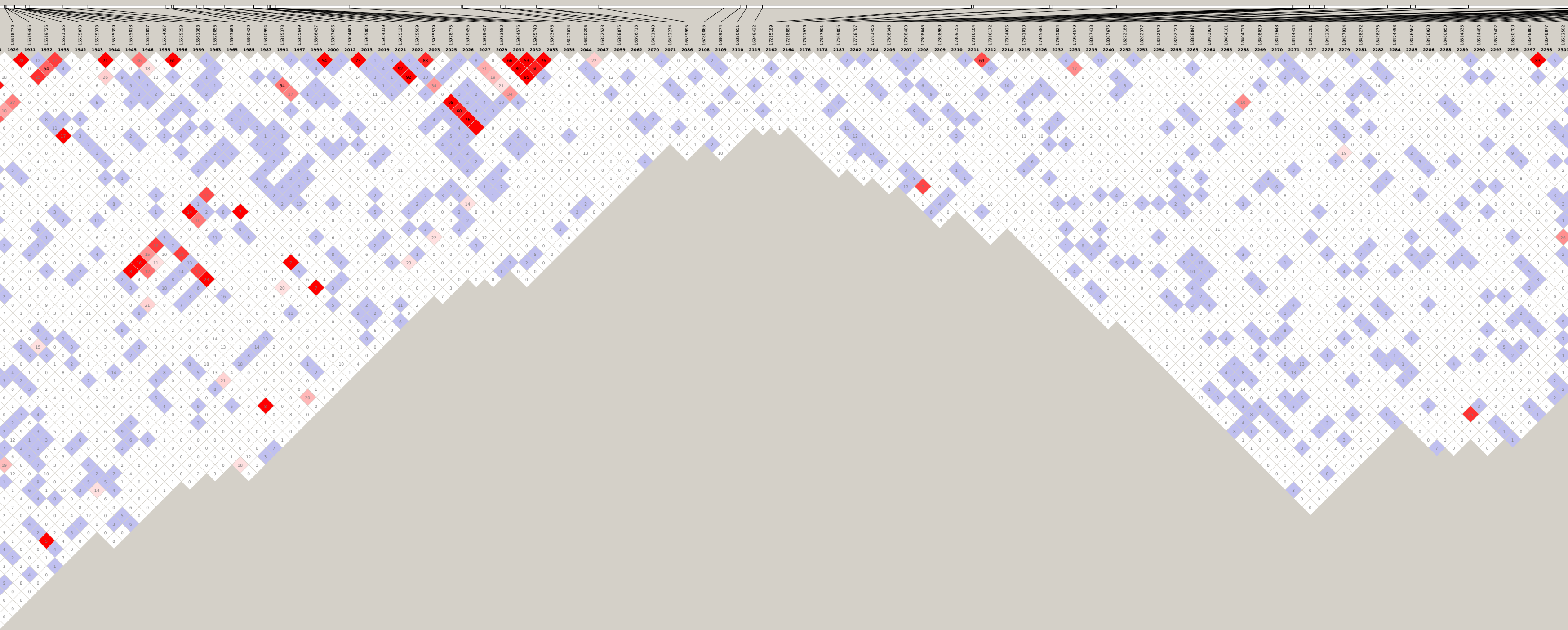

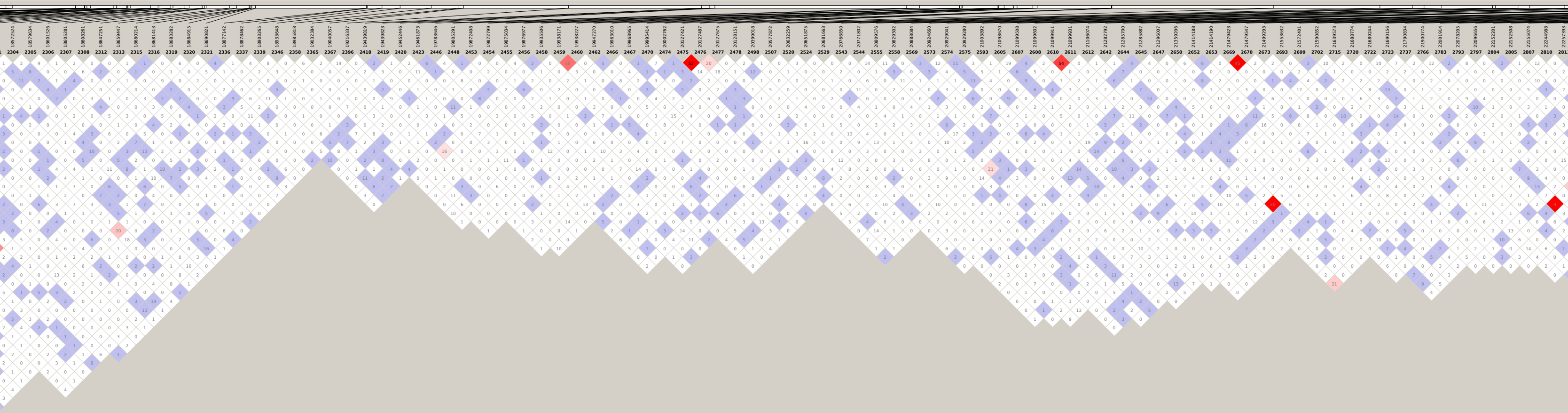

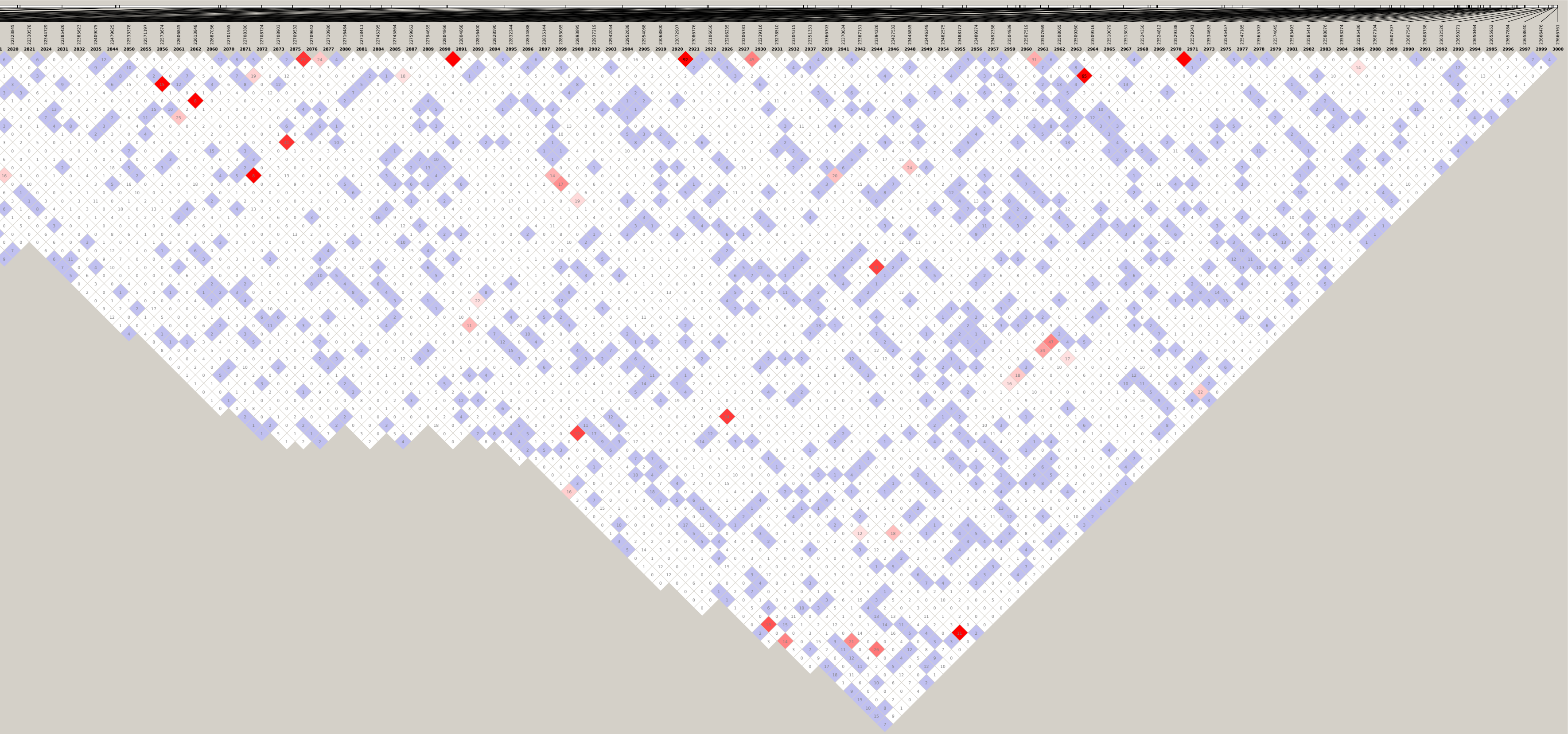

Supplement: Supplementary file 9 — Additional file 9: Figure S10. Haploview plots. Linkage disequilibrium (LD) plots generated with Haploview for each of the 24 medaka chromosomes, showing r2 values in the MIKK panel for each pair of SNPs within 1 Mb of one another. [file 13059_2022_2623_MOESM9_ESM.pdf]
