# Supplementary material for: The Medaka Inbred Kiyosu-Karlsruhe (MIKK) panel
Source: Genome Biol. 2022 Feb 21;23:59. doi: 10.1186/s13059-022-02623-z (PMC8862526; doi:10.1186/s13059-022-02623-z)
Supplement: Supplementary file 10 — Additional file 10. Review history. [file 13059_2022_2623_MOESM10_ESM.docx]

Review History

**First round of review**

**Reviewer 1**

**Are you able to assess all statistics in the manuscript, including the appropriateness of statistical tests used?**

No, I do not feel adequately qualified to assess the statistics.

**Comments to author:**

This study characterized population genomic features of the near-isogenic Medaka inbred Kiyosu-Karlsruhe panel with 80 inbred lines derived from a wild founder population, using WGS Illumina data. I see that this medaka MIKK panel would be broadly useful in genotype-phenotype mapping, GxE, sex determination, etc, according to convincing results presented. In general, the study is well designed and would have far-reaching implications for broader vertebrate genetics community to make use of the resources gathered in this study. Thus, I have only minor comments below.

1. I am wondering why the relative size of the eye and the relative distance between the eyes have been chosen to shown phetypic variation and their heritability.

2. "Overall, the between-line variance for these simple morphological measurements across the MIKK panel is high compared to the within-line variance, and broadly similar to human and mouse morphometric measurements, suggesting that these traits are heritable in medaka as expected."

It is difficult to understand "broadly similar to human and mouse morphometric measurements".

3. "a subset of lines that show a considerably lower degree of homozygosity"

It would be nice to justify this and to see chromosome-level or genome-wide pattern of homozygosity in those lines.

4. The potential functional impact of genomic variation has been compared to their human orthologs. I understand that the authors aim to use this medaka MIKK panel for human gene study. However, It would be interesting to know, for example, what genes with homozygous high-confidence LoF variations are and what GOs/pathways they are enriched.

5. In the genetic composition of the MIKK panel section, although the authors claim that very little allele skew genome-wide with no large regions showing high FST values, it seems that large genomic regions in chromosome 2 & 9 have large Fst. It is curious to know how Fig.6A would be if the authors removed genomic regions with log10(SNPs) < 1.5 in Fig.6B.

6. For whole genome sequencing, medaka organs were dissected from 6-month-old male adults. Fish were sacrificed by hypothermic shock. The brain was dissected and shock frozen in liquid nitrogen. For RNAseq analysis 12 month old adults that were kept at either 14 light:10 dark (summer condition) or 10 light:14 dark (winter condition) light cycles respectively were sacrificed by hypothermic shock andthe organs after dissection were shock frozen in liquid nitrogen.

Why were 12 month old female adults used in the eQTL study rather than those 6-month-old male adults used in WGS? Will this affect the eQTL analysis?

ENSORLT00000002786.2 is located in chromosome 18, so in the legend of Fig.10B, chr8 -> chr18?

Cis-eQTLs within 250 kb ... the genotypes of every SNPs within 500 kb upstream and downstream, 250 or 500 kb? 500kb seems correct according to Fig.10C.

telost -> teleost

When the authors are trying to tell where the MIKK was set up and kept, abbreviations come from nowhere, e.g., KIT. I have to google to know IBCS-BIP belongs to KIT.

**Reviewer 2**

**Are you able to assess all statistics in the manuscript, including the appropriateness of statistical tests used?**

Yes, and I have assessed the statistics in my report.

**Comments to author:**

As a resource paper, authors generated an inbreeding population in medaka, i.e., Medaka Inbred Kiyosu-Karlsruhe (MIKK) Panel. They first documented the phenotypic variation of MIKK (Fig. 1). Then they generated DNA resequencing data across lines and population transcriptome data of liver. Later on, they carried out numerous analyses with respect to residue heterozygosity (Fig. 2/3), loss of function (LoF) mutations (Fig. 4), population structure (Fig. 5), phylogenetic relationship relative to other medaka strains (Fig. 6/7), linkage disequilibrium (LD, Fig. 8), copy number variants (CNV, Fig. 9) and expression QTL (eQTL, Fig. 10).

Overall, I believe that MIKK could be useful: although there are many population data in human (e.g. 1000 GP), mouse (CC) and fly (DGRP), it is good to have MIKK especially considering that these inbreeding lines better mimic the outcross status of humans. However, this version of manuscript is problematic in terms of presentation and methodological stringency. I am thus making the following specific comments with the hope to help authors to better write a resource-type paper.

Major concerns:

1. The uniqueness or the value of MIKK should be better described.

a. Authors argued for the value of MIKK by stating that mouse populations could not approximate the out-crossed humans. However, medaka is really deeply diverged from humans compared to mouse. Authors should acknowledge this limit in Discussion.

b. Analogously, due to teleost specific whole genome duplication, I am not sure how many genes could reach the clear orthology mapping between human and medaka, while authors extensively used human orthologs in their analyses. Thus, authors should explain how to define the orthologly in Methods, especially how many genes could form one-to-one mapping. If the number is very small, they better use term "homolog" rather than "ortholog".

c. Authors tried to argue the significance of this resource with the implication of human diseases. However, as aforementioned, this link could be weak. So could authors better elaborate on how their study shed light on human disease research? Alternatively, I suggest authors to argue the significance of MIKK for fish biology (e.g., conservation or domestication).

2. Population resource is often used for association studies (GWAS) and thus the diversity level of phenotypes and genotypes is critical. Authors should better demonstrate these features.

a) Only Fig. 1b shows distribution of one phenotype (fecundity). I wonder whether authors already have some other phenotypic data to show that this population really has a rich phenotypic variance. If yes, such data should be put into Fig. 1.

b) Authors should calculate the conventionally used parameter (nucleotide diversity or Pi) to summarize the genetic diversity. For this analyses, authors could do the sliding window analyses within each chromosome. The sex determination locus in Chr1 should show higher Pi. Moreover, I wonder how high the Pi is for the natural population of medaka. If MIKK's genome-wide median Pi is similar to natural medaka, it would strongly argue that MIKK has a high level of diversity.

c) I suggest authors to add a new Figure to summarize their sequencing data e.g. the Q30 read count, depth and mappability of re-sequencing data and transcriptome data of liver.

3. Authors perform lots of analyses and generated nine figures. However, many of them are simply descriptive, difficult to interpret or irrelevant for the demonstration of the value of MIKK. Many figures share the same three types of problems. So, I just gave one or two examples for each problem.

a) For Fig. 9, authors said "The majority of common CNVs do not fall in regions of high dosage intolerance for human orthologous genes across the medaka genome (Figure 9A), and as expected higher frequency CNVs tend to have lower pLI LOD scores (Figure 9C). CNV events with very large pLI LOD scores (> 20) are observed exclusively as either 1 or 3 copy events (Figure 9B), with those with more extreme copy numbers (zero or greater than 3 copies) showing a clear decrease in the number of positive pLI LOD scores (Figure 9B)." Authors made many statements here. However, the corresponding figures are just scatter or box plots without any statistical tests. For example, I do not understand why Fig. 9A could show "The majority of common CNVs do not fall in regions of high dosage intolerance for human orthologous genes across the medaka genome" or Fig. 9C could support "higher frequency CNVs tend to have lower pLI LOD scores". Even for some figures, I can guess that there are some patterns there as Authors described. However, are these patterns statistically significant? Such a descriptive tone is present across the analyses and figures.

b) For Fig. 2, chr1 shows the highest level of heterozygosity followed by chr3 and chr2. Authors explained chr1 (sex chromosome) and chr2 (high recombination). But how about chr3? In case of Fig. 4, chr2/chr18 are enriched with multiple types of LoF mutations. Why should be so? Does it mean that these two chromosomes encode less important genes? That seems less likely.

c) For a population resource, Fig. 2, 3, 5, 8 and 10 seems to be relevant for the downstream studies (e.g., GWAS). I suggest to move the other figures to the supplemental figs or better explain why they are important for this resource. If authors prefer to keep these figures, they have to better present and explain them, which mean more efforts.

4. Related with the above comments, authors' analyses in Fig. 9 are unconventional and thus it is difficult for readers to evaluate how the calling quality is. That is, there are many published (even independently evaluated) softwares (LUMPY, Genome STRiP, CNVnnator, etc) designed to call CNVs. Authors designed a new software, but did not perform any validation. Thus, how reliable data in Fig. 9 is remains uncertain. Authors at least need to explain why they believed their method outperformed the preexisting ones. Alternatively, as I suggested above, this whole section could be removed.

5. How authors analyzed heterozygosity is also unconventional. Presumably, authors can simply tell the readers how many SNPs are homozygous as the previous practices (eg. DGRP) did. However, authors used "heterozygous blocks", which is not easy to understand. Authors further stated that "Over 70% of MIKK panel lines are homozygous for greater than 70% of their entire genome, which is equivalent to the similar inbred Drosophila resources (5)." Ref 5 refers to DGRP, whose heterozygosity level is evaluated in Huang et al Genome Res. 2014 paper. It stated "Approximately 96% of the lines had 2% or fewer segregating X-linked variants, while on average 84% of the lines had 2% or fewer segregating autosomal variants". Thus, it seems that MIKK has much higher heterozygosity level, which may bring difficulty for downstream analyses (e.g, GWAS). If yes, I suggest authors to discuss this caveat.

6. Related with the above comment, most residue heterozygosity of DGRP are caused by big chromosomal inversions, which could not be removed by inbreeding. Could authors identify inversions in MIKK?

7. Sex determination or sex chromosome of medaka should be explained in the Introduction. Fig. 3B seems to suggest that recombination suppression occurs for almost half chromosome (potentially the first evolutionary strata). Authors could cite some classical paper on sex chromosome evolution (eg. those by Doris Bachtrog) to provide readers with a context.

8. For dosage sensitive genes, authors used human pLI to approximate. Again, due to the divergence between human and medaka, whether this assumption holds is somewhat uncertain. So, I suggest authors to use genes with zebrafish ortholog annotated in protein complex as dosage sensitive genes in their analysis, since protein complex members are well known to be dosage sensitive.

Minor concerns:

The presentation is sloppy. I just give a few examples here.

1. Multiple figures (e.g. Figs. 4, 5) lack high resolution version, either in the merged pdf file or in the online file.

2. The figure is not well ordered. For example, figure 9c occurs before figure 9b.

3. For Fig. 1A, the color code for infertile male and "No males" are largely not distinguishable.

4. "However, 63% had confident homozygous calls, with 34% of LoF SNPs showing no evidence of heterozygosity in any of the individual lines (Figure 4F)". I do not understand why Fig. 4F is used in this context or maybe because this panel is too blur to be understood.

5. "Segregation analysis of trios revealed advantageous linkage disequilibrium (LD) properties". What does "advantageous" mean here?

**Authors Response**

**Point-by-point responses to the reviewers’ comments:**

Reviewer #1:

This study characterized population genomic features of the near-isogenic Medaka inbred Kiyosu-Karlsruhe panel with 80 inbred lines derived from a wild founder population, using WGS Illumina data. I see that this medaka MIKK panel would be broadly useful in genotype-phenotype mapping, GxE, sex determination, etc, according to convincing results presented. In general, the study is well designed and would have far-reaching implications for broader vertebrate genetics community to make use of the resources gathered in this study. Thus, I have only minor comments below.

1. I am wondering why the relative size of the eye and the relative distance between the eyes have been chosen to shown phetypic variation and their heritability.

*Response: Previous publications and our own work show a significant genetic contribution to craniofacial variance in established isogenic inbred medaka strains, including relative eye diameter and distance between the eyes (Kimura et al., 2007, Spivakov et al., 2014). To analyze whether MIKK lines show line-specific craniofacial variance we chose these two parameters as they are conspicuous and therefore permit reliable quantification for the required statistical analysis of broad sense heritability.*

*In the revised version we have changed the respective section accordingly and included this information (Results: Fecundity and morphological measures across the MIKK Panel/ second paragraph).*

2. "Overall, the between-line variance for these simple morphological measurements across the MIKK panel is high compared to the within-line variance, and broadly similar to human and mouse morphometric measurements, suggesting that these traits are heritable in medaka as expected."

It is difficult to understand "broadly similar to human and mouse morphometric measurements”.

*Response: This was indeed a confusing statement and we have updated the sentence and added some references to make it clearer that we are talking about “broadly similar heritability estimates to human and mouse for these morphometric measurements”.(Results: Fecundity and morphological measures across the MIKK Panel/ last paragraph).*

3. "a subset of lines that show a considerably lower degree of homozygosity"

It would be nice to justify this and to see chromosome-level or genome-wide pattern of homozygosity in those lines.

*Response: We hypothesise that the lower degree of homozygosity in a small subset of lines (i.e. 5 lines that show < 50% homozygosity) is due to an accidental outcross with distantly related MIKK fish during the inbreeding crosses, leading to a significant drop in the homozygosity. To test our hypothesis we are currently conducting additional inbreeding crosses with these lines with the aim to examine whether these crosses will increase the lower homozygosity to levels corresponding with the average MIKK inbred lines.*

*We have added this information in the revised version (Results: Homozygosity of the MIKK panel genomes, second paragraph).*

4. The potential functional impact of genomic variation has been compared to their human orthologs. I understand that the authors aim to use this medaka MIKK panel for human gene study. However, It would be interesting to know, for example, what genes with homozygous high-confidence LoF variations are and what GOs/pathways they are enriched.

*Response: These genes are listed in the additional data file 4 - table 5 and we carried out a basic GO analysis across these 614 unique medaka genes. We have added a short description of the top molecular function, biological process and pathways at the end of this section “Potential functional impact of small genomic variation”.*

5. In the genetic composition of the MIKK panel section, although the authors claim that very little allele skew genome-wide with no large regions showing high FST values, it seems that large genomic regions in chromosome 2 & 9 have large Fst. It is curious to know how Fig.6A would be if the authors removed genomic regions with log10(SNPs) < 1.5 in Fig.6B.

*Response: This was an interesting point and we have updated the section, including a supplementary figure showing the genome wide Fst values for regions with at least 50 SNP (log10(SNP) > 1.69). As expected the removal of sites with low numbers of SNPs has resulted in a substantial decrease in the number of high Fst regions and the regions mentioned on both chromosome 2 and 9 with higher Fst have largely disappeared.*

6. For whole genome sequencing, medaka organs were dissected from 6-month-old male adults. Fish were sacrificed by hypothermic shock. The brain was dissected and shock frozen in liquid nitrogen. For RNAseq analysis 12 month old adults that were kept at either 14 light:10 dark (summer condition) or 10 light:14 dark (winter condition) light cycles respectively were sacrificed by hypothermic shock andthe organs after dissection were shock frozen in liquid nitrogen.

Why were 12 month old female adults used in the eQTL study rather than those 6-month-old male adults used in WGS? Will this affect the eQTL analysis?

*Response: For whole genome sequencing the brain of 6 month old medaka males was used as donor tissue. The aim was to sequence both sex chromosomes (i.e. X-chromosome and male specific Y-chromosome; these are the two variants of chromosome 1) therefore brains of males were used as they have the XY genomic configuration. The age of the medaka adult has a negligible effect on the genome. For logistical reasons we used 6 month old male adults to carry out the whole genome sequencing. Therefore, since the genomic sequence is unaffected by the donor age, the eQTL analysis of sexually mature 12 months old MIKK fish is not affected by this age difference.*

*For the eQTL analysis sexually mature adults were analysed to include mature gonads of sexually active adults in the expression analysis (data not shown). MIKK adults reach sexual maturity after about 6 month. For the RNAseq analysis we dissected tissues of both males and females under summer"(long photoperiod 14/10LD) and winter (short photoperiod 10/14 LD) conditions. As the standard condition in the medaka facility at KIT is set to 14/10 LD, MIKK fish had therefore to be acclimatized for the short photoperiod for at least 1 month. Therefore to ensure sexual maturity for all donor fish and full acclimatization, 12 months old MIKK fish were chosen for the RNA seq analysis.*

*We have added this information in the revised version (Results: Homozygosity of the MIKK panel genomes, first paragraph; Material & Methods: Tissue dissection)*

ENSORLT00000002786.2 is located in chromosome 18, so in the legend of Fig.10B, chr8 -> chr18?

*Response: This has been corrected.*

Cis-eQTLs within 250 kb ... the genotypes of every SNPs within 500 kb upstream and downstream, 250 or 500 kb? 500kb seems correct according to Fig.10C.

*Response: This has been corrected.*

telost -> teleost

*Response: This has been corrected.*

*We would like to apologize for this spelling error. We have thoroughly edited the entire revised manuscript to prevent further errors.*

When the authors are trying to tell where the MIKK was set up and kept, abbreviations come from nowhere, e.g., KIT. I have to google to know IBCS-BIP belongs to KIT.

*Response: We apologize for this lack of providing important information. In the revised version we have included the full name: Karlsruhe Institute of Technology (KIT). The respective first paragraph of the Materials and methods section has now been changed to:*

*Medaka (Oryzias latipes) fish were maintained at the medaka facility of the Institute of Biological and Chemical Systems, Biological Information Processing (IBCS-BIP) at the Karlsruhe Institute of Technology (KIT); Germany. All animal husbandry and experimental procedures were carried out in accordance with local and European Union animal welfare standards (Tierschutzgesetz §11, Abs. 1, Nr. 1, AZ35-9185.64/BH). The facility is under the supervision of the local representative of the animal welfare agency.*

Reviewer #2:

As a resource paper, authors generated an inbreeding population in medaka, i.e., Medaka Inbred Kiyosu-Karlsruhe (MIKK) Panel. They first documented the phenotypic variation of MIKK (Fig. 1). Then they generated DNA resequencing data across lines and population transcriptome data of liver. Later on, they carried out numerous analyses with respect to residue heterozygosity (Fig. 2/3), loss of function (LoF) mutations (Fig. 4), population structure (Fig. 5), phylogenetic relationship relative to other medaka strains (Fig. 6/7), linkage disequilibrium (LD, Fig. 8), copy number variants (CNV, Fig. 9) and expression QTL (eQTL, Fig. 10).

Overall, I believe that MIKK could be useful: although there are many population data in human (e.g. 1000 GP), mouse (CC) and fly (DGRP), it is good to have MIKK especially considering that these inbreeding lines better mimic the outcross status of humans. However, this version of manuscript is problematic in terms of presentation and methodological stringency. I am thus making the following specific comments with the hope to help authors to better write a resource-type paper.

Major concerns:

1. The uniqueness or the value of MIKK should be better described.

a. Authors argued for the value of MIKK by stating that mouse populations could not approximate the out-crossed humans. However, medaka is really deeply diverged from humans compared to mouse. Authors should acknowledge this limit in Discussion.

*Response: The reviewer is right, the divergence of medaka to humans is considerably larger than that of mouse and humans. We have added a paragraph in the Discussion section where we discuss this and options to improve the bridging of medaka and mammalian genomes However, we see the uniqueness and advantage of the MIKK panel not only in potential biomedical modelling applications but also for basic research, to develop tools for GWAS and testing of basic hypothesis. The MIKK panel provides considerable advantages, including economical husbandry and the ease with which wild fish can be obtained and phenotyped (including behavioural tests). Thus a main advantage of medaka is the option to test hypotheses obtained from laboratory animals on wild individuals of the same species.*

*We have changed the Discussion section in the revised version accordingly (Discussion: last two paragraphs).*

b. Analogously, due to teleost specific whole genome duplication, I am not sure how many genes could reach the clear orthology mapping between human and medaka, while authors extensively used human orthologs in their analyses. Thus, authors should explain how to define the orthologly in Methods, especially how many genes could form one-to-one mapping. If the number is very small, they better use term "homolog" rather than “ortholog".

*Response: We apologize for this omission - we have now added a methods section describing the orthology mapping to human orthologs using the PANTHER resource. Overall we were able to map a total of 12,952 medaka genes to a human ortholog, over 90% (11,678) of which had a direct one-to-one mapping.*

*The larger divergence of medaka to humans can at least in part be overcome as shown in a recent publication, where spotted gar (lepisosteus oculatus) served as a bridge to improve orthology relations between humans and medeka. This helped to overcome problems imposed by the additional genome duplication of teleosts. We have added a section and citation to this work in the discussion section (Discussion, last two paragraphs).*

c. Authors tried to argue the significance of this resource with the implication of human diseases. However, as aforementioned, this link could be weak. So could authors better elaborate on how their study shed light on human disease research? Alternatively, I suggest authors to argue the significance of MIKK for fish biology (e.g., conservation or domestication).

*Response: As outlined above (see point 1a) we see an important advantage of the MIKK panel for basic research, as a model to develop tools for association studies in vertebrates. Furthermore the option to sample from the wild will allow us to test hypotheses obtained from GWAS of the laboratory lines in wild animals. This will prove to be important for addressing questions related to ecology.*

*We have added these arguments in the revised version in the Discussion section, last paragraph and also provided further information into the medaka gene to human mapping performed in this study as well as a discussion into how this might be improved in the future.*

2. Population resource is often used for association studies (GWAS) and thus the diversity level of phenotypes and genotypes is critical. Authors should better demonstrate these features.

a) Only Fig. 1b shows distribution of one phenotype (fecundity). I wonder whether authors already have some other phenotypic data to show that this population really has a rich phenotypic variance. If yes, such data should be put into Fig. 1.

*Response: We have added an additional phenotype into Figure 1 showing the distribution in relative size of the eye (relative to the total body size) for male and female medaka fish across the MIKK panel lines (see also our answer to point 1 of reviewer #1).*

b) Authors should calculate the conventionally used parameter (nucleotide diversity or Pi) to summarize the genetic diversity. For this analyses, authors could do the sliding window analyses within each chromosome. The sex determination locus in Chr1 should show higher Pi. Moreover, I wonder how high the Pi is for the natural population of medaka. If MIKK's genome-wide median Pi is similar to natural medaka, it would strongly argue that MIKK has a high level of diversity.

*Response: We thank the reviewer for this valuable suggestion and have added a section looking at the nucleotide diversity measured by Pi in the “Homozygosity of the MIKK panel genomes” results section. Overall, we observe similar levels of Pi between the MIKK panel genomes and wild medaka showing that MIKK contains a good representation of the wild variation and we observe the expected increase in Pi around the sex determination region as well as in regions containing more repetitive sequences.*

c) I suggest authors to add a new Figure to summarize their sequencing data e.g. the Q30 read count, depth and mappability of re-sequencing data and transcriptome data of liver.

*Response: The read count, depth and mappabiliy of resequencing nd transcripme data of the liver and additional QC reports can be found at the following repository - https://github.com/birneylab/MIKK_genome_main_paper/tree/master/eQTL*

3. Authors perform lots of analyses and generated nine figures. However, many of them are simply descriptive, difficult to interpret or irrelevant for the demonstration of the value of MIKK. Many figures share the same three types of problems. So, I just gave one or two examples for each problem.

a) For Fig. 9, authors said "The majority of common CNVs do not fall in regions of high dosage intolerance for human orthologous genes across the medaka genome (Figure 9A), and as expected higher frequency CNVs tend to have lower pLI LOD scores (Figure 9C). CNV events with very large pLI LOD scores (> 20) are observed exclusively as either 1 or 3 copy events (Figure 9B), with those with more extreme copy numbers (zero or greater than 3 copies) showing a clear decrease in the number of positive pLI LOD scores (Figure 9B)." Authors made many statements here. However, the corresponding figures are just scatter or box plots without any statistical tests. For example, I do not understand why Fig. 9A could show "The majority of common CNVs do not fall in regions of high dosage intolerance for human orthologous genes across the medaka genome" or Fig. 9C could support "higher frequency CNVs tend to have lower pLI LOD scores". Even for some figures, I can guess that there are some

patterns there as Authors described. However, are these patterns statistically significant? Such a descriptive tone is present across the analyses and figures.

*Response: We agree with the reviewer and on reflection realise that we included too many figures some of which are meaningful to us but not so well described and challenging to interpret for the reader. Therefore, we have taken this reviewer's advice and moved a number of figures to supplementary information and also aimed to better explain certain analysis (see below).*

b) For Fig. 2, chr1 shows the highest level of heterozygosity followed by chr3 and chr2. Authors explained chr1 (sex chromosome) and chr2 (high recombination). But how about chr3? In case of Fig. 4, chr2/chr18 are enriched with multiple types of LoF mutations. Why should be so? Does it mean that these two chromosomes encode less important genes? That seems less likely.

*Response: The reviewer makes an interesting observation about chromosome 3 - we have checked certain characteristic of chromosome 3 in detail, for example repeat content, relative number of coding genes and potential divergence from the reference genome however we have been unable to find any clear indication as to why it displays a lower level of homozygosity than most other chromosomes.*

*For the enrichment in LoF variation in chr2 and 18, we do not believe that chr2 and 18 encode less important genes however both chromosomes are enriched for repeat content and for inversions (Additional File 5 Figure 3 and 4) and we expect that the apparent LoF enrichment is a result of both complex repetitive genome content and a potentially higher mutation rate for these chromosomes.*

c) For a population resource, Fig. 2, 3, 5, 8 and 10 seems to be relevant for the downstream studies (e.g., GWAS). I suggest to move the other figures to the supplemental figs or better explain why they are important for this resource. If authors prefer to keep these figures, they have to better present and explain them, which mean more efforts.

*Response: We have taken this reviewer's advice and moved the relevant figures to Supplementary with the exception of Figure1 for which we added some additional phenotypic information and better described the content.*

4. Related with the above comments, authors' analyses in Fig. 9 are unconventional and thus it is difficult for readers to evaluate how the calling quality is. That is, there are many published (even independently evaluated) softwares (LUMPY, Genome STRiP, CNVnnator, etc) designed to call CNVs. Authors designed a new software, but did not perform any validation. Thus, how reliable data in Fig. 9 is remains uncertain. Authors at least need to explain why they believed their method outperformed the preexisting ones. Alternatively, as I suggested above, this whole section could be removed.

*Response: We thank the reviewer for the advice concerning these sections; we also agree that the analysis of CNV in the MIKK panel is somewhat incomplete and non essential for this manuscript. Therefore, we have completely removed the CNV analysis from the paper and added a small section in the companion paper (which is much more focussed on SVs from ONT data) comparing SV detection between linear and graph based approaches.*

5. How authors analyzed heterozygosity is also unconventional. Presumably, authors can simply tell the readers how many SNPs are homozygous as the previous practices (eg. DGRP) did. However, authors used "heterozygous blocks", which is not easy to understand. Authors further stated that "Over 70% of MIKK panel lines are homozygous for greater than 70% of their entire genome, which is equivalent to the similar inbred Drosophila resources (5)." Ref 5 refers to DGRP, whose heterozygosity level is evaluated in Huang et al Genome Res. 2014 paper. It stated "Approximately 96% of the lines had 2% or fewer segregating X-linked variants, while on average 84% of the lines had 2% or fewer segregating autosomal variants". Thus, it seems that MIKK has much higher heterozygosity level, which may bring difficulty for downstream analyses (e.g, GWAS). If yes, I suggest authors to discuss this caveat.

*Response: We have added into this section the total proportion of homozygous called variants across all MIKK panel lines showing that by far the majority of variants are indeed homozygous (only 6% of all SNP genotypes across the MIKK panel are called as heterozygous). We have also included an analysis of the nucleotide diversity measure and retained the analysis of homozygous blocks as it allows us to assess the distribution of residual heterozygosity across all panel members genome wide.*

*We apologize for the statement “equivalent to the similar inbred Drosophila resource” - this has been removed in the revised version.*

6. Related with the above comment, most residue heterozygosity of DGRP are caused by big chromosomal inversions, which could not be removed by inbreeding. Could authors identify inversions in MIKK?

*Response: We investigated SV content - including inversions – using ONT data from the companion paper (Additional File 5 Figure 3), and observed large (>250 kb) inversions and other structural variants on specific regions of chr 2, 11 and 18, which correspond closely with the increased level of heterozygosity also observed in those regions.*

7. Sex determination or sex chromosome of medaka should be explained in the Introduction. Fig. 3B seems to suggest that recombination suppression occurs for almost half chromosome (potentially the first evolutionary strata). Authors could cite some classical paper on sex chromosome evolution (eg. those by Doris Bachtrog) to provide readers with a context.

*Response: Following the reviewers suggestion, we have added a paragraph in the Introduction section on sex determination and sex chromosomes in medaka and related Oryzias species, where we also cite a key review on Y chromosome evolution by Doris Bachtrog.*

8. For dosage sensitive genes, authors used human pLI to approximate. Again, due to the divergence between human and medaka, whether this assumption holds is somewhat uncertain. So, I suggest authors to use genes with zebrafish ortholog annotated in protein complex as dosage sensitive genes in their analysis, since protein complex members are well known to be dosage sensitive.

*Response: The relationship between medaka and zebrafish gene orthologs will undoubtedly be one important source of evidence when mapping genes to function during subsequent research using the MIKK panel.*

*However, given the reasonable mapping of medaka to human orthologs for approximately half of coding genes to medaka and the extensive work into dosage intolerance predictions from large human cohorts (pLI score) we think that the inclusion of pLI scores mapped to medaka genes is of general interest. For example, medaka genes with a direct one-to-one mapping to a human ortholog with high pLI scores and relatively large numbers of loss of function variation in the MIKK panel may help to prioritise gene candidates during e.g gene mutagenesis experiments. We have also added more discussion around caveats and potential improvements during medaka to human orthology mapping.*

*It is worth noting that there are not as deep “fully wild” resources on Zebrafish, so an equivalent pLi score is not available (to our knowledge). We will certainly maintain links to Zebrafish resources and genetics, and expect to use Zebrafish phenotypes in later comparisons. We have added to the discussion on this effect.*

Minor concerns:

The presentation is sloppy. I just give a few examples here.

1. Multiple figures (e.g. Figs. 4, 5) lack high resolution version, either in the merged pdf file or in the online file.

*Response: Please accept our apologies for the lack of resolution in the mentioned figures - this has been resolved in the revised submission.*

2. The figure is not well ordered. For example, figure 9c occurs before figure 9b.

*Response: We have reviewed the order of all figures and ensured that the labelling is consistent with the first reference within the text.*

3. For Fig. 1A, the color code for infertile male and "No males" are largely not distinguishable.

*Response: This has been correct in the revised version.*

4. "However, 63% had confident homozygous calls, with 34% of LoF SNPs showing no evidence of heterozygosity in any of the individual lines (Figure 4F)". I do not understand why Fig. 4F is used in this context or maybe because this panel is too blur to be understood.

*Response: This was indeed an incorrect reference to the figure and has been removed.*

5. "Segregation analysis of trios revealed advantageous linkage disequilibrium (LD) properties". What does "advantageous" mean here?

*Response: In this statement we were referring to the relatively short blocks and sharp LD decay observed in medaka - we have added a clarifying statement within the sentence.*

**Second round of review**

**Reviewer 1**

The authors have improved the MS in this revised version, and addressed my concerns of the previous version.

**Reviewer 2**

As I commented previously, the previous version is simply too descriptive, difficult to interpret or irrelevant for the demonstration of the value of MIKK. Authors clearly tried to revise. However, they only stated which part of the manuscript was revised for some comments. Thus, I have to use “search” to localize how the manuscript was actually revised, which made me spend more time. But anyway, I believe that the new version is better. However, the manuscript is still somewhat sloppy in terms of presentation or logical flow. So, I am making the following comments to help further improve.

Major concerns:

1. “we observe the expected increase in Pi around the sex determination region as well as in regions containing more repetitive sequences. “This response corresponds to Page 11: “The higher level of 𝜋̂ observed within specific regions on chromosomes 2, 11, and 18 correspond closely to the regions we identified as containing large (>250 kb) inversions that appear to be shared across at least some of the MIKK panel (Additional File 5 Figure 3). These regions are also enriched for large deletions and duplications (44). The high amounts of repeat content in these regions (Additional File 5 Figure 4) may explain this enrichment of large structural variation, which in turn could hinder recombination, resulting in the observed depressions in homozygosity at these loci”. As I commented previously, inversions are well-known to cause permanent heterozygosity and thus increase Pi. However, why repetitive regions should show high Pi is difficult to understand. Usually, these regions are excluded from analyses due to multi-mapping issue and thus these regions would appear to be “homozygous”. Moreover, repeat is known to facilitate recombination and thus lead to copy number gain or loss. Did authors mean that duplications or deletions would be associated with more SNPs? Anyway, this section needs to be clarified.

2. “The reviewer makes an interesting observation about chromosome 3 - we have checked certain characteristic of chromosome 3 in detail, for example repeat content, relative number of coding genes and potential divergence from the reference genome however we have been unable to find any clear indication as to why it displays a lower level of homozygosity than most other chromosomes. “ I searched and found in Page 10: “In addition, chromosomes 2 and 3 showed higher levels of heterozygosity (see discussion) compared to the other chromosomes.”. I then searched the Discussion section for several times but couldn't find where authors discussed about this issue. Actually, if authors do not know why chromosome 3 behaves like this, they can simply acknowledge this limit in Discussion.

3. “For the enrichment in LoF variation in chr2 and 18, we do not believe that chr2 and 18 encode less important genes however both chromosomes are enriched for repeat content and for inversions (Additional File 5 Figure 3 and 4) and we expect that the apparent LoF enrichment is a result of both complex repetitive genome content and a potentially higher mutation rate for these chromosomes.” The revision corresponds to “interestingly both chromosomes 2 and 18 show consistently higher numbers of LoF variation for all LoF variant classes (Additional File 5 Figure 5 A-D) (see discussion).” Again, there is no relevant content in Discussion. Since the same mistake occurred twice, I sort of wonder whether the authors uploaded a wrong version of manuscript. For the argument per se, repetitive regions lead to missing data due to the aforementioned multi-mapping issue. Similarly, the enrichment of LoF in chr2 or chr18 should be unrelated with mutation rate. The most likely scenario is that these LoFs are recessive due to inversions, i.e., they are shielded by permanent heterozygosity.

4. “We investigated SV content - including inversions – using ONT data from the companion paper (Additional File 5 Figure 3), and observed large (>250 kb) inversions and other structural variants on specific regions of chr 2, 11 and 18, which correspond closely with the increased level of heterozygosity also observed in those regions.” How about chr3? Did this chromosome also encode inversions? 5. Following the reviewers suggestion, we have added a paragraph in the Introduction section on sex determination and sex chromosomes in medaka and related Oryzias species, where we also cite a key review on Y chromosome evolution by Doris Bachtrog. When describing Fig. 3, I suggest to recite this paper and state that the region with high heterozygosity could be the first evolutionary strata. In this way, readers can better understand this phenomenon. Minor concern: 1. “Please accept our apologies for the lack of resolution in the mentioned figures - this has been resolved in the revised submission.” Yes, the new figures indeed have higher resolution. However, I noticed another issue that font sizes for X- and Y-axis labels and legends are very small for almost all figures. In my computer screen, I need to enlarge several times to recognize them.

**Authors Response**

**Point-by-point responses to the reviewers’ comments:**

Reviewer #1:

The authors have improved the MS in this revised version, and addressed my concerns of the previous version.

*Response: We thank the reviewer for the positive evaluation of our previous revision.*

Reviewer #2:

As I commented previously, the previous version is simply too descriptive, difficult to interpret or irrelevant for the demonstration of the value of MIKK. Authors clearly tried to revise. However, they only stated which part of the manuscript was revised for some comments. Thus, I have to use “search” to localize how the manuscript was actually revised, which made me spend more time. But anyway, I believe that the new version is better. However, the manuscript is still somewhat sloppy in terms of presentation or logical flow. So, I am making the following comments to help further improve.

*Response: We apologize for the problems locating our revisions and would like to point out that we were asked by the editors to omit line numbers in the revised version. We have tried to give precise information on where we have made changes to the manuscript in this second revision.*

Major concerns:

1. “we observe the expected increase in Pi around the sex determination region as well as in regions containing more repetitive sequences. “This response corresponds to Page 11: “The higher level of ̂ observed within specific regions on chromosomes 2, 11, and 18 correspond closely to the regions we identified as containing large (>250 kb) inversions that appear to be shared across at least some of the MIKK panel (Additional File 5 Figure 3). These regions are also enriched for large deletions and duplications (44). The high amounts of repeat content in these regions (Additional File 5 Figure 4) may explain this enrichment of large structural variation, which in turn could hinder recombination, resulting in the observed depressions in homozygosity at these loci”.

As I commented previously, inversions are well-known to cause permanent heterozygosity and thus increase Pi. However, why repetitive regions should show high Pi is difficult to understand. Usually, these regions are excluded from analyses due to multi-mapping issue and thus these regions would appear to be “homozygous”. Moreover, repeat is known to facilitate recombination and thus lead to copy number gain or loss. Did authors mean that duplications or deletions would be associated with more SNPs? Anyway, this section needs to be clarified.

*Response: We have clarified this observation providing a better explanation on page 11, modifying the section to read as:*

*“The higher level of Pi observed within specific regions on several chromosomes – such as chromosomes 2, 11, and 18 – correspond closely to the regions we identified as containing large (>250 kb) inversions that appear to be shared across at least some of the MIKK panel (Additional file 4: Fig. S3). These regions are also enriched for large deletions and duplications (44). Inversions cause permanent heterozygosity (45), and duplications and deletions may have increased the density of called SNPs in these regions (46), so the observed depressions in homozygosity at these loci may be the result of such large structural variants that are present in the MIKK panel’s genomes”.*

2. “The reviewer makes an interesting observation about chromosome 3 - we have checked certain characteristic of chromosome 3 in detail, for example repeat content, relative number of coding genes and potential divergence from the reference genome however we have been unable to find any clear indication as to why it displays a lower level of homozygosity than most other chromosomes. “

I searched and found in Page 10: “In addition, chromosomes 2 and 3 showed higher levels of heterozygosity (see discussion) compared to the other chromosomes.”. I then searched the Discussion section for several times but couldn't find where authors discussed about this issue. Actually, if authors do not know why chromosome 3 behaves like this, they can simply acknowledge this limit in Discussion.

*Response: We have acknowledged that we do not fully understand why chromosome 3 shows an increased level of heterozygosity and have added information both on page 10 and in the discussion (page 22) highlighting some potential causes at chromosomes 2 and 18.*

3. “For the enrichment in LoF variation in chr2 and 18, we do not believe that chr2 and 18 encode less important genes however both chromosomes are enriched for repeat content and for inversions (Additional File 5 Figure 3 and 4) and we expect that the apparent LoF enrichment is a result of both complex repetitive genome content and a potentially higher mutation rate for these chromosomes.”

The revision corresponds to “interestingly both chromosomes 2 and 18 show consistently higher numbers of LoF variation for all LoF variant classes (Additional File 5 Figure 5 A-D) (see discussion).” Again, there is no relevant content in Discussion. Since the same mistake occurred twice, I sort of wonder whether the authors uploaded a wrong version of manuscript.

For the argument per se, repetitive regions lead to missing data due to the aforementioned multi-mapping issue. Similarly, the enrichment of LoF in chr2 or chr18 should be unrelated with mutation rate. The most likely scenario is that these LoFs are recessive due to inversions, i.e., they are shielded by permanent heterozygosity.

*Response: We have made this section clearer adding content both on page 13 and page 23 (discussion) potentially linking the observed increase in LoF to the higher number of structural variations observed in these chromosomes.*

4. “We investigated SV content - including inversions – using ONT data from the companion paper (Additional File 5 Figure 3), and observed large (>250 kb) inversions and other structural variants on specific regions of chr 2, 11 and 18, which correspond closely with the increased level of heterozygosity also observed in those regions.”

How about chr3? Did this chromosome also encode inversions?

*Response: We have added clarification that we do not fully understand why chromosome 3 shows a higher level of heterozygosity relative to other chromosome but have highlighted two specific smaller inversions that were detected in some MIKK panel lines on page 10, referring to the companion paper.*

5. Following the reviewers suggestion, we have added a paragraph in the Introduction section on sex determination and sex chromosomes in medaka and related Oryzias species, where we also cite a key review on Y chromosome evolution by Doris Bachtrog.

When describing Fig. 3, I suggest to recite this paper and state that the region with high heterozygosity could be the first evolutionary strata. In this way, readers can better understand this phenomenon.

*Response: Following the reviewer's suggestions, we have commented on the evolutionary aspect of the region of high heterozygosity and recited the review of Bachtrog as well as a relevant review on medaka sex chromosome by Schartl (page 13, end of sex determination section).*

Minor concern:

1. “Please accept our apologies for the lack of resolution in the mentioned figures - this has been resolved in the revised submission.”

Yes, the new figures indeed have higher resolution. However, I noticed another issue that font sizes for X- and Y-axis labels and legends are very small for almost all figures. In my computer screen, I need to enlarge several times to recognize them.

*Response: We have reviewed all figures and increased the font size for axis labels where needed.*

**Third round of review**

**Reviewer 2**

This version is better and I only have minor comments.

“We have acknowledged that we do not fully understand why chromosome 3 shows an increased level of heterozygosity and have added information both on page 10 and in the discussion (page 22) highlighting some potential causes at chromosomes 2 and 18.”

I again used the search function to locate where the change is. It seems that chromosome 2 was explained on page 21 rather than 22. In addition, I could not find where chromosome 18 was discussed. Please make sure that this issue has been covered.

“We apologize for the problems locating our revisions and would like to point out that we were asked by the editors to omit line numbers in the revised version. We have tried to give precise information on where we have made changes to the manuscript in this second revision.”

You can simply copy your revised text in the response letter as you responded to the first question, which could save me lots of time.

“We have made this section clearer adding content both on page 13 and page 23 (discussion) potentially linking the observed increase in LoF to the higher number of structural variations observed in these chromosomes. “

It seems to be page 22.

“Following the reviewer's suggestions, we have commented on the evolutionary aspect of the region of high heterozygosity and recited the review of Bachtrog as well as a relevant review on medaka sex chromosome by Schartl (page 13, end of sex determination section) “

It seems to be page 12. Later on, please double check the page numbers.

**Authors Response**

**Point-by-point responses to the reviewers’ comments:**

Reviewer #2:

This version is better and I only have minor comments.

*Response: We would like to thank the reviewer for this positive evaluation and have addressed the open issues.*

“We have acknowledged that we do not fully understand why chromosome 3 shows an increased level of heterozygosity and have added information both on page 10 and in the discussion (page 22) highlighting some potential causes at chromosomes 2 and 18.”

I again used the search function to locate where the change is. It seems that chromosome 2 was explained on page 21 rather than 22. In addition, I could not find where chromosome 18 was discussed. Please make sure that this issue has been covered.

*Response: We apologise for the error, chromosome 2 is indeed discussed on page 21 rather than page 22.*

*The revised text on page 21, end of second paragraph is as follows:*

*"We note the faster decay of LD in chromosome 2, which is consistent with the known high recombination rate seen across this chromosome relative to its physical distance (3.36 cM/Mb in chromosome 2 vs 1.02-2.76 cM/Mb for the other chromosomes) (58), possibly attributable to that chromosome’s high repeat content (Additional file 4: Fig. S4A). There are potential analogies with the high recombination frequency present across chromosome 19 in humans (66)."*

*Chromosome 3 is discussed on page 10, the revised text on page 10, first paragraph is as follows:*

*"In addition, chromosomes 2 and 3 showed higher levels of heterozygosity compared to the other chromosomes, which in the case of chromosome 2 may be due to many large structural variants including inversions. However in the case of chromosome 3 it is unclear what is causing this increased heterozygosity although we did detect two relatively small inversions at specific regions on chromosome 3 in some MIKK panel lines (44)."*

*Chromosomes 18, together with chromosomes 2 and 11 are discussed on page 11. The revised text on page 11, second paragraph is as follows:*

*"The higher level of π ̂ observed within specific regions on several chromosomes – such as chromosomes 2, 11, and 18 – correspond closely to the regions we identified as containing large (>250 kb) inversions that appear to be shared across at least some of the MIKK panel (Additional file 4: Fig. S3). These regions are also enriched for large deletions and duplications (44). Inversions cause permanent heterozygosity (45), and duplications and deletions may have increased the density of called SNPs in these regions (46), so the observed depressions in homozygosity at these loci may be the result of such large structural variants that are present in the MIKK panel’s genomes."*

“We apologize for the problems locating our revisions and would like to point out that we were asked by the editors to omit line numbers in the revised version. We have tried to give precise information on where we have made changes to the manuscript in this second revision.”

You can simply copy your revised text in the response letter as you responded to the first question, which could save me lots of time.

*Response: We would like to apologize once more for the inconvenience and have copied the revised texts to our answers to simplify matters*

“We have made this section clearer adding content both on page 13 and page 23 (discussion) potentially linking the observed increase in LoF to the higher number of structural variations observed in these chromosomes. “

It seems to be page 22.

*Response: Again, we apologise for this error. This point is indeed discussed on page 13 and 22. The revised text on page 13, end of first paragraph is as follows:*

*"Unsurprisingly, we see the expected increase in the number of LoF variants in heterozygous blocks for chromosome 1 due the sex determination region, and interestingly both chromosomes 2 and 18 show consistently higher numbers of LoF variation for all LoF variant classes (Additional file 4: Fig. 5A-D) which is likely a result of larger numbers of structural variation observed within these chromosomes (44)."*

*On page 22, third paragraph, the respective text is as follows:*

*"Small homozygous LoF variants (SNPs and INDELs) show a slight enrichment in regions of residual heterozygosity in the MIKK panel lines and on chromosomes that show higher levels of larger structural variation overall. This may indicate a purging of LoF variation in essential dosage-sensitive genes, although the observed enrichment is slight, which is likely due to the low chance of LoF variation occurring in essential genes across the 9 generations of inbreeding, given the very low rate of spontaneous mutations."*

“Following the reviewer's suggestions, we have commented on the evolutionary aspect of the region of high heterozygosity and recited the review of Bachtrog as well as a relevant review on medaka sex chromosome by Schartl (page 13, end of sex determination section) “

It seems to be page 12. Later on, please double check the page numbers.

*Response: The reviewer is correct, we have added the respective comments and citation on page 12, end of the first paragraph. The revised text is as follows:*

*"This region includes 17 genes in the HdrR reference genome and could be of interest for further mapping studies to investigate the precise location of the duplicated copy of the DMRT1 gene known to be critical in sex determination (47), and how the suppression of recombination creates hetereogametic sex determination. It has been suggested that this 0.5 Mb region with higher levels of heterozygosity corresponds to an early stage of Y-chromosome evolution (31) which could eventually result in a non-recombining mature sex-chromosome (30). We note that a small number of lines were homozygous across this region, consistent with occasional XX male sex determination seen in medaka (see discussion)."*
